# Length-independent structural similarities enrich the antibody CDR canonical class model

**DOI:** 10.1080/19420862.2016.1158370

**Published:** 2016-03-10

**Authors:** Jaroslaw Nowak, Terry Baker, Guy Georges, Sebastian Kelm, Stefan Klostermann, Jiye Shi, Sudharsan Sridharan, Charlotte M. Deane

**Affiliations:** a Department of Statistics, University of Oxford, Peter Medawar Building, Oxford, UK; b Doctoral Training Center, University of Oxford, Rex Richards Building, Oxford, UK; c Informatics Department, UCB Pharma, Slough, UK; d Roche Pharma Research and Early Development, Therapeutic Modalities, Roche Innovation Center, Penzberg, Germany; e Roche Pharma Research and Early Development, PRED Informatics, Roche Innovation Center, Penzberg, Germany; f Department of Antibody Discovery and Protein Engineering, MedImmune Ltd, Granta Park, Cambridge, UK

**Keywords:** Canonical class, clustering, complementarity determining regions, length-independent, loop modeling

## Abstract

Complementarity-determining regions (CDRs) are antibody loops that make up the antigen binding site. Here, we show that all CDR types have structurally similar loops of different lengths. Based on these findings, we created length-independent canonical classes for the non-H3 CDRs. Our length variable structural clusters show strong sequence patterns suggesting either that they evolved from the same original structure or result from some form of convergence. We find that our length-independent method not only clusters a larger number of CDRs, but also predicts canonical class from sequence better than the standard length-dependent approach.

To demonstrate the usefulness of our findings, we predicted cluster membership of CDR-L3 sequences from 3 next-generation sequencing datasets of the antibody repertoire (over 1,000,000 sequences). Using the length-independent clusters, we can structurally classify an additional 135,000 sequences, which represents a ∼20% improvement over the standard approach. This suggests that our length-independent canonical classes might be a highly prevalent feature of antibody space, and could substantially improve our ability to accurately predict the structure of novel CDRs identified by next-generation sequencing.

## Abbreviations and acronyms

CDR: Complementarity-Determining Region
PDB: Protein Data Bank
V-region: Variable region
HMM: Hidden Markov Model
RMSD: Root Mean Square Deviation
DTW: Dynamic Time Warping
UPGMA: Unweighted Pair Group Method with Arithmetic Mean
DBSCAN: Density-Based Spatial Clustering of Applications with Noise
OPTICS: Ordering Points to Identify the Clustering Structure
AUC: Area Under the Curve
ROC: Receiver Operating Characteristics

## Introduction

Standard antibodies are proteins with a Y-shaped configuration, composed of 2 chains, heavy and light. They are produced by the immune system to detect and act upon foreign molecules, which are also known as antigens. Antibodies are one of the most-studied protein types. Since the first antibody crystal structure was solved in the 1970s, the number of available structures has grown exponentially.^1^ This growth has been accompanied by a similar trend in sequence data,^2^ leading to the creation of several publicly available sequence databases that aim to collect and analyze the results of antibody sequencing experiments (e.g., Kabat database,^3^ IMGT/LIGM-DB,^4^ abYsis,^5^ VBASE2,^6^ DIGIT^7^).

The binding properties of an antibody are primarily determined by the sequence and structure of just 6 loops called complementarity-determining regions (CDRs). Three CDRs are found on the light chain (L1-L3) and 3 on the heavy chain (H1-H3). Due to the importance of the CDRs, substantial efforts have been made to characterize them. Comparison of the structures of antibodies showed that the non-H3 CDRs (L1, L2, L3, H1, H2) form only a relatively small number of shapes, referred to as canonical classes.^8^ A canonical class describes a set of loops that assume similar conformations, with the conformation being determined by the number and identity of the residues that constitute the loop and some residues in the framework region adjacent to the loop. The theory of canonical classes postulates that the class of a loop can be identified by the presence of a few “key” residues at particular positions.^8^ Thus, using canonical classes, it should be possible to predict the structure of a novel CDR, by classifying it using key features of its sequence. Since the original canonical class study of Chothia and Lesk,^8^ the clustering of non-H3 CDRs into canonical forms has been extended several times.^1,9-18^

The earliest clustering of CDR structures by Chothia and Lesk^8^ was performed with only 5 antibody structures and the comparison was done manually. In contrast, Martin and Thornton^13^ created a fully automatic method for classification of CDRs into canonical forms, first clustering the structures in torsional space and then merging the clusters using root-mean square deviation (RMSD). Martin and Thornton^13^ were also the first to note the limitations of the canonical model, in particular that sequence is not a perfect determinant of cluster membership. In the more recent study of North et al.,^17^ CDR structures were clustered in torsional space, using the affinity propagation algorithm. This clustering is available as an online database (http://dunbrack2.fccc.edu/PyIgClassify/).^19^

There have also been studies of canonical shapes that involved only a subset of available structures. Some analyzed only specific chains^12,20,21^ while others focused on individual non-H3 CDRs, in particular the CDR-L3.^22,23^ Apart from studies of the structural repertoire of non-H3 CDRs, substantial efforts have been made to understand the structural patterns of CDR-H3.^24-31^ In their work on CDR clustering, North et al.^17^ classified the anchor region of CDR-H3, defined as the first 3 residues and the last 4 residues of the loop, into clusters.

These studies of the structural repertoire of CDRs (and antibodies in general) have improved our ability to model antibody structure from sequence,^32,33^ added valuable insights into antigen recognition^13,15^ and inspired novel methods for antibody design.^10,34-37^

In the earliest clustering study Chothia and Lesk^8^ noticed that there are CDR loops that, despite differences in length, are more structurally similar to each other than to other CDR loops of the same length. The clustering method used by Martin and Thornton^13^ allowed for comparison between loops of different length, but all the clusters discovered by the authors contained CDRs of only a single length. Most of the later clusterings were performed under the assumption that CDRs of different length are structurally distinct. Here, we quantify the structural similarities between loops of different lengths and create a methodology to find length-independent structural clusters of CDRs. We show that these length-independent clusters contain a larger number of unique sequences and are better able to predict structure from sequence than their length-dependent counterparts.

The latter result emphasizes the fact that the structural relationships between different length CDRs are based on sequence patterns. Using our length-independent structural clusters, we identified the most common causes of similarity between loop structures of different lengths. We demonstrate the impact of our study by analyzing the cluster membership of CDR sequences from next-generation sequencing datasets. We show that by taking into account the structural similarities between loops of different length, we are able to classify significantly more CDR sequences into structural clusters.

## Results

The structures of CDR loops were extracted from antibody structures available in the SAbDab^1^ database and filtered as described in the Methods section. Using the structural alignment produced by the dynamic time warping (DTW) algorithm, we found that across all CDR types, in about 50% of cases the insertion site identified by Chothia alignment is structurally correct, and in about 77% of cases the correct site is within one residue of the Chothia site.

**Figure 1.**
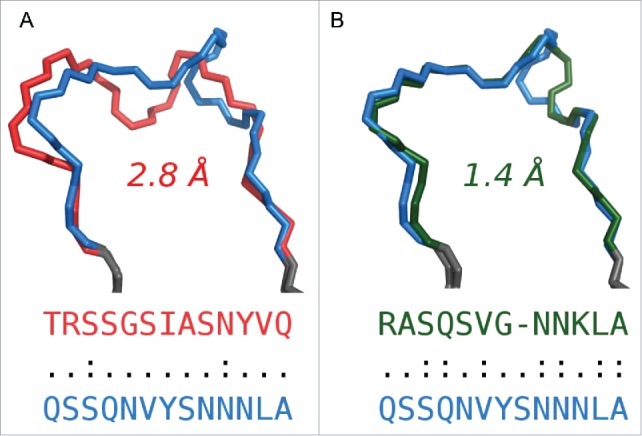
(A) Structure of CDR-L1 from 4JO2_M (blue, length 13) aligned with its closest structural partner of the same length, the CDR-L1 from 3BDX_A (red, length 13), which is 2.8 Å away, as measured using the DTW score. The loops have only 2 identical residues. (B) Structure of CDR-L1 from 4JO2_M (blue, length 13) aligned with its closest structural partner of different length, the CDR-L1 from 3LHP_M (green, length 12), which is 1.4 Å away, as measured using the DTW score. The loops have 7 residues in common. In both panels A and B the anchors of the CDRs are shown in gray.

Taking all the unique CDR sequences from our structural set, we identified the structurally closest loop to each using the DTW score (see Materials and Methods). In all CDR types, apart from CDR-L2, for some fraction of CDRs the structurally closest partner was of a different length (Table 1, Fig. 1). This result suggested that length-independent canonical classes could exist.

**Table 1.** Length-independent structural similarity. For each CDR type the Table shows: First row - number of CDR structures, after the filtering described in the Methods section was applied. This is also the number of structures that were used as input to our clustering method. Second row - number of unique CDR sequences. Third row - number of unique sequences for which the closest structural partner is of a different length. Fourth row - fraction of unique sequences for which the closest structural partner is of a different length.

CDR type	CDRL1	CDRL2	CDRL3	CDRH1	CDRH2	CDRH3
Number of structures	1701	1762	1752	1734	1779	1671
Number of unique sequences	455	302	518	374	493	614
Number of times the closest structure is of a different length	20	0	35	18	15	288
Fraction	4%	0%	7%	5%	3%	47%

Motivated by this result, we combined ideas from density-based and hierarchical clustering methods to create length-independent canonical classes. We used all CDR structures, regardless of sequence redundancy, as input to our clustering method (see Materials and Methods). Using the length-independent methodology, we discovered 17 large clusters in total, 4 of which contained CDRs of more than one length (for a cluster to be classified as large, it had to contain at least 6 unique sequences). The results for the large clusters are summarized in Table 2. For a detailed description of the clustering results please see the Supplementary Information (SI) *Clustering details* section.

**Table 2.** Information on CDR clusters that contain at least 6 unique sequences. The following nomenclature is used: 2 letters describing the CDR type, followed by a dash and the lengths of the CDRs contained within the cluster, separated by commas, followed by another dash and a capital letter describing the order of the cluster (e.g., L1–13,14-A corresponds to the first cluster containing CDR-L1 structures of lengths 13 and 14). The “middle structure” column shows the PDB ID and the name of the chain containing the CDR structure that is in the center of the corresponding cluster. The clusters are ordered first by length, then by number of structures and finally by number of sequences. For a detailed information on each cluster please see the SI Tables S1 – S5.

Cluster name	Length	Number of structures	Middle structure	Number of unique sequences
CDR-L1 (κ)				
L1–10,11,12-A	10, 11, 12	779	3SOB_L	204
L1–12-A	12	22	1HQ4_A	12
L1–15-A	15	55	3QRG_L	26
L1–16-A	16	273	1KFA_M	65
L1–17-A	17	113	2R1X_A	31
CDR-L1 (λ)				
L1–11-A	11	38	4IMK_C	9
L1–11-B	11	24	3MLS_M	8
L1–13,14-A	13, 14	117	4FQJ_L	37
L1–13-A	13	23	2WOL_C	6
L1–14-A	14	92	1YOL_C	7
CDR-L2				
L2–7-A	7	1708	2G5B_A	291
L2–7-B	7	21	3I9G_L	6
CDR-L3 (mixed λ and κ)				
L3–5-A	5	17	4JPI_B	6
L3–9-A	9	107	1Y0L_C	22
CDR-L3 (κ)				
L3–8-A	8	106	4HGW A	29
L3–9,10-A	9, 10	1133	3RVV_C	335
CDR-L3 (λ)				
L3–10,11-A	10, 11	53	3MLX_L	23
CDR-H1				
H1–7-A	7	1267	1PLG_H	357
H1–7-B	7	18	4FQQ_F	6
H1–8-A	8	37	3RVW_D	8
H1–9-A	9	86	3IDN_B	9
CDR-H2				
H2–7-A	7	387	3ZKM_H	91
H2–8-A	8	650	1I8M_B	197
H2–8-B	8	305	2VXS_K	93
H2–8-D	8	19	1YQV_H	9
H2–10-A	10	147	3HZV_B	25

We find that most of the large light chain clusters contain only either the κ or λ light chains. The two exceptions are L3-5-A and L3-9-A. The cluster L3-9-A has been described previously by North et al.^17^ (as the cluster L3-9-1). The cluster L3-5-A contains structures that were not available at the time the work of North et al. was published, and are all from broadly neutralizing antibodies, suggesting that such loops tend to take a similar shape, irrespective of the chain type.

We use the following nomenclature for our clusters: 2 letters describing the CDR type, followed by a dash and the lengths of the CDRs contained within the cluster, separated by commas, followed by another dash and a capital letter describing the order of the cluster (e.g., L1-13,14-A corresponds to the first cluster containing CDR-L1 structures of lengths 13 and 14).

### Sequence patterns in length-independent clusters

**Figure 2.**
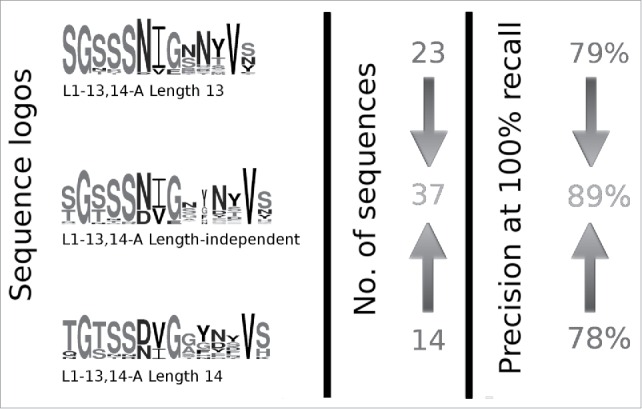
An illustration of how length-independent clustering improves the precision of prediction. The first column shows logos created using sequences of CDRs of length 13 (top) and 14 (bottom) inside cluster L1-13,14-A, with the logo for the complete length-independent cluster in the middle. The second column shows the number of sequences of each length (top and bottom) and the number of sequences in the complete length-independent cluster (middle). In the third column the precision at 100% recall is reported for the complete cluster (middle) and for the 2 length-dependent clusters resulting from splitting L1-13,14-A by length (top and bottom).

For the concept of length-independent structural similarity to be useful in loop modeling, the structural relationships between CDRs of different length must be matched by sequence similarity. To investigate whether the length-independent clusters contain clear sequence patterns, we compared the performance of a prediction method to the length-dependent version of our clustering (see Materials and Methods). We find that the increased number of sequences in the length-independent clusters improves the precision of prediction. Fig. 2 illustrates this principle with the example of CDR-L1 cluster L1-13,14-A, which contains λ CDRs of length 13 and 14. If the cluster is split by length, prediction precision decreases. There are clear similarities between the sequence logos of CDRs of length 13 and length 14, especially the presence of Asn/Asp at Chothia position 29, which appears to be key for maintaining the structures of the loops in this cluster.

The importance of consistent sequence patterns is further illustrated by the CDR-L3s of length 10, which are part of the cluster L3-10,11-A. These CDRs have no close structural homologs among the other CDR-L3s of length 10 and, in the length-dependent version of the clustering, are not clustered. In the length-independent version of the clustering, they are part of the cluster L3-10, 11-A, which contains primarily CDRs of length 11.

To assess the global performance of the prediction method on our clusters, we plotted receiver operating characteristic curves for each CDR type (see SI Figs. S6-SB). The area under the curve (AUC) for each CDR type was above 0.90 (a perfect model would get an AUC score of 1 while a random predictor would receive a score of 0.5).

We show in the next section how our clustering improves predictions in the context of next-generation sequencing (NGS) of CDR-L3 repertoire.

### Analysis of next-generation sequencing data

Given that the length-independent clusters contain such clear sequence patterns, making them useful for prediction, we investigated whether the small gains in prediction coverage shown in the structural set have a significant effect when considering the large next-generation sequencing (NGS) sets of CDR-L3 sequences. We examined 3 large antibody NGS datasets: the first dataset was created through sequencing experiments performed by UCB Pharma Ltd and contains over ∼9,000,000 human light chain sequences; the second dataset was obtained by DeKosky et al. in 2015^38^ and contains 198,148 human paired CDR-H3 - CDR-L3 sequences from 3 donors; and the third dataset was extracted from the DIGIT database^7^ and consists of 71,404 light chain sequences from over 100 different species. Since only the CDR-L3 sequences were available in all datasets, we extracted the unique sequences of this type, obtaining ∼1,000,000 sequences from the UCB dataset, 72,045 from the DeKosky et al. dataset and 12,960 from the DIGIT data set.

We found that the length-distribution of CDR-L3 sequences in these datasets differs significantly from the length distribution of CDR-L3s whose structure is known (see SI Figs. S3-S5). For example, sequences of length 10 comprise ∼26% of the UCB dataset (290,000 sequences) and only ∼6% of the SAbDab database. A major reason for this disparity is the relative abundance of κ chains in the structural dataset in comparison to the NGS dataset. The structural dataset consists of about 78% κ light chains and 22% λ light chains, while a more balanced distribution of 47% κ chains and 53% λ chains is observed in the NGS dataset (which contains only human sequences). Nevertheless, even after separating the CDR-L3 sequences by the chain type, we still observe that the sequences of length 9 are overrepresented and sequences of length 10 underrepresented in the structural dataset. Due to this disparity, the canonical class assignment would be more difficult if performed in a length-dependent way.

**Figure 3.**
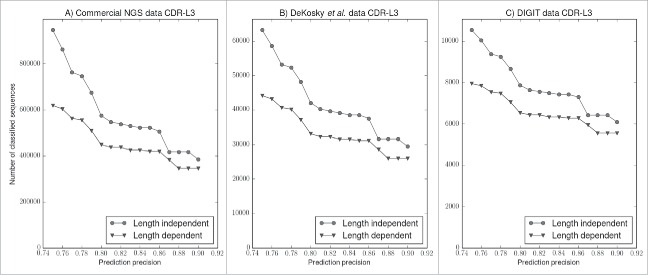
Length-independent clusters increase the number of sequences that can be classified. The expected precision of prediction (x axis) was calculated from our structural data based on the HMM score returned by HMMER.^39^ The circles show the number of sequences that can be classified using our length-independent approach, while the triangles show the number of sequences that can be classified by the length-dependent approach. (A) The classification of ∼1,000,000 unique CDR-L3 sequences from the UCB dataset. At 0.8 precision we can classify 125,000 or about 21% more sequences into clusters. (B) The classification of 72,045 CDR-L3 sequences from the DeKosky et al.^38^ dataset. At 0.8 precision we can classify 8,958 or ∼21% more sequences into clusters. (C) The classification of 12,960 CDR-L3 sequences from the DIGIT^7^ dataset. At 0.8 precision we can classify 1,338 or ∼17% more CDR-L3 sequences into clusters.

To test whether we can assign more sequences to clusters using the length-independent methodology, we evaluated the cluster membership of the unique CDR-L3 sequences in both a length-dependent and length-independent way at expected precisions between 75% and 90% (Fig. 3). Precision of cluster membership assignment was estimated using the structural data and the HMM scores returned by HMMER^39^ (see Materials and Methods). We found that across all 3 datasets we can predict more sequences using the length-independent approach. For example, at 80% precision, we can assign into clusters an additional ∼125,000 sequences (∼21% improvement, Fig. 3A) from the UCB dataset, 8,958 sequences (∼21% improvement, Fig. 3B) from the DeKosky et al. dataset and 1,338 sequences (∼17% improvement, Fig. 3C) from the DIGIT dataset. Together, these results illustrate that using length-independent clustering we can structurally characterize a much larger part of antibody sequence space.

### Reasons for length-independent structure similarity

Because our length-independent clusters show strong sequence patterns, we investigated the possible causes of similarity between CDR structures of different lengths. We propose 3 natural mechanisms for the generation of structurally and sequence similar CDRs of different lengths.

**Figure 4.**
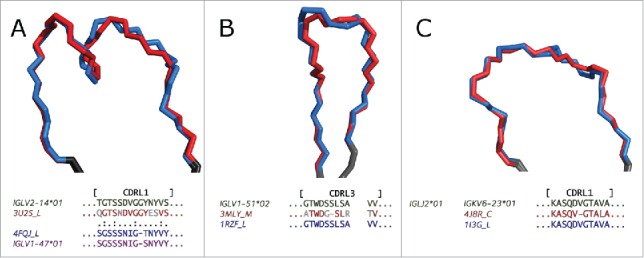
CDRs with different lengths, but similar structures, with their anchors aligned, shown in gray. This Figure demonstrates how length-independent shape similarity may arise. (A) CDR-L1 of 3U2S_L (length 13, red) and 4FQJ_L (length 14, blue). The two CDRs are coded for by human germlines from different subgroups (IGLV2-14*01 and IGLV1-47*01 respectively), but the identity of certain key residues results in a similar shape. Especially important seems to be the presence of Asp/Asn at Chothia position 29. (B) CDR-L3 of 3MLY_M (length 10, red) and 1RZF_L (length 11, blue). The two CDRs have similar structures and appear to be coded for by the same human V-gene (IGLV1-51*02) and human J-gene (IGLJ2*01). The observed length difference seems to be caused by different rearrangement of genes during VJ recombination. (C) CDR-L1 of 4J8R_C (length 10, red) and 1I3G_L (length 11, blue). This is an example of 2 structurally similar CDRs that appear to come from the same murine germline (IGKV6-23*01), but in the case of 4J8R_C an Asp has been deleted during somatic hypermutation.

Firstly, the germline contains a large repertoire of V-region genes.^6^ One of the causes of similarity between structures of different lengths appears to be the identity of certain key residues, common between different germlines (see Fig. 4A).

Secondly, in the early stages of development the antibody-producing B cells undergo a somatic recombination, during which V (variable), J (joining) and, in the case of the heavy chain, D (diversity) gene segments are randomly spliced together. This results in a novel sequence for the variable domain of the antibody. The VJ recombination affects the sequence of CDR-L3, which explains why CDR-L3 is more variable than the other light chain CDR types.^40^ We have found that the different rearrangements of the V and J genes may not always result in a significant change to the CDR structure, which could lead to shape similarity between CDR-L3 loops of different lengths (Fig. 4B).

Thirdly, B cells proliferate when they are stimulated by antigens. During this proliferation, the V-region coding sequences of both heavy and light chain accumulate point mutations at a rate that is about a million times greater than in other genes.^41^ The few mutated B cells, which express antibodies with higher affinity, are further stimulated to proliferate. This process, which is called somatic hypermutation, can result in a 1000-fold increase in affinity to the target.^42^ During the hypermutation phase, deletions and insertions may arise, although they are far less common than substitutions.^43,44^ The change in sequence length generated by somatic hypermutation may result in 2 CDRs having similar structure, despite being of different length. A possible example of this is shown in Fig. 4C.

Assuming that the human germline repertoire contains ∼40 functional variable genes of each type (heavy, λ, κ), 5 functional joining genes of each type, 23 functional diversity genes, and that the N-diversity and somatic hypermutation increase the number of possible light and heavy chain sequences by about 1000-fold, we can estimate that the human organism can produce about 10^12^ distinct antibodies. The fact that we observe length-independent structural similarities in the limited number of antibody crystal structures available to us suggests that it may be a relatively common occurrence in nature.

### Heavy chain complementarity-determining regions

Despite the indication that the natural antibody diversity-generating processes are a major reason for the observed length-independent structural clusters, we did not find any length-variable clusters in the heavy chain CDRs. Here, we describe the clustering results for CDR-H1 and CDR-H2 in more detail and discuss the possible reasons behind the apparent lack of length-independent structural similarities.

The CDR-H1 loops are 3 to 13 residues long. The majority of structures (87%) are of length 7. There are 14 clusters in total, but virtually all human and mouse CDRs of this type are concentrated in the 4 largest clusters (H1-7-A, H1-7-B, H1-8-A, H1-9-A). The observed length and structural variability seems to come mostly from the structures of the Camelid antibodies, which are composed of only the heavy chain.^37^

The length diversity of CDR-H2 is relatively low – only loops of length between 7 and 12 residues are observed in our structural dataset. Most CDR structures, including the Camelid ones, are contained within the 5 largest clusters (H2-7-A, H2-8-A, H2-8-B, H2-8-D and H2-10-A). The structures of loops in clusters H2-7-A and H2-8-A are similar, but not enough to belong to the same cluster.

Previous analyses of CDR structures^17,45^ discussed how the framework residue at Chothia position 71 influences the conformation of CDR-H2. We analyzed the amino acid distribution of residue 71 across our large clusters (shown in SI Fig. S2) and found that the framework sequences in clusters H2-8-B and H2-10-A show a clear preference for Arg at this position, in agreement with previous work.^17,45^ We also find that, compared to previous work, the framework sequences in cluster H2-8-A show an increased abundance of Arg at position 71 (∼5% in equivalent North et al. cluster H2-10-1, ∼10% in H2-8-A), making the residue less predictive of cluster membership.

### Comparison to previous clusterings

As we noted above, many length-dependent clusterings of CDR structures have previously been reported. In this section, we describe the differences between our clustering and a recent clustering of CDRs into length-dependent canonical classes by North et al.^17^ Tables containing the full comparison are given in SI Tables S6-S10.

The large clusters (those containing at least 6 unique sequences) map well from our work to North et al.,^17^ usually having a one-to-one correspondence. Some clusters, however, are split or joined due to differences in methodology or length-independence. For example, loops of length 11 from our cluster L1-10,11,12-A are split into 2 clusters L1-11-1 and L1-11-2 in the work of North et al.^17^ This cluster is split by North et al.^17^ due to a change in conformation of a single residue at position 30. This does not lead to a large RMSD between the loops, but leads to a large change in dihedral angle, and, as North et al.^17^ cluster in dihedral space, the length 11 CDRs in L1-10,11,12-A are split into L1-11-1 and L1-11-2. The opposite effect can be seen for our clusters L1-11-A and L1-11-B. The central L1 loops of these 2 clusters (from 4IMK_C and 3MLS_M, respectively) are 1.5 Å apart, but are considered close enough in dihedral space to belong to North et al.^17^ cluster L1-11-3. Some clusters are split in North et al.^17^ due to our length-independent approach. For example our cluster L1-13,14-A is split by length into L1-13-1 and L1-14-2 in North et al.^17^

The smaller clusters (containing less than 6 unique sequences) map less well and there is usually no corresponding cluster in our work to match the cluster in North et al.^17^ One further difference between our work and that of North et al.^17^ is that North et al. used a non-redundant CDR set, filtering out the structures of the same antibody solved multiple times. We observed that these identical sequences can have structures with significantly different loop conformations (e.g., CDR-L1 loops with sequence TGTSSDVGGYNYVS, have been structurally characterized multiple times as part of the structures 1MCB, 1MCC, 1MCD, 1MCE, 1MCF, 1MCH, 1MCI, 1MCJ, 1MCK, 1MCL, 1MCN, 1MCQ, 1MCR, 1MCS,^46^ and are found in conformations differing by over 1.5 Å between different PDB IDs). Therefore, we made a decision to include all CDR structures, regardless of sequence redundancy. By doing so we avoid picking a structure that is non-representative due to crystal packing, or mistakes in solving the structure.^18^ This approach also allowed us to observe CDR sequences that can exist in 2 canonical states (see SI). However, it will also reduce our ability to predict conformations as an identical sequence could be found in 2 different structural clusters.

## Discussion

We analyzed structural similarities between CDRs of different lengths and used them to generate length-independent structural clusters. Compared to the commonly used length-dependent approach, we generate a smaller number of clusters, containing more unique sequences. This improves our ability to classify CDRs into clusters by sequence alone.

Given that for a portion of CDRs the most similar available structure is one of a different length, and such structural similarity is usually matched by sequence similarity, developing CDR modeling methods that utilize this information should significantly improve prediction accuracy.

We have described how natural antibody affinity maturation processes can produce CDRs having different lengths, but similar structure. Since the probability of these processes generating insertions and deletions is relatively low, the length-independent structural similarities are likewise infrequent. Nevertheless, we believe that as new antibodies' crystal structures become available, length-variable clusters will become a more common occurrence.

We tested our method on 3 large NGS datasets of CDR-L3 sequences and found that our length-independent methodology can classify ∼135,000 or ∼20% more sequences into clusters than standard techniques. We also observed significant differences in distribution of CDR-L3 lengths between the structural dataset and the NGS datasets. This disparity, together with the imbalance between λ and κ chains in the structural dataset, is a major obstacle toward increasing the structural coverage of human antibody sequence space.

## Materials and methods

### Choice of CDR definition

For this study, we used the Chothia definition of CDR loops^8^ for all CDR types except for CDR-H2, where 2 residues before the N-terminus were also included. This choice was made as we tested if extending Chothia defined CDRs by up to 3 residues at either end would change the clustering results, especially the prediction accuracy (*see cluster prediction from sequence section*). A change in length only made a statistically significant change to the results for CDR-H2, where it improved prediction accuracy. The resulting boundaries of each CDR in Kabat-Chothia numbering are as follows: CDR-L1: 24–34, CDR-L2: 50–56, CDR-L3: 89–97, CDR-H1: 26–32, CDR-H2: 50–56, CDR-H3: 95–102.

### Data selection

The dataset was built from the 1833 antibody PDBs (www.rcsb.org) ^47^ available in the SAbDab database as of September 2014 (http://opig.stats.ox.ac.uk/webapps/abdb/web_front/Welcome.php).^1^ Antibody structures solved using methods other than X-ray crystallography and those solved with a resolution above 2.8 Å were removed from the dataset. Structures of CDR loops were extracted from the remaining PDBs along with their anchors, 5 residues before the N-terminus and 5 after the C-terminus. CDR structures were removed from the dataset if they had atoms missing from the loop or anchor region or if they contained backbone atoms with B-factors above 80 or equal to zero. Loops with identical sequences resulting from solving the structure of the same antibody multiple times were not removed because they can have different structures.

We use the following nomenclature for our structures: 4 letters for the PDB code of an antibody, followed by underscore and the chain identifier (e.g., 7FAB_L corresponds to chain L of the antibody with PDB code 7FAB).

### Similarity calculations

Initially, the anchors of all CDRs of a type (e.g., L1) were superposed,^48^ regardless of length (superposing the anchors reflects how the loops are oriented with respect to the rest of the antibody). To calculate the structural similarity score between CDRs, we used the DTW algorithm.^49^ The algorithm uses dynamic programming to find the optimum path through the low-cost areas of a cost matrix.^50^ When 2 loops of the same length are compared, the algorithm returns the RMSD between the backbone atoms of the loops. When two loops of different lengths are compared, the algorithm calculates the RMSD between backbone atoms of residues matched by the walk through the cost matrix (the method is analogous to the Needleman–Wunsch algorithm for sequence alignment,^51^ except that the scores are calculated from RMSD between backbone atoms of the residues, instead of being taken from a sequence similarity matrix).

All images of CDR structures were generated using program PyMOL.^52^

### The clustering pipeline

To ensure that the discovered clusters reflect all the underlying structural and sequence patterns, the CDRs were first clustered using the DTW score as a distance measure between structures and the Unweighted Pair Group Method with Arithmetic Mean (UPGMA)^53^ algorithm with a cutoff of 1.5 Å. Next, the ability to predict canonical forms from sequence was assessed using Hidden Markov Models (HMM) (*see cluster prediction from sequence section* Finally, the canonical forms that contained more than 6 unique sequences, but could be predicted with less than 75% precision and 25% recall were re-clustered using Density-Based Spatial Clustering of Applications with Noise (DBSCAN),^54^ choosing the optimal parameter using the Ordering Points to Identify the Clustering Structure (OPTICS)^55^ algorithm (once again using the DTW score as a distance measure). The choice of 6 sequences was made because the prediction results were unreliable in smaller clusters. The resulting parameters are shown in Table 3. This re-clustering with DBSCAN and OPTICS was performed in order to ensure that every cluster was both structurally coherent and, if the data allowed it, sequence coherent.

**Table 3.** The parameters for DBSCAN algorithm for each non-H3 CDR type. In the case of CDR-L2 the UPGMA clustering was deemed sufficient.

	Distance cut-off
CDR-L1	0.82 Å
CDR-L2	–
CDR-L3	0.91 Å
CDR-H1	0.80 Å
CDR-H2	0.63 Å

In order to ensure there is no drop in accuracy, we cross–validated our length-independent clustering against a length-dependent version, created using the same methodology, parameters and validation methods. Using the HMMER predictor, the True Positive Rates (TPRs) and False Positive Rates (FPRs) were calculated across a range of different HMM score thresholds for each cluster. The TPRs and FPRs were macro-averaged across our clusters and used to plot Receiver Operating Characteristic (ROC) curves, separately for each non-H3 CDR type and, in case of CDR-L1 and CDR-L3, separately for the length-independent and the length-dependent version. To measure the statistical significance of the difference between the length-independent and the length-dependent ROC curves, 1,000 bootstrap replicates were sampled from the TPR and FPR data and the Area Under the Curve was calculated for each ROC replicate. The resulting mean and standard deviation were used to calculate p-values of the difference in AUC. It was found that there is likely no difference between the curves (the p-values were 0.48 and 0.07 for CDR-L1 curves and CDR-L3 curves, respectively). The ROC curves for all clusters and the comparisons between length-dependent and length-independent versions for CDR-L1 and CDR-L3 are shown in the SI Figs. S6-S8.

### Cluster prediction from sequence

To predict canonical forms from sequence, the leave-one-out cross-validation procedure was followed. First, the identical CDR sequences were removed from each cluster. Then, one sequence was selected at random and removed from each cluster. Hidden Markov Models (HMMs) were constructed for each cluster from the remaining data using the program HMMER 3.0.^39^ Finally, background distribution HMMs were built for each cluster from all sequences outside of the cluster (to use a custom background distribution, rather than the one hardcoded in HMMER, the HMMER source code was modified to return the ”raw” log-likelihood rather than the score with the background distribution already subtracted). The selected sequences were scored against the clusters that contained sequences of the same length and assigned to the cluster with which they scored the highest (one-vs-all classification). The procedure was repeated until all sequences had been classified. A similar procedure was followed to score the sequences of loops in clusters containing less than 6 unique sequences and for loops falling outside the clusters, but in those cases the complete sequence data was used to create HMMs for the large clusters.

To visualize the sequence patterns of the CDR clusters, used as input to our HMMs, we generated sequence logos, using the Weblogo software package (http://weblogo.berkeley.edu/).^56^ The sequence logos for the clusters containing at least 6 unique sequences are shown in the Supplementary Information.

### Genetic data

Species and germline data were extracted from the IMGT database (International ImMunoGeneTics information system®, http://www.imgt.org)^57^ and from the SAbDab^1^ database when the respective IMGT entry was not available. If there was a discrepancy between the species annotation in the IMGT record and the PDB file header, or if a human germline was reported for a CDR belonging to a cluster containing primarily mouse antibodies (or vice versa), the article associated with the PDB entry was inspected to learn the origin of the CDRs.

## Supplementary Material

Supplemental_Datas.zip
