# Supplementary material for: Length-independent structural similarities enrich the antibody CDR canonical class model
Source: MAbs. 2016 Mar 10;8(4):751–60. doi: 10.1080/19420862.2016.1158370 (PMC4966832; doi:10.1080/19420862.2016.1158370)
Supplement: Supplemental_Datas.zip [file kmab-08-04-1158370-s001.zip › 2015MABS1071R-s01.pdf]

Clustering details

Here we describe in detail the clustering results for each non-H3 CDR type. We include descriptions of interactions that stabilize the loop structures and describe sequence patterns. The clusters are ordered first by length of the shortest loop, then by number of structures and, finally, by number of sequences. The clustering results are summarized in Tables S1-S5.

1. CDR-L1 clusters

CDR-L1 is the second most length variable of all CDR types, behind CDR-H3. The shortest loops of this type are of length seven and the longest of length 17. There are 17 clusters in total, 10 containing at least six sequences, two of which contain loops of more than one length (Table S1).

The cluster L1-10,11,12-A contains the majority of loops of length 10 and 11 and three loops of length 12 (Figure S1A). The most common interaction, present in 194 out of 204 sequences in this cluster, is the hydrogen bond formed between a Ser at position 26 and the backbone of the residue at position 3. In addition, all CDRs in this cluster contain a hydrophobic residue at position 33, the side chain of which is buried inside the loop structure.

The second largest cluster of CDR-L1s of length 11 is L1-11-A, coded for by germlines from the lambda Immunoglobulin locus. Loops in this cluster do not have a Ser at position 26; instead all loops in this cluster display an interaction between residues at positions 27 and 30 creating a more ”compact” structure that is aided by presence of two Gly (present in all loops in this cluster) at positions 25 and 29.

The third cluster containing loops of length 11 - L1-11-B contains structures which in all cases are stabilized by the hydrogen bond between the backbone oxygen of Leu at Chothia position 28 and the backbone nitrogens of residues 25 and 31.

Many of the length 12 CDR-L1s not in cluster L1-10,11,12-A are found in cluster L1-12-A which consists of 12 unique sequences. In 11 of the 12 unique sequences in this cluster, there is a hydrogen bond between the sidechain of the residue at position 30 and the backbone of residue 31, an interaction not present in L1-10,11,12-A.

Most unique sequences of length 13 and 14 belong to cluster L1-13,14-A, which forms a compact shape stabilized by a number of interactions. All of the CDRs in this cluster form hydrogen bonds between the backbones of residues 26 and 29, 26 and 30A, and 29 and 30B (Figure S1B). The loops in clusters L1-13-A and L1-14-A, which contain the remaining structures of length 13 and 14, respectively, lack the aforementioned interactions which results in a more extended shape without the ”hoop” in the centre of the CDR.

The majority of the longer CDR-L1 loops are contained in clusters L1-15-A, L1-16-A and L1-17-A. The first part of long CDR-L1 loops, up to the central hydrophobic residue at position 29, resembles the loops in cluster L1-10,11,12-A. The remaining part of the loop structure is a protrusion of varying length stabilized by hydrogen bonds between backbone atoms (see Figure S1C).

2. CDR-L2 clusters

CDR-L2 is the least variable in terms of length of all CDRs, having only two lengths in our set - seven or 11. There are five clusters and only two which contain more than six unique sequences (Table S2).

The vast majority of the CDR-L2s of length seven are in cluster L2-7-A. This shape occurs universally across all available species and in both κ and λ light chains. The second largest cluster is L2-7-B, also containing loops of length seven. The structures of the CDRs in the two clusters differ only by the conformation of the first three residues. The two clusters containing CDR-L2s of length 11, L2-11-A and L2-11-B, are very small and contain only two and three sequences respectively. L2-11-B is more diverse of the two containing two loops from Rhesus Monkey anti-HIV antibodies and a CDR-L2 from a human pre B-cell receptor.

3. CDR-L3 clusters

CDR-L3 loops in our set span lengths between five and 12 residues. The majority of CDR-L3 loops are of length nine. There are 11 clusters in total, five containing at least six unique sequences, two of which contain loops of more than one length (Table S3).

All CDR-L3 loops of length five are found in cluster L3-5-A and are all from HIV-1 neutralizing antibodies that recognize the epitope of a CD4 binding site[1].

The cluster L3-8-A contains the majority of loops of length 8. The structure of these loops is stabilized by Gln at Chothia position 90 forming hydrogen bonds with residues at positions 92 (in 97 out of 106 cases), 93 (in 91 out of 106 cases) and the side-chain of the Thr at position 97 (in 86 out of 106 cases) (Figure S1D).

L3-9,10-A contains the largest number of CDR-L3 loops and contains loops of length nine and ten. Most loops of length nine belong in this cluster. The loops in this cluster are stabilized by the same interactions as the loops in cluster L3-8-A the difference being that 325 out of 335 of loops of length nine contain a Pro in cis conformation at position 95 and all loops of length ten contain two Pro at positions 95 and 95A. The Pro create a sharp turn making the loop conformations similar for loops of both lengths (Figure S1E).

Cluster L3-9-A is the second largest cluster containing loops of length nine (107 structures, 22 sequences). The residue at position 91 usually contains a large side-chain with an aromatic ring (Trp, Tyr, Phe). The CDR conformation is stabilized, in 20 out of 22 cases, by a hydrogen bond between backbone carboxyl oxygen of residue 92 and backbone nitrogen of residue 94.

Figure S1F illustrates the differences in conformations between CDR-L3 loops of length nine in cluster L3-9,10-A and in cluster L3-9-A.

The structures of CDR-L3 loops that are part of the cluster L3-10,11-A are stabilized by similar interactions to the CDRs in cluster L3-9-A. The difference is the presence of a highly conserved Asp/Asn forming a hydrogen bond with the residue at position 94 creating a bend in the structure (Figure S1G). In all CDRs in this cluster there is a hydrogen bond between backbone nitrogen of residue at position 92 and the backbone oxygen of residue at position 95. In 17 out of 23 sequences, there is also a hydrogen bond between backbone nitrogen at position 93 and the backbone carboxyl oxygen of residue 28, which is a part of the CDR-L1 loop.

4. CDR-H1 clusters

CDR-H1 loops are three to 13 residues long. CDRs of length seven are most prevalent (87% of all structures). There are 14 clusters, but out of these only four contain at least six unique sequences. There are no clusters containing loops of more than one length. Overall, this CDR type contains the largest number of small clusters, mostly coming from camelid antibodies (Table S4).

The largest cluster of CDR-H1 is H1-7-A containing 73% of all H1 structures. CDRs in this cluster tend to include (221 out of 257 sequences) a residue with a large aromatic ring at position 27 (Phe or Tyr) and, in 218 out of 257 of cases, a hydrogen bond between backbone atoms of residues 28 and 31.

The second cluster containing CDRs of length seven is H1-7-B. The difference in conformation between CDR-H1 loops in H1-7-A and H1-7-B is subtle, but most pronounced around residues 28 and 31.

The other two large clusters are H1-8-A and H1-9-A, containing CDRs of length eight and 10, respectively. All structures in cluster H1-8-A contain a hydrogen bond between the backbone atoms of residues 28 and 31. The structures of loops in cluster H1-9-A are similar to H1-8-A, except for a large “bulge” around residue 32. All CDR structures in cluster H1-9-A contain a bond between backbone atoms of residues 31 and 32.

5. CDR-H2 clusters

CDR-H2 loops in our set show only six lengths, between seven and 12 residues. Most of the CDRs of this type are of length seven, eight or 10. There are 12 clusters in total, five containing at least six unique sequences (Table S5). There are no clusters containing loops of more than one length but the conformations in clusters H2-7-A (containing CDRs of length seven) and H2-8-A (containing CDRs of length eight) are similar.

The majority of CDR-H2s of length seven belong to cluster H2-7-A. The sequence pattern for this cluster shows a preference for Gly at position 55 and a residue with side-chain oxygen at position 56 (Ser/Thr/Asn/Asp). In almost all structures (90 out of 91) there is a bond between residues at positions 52 and 55.

There are two clusters containing loops of length eight - H2-8-A and H2-8-B. In cluster H2-8-A, 195 out of 197 structures contain a bond between backbone atoms of residues 52 and 55 (same as H2-7-A). In addition, 154 loops contain a Pro at position 52A and a Gly at position 56. The structures of CDRs in this cluster are similar to H2-7-A with the Pro-52A creating a sharp turn in the loop.

In contrast, the CDR structures in cluster H2-8-B lack the Pro-52A and the hydrogen bond between backbone atoms of residues 52 and 56. Instead, 92 out of 93 structures contain a hydrogen bond between residues 52 and 54, and, in 76 cases a hydrogen bond between side-chain of residue 52 and residue 56. In 84 cases, the conformation in cluster H2-8-B is also stabilized by the hydrogen bond between the side-chain nitrogen of the framework residue Arg-71 and the backbone oxygen of residue at position 52. The importance of the identity of residue 71 for the conformation of CDR-H2 has been described before[2,3].

Most of the CDR-H2 loops of length 10 are concentrated in cluster H2-10-A. Loops of this length are usually from mouse antibodies. All structures in this clusters contain hydrogen bonds between residues 52A and 55 and 52B and 54. The structures of loops in this cluster in 23 out of 25 cases also display the interaction between Arg-71 and the backbone carboxyl oxygen of residue at position 52.

As discussed in the main text it is possible for CDRs of the same sequence to belong to different clusters, this structural variation is most common in CDR-H2. For example, the CDR-H2 loop with sequence EILPGSGS in the unliganded structure 1MLB_B belongs to cluster H2-8-A but in the bound form, in structures 1MLC_B and 1MLC_D, the loop belongs to cluster H2-8-D with 2.1 Å difference in RMSD (1MLB are 1MLC are crystal structures of the same antibody). YIWPSGGN is the sequence of CDR-H2 loops in structures 3HI6_X and 3HI6_H and in 3HI6_X the loop belongs to cluster H2-8-B but in 3HI6_H the loop is part of cluster H2-8-H. This result implies there are CDRs which can exist in different structural states. Unfortunately, it will also confuse cluster prediction from sequence.

References

1. Wu X, Zhou T, Zhu J, Zhang B, Georgiev I, Wang C, Chen X, Longo NS, Louder M, McKee K, et al. Focused evolution of HIV-1 neutralizing antibodies revealed by structures and deep sequencing. Science (80-) [Internet] 2011; 333:1593–602. Available from: http://www.sciencemag.org/content/333/6049/1593.short;
2. North B, Lehmann A, Dunbrack Jr RL. A new clustering of antibody CDR loop conformations. J Mol Biol [Internet] 2011; 406:228–56. Available from: http://www.sciencedirect.com/science/article/pii/S0022283610011496;
3. Tramontano A, Chothia C, Lesk AM. Framework residue 71 is a major determinant of the position and conformation of the second hypervariable region in the VH domains of immunoglobulins. J Mol Biol [Internet] 1990; 215:175–82. Available from: http://www.sciencedirect.com/science/article/pii/S0022283605801020

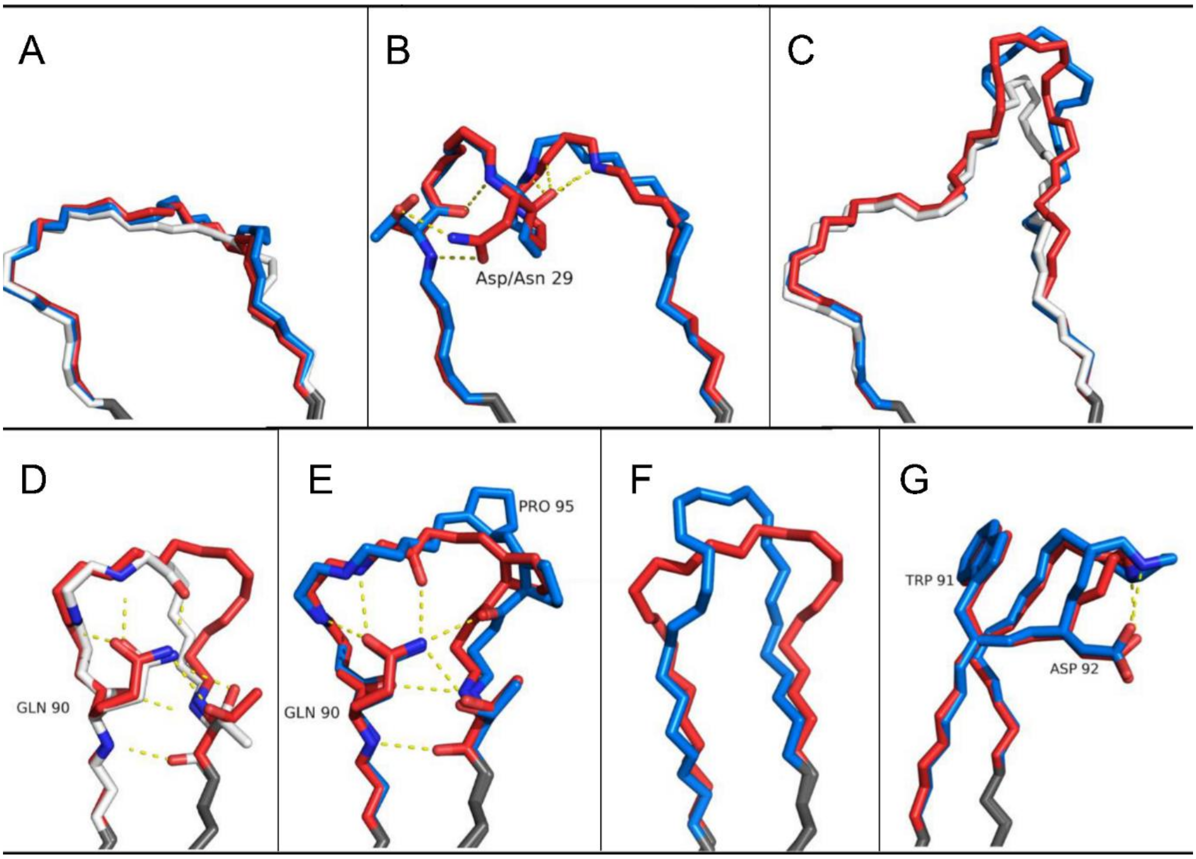

Figure S1: CDRs with different lengths, but similar structures, with their anchors aligned, shown in grey. A) CDR-L1 of 4F33_E (length 10, white), 3SOB_L (length 11, red) and 3EO0_A (length 12, blue) from cluster L1-10,11,12-A. The three CDRs are structurally similar, except for the insertion site at position 30. B) CDR-L1 loops of 4FQJ_L (length 13, red) and 3U2S_L (length 14, blue) from cluster L1-13,14-A. Presence of Asp/Asn at position 29 (the side chain of which is shown in the figure, along with the hydrogen bonds it makes) creates a ”hoop” in the structures of both lengths in cluster L1-13,14-A. C) CDR-L1 of 3QRG L (length 15, white, cluster L1-15-A), 1KFA M (length 16, red, cluster L1-16-A) and 2R1X_ A (length 17, blue, cluster L1-17-A). The structures of longer CDR-L1 loops containing a “protrusion” starting around residue 29. D) CDR-L3 of 4HGW_A, (length 8, white, cluster L3-8-A) and 3RVV_C, (length 9, red, cluster L3-9,10-A). The conformations of CDRs in both clusters appear to be maintained by Gln at position 90 (the side chain of which is shown, along with the contacts to other residues). E) CDR-L3

of 3RVV_C, (length 9, red) and 4HHA_A, (length 10, blue) from cluster L3-9,10-A. CDRs of both length have similar conformations, maintained by the Gln at position 90, with the CDR of length 10 containing an additional Pro at position 95A (The side chains of both Pro are shown in the figure). F) CDR-L3 of 3RVV_C (length 9, red, cluster L3-9,10-A) and 1Y0L_C (length 9, blue, cluster L3-9-A). The alignment shows the difference in conformation between the two largest clusters containing CDRs of length 9. G) CDR-L3 of 3MLX L, (length 10, red) and 4NZT_L, (length 11, blue) from cluster L3-10,11-A. The conformation of both CDRs seems to be stabilized by the interaction between Asn at position 92 and the backbone nitrogen of residue 94 and the presence of large hydrophobic residue at position 91 (the side chains of Asn-92 and Trp-91 are shown in the figure).

Cluster name	Length	Number of structures	Middle structure	Number of unique sequences	Species	Germline
L1-10,11,12-A	10	69	4F33_E	28	Mo	IGKV4
	11	707	3SOB_L	174	Mo, Hu, Ra, Rabbit	IGKV
	12	3	3EOO_A	2	Hu	IGKV3
L1-11-A	11	38	4IMK_C	9	Hu	IGLV3
L1-11-B	11	24	3MLS_M	8	Hu	IGLV3
L1-11-C	11	5	4FQC_L	3	Hu	IGLV3
L1-12-A	12	22	1HQ4_A	12	Hu, Mo	Hu_IGKV, Mo_IGKV4
L1-12-B	12	28	4LLV_L	5	Hu	IGKV3-20*01
L1-12-C	12	13	2OTU_E	3	Mo	IGLV3
L1-12-D	12	7	1HZH_M	3	Hu	IGKV3
L1-13,14-A	13	66	4FQJ_L	23	Hu	IGLV1
	14	51	3U2S_L	14	Hu	IGLV2
L1-13-A	13	23	2WOL_C	6	Hu	IGLV6-57*01
L1-14-A	14	92	1YOL_C	7	Hu, Mo	Mo_IGLV1, Hu_IGLV7
L1-14-B	14	2	4KTD_L	2	Hu	Hu_IGLV5
L1-15-A	15	55	3QRG_L	26	Hu, Mo	IGKV
L1-15-B	15	3	3DGG_C	2	Mo	IGKV3-12*01
L1-16-A	16	273	1KFA_M	65	Hu, Mo	IGKV
L1-17-A	17	113	2R1X_A	31	Hu, Mo	Mo_IGKV8, Hu_IGKV4
L1-17-B	17	3	3LDB_B	2	Ra, Mo	IGKV8
Unclustered	7, 8, 9, 10, 11, 12, 13, 14, 15, 16, 17	104	-	47	-	-

Table S1: Information on canonical forms of CDR-L1. The clusters are ordered first by length, then by number of structures and finally by number of sequences. The germlines are reported in the following way: if over 90% of the structures come from the same allele, that allele is shown in the Table. Otherwise, we report the lowest step in the classification hierarchy that can explain 90% of the data. If two different loci are present in a cluster we report both of them.

Cluster name	Length	Number of structures	Middle structure	Number of unique sequences	Species	Germline
L2-7-A	7	1708	2G5B_A	291	Hu, Mo, Ra,	IGKV, IGLV
L2-7-B	7	21	3I9G_L	6	Hu, Mo	IGKV, IGLV
L2-7-C	7	6	2ABI_A	3	Mo	IGKV10-96*01
L2-11-A	11	13	2GSG_A	2	Mo	IGLV3*01
L2-11-B	11	5	2H3N_C	3	Rhesus monkey, Hu	IGLV, Hu_VPREB1*02
Unclustered	7	9	-	6	-	-

Table S2: Information on canonical forms of CDR-L2. See description below Table S1

Cluster name	Length	Number of Structures	Middle structure	Number of unique structures	Species	Framework germline	Joining germline
L3-5-A	5	17	4JPI_B	6	Hu	IGKV3, IGLV2	IGLJ, IGKJ
L3-7-A	7	2	1DFB_L	2	Hu	IGKV01-5*03	IGKJ3
L3-8-A	8	106	4HGW_A	29	Hu, Mo, Ra	IGKV	IGKJ
L3-8-B	8	9	3VW3_L	4	Mo	IGKV1-117*01	IGKJ
L3-8-C	8	6	2FD6_L	2	Mo	IGKV	IGKJ
L3-8-D	8	3	1TZH_A	2	Mo	IGKV12-44*01	IGKJ
L3-9,10-A	9	1123	3RVV_C	331	Hu, Mo, Ra	IGKV	IGKJ
	10	10	4HHA_A	4	Hu, Mo	Mo_IGKV1-110*01, Hu_IGKV3-11*01	IGKJ5*01
L3-9-A	9	107	1Y0L_C	22	Hu, Mo	IGLV, IGKV4-86*01	IGLJ, IGKL5*01
L3-9-B	9	5	2VXS_O	2	Hu	IGLV6-57*01	IGLJ
L3-9-C	9	2	2HWZ_L	2	Mo	IGKV1-117*01	IGKJ4
L3-10,11-A	10	4	3MLX_L	1	Hu	IGLV1-51*02	IGLJ
	11	49	4NZT_L	22	Hu	IGLV1, IGLV3-21	IGLJ
L3-10-A	10	31	3U2S_L	5	Hu	IGLV2-14*01	IGLJ
L3-10-B	10	9	3U79_B	2	Hu	IGKV1-33*01	IGKJ
L3-10-C	10	4	2DD8_L	3	Hu	IGLV3	IGLJ
L3-10-D	10	3	4JAM_L	2	Hu	IGLV3	IGLJ6
L3-11-A	11	3	3MA9_L	3	Hu	IGLV3, IGKJ1	IGLJ, IGKJ1
L3-12-A	12	4	3QHZ_L	2	Hu	IGLV1-44*01	IGLJ
L3-12-B	12	2	4JY5_L	2	Hu	IGLV3-21	IGLJ
L3-13-A	13	5	2BOS_L	2	Hu	IGLV1-47*01	IGLJ
Unclustered	6, 8, 9, 10, 11, 12 , 13, 19	248	-	80	-	-	-

Table S3: Information on canonical forms of CDR-L3. See description below Table S1

Cluster name	Length	Number of structures	Middle structure	Number of unique sequences	Species	Germline
H1-4-A	4	8	1KXQ_H	3	Camel	IGHV1S45*01
H1-6-A	6	7	2QQQ_B	2	Channel catfish	NITR11
H1-7-A	7	1267	1PLG_H	257	Hu, Mo, Ra, Camel, Llama, Rabbit, Rhesus Monkey, Sheep, Hamster	IGHV
H1-7-B	7	18	4FQQ_F	6	Hu, Mo	Hu_IGHV, Mo_IGHV5S21*01
H1-7-C	7	10	4KPH_H	2	Mo	IGHV3-8*02
H1-7-D	7	8	1BZQ_K	3	Camel, Llama	IGHV1S45
H1-7-E	7	7	4DKA_A	2	Llama	IGV1S3*01
H1-7-F	7	6	4NBZ_D	3	Llama, Mo	Mo_IGHV9-1*01, Llama_IGHV1S3*01
H1-7-G	7	6	3EZJ_B	3	Camel, Llama	IGHV
H1-8-A	8	37	3RVW_D	8	Hu, Mo	Hu_IGHV4-38-2*01, Mo_IGHV3
H1-8-B	8	14	1RVK_H	3	Mo	Mo_IGHV3-2*02
H1-8-C	8	7	1F58_H	2	Hu, Mo	Hu_IGHV4-38-2*01, Mo_IGHV3-1*02
H1-9-A	9	86	3IDN_B	9	Hu, Mo	Mo_IGHV8, Hu_IGHV2
H1-9-B	9	5	3BKJ_H	2	Mo	IGHV8-12*01
Unclustered	3,6,7, 8,9,10, 12,14	248	-	102	-	-

Table S4: Information on canonical forms of CDR-H1. See description below Table S1

Cluster name	Length	Number of structures	Middle structure	Number of unique sequences	Species	Germline
H2-7-A	7	387	3ZKM_H	91	Hu, Mo, Rat, Sheep, Camel, Llama, Rabbit	IGHV
H2-7-B	7	4	4FQC_H	3	Hu	IGHV4
H2-8-A	8	650	1I8M_B	197	Hu, Mo, Rat, Camel, Rhesus Monkey, Llama, Rabbit	IGHV
H2-8-B	8	305	2VXS_K	93	Hu, Mo, Camel, Rat, Llama	IGHV
H2-8-C	8	23	1ZLV_H	2	Hu	IGHV3
H2-8-D	8	19	1YQV_H	9	Hu, Mo	Hu_IGHV1, Mo_IGHV1
H2-8-E	8	8	3OGO_E	3	Hu, Rat, Camel	IGHV
H2-8-F	8	7	2XVM_B	2	Hu, Llama	Hu_IGHV1, Llama_IGHV1S4
H2-8-G	8	6	1ZA6_B	3	Mo	IGHV1
H2-8-H	8	4	3EYV_H	2	Mo, Hu	IGHV
H2-8-I	8	3	3QOS_B	3	Hu, Mo	Mo_IGHV5, Hu_IGHV3
H2-8-J	8	3	1F4X_H	2	Mo	IGHV5
H2-10-A	10	147	3HZV_B	25	Mo, Hu, Ra, Hamster	IGHV
Unclustered	8,9,10, 11,12	213	-	103	-	-

Table S5: Information on canonical forms of CDR-H2. See description below Table S1

This file shows the detailed comparison between our length-independent clustering and the recent clustering of CDR structures by North & Dunbrack *et al.*

Our cluster	North & Dunbrack *et al.*	Fraction of CDRs in North & Dunbrack *et al.* cluster found in our cluster
**L1-10,11,12-A**	**L1-10-1**	**95%**
	**L1-11-1**	**100%**
	**L1-11-1**	**100%**
**L1-11-A**	**L1-11-3**	**40%**
**L1-11-B**	**L1-11-3**	**20%**
L1-11-C	-	-
**L1-12-A**	**L1-12-1**	**100%**
L1-12-B	L1-12-2	80%
L1-12-C	L1-12-3	100%
L1-12-D	-	-
**L1-13,14-A**	**L1-13-1**	**83%**
	**L1-14-2**	**75%**
**L1-13-A**	**L1-13-2**	**100%**
**L1-14-A**	**L1-14-1**	**100%**
L1-14-B	-	-
**L1-15-A**	**L1-15-1**	**100%**
L1-15-B	L1-15-2	100%
**L1-16-A**	**L1-16-1**	**90%**
**L1-17-A**	**L1-17-1**	**94%**
L1-17-B	-	-

Table S6: The comparison between North & Dunbrack *et al.* work and our clustering for CDR-L1. The first column shows the cluster label in our set while the second contains the labels of corresponding North & Dunbrack *et al.* clusters. The third column shows the fraction of CDRs in North & Dunbrack *et al.* cluster that is contained within our cluster. Two clusters were considered equivalent if our cluster contained at least 50% of CDRs present in the corresponding North & Dunbrack *et al.* cluster. North & Dunbrack *et al.* cluster was considered to be split between two of our clusters if the two clusters together contained at least 50% of CDRs from North & Dunbrack *et al.* cluster (for example the CDRs from North & Dunbrack *et al.* cluster L1-11-3 are split between our clusters L1-11-A and L1-11-B). The clusters in our work which contain at least six unique sequences are shown in bold

Our cluster	North & Dunbrack *et al.*	Fraction of CDRs in North & Dunbrack *et al.* cluster found in our cluster
**L2-7-A**	**L2-8-1**	**100%**
	**L2-8-2**	**100%**
	**L2-8-4**	**100%**
	**L2-8-5**	**100%**
L2-7-B	**L2-8-3**	**100%**
L2-7-C	-	-
L2-11-A	L2-12-2	100%
L2-11-B	L2-12-1	100%

Table S7: The comparison between North & Dunbrack *et al.* work and our clustering for CDR-L2. See description under Table S6

Our cluster	North & Dunbrack *et al.*	Fraction of CDRs in North & Dunbrack *et al.* cluster found in our cluster
**L3-5-A**	**-**	**-**
L3-7-A	L3-7-1	100%
**L3-8-A**	**L3-8-1**	**93%**
L3-8-B	L3-8-2	50%
L3-8-C	L3-8-cis6-1	66%
L3-8-D	L3-8-cis6-1	33%
**L3-9,10-A**	**L3-9-2**	**100%**
	**L3-9-cis7-1**	**98%**
	**L3-9-cis7-2**	**88%**
	**L3-9-cis7-3**	**100%**
	**L3-10-cis7and8-1**	**100%**
**L3-9-A**	**L3-9-1**	**68%**
	**L3-9-cis6-1**	**100%**
L3-9-B	-	-
L3-9-C	-	-
**L3-10,11-A**	**L3-11-1**	**63%**
L3-10-A	-	-
L3-10-B	-	-
L3-10-C	-	-
L3-10-D	-	-
L3-11-A	-	-
L3-12-A	-	-
L3-12-B	-	-
L3-13-A	-	-

Table S8: The comparison between North & Dunbrack *et al.* work and our clustering for CDR-L3. See description under Table S6

Our cluster	North & Dunbrack *et al.*	Fraction of CDRs in North & Dunbrack *et al.* cluster found in our cluster
H1-4-A	H1-10-1	100%
H1-6-A	-	-
**H1-7-A**	**H1-13-1**	**98%**
	**H1-13-2**	**57%**
**H1-7-B**	**-**	**-**
H1-7-C	H1-13-7	67%
H1-7-D	-	-
H1-7-E	H1-13-5	33%
H1-7-F	-	-
H1-7-G	H1-13-5	33%
**H1-8-A**	**H1-14-1**	**91%**
H1-8-B	-	-
H1-8-C	-	-
**H1-9-A**	**H1-15-1**	**78%**
H1-9-B	-	-

Table S9: The comparison between North & Dunbrack *et al.* work and our clustering for CDR-H1. See description under Table S6

Our cluster	North & Dunbrack *et al.*	Fraction of CDRs in North & Dunbrack *et al.* cluster found in our cluster
**H2-7-A**	**H2-9-1**	**96%**
H2-7-B	-	-
**H2-8-A**	**H2-10-1**	**95%**
**H2-8-B**	**H2-10-2**	**90%**
H2-8-C	-	-
**H2-8-D**	**H2-10-3**	**66%**
H2-8-E	-	-
H2-8-F	-	-
H2-8-G	H2-10-3	22%
H2-8-H	H2-10-6	50%
H2-8-I	-	-
H2-8-J	-	-
**H2-10-A**	**H2-12-1**	**100%**

Table S10: The comparison between North & Dunbrack *et al.* work and our clustering for CDR-H2. See description under Table S6

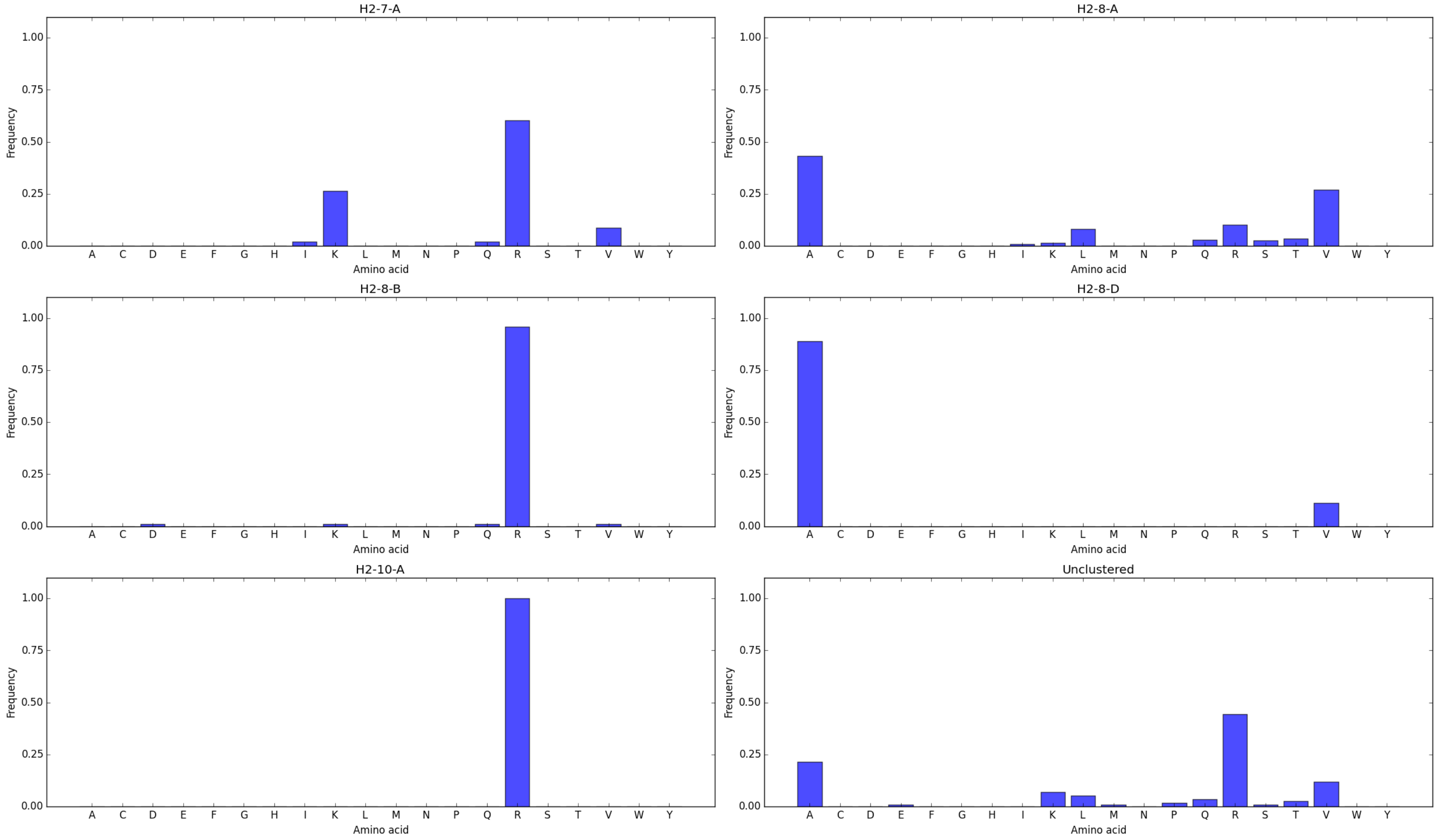

Figure S2: The amino acid distributions of the framework residue at Chothia position 71, plotted for each CDR-H2 cluster that contains at least six unique sequences. The y-axis in each panel shows the frequency of each amino acid type and the x-axis shows all standard amino acids. The "Unclustered" panel shows the distribution for all structures that are not contained within the large clusters.

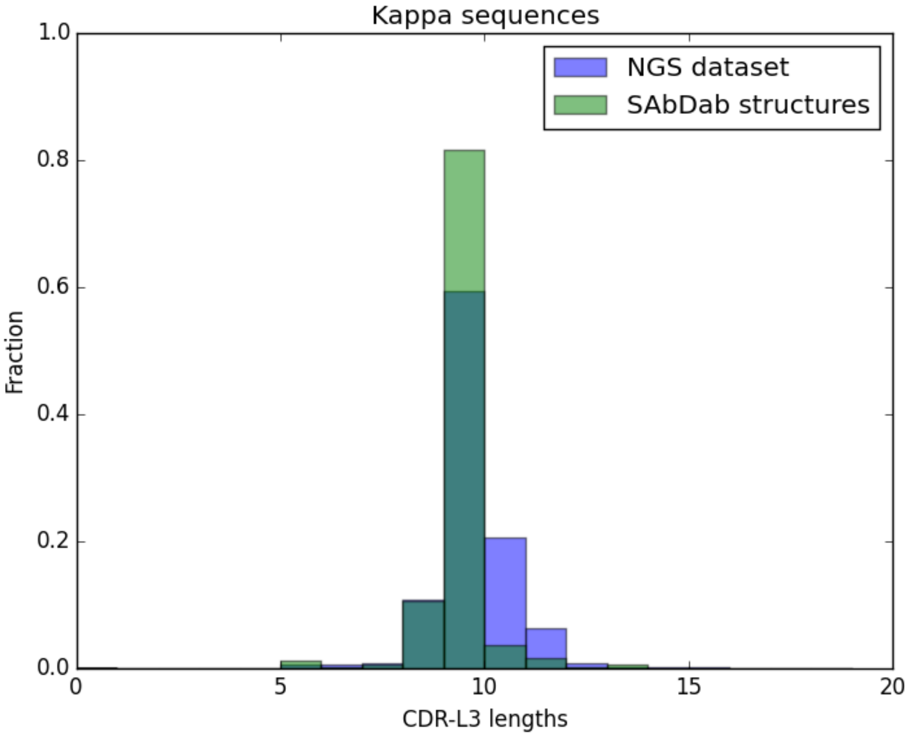

Figure S3: Comparison between the distribution of lengths of unique kappa CDR-L3 sequences in the UCB NGS dataset and in our structural data (Main text Section 2.2). The NGS distribution is shown in blue and the structural distribution is shown in green. The histograms have been normalized and the height of the bars shows the fraction of sequences that have a particular length. The graph shows that sequences of length nine are overrepresented and the sequences of length 10 are underrepresented in the structural dataset.

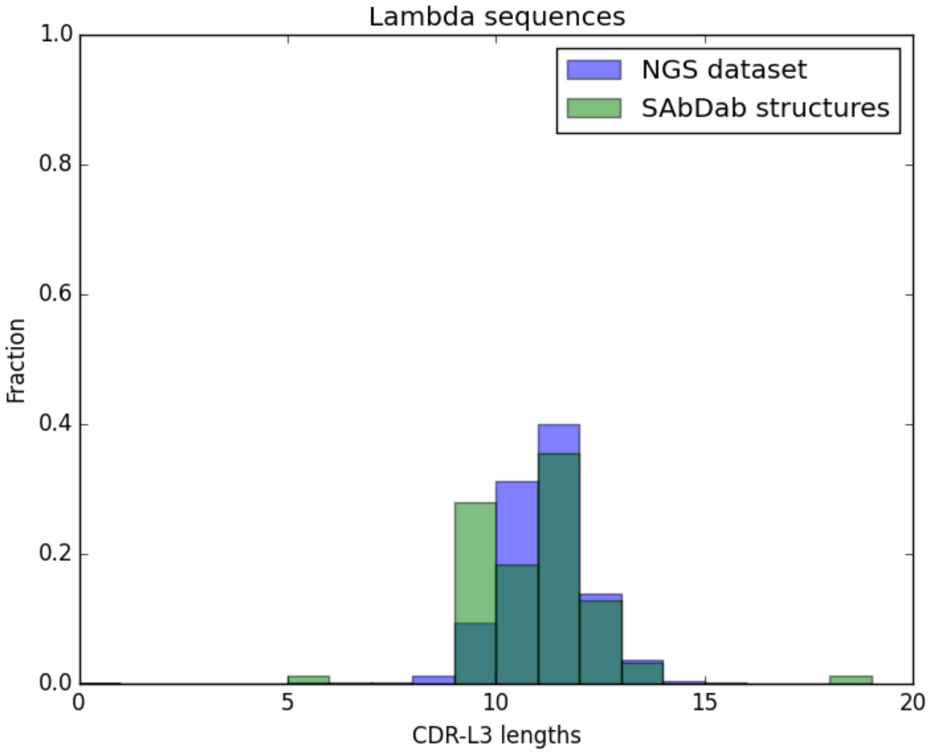

Figure S4: Comparison between the distribution of lengths of unique lambda CDR-L3 sequences in the UCB NGS dataset and the distribution in our structure data (Main text Section 2.2). The NGS distribution is shown in blue and the structural distribution is shown in green. The histograms have been normalized and the height of the bars shows the fraction of sequences that have a particular length. The graph shows that sequences of length nine are overrepresented and the sequences of length 10 are underrepresented in the structural dataset.

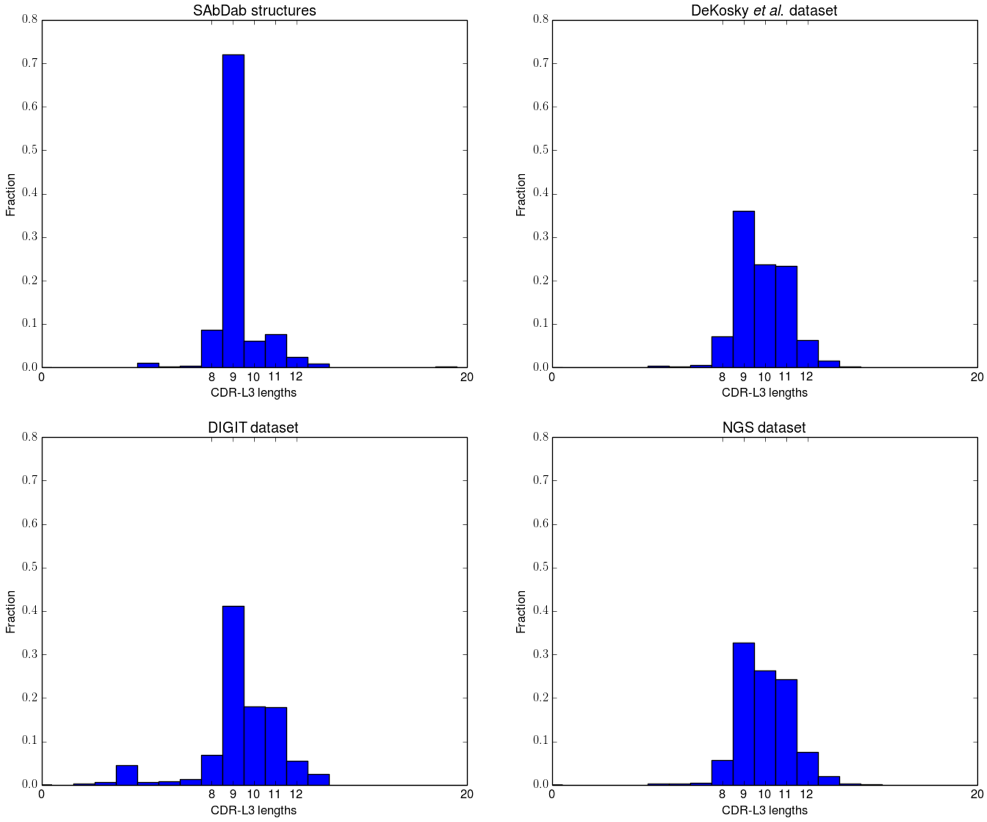

Figure S5: Comparison between the length distributions of the unique CDR-L3 sequences in the three NGS datasets and the distribution in our structure data (Main text Section 2.2). The top left panel shows the distribution in our structural dataset, the top right panel shows the distribution in the DeKosky *et al.* (DeKosky et al., 2014) dataset, the bottom left panel shows the distribution for the DIGIT (Chailyan, Tramontano, & Marcatili, 2012) dataset and the bottom right panel shows the distribution in the UCB NGS dataset. The histograms have been normalized and the height of the bars shows the fraction of sequences with a particular length. The distributions of lengths in the UCB NGS and the DeKosky *et al.* datasets are similar, which is expected as they both contain only human sequences. The structural dataset contains mostly sequences of length nine, other lengths are significantly underrepresented.

ROC curves

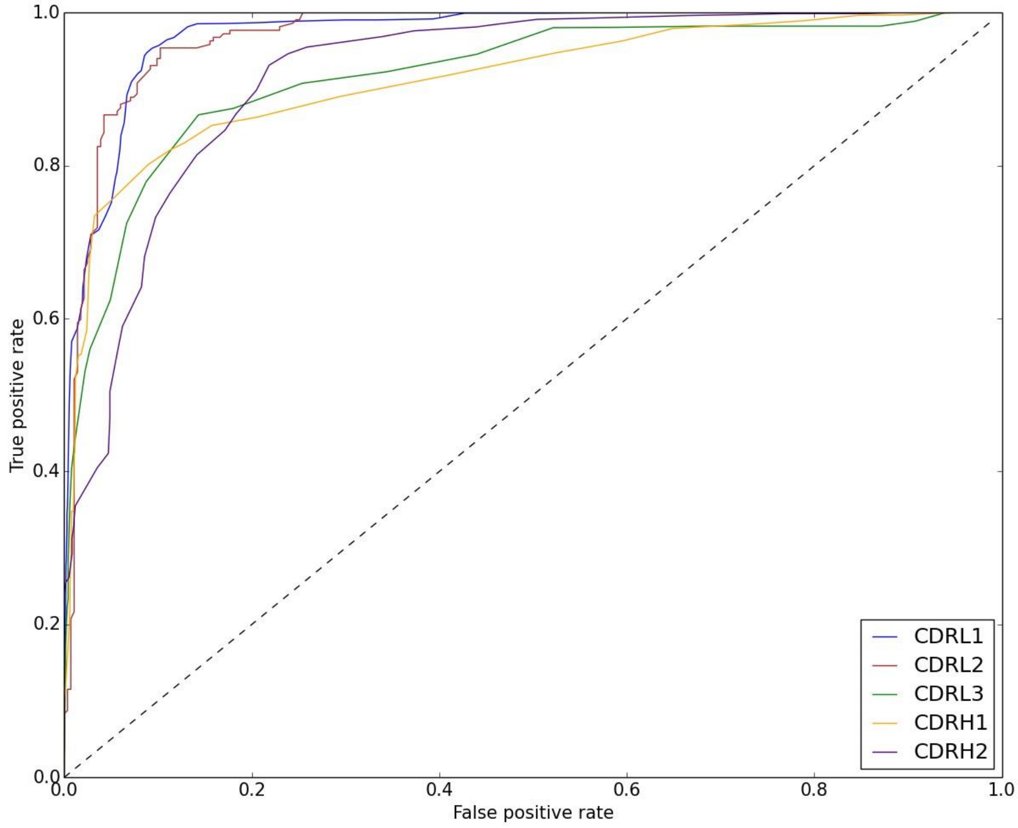

Figure S6: Receiver Operating Characteristic (ROC) curves for length-independent clustering for all CDR types, obtained by macro-averaging the results for each constituent cluster for each CDR type. The ROC curve for CDRL1 is shown in blue, for CDRL2 in brown, for CDRL3 in green, for CDRH1 in yellow and for CDRH2 in violet. The Area Under the Curve (AUC) for CDRL1 is 0.97, for CDRL2 is 0.97, for CDRL3 is 0.92, for CDRH1 is 0.91 and for CDRH2 is 0.92. A perfect model would get an AUC score of 1.0 while a random predictor would receive a score of 0.5.

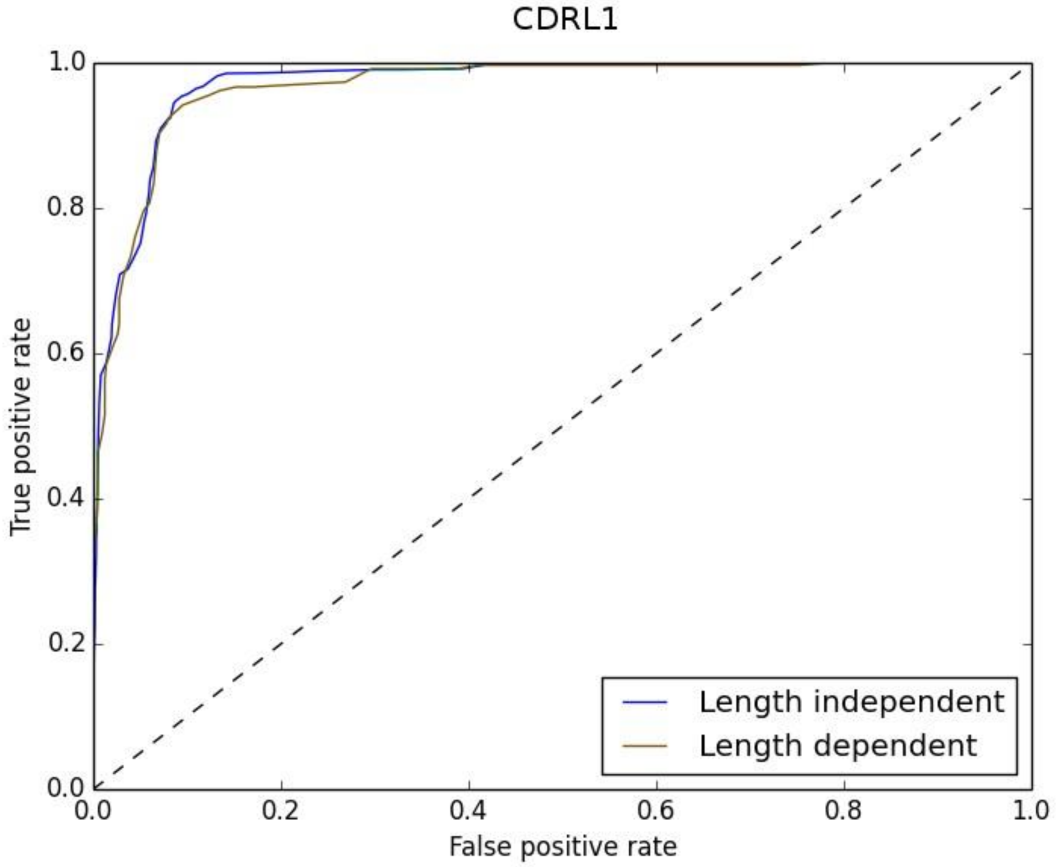

Figure S7: Comparison between ROCs plotted for length-independent and length-dependent clustering of CDR-L1 loops, obtained by macro-averaging the results for each constituent cluster. The length independent curve is shown in blue and the length dependent one in brown. The AUC for both curves is 0.97. The difference between the curves is not statistically significant with a p-value of 0.48 (see Section 4.4).

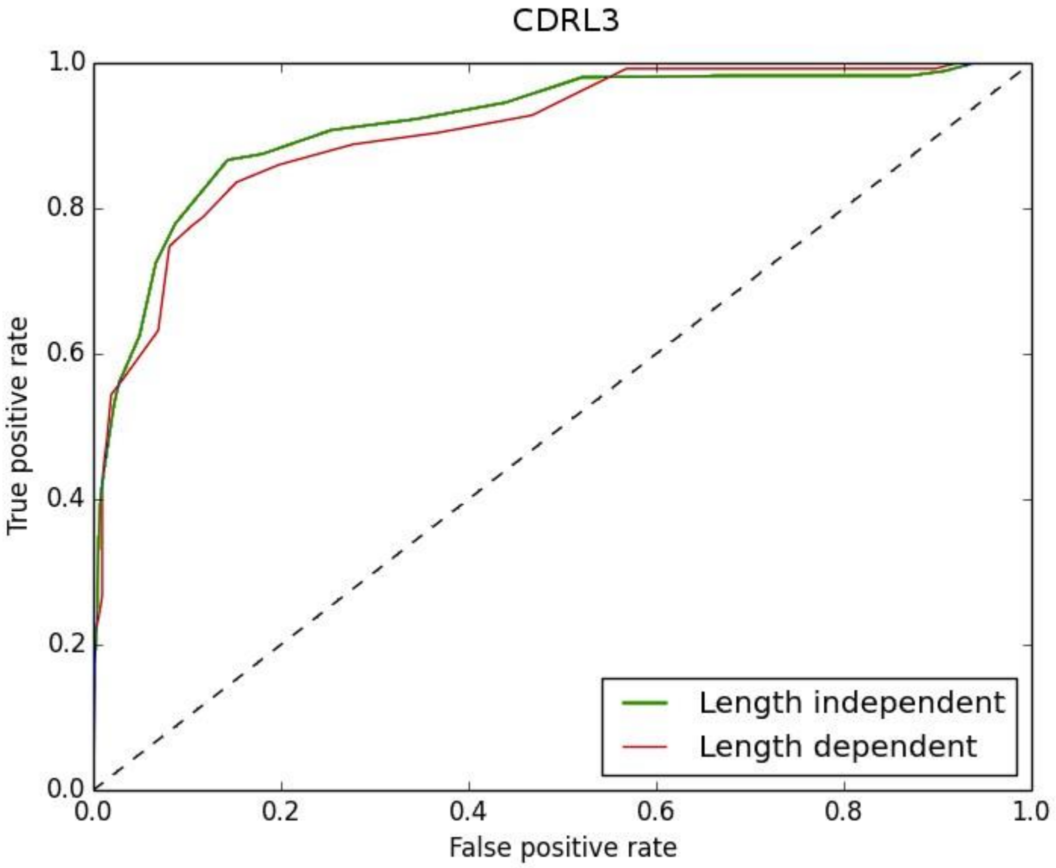

Figure S8: Comparison between ROCs plotted for length-independent and length-dependent clustering of CDR-L3 loops, obtained by macro-averaging the results for each constituent cluster. The length independent curve is shown in green and the length dependent one in red. The AUC for the length-independent curve is 0.92 while the AUC for length-dependent curve is 0.91. The difference between the curves is not statistically significant with a p-value of 0.07 (see Section 4.4).

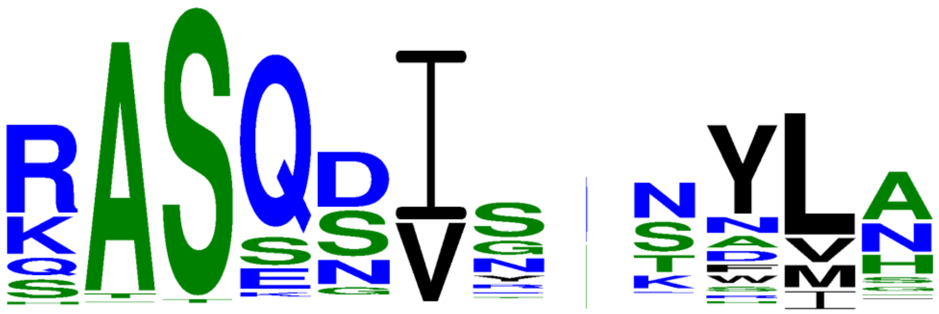

L1-10,11,12-A

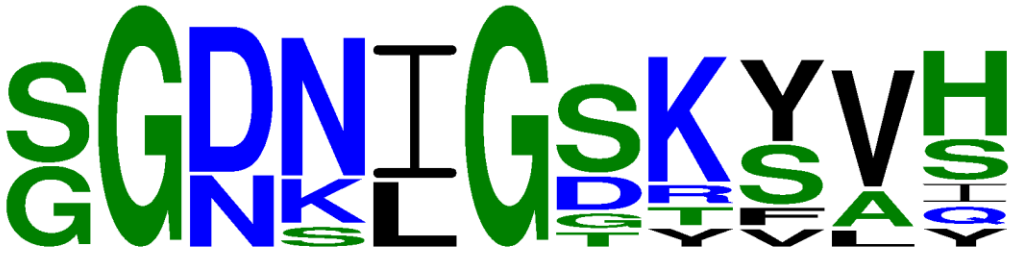

L1-11-A

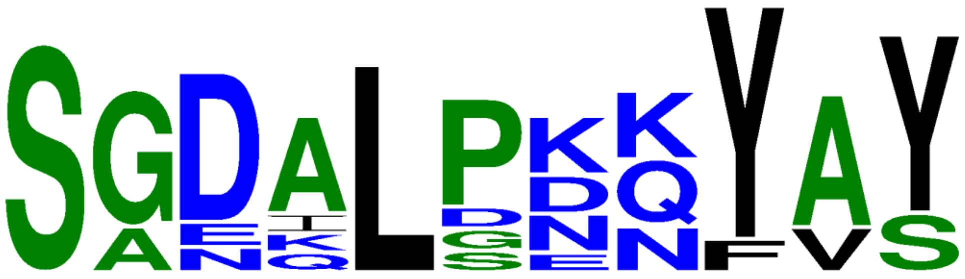

L1-11-B

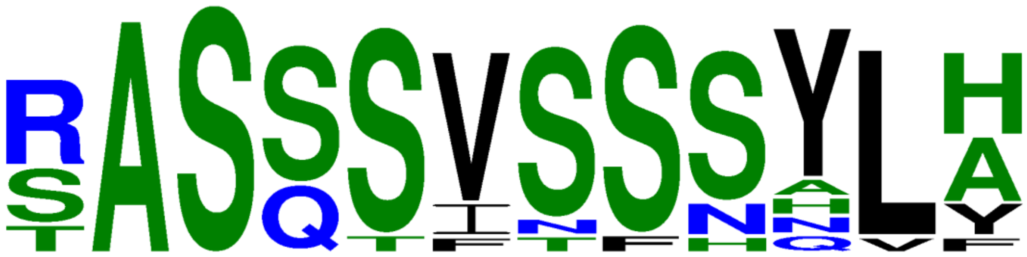

L1-12-A

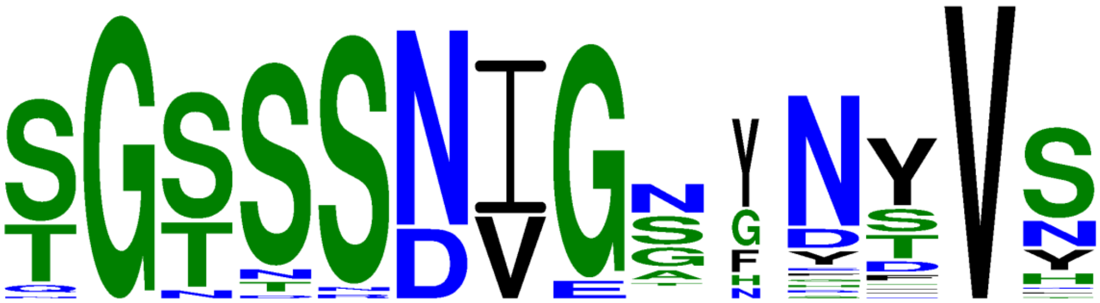

L1-13,14-A

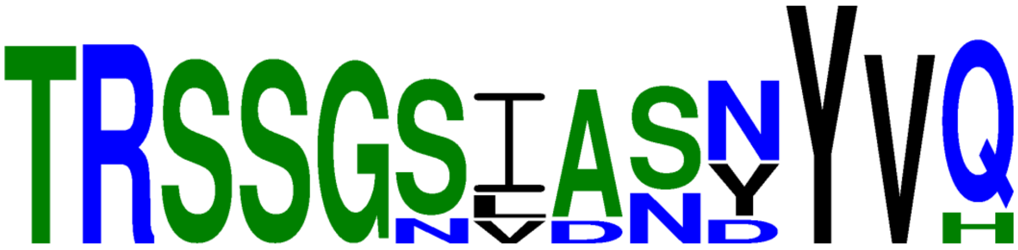

L1-13-A

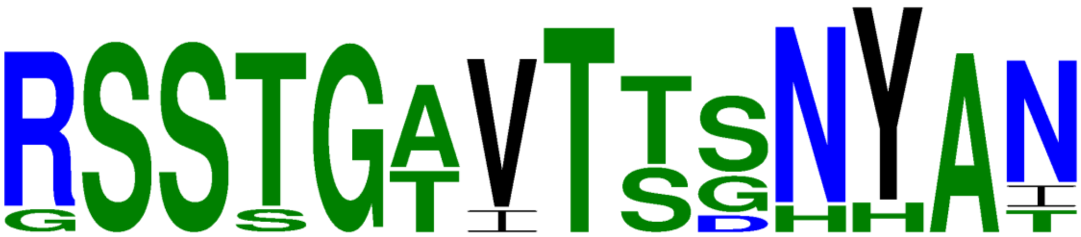

L1-14-A

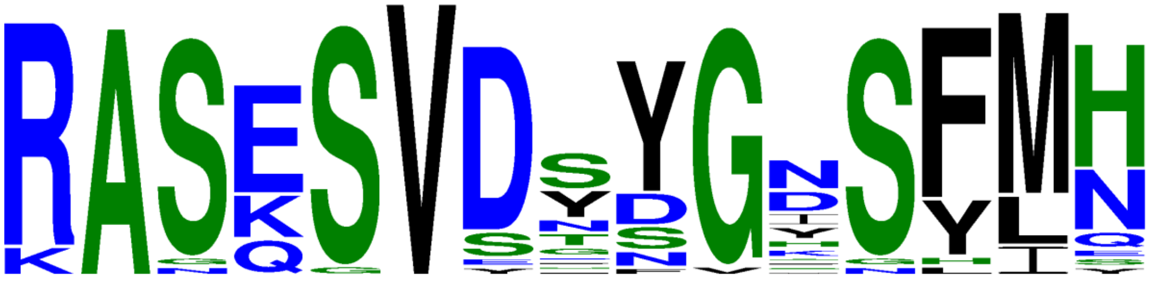

L1-15-A

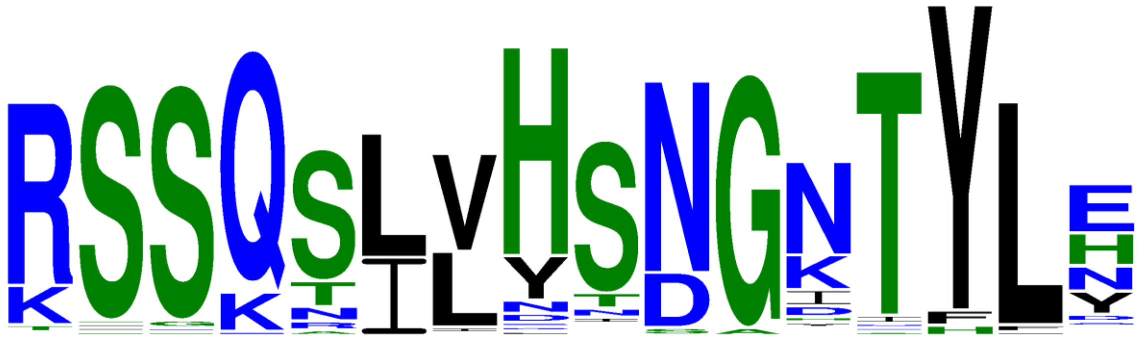

L1-16-A

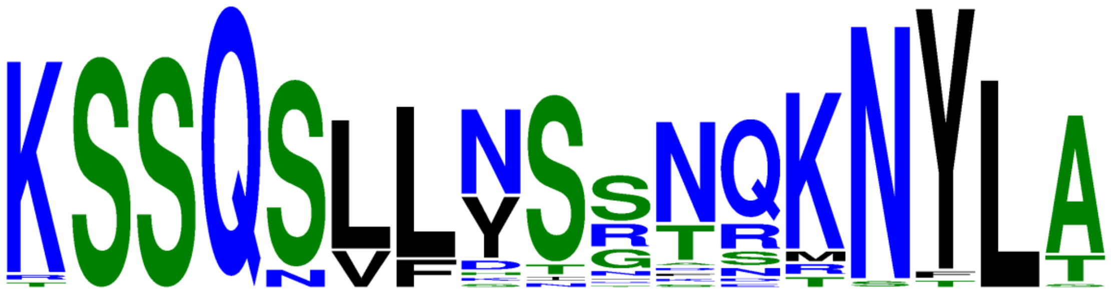

L1-17-A

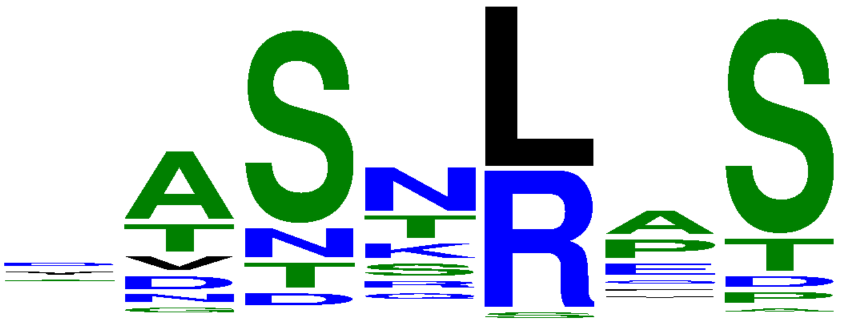

L2-7-A

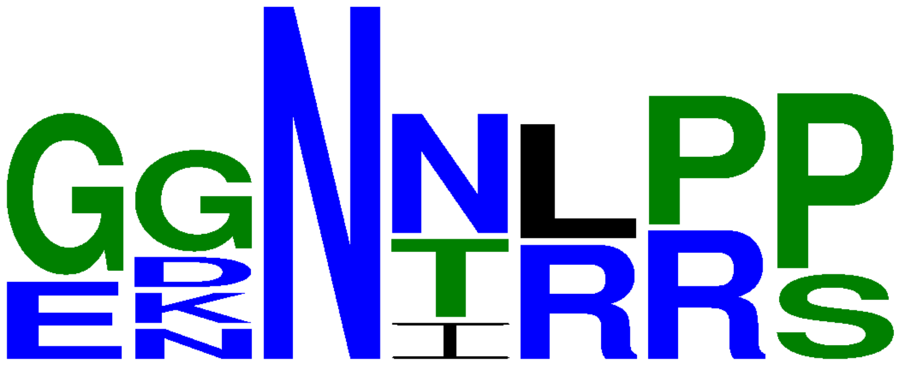

L2-7-B

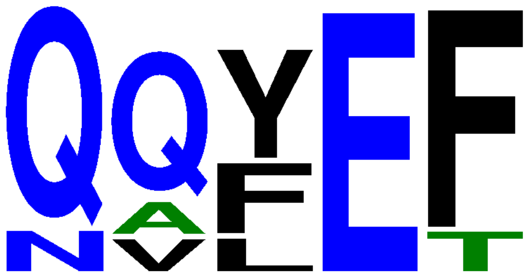

L3-5-A

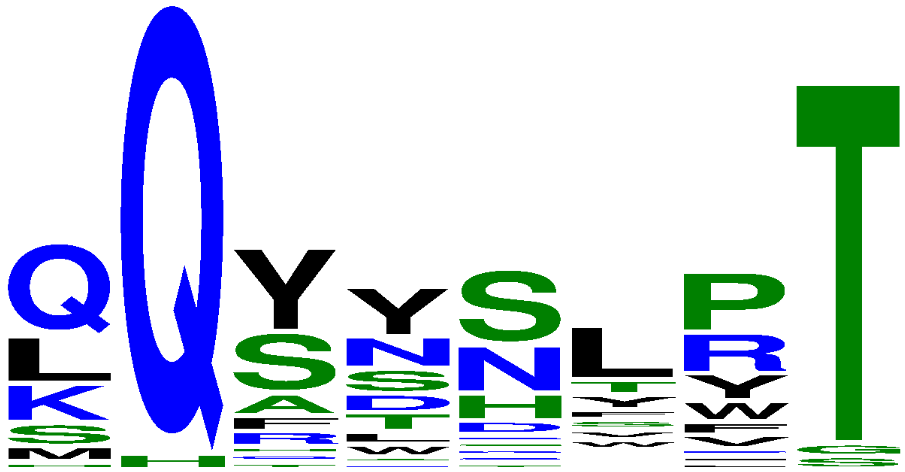

L3-8-A

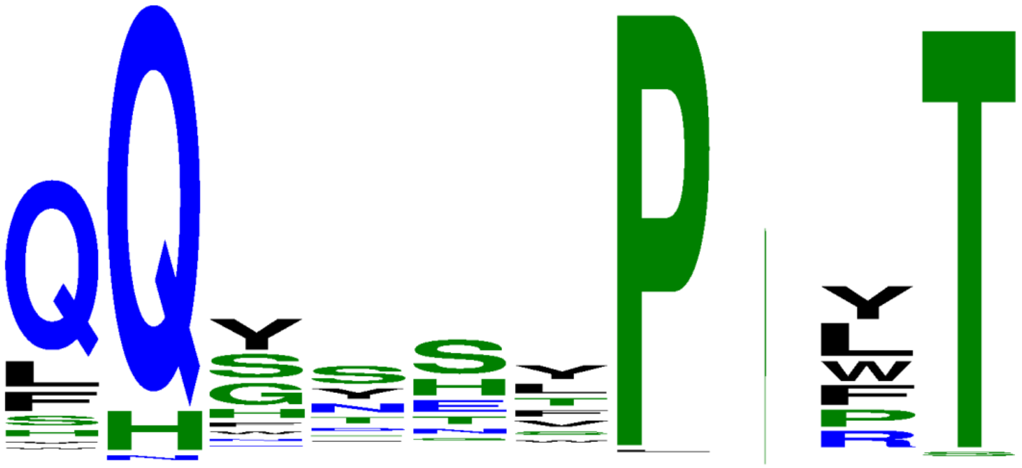

L3-9,10-A

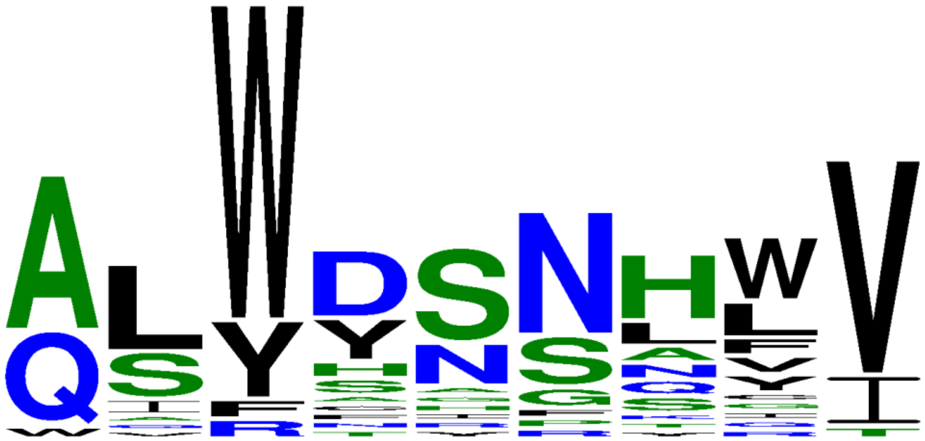

L3-9-A

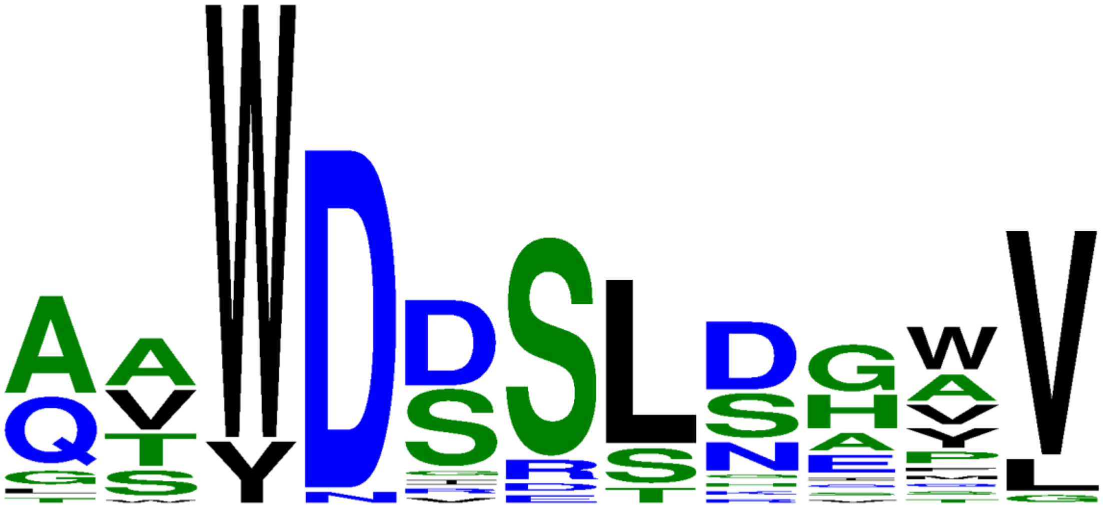

L3-10,11-A

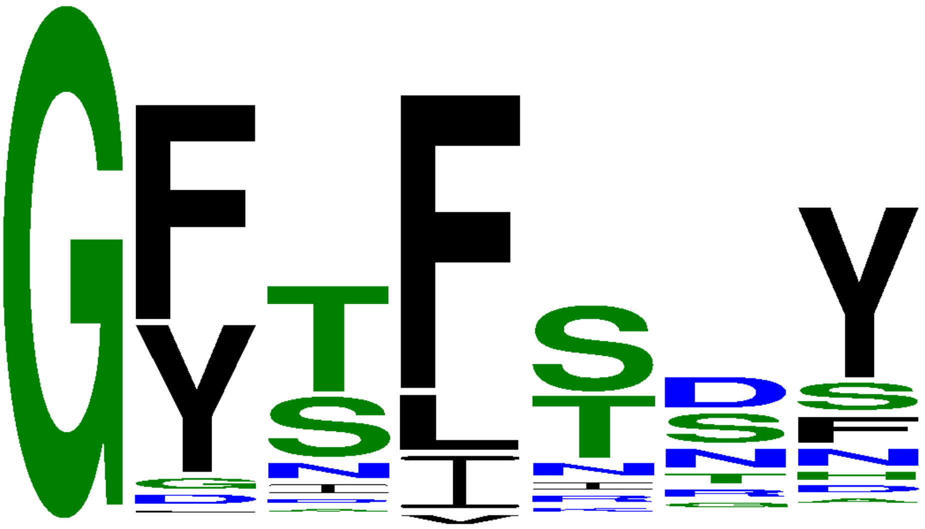

H1-7-A

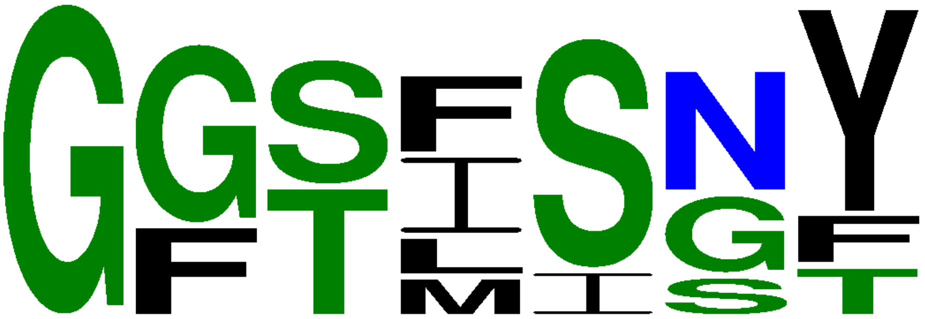

H1-7-B

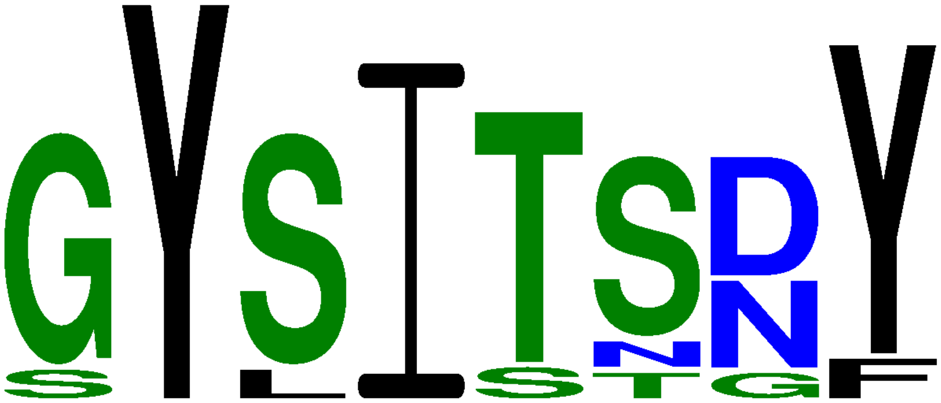

H1-8-A

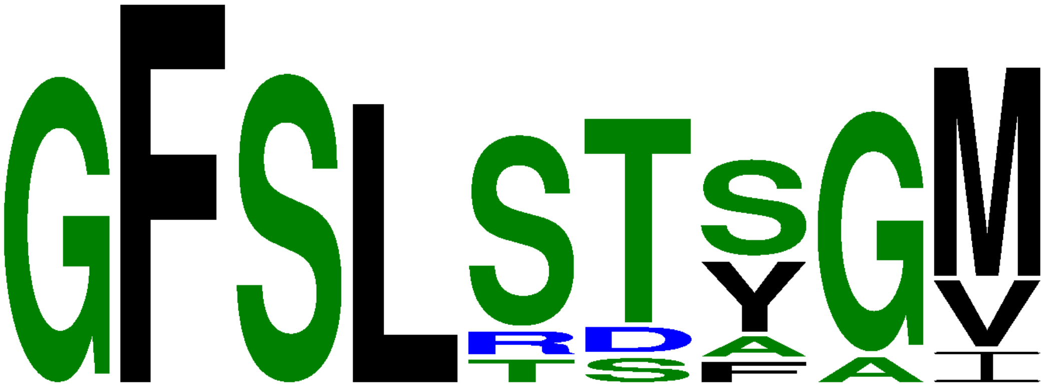

H1-9-A

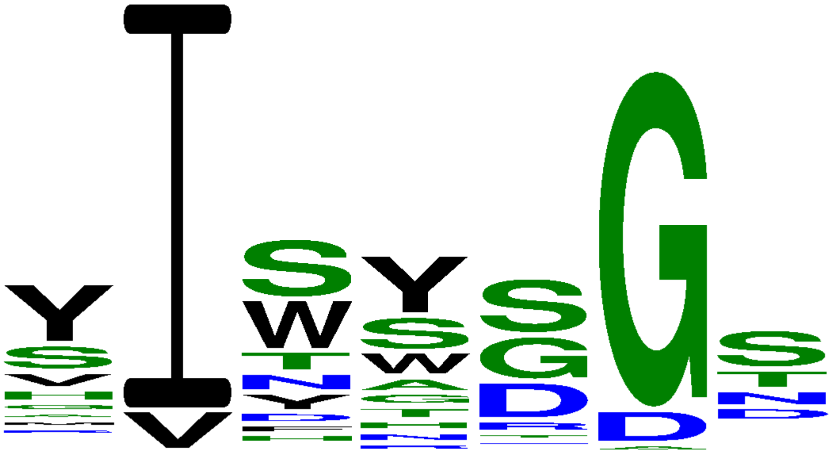

H2-7-A

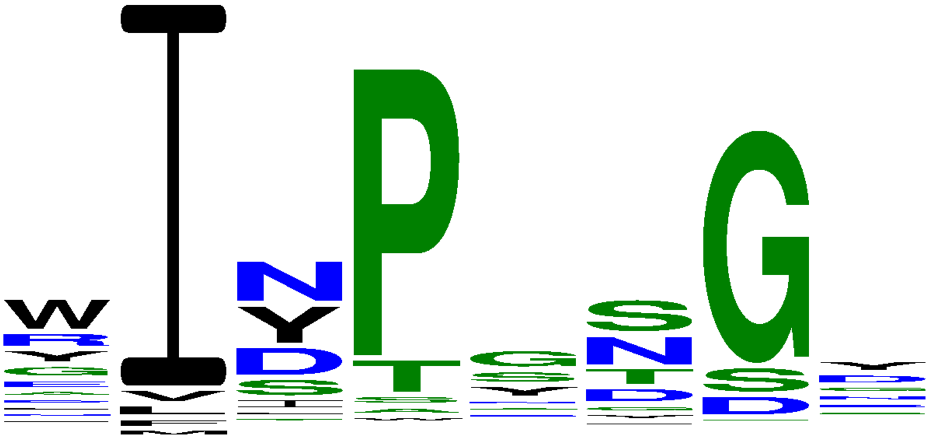

H2-8-A

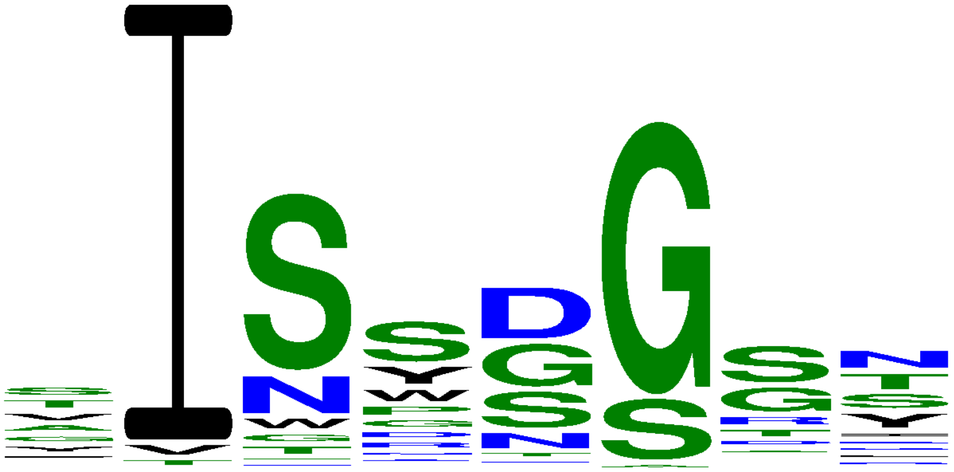

H2-8-B

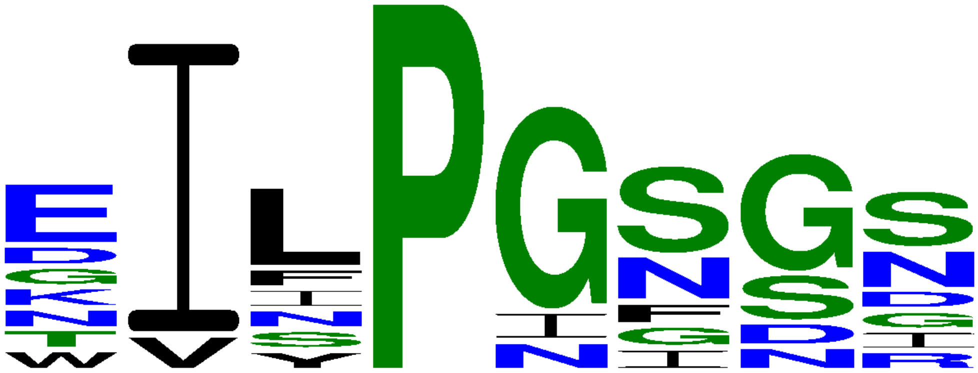

H2-8-D

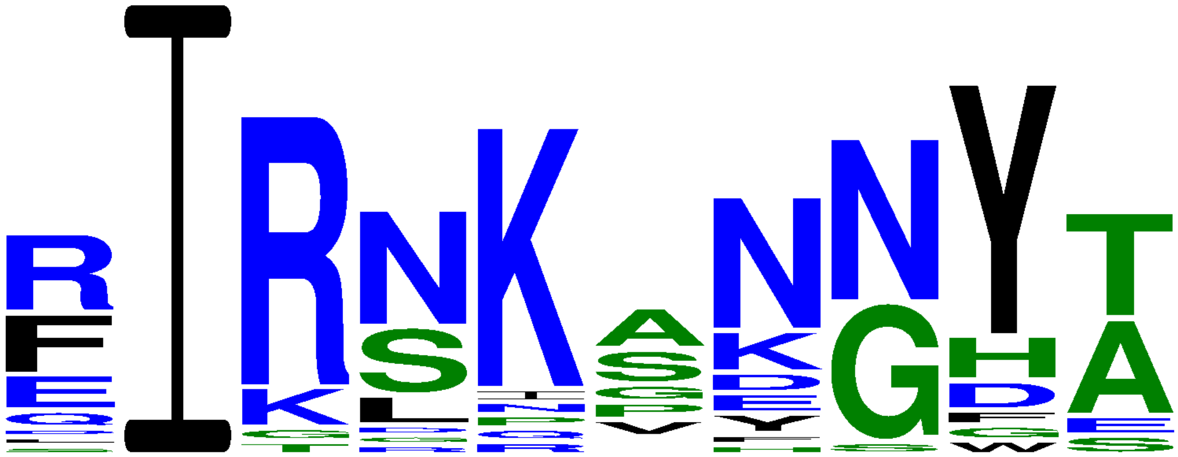

H2-10-A

ClusterID	CDR_type	PDBcode	Chain	Sequence	Species	Germline
L1-10,11,12-A	CDRL1	3EO9	L	RASKTISKYLA	human	IGKV1-16
L1-10,11,12-A	CDRL1	1FN4	A	KGSQNINNYLA	Norway rat	IGKV22S2
L1-10,11,12-A	CDRL1	3D0L	A	RASQGVTSALA	human	IGKV1D-13
L1-10,11,12-A	CDRL1	3A67	L	RASQSIGDNLH	house mouse	IGKV5-43
L1-10,11,12-A	CDRL1	1IC4	L	RASQSIGNNLH	house mouse	IGKV5-43
L1-10,11,12-A	CDRL1	2AJ3	A	RASQDIQKFLA	human	IGKV1-9
L1-10,11,12-A	CDRL1	1MQK	L	RASENIYSYLA	house mouse	IGKV12-44
L1-10,11,12-A	CDRL1	1B0W	A	QASQDISDYLI	human	IGKV1-33
L1-10,11,12-A	CDRL1	2BX5	G	RASQSISSYLN	human	IGKV1-39
L1-10,11,12-A	CDRL1	1UYW	L	KASENVVTYVS	house mouse	IGKV6-20
L1-10,11,12-A	CDRL1	2ADF	L	KASQDINKYIA	house mouse	IGKV19-93
L1-10,11,12-A	CDRL1	4HJJ	L	RASESISSNLA	human	IGKV4-1
L1-10,11,12-A	CDRL1	2B2X	L	SASSQVNHMF	house mouse	IGKV4-68
L1-10,11,12-A	CDRL1	1AR2	A	QASQDIIKHLN	human	IGKV1-33
L1-10,11,12-A	CDRL1	4LLW	D	KASQDVSIGVA	human	IGKV1-16
L1-10,11,12-A	CDRL1	3U0W	L	SASQGISNYLN	house mouse	IGKV10-94
L1-10,11,12-A	CDRL1	1UWG	L	KASQNVGTHVA	house mouse	IGKV6-15
L1-10,11,12-A	CDRL1	1AD0	C	RASSSVTYIH	human	IGKV1-16
L1-10,11,12-A	CDRL1	3IDI	A	RASQGVTSALA	human	IGKV1-13
L1-10,11,12-A	CDRL1	1I8M	A	RASENIYSYLA	house mouse	IGKV12-44
L1-10,11,12-A	CDRL1	4F33	C	SASSSVSYMH	house mouse	IGKV4-59
L1-10,11,12-A	CDRL1	1BVK	D	RASGNIHNYLA	human	IGKV1-27
L1-10,11,12-A	CDRL1	3DIF	C	KASQDVSTAVA	house mouse	IGKV6-17
L1-10,11,12-A	CDRL1	2RCS	L	RASQEISGYLS	house mouse	IGKV9-124
L1-10,11,12-A	CDRL1	3QPQ	E	RASGNIHNYLA	house mouse	IGKV12-41
L1-10,11,12-A	CDRL1	1NMC	C	RASQDISNYLN	house mouse	IGKV10-96
L1-10,11,12-A	CDRL1	1J1X	L	RASQSIGNNLH	house mouse	IGKV5-43
L1-10,11,12-A	CDRL1	1FE8	M	RASQDIGNYLN	house mouse	IGKV10-96
L1-10,11,12-A	CDRL1	1C5B	L	RTSQEISGYLS	house mouse	IGKV9-124
L1-10,11,12-A	CDRL1	1D5B	A	KASQDINSYLN	house mouse	IGKV14-111
L1-10,11,12-A	CDRL1	4N9G	B	RASQGISNYLA	human	IGKV1-16
L1-10,11,12-A	CDRL1	2YBR	B	RASQSVRSYLA	human	IGKV3D-11
L1-10,11,12-A	CDRL1	2FJF	E	RASQDVSTAVA	human	IGKV1-39
L1-10,11,12-A	CDRL1	4AEH	L	RASENIYRNLA	house mouse	IGKV12-46
L1-10,11,12-A	CDRL1	1REI	A	QASQDIIKYLN	human	IGKV1-33
L1-10,11,12-A	CDRL1	2BMK	L	SATSGVNYMH	house mouse	IGKV4-57
L1-10,11,12-A	CDRL1	1BRE	B	QASQDISDYLI	human	IGKV1-33
L1-10,11,12-A	CDRL1	4NRZ	L	RASQHIKKYLN	human	IGKV1-39
L1-10,11,12-A	CDRL1	2PW2	A	RASQGVTSALA	human	IGKV1D-13
L1-10,11,12-A	CDRL1	1UWE	U	AASQNVGTNVA	house mouse	IGKV6-15
L1-10,11,12-A	CDRL1	1UWG	X	KASQNVGTHVA	house mouse	IGKV6-15
L1-10,11,12-A	CDRL1	4LF3	A	RASQGIRNDLG	house mouse	IGKV11-125
L1-10,11,12-A	CDRL1	2YSS	A	RASQSIGNNLH	human	IGKV3-15
L1-10,11,12-A	CDRL1	2OSL	B	RASSSVSYIH	house mouse	IGKV4-72
L1-10,11,12-A	CDRL1	1BFO	A	KASQNIDKYLN	Norway rat	IGKV22S7
L1-10,11,12-A	CDRL1	4HFW	L	RASQSISNYLN	human	IGKV1-6
L1-10,11,12-A	CDRL1	1C5D	A	QASQDINKYIA	Norway rat	IGKV19S2
L1-10,11,12-A	CDRL1	3SKJ	L	RASQSISTWLA	human	IGKV1-5
L1-10,11,12-A	CDRL1	1UWE	L	AASQNVGTNVA	house mouse	IGKV6-15

L1-10,11,12-A	CDRL1	1IGT	C	HASQNINVWLS	house mouse	IGKV10-94
L1-10,11,12-A	CDRL1	2DQF	D	RASQSIGNNLH	house mouse	IGKV5-43
L1-10,11,12-A	CDRL1	3MOA	L	RASQGVTSALA	human	IGKV1-13
L1-10,11,12-A	CDRL1	3HNV	L	RASQDIGSYLN	house mouse	IGKV10-96
L1-10,11,12-A	CDRL1	2FJF	S	RASQDVSTAVA	human	IGKV1-39
L1-10,11,12-A	CDRL1	1U95	A	RASQGVTSALA	human	IGKV1D-13
L1-10,11,12-A	CDRL1	2F19	L	RASQDISNYLN	house mouse	IGKV10-96
L1-10,11,12-A	CDRL1	3EO0	A	RASQSLGSSYLA	human	IGKV3-20
L1-10,11,12-A	CDRL1	4NCC	L	SASSSVSYMH	house mouse	IGKV4-57
L1-10,11,12-A	CDRL1	3KYM	K	RASQSVSSSYLA	human	IGKV3-15
L1-10,11,12-A	CDRL1	1DQL	L	RASQDIRNDLG	human	IGKV1-6
L1-10,11,12-A	CDRL1	2V7H	L	RASQDINNYLN	house mouse	IGKV10-96
L1-10,11,12-A	CDRL1	4CAD	D	RASGNIHNYLA	house mouse	IGKV12-41
L1-10,11,12-A	CDRL1	3CDY	A	QASQDINNYLI	human	IGKV1-33
L1-10,11,12-A	CDRL1	3A6B	L	RASQSIGNDLH	house mouse	IGKV5-43
L1-10,11,12-A	CDRL1	2ITD	B	RASQSIGTDIH	house mouse	IGKV5-48
L1-10,11,12-A	CDRL1	2AJ3	C	RASQDIQKFLA	human	IGKV1-9
L1-10,11,12-A	CDRL1	1HH6	A	KASQDINSFLT	house mouse	IGKV14-111
L1-10,11,12-A	CDRL1	3NFS	L	SASSSISYMH	human	IGKV1-5
L1-10,11,12-A	CDRL1	1A14	L	RASQDISNYLN	house mouse	IGKV10-96
L1-10,11,12-A	CDRL1	1CIC	C	RASGNIHNYLA	house mouse	IGKV12-41
L1-10,11,12-A	CDRL1	3KYM	A	RASQSVSSSYLA	human	IGKV3-15
L1-10,11,12-A	CDRL1	2VDO	L	HASQGISSNIG	house mouse	IGKV14-100
L1-10,11,12-A	CDRL1	2NY2	C	RASESVSSDLA	human	IGKV3-15
L1-10,11,12-A	CDRL1	4JHA	L	QASQDIVNYLN	human	IGKV1-33
L1-10,11,12-A	CDRL1	3OAZ	K	RASQSIETWLA	human	IGKV1-5
L1-10,11,12-A	CDRL1	1D6V	L	KASQDINSYLS	house mouse	IGKV14-111
L1-10,11,12-A	CDRL1	1VGE	L	RASQGISSALA	human	IGKV1-13
L1-10,11,12-A	CDRL1	2BMK	A	SATSGVNYMH	house mouse	IGKV4-57
L1-10,11,12-A	CDRL1	4G5Z	L	RASQSIGSSLH	human	IGKV1-39
L1-10,11,12-A	CDRL1	3IU4	L	KASQDVSTAVA	house mouse	IGKV6-17
L1-10,11,12-A	CDRL1	3V6O	F	KATQNVRTAVT	house mouse	IGKV6-14
L1-10,11,12-A	CDRL1	4NZR	L	RASQNINKNLA	human	IGKV3-15
L1-10,11,12-A	CDRL1	2HVK	B	RASQSIGTDIH	house mouse	IGKV5-48
L1-10,11,12-A	CDRL1	4KPH	M	SASSSVNYMH	house mouse	IGKV4-80
L1-10,11,12-A	CDRL1	1FVE	A	RASQDVNTAVA	human	IGKV1-39
L1-10,11,12-A	CDRL1	2HFF	L	RASQDVSTAVA	human	IGKV1-39
L1-10,11,12-A	CDRL1	3STZ	B	RASQSIGTDIH	house mouse	IGKV5-48
L1-10,11,12-A	CDRL1	2FR4	L	RASENIYSYLA	house mouse	IGKV12-44
L1-10,11,12-A	CDRL1	3CLF	L	KASQNVGNIIA	house mouse	IGKV6-15
L1-10,11,12-A	CDRL1	4JRE	L	QASQNITNYIV	house mouse	IGKV19-93
L1-10,11,12-A	CDRL1	1CLO	L	RASSSVTYIH	house mouse	IGKV4-72
L1-10,11,12-A	CDRL1	3PNW	A	RASQSVSSAVA	human	IGKV1-39
L1-10,11,12-A	CDRL1	3EYO	A	RASQSVGGYLT	human	IGKV3-11
L1-10,11,12-A	CDRL1	3CVI	L	KASEDIYNRLA	house mouse	IGKV13-84
L1-10,11,12-A	CDRL1	4F33	E	SASSSVSYMH	house mouse	IGKV4-59
L1-10,11,12-A	CDRL1	1BM3	L	RASQSISNNLH	house mouse	IGKV5-43
L1-10,11,12-A	CDRL1	1RZ8	A	RASESVSSDLA	human	IGKV3-15
L1-10,11,12-A	CDRL1	3MXV	L	KASQSVSNDLT	house mouse	IGKV6-32
L1-10,11,12-A	CDRL1	1G7I	A	RASGNIHNYLA	house mouse	IGKV12-41

L1-10,11,12-A	CDRL1	4NCC	1	SASSSVSYMH	house mouse	IGKV4-57
L1-10,11,12-A	CDRL1	2QQN	L	RASQYFSSYLA	human	IGKV1-5
L1-10,11,12-A	CDRL1	4F33	A	SASSSVSYMH	house mouse	IGKV4-59
L1-10,11,12-A	CDRL1	4LSP	L	QASRGIGKDLN	human	IGKV1-NL1
L1-10,11,12-A	CDRL1	4AL8	L	KASQNVRTSVA	human	IGKV1-17
L1-10,11,12-A	CDRL1	1TJH	L	RASQGVTSALA	human	IGKV1D-13
L1-10,11,12-A	CDRL1	3L5Y	L	RASKSISKYLA	house mouse	IGKV12-44
L1-10,11,12-A	CDRL1	1IGC	L	KASENVVTYVS	house mouse	IGKV6-20
L1-10,11,12-A	CDRL1	1HI6	A	KASQDINSFLT	house mouse	IGKV14-111
L1-10,11,12-A	CDRL1	4I2X	C	KASQDVSTAVA	human	IGKV6-17
L1-10,11,12-A	CDRL1	3ZDY	L	HASQGISSNIG	house mouse	IGKV14-100
L1-10,11,12-A	CDRL1	3RVW	C	KASQDIYSYLS	house mouse	IGKV14-111
L1-10,11,12-A	CDRL1	1IKF	L	RASQDISTYLN	house mouse	IGKV10-96
L1-10,11,12-A	CDRL1	1U8Q	A	RASQGVTSALA	human	IGKV1D-13
L1-10,11,12-A	CDRL1	1YYL	Q	RASESVSSDLA	human	IGKV3-15
L1-10,11,12-A	CDRL1	2R8S	L	RASQSVSSAVA	human	IGKV1-39
L1-10,11,12-A	CDRL1	2W9D	L	SASSSVSYMH	house mouse	IGKV4-59
L1-10,11,12-A	CDRL1	2ADG	A	ITSTDIDDDMN	house mouse	IGKV17-121
L1-10,11,12-A	CDRL1	2H9G	L	RASQDVSTAVA	human	IGKV1-39
L1-10,11,12-A	CDRL1	3U79	E	QASQDISNFLI	human	IGKV1-33
L1-10,11,12-A	CDRL1	1K6Q	L	KASRDIKSYLS	house mouse	IGKV14-126
L1-10,11,12-A	CDRL1	1L7I	L	KASQDVSIGVA	human	IGKV1-16
L1-10,11,12-A	CDRL1	2PR4	L	RASQGVTSALA	human	IGKV1D-13
L1-10,11,12-A	CDRL1	1CFQ	A	KASQDINSFLT	house mouse	IGKV14-111
L1-10,11,12-A	CDRL1	4CAD	G	RASGNIHNYLA	house mouse	IGKV12-41
L1-10,11,12-A	CDRL1	3HI6	Y	RASQSIGSYLN	human	IGKV1-39
L1-10,11,12-A	CDRL1	1FJ1	A	KASQDINKYIA	house mouse	IGKV19-93
L1-10,11,12-A	CDRL1	1ZLV	L	RASQSIETWLA	human	IGKV1-5
L1-10,11,12-A	CDRL1	1YQV	L	SASSSVNYMY	house mouse	IGKV4-59
L1-10,11,12-A	CDRL1	2R56	L	RASQGISSRLA	human	IGKV1D-16
L1-10,11,12-A	CDRL1	3OAY	K	RASQSIETWLA	human	IGKV1-5
L1-10,11,12-A	CDRL1	1VFB	A	RASGNIHNYLA	house mouse	IGKV12-41
L1-10,11,12-A	CDRL1	1OPG	L	RASQSISNNLH	house mouse	IGKV5-43
L1-10,11,12-A	CDRL1	2DWE	B	RASQSIGTDIH	house mouse	IGKV5-48
L1-10,11,12-A	CDRL1	3EYQ	C	RASQSVSSYLA	human	IGKV3-11
L1-10,11,12-A	CDRL1	1OM3	M	RASQSIETWLA	human	IGKV1-5
L1-10,11,12-A	CDRL1	1OTS	F	SASSSVSYIH	house mouse	IGKV4-59
L1-10,11,12-A	CDRL1	3QPX	L	KASQNIYNYLN	Norway rat	IGKV22S9
L1-10,11,12-A	CDRL1	3K2U	L	RASQDVSTAVA	human	IGKV1D-13
L1-10,11,12-A	CDRL1	1FNS	L	SASQDINKYLN	house mouse	IGKV10-94
L1-10,11,12-A	CDRL1	1BJ1	L	SASQDISNYLN	human	IGKV1-33
L1-10,11,12-A	CDRL1	4N9G	F	RASQGISNYLA	human	IGKV1-16
L1-10,11,12-A	CDRL1	3G5X	A	HSSQDINSNIG	house mouse	IGKV14-100
L1-10,11,12-A	CDRL1	1ZAN	L	RASEDIYNALA	Norway rat	IGKV12S34
L1-10,11,12-A	CDRL1	4D9R	L	ITSTDIDDDMN	human	IGKV1-27
L1-10,11,12-A	CDRL1	2BX5	M	RASQSISSYLN	human	IGKV1-39
L1-10,11,12-A	CDRL1	2I60	Q	RASESVSSDLA	human	IGKV3-15
L1-10,11,12-A	CDRL1	1QBM	L	RASGNIHNYLA	house mouse	IGKV12-41
L1-10,11,12-A	CDRL1	1P7K	A	RASENIYSYLA	house mouse	IGKV12-44
L1-10,11,12-A	CDRL1	3L95	A	RASQDVSTAVA	human	IGKV1-39

L1-10,11,12-A	CDRL1	1EMT	L	SASQDISNYLN	house mouse	IGKV10-94
L1-10,11,12-A	CDRL1	2VDP	L	HASQGISSNIG	house mouse	IGKV14-100
L1-10,11,12-A	CDRL1	3UPA	A	RASQSISSYLN	human	IGKV1-39
L1-10,11,12-A	CDRL1	1KB5	L	RASKNIYSYLA	house mouse	IGKV12-44
L1-10,11,12-A	CDRL1	2VDR	L	HASQGISSNIG	house mouse	IGKV14-100
L1-10,11,12-A	CDRL1	2QSC	L	QASQDISNYLN	human	IGKV1-33
L1-10,11,12-A	CDRL1	2F5B	L	RASQGVTSALA	human	IGKV1D-13
L1-10,11,12-A	CDRL1	1UAC	L	RASQSIGNNLH	house mouse	IGKV5-43
L1-10,11,12-A	CDRL1	2ADI	A	ITSTDIDDDMN	house mouse	IGKV17-121
L1-10,11,12-A	CDRL1	1E4X	M	RASQDISHYLN	house mouse	IGKV10-96
L1-10,11,12-A	CDRL1	1NMB	L	RASQDISNYLN	house mouse	IGKV10-96
L1-10,11,12-A	CDRL1	3OR7	B	RASQSIGTDIH	house mouse	IGKV5-48
L1-10,11,12-A	CDRL1	1N7M	H	KASQNVGTAVA	house mouse	IGKV6-13
L1-10,11,12-A	CDRL1	4NZU	L	QASQDIAKFLD	human	IGKV1-33
L1-10,11,12-A	CDRL1	1YY9	C	RASQSIGTNIH	house mouse	IGKV5-48
L1-10,11,12-A	CDRL1	3CFD	A	RASQDISNYLN	house mouse	IGKV10-96
L1-10,11,12-A	CDRL1	2BX5	B	RASQSISSYLN	human	IGKV1-39
L1-10,11,12-A	CDRL1	4IOI	A	RASQDVNTAVA	human	IGKV1-39
L1-10,11,12-A	CDRL1	3QPQ	L	RASGNIHNYLA	house mouse	IGKV12-41
L1-10,11,12-A	CDRL1	2NY3	C	RASESVSSDLA	human	IGKV3-15
L1-10,11,12-A	CDRL1	1J1P	L	RASQSIGNNLH	house mouse	IGKV5-43
L1-10,11,12-A	CDRL1	3CDF	A	QASQDISNYLN	human	IGKV1-33
L1-10,11,12-A	CDRL1	1MLC	A	RASQSISNNLH	house mouse	IGKV5-43
L1-10,11,12-A	CDRL1	1J1O	L	RASQSIGNNLH	house mouse	IGKV5-43
L1-10,11,12-A	CDRL1	3MCL	L	SASSSVSYMH	house mouse	IGKV4-59
L1-10,11,12-A	CDRL1	1KIP	A	RASGNIHNYLA	house mouse	IGKV12-41
L1-10,11,12-A	CDRL1	1D5B	L	KASQDINSYLN	house mouse	IGKV14-111
L1-10,11,12-A	CDRL1	2B2X	M	SASSQVNHMF	house mouse	IGKV4-68
L1-10,11,12-A	CDRL1	2VXQ	L	RASQRINTYLN	human	IGKV1-6
L1-10,11,12-A	CDRL1	3U79	C	QASQDISNFLI	human	IGKV1-33
L1-10,11,12-A	CDRL1	1G9M	L	RASESVSSDLA	human	IGKV3-15
L1-10,11,12-A	CDRL1	2J88	L	RASENIYSYLT	house mouse	IGKV12-44
L1-10,11,12-A	CDRL1	3U9P	M	QASQIIYNYIA	Norway rat	IGKV19S2
L1-10,11,12-A	CDRL1	2FJF	A	RASQDVSTAVA	human	IGKV1-39
L1-10,11,12-A	CDRL1	4LLY	D	KASQDVSIGVA	human	IGKV1D-13
L1-10,11,12-A	CDRL1	3EOA	A	RASKTISKYLA	human	IGKV1-16
L1-10,11,12-A	CDRL1	2BX5	A	RASQSISSYLN	human	IGKV1-39
L1-10,11,12-A	CDRL1	3FCT	C	KASQNVGTPVA	house mouse	IGKV6-13
L1-10,11,12-A	CDRL1	2BX5	K	RASQSISSYLN	human	IGKV1-39
L1-10,11,12-A	CDRL1	1AJ7	L	RASQEISGYLS	house mouse	IGKV9-124
L1-10,11,12-A	CDRL1	4LBE	B	RASQSIGTDIH	house mouse	IGKV5-48
L1-10,11,12-A	CDRL1	3IXT	B	SASSRVGYMH	human	IGKV1-5
L1-10,11,12-A	CDRL1	3B9K	L	QASQNIDKYIA	Norway rat	IGKV19S2
L1-10,11,12-A	CDRL1	1DVF	C	RASQDISNYLN	house mouse	IGKV10-96
L1-10,11,12-A	CDRL1	3U30	B	RASQDVSTAVA	human	IGKV1-39
L1-10,11,12-A	CDRL1	2AJ3	E	RASQDIQKFLA	human	IGKV1-9
L1-10,11,12-A	CDRL1	3IDN	A	RASQGVTSALA	human	IGKV1-13
L1-10,11,12-A	CDRL1	1EAP	A	KASQDIKKYIG	house mouse	IGKV19-93
L1-10,11,12-A	CDRL1	2Q1E	D	QASQDINNYLI	human	IGKV1-33
L1-10,11,12-A	CDRL1	1RZG	B	RASQSISNWLA	human	IGKV1-5

L1-10,11,12-A	CDRL1	1BWW	B	QASQDIIKYLN	human	IGKV1-33
L1-10,11,12-A	CDRL1	3IDM	A	RASQGVTSALA	human	IGKV1-13
L1-10,11,12-A	CDRL1	4DKF	M	RASQSISSYLA	human	IGKV1-39
L1-10,11,12-A	CDRL1	1U92	A	RASQGVTSALA	human	IGKV1D-13
L1-10,11,12-A	CDRL1	3MXW	L	KASQSVSNDLT	human	IGKV1-33
L1-10,11,12-A	CDRL1	1FN4	C	KGSQNINNYLA	Norway rat	IGKV22S2
L1-10,11,12-A	CDRL1	3L5W	L	RASKSISKYLA	house mouse	IGKV16-104
L1-10,11,12-A	CDRL1	3FN0	L	RASQSVGRNLG	human	IGKV3D-11
L1-10,11,12-A	CDRL1	4D9R	D	ITSTDIDDDMN	human	IGKV1-27
L1-10,11,12-A	CDRL1	2XKN	C	RASQDISNYLN	house mouse	IGKV10-96
L1-10,11,12-A	CDRL1	3G5Y	A	HSSQDISSNIG	house mouse	IGKV14-100
L1-10,11,12-A	CDRL1	1DQM	L	RASQSISNNLH	house mouse	IGKV5-43
L1-10,11,12-A	CDRL1	3EHB	D	RASENIYSYLA	house mouse	IGKV12-44
L1-10,11,12-A	CDRL1	1AY1	L	SASSSVSYMY	house mouse	IGKV4-55
L1-10,11,12-A	CDRL1	3V0W	L	RASQNINIWLS	house mouse	IGKV11-125
L1-10,11,12-A	CDRL1	1KIR	A	RASGNIHNYLA	house mouse	IGKV12-41
L1-10,11,12-A	CDRL1	3HC3	L	KASQNVGINVA	human	IGKV1-16
L1-10,11,12-A	CDRL1	3HI5	L	RASQSIGSYLN	human	IGKV1-39
L1-10,11,12-A	CDRL1	1A6T	A	SPSSSVSYMQ	house mouse	IGKV4-72
L1-10,11,12-A	CDRL1	3VFG	L	KASQSVSNDVT	house mouse	IGKV6-32
L1-10,11,12-A	CDRL1	4G6J	L	RASQSIGSSLH	human	IGKV1-39
L1-10,11,12-A	CDRL1	2WUC	L	RASQDVSTAVA	human	IGKV1D-13
L1-10,11,12-A	CDRL1	2NY6	C	RASESVSSDLA	human	IGKV3-15
L1-10,11,12-A	CDRL1	1A0Q	L	KASQDIKKYIG	house mouse	IGKV19-93
L1-10,11,12-A	CDRL1	1JHL	L	RASKSISKSLA	house mouse	IGKV16-104
L1-10,11,12-A	CDRL1	1QP1	B	QASQDISDYLI	human	IGKV1-33
L1-10,11,12-A	CDRL1	4LLU	D	KASQDVSIGVA	human	IGKV1-16
L1-10,11,12-A	CDRL1	1G7M	A	RASGNIHNYLA	house mouse	IGKV12-41
L1-10,11,12-A	CDRL1	3RVX	C	KASQDIYSYLS	house mouse	IGKV14-111
L1-10,11,12-A	CDRL1	3QPQ	I	RASGNIHNYLA	house mouse	IGKV12-41
L1-10,11,12-A	CDRL1	1BRE	D	QASQDISDYLI	human	IGKV1-33
L1-10,11,12-A	CDRL1	2FJF	Q	RASQDVSTAVA	human	IGKV1-39
L1-10,11,12-A	CDRL1	1FDL	L	RASGNIHNYLA	house mouse	IGKV12-41
L1-10,11,12-A	CDRL1	1E6O	L	SASSSVSYMH	house mouse	IGKV4-86
L1-10,11,12-A	CDRL1	1U8I	A	RASQGVTSALA	human	IGKV1D-13
L1-10,11,12-A	CDRL1	3FCT	A	KASQNVGTPVA	house mouse	IGKV6-13
L1-10,11,12-A	CDRL1	1UYW	N	KASENVVTYVS	house mouse	IGKV6-20
L1-10,11,12-A	CDRL1	1XF4	A	RASENIYSYLA	house mouse	IGKV12-44
L1-10,11,12-A	CDRL1	3MOB	L	RASQGVTSALA	human	IGKV1-13
L1-10,11,12-A	CDRL1	2BX5	C	RASQSISSYLN	human	IGKV1-39
L1-10,11,12-A	CDRL1	3KR3	L	RASQSISNYLN	human	IGKV1-39
L1-10,11,12-A	CDRL1	3RA7	L	RASQDIKNYLN	house mouse	IGKV10-96
L1-10,11,12-A	CDRL1	1NGX	A	KASQNVGTAVA	house mouse	IGKV6-13
L1-10,11,12-A	CDRL1	3OAZ	L	RASQSIETWLA	human	IGKV1-5
L1-10,11,12-A	CDRL1	3L5X	L	RASKSISKYLA	human	IGKV3-11
L1-10,11,12-A	CDRL1	2VXV	L	RASESISSNLA	human	IGKV3-15
L1-10,11,12-A	CDRL1	3L5W	A	RASKSISKYLA	house mouse	IGKV16-104
L1-10,11,12-A	CDRL1	4G6K	L	RASQDISNYLS	human	IGKV1-39
L1-10,11,12-A	CDRL1	1DEE	E	RTSQSISSYLN	human	IGKV1-39
L1-10,11,12-A	CDRL1	1I8K	A	MTSTDIDDDMN	house mouse	IGKV17-127

L1-10,11,12-A	CDRL1	1UWE	X	AASQNVGTNVA	house mouse	IGKV6-15
L1-10,11,12-A	CDRL1	4LF3	D	RASQGIRNDLG	house mouse	IGKV11-125
L1-10,11,12-A	CDRL1	1MIM	L	SASSSRSYMQ	human	IGKV1-5
L1-10,11,12-A	CDRL1	3B2U	G	RASQSVSSYLA	human	IGKV3D-11
L1-10,11,12-A	CDRL1	1I3G	L	KASQDVGTAVA	house mouse	IGKV6-23
L1-10,11,12-A	CDRL1	2R0L	L	RASQDVSTAVA	human	IGKV1-39
L1-10,11,12-A	CDRL1	4JFX	A	MTSTDIDDDMN	human	IGKV3-11
L1-10,11,12-A	CDRL1	2Z91	B	SASSSVSSVH	house mouse	IGKV4-59
L1-10,11,12-A	CDRL1	3U79	G	QASQDISNFLI	human	IGKV1-33
L1-10,11,12-A	CDRL1	4H88	L	RASQNIGTSIH	house mouse	IGKV5-48
L1-10,11,12-A	CDRL1	2FJF	G	RASQDVSTAVA	human	IGKV1-39
L1-10,11,12-A	CDRL1	1UJ3	A	KASQDIKSFLS	human	IGKV1D-16
L1-10,11,12-A	CDRL1	1FVC	A	RASQDVNTAVA	human	IGKV1-39
L1-10,11,12-A	CDRL1	3B2U	K	RASQSVSSYLA	human	IGKV3D-11
L1-10,11,12-A	CDRL1	4MA7	L	RASQNIGTSIH	house mouse	IGKV5-48
L1-10,11,12-A	CDRL1	1IC7	L	RASQSIGNNLH	house mouse	IGKV5-43
L1-10,11,12-A	CDRL1	1R0A	L	SASQDISSYLN	house mouse	IGKV10-94
L1-10,11,12-A	CDRL1	1XIW	G	RASQDIRNYLN	house mouse	IGKV10-96
L1-10,11,12-A	CDRL1	1G7J	A	RASGNIHNYLA	house mouse	IGKV12-41
L1-10,11,12-A	CDRL1	1U8K	A	RASQGVTSALA	human	IGKV1D-13
L1-10,11,12-A	CDRL1	1FGN	L	KASQDIRKYLN	house mouse	IGKV14-126
L1-10,11,12-A	CDRL1	3PNW	V	RASQSVSSAVA	human	IGKV1-39
L1-10,11,12-A	CDRL1	1AXS	A	KASQDINSYLN	house mouse	IGKV14-111
L1-10,11,12-A	CDRL1	1FVD	A	RASQDVNTAVA	human	IGKV1-39
L1-10,11,12-A	CDRL1	3NCJ	L	RASQSIGLYLA	human	IGKV1-39
L1-10,11,12-A	CDRL1	1BFO	E	KASQNIDKYLN	Norway rat	IGKV22S7
L1-10,11,12-A	CDRL1	1MAM	L	RASQDIYNYLN	house mouse	IGKV10-96
L1-10,11,12-A	CDRL1	4ETQ	L	SASSSVSYMY	house mouse	IGKV4-55
L1-10,11,12-A	CDRL1	1YYM	Q	RASESVSSDLA	human	IGKV3-15
L1-10,11,12-A	CDRL1	3ZE2	F	HASQGISSNIG	house mouse	IGKV14-100
L1-10,11,12-A	CDRL1	2VDQ	L	HASQGISSNIG	house mouse	IGKV14-100
L1-10,11,12-A	CDRL1	2R56	M	RASQGISSRLA	human	IGKV1D-16
L1-10,11,12-A	CDRL1	2Z91	D	SASSSVSSVH	house mouse	IGKV4-59
L1-10,11,12-A	CDRL1	3EOA	L	RASKTISKYLA	human	IGKV1-16
L1-10,11,12-A	CDRL1	2FJF	L	RASQDVSTAVA	human	IGKV1-39
L1-10,11,12-A	CDRL1	1CFN	A	KASQDINSFLT	house mouse	IGKV14-111
L1-10,11,12-A	CDRL1	1ZLV	K	RASQSIETWLA	human	IGKV1-5
L1-10,11,12-A	CDRL1	2BX5	H	RASQSISSYLN	human	IGKV1-39
L1-10,11,12-A	CDRL1	1XF3	A	RASENIYSYLA	house mouse	IGKV12-44
L1-10,11,12-A	CDRL1	4MWF	L	RASQSVSSNLA	human	IGKV3-15
L1-10,11,12-A	CDRL1	1G7H	A	RASGNIHNYLA	house mouse	IGKV12-41
L1-10,11,12-A	CDRL1	4NRX	B	RASQSIGSYLN	human	IGKV1-39
L1-10,11,12-A	CDRL1	2Q8A	L	KASQSVSNDVV	house mouse	IGKV6-32
L1-10,11,12-A	CDRL1	4LCU	B	RASQSIGTDIH	house mouse	IGKV5-48
L1-10,11,12-A	CDRL1	4GW1	A	RASQSIGTNIH	house mouse	IGKV5-48
L1-10,11,12-A	CDRL1	2BX5	F	RASQSISSYLN	human	IGKV1-39
L1-10,11,12-A	CDRL1	1HH9	A	KASQDINSFLT	house mouse	IGKV14-111
L1-10,11,12-A	CDRL1	1KCV	L	KASENVGTYVS	house mouse	IGKV6-20
L1-10,11,12-A	CDRL1	4AG4	L	SASSSVTFMY	house mouse	IGKV4-68
L1-10,11,12-A	CDRL1	4HXA	L	SVSSSVNYMH	house mouse	IGKV4-63

L1-10,11,12-A	CDRL1	2I5Y	L	RASESVSSDLA	human	IGKV3-15
L1-10,11,12-A	CDRL1	1DQD	L	SASSSVSYMH	house mouse	IGKV4-57
L1-10,11,12-A	CDRL1	3U79	H	QASQDISNFLI	human	IGKV1-33
L1-10,11,12-A	CDRL1	3RVU	C	KASQDIYSYLS	house mouse	IGKV14-111
L1-10,11,12-A	CDRL1	1B6D	B	QASQDISSYLN	human	IGKV1-33
L1-10,11,12-A	CDRL1	2XRA	L	QASQDIRNHLN	human	IGKV1-33
L1-10,11,12-A	CDRL1	12E8	M	KASQNVGTAVA	house mouse	IGKV6-13
L1-10,11,12-A	CDRL1	6FAB	L	RASQDINNFLN	house mouse	IGKV10-96
L1-10,11,12-A	CDRL1	4KI5	F	RASQEISGYLS	house mouse	IGKV9-124
L1-10,11,12-A	CDRL1	1WTL	A	RASQDITNYVN	human	IGKV1-33
L1-10,11,12-A	CDRL1	1E4X	L	RASQDISHYLN	house mouse	IGKV10-96
L1-10,11,12-A	CDRL1	4ENE	F	SASSSVSYIH	house mouse	IGKV4-59
L1-10,11,12-A	CDRL1	1RIH	L	RASQSISNNLH	house mouse	IGKV5-43
L1-10,11,12-A	CDRL1	4F3F	A	SASSSVSYMH	house mouse	IGKV4-59
L1-10,11,12-A	CDRL1	3B2U	U	RASQSVSSYLA	human	IGKV3D-11
L1-10,11,12-A	CDRL1	1UM5	L	RASQEISGYLY	house mouse	IGKV9-124
L1-10,11,12-A	CDRL1	1CIC	A	KASQDVRIAVA	house mouse	IGKV6-23
L1-10,11,12-A	CDRL1	4HHA	A	RASQSVGGYLT	human	IGKV3-11
L1-10,11,12-A	CDRL1	1LK3	M	KASESVTSRMH	Norway rat	IGKV3S11
L1-10,11,12-A	CDRL1	1XGT	A	RASQSISNNLH	house mouse	IGKV5-43
L1-10,11,12-A	CDRL1	3U79	F	QASQDISNFLI	human	IGKV1-33
L1-10,11,12-A	CDRL1	2P8L	A	RASQGVTSALA	human	IGKV1D-13
L1-10,11,12-A	CDRL1	4DGV	L	RASQSVSSYLA	human	IGKV3-11
L1-10,11,12-A	CDRL1	1QP1	C	QASQDISDYLI	human	IGKV1-33
L1-10,11,12-A	CDRL1	1YJD	L	HASQNIYVWLN	house mouse	IGKV10-94
L1-10,11,12-A	CDRL1	1ZLS	L	RASQSIETWLA	human	IGKV1-5
L1-10,11,12-A	CDRL1	2OSL	L	RASSSVSYIH	house mouse	IGKV4-72
L1-10,11,12-A	CDRL1	1R3J	A	RASQSIGTDIH	house mouse	IGKV5-48
L1-10,11,12-A	CDRL1	2IH1	B	RASQSIGTDIH	house mouse	IGKV5-48
L1-10,11,12-A	CDRL1	1YYM	L	RASESVSSDLA	human	IGKV3-15
L1-10,11,12-A	CDRL1	1IC5	L	RASQSIGNNLH	house mouse	IGKV5-43
L1-10,11,12-A	CDRL1	1OP3	L	RASQSIETWLA	human	IGKV1-5
L1-10,11,12-A	CDRL1	1OP3	K	RASQSIETWLA	human	IGKV1-5
L1-10,11,12-A	CDRL1	1OSP	L	KASEDIYSRLA	house mouse	IGKV13-84
L1-10,11,12-A	CDRL1	1PG7	M	RASRDIKSYLN	human	IGKV1-39
L1-10,11,12-A	CDRL1	3B2U	L	RASQSVSSYLA	human	IGKV3D-11
L1-10,11,12-A	CDRL1	1A7N	L	RASGNIHNYLA	house mouse	IGKV12-41
L1-10,11,12-A	CDRL1	1C08	A	RASQSIGNNLH	house mouse	IGKV5-43
L1-10,11,12-A	CDRL1	2BDN	L	KATEDIYNRLA	house mouse	IGKV13-84
L1-10,11,12-A	CDRL1	1FJ1	C	KASQDINKYIA	house mouse	IGKV19-93
L1-10,11,12-A	CDRL1	2QQK	L	RASQDVSTAVA	human	IGKV1D-13
L1-10,11,12-A	CDRL1	3GRW	L	RASQDVSTAVA	human	IGKV1-39
L1-10,11,12-A	CDRL1	1JGL	L	RASGNIHNYLA	house mouse	IGKV12-41
L1-10,11,12-A	CDRL1	3L7F	L	RASKSISKYLA	human	IGKV3-11
L1-10,11,12-A	CDRL1	4IJ3	B	KASQDVSTAVA	house mouse	IGKV6-17
L1-10,11,12-A	CDRL1	3KYM	M	RASQSVSSYLA	human	IGKV3-15
L1-10,11,12-A	CDRL1	1U8H	A	RASQGVTSALA	human	IGKV1D-13
L1-10,11,12-A	CDRL1	1DQQ	A	RASQSISNNLH	house mouse	IGKV5-43
L1-10,11,12-A	CDRL1	3CLE	L	KASQNVGNIIA	house mouse	IGKV6-15
L1-10,11,12-A	CDRL1	2YC1	B	RASQSVRSYLA	human	IGKV3D-11

L1-10,11,12-A	CDRL1	3CDF	C	QASQDISNYLN	human	IGKV1-33
L1-10,11,12-A	CDRL1	3Q1S	L	RASQSVGRNLG	human	IGKV3D-11
L1-10,11,12-A	CDRL1	2I60	L	RASESVSSDLA	human	IGKV3-15
L1-10,11,12-A	CDRL1	1B6D	A	QASQDISSYLN	human	IGKV1-33
L1-10,11,12-A	CDRL1	1BVK	A	RASGNIHNYLA	human	IGKV1-27
L1-10,11,12-A	CDRL1	2F5A	L	RASQGVTSALA	human	IGKV1D-13
L1-10,11,12-A	CDRL1	2HVJ	B	RASQSIGTDIH	house mouse	IGKV5-48
L1-10,11,12-A	CDRL1	4JO4	M	QASETISNYLA	rabbit	IGKV1S3
L1-10,11,12-A	CDRL1	3L7E	L	RASKSISKYLA	house mouse	IGKV16-104
L1-10,11,12-A	CDRL1	1HEZ	A	RTSQSISSYLN	human	IGKV1-39
L1-10,11,12-A	CDRL1	3G5X	C	HSSQDINSNIG	house mouse	IGKV14-100
L1-10,11,12-A	CDRL1	1UWX	K	LASQTIGTWLA	house mouse	IGKV12-98
L1-10,11,12-A	CDRL1	2VH5	L	RASQSISSYLN	human	IGKV1-39
L1-10,11,12-A	CDRL1	4KVC	L	KASENVVTYVS	house mouse	IGKV6-20
L1-10,11,12-A	CDRL1	2DQC	L	RASQSIGNNLH	house mouse	IGKV5-43
L1-10,11,12-A	CDRL1	2AEP	L	KASQNVGTNVA	house mouse	IGKV6-15
L1-10,11,12-A	CDRL1	2DQH	L	RASQSIGNNLH	house mouse	IGKV5-43
L1-10,11,12-A	CDRL1	3EYF	C	RASQSVGGYLT	human	IGKV3-11
L1-10,11,12-A	CDRL1	1S5H	A	RASQSIGTDIH	house mouse	IGKV5-48
L1-10,11,12-A	CDRL1	2FJF	O	RASQDVSTAVA	human	IGKV1-39
L1-10,11,12-A	CDRL1	2OQJ	A	RASQSIETWLA	human	IGKV1-5
L1-10,11,12-A	CDRL1	4KI5	D	SASSTVSYMY	house mouse	IGKV4-55
L1-10,11,12-A	CDRL1	4HKZ	A	RASQDVNTAVA	human	IGKV1-39
L1-10,11,12-A	CDRL1	2V17	L	RASKSIRKFLA	house mouse	IGKV16-104
L1-10,11,12-A	CDRL1	1GHF	L	RESQDISNSLN	house mouse	IGKV10-96
L1-10,11,12-A	CDRL1	4N9G	L	RASQGISNYLA	human	IGKV1-16
L1-10,11,12-A	CDRL1	2VDK	L	HASQGISSNIG	house mouse	IGKV14-100
L1-10,11,12-A	CDRL1	1U91	A	RASQGVTSALA	human	IGKV1D-13
L1-10,11,12-A	CDRL1	1AD0	A	RASSSVTYIH	human	IGKV1-16
L1-10,11,12-A	CDRL1	4DGY	L	RASQSVSSYLA	human	IGKV3-11
L1-10,11,12-A	CDRL1	3U30	E	RASQDVSTAVA	human	IGKV1-39
L1-10,11,12-A	CDRL1	1A2Y	A	RASGNIHNYLA	house mouse	IGKV12-41
L1-10,11,12-A	CDRL1	1DQJ	A	RASQSISNNLH	house mouse	IGKV5-43
L1-10,11,12-A	CDRL1	1IGT	A	HASQNINVWLS	house mouse	IGKV10-94
L1-10,11,12-A	CDRL1	3HNT	L	RASQDIGSYLN	house mouse	IGKV10-96
L1-10,11,12-A	CDRL1	2Q1E	A	QASQDINNYLI	human	IGKV1-33
L1-10,11,12-A	CDRL1	4MA8	L	RASQNIGTSIH	house mouse	IGKV5-48
L1-10,11,12-A	CDRL1	1JHK	L	RASGNIHNYLA	house mouse	IGKV12-41
L1-10,11,12-A	CDRL1	2FJG	A	RASQDVSTAVA	human	IGKV1-39
L1-10,11,12-A	CDRL1	1MEX	L	KASQNVGTNVA	house mouse	IGKV6-15
L1-10,11,12-A	CDRL1	3EYF	A	RASQSVGGYLT	human	IGKV3-11
L1-10,11,12-A	CDRL1	3QPQ	C	RASGNIHNYLA	house mouse	IGKV12-41
L1-10,11,12-A	CDRL1	4ENE	D	SASSSVSYIH	house mouse	IGKV4-59
L1-10,11,12-A	CDRL1	2XKN	A	RASQDISNYLN	house mouse	IGKV10-96
L1-10,11,12-A	CDRL1	3D0V	A	RASQGVTSALA	human	IGKV1D-13
L1-10,11,12-A	CDRL1	4HH9	C	RASQSVGGYLT	human	IGKV3-11
L1-10,11,12-A	CDRL1	1PG7	L	RASRDIKSYLN	human	IGKV1-39
L1-10,11,12-A	CDRL1	1XGR	A	RASQSISNNLH	house mouse	IGKV5-43
L1-10,11,12-A	CDRL1	1QBL	L	RASGNIHNYLA	house mouse	IGKV12-41
L1-10,11,12-A	CDRL1	1U93	A	RASQGVTSALA	human	IGKV1D-13

L1-10,11,12-A	CDRL1	1V7M	L	SASSSVSYMY	house mouse	IGKV4-57
L1-10,11,12-A	CDRL1	2NLJ	A	RASQSIGTDIH	house mouse	IGKV5-48
L1-10,11,12-A	CDRL1	1MHP	Y	SASSSVNHMF	human	IGKV1D-13
L1-10,11,12-A	CDRL1	1OAK	L	SASQDINKYLN	house mouse	IGKV10-94
L1-10,11,12-A	CDRL1	2A6J	L	RASQDISNYLN	house mouse	IGKV10-96
L1-10,11,12-A	CDRL1	2HFF	A	RASQDVSTAVA	human	IGKV1-39
L1-10,11,12-A	CDRL1	2P7T	B	RASQSIGTDIH	house mouse	IGKV5-48
L1-10,11,12-A	CDRL1	1ZLU	L	RASQSIETWLA	human	IGKV1-5
L1-10,11,12-A	CDRL1	2FJF	J	RASQDVSTAVA	human	IGKV1-39
L1-10,11,12-A	CDRL1	2FJF	C	RASQDVSTAVA	human	IGKV1-39
L1-10,11,12-A	CDRL1	1NCC	L	KASQDVSTAVV	house mouse	IGKV6-25
L1-10,11,12-A	CDRL1	4JO3	M	QASETISNYLA	rabbit	IGKV1S3
L1-10,11,12-A	CDRL1	1CZ8	L	SASQDISNYLN	human	IGKV1-16
L1-10,11,12-A	CDRL1	2CMR	L	RASEGIYHWLA	human	IGKV1-5
L1-10,11,12-A	CDRL1	4L5F	L	ITSTDIDDDMN	house mouse	IGKV17-127
L1-10,11,12-A	CDRL1	2Q76	C	RASQSISNNLH	house mouse	IGKV5-43
L1-10,11,12-A	CDRL1	2I5Y	Q	RASESVSSDLA	human	IGKV3-15
L1-10,11,12-A	CDRL1	2J4W	L	RARSSVSYMH	house mouse	IGKV4-72
L1-10,11,12-A	CDRL1	2BX5	I	RASQSISSYLN	human	IGKV1-39
L1-10,11,12-A	CDRL1	2YBR	E	RASQSVRSYLA	human	IGKV3D-11
L1-10,11,12-A	CDRL1	1A7O	L	RASGNIHNYLA	house mouse	IGKV12-41
L1-10,11,12-A	CDRL1	3NAA	L	RASQSIGLYLA	human	IGKV1-39
L1-10,11,12-A	CDRL1	4HJG	A	RASQDVNTAVA	human	IGKV1-39
L1-10,11,12-A	CDRL1	3I9G	L	ITTTDIDDDMN	house mouse	IGKV17-121
L1-10,11,12-A	CDRL1	1A6T	C	SPSSSVSYMQ	house mouse	IGKV4-72
L1-10,11,12-A	CDRL1	2IFF	L	SASSSVNYMY	house mouse	IGKV4-59
L1-10,11,12-A	CDRL1	4CAD	A	RASGNIHNYLA	house mouse	IGKV12-41
L1-10,11,12-A	CDRL1	3LIZ	L	KASQDINNYLS	house mouse	IGKV14-111
L1-10,11,12-A	CDRL1	3PNW	G	RASQSVSSAVA	human	IGKV1-39
L1-10,11,12-A	CDRL1	1DQQ	C	RASQSISNNLH	house mouse	IGKV5-43
L1-10,11,12-A	CDRL1	3CDC	A	QASQDISNYLI	human	IGKV1-33
L1-10,11,12-A	CDRL1	3OR6	B	RASQSIGTDIH	house mouse	IGKV5-48
L1-10,11,12-A	CDRL1	1P2C	D	RASQSISNNLH	house mouse	IGKV5-43
L1-10,11,12-A	CDRL1	2DQF	A	RASQSIGNNLH	house mouse	IGKV5-43
L1-10,11,12-A	CDRL1	3U7A	A	QASQDINNFLI	human	IGKV1-33
L1-10,11,12-A	CDRL1	3CDF	B	QASQDISNYLN	human	IGKV1-33
L1-10,11,12-A	CDRL1	2EKS	A	RASQSIGNNLH	human	IGKV3-15
L1-10,11,12-A	CDRL1	1JV5	A	RASQDINNYLN	human	IGKV1-33
L1-10,11,12-A	CDRL1	2VDL	L	HASQGISSNIG	house mouse	IGKV14-100
L1-10,11,12-A	CDRL1	3DVI	A	QASQDISNYLI	human	IGKV1-33
L1-10,11,12-A	CDRL1	1HKL	L	RASQEINGYLG	house mouse	IGKV9-124
L1-10,11,12-A	CDRL1	1R3K	A	RASQSIGTDIH	house mouse	IGKV5-48
L1-10,11,12-A	CDRL1	2Q1E	C	QASQDINNYLI	human	IGKV1-33
L1-10,11,12-A	CDRL1	3U6R	L	RASQSVNKYLA	human	IGKV3-11
L1-10,11,12-A	CDRL1	1GAF	L	RASQEINGYLG	house mouse	IGKV9-124
L1-10,11,12-A	CDRL1	3RVT	C	KASQDIYSYLS	house mouse	IGKV14-111
L1-10,11,12-A	CDRL1	4KPH	L	SASSSVNYMH	house mouse	IGKV4-80
L1-10,11,12-A	CDRL1	4FFV	C	RASSSVNNMH	house mouse	IGKV4-72
L1-10,11,12-A	CDRL1	3BN9	C	RASQGISSYLA	human	IGKV1-12
L1-10,11,12-A	CDRL1	3U6R	B	RASQSVNKYLA	human	IGKV3-11

L1-10,11,12-A	CDRL1	4N9G	N	RASQGISNYLA	human	IGKV1-16
L1-10,11,12-A	CDRL1	2DQD	L	RASQSIGNNLH	house mouse	IGKV5-43
L1-10,11,12-A	CDRL1	4ETQ	B	SASSSVSYMY	house mouse	IGKV4-55
L1-10,11,12-A	CDRL1	3IXT	L	SASSRVGYMH	human	IGKV1-5
L1-10,11,12-A	CDRL1	1FVD	C	RASQDVNTAVA	human	IGKV1-39
L1-10,11,12-A	CDRL1	1JPS	L	RASRDIKSYLN	human	IGKV1-39
L1-10,11,12-A	CDRL1	3HNS	L	RASQDIGSYLN	house mouse	IGKV10-96
L1-10,11,12-A	CDRL1	1ZLU	K	RASQSIETWLA	human	IGKV1-5
L1-10,11,12-A	CDRL1	3DRQ	A	RASQGVTSALA	human	IGKV1D-13
L1-10,11,12-A	CDRL1	1WCB	L	SATSGVNYMH	house mouse	IGKV4-57
L1-10,11,12-A	CDRL1	1FE8	L	RASQDIGNYLN	house mouse	IGKV10-96
L1-10,11,12-A	CDRL1	2VXU	M	RASQDIGSKLY	house mouse	IGKV9-120
L1-10,11,12-A	CDRL1	2VXT	L	RASQDIGSKLY	house mouse	IGKV9-120
L1-10,11,12-A	CDRL1	1YY8	C	RASQSIGTNIH	house mouse	IGKV5-48
L1-10,11,12-A	CDRL1	4JFZ	L	MTSTDIDDDMN	human	IGKV3-11
L1-10,11,12-A	CDRL1	4GMT	M	KASQNVDTNVA	house mouse	IGKV6-15
L1-10,11,12-A	CDRL1	1CZ8	X	SASQDISNYLN	human	IGKV1-16
L1-10,11,12-A	CDRL1	3NIG	L	HASQGISSNIG	house mouse	IGKV14-100
L1-10,11,12-A	CDRL1	1AXS	L	KASQDINSYLN	house mouse	IGKV14-111
L1-10,11,12-A	CDRL1	3IDJ	A	RASQGVTSALA	human	IGKV1-13
L1-10,11,12-A	CDRL1	3B2U	R	RASQSVSSYLA	human	IGKV3D-11
L1-10,11,12-A	CDRL1	1NCA	L	KASQDVSTAVV	house mouse	IGKV6-25
L1-10,11,12-A	CDRL1	2Q20	A	QASQDISNYLN	human	IGKV1-33
L1-10,11,12-A	CDRL1	1BJ1	J	SASQDISNYLN	human	IGKV1-33
L1-10,11,12-A	CDRL1	2Q20	B	QASQDISNYLN	human	IGKV1-33
L1-10,11,12-A	CDRL1	4D9Q	L	ITSTDIDDDMN	human	IGKV1-27
L1-10,11,12-A	CDRL1	2NY0	C	RASESVSSDLA	human	IGKV3-15
L1-10,11,12-A	CDRL1	3V0V	L	RASQNINIWLS	house mouse	IGKV11-125
L1-10,11,12-A	CDRL1	1R3I	L	RASQSIGTDIH	house mouse	IGKV5-48
L1-10,11,12-A	CDRL1	1NCB	L	KASQDVSTAVV	house mouse	IGKV6-25
L1-10,11,12-A	CDRL1	3G5V	A	HSSQDINSNIG	house mouse	IGKV14-100
L1-10,11,12-A	CDRL1	1XF3	L	RASENIYSYLA	house mouse	IGKV12-44
L1-10,11,12-A	CDRL1	3UC0	M	RASQDISIRLN	human	IGKV1-13
L1-10,11,12-A	CDRL1	1BZ7	A	RASQDIGNFLN	house mouse	IGKV10-96
L1-10,11,12-A	CDRL1	4MSW	B	RASQSIGTDIH	house mouse	IGKV5-48
L1-10,11,12-A	CDRL1	2OQJ	J	RASQSIETWLA	human	IGKV1-5
L1-10,11,12-A	CDRL1	3DVF	A	QASQDITNHLN	human	IGKV1-33
L1-10,11,12-A	CDRL1	2HMI	C	SASQDISSYLN	house mouse	IGKV10-94
L1-10,11,12-A	CDRL1	3HI6	L	RASQSIGSYLN	human	IGKV1-39
L1-10,11,12-A	CDRL1	3CDF	F	QASQDISNYLN	human	IGKV1-33
L1-10,11,12-A	CDRL1	1SEQ	L	SASSSVSYMH	house mouse	IGKV4-80
L1-10,11,12-A	CDRL1	1XF2	A	RASENIYSYLA	house mouse	IGKV12-44
L1-10,11,12-A	CDRL1	3SKJ	M	RASQSISTWLA	human	IGKV1-5
L1-10,11,12-A	CDRL1	1CT8	C	RASQSVSNKLH	house mouse	IGKV5-43
L1-10,11,12-A	CDRL1	1IVL	A	RASQSIGNRLF	house mouse	IGKV5-43
L1-10,11,12-A	CDRL1	1TJI	L	RASQGVTSALA	human	IGKV1D-13
L1-10,11,12-A	CDRL1	4HBC	L	QASQSISSYLA	rabbit	IGKV1S3
L1-10,11,12-A	CDRL1	2P8P	A	RASQGVTSALA	human	IGKV1D-13
L1-10,11,12-A	CDRL1	3CDY	B	QASQDINNYLI	human	IGKV1-33
L1-10,11,12-A	CDRL1	1DEE	C	RTSQSISSYLN	human	IGKV1-39

L1-10,11,12-A	CDRL1	2BX5	L	RASQSISSYLN	human	IGKV1-39
L1-10,11,12-A	CDRL1	1IVL	B	RASQSIGNRLF	house mouse	IGKV5-43
L1-10,11,12-A	CDRL1	4F33	G	SASSSVSYMH	house mouse	IGKV4-59
L1-10,11,12-A	CDRL1	2Q76	A	RASQSISNNLH	house mouse	IGKV5-43
L1-10,11,12-A	CDRL1	2DQG	L	RASQSIGNNLH	house mouse	IGKV5-43
L1-10,11,12-A	CDRL1	2IH3	B	RASQSIGTDIH	house mouse	IGKV5-48
L1-10,11,12-A	CDRL1	1MLC	C	RASQSISNNLH	house mouse	IGKV5-43
L1-10,11,12-A	CDRL1	1CT8	A	RASQSVSNKLH	house mouse	IGKV5-43
L1-10,11,12-A	CDRL1	3KYM	G	RASQSVSSYLA	human	IGKV3-15
L1-10,11,12-A	CDRL1	2E27	L	KANQDIKKKIA	house mouse	IGKV19-93
L1-10,11,12-A	CDRL1	1FE8	N	RASQDIGNYLN	house mouse	IGKV10-96
L1-10,11,12-A	CDRL1	1D5I	L	KASQDINSYLS	house mouse	IGKV14-111
L1-10,11,12-A	CDRL1	1VFA	A	RASGNIHNYLA	house mouse	IGKV12-41
L1-10,11,12-A	CDRL1	4JFX	L	MTSTDIDDDMN	human	IGKV3-11
L1-10,11,12-A	CDRL1	1BRE	A	QASQDISDYLI	human	IGKV1-33
L1-10,11,12-A	CDRL1	4DGI	L	RASQNIGTSIH	house mouse	IGKV5-48
L1-10,11,12-A	CDRL1	1NMC	L	RASQDISNYLN	house mouse	IGKV10-96
L1-10,11,12-A	CDRL1	4FNL	L	QASQDIRKFLN	human	IGKV1-33
L1-10,11,12-A	CDRL1	3NAC	L	RASQSIGLYLA	human	IGKV1-39
L1-10,11,12-A	CDRL1	1RZJ	L	RASESVSSDLA	human	IGKV3-15
L1-10,11,12-A	CDRL1	4LLW	B	KASQDVSIGVA	human	IGKV1-16
L1-10,11,12-A	CDRL1	3L7E	A	RASKSISKYLA	house mouse	IGKV16-104
L1-10,11,12-A	CDRL1	3EYO	C	RASQSVGGYLT	human	IGKV3-11
L1-10,11,12-A	CDRL1	3CFD	L	RASQDISNYLN	house mouse	IGKV10-96
L1-10,11,12-A	CDRL1	4HFW	A	RASQSISNYLN	human	IGKV1-6
L1-10,11,12-A	CDRL1	1B0W	C	QASQDISDYLI	human	IGKV1-33
L1-10,11,12-A	CDRL1	3T3P	F	HASQGISSNIG	house mouse	IGKV14-100
L1-10,11,12-A	CDRL1	4GW5	C	RASQSIGTNIH	house mouse	IGKV5-48
L1-10,11,12-A	CDRL1	1XGP	A	RASQSISNNLH	house mouse	IGKV5-43
L1-10,11,12-A	CDRL1	3L1O	L	RASKSIRKFLA	house mouse	IGKV16-104
L1-10,11,12-A	CDRL1	1SY6	L	SASSSVSYMN	house mouse	IGKV4-59
L1-10,11,12-A	CDRL1	2H9G	A	RASQDVSTAVA	human	IGKV1-39
L1-10,11,12-A	CDRL1	1LK3	L	KASESVTSRMH	Norway rat	IGKV3S11
L1-10,11,12-A	CDRL1	1YY8	A	RASQSIGTNIH	house mouse	IGKV5-48
L1-10,11,12-A	CDRL1	3B2U	D	RASQSVSSYLA	human	IGKV3D-11
L1-10,11,12-A	CDRL1	1P2C	A	RASQSISNNLH	house mouse	IGKV5-43
L1-10,11,12-A	CDRL1	4GW1	C	RASQSIGTNIH	house mouse	IGKV5-48
L1-10,11,12-A	CDRL1	2Q8B	L	KASQSVSNDVV	house mouse	IGKV6-32
L1-10,11,12-A	CDRL1	1FVE	C	RASQDVNTAVA	human	IGKV1-39
L1-10,11,12-A	CDRL1	3EFD	L	RASQSVSSAVA	human	IGKV1-39
L1-10,11,12-A	CDRL1	1I8M	L	RASENIYSYLA	house mouse	IGKV12-44
L1-10,11,12-A	CDRL1	4ERS	L	RASQGIRNDLG	human	IGKV1-17
L1-10,11,12-A	CDRL1	3U79	D	QASQDISNFLI	human	IGKV1-33
L1-10,11,12-A	CDRL1	3NIF	L	HASQGISSNIG	house mouse	IGKV14-100
L1-10,11,12-A	CDRL1	2NY5	L	RASESVSSDLA	human	IGKV3-15
L1-10,11,12-A	CDRL1	1BWW	A	QASQDIIKYLN	human	IGKV1-33
L1-10,11,12-A	CDRL1	1DVF	A	RASGNIHNYLA	house mouse	IGKV12-41
L1-10,11,12-A	CDRL1	4DKF	L	RASQSISSYLA	human	IGKV1-39
L1-10,11,12-A	CDRL1	2W0F	B	RASQSIGTDIH	house mouse	IGKV5-48
L1-10,11,12-A	CDRL1	3U79	A	QASQDISNFLI	human	IGKV1-33

L1-10,11,12-A	CDRL1	1RZ8	C	RASESVSSDLA	human	IGKV3-15
L1-10,11,12-A	CDRL1	1T3F	A	KASENVDTYVS	human	IGKV1-5
L1-10,11,12-A	CDRL1	3RVV	C	KASQDIYSYLS	house mouse	IGKV14-111
L1-10,11,12-A	CDRL1	2PW1	A	RASQGVTSALA	human	IGKV1D-13
L1-10,11,12-A	CDRL1	3BKY	L	RASSSVSYMH	house mouse	IGKV4-72
L1-10,11,12-A	CDRL1	1RZ7	L	RASQDISTWLA	human	IGKV1-12
L1-10,11,12-A	CDRL1	1K4C	B	RASQSIGTDIH	house mouse	IGKV5-48
L1-10,11,12-A	CDRL1	1CFT	A	KASQDINSFLT	house mouse	IGKV14-111
L1-10,11,12-A	CDRL1	3CMO	X	RASQDISNYLN	house mouse	IGKV10-94
L1-10,11,12-A	CDRL1	2FJF	W	RASQDVSTAVA	human	IGKV1-39
L1-10,11,12-A	CDRL1	1CE1	L	KASQNIDKYLN	human	IGKV1-33
L1-10,11,12-A	CDRL1	4EBQ	L	SASSSVSYMY	house mouse	IGKV4-55
L1-10,11,12-A	CDRL1	1RJL	A	HASQNINVWLN	house mouse	IGKV11-125
L1-10,11,12-A	CDRL1	3U9P	L	QASQIIYNYIA	Norway rat	IGKV19S2
L1-10,11,12-A	CDRL1	1G7L	A	RASGNIHNYLA	house mouse	IGKV12-41
L1-10,11,12-A	CDRL1	4KUC	D	RASKSVSYMH	house mouse	IGKV3-12
L1-10,11,12-A	CDRL1	1C5C	L	RTSQEISGYLS	house mouse	IGKV9-124
L1-10,11,12-A	CDRL1	1NGX	L	KASQNVGTAVA	house mouse	IGKV6-13
L1-10,11,12-A	CDRL1	4GW5	A	RASQSIGTNIH	house mouse	IGKV5-48
L1-10,11,12-A	CDRL1	3IDG	A	RASQGVTSALA	human	IGKV1-13
L1-10,11,12-A	CDRL1	4ALA	L	KASQNVRTSVA	house mouse	IGKV6-14
L1-10,11,12-A	CDRL1	4JG1	L	MTSTDIDDDMN	human	IGKV3-11
L1-10,11,12-A	CDRL1	2YMX	L	RTSENIDSYLA	house mouse	IGKV12-46
L1-10,11,12-A	CDRL1	4G7Y	L	RASQSVSSAVA	human	IGKV1-5
L1-10,11,12-A	CDRL1	4GMT	L	KASQNVDTNVA	house mouse	IGKV6-15
L1-10,11,12-A	CDRL1	1IGM	L	QASQDISNYLA	human	IGKV1-33
L1-10,11,12-A	CDRL1	2BX5	N	RASQSISSYLN	human	IGKV1-39
L1-10,11,12-A	CDRL1	1PSK	L	SASSSVSNIH	house mouse	IGKV4-57
L1-10,11,12-A	CDRL1	3HB3	D	RASENIYSYLA	house mouse	IGKV12-44
L1-10,11,12-A	CDRL1	1KCS	L	KASENVGTYVS	house mouse	IGKV6-20
L1-10,11,12-A	CDRL1	3L95	L	RASQDVSTAVA	human	IGKV1-39
L1-10,11,12-A	CDRL1	2DQE	L	RASQSIGNNLH	house mouse	IGKV5-43
L1-10,11,12-A	CDRL1	1E4W	L	RASQDISHYLN	house mouse	IGKV10-96
L1-10,11,12-A	CDRL1	1K4D	B	RASQSIGTDIH	house mouse	IGKV5-48
L1-10,11,12-A	CDRL1	2A6I	A	RASQDISNYLN	house mouse	IGKV10-96
L1-10,11,12-A	CDRL1	1UWX	L	LASQTIGTWLA	house mouse	IGKV12-98
L1-10,11,12-A	CDRL1	4CAD	J	RASGNIHNYLA	house mouse	IGKV12-41
L1-10,11,12-A	CDRL1	1FAI	L	RASQDISNYLN	house mouse	IGKV10-96
L1-10,11,12-A	CDRL1	3BN9	E	RASQGISSYLA	human	IGKV1-12
L1-10,11,12-A	CDRL1	2ATK	B	RASQSIGTDIH	house mouse	IGKV5-48
L1-10,11,12-A	CDRL1	2BX5	D	RASQSISSYLN	human	IGKV1-39
L1-10,11,12-A	CDRL1	3A6C	L	RASQSIGNNLH	house mouse	IGKV5-43
L1-10,11,12-A	CDRL1	1U8M	A	RASQGVTSALA	human	IGKV1D-13
L1-10,11,12-A	CDRL1	3MOD	L	RASQGVTSALA	human	IGKV1-13
L1-10,11,12-A	CDRL1	1NGY	A	KASQNVGTPVA	house mouse	IGKV6-13
L1-10,11,12-A	CDRL1	4JM4	L	RASQNINKNLA	human	IGKV3-15
L1-10,11,12-A	CDRL1	1ZWI	B	RASQSIGTDIH	house mouse	IGKV5-48
L1-10,11,12-A	CDRL1	3I75	A	QSSQDINKYIG	house mouse	IGKV19-93
L1-10,11,12-A	CDRL1	2JK5	B	RASQSIGTDIH	house mouse	IGKV5-48
L1-10,11,12-A	CDRL1	1XIW	C	RASQDIRNYLN	house mouse	IGKV10-96

L1-10,11,12-A	CDRL1	1REI	B	QASQDIIKYLN	human	IGKV1-33
L1-10,11,12-A	CDRL1	4G3Y	L	RASQFVGSSIH	house mouse	IGKV5-48
L1-10,11,12-A	CDRL1	2NXY	C	RASESVSSDLA	human	IGKV3-15
L1-10,11,12-A	CDRL1	1NDM	A	RASQSISNNLH	house mouse	IGKV5-43
L1-10,11,12-A	CDRL1	1DEE	A	RTSQSISSYLN	human	IGKV1-39
L1-10,11,12-A	CDRL1	2YBR	H	RASQSVRSYLA	human	IGKV3D-11
L1-10,11,12-A	CDRL1	4JO3	L	QASETISNYLA	rabbit	IGKV1S3
L1-10,11,12-A	CDRL1	4NRX	L	RASQSIGSYLN	human	IGKV1-39
L1-10,11,12-A	CDRL1	4LMQ	I	RASESISYSLS	human	IGKV1-5
L1-10,11,12-A	CDRL1	4JPI	B	RASQSVSSYLA	human	IGKV3-11
L1-10,11,12-A	CDRL1	1WCB	A	SATSGVNYMH	house mouse	IGKV4-57
L1-10,11,12-A	CDRL1	2BOB	B	RASQSIGTDIH	house mouse	IGKV5-48
L1-10,11,12-A	CDRL1	2FJG	L	RASQDVSTAVA	human	IGKV1-39
L1-10,11,12-A	CDRL1	1TJG	L	RASQGVTSALA	human	IGKV1D-13
L1-10,11,12-A	CDRL1	4G6M	L	RASQDISNYLS	house mouse	IGKV10-96
L1-10,11,12-A	CDRL1	2BX5	E	RASQSISSYLN	human	IGKV1-39
L1-10,11,12-A	CDRL1	1YYL	L	RASESVSSDLA	human	IGKV3-15
L1-10,11,12-A	CDRL1	1C12	A	HASQGISSNIG	house mouse	IGKV14-100
L1-10,11,12-A	CDRL1	3D85	A	RASQSISDYLH	house mouse	IGKV5-39
L1-10,11,12-A	CDRL1	3OJD	A	KASGNIHNYLA	house mouse	IGKV12-41
L1-10,11,12-A	CDRL1	3HC4	L	KASQNVGINVA	human	IGKV1-16
L1-10,11,12-A	CDRL1	1JRH	L	KASEDIYNRLA	house mouse	IGKV13-84
L1-10,11,12-A	CDRL1	1WEJ	L	RASGNIHNYLA	house mouse	IGKV12-41
L1-10,11,12-A	CDRL1	3D9A	L	RASQSIGNNLH	house mouse	IGKV5-43
L1-10,11,12-A	CDRL1	2HFG	L	RASQDVSTAVA	human	IGKV1-5
L1-10,11,12-A	CDRL1	4G7V	L	RASQSVSSAVA	human	IGKV1-5
L1-10,11,12-A	CDRL1	3OAY	L	RASQSIETWLA	human	IGKV1-5
L1-10,11,12-A	CDRL1	3V0V	B	RASQNINIWLS	house mouse	IGKV11-125
L1-10,11,12-A	CDRL1	1B0W	B	QASQDISDYLI	human	IGKV1-33
L1-10,11,12-A	CDRL1	1BFO	C	KASQNIDKYLN	Norway rat	IGKV22S7
L1-10,11,12-A	CDRL1	3DSF	L	RASENIYSFLA	house mouse	IGKV12-46
L1-10,11,12-A	CDRL1	1R3L	A	RASQSIGTDIH	house mouse	IGKV5-48
L1-10,11,12-A	CDRL1	1HEZ	C	RTSQSISSYLN	human	IGKV1-39
L1-10,11,12-A	CDRL1	3T3P	L	HASQGISSNIG	house mouse	IGKV14-100
L1-10,11,12-A	CDRL1	1KC5	L	KASENVDTYVS	house mouse	IGKV6-20
L1-10,11,12-A	CDRL1	3RA7	M	RASQDIKNYLN	house mouse	IGKV10-96
L1-10,11,12-A	CDRL1	2Z92	B	SASSSVSSVH	house mouse	IGKV4-59
L1-10,11,12-A	CDRL1	3L7F	D	RASKSISKYLA	human	IGKV3-11
L1-10,11,12-A	CDRL1	4LLY	B	KASQDVSIGVA	human	IGKV1D-13
L1-10,11,12-A	CDRL1	4HT1	L	QASQNIYSNLA	rabbit	IGKV1S3
L1-10,11,12-A	CDRL1	3P0Y	L	RASQDLATDVA	human	IGKV1-5
L1-10,11,12-A	CDRL1	3SOB	L	RASQDVSTAVA	human	IGKV1-39
L1-10,11,12-A	CDRL1	2YPV	L	KASQDIHNYLN	house mouse	IGKV14-111
L1-10,11,12-A	CDRL1	1BGX	L	SASSSVSYMY	house mouse	IGKV4-55
L1-10,11,12-A	CDRL1	2EIZ	A	RASQSIGNNLH	human	IGKV3-15
L1-10,11,12-A	CDRL1	1U8L	A	RASQGVTSALA	human	IGKV1D-13
L1-10,11,12-A	CDRL1	4JG0	L	MTSTDIDDDMN	human	IGKV3-11
L1-10,11,12-A	CDRL1	1A7Q	L	RAGGNTHNYLA	house mouse	IGKV12-41
L1-10,11,12-A	CDRL1	3STL	B	RASQSIGTDIH	house mouse	IGKV5-48
L1-10,11,12-A	CDRL1	3RKD	L	RASEIIYSNLA	house mouse	IGKV12-46

L1-10,11,12-A	CDRL1	1BOG	A	KASQDINSFLT	house mouse	IGKV14-111
L1-10,11,12-A	CDRL1	1BRE	C	QASQDISDYLI	human	IGKV1-33
L1-10,11,12-A	CDRL1	4LLU	B	KASQDVSIGVA	human	IGKV1-16
L1-10,11,12-A	CDRL1	2BX5	O	RASQSISSYLN	human	IGKV1-39
L1-10,11,12-A	CDRL1	4JR9	L	QASQNITNYIV	house mouse	IGKV19-93
L1-10,11,12-A	CDRL1	4NRX	E	RASQSIGSYLN	human	IGKV1-39
L1-10,11,12-A	CDRL1	1FVC	C	RASQDVNTAVA	human	IGKV1-39
L1-10,11,12-A	CDRL1	3QWO	B	SASSRVGYMH	human	IGKV1-5
L1-10,11,12-A	CDRL1	3KYM	I	RASQSVSSYLA	human	IGKV3-15
L1-10,11,12-A	CDRL1	1ZTX	L	KASQDVSTAVA	house mouse	IGKV6-25
L1-10,11,12-A	CDRL1	3CMO	L	RASQDISNYLN	house mouse	IGKV10-94
L1-10,11,12-A	CDRL1	2P8M	A	RASQGVTSALA	human	IGKV1D-13
L1-10,11,12-A	CDRL1	3KYM	E	RASQSVSSYLA	human	IGKV3-15
L1-10,11,12-A	CDRL1	1UM6	L	RASQEISGYLY	house mouse	IGKV9-124
L1-10,11,12-A	CDRL1	4JO4	L	QASETISNYLA	rabbit	IGKV1S3
L1-10,11,12-A	CDRL1	3HC0	L	KASQNVGINVA	human	IGKV1-16
L1-10,11,12-A	CDRL1	1V7M	M	SASSSVSYMY	house mouse	IGKV4-57
L1-10,11,12-A	CDRL1	1C5D	L	QASQDINKYIA	Norway rat	IGKV19S2
L1-10,11,12-A	CDRL1	2FAT	L	SASSSVSYMH	house mouse	IGKV4-86
L1-10,11,12-A	CDRL1	2HWZ	L	KCQLSVGYMH	house mouse	IGKV1-117
L1-10,11,12-A	CDRL1	1XF2	L	RASENIYSYLA	house mouse	IGKV12-44
L1-10,11,12-A	CDRL1	3UC0	L	RASQDISIRLN	human	IGKV1-13
L1-10,11,12-A	CDRL1	1NBZ	A	RASQSISNNLH	house mouse	IGKV5-43
L1-10,11,12-A	CDRL1	1A7P	L	RASGNIHNYLA	house mouse	IGKV12-41
L1-10,11,12-A	CDRL1	1JFQ	L	RASQDINNFLN	house mouse	IGKV10-96
L1-10,11,12-A	CDRL1	3HC0	B	KASQNVGINVA	human	IGKV1-16
L1-10,11,12-A	CDRL1	2ZKH	L	SASSSVSYMY	house mouse	IGKV4-57
L1-10,11,12-A	CDRL1	1U8J	A	RASQGVTSALA	human	IGKV1D-13
L1-10,11,12-A	CDRL1	3OZ9	L	SASSSVSYMH	house mouse	IGKV4-80
L1-10,11,12-A	CDRL1	2V7H	A	RASQDINNYLN	house mouse	IGKV10-96
L1-10,11,12-A	CDRL1	3NZH	L	KASQSVSNDVA	house mouse	IGKV6-32
L1-10,11,12-A	CDRL1	3R1G	L	RASQDVSTAVA	human	IGKV1-13
L1-10,11,12-A	CDRL1	1NDG	A	RASQSISNNLH	house mouse	IGKV5-43
L1-10,11,12-A	CDRL1	1U8N	A	RASQGVTSALA	human	IGKV1D-13
L1-10,11,12-A	CDRL1	2HG5	B	RASQSIGTDIH	house mouse	IGKV5-48
L1-10,11,12-A	CDRL1	3B2U	O	RASQSVSSYLA	human	IGKV3D-11
L1-10,11,12-A	CDRL1	2FR4	A	RASENIYSYLA	house mouse	IGKV12-44
L1-10,11,12-A	CDRL1	2Q1E	B	QASQDINNYLI	human	IGKV1-33
L1-10,11,12-A	CDRL1	2YC1	E	RASQSVRSYLA	human	IGKV3D-11
L1-10,11,12-A	CDRL1	3UPA	B	RASQSISSYLN	human	IGKV1-39
L1-10,11,12-A	CDRL1	1MLB	A	RASQSISNNLH	house mouse	IGKV5-43
L1-10,11,12-A	CDRL1	3U79	B	QASQDISNFLI	human	IGKV1-33
L1-10,11,12-A	CDRL1	1MHP	L	SASSSVNHMF	human	IGKV1D-13
L1-10,11,12-A	CDRL1	4J6R	L	RASQGVGSDVA	human	IGKV3D-7
L1-10,11,12-A	CDRL1	1A7R	L	RASGNIHNYLA	house mouse	IGKV12-41
L1-10,11,12-A	CDRL1	2D7T	L	RASQNINNYLH	human	IGKV1-6
L1-10,11,12-A	CDRL1	1GC1	L	RASESVSSDLA	human	IGKV3-15
L1-10,11,12-A	CDRL1	1BFO	G	KASQNIDKYLN	Norway rat	IGKV22S7
L1-10,11,12-A	CDRL1	2H8P	B	RASQSIGTDIH	house mouse	IGKV5-48
L1-10,11,12-A	CDRL1	4I2X	A	KASQDVSTAVA	human	IGKV6-17

L1-10,11,12-A	CDRL1	4LKC	A	RASQGISSALA	human	IGKV1-13
L1-10,11,12-A	CDRL1	3CXD	L	RASENIYSFLA	house mouse	IGKV12-46
L1-10,11,12-A	CDRL1	3PNW	P	RASQSVSSAVA	human	IGKV1-39
L1-10,11,12-A	CDRL1	2BX5	J	RASQSISSYLN	human	IGKV1-39
L1-10,11,12-A	CDRL1	2NY4	C	RASESVSSDLA	human	IGKV3-15
L1-10,11,12-A	CDRL1	2DQJ	L	RASQSIGNNLH	house mouse	IGKV5-43
L1-10,11,12-A	CDRL1	2HFE	B	RASQSIGTDIH	house mouse	IGKV5-48
L1-10,11,12-A	CDRL1	1RZG	D	RASQSISNWLA	human	IGKV1-5
L1-10,11,12-A	CDRL1	3B9K	C	QASQNIDKYIA	Norway rat	IGKV19S2
L1-10,11,12-A	CDRL1	4LMQ	L	RASESISYSLS	human	IGKV1-5
L1-10,11,12-A	CDRL1	1BRE	F	QASQDISDYLI	human	IGKV1-33
L1-10,11,12-A	CDRL1	2OQJ	G	RASQSIETWLA	human	IGKV1-5
L1-10,11,12-A	CDRL1	1P7K	L	RASENIYSYLA	house mouse	IGKV12-44
L1-10,11,12-A	CDRL1	2DWD	B	RASQSIGTDIH	house mouse	IGKV5-48
L1-10,11,12-A	CDRL1	2FJF	M	RASQDVSTAVA	human	IGKV1-39
L1-10,11,12-A	CDRL1	2NXZ	C	RASESVSSDLA	human	IGKV3-15
L1-10,11,12-A	CDRL1	1FGV	L	RASQDINNYLN	human	IGKV1-33
L1-10,11,12-A	CDRL1	1N8Z	A	RASQDVNTAVA	human	IGKV1-39
L1-10,11,12-A	CDRL1	1XF4	L	RASENIYSYLA	house mouse	IGKV12-44
L1-10,11,12-A	CDRL1	1UA6	L	RASQSIGNNLH	house mouse	IGKV5-43
L1-10,11,12-A	CDRL1	1KIQ	A	RASGNIHNYLA	house mouse	IGKV12-41
L1-10,11,12-A	CDRL1	4M43	L	RASQIIGTSIH	house mouse	IGKV5-48
L1-10,11,12-A	CDRL1	4JRE	C	QASQNITNYIV	house mouse	IGKV19-93
L1-10,11,12-A	CDRL1	3QWO	L	SASSRVGYMH	human	IGKV1-5
L1-10,11,12-A	CDRL1	1CFS	A	KASQDINSFLT	house mouse	IGKV14-111
L1-10,11,12-A	CDRL1	3DIF	A	KASQDVSTAVA	house mouse	IGKV6-17
L1-10,11,12-A	CDRL1	2VXU	L	RASQDIGSKLY	house mouse	IGKV9-120
L1-10,11,12-A	CDRL1	3RKD	C	RASEIIYSNLA	house mouse	IGKV12-46
L1-10,11,12-A	CDRL1	1EO8	L	SASSDISYMH	house mouse	IGKV4-70
L1-10,11,12-A	CDRL1	1NGZ	A	KASQNVGTAVA	house mouse	IGKV6-13
L1-10,11,12-A	CDRL1	3CDF	D	QASQDISNYLN	human	IGKV1-33
L1-10,11,12-A	CDRL1	2A6J	A	RASQDISNYLN	house mouse	IGKV10-96
L1-10,11,12-A	CDRL1	2NY1	C	RASESVSSDLA	human	IGKV3-15
L1-10,11,12-A	CDRL1	1DFB	L	RASQSISRWLA	human	IGKV1-5
L1-10,11,12-A	CDRL1	1WTL	B	RASQDITNYVN	human	IGKV1-33
L1-10,11,12-A	CDRL1	1BQL	L	SASSSVNYMY	house mouse	IGKV4-59
L1-10,11,12-A	CDRL1	3GK8	L	KASQDVNTALA	house mouse	IGKV6-17
L1-10,11,12-A	CDRL1	3FZU	L	RASQDIRYYLN	human	IGKV1-39
L1-10,11,12-A	CDRL1	4FNL	M	QASQDIRKFLN	human	IGKV1-33
L1-10,11,12-A	CDRL1	1BRE	E	QASQDISDYLI	human	IGKV1-33
L1-10,11,12-A	CDRL1	3L7F	A	RASKSISKYLA	human	IGKV3-11
L1-10,11,12-A	CDRL1	1XGU	A	RASQSISNNLH	house mouse	IGKV5-43
L1-10,11,12-A	CDRL1	3G5Z	A	HASQDIISNIG	house mouse	IGKV14-100
L1-10,11,12-A	CDRL1	3CDF	E	QASQDISNYLN	human	IGKV1-33
L1-10,11,12-A	CDRL1	1UM4	L	RASQEISGYLY	house mouse	IGKV9-124
L1-10,11,12-A	CDRL1	4D9Q	D	ITSTDIDDDMN	human	IGKV1-27
L1-10,11,12-A	CDRL1	3PGF	L	RASQSVSSAVA	human	IGKV1-39
L1-10,11,12-A	CDRL1	4J8R	A	KASQVGTALA	house mouse	IGKV6-23
L1-10,11,12-A	CDRL1	3B2U	X	RASQSVSSYLA	human	IGKV3D-11
L1-10,11,12-A	CDRL1	1QP1	A	QASQDISDYLI	human	IGKV1-33

L1-10,11,12-A	CDRL1	4J8R	C	KASQVGTALA	house mouse	IGKV6-23
L1-10,11,12-A	CDRL1	3S34	L	RASQGIDNWLG	human	IGKV1-12
L1-10,11,12-A	CDRL1	12E8	L	KASQNVGTAVA	house mouse	IGKV6-13
L1-10,11,12-A	CDRL1	1JPT	L	RASRDIKSYLN	human	IGKV1-39
L1-10,11,12-A	CDRL1	2UZI	L	RASQSISSYLN	human	IGKV1-39
L1-10,11,12-A	CDRL1	2XTJ	B	RASQGIRSALN	human	IGKV1-6
L1-10,11,12-A	CDRL1	3EO0	C	RASQSLGSSYLA	human	IGKV3-20
L1-10,11,12-A	CDRL1	1I8I	A	MTSTDIDDDMN	house mouse	IGKV17-127
L1-10,11,12-A	CDRL1	3CDC	B	QASQDISNYLI	human	IGKV1-33
L1-10,11,12-A	CDRL1	1FOR	L	SATSSVNYMH	house mouse	IGKV4-57
L1-10,11,12-A	CDRL1	3KYM	C	RASQSVSSYLA	human	IGKV3-15
L1-10,11,12-A	CDRL1	3NA9	L	RASQSIGLYLA	human	IGKV1-39
L1-10,11,12-A	CDRL1	4H20	L	KASQDVTSAVA	house mouse	IGKV6-17
L1-10,11,12-A	CDRL1	3GIZ	L	RASQSVSSYLA	human	IGKV3-11
L1-10,11,12-A	CDRL1	2FD6	L	SASSSVSYMH	house mouse	IGKV4-86
L1-10,11,12-A	CDRL1	2FJF	U	RASQDVSTAVA	human	IGKV1-39
L1-10,11,12-A	CDRL1	1OM3	L	RASQSIETWLA	human	IGKV1-5
L1-10,11,12-A	CDRL1	1KCU	L	KASENVDTYVS	house mouse	IGKV6-20
L1-10,11,12-A	CDRL1	1F6L	L	RASENIYSYLA	human	IGKV1-39
L1-10,11,12-A	CDRL1	2OQJ	D	RASQSIETWLA	human	IGKV1-5
L1-10,11,12-A	CDRL1	3FZU	D	RASQDIRYYLN	human	IGKV1-39
L1-10,11,12-A	CDRL1	2DQI	L	RASQSIGNNLH	house mouse	IGKV5-43
L1-10,11,12-A	CDRL1	3V6O	E	KATQNVRTAVT	house mouse	IGKV6-14
L1-10,11,12-A	CDRL1	4NRZ	B	RASQHIKKYLN	human	IGKV1-39
L1-10,11,12-A	CDRL1	1XGQ	A	RASQSISNNLH	house mouse	IGKV5-43
L1-10,11,12-A	CDRL1	1NBY	A	RASQSISNNLH	house mouse	IGKV5-43
L1-10,11,12-A	CDRL1	3OAU	L	RASQSIETWLA	human	IGKV1-5
L1-10,11,12-A	CDRL1	4HH9	A	RASQSVGGYLT	human	IGKV3-11
L1-10,11,12-A	CDRL1	3ZDX	L	HASQGISSNIG	house mouse	IGKV14-100
L1-10,11,12-A	CDRL1	4DN3	L	RASQSVSDAYLA	human	IGKV3-11
L1-11-A	CDRL1	4M5Y	L	GGNNIGTKVLH	human	IGLV3-21
L1-11-A	CDRL1	4JZN	P	GGNNIGSKSVH	human	IGLV3-21
L1-11-A	CDRL1	4FQQ	C	GGNNIGSKSVH	human	IGLV3-21
L1-11-A	CDRL1	4NPY	L	GGNNIGSKSVH	human	IGLV3-21
L1-11-A	CDRL1	4IMK	C	GGNNIGSKSVH	human	IGLV3-21
L1-11-A	CDRL1	4JZO	F	SGDKLGDKYAS	human	IGLV3-1
L1-11-A	CDRL1	1W72	M	GGNNIGSRSVH	human	IGLV3-21
L1-11-A	CDRL1	3ULS	L	SGDNIGSYYVH	human	IGLV3-21
L1-11-A	CDRL1	3NH7	N	SGDSLGSKYVI	human	IGLV3-1
L1-11-A	CDRL1	4AJ0	C	SGDKLGDKYAY	human	IGLV3-1
L1-11-A	CDRL1	4HK0	D	GGNNIGSKSVH	human	IGLV3-21
L1-11-A	CDRL1	4AJ0	B	SGDKLGDKYAY	human	IGLV3-1
L1-11-A	CDRL1	2DD8	L	GGNNIGSKSVH	human	IGLV3-21
L1-11-A	CDRL1	4JZN	B	GGNNIGSKSVH	human	IGLV3-21
L1-11-A	CDRL1	4AJ0	D	SGDKLGDKYAY	human	IGLV3-1
L1-11-A	CDRL1	4AIX	D	SGDKLGDKYAY	human	IGLV3-1
L1-11-A	CDRL1	1W72	L	GGNNIGSRSVH	human	IGLV3-21
L1-11-A	CDRL1	4JZO	B	SGDKLGDKYAS	human	IGLV3-1
L1-11-A	CDRL1	3NH7	L	SGDSLGSKYVI	human	IGLV3-1
L1-11-A	CDRL1	3G6A	A	SGDNIGGTFVS	human	IGLV3-1

L1-11-A	CDRL1	4HK0	B	GGNNIGSKSVH	human	IGLV3-21
L1-11-A	CDRL1	4M5Y	M	GGNNIGTKVLH	human	IGLV3-21
L1-11-A	CDRL1	4FQQ	L	GGNNIGSKSVH	human	IGLV3-21
L1-11-A	CDRL1	4AIX	A	SGDKLGDKYAY	human	IGLV3-1
L1-11-A	CDRL1	4IMK	D	GGNNIGSKSVH	human	IGLV3-21
L1-11-A	CDRL1	4FQQ	A	GGNNIGSKSVH	human	IGLV3-21
L1-11-A	CDRL1	4NPY	A	GGNNIGSKSVH	human	IGLV3-21
L1-11-A	CDRL1	4AIX	C	SGDKLGDKYAY	human	IGLV3-1
L1-11-A	CDRL1	4AJ0	A	SGDKLGDKYAY	human	IGLV3-1
L1-11-A	CDRL1	3NH7	O	SGDSLGSKYVI	human	IGLV3-1
L1-11-A	CDRL1	3Q6G	M	GGDNIGSKSVQ	human	IGLV3-21
L1-11-A	CDRL1	3NH7	M	SGDSLGSKYVI	human	IGLV3-1
L1-11-A	CDRL1	3G6A	L	SGDNIGGTFVS	human	IGLV3-1
L1-11-A	CDRL1	4FQQ	E	GGNNIGSKSVH	human	IGLV3-21
L1-11-A	CDRL1	4JZO	H	SGDKLGDKYAS	human	IGLV3-1
L1-11-A	CDRL1	4JZO	E	SGDKLGDKYAS	human	IGLV3-1
L1-11-A	CDRL1	4AIX	B	SGDKLGDKYAY	human	IGLV3-1
L1-11-A	CDRL1	3Q6G	L	GGDNIGSKSVQ	human	IGLV3-21
L1-11-B	CDRL1	1NFD	G	SGDQLPKNFAY	hamster	IGLV
L1-11-B	CDRL1	3MLU	L	SGDKLDDKYVS	human	IGLV3-1
L1-11-B	CDRL1	3MLR	L	SGDKLDDKYVS	human	IGLV3-1
L1-11-B	CDRL1	3MLS	L	SGDKLDDKYVS	human	IGLV3-1
L1-11-B	CDRL1	3MLS	N	SGDKLDDKYVS	human	IGLV3-1
L1-11-B	CDRL1	3UJI	L	SGDILGDKYVS	human	IGLV3-1
L1-11-B	CDRL1	1NFD	E	SGDQLPKNFAY	hamster	IGLV
L1-11-B	CDRL1	3MLV	L	SGDKLDDKYVS	human	IGLV3-1
L1-11-B	CDRL1	3MLT	A	SGDKLDDKYVS	human	IGLV3-1
L1-11-B	CDRL1	3UJJ	L	SGDALPEKYAY	human	IGLV3-10
L1-11-B	CDRL1	3MLS	M	SGDKLDDKYVS	human	IGLV3-1
L1-11-B	CDRL1	3MLV	M	SGDKLDDKYVS	human	IGLV3-1
L1-11-B	CDRL1	3MLT	G	SGDKLDDKYVS	human	IGLV3-1
L1-11-B	CDRL1	4AIZ	D	SGDALPKQYAY	human	IGLV3-25
L1-11-B	CDRL1	8FAB	C	SANALPNQYAY	human	IGLV3-25
L1-11-B	CDRL1	4AIZ	C	SGDALPKQYAY	human	IGLV3-25
L1-11-B	CDRL1	4AIZ	A	SGDALPKQYAY	human	IGLV3-25
L1-11-B	CDRL1	4AIZ	B	SGDALPKQYAY	human	IGLV3-25
L1-11-B	CDRL1	3MLT	D	SGDKLDDKYVS	human	IGLV3-1
L1-11-B	CDRL1	4HPY	L	SGDALPKNYAY	human	IGLV3-10
L1-11-B	CDRL1	8FAB	A	SANALPNQYAY	human	IGLV3-25
L1-11-B	CDRL1	3MLS	O	SGDKLDDKYVS	human	IGLV3-1
L1-11-B	CDRL1	3GO1	L	SAEALSNQYAY	human	IGLV3-25
L1-11-B	CDRL1	3MLT	L	SGDKLDDKYVS	human	IGLV3-1
L1-11-C	CDRL1	4FQC	L	GEKSLGSRAVQ	human	IGLV3-21
L1-11-C	CDRL1	4JY5	L	GEESLGSRSVI	human	IGLV3-21
L1-11-C	CDRL1	4JY4	A	GEKSLGSRAVQ	human	IGLV3-21
L1-11-C	CDRL1	4JY6	C	GKESIGSRAVQ	human	IGLV3-21
L1-11-C	CDRL1	4JY6	A	GKESIGSRAVQ	human	IGLV3-21
L1-12-A	CDRL1	3W9E	B	RASQSVTSSQLA	house mouse	IGKV1-117
L1-12-A	CDRL1	1IQD	A	RASQSFSSSYLA	human	IGKV3-20
L1-12-A	CDRL1	2BRR	L	RASSSVSSSYLH	house mouse	IGKV4-57-1

L1-12-A	CDRL1	3SO3	B	RASQSVSSSYLA	human	IGKV3-11
L1-12-A	CDRL1	4DVB	L	RASSTVSFHYLH	house mouse	IGKV4-57-1
L1-12-A	CDRL1	4HWE	L	RASQSISSSYLA	human	IGKV3-20
L1-12-A	CDRL1	1HQ4	A	SASSSVSSNYVY	house mouse	IGKV4-79
L1-12-A	CDRL1	4LAS	L	SASSSVSSSHLY	house mouse	IGKV4-79
L1-12-A	CDRL1	1ORS	A	TASSSVSSSYLH	house mouse	IGKV4-74
L1-12-A	CDRL1	25C8	L	TASSSVSSSNLH	house mouse	IGKV4-74
L1-12-A	CDRL1	3LS4	L	SASSSISSNYLH	house mouse	IGKV4-91
L1-12-A	CDRL1	2BRR	X	RASSSVSSSYLH	house mouse	IGKV4-57-1
L1-12-A	CDRL1	1CF8	L	SASSSVSSNYVY	house mouse	IGKV4-79
L1-12-A	CDRL1	3QEG	L	RASQSVSSSYLA	human	IGKV3-20
L1-12-A	CDRL1	35C8	L	TASSSVSSSNLH	house mouse	IGKV4-74
L1-12-A	CDRL1	3W9D	B	RASQSVTSSQLA	house mouse	IGKV1-117
L1-12-A	CDRL1	3EOT	L	SASSSVNSSALF	house mouse	IGKV4-68
L1-12-A	CDRL1	15C8	L	TASSSVSSSNLH	house mouse	IGKV4-74
L1-12-A	CDRL1	4DVB	B	RASSTVSFHYLH	house mouse	IGKV4-57-1
L1-12-A	CDRL1	1HQ4	C	SASSSVSSNYVY	house mouse	IGKV4-79
L1-12-A	CDRL1	4LAR	L	SASSSVSSSHLY	house mouse	IGKV4-79
L1-12-A	CDRL1	3LS5	L	SASSSISSNYLH	house mouse	IGKV4-91
L1-12-B	CDRL1	2V7N	G	RASQSVSSNYLA	human	IGKV3-11
L1-12-B	CDRL1	3LHP	L	RASQSVGNNKLA	human	IGKV3-20
L1-12-B	CDRL1	3LH2	N	RASQSVGNNKLA	human	IGKV3-20
L1-12-B	CDRL1	3LH2	O	RASQSVGNNKLA	human	IGKV3-20
L1-12-B	CDRL1	2FX8	N	RASQSVGNNKLA	human	IGKV3-20
L1-12-B	CDRL1	2FX8	M	RASQSVGNNKLA	human	IGKV3-20
L1-12-B	CDRL1	3LHP	M	RASQSVGNNKLA	human	IGKV3-20
L1-12-B	CDRL1	2FX7	L	RASQSVGNNKLA	human	IGKV3-20
L1-12-B	CDRL1	2V7N	C	RASQSVSSNYLA	human	IGKV3-11
L1-12-B	CDRL1	1RHH	A	RASQSVSSGSLA	human	IGKV3-20
L1-12-B	CDRL1	1DN0	C	GASQSVSSNYLA	human	IGKV3D-20
L1-12-B	CDRL1	2V7N	E	RASQSVSSNYLA	human	IGKV3-11
L1-12-B	CDRL1	2FX9	M	RASQSVGNNKLA	human	IGKV3-20
L1-12-B	CDRL1	4FQL	L	RASQSVSSSYLA	human	IGKV3-20
L1-12-B	CDRL1	2FX9	L	RASQSVGNNKLA	human	IGKV3-20
L1-12-B	CDRL1	4LLV	B	RASQSVGNNKLA	human	IGKV3-20
L1-12-B	CDRL1	3LH2	M	RASQSVGNNKLA	human	IGKV3-20
L1-12-B	CDRL1	1TZG	L	RASQSVGNNKLA	human	IGKV3-20
L1-12-B	CDRL1	1TZG	M	RASQSVGNNKLA	human	IGKV3-20
L1-12-B	CDRL1	1RHH	C	RASQSVSSGSLA	human	IGKV3-20
L1-12-B	CDRL1	2FX8	O	RASQSVGNNKLA	human	IGKV3-20
L1-12-B	CDRL1	1DN0	A	GASQSVSSNYLA	human	IGKV3D-20
L1-12-B	CDRL1	3LH2	L	RASQSVGNNKLA	human	IGKV3-20
L1-12-B	CDRL1	4LLV	L	RASQSVGNNKLA	human	IGKV3-20
L1-12-B	CDRL1	3H3P	M	RASQSVGNNKLA	human	IGKV3-20
L1-12-B	CDRL1	2FX8	L	RASQSVGNNKLA	human	IGKV3-20
L1-12-B	CDRL1	2V7N	A	RASQSVSSNYLA	human	IGKV3-11
L1-12-B	CDRL1	3H3P	L	RASQSVGNNKLA	human	IGKV3-20
L1-12-C	CDRL1	2OTU	C	TLSSQHSTYTIE	house mouse	IGLV3
L1-12-C	CDRL1	3S96	D	TLNSQHSTYTIE	house mouse	IGLV3
L1-12-C	CDRL1	2GSG	A	TLSSQHSTYTIE	house mouse	IGLV3

L1-12-C	CDRL1	3FFD	B	TLSSQHSTYTIE	house mouse	IGLV3
L1-12-C	CDRL1	2OTU	E	TLSSQHSTYTIE	house mouse	IGLV3
L1-12-C	CDRL1	3S96	B	TLNSQHSTYTIE	house mouse	IGLV3
L1-12-C	CDRL1	4JJ5	A	TLSRQHSTYTIE	house mouse	IGLV3
L1-12-C	CDRL1	2OTW	A	TLSSQHSTYTIE	house mouse	IGLV3
L1-12-C	CDRL1	4ISV	A	TLSRQHSTYTIE	house mouse	IGLV3
L1-12-C	CDRL1	2OTU	A	TLSSQHSTYTIE	house mouse	IGLV3
L1-12-C	CDRL1	2QHR	L	TLSRQHSTYTIE	house mouse	IGLV3
L1-12-C	CDRL1	4DCQ	A	TLNSQHSTYTIE	house mouse	IGLV3
L1-12-C	CDRL1	2OTU	G	TLSSQHSTYTIE	house mouse	IGLV3
L1-12-D	CDRL1	1N0X	L	RSSHSIRSRRVA	human	IGKV3-20
L1-12-D	CDRL1	3W9D	D	RASQSVTSSQLA	human	IGKV3
L1-12-D	CDRL1	2NY7	L	RSSHSIRSRRVA	human	IGKV3-20
L1-12-D	CDRL1	1HZH	M	RSSHSIRSRRVA	human	IGKV3-20
L1-12-D	CDRL1	4DN4	L	RASQSVSDAYLA	human	IGKV3-11
L1-12-D	CDRL1	1N0X	M	RSSHSIRSRRVA	human	IGKV3-20
L1-12-D	CDRL1	3RU8	L	RSSHSIRSRRVA	human	IGKV3-20
L1-13,14-A	CDRL1	2XZC	L	SGSSSNIGNNYVS	human	IGLV1-51
L1-13,14-A	CDRL1	2YK1	L	SGSSSNIGSNYVY	human	IGLV1-47
L1-13,14-A	CDRL1	1NL0	L	SGSTSNIGNNYVS	human	IGLV1-51
L1-13,14-A	CDRL1	2B1A	L	SGTSSNVENNYVY	human	IGLV1-47
L1-13,14-A	CDRL1	3TNN	B	TGTSSDVGSYNFVS	human	IGLV2-23
L1-13,14-A	CDRL1	3TNM	B	TGTSSDVGGYNYVS	human	IGLV2-8
L1-13,14-A	CDRL1	1MCI	A	TGTSSDVGGYNYVS	human	IGLV2-8
L1-13,14-A	CDRL1	4F57	L	TGTSSDVGGYNYVS	human	IGLV2-14
L1-13,14-A	CDRL1	4BJL	B	SGSSSNIGENSVT	human	IGLV1-44
L1-13,14-A	CDRL1	3MLX	M	SGSSSNIGNNMVS	human	IGLV1-51
L1-13,14-A	CDRL1	4DQO	L	QGTSSDVGGFDSVS	human	IGLV2-14
L1-13,14-A	CDRL1	2XWT	B	SGSSSDIGSNYVS	human	IGLV1-51
L1-13,14-A	CDRL1	2B1H	L	SGTSSNVENNYVY	human	IGLV1-47
L1-13,14-A	CDRL1	4FQI	L	SGSDSNIGRRSVN	human	IGLV1-44
L1-13,14-A	CDRL1	3QHZ	L	SGSSSNIGSNTVS	human	IGLV1-44
L1-13,14-A	CDRL1	3LRH	M	SGSNSNIGSNTVN	human	IGLV1-36
L1-13,14-A	CDRL1	3LRH	C	SGSNSNIGSNTVN	human	IGLV1-36
L1-13,14-A	CDRL1	3LRS	D	NGTSSDVGGFDSVS	human	IGLV2-14
L1-13,14-A	CDRL1	3GJE	L	TGTSRDVGGYNYVS	human	IGLV2-11
L1-13,14-A	CDRL1	3GBN	L	SGSSSNIGNDYVS	human	IGLV1-51
L1-13,14-A	CDRL1	4GXV	L	SGSSSNIGSYTVN	human	IGLV1-44
L1-13,14-A	CDRL1	4NZT	L	SGSDSNIGRRSVN	human	IGLV1-44
L1-13,14-A	CDRL1	4M1D	M	SGSSSNIGNNYVL	human	IGLV1-51
L1-13,14-A	CDRL1	2B0S	L	SGTSSNVENNYVY	human	IGLV1-47
L1-13,14-A	CDRL1	3T0X	B	SGSSSNVEDNSVY	human	IGLV1-47
L1-13,14-A	CDRL1	3GJF	L	TGTSRDVGGYNYVS	human	IGLV2-11
L1-13,14-A	CDRL1	4F58	N	TGTTSDVGTYNFVS	human	IGLV2-14
L1-13,14-A	CDRL1	3C2A	L	SGSSSNIGNNYVL	human	IGLV1-51
L1-13,14-A	CDRL1	4D9L	M	TGSSSNIGAHYDVH	human	IGLV1-40
L1-13,14-A	CDRL1	2YKL	L	SGSSSNIGSNYVY	human	IGLV1-47
L1-13,14-A	CDRL1	3TNN	D	TGTSSDVGSYNFVS	human	IGLV2-23
L1-13,14-A	CDRL1	3H42	L	TGSSSNIGAGYDVH	human	IGLV1-40
L1-13,14-A	CDRL1	3MUG	G	NGTSSDVGGFDSVS	human	IGLV2-14

L1-13,14-A	CDRL1	3QHF	L	SGSSSNIGSNTVS	human	IGLV1-44
L1-13,14-A	CDRL1	3MUG	E	NGTSSDVGGFDSVS	human	IGLV2-14
L1-13,14-A	CDRL1	3N9G	L	SGSSSNIGSNTVN	human	IGLV1-44
L1-13,14-A	CDRL1	1MCD	A	TGTSSDVGGYNYVS	human	IGLV2-8
L1-13,14-A	CDRL1	3LRH	A	SGSNSNIGSNTVN	human	IGLV1-36
L1-13,14-A	CDRL1	3G04	A	SGNSSNIGNNAVN	human	IGLV1-36
L1-13,14-A	CDRL1	3T0W	A	SGSSSNVEDNSVY	human	IGLV1-47
L1-13,14-A	CDRL1	4D9L	L	TGSSSNIGAHYDVH	human	IGLV1-40
L1-13,14-A	CDRL1	4FQH	B	SGSDSNIGRRSVN	human	IGLV1-44
L1-13,14-A	CDRL1	3BJL	A	SGSSSNIGENSVT	human	IGLV1-44
L1-13,14-A	CDRL1	2FB4	L	SGTSSNIGSSTVN	human	IGLV1-44
L1-13,14-A	CDRL1	3MUG	I	NGTSSDVGGFDSVS	human	IGLV2-14
L1-13,14-A	CDRL1	3T0V	A	SGSSSNVEDNSVY	human	IGLV1-47
L1-13,14-A	CDRL1	3U2S	L	QGTSNDVGGYESVS	human	IGLV2-14
L1-13,14-A	CDRL1	1RZF	L	SGSSSNIGNNDVS	human	IGLV1-51
L1-13,14-A	CDRL1	4F58	O	TGTTSDVGTYNFVS	human	IGLV2-14
L1-13,14-A	CDRL1	3GHB	M	SGSSSNIGNNYVL	human	IGLV1-51
L1-13,14-A	CDRL1	4FQJ	L	SGSSSNIGTNYVY	human	IGLV1-47
L1-13,14-A	CDRL1	3GHB	L	SGSSSNIGNNYVL	human	IGLV1-51
L1-13,14-A	CDRL1	3GJE	A	TGTSRDVGGYNYVS	human	IGLV2-11
L1-13,14-A	CDRL1	3TNN	L	TGTSSDVGSYNFVS	human	IGLV2-23
L1-13,14-A	CDRL1	3LMJ	L	SGSSSNIGKNYVS	human	IGLV1-51
L1-13,14-A	CDRL1	3BJL	B	SGSSSNIGENSVT	human	IGLV1-44
L1-13,14-A	CDRL1	1AQK	L	TGSNSNIGAGFTVH	human	IGLV1-40
L1-13,14-A	CDRL1	1MCC	A	TGTSSDVGGYNYVS	human	IGLV2-8
L1-13,14-A	CDRL1	1MCH	A	TGTSSDVGGYNYVS	human	IGLV2-8
L1-13,14-A	CDRL1	3MUG	A	NGTSSDVGGFDSVS	human	IGLV2-14
L1-13,14-A	CDRL1	3U2S	B	QGTSNDVGGYESVS	human	IGLV2-14
L1-13,14-A	CDRL1	7FAB	L	TGSSSNIGAGHNVK	human	IGLV1-40
L1-13,14-A	CDRL1	4K3E	M	SGSSSNVGNGYVS	sheep	IGLV1S8
L1-13,14-A	CDRL1	3TNN	F	TGTSSDVGSYNFVS	human	IGLV2-23
L1-13,14-A	CDRL1	3LRH	O	SGSNSNIGSNTVN	human	IGLV1-36
L1-13,14-A	CDRL1	3GJF	K	TGTSRDVGGYNYVS	human	IGLV2-11
L1-13,14-A	CDRL1	1BJM	A	SGSSSNIGENSVT	human	IGLV1-44
L1-13,14-A	CDRL1	2JB5	L	TGTSSDVGSNNYVS	human	IGLV2-23
L1-13,14-A	CDRL1	3GBM	M	SGSSSNIGNDYVS	human	IGLV1-51
L1-13,14-A	CDRL1	1Q1J	L	SGSSSNIGNNYVL	human	IGLV1-51
L1-13,14-A	CDRL1	2OLD	B	TGTSSDVGGYDLVS	human	IGLV2-14
L1-13,14-A	CDRL1	3LRG	A	SGSNSNIGSNTVN	human	IGLV1-36
L1-13,14-A	CDRL1	3MLY	L	SGSSSNIGNNMVS	human	IGLV1-51
L1-13,14-A	CDRL1	1MCB	A	TGTSSDVGGYNYVS	human	IGLV2-8
L1-13,14-A	CDRL1	3LRH	G	SGSNSNIGSNTVN	human	IGLV1-36
L1-13,14-A	CDRL1	1A8J	L	TGTSSDVGGYNYVS	human	IGLV2-8
L1-13,14-A	CDRL1	1MCS	A	TGTSSDVGGYNYVS	human	IGLV2-8
L1-13,14-A	CDRL1	4M1D	L	SGSSSNIGNNYVL	human	IGLV1-51
L1-13,14-A	CDRL1	1BJM	B	SGSSSNIGENSVT	human	IGLV1-44
L1-13,14-A	CDRL1	3NPS	C	SGSSSNIGSNYVS	human	IGLV1-44
L1-13,14-A	CDRL1	3MLW	M	SGNSSNIENNYVY	human	IGLV1-47
L1-13,14-A	CDRL1	3MLY	M	SGSSSNIGNNMVS	human	IGLV1-51
L1-13,14-A	CDRL1	1MCJ	A	TGTSSDVGGYNYVS	human	IGLV2-8

L1-13,14-A	CDRL1	3LRH	E	SGSNSNIGSNTVN	human	IGLV1-36
L1-13,14-A	CDRL1	4K3D	L	SGSSSNVGNGYVS	sheep	IGLV1S8
L1-13,14-A	CDRL1	2OLD	A	TGTSSDVGGYDLVS	human	IGLV2-14
L1-13,14-A	CDRL1	3T0W	B	SGSSSNVEDNSVY	human	IGLV1-47
L1-13,14-A	CDRL1	1MCK	A	TGTSSDVGGYNYVS	human	IGLV2-8
L1-13,14-A	CDRL1	3ZL4	L	SGSSSNIGNNYVS	human	IGLV1-51
L1-13,14-A	CDRL1	4K3E	L	SGSSSNVGNGYVS	sheep	IGLV1S8
L1-13,14-A	CDRL1	3LRG	B	SGSNSNIGSNTVN	human	IGLV1-36
L1-13,14-A	CDRL1	3LRH	K	SGSNSNIGSNTVN	human	IGLV1-36
L1-13,14-A	CDRL1	4GXV	M	SGSSSNIGSYTVN	human	IGLV1-44
L1-13,14-A	CDRL1	2OMN	A	TGTSSDVGGYDLVS	human	IGLV2-14
L1-13,14-A	CDRL1	3T0X	A	SGSSSNVEDNSVY	human	IGLV1-47
L1-13,14-A	CDRL1	4D9L	O	TGSSSNIGAHYDVH	human	IGLV1-40
L1-13,14-A	CDRL1	3MUG	K	NGTSSDVGGFDSVS	human	IGLV2-14
L1-13,14-A	CDRL1	1MCN	A	TGTSSDVGGYNYVS	human	IGLV2-8
L1-13,14-A	CDRL1	3LRH	I	SGSNSNIGSNTVN	human	IGLV1-36
L1-13,14-A	CDRL1	2XZA	L	SGSSSNIGNNYVS	human	IGLV1-51
L1-13,14-A	CDRL1	3QHZ	M	SGSSSNIGSNTVS	human	IGLV1-44
L1-13,14-A	CDRL1	3GBM	L	SGSSSNIGNDYVS	human	IGLV1-51
L1-13,14-A	CDRL1	3MLW	L	SGNSSNIENNYVY	human	IGLV1-47
L1-13,14-A	CDRL1	2OMN	B	TGTSSDVGGYDLVS	human	IGLV2-14
L1-13,14-A	CDRL1	4F58	L	TGTTSDVGTYNFVS	human	IGLV2-14
L1-13,14-A	CDRL1	4K3D	M	SGSSSNVGNGYVS	sheep	IGLV1S8
L1-13,14-A	CDRL1	1DCL	B	TGTSSNVGGYNYVS	human	IGLV2-8
L1-13,14-A	CDRL1	4D9L	N	TGSSSNIGAHYDVH	human	IGLV1-40
L1-13,14-A	CDRL1	3U4E	B	QGTSNDVGGYESVS	human	IGLV2-14
L1-13,14-A	CDRL1	3LRS	B	NGTSSDVGGFDSVS	human	IGLV2-14
L1-13,14-A	CDRL1	4FQH	L	SGSDSNIGRRSVN	human	IGLV1-44
L1-13,14-A	CDRL1	4F58	M	TGTTSDVGTYNFVS	human	IGLV2-14
L1-13,14-A	CDRL1	3U4E	L	QGTSNDVGGYESVS	human	IGLV2-14
L1-13,14-A	CDRL1	1Q1J	M	SGSSSNIGNNYVL	human	IGLV1-51
L1-13,14-A	CDRL1	3MLX	L	SGSSSNIGNNMVS	human	IGLV1-51
L1-13,14-A	CDRL1	3LRS	F	NGTSSDVGGFDSVS	human	IGLV2-14
L1-13,14-A	CDRL1	1MCF	A	TGTSSDVGGYNYVS	human	IGLV2-8
L1-13-A	CDRL1	1CD0	B	TRSSGNIDSNYVQ	human	IGLV6-57
L1-13-A	CDRL1	2W0L	D	TRSSGSIASNYVQ	human	IGLV6-57
L1-13-A	CDRL1	1CD0	A	TRSSGNIDSNYVQ	human	IGLV6-57
L1-13-A	CDRL1	2VXS	M	TRSSGSLANYYVQ	human	IGLV6-57
L1-13-A	CDRL1	2W0L	A	TRSSGSIASNYVQ	human	IGLV6-57
L1-13-A	CDRL1	2CD0	B	TRSSGSIANNYVH	human	IGLV6-57
L1-13-A	CDRL1	3B5G	A	TRSSGSIASNYVQ	human	IGLV6-57
L1-13-A	CDRL1	2W0L	C	TRSSGSIASNYVQ	human	IGLV6-57
L1-13-A	CDRL1	3H0T	A	TRSSGSIASYYVQ	human	IGLV6-57
L1-13-A	CDRL1	2VXS	O	TRSSGSLANYYVQ	human	IGLV6-57
L1-13-A	CDRL1	2CD0	A	TRSSGSIANNYVH	human	IGLV6-57
L1-13-A	CDRL1	2VXS	N	TRSSGSLANYYVQ	human	IGLV6-57
L1-13-A	CDRL1	2W0K	A	TRSSGSIASNYVQ	human	IGLV6-57
L1-13-A	CDRL1	1PW3	A	TRSSGNIDSNYVQ	human	IGLV6-57
L1-13-A	CDRL1	2VXS	L	TRSSGSLANYYVQ	human	IGLV6-57
L1-13-A	CDRL1	3B5G	B	TRSSGSIASNYVQ	human	IGLV6-57

L1-13-A	CDRL1	2W0L	B	TRSSGSIASNYVQ	human	IGLV6-57
L1-13-A	CDRL1	3BDX	C	TRSSGSIASNYVQ	human	IGLV6-57
L1-13-A	CDRL1	1PW3	B	TRSSGNIDSNYVQ	human	IGLV6-57
L1-13-A	CDRL1	4HPO	L	TRSSGSVASDYVQ	human	IGLV6-57
L1-13-A	CDRL1	3BDX	B	TRSSGSIASNYVQ	human	IGLV6-57
L1-13-A	CDRL1	3BDX	A	TRSSGSIASNYVQ	human	IGLV6-57
L1-13-A	CDRL1	2W0K	B	TRSSGSIASNYVQ	human	IGLV6-57
L1-14-A	CDRL1	4K3H	E	GSSTGAVTSGHYAN	human	IGLV7-46
L1-14-A	CDRL1	1NC4	C	RSSTGAVTTSNYAN	house mouse	IGLV1
L1-14-A	CDRL1	1GIG	L	RSSTGAVTTSNYAN	house mouse	IGLV1
L1-14-A	CDRL1	4K3G	B	GSSTGAVTSGHYAN	human	IGLV7-46
L1-14-A	CDRL1	4K3H	C	GSSTGAVTSGHYAN	human	IGLV7-46
L1-14-A	CDRL1	3CFJ	A	RSSSGAVTTSNYAT	house mouse	IGLV1
L1-14-A	CDRL1	1A6V	M	RSSTGAVTTSNYAN	house mouse	IGLV1
L1-14-A	CDRL1	1OAX	N	RSSTGAVTTSNYAN	house mouse	IGLV1
L1-14-A	CDRL1	1OAU	N	RSSTGAVTTSNYAN	house mouse	IGLV1
L1-14-A	CDRL1	4AT6	D	RSSTGAVTTSNYAN	house mouse	IGLV1
L1-14-A	CDRL1	1OAX	M	RSSTGAVTTSNYAN	house mouse	IGLV1
L1-14-A	CDRL1	1Y18	E	RSSSGAVTTSNYAT	house mouse	IGLV1
L1-14-A	CDRL1	1OCW	L	RSSTGAVTTSNYAN	house mouse	IGLV1
L1-14-A	CDRL1	1Y0L	L	RSSSGAVTTSNYAT	house mouse	IGLV1
L1-14-A	CDRL1	1OAY	N	RSSTGAVTTSNYAN	house mouse	IGLV1
L1-14-A	CDRL1	1A6W	L	RSSTGAVTTSNYAN	house mouse	IGLV1
L1-14-A	CDRL1	3CFK	A	RSSSGAVTTSNYAT	house mouse	IGLV1
L1-14-A	CDRL1	4K3H	A	GSSTGAVTSGHYAN	human	IGLV7-46
L1-14-A	CDRL1	4AT6	N	RSSTGAVTTSNYAN	house mouse	IGLV1
L1-14-A	CDRL1	4AT6	L	RSSTGAVTTSNYAN	house mouse	IGLV1
L1-14-A	CDRL1	1Y18	A	RSSSGAVTTSNYAT	house mouse	IGLV1
L1-14-A	CDRL1	2XZQ	L	RSSTGAVTTSNYAN	house mouse	IGLV1
L1-14-A	CDRL1	1Y18	C	RSSSGAVTTSNYAT	house mouse	IGLV1
L1-14-A	CDRL1	1A6V	N	RSSTGAVTTSNYAN	house mouse	IGLV1
L1-14-A	CDRL1	1OAU	M	RSSTGAVTTSNYAN	house mouse	IGLV1
L1-14-A	CDRL1	1F4W	L	RSSTGTVTTSNYAN	house mouse	IGLV1
L1-14-A	CDRL1	1SM3	L	RSSTGAVTTSNYAN	house mouse	IGLV1
L1-14-A	CDRL1	3CFK	E	RSSSGAVTTSNYAT	house mouse	IGLV1
L1-14-A	CDRL1	4AT6	P	RSSTGAVTTSNYAN	house mouse	IGLV1
L1-14-A	CDRL1	3CFK	L	RSSSGAVTTSNYAT	house mouse	IGLV1
L1-14-A	CDRL1	1A6U	L	RSSTGAVTTSNYAN	house mouse	IGLV1
L1-14-A	CDRL1	1IND	L	RSSTGAVTTSNYAN	house mouse	IGLV1
L1-14-A	CDRL1	1OAU	O	RSSTGAVTTSNYAN	house mouse	IGLV1
L1-14-A	CDRL1	1PG7	Y	RSSTGAVTTSNYAN	house mouse	IGLV1
L1-14-A	CDRL1	1ETZ	A	RSSTGAVTTSNYAI	house mouse	IGLV1
L1-14-A	CDRL1	1NC2	C	RSSTGAVTTSNYAN	house mouse	IGLV1
L1-14-A	CDRL1	2Y07	L	RSSTGAVTTSNYAN	house mouse	IGLV1
L1-14-A	CDRL1	3CFK	J	RSSSGAVTTSNYAT	house mouse	IGLV1
L1-14-A	CDRL1	1Q0X	L	RSSTGAVTTSNYAN	house mouse	IGLV1
L1-14-A	CDRL1	1Y0L	A	RSSSGAVTTSNYAT	house mouse	IGLV1
L1-14-A	CDRL1	3KS0	J	RSSTGAVTTSNYAN	house mouse	IGLV1
L1-14-A	CDRL1	3CFJ	L	RSSSGAVTTSNYAT	house mouse	IGLV1
L1-14-A	CDRL1	1INE	L	RSSTGAVTTSNYAN	house mouse	IGLV1

L1-14-A	CDRL1	4K3H	B	GSSTGAVTSGHYAN	human	IGLV7-46
L1-14-A	CDRL1	1OAQ	L	RSSTGAVTTSNYAN	house mouse	IGLV1
L1-14-A	CDRL1	1OAX	L	RSSTGAVTTSNYAN	house mouse	IGLV1
L1-14-A	CDRL1	4AT6	F	RSSTGAVTTSNYAN	house mouse	IGLV1
L1-14-A	CDRL1	1NGP	L	RSSTGAVTTSNYAN	house mouse	IGLV1
L1-14-A	CDRL1	1Q0Y	L	RSSTGAVTTSNYAN	house mouse	IGLV1
L1-14-A	CDRL1	1NC2	A	RSSTGAVTTSNYAN	house mouse	IGLV1
L1-14-A	CDRL1	1NJ9	L	RSSTGTITSDNYAN	house mouse	IGLV1
L1-14-A	CDRL1	1MFD	L	RSSTGTVTSGNHAN	house mouse	IGLV1
L1-14-A	CDRL1	1Y0L	E	RSSSGAVTTSNYAT	house mouse	IGLV1
L1-14-A	CDRL1	1F4X	L	RSSTGTVTTSNYAN	house mouse	IGLV1
L1-14-A	CDRL1	3T2N	L	RSSTGAVTTSNYAN	human	IGLV7-43
L1-14-A	CDRL1	1A6V	L	RSSTGAVTTSNYAN	house mouse	IGLV1
L1-14-A	CDRL1	2ZPK	L	RSSTGAVTTSNYAN	house mouse	IGLV1
L1-14-A	CDRL1	1MFE	L	RSSTGTVTSGNHAN	house mouse	IGLV1
L1-14-A	CDRL1	2Y36	L	RSSTGAVTTSNYAN	house mouse	IGLV1
L1-14-A	CDRL1	1OAR	N	RSSTGAVTTSNYAN	house mouse	IGLV1
L1-14-A	CDRL1	3KS0	L	RSSTGAVTTSNYAN	house mouse	IGLV1
L1-14-A	CDRL1	3CFJ	C	RSSSGAVTTSNYAT	house mouse	IGLV1
L1-14-A	CDRL1	2BJM	L	RSSTGAVTTSNYAN	house mouse	IGLV1
L1-14-A	CDRL1	4AT6	B	RSSTGAVTTSNYAN	house mouse	IGLV1
L1-14-A	CDRL1	1OAZ	L	RSSTGAVTTSNYAN	house mouse	IGLV1
L1-14-A	CDRL1	1ETZ	L	RSSTGAVTTSNYAI	house mouse	IGLV1
L1-14-A	CDRL1	1Y0L	C	RSSSGAVTTSNYAT	house mouse	IGLV1
L1-14-A	CDRL1	3CFK	M	RSSSGAVTTSNYAT	house mouse	IGLV1
L1-14-A	CDRL1	3T2N	M	RSSTGAVTTSNYAN	human	IGLV7-43
L1-14-A	CDRL1	3CFK	G	RSSSGAVTTSNYAT	house mouse	IGLV1
L1-14-A	CDRL1	1OAZ	N	RSSTGAVTTSNYAN	house mouse	IGLV1
L1-14-A	CDRL1	1OAY	L	RSSTGAVTTSNYAN	house mouse	IGLV1
L1-14-A	CDRL1	1DL7	L	RSSTGAVTTSNYAN	house mouse	IGLV1
L1-14-A	CDRL1	1F4Y	L	RSSTGTVTTSNYAN	house mouse	IGLV1
L1-14-A	CDRL1	1NC4	A	RSSTGAVTTSNYAN	house mouse	IGLV1
L1-14-A	CDRL1	4K3H	H	GSSTGAVTSGHYAN	human	IGLV7-46
L1-14-A	CDRL1	3CFK	O	RSSSGAVTTSNYAT	house mouse	IGLV1
L1-14-A	CDRL1	1PG7	W	RSSTGAVTTSNYAN	house mouse	IGLV1
L1-14-A	CDRL1	1MFC	L	RSSTGTVTSGNHAN	house mouse	IGLV1
L1-14-A	CDRL1	1MFB	L	RSSTGTVTSGNHAN	house mouse	IGLV1
L1-14-A	CDRL1	3CFJ	E	RSSSGAVTTSNYAT	house mouse	IGLV1
L1-14-A	CDRL1	1OAU	L	RSSTGAVTTSNYAN	house mouse	IGLV1
L1-14-A	CDRL1	1NJ9	A	RSSTGTITSDNYAN	house mouse	IGLV1
L1-14-A	CDRL1	4K3G	A	GSSTGAVTSGHYAN	human	IGLV7-46
L1-14-A	CDRL1	1Y18	L	RSSSGAVTTSNYAT	house mouse	IGLV1
L1-14-A	CDRL1	2ZPK	M	RSSTGAVTTSNYAN	house mouse	IGLV1
L1-14-A	CDRL1	1OAR	L	RSSTGAVTTSNYAN	house mouse	IGLV1
L1-14-A	CDRL1	1MFA	L	RSSTGTVTSGNHAN	house mouse	IGLV1
L1-14-A	CDRL1	3CFK	C	RSSSGAVTTSNYAT	house mouse	IGLV1
L1-14-A	CDRL1	2Y06	L	RSSTGAVTTSNYAN	house mouse	IGLV1
L1-14-A	CDRL1	4K3H	D	GSSTGAVTSGHYAN	human	IGLV7-46
L1-14-A	CDRL1	1NGQ	L	RSSTGAVTTSNYAN	house mouse	IGLV1
L1-14-B	CDRL1	4KTD	L	TLSSGFTVGRYSIF	rhesus monkey	IGLV

L1-14-B	CDRL1	4KTE	L	TLSSGINVGSYSIF	rhesus monkey	IGLV
L1-15-A	CDRL1	1MF2	L	RASESVDYYGKSFMN	house mouse	IGKV3-2
L1-15-A	CDRL1	1GGI	M	RASESVDDDGNSFLH	house mouse	IGKV3-5
L1-15-A	CDRL1	1GGC	L	RASESVDDDGNSFLH	house mouse	IGKV3-5
L1-15-A	CDRL1	3O41	B	RASQSVDYNGISYMH	house mouse	IGKV3-3
L1-15-A	CDRL1	3QRG	L	RASQSVDYNGISYMH	house mouse	IGKV3
L1-15-A	CDRL1	4HS6	A	RASESVDGYGYSFLH	house mouse	IGKV3
L1-15-A	CDRL1	1AI1	L	RASESVDSYGKSFMH	house mouse	IGKV3-10
L1-15-A	CDRL1	3WKM	M	RASESVDSYGDSFMH	house mouse	IGKV3-10
L1-15-A	CDRL1	2OR9	L	RASESVDNYGFSFMN	house mouse	IGKV3-2
L1-15-A	CDRL1	2F58	L	KASQGVDFDGASFMN	house mouse	IGKV3-4
L1-15-A	CDRL1	3ZKN	D	RASKSVDTYGHSFIH	house mouse	IGKV3-10
L1-15-A	CDRL1	4G6A	L	RASESVDGYGNSFLH	house mouse	IGKV3
L1-15-A	CDRL1	2HRP	M	RASESVDYYGKSFMN	house mouse	IGKV3-2
L1-15-A	CDRL1	3QQ9	C	RASQSVDYNGISYMH	house mouse	IGKV3-3
L1-15-A	CDRL1	3E8U	L	KASQSVDYNGDSYLN	house mouse	IGKV3-4
L1-15-A	CDRL1	4GAY	L	RASESVDGYGNSFLH	house mouse	IGKV3-10
L1-15-A	CDRL1	3ZKM	L	RASKSVDTYGHSFIH	house mouse	IGKV3-10
L1-15-A	CDRL1	1J05	L	RAGESVDIFGVGFLH	house mouse	IGKV3-5
L1-15-A	CDRL1	1J05	A	RAGESVDIFGVGFLH	house mouse	IGKV3-5
L1-15-A	CDRL1	3PHQ	A	RASKSVSSSVNSYMH	house mouse	IGKV3-12
L1-15-A	CDRL1	1IQW	L	KASQSVDYDGDSYMN	house mouse	IGKV3-4
L1-15-A	CDRL1	2XA8	L	RASQSVDYDGDSYMN	house mouse	IGKV3
L1-15-A	CDRL1	1I7Z	C	RASKSVSTSGYNYMH	house mouse	IGKV3-12
L1-15-A	CDRL1	1GGI	L	RASESVDDDGNSFLH	house mouse	IGKV3-5
L1-15-A	CDRL1	1MF2	M	RASESVDYYGKSFMN	house mouse	IGKV3-2
L1-15-A	CDRL1	2HRP	L	RASESVDYYGKSFMN	house mouse	IGKV3-2
L1-15-A	CDRL1	2NYY	C	RASESVDSYGHSFMQ	house mouse	IGKV3-5
L1-15-A	CDRL1	1F58	L	KASQGVDFDGASFMN	house mouse	IGKV3-4
L1-15-A	CDRL1	1IT9	L	KASQSVDYDGDSYMN	house mouse	IGKV3-4
L1-15-A	CDRL1	3ZKM	D	RASKSVDTYGHSFIH	house mouse	IGKV3-10
L1-15-A	CDRL1	4HS6	L	RASESVDGYGYSFLH	house mouse	IGKV3
L1-15-A	CDRL1	3PHO	A	RASKSVSSSVNSYMH	house mouse	IGKV3-12
L1-15-A	CDRL1	2OR9	M	RASESVDNYGFSFMN	house mouse	IGKV3-2
L1-15-A	CDRL1	2Y5T	B	RASKSVDSYGNSFME	house mouse	IGKV3-5
L1-15-A	CDRL1	3O41	L	RASQSVDYNGISYMH	house mouse	IGKV3-3
L1-15-A	CDRL1	1H0D	A	RASESVDNYGISFMS	house mouse	IGKV3-2
L1-15-A	CDRL1	2VL5	B	RASKSVDSYGNSFME	house mouse	IGKV3-5
L1-15-A	CDRL1	4I77	L	RASKSVDSYGNSFMH	house mouse	IGKV3
L1-15-A	CDRL1	2VL5	D	RASKSVDSYGNSFME	house mouse	IGKV3-5
L1-15-A	CDRL1	1IBG	L	RASKSVSTSGYSHIH	house mouse	IGKV3-12
L1-15-A	CDRL1	1EJO	L	RASESVDSYGNSFMH	house mouse	IGKV3-5
L1-15-A	CDRL1	3ZKN	L	RASKSVDTYGHSFIH	house mouse	IGKV3-10
L1-15-A	CDRL1	4G6A	D	RASESVDGYGNSFLH	house mouse	IGKV3
L1-15-A	CDRL1	4GAJ	L	RASESVDGYGNSFLH	house mouse	IGKV3-10
L1-15-A	CDRL1	3F58	L	KASQGVDFDGASFMN	house mouse	IGKV3-4
L1-15-A	CDRL1	3S35	L	RASESVDSYGNSFMH	house mouse	IGKV3-5
L1-15-A	CDRL1	3QQ9	L	RASQSVDYNGISYMH	house mouse	IGKV3-3
L1-15-A	CDRL1	4GAG	L	RASESVDGYGNSFLH	house mouse	IGKV3-10
L1-15-A	CDRL1	1EGJ	L	RANESVYSYGDSFMH	house mouse	IGKV3-10

L1-15-A	CDRL1	4GAY	B	RASESVDGYGNSFLH	house mouse	IGKV3-10
L1-15-A	CDRL1	4HS8	L	RASESVDNYGISFMN	house mouse	IGKV3
L1-15-A	CDRL1	2AAB	L	RASESVEYYGSSLMQ	house mouse	IGKV3-1
L1-15-A	CDRL1	3WKM	L	RASESVDSYGDSFMH	house mouse	IGKV3-10
L1-15-A	CDRL1	2GCY	A	RASESVDSYGHSFMQ	house mouse	IGKV3-5
L1-15-A	CDRL1	4FFY	L	RASESVDHYGNSFIY	house mouse	IGKV3-10
L1-15-B	CDRL1	3DGG	C	RASKSVSTSGYSYMH	house mouse	IGKV3-12
L1-15-B	CDRL1	1I7Z	A	RASKSVSTSGYNYMH	house mouse	IGKV3-12
L1-15-B	CDRL1	3DGG	A	RASKSVSTSGYSYMH	house mouse	IGKV3-12
L1-16-A	CDRL1	1PLG	L	RSSQSLVHSNGNTYLY	house mouse	IGKV1-110
L1-16-A	CDRL1	2OK0	L	RSSQSIVHSNGNTYLE	house mouse	IGKV1-117
L1-16-A	CDRL1	2OJZ	L	RSSQSIVHSNGNTYLE	house mouse	IGKV1-117
L1-16-A	CDRL1	1RUQ	L	RSSKSLLNSNGIIHMY	house mouse	IGKV2-109
L1-16-A	CDRL1	2A1W	L	RCSQSIVKSNGHTYLE	house mouse	IGKV1-117
L1-16-A	CDRL1	3IFL	L	RSSQSIVHSNGNTYLE	house mouse	IGKV1-117
L1-16-A	CDRL1	2G60	L	RSSQAIVHANGNTYLE	house mouse	IGKV1-117
L1-16-A	CDRL1	4K23	L	KSSQSLLDSDGKTYLN	house mouse	IGKV1-135
L1-16-A	CDRL1	3IFO	L	RSSQNIIHSNGNTYLE	house mouse	IGKV1-117
L1-16-A	CDRL1	1YEJ	L	KSSQSLLYSNGKTYLN	house mouse	IGKV1-133
L1-16-A	CDRL1	1CR9	L	KSSQSLLDSDGKTYLI	house mouse	IGKV1-135
L1-16-A	CDRL1	3QCU	M	TSGQSLVHINGNTYLH	house mouse	IGKV
L1-16-A	CDRL1	1ND0	E	KSSQRLLNSNGKTFLN	house mouse	IGKV1-133
L1-16-A	CDRL1	1M7I	A	RSSQSLLHSDGNTYLH	house mouse	IGKV1-110
L1-16-A	CDRL1	4HCR	N	KSSQSLLHTDGTTYLY	house mouse	IGKV
L1-16-A	CDRL1	1MRE	L	RSSQSLVHSNGNTYLH	house mouse	IGKV1-110
L1-16-A	CDRL1	1TQB	C	KSSQSLLDSDGKTYLN	house mouse	IGKV1-135
L1-16-A	CDRL1	1LO4	L	RSSQTIVHTNGNTYFE	house mouse	IGKV1-117
L1-16-A	CDRL1	2AJZ	A	RSSRSLLYKDGRTYLN	house mouse	IGKV2-112
L1-16-A	CDRL1	3QEH	B	RSSQSLLHTNGFQYLD	house mouse	IGKV
L1-16-A	CDRL1	3C6S	C	RSSKSLLHSDGITYLY	house mouse	IGKV2-109
L1-16-A	CDRL1	1MRC	L	RSSQSLVHSNGNTYLH	house mouse	IGKV1-110
L1-16-A	CDRL1	1GPO	L	RASQSLVNEDGNTYLF	house mouse	IGKV5-43
L1-16-A	CDRL1	4LEX	L	RSSQSLVHSNGNTYLH	house mouse	IGKV1-110
L1-16-A	CDRL1	1F8T	L	ESSQSLLYSNGKTYLN	house mouse	IGKV1-133
L1-16-A	CDRL1	3FO2	A	RSSQSIVHSNGNTYLQ	house mouse	IGKV1-117
L1-16-A	CDRL1	1DSF	L	RSSQNLVHSDGKTYLH	house mouse	IGKV1-110
L1-16-A	CDRL1	3AAZ	B	RSSQSIVHSNGHTYLE	house mouse	IGKV
L1-16-A	CDRL1	3IFO	B	RSSQNIIHSNGNTYLE	house mouse	IGKV1-117
L1-16-A	CDRL1	2JEL	L	RSSQSIVHGNGNTYLE	house mouse	IGKV1-117
L1-16-A	CDRL1	1KFA	M	RSSQSLVHSNGNTYLH	house mouse	IGKV1-110
L1-16-A	CDRL1	2DDQ	L	RSSQSIVHSNGNTYLE	house mouse	IGKV1-117
L1-16-A	CDRL1	1QYG	L	RSSKSLLHSNGYTYLH	house mouse	IGKV2-137
L1-16-A	CDRL1	1IGJ	C	RSSQSLVHSNGNTYLN	house mouse	IGKV1-110
L1-16-A	CDRL1	3IFN	L	RSSQSIVHSNGNTYLE	house mouse	IGKV1-117
L1-16-A	CDRL1	1UB5	L	RSTKSLLHSNGITYLY	house mouse	IGKV2-109
L1-16-A	CDRL1	3O6M	L	KSGQSLLYSDGKTYLN	house mouse	IGKV1-135
L1-16-A	CDRL1	1TPX	C	KSSQSLLDSDGKTYLN	house mouse	IGKV1-135
L1-16-A	CDRL1	2UYL	X	RSSQSIVHSNGDTYLE	house mouse	IGKV1-117
L1-16-A	CDRL1	1T2Q	L	RSSQSLVHSSGNTYLH	house mouse	IGKV1-117
L1-16-A	CDRL1	1UZ6	M	RSSKSLLYSNGITYLY	house mouse	IGKV2-109

L1-16-A	CDRL1	1S3K	L	RSSQRIVHSNGNTYLE	house mouse	IGKV
L1-16-A	CDRL1	3BAE	L	RSSQSIVHSNGHTYLE	house mouse	IGKV1-117
L1-16-A	CDRL1	2AI0	O	RCSQSIVKSNGHTYLE	house mouse	IGKV1-117
L1-16-A	CDRL1	2AI0	N	RCSQSIVKSNGHTYLE	house mouse	IGKV1-117
L1-16-A	CDRL1	1FL5	L	RSSQSIVHSNGNTYLE	house mouse	IGKV1-117
L1-16-A	CDRL1	3BKM	L	RSSQSIVHSNGHTYLE	house mouse	IGKV1-117
L1-16-A	CDRL1	1UB6	B	RSTKSLLHSNGITYLY	house mouse	IGKV2-109
L1-16-A	CDRL1	1UZ6	E	RSSKSLLYSNGITYLY	house mouse	IGKV2-109
L1-16-A	CDRL1	4AEI	M	RSSQSIVHSNGNTYLE	house mouse	IGKV1-117
L1-16-A	CDRL1	1DLF	L	RSSQSLVHSNGNTYLH	house mouse	IGKV1-110
L1-16-A	CDRL1	1YEF	L	KSSQSLLYSNGKTYLN	house mouse	IGKV1-133
L1-16-A	CDRL1	2AJZ	L	RSSRSLLYKDGRTYLN	house mouse	IGKV2-112
L1-16-A	CDRL1	2O5Z	L	RSSQSLVHSNGNTYLH	house mouse	IGKV1-110
L1-16-A	CDRL1	3FO0	L	RSSQSIVHSNGNTYLE	house mouse	IGKV1-117
L1-16-A	CDRL1	3CFB	A	RSTKSLLHSNGITYLY	house mouse	IGKV2-109
L1-16-A	CDRL1	1MRD	L	RSSQSLVHSNGNTYLH	house mouse	IGKV1-110
L1-16-A	CDRL1	3QCT	L	TSGQSLVHINGNTYLH	house mouse	IGKV
L1-16-A	CDRL1	1KEL	L	RFSQSIVHSNGNTYLE	house mouse	IGKV1-117
L1-16-A	CDRL1	2W65	B	KSSQSLLDSDGKTYLN	house mouse	IGKV1-135
L1-16-A	CDRL1	1C1E	L	RSSQSLVHSNGNTYLH	house mouse	IGKV1-110
L1-16-A	CDRL1	4F37	K	RSSQTILHSNGNTYLE	house mouse	IGKV1-117
L1-16-A	CDRL1	3CFB	L	RSTKSLLHSNGITYLY	house mouse	IGKV2-109
L1-16-A	CDRL1	1XGY	L	RSSKSLLHSNGITYLY	house mouse	IGKV2-109
L1-16-A	CDRL1	1A3L	L	RSSKSLLNSNGIIHMY	house mouse	IGKV2-109
L1-16-A	CDRL1	1UZ6	L	RSSKSLLYSNGITYLY	house mouse	IGKV2-109
L1-16-A	CDRL1	4AEI	L	RSSQSIVHSNGNTYLE	house mouse	IGKV1-117
L1-16-A	CDRL1	2AJY	L	RSSRSLLYKDGRTYLN	house mouse	IGKV2-112
L1-16-A	CDRL1	3UYR	L	RSSQSLVHSNGNTYLH	house mouse	IGKV1-110
L1-16-A	CDRL1	1QFU	L	RSSQTLVHSNGNTYLH	house mouse	IGKV1-110
L1-16-A	CDRL1	1MIE	L	RSSKSLLHSNGNTYLN	house mouse	IGKV2-137
L1-16-A	CDRL1	3HR5	P	RSSQSLVHNNANTYLH	house mouse	IGKV
L1-16-A	CDRL1	2IPU	K	RSSQSIVHSNGNTYLE	house mouse	IGKV1-117
L1-16-A	CDRL1	1XGY	M	RSSKSLLHSNGITYLY	house mouse	IGKV2-109
L1-16-A	CDRL1	2CGR	L	RPSQSLVHSNGNTYLH	house mouse	IGKV1-110
L1-16-A	CDRL1	1DBM	L	RSSQSLIHSNGNTYLH	house mouse	IGKV1-110
L1-16-A	CDRL1	1TET	L	KSSQSIVHSSGNTYFE	house mouse	IGKV1-117
L1-16-A	CDRL1	2AJV	L	RSSRSLLYKDGRTYLN	house mouse	IGKV2-112
L1-16-A	CDRL1	3QCU	L	TSGQSLVHINGNTYLH	house mouse	IGKV
L1-16-A	CDRL1	1UB5	B	RSTKSLLHSNGITYLY	house mouse	IGKV2-109
L1-16-A	CDRL1	3PP3	K	RSSKSLLHSNGITYLY	house mouse	IGKV
L1-16-A	CDRL1	1MJ7	L	RSSKSLLHSNGNTYLN	house mouse	IGKV2-137
L1-16-A	CDRL1	3U0T	C	KSSQSLLYSDAKTYLN	house mouse	IGKV
L1-16-A	CDRL1	2DQU	L	RSSQTIVHSNGDTYLD	house mouse	IGKV1-117
L1-16-A	CDRL1	1KFA	L	RSSQSLVHSNGNTYLH	house mouse	IGKV1-110
L1-16-A	CDRL1	2PCP	A	RSSQTIVHSNGNTYLE	house mouse	IGKV1-117
L1-16-A	CDRL1	1MRF	L	RSSQSLVHSNGNTYLH	house mouse	IGKV1-110
L1-16-A	CDRL1	1MJJ	L	RSSKSLLHSNGNTYLY	house mouse	IGKV2-137
L1-16-A	CDRL1	1RUM	L	KSSQRLLNSNGKTFLN	house mouse	IGKV1-133
L1-16-A	CDRL1	1GPO	M	RASQSLVNEDGNTYLF	house mouse	IGKV5-43
L1-16-A	CDRL1	1FL3	B	RSTKSLLHSNGITYLY	house mouse	IGKV2-109

L1-16-A	CDRL1	4AEI	N	RSSQSIVHSNGNTYLE	house mouse	IGKV1-117
L1-16-A	CDRL1	1YEE	L	KSSQSLLYSNGKTYLS	house mouse	IGKV1-133
L1-16-A	CDRL1	2AI0	M	RCSQSIVKSNGHTYLE	house mouse	IGKV1-117
L1-16-A	CDRL1	2VQ1	E	RSSQSLVHSNGNTFLQ	house mouse	IGKV1-110
L1-16-A	CDRL1	1MJ8	L	RSSKSLLHSNGNTYLY	house mouse	IGKV2-137
L1-16-A	CDRL1	1RMF	L	RSSQSLVHSNGNNYLH	house mouse	IGKV1-110
L1-16-A	CDRL1	2PCP	C	RSSQTIVHSNGNTYLE	house mouse	IGKV1-117
L1-16-A	CDRL1	2IPT	L	RSSQSIVHSNGNTYLE	house mouse	IGKV1-117
L1-16-A	CDRL1	3PP3	L	RSSKSLLHSNGITYLY	house mouse	IGKV
L1-16-A	CDRL1	1RUL	L	KSSQRLLNSNGKTFLN	house mouse	IGKV1-133
L1-16-A	CDRL1	1S5I	L	ESSQSLLYSNGKTYLN	house mouse	IGKV1-133
L1-16-A	CDRL1	1YNL	L	RPSQSLVHSNGNTYLH	house mouse	IGKV1-110
L1-16-A	CDRL1	4HIX	L	KSSQSLLDSDGKTYLN	house mouse	IGKV
L1-16-A	CDRL1	2R0Z	L	RSSQSIVHSNGNTYLE	house mouse	IGKV1-117
L1-16-A	CDRL1	2HKH	L	RSSQSLVHSNGNTYLH	house mouse	IGKV1-110
L1-16-A	CDRL1	1DBB	L	RSSQSLIHSNGNTYLH	house mouse	IGKV1-110
L1-16-A	CDRL1	1RIV	L	RSSKSLLHSNGYTYLH	house mouse	IGKV2-137
L1-16-A	CDRL1	2UYL	A	RSSQSIVHSNGDTYLE	house mouse	IGKV1-117
L1-16-A	CDRL1	1MPA	L	RSSQALVHSNGNTYLH	house mouse	IGKV1-110
L1-16-A	CDRL1	1IGI	L	RSSQSLVHSNGNTYLN	house mouse	IGKV1-110
L1-16-A	CDRL1	2AK1	L	RSSRSLLYKDGRTYLN	house mouse	IGKV2-112
L1-16-A	CDRL1	3HR5	A	RSSQSLVHNNANTYLH	house mouse	IGKV
L1-16-A	CDRL1	1ND0	A	KSSQRLLNSNGKTFLN	house mouse	IGKV1-133
L1-16-A	CDRL1	2AJX	L	RSSRSLLYKDGRTYLN	house mouse	IGKV2-112
L1-16-A	CDRL1	1RUA	L	KSSQRLLNSNGKTFLN	house mouse	IGKV1-133
L1-16-A	CDRL1	3QG6	L	RSSQRLVHSNGNIYLH	house mouse	IGKV1-110
L1-16-A	CDRL1	2G2R	A	RSSQSLLYINGKTHLN	house mouse	IGKV1-133
L1-16-A	CDRL1	2O5X	L	RSSQSLVHSNGNTYLH	house mouse	IGKV1-110
L1-16-A	CDRL1	3EYU	L	RSSQSIVHSNGNTYLE	house mouse	IGKV1-117
L1-16-A	CDRL1	1N4X	M	RSSQTIVHNNGNTYLE	house mouse	IGKV1-117
L1-16-A	CDRL1	3IF1	A	RSSQSLVHSNGNTYLH	house mouse	IGKV1-110
L1-16-A	CDRL1	3QCV	M	TSGQSLVHINGNTYLH	house mouse	IGKV
L1-16-A	CDRL1	2EH8	L	KSSQSLLYSNGKTYLN	house mouse	IGKV1-133
L1-16-A	CDRL1	3HR5	Q	RSSQSLVHNNANTYLH	house mouse	IGKV
L1-16-A	CDRL1	1N4X	L	RSSQTIVHNNGNTYLE	house mouse	IGKV1-117
L1-16-A	CDRL1	3C6S	E	RSSKSLLHSDGITYLY	house mouse	IGKV2-109
L1-16-A	CDRL1	1LO2	L	TSSQTIVHTNGNTYLE	house mouse	IGKV1-117
L1-16-A	CDRL1	4JN2	A	KSSQSLLYTDGKTYLY	house mouse	IGKV1-133
L1-16-A	CDRL1	1UB6	L	RSTKSLLHSNGITYLY	house mouse	IGKV2-109
L1-16-A	CDRL1	2OJZ	M	RSSQSIVHSNGNTYLE	house mouse	IGKV1-117
L1-16-A	CDRL1	1F3D	J	RSSQSIFHSDGKTYLE	house mouse	IGKV1-117
L1-16-A	CDRL1	1PZ5	A	RSSQSLLHSDGNTYLH	house mouse	IGKV1-110
L1-16-A	CDRL1	1UZ8	A	RSSKSLLYSNGITYLY	house mouse	IGKV2-109
L1-16-A	CDRL1	1UZ6	V	RSSKSLLYSNGITYLY	house mouse	IGKV2-109
L1-16-A	CDRL1	1TQC	C	KSSQSLLDSDGKTYLN	house mouse	IGKV1-135
L1-16-A	CDRL1	2VQ1	A	RSSQSLVHSNGNTFLQ	house mouse	IGKV1-110
L1-16-A	CDRL1	4JN1	L	KSSQSLLYTDGKTYLY	house mouse	IGKV1-133
L1-16-A	CDRL1	3C6S	A	RSSKSLLHSDGITYLY	house mouse	IGKV2-109
L1-16-A	CDRL1	1ZEA	L	KSSQSIVHSSGNTYFE	house mouse	IGKV1-117
L1-16-A	CDRL1	2UYL	M	RSSQSIVHSNGDTYLE	house mouse	IGKV1-117

L1-16-A	CDRL1	2D03	L	RSSQSLVHSSGNTYLH	house mouse	IGKV1-110
L1-16-A	CDRL1	3LEX	B	KSSQSLLDSDGKTYLN	house mouse	IGKV1-135
L1-16-A	CDRL1	3BZ4	C	RSSKSLLHSDGITYLY	house mouse	IGKV2-109
L1-16-A	CDRL1	4F37	L	RSSQTILHSNGNTYLE	house mouse	IGKV1-117
L1-16-A	CDRL1	2IQA	L	RSSQSIVHSNGNTYLE	house mouse	IGKV1-117
L1-16-A	CDRL1	2HKF	L	RSSQSLVHSNGNTYLH	house mouse	IGKV1-110
L1-16-A	CDRL1	2C1P	L	KSSQSLLYSNGKTYLN	house mouse	IGKV1-133
L1-16-A	CDRL1	1ND0	C	KSSQRLLNSNGKTFLN	house mouse	IGKV1-133
L1-16-A	CDRL1	1FL3	L	RSTKSLLHSNGITYLY	house mouse	IGKV2-109
L1-16-A	CDRL1	3BZ4	E	RSSKSLLHSDGITYLY	house mouse	IGKV2-109
L1-16-A	CDRL1	1RFD	L	RSSKSLLHSNGYTYLH	house mouse	IGKV2-137
L1-16-A	CDRL1	1NCW	L	KSSQRLLNSNGKTFLN	house mouse	IGKV1-133
L1-16-A	CDRL1	2AI0	L	RCSQSIVKSNGHTYLE	house mouse	IGKV1-117
L1-16-A	CDRL1	2R0W	L	RSSQSIVHSNGNTYLE	house mouse	IGKV1-117
L1-16-A	CDRL1	1YEC	L	KSSQSLLYSNGKTYLN	house mouse	IGKV1-133
L1-16-A	CDRL1	4JN2	L	KSSQSLLYTDGKTYLY	house mouse	IGKV1-133
L1-16-A	CDRL1	1LO3	L	TSSQTIVHTNGNTYLE	house mouse	IGKV1-117
L1-16-A	CDRL1	1WT5	D	RSSQNIVHNNGITYLE	house mouse	IGKV
L1-16-A	CDRL1	3O6K	L	KSGQSLLYSDGKTYLN	house mouse	IGKV1-135
L1-16-A	CDRL1	1Q72	L	RSSKSLLHSNGYTYLH	house mouse	IGKV2-137
L1-16-A	CDRL1	1KN2	L	KSSQSLLYSNGKTYLN	house mouse	IGKV1-133
L1-16-A	CDRL1	1UZ8	L	RSSKSLLYSNGITYLY	house mouse	IGKV2-109
L1-16-A	CDRL1	2GK0	L	RSSQSIVHSNGNTYLE	house mouse	IGKV1-117
L1-16-A	CDRL1	1T66	C	RSSQSLVHSNGNTYLH	house mouse	IGKV1-110
L1-16-A	CDRL1	3IET	C	RSSQSLVHSNGNTYLH	house mouse	IGKV1-110
L1-16-A	CDRL1	2IQA	A	RSSQSIVHSNGNTYLE	house mouse	IGKV1-117
L1-16-A	CDRL1	4HCR	L	KSSQSLLHTDGTTYLY	house mouse	IGKV
L1-16-A	CDRL1	1JGU	L	RSSQSLVHSNGNTYLH	house mouse	IGKV1-110
L1-16-A	CDRL1	3GGW	A	RSSKSLLHSDGITYLY	house mouse	IGKV2-109
L1-16-A	CDRL1	4LKX	B	RSSQSIVHSNGNTYLE	house mouse	IGKV
L1-16-A	CDRL1	1YEH	L	KSSQSLLYSNGKTYLN	house mouse	IGKV1-133
L1-16-A	CDRL1	3IET	A	RSSQSLVHSNGNTYLH	house mouse	IGKV1-110
L1-16-A	CDRL1	1RUP	L	KSSQRLLNSNGKTFLN	house mouse	IGKV1-133
L1-16-A	CDRL1	1FL5	A	RSSQSIVHSNGNTYLE	house mouse	IGKV1-117
L1-16-A	CDRL1	1RU9	L	KSSQRLLNSNGKTFLN	house mouse	IGKV1-133
L1-16-A	CDRL1	1JGV	L	RSSQSLVHSNGNTYLH	house mouse	IGKV1-110
L1-16-A	CDRL1	3HR5	L	RSSQSLVHNNANTYLH	house mouse	IGKV
L1-16-A	CDRL1	3EYS	L	RSSQSIVHSNGNTYLE	house mouse	IGKV1-117
L1-16-A	CDRL1	2CJU	L	RSSQSIVHSNGNTYLE	house mouse	IGKV1-117
L1-16-A	CDRL1	2AJU	L	RSSRSLLYKDGRTYLN	house mouse	IGKV2-112
L1-16-A	CDRL1	2C1P	A	KSSQSLLYSNGKTYLN	house mouse	IGKV1-133
L1-16-A	CDRL1	3QG6	A	RSSQRLVHSNGNIYLH	house mouse	IGKV1-110
L1-16-A	CDRL1	3LEY	L	KSSQSLLDSDGKTYLN	house mouse	IGKV1-135
L1-16-A	CDRL1	1I9J	L	RSSQSIVHSNGNSYLE	house mouse	IGKV1-117
L1-16-A	CDRL1	1KN4	L	KSSQSLLYSNGKTYLN	house mouse	IGKV1-133
L1-16-A	CDRL1	2IPU	L	RSSQSIVHSNGNTYLE	house mouse	IGKV1-117
L1-16-A	CDRL1	1F3D	L	RSSQSIFHSDGKTYLE	house mouse	IGKV1-117
L1-16-A	CDRL1	3QEH	F	RSSQSLLHTNGFQYLD	house mouse	IGKV
L1-16-A	CDRL1	1RUK	L	KSSQRLLNSNGKTFLN	house mouse	IGKV1-133
L1-16-A	CDRL1	2IGF	L	RSNQTILLSDGDTYLE	house mouse	IGKV1-117

L1-16-A	CDRL1	3GGW	C	RSSKSLLHSDGITYLY	house mouse	IGKV2-109
L1-16-A	CDRL1	1RIU	L	RSSKSLLHSNGYTYLH	house mouse	IGKV2-137
L1-16-A	CDRL1	1LO0	L	TSSQTIVHTNGNTYLE	house mouse	IGKV1-117
L1-16-A	CDRL1	2W60	B	KSSQSLLDSDGKTYLN	house mouse	IGKV1-135
L1-16-A	CDRL1	2C1O	L	KSSQSLLYSNGKTYLN	house mouse	IGKV1-133
L1-16-A	CDRL1	2DLF	L	RSSQSLVHSNGNTYLH	house mouse	IGKV1-110
L1-16-A	CDRL1	1LO3	X	TSSQTIVHTNGNTYLE	house mouse	IGKV1-117
L1-16-A	CDRL1	2W65	D	KSSQSLLDSDGKTYLN	house mouse	IGKV1-135
L1-16-A	CDRL1	3SGD	I	KSSQSLLDSDGKTYLN	house mouse	IGKV1-135
L1-16-A	CDRL1	3QG7	L	RSSQRLVHSNGNIYLH	house mouse	IGKV1-110
L1-16-A	CDRL1	3EYV	L	RSSQRIVHSNGNTYLE	house mouse	IGKV1-117
L1-16-A	CDRL1	3AAZ	L	RSSQSIVHSNGHTYLE	house mouse	IGKV
L1-16-A	CDRL1	2H1P	L	RSSQSLVHSNGNTYLH	house mouse	IGKV1-110
L1-16-A	CDRL1	1M7D	A	RSSQSLLHSDGNTYLH	house mouse	IGKV1-110
L1-16-A	CDRL1	1EHL	L	RSSQSIVHSNGNTYLE	house mouse	IGKV1-117
L1-16-A	CDRL1	1ND0	G	KSSQRLLNSNGKTFLN	house mouse	IGKV1-133
L1-16-A	CDRL1	1DBA	L	RSSQSLIHSNGNTYLH	house mouse	IGKV1-110
L1-16-A	CDRL1	3BKC	L	RSSQSIVHSNGHTYLE	house mouse	IGKV1-117
L1-16-A	CDRL1	2AJS	L	RSSRSLLYKDGRTYLN	house mouse	IGKV2-112
L1-16-A	CDRL1	3SGD	L	KSSQSLLDSDGKTYLN	house mouse	IGKV1-135
L1-16-A	CDRL1	3V52	L	RSSQSLVHSNGNTYLH	house mouse	IGKV1-110
L1-16-A	CDRL1	3V4U	L	RSSQSLVHSNGNTYLH	house mouse	IGKV1-110
L1-16-A	CDRL1	1M71	A	RSSQSLLHSDGNTYLH	house mouse	IGKV1-110
L1-16-A	CDRL1	3FO2	L	RSSQSIVHSNGNTYLQ	house mouse	IGKV1-117
L1-16-A	CDRL1	1YNK	L	RPSQSLVHSNGNTYLH	house mouse	IGKV1-110
L1-16-A	CDRL1	1YEG	L	KSSQSLLYSNGKTYLN	house mouse	IGKV1-133
L1-16-A	CDRL1	2Z4Q	A	RSSQNIVHNNGITYLE	house mouse	IGKV1-117
L1-16-A	CDRL1	3GNM	L	RSSQTIVYSNGNTYLE	house mouse	IGKV1-117
L1-16-A	CDRL1	3IF1	C	RSSQSLVHSNGNTYLH	house mouse	IGKV1-110
L1-16-A	CDRL1	1WZ1	L	RSSQSLVHSNGNTYLH	house mouse	IGKV1-110
L1-16-A	CDRL1	3SGE	L	KSSQSLLDSDGKTYLN	house mouse	IGKV1-135
L1-16-A	CDRL1	3CFC	L	RSTKSLLHSNGITYLY	house mouse	IGKV2-109
L1-16-A	CDRL1	1QKZ	L	RSSQSLVHSNGNTYLH	house mouse	IGKV1-110
L1-16-A	CDRL1	1A4J	L	RSSQSLVHSNGNTYLH	house mouse	IGKV1-110
L1-16-A	CDRL1	3QEH	D	RSSQSLLHTNGFQYLD	house mouse	IGKV
L1-16-A	CDRL1	1MJU	L	RSSKSLLHSNGNTYLY	house mouse	IGKV2-137
L1-16-A	CDRL1	1DBJ	L	RSSQSLIHSNGNTYLH	house mouse	IGKV1-110
L1-16-A	CDRL1	1A4K	L	RSSQSLLHSNGNTYLH	house mouse	IGKV1-110
L1-16-A	CDRL1	3BZ4	A	RSSKSLLHSDGITYLY	house mouse	IGKV2-109
L1-16-A	CDRL1	1YEK	L	KSSQSLLYSNGKTYLN	house mouse	IGKV1-133
L1-16-A	CDRL1	3LEX	L	KSSQSLLDSDGKTYLN	house mouse	IGKV1-135
L1-16-A	CDRL1	3EYV	A	RSSQRIVHSNGNTYLE	house mouse	IGKV1-117
L1-16-A	CDRL1	3QCV	L	TSGQSLVHINGNTYLH	house mouse	IGKV
L1-16-A	CDRL1	1AD9	L	RSSKSLLHSNGDTFLY	house mouse	IGKV
L1-16-A	CDRL1	1YEI	L	KSSQSLLYSNGKTYLN	house mouse	IGKV1-133
L1-16-A	CDRL1	2UYL	V	RSSQSIVHSNGDTYLE	house mouse	IGKV1-117
L1-16-A	CDRL1	3QEH	H	RSSQSLLHTNGFQYLD	house mouse	IGKV
L1-16-A	CDRL1	3UO1	L	RSSQSLVHSNGNTYLH	house mouse	IGKV1-110
L1-16-A	CDRL1	1MJJ	A	RSSKSLLHSNGNTYLY	house mouse	IGKV2-137
L1-16-A	CDRL1	1IGJ	A	RSSQSLVHSNGNTYLN	house mouse	IGKV1-110

L1-16-A	CDRL1	1T66	L	RSSQSLVHSNGNTYLH	house mouse	IGKV1-110
L1-16-A	CDRL1	2DQT	L	RSSQTIVHSNGDTYLD	house mouse	IGKV1-117
L1-16-A	CDRL1	4DTG	L	KSSQSLLESDGKTYLN	house mouse	IGKV
L1-16-A	CDRL1	1CGS	L	RPSQSLVHSNGNTYLH	house mouse	IGKV1-110
L1-16-A	CDRL1	1LO2	X	TSSQTIVHTNGNTYLE	house mouse	IGKV1-117
L1-16-A	CDRL1	1WT5	C	RSSQNIVHNNGITYLE	house mouse	IGKV
L1-16-A	CDRL1	3GKW	L	RSSQNIVHSNGNTYLD	house mouse	IGKV
L1-16-A	CDRL1	3O6L	L	KSGQSLLYSDGKTYLN	house mouse	IGKV1-135
L1-16-A	CDRL1	2C1O	A	KSSQSLLYSNGKTYLN	house mouse	IGKV1-133
L1-16-A	CDRL1	1KEG	L	RSSQSIVHSNGNTYLE	house mouse	IGKV1-117
L1-16-A	CDRL1	1A4J	A	RSSQSLVHSNGNTYLH	house mouse	IGKV1-110
L1-16-A	CDRL1	3BKJ	L	RSSQSIVHSNGHTYLE	house mouse	IGKV1-117
L1-16-A	CDRL1	3BZ4	G	RSSKSLLHSDGITYLY	house mouse	IGKV2-109
L1-16-A	CDRL1	2A1W	M	RCSQSIVKSNGHTYLE	house mouse	IGKV1-117
L1-16-A	CDRL1	2MPA	L	RSSQALVHSNGNTYLH	house mouse	IGKV1-110
L1-16-A	CDRL1	1F90	L	ESSQSLLYSNGKTYLN	house mouse	IGKV1-133
L1-16-A	CDRL1	2A77	L	RCSQSIVKSNGHTYLE	house mouse	IGKV1-117
L1-16-A	CDRL1	3U0T	A	KSSQSLLYSDAKTYLN	house mouse	IGKV
L1-16-A	CDRL1	3C6S	G	RSSKSLLHSDGITYLY	house mouse	IGKV2-109
L1-16-A	CDRL1	3PP4	L	RSSKSLLHSNGITYLY	house mouse	IGKV
L1-16-A	CDRL1	2G2R	L	RSSQSLLYINGKTHLN	house mouse	IGKV1-133
L1-16-A	CDRL1	3FO1	A	RSSQSIVHSNGNTYLA	house mouse	IGKV1-117
L1-16-A	CDRL1	1RUR	L	RSSKSLLNSNGIIHMY	house mouse	IGKV2-109
L1-16-A	CDRL1	3FO1	L	RSSQSIVHSNGNTYLA	house mouse	IGKV1-117
L1-16-A	CDRL1	4HXB	L	RSSQTIVHSNGNTYLE	house mouse	IGKV1-117
L1-16-A	CDRL1	1LO0	X	TSSQTIVHTNGNTYLE	house mouse	IGKV1-117
L1-16-A	CDRL1	3SGE	I	KSSQSLLDSDGKTYLN	house mouse	IGKV1-135
L1-16-A	CDRL1	1MNU	L	RSSQALVHSNGNTYLH	house mouse	IGKV1-110
L1-16-A	CDRL1	3VW3	L	RSSQNIVHSNGYTYLE	house mouse	IGKV1-117
L1-16-A	CDRL1	1AD9	A	RSSKSLLHSNGDTFLY	house mouse	IGKV
L1-16-A	CDRL1	1A4K	A	RSSQSLLHSNGNTYLH	house mouse	IGKV1-110
L1-16-A	CDRL1	2IQ9	L	RSSQSIVHSNGNTYLE	house mouse	IGKV1-117
L1-17-A	CDRL1	1Q9O	C	KSSQSLLNSRTRKSYLA	house mouse	IGKV8-21
L1-17-A	CDRL1	3OKO	A	KSSQSLLNSRTRKNYLA	house mouse	IGKV8-21
L1-17-A	CDRL1	4LVE	A	KSSQSVLYSSNSTNYLA	human	IGKV4-1
L1-17-A	CDRL1	3HZY	A	KSSQSLLNSRTRKNYLA	house mouse	IGKV8-21
L1-17-A	CDRL1	3DUS	A	KSSQSLFKSRNQKNYLA	house mouse	IGKV8-21
L1-17-A	CDRL1	3IJS	A	KSSQSLLNSRTRKNYLA	house mouse	IGKV8-21
L1-17-A	CDRL1	4LVE	B	KSSQSVLYSSNSTNYLA	human	IGKV4-1
L1-17-A	CDRL1	1ZA6	A	KSSQSLLYSGNQKNYLA	house mouse	IGKV
L1-17-A	CDRL1	43CA	C	KSSQSLLNISNQKNYLA	house mouse	IGKV8-24
L1-17-A	CDRL1	1SBS	L	KSSQSLLYSSNQMNYLA	house mouse	IGKV8-30
L1-17-A	CDRL1	1EEQ	B	KSSQSVLDSSNSKNYLA	human	IGKV4-1
L1-17-A	CDRL1	1IFH	L	TSSQSLFNSGKQKNYLT	house mouse	IGKV8-19
L1-17-A	CDRL1	3OKM	A	KSSQSLLNSRTRKNYLA	house mouse	IGKV8-21
L1-17-A	CDRL1	2R2B	A	KSSQSLLNSRTRKNYLA	house mouse	IGKV8-21
L1-17-A	CDRL1	1Q9L	A	KSSQSLLNSRTRKNYLA	house mouse	IGKV8-21
L1-17-A	CDRL1	3BPC	A	KSSQSLLNSRTRKNYLA	house mouse	IGKV8-21
L1-17-A	CDRL1	3QOT	L	KSSQSVLYSSNNKNYLA	human	IGKV4-1
L1-17-A	CDRL1	3MNZ	A	KSSQSLLNSRNERNYLA	house mouse	IGKV8-24

L1-17-A	CDRL1	1A5F	L	KSSQSLLNSGAQKNYLT	house mouse	IGKV8-19
L1-17-A	CDRL1	3Q3G	F	KSSQNLLYSSNQKNYLA	house mouse	IGKV8-30
L1-17-A	CDRL1	1EEU	B	KSSQSVLDSSNSKNYLA	human	IGKV4-1
L1-17-A	CDRL1	3DUU	A	KSSQSLFKSRNQKNYLA	house mouse	IGKV8-21
L1-17-A	CDRL1	1EEU	A	KSSQSVLDSSNSKNYLA	human	IGKV4-1
L1-17-A	CDRL1	2G5B	C	KSSQSLFNSKTRRNYLA	house mouse	IGKV8-21
L1-17-A	CDRL1	1ZA6	C	KSSQSLLYSGNQKNYLA	house mouse	IGKV
L1-17-A	CDRL1	3T77	A	KSSQSLLNSRTRKNYLA	house mouse	IGKV8-21
L1-17-A	CDRL1	1A3R	L	KSSQSLLNSRTQKNYLT	house mouse	IGKV8-19
L1-17-A	CDRL1	3MNV	C	KSSQSLLNSRNERNYLA	house mouse	IGKV8-24
L1-17-A	CDRL1	3GI8	L	KSSQSLLNTSNQKNYLA	house mouse	IGKV8-24
L1-17-A	CDRL1	3DV6	A	KSSQSLFKSRNQKNYLA	house mouse	IGKV8-21
L1-17-A	CDRL1	2G5B	G	KSSQSLFNSKTRRNYLA	house mouse	IGKV8-21
L1-17-A	CDRL1	3T4Y	A	KSSQSLLNSRTRKNYLA	house mouse	IGKV8-21
L1-17-A	CDRL1	1NLB	L	KSSQSLLNSRTRKNYLA	house mouse	IGKV8-21
L1-17-A	CDRL1	2R1Y	A	KSSQSLLNSRTRKNYLA	house mouse	IGKV8-21
L1-17-A	CDRL1	3OKL	A	KSSQSLLNSRTRKNYLA	house mouse	IGKV8-21
L1-17-A	CDRL1	1EK3	A	KSSQNLLDSSFDTNTLA	human	IGKV4-1
L1-17-A	CDRL1	2LVE	A	KSSQSVLYSSNSKNYLA	human	IGKV4-1
L1-17-A	CDRL1	2R1X	A	KSSQSLLNSRTRKNYLA	house mouse	IGKV8-21
L1-17-A	CDRL1	5LVE	A	KSSQSVLYSSNSKNYLA	human	IGKV4-1
L1-17-A	CDRL1	3GI9	L	KSSQSLLNTSNQKNYLA	house mouse	IGKV8-24
L1-17-A	CDRL1	1QAC	B	KSSQSVLYSSNSKNYLA	human	IGKV4-1
L1-17-A	CDRL1	1N64	L	KSSQSLLNSRTRKNYLA	house mouse	IGKV8-21
L1-17-A	CDRL1	1ZA6	G	KSSQSLLYSGNQKNYLA	house mouse	IGKV
L1-17-A	CDRL1	4K3J	L	KSSQSLLYTSSQKNYLA	human	IGKV1D-13
L1-17-A	CDRL1	1ZA6	E	KSSQSLLYSGNQKNYLA	house mouse	IGKV
L1-17-A	CDRL1	1EEQ	A	KSSQSVLDSSNSKNYLA	human	IGKV4-1
L1-17-A	CDRL1	2R1W	A	KSSQSLLNSRTRKNYLA	house mouse	IGKV8-21
L1-17-A	CDRL1	1EFQ	A	KSSQSVLYSSNSKNYLA	human	IGKV4-1
L1-17-A	CDRL1	1Q9W	C	KSSQSLLNSRTRKSYLA	house mouse	IGKV8-21
L1-17-A	CDRL1	3HZM	A	KSSQSLLNSRTRKNYLA	house mouse	IGKV8-21
L1-17-A	CDRL1	2IMM	A	KSSQSLLNSGNQKNFLA	house mouse	IGKV8-28
L1-17-A	CDRL1	1QAC	A	KSSQSVLYSSNSKNYLA	human	IGKV4-1
L1-17-A	CDRL1	3DUS	C	KSSQSLFKSRNQKNYLA	house mouse	IGKV8-21
L1-17-A	CDRL1	3UJT	L	KSSQSLLNSSNQKNYLA	house mouse	IGKV8-24
L1-17-A	CDRL1	3T65	A	KSSQSLLNSRTRKNYLA	house mouse	IGKV8-21
L1-17-A	CDRL1	1Q9W	A	KSSQSLLNSRTRKSYLA	house mouse	IGKV8-21
L1-17-A	CDRL1	1MHH	C	KSSQSLLNSRTRKNYLA	house mouse	IGKV8-21
L1-17-A	CDRL1	43CA	E	KSSQSLLNISNQKNYLA	house mouse	IGKV8-24
L1-17-A	CDRL1	3LVE	A	KSSQSVLYSSNSKNYLA	human	IGKV4-1
L1-17-A	CDRL1	3MO1	A	KSSQSLLNSRNERNYLA	house mouse	IGKV8-24
L1-17-A	CDRL1	1MHH	A	KSSQSLLNSRTRKNYLA	house mouse	IGKV8-21
L1-17-A	CDRL1	2R2H	A	KSSQSLLNSRTRKNYLA	house mouse	IGKV8-21
L1-17-A	CDRL1	3IJH	A	KSSQSLLNSRTRKNYLA	house mouse	IGKV8-21
L1-17-A	CDRL1	1IL1	B	KSSQSLLYSRNQMNYLS	house mouse	IGKV8-30
L1-17-A	CDRL1	1Q9K	A	KSSQSLLNSRTRKNYLA	house mouse	IGKV8-21
L1-17-A	CDRL1	2G5B	E	KSSQSLFNSKTRRNYLA	house mouse	IGKV8-21
L1-17-A	CDRL1	2CK0	L	KSSQNLLHSITRKNYLA	house mouse	IGKV8-21
L1-17-A	CDRL1	3Q3G	C	KSSQNLLYSSNQKNYLA	house mouse	IGKV8-30

L1-17-A	CDRL1	3OKD	A	KSSQSLLNSRTRKNYLA	house mouse	IGKV8-21
L1-17-A	CDRL1	3OKK	A	KSSQSLLNSRTRKNYLA	house mouse	IGKV8-21
L1-17-A	CDRL1	1Q9L	C	KSSQSLLNSRTRKNYLA	house mouse	IGKV8-21
L1-17-A	CDRL1	43C9	A	KSSQSLLNISNQKNYLA	house mouse	IGKV8-24
L1-17-A	CDRL1	4M61	A	KSSQSVLYSSNQKNYLA	house mouse	IGKV8-27
L1-17-A	CDRL1	1HIL	C	TSSQSLFNSGKQKNYLT	house mouse	IGKV8-19
L1-17-A	CDRL1	3O2D	L	KSSQSLLYSTNQKNYLA	human	IGKV4-1
L1-17-A	CDRL1	4M61	C	KSSQSVLYSSNQKNYLA	house mouse	IGKV8-27
L1-17-A	CDRL1	3MBX	L	KSSQSLLYNNNQKNYLA	house mouse	IGKV8-30
L1-17-A	CDRL1	3Q3G	J	KSSQNLLYSSNQKNYLA	house mouse	IGKV8-30
L1-17-A	CDRL1	2R23	A	KSSQSLLNSRTRKNYLA	house mouse	IGKV8-21
L1-17-A	CDRL1	3MNW	A	KSSQSLLNSRNERNYLA	house mouse	IGKV8-24
L1-17-A	CDRL1	3IKC	C	KSSQSLLNSRTRKNYLA	house mouse	IGKV8-21
L1-17-A	CDRL1	4NM4	L	KSSQSVFSSSTNKNYLA	human	IGKV4-1
L1-17-A	CDRL1	3Q3G	A	KSSQNLLYSSNQKNYLA	house mouse	IGKV8-30
L1-17-A	CDRL1	3DUR	A	KSSQSLFKSRNQKNYLA	house mouse	IGKV8-21
L1-17-A	CDRL1	1LVE	A	KSSQSVLYSSNSKNYLA	human	IGKV4-1
L1-17-A	CDRL1	3HZV	A	KSSQSLLNSRTRKNYLA	house mouse	IGKV8-21
L1-17-A	CDRL1	1MCP	L	KSSQSLLNSGNQKNFLA	house mouse	IGKV8-28
L1-17-A	CDRL1	3IKC	A	KSSQSLLNSRTRKNYLA	house mouse	IGKV8-21
L1-17-A	CDRL1	3DUU	C	KSSQSLFKSRNQKNYLA	house mouse	IGKV8-21
L1-17-A	CDRL1	1T4K	A	RSSQSLFNSRTRKNYLA	house mouse	IGKV8-21
L1-17-A	CDRL1	1Q9O	A	KSSQSLLNSRTRKSYLA	house mouse	IGKV8-21
L1-17-A	CDRL1	1CK0	L	KSSQNLLHSITRKNYLA	house mouse	IGKV8-21
L1-17-A	CDRL1	3DV4	A	KSSQSLFKSRNQKNYLA	house mouse	IGKV8-21
L1-17-A	CDRL1	3I02	A	KSSQSLLNSRTRKNYLA	house mouse	IGKV8-21
L1-17-A	CDRL1	3NTC	L	KSSQSLLNSGDQKNYLT	house mouse	IGKV8-19
L1-17-A	CDRL1	43CA	A	KSSQSLLNISNQKNYLA	house mouse	IGKV8-24
L1-17-A	CDRL1	3UJT	M	KSSQSLLNSSNQKNYLA	house mouse	IGKV8-24
L1-17-A	CDRL1	4LEO	B	KSSQSVLNSGNQKNYLT	human	IGKV4-1
L1-17-A	CDRL1	1XF5	A	KSSQSLLNSRTRKNYLA	house mouse	IGKV8-21
L1-17-A	CDRL1	3IJS	C	KSSQSLLNSRTRKNYLA	house mouse	IGKV8-21
L1-17-A	CDRL1	1BBD	L	KSSQSLLNSRTQKNYLT	house mouse	IGKV8-19
L1-17-A	CDRL1	2R2E	A	KSSQSLLNSRTRKNYLA	house mouse	IGKV8-21
L1-17-A	CDRL1	2G5B	A	KSSQSLFNSKTRRNYLA	house mouse	IGKV8-21
L1-17-A	CDRL1	4NM4	M	KSSQSVFSSSTNKNYLA	human	IGKV4-1
L1-17-A	CDRL1	3OKE	A	KSSQSLLNSRTRKNYLA	house mouse	IGKV8-21
L1-17-A	CDRL1	3I02	C	KSSQSLLNSRTRKNYLA	house mouse	IGKV8-21
L1-17-A	CDRL1	1HIL	A	TSSQSLFNSGKQKNYLT	house mouse	IGKV8-19
L1-17-A	CDRL1	3IJH	C	KSSQSLLNSRTRKNYLA	house mouse	IGKV8-21
L1-17-A	CDRL1	3DUR	C	KSSQSLFKSRNQKNYLA	house mouse	IGKV8-21
L1-17-A	CDRL1	3HZK	A	KSSQSLLNSRTRKNYLA	house mouse	IGKV8-21
L1-17-A	CDRL1	4NX3	A	KSSQSLLNSNNQKNYLA	house mouse	IGKV8-24
L1-17-A	CDRL1	3SY0	A	KSSQSLLNSRTRKNYLA	house mouse	IGKV8-21
L1-17-A	CDRL1	4HGW	A	KSSQSLLNSRTRKNYLA	house mouse	IGKV8-21
L1-17-B	CDRL1	3LDB	B	RSSQSLYYSGIKKNLLA	Norway rat	IGKV8S6
L1-17-B	CDRL1	3LD8	B	RSSQSLYYSGIKKNLLA	Norway rat	IGKV8S6
L1-17-B	CDRL1	1FRG	L	KSSQSLFNSGKRKNFLT	house mouse	IGKV8-19
Unclustered	CDRL1	2FBJ	L	SASSSVSSLH	house mouse	IGKV4-86
Unclustered	CDRL1	1MCC	B	TGTSSDVGGYNYVS	human	IGLV2-8

Unclustered	CDRL1	1VPO	L	RSSEVIVTRNGYTPIE	house mouse	IGKV1-117
Unclustered	CDRL1	1MCS	B	TGTSSDVGGYNYVS	human	IGLV2-8
Unclustered	CDRL1	1MCF	B	TGTSSDVGGYNYVS	human	IGLV2-8
Unclustered	CDRL1	1TZH	L	RASQASYSSVA	house mouse	IGKV12-44
Unclustered	CDRL1	1MCI	B	TGTSSDVGGYNYVS	human	IGLV2-8
Unclustered	CDRL1	1LIL	A	SGEKLGDAYVC	human	IGLV3-1
Unclustered	CDRL1	3BJ9	1	TLRNDHDIGVYSVY	human	VPREB1
Unclustered	CDRL1	1A8J	H	TGTSSDVGGYNYVS	human	IGLV2-8
Unclustered	CDRL1	3TCL	L	RASQSVHSRYFA	human	IGKV3-20
Unclustered	CDRL1	1LIL	B	SGEKLGDAYVC	human	IGLV3-1
Unclustered	CDRL1	4K94	L	RASQSVSSAVA	human	IGKV1-39
Unclustered	CDRL1	3M8O	L	RSSQSLLRRDGHNDLE	human	IGKV2-28
Unclustered	CDRL1	4BJL	A	SGSSSNIGENSVT	human	IGLV1-44
Unclustered	CDRL1	1MCH	B	TGTSSDVGGYNYVS	human	IGLV2-8
Unclustered	CDRL1	1MCN	B	TGTSSDVGGYNYVS	human	IGLV2-8
Unclustered	CDRL1	3U7W	L	RTSQSGSLA	human	IGKV3-11
Unclustered	CDRL1	4JO2	L	QSSQNVYSNNNLA	rabbit	IGKV1S5
Unclustered	CDRL1	4HIE	A	RASQSVTNYLA	human	IGKV3-11
Unclustered	CDRL1	4LSU	L	TGASTSVA	human	IGLV2-14
Unclustered	CDRL1	3C5S	C	RSSKSLLHSDGITYLY	house mouse	IGKV2-109
Unclustered	CDRL1	2H3N	A	TLRNDHDIGVYSVY	human	VPREB1
Unclustered	CDRL1	1BLN	A	RSSQSIVHSTGNTYLE	house mouse	IGKV1-117
Unclustered	CDRL1	3MA9	L	SGDNIPYEYAS	human	IGLV3-1
Unclustered	CDRL1	4FAB	L	RSSQSLVHSQGNTYLR	house mouse	IGKV1-110
Unclustered	CDRL1	4HIJ	A	RASQSVTNYLA	human	IGKV3-11
Unclustered	CDRL1	2BFV	L	RSSQSLVSNNRRNYLH	house mouse	IGKV1-110
Unclustered	CDRL1	1CLZ	L	RSSQIIVHNNGNTYLE	house mouse	IGKV1-117
Unclustered	CDRL1	4JAM	L	SGASTNVC	human	IGLV3-1
Unclustered	CDRL1	4JO1	M	QSSQNVYSNNNLA	rabbit	IGKV1S5
Unclustered	CDRL1	4JPI	L	RASQSVSSYLA	human	IGKV3-11
Unclustered	CDRL1	4HKX	B	GGNNIGRKRVD	human	IGLV3-21
Unclustered	CDRL1	4JO2	M	QSSQNVYSNNNLA	rabbit	IGKV1S5
Unclustered	CDRL1	3GHE	L	TGTSTDVNGYNYVS	human	IGLV2-14
Unclustered	CDRL1	4JAM	B	SGASTNVC	human	IGLV3-1
Unclustered	CDRL1	3RPI	L	QANGYLN	human	IGKV1-33
Unclustered	CDRL1	1JVK	A	TGVSSIVGSYNLVS	human	IGLV2-23
Unclustered	CDRL1	1MCJ	B	TGTSSDVGGYNYVS	human	IGLV2-8
Unclustered	CDRL1	1MCE	A	TGTSSDVGGYNYVS	human	IGLV2-8
Unclustered	CDRL1	4G6F	L	RGDSLRSHYAS	human	IGLV3-19
Unclustered	CDRL1	1MCQ	B	TGTSSDVGGYNYVS	human	IGLV2-8
Unclustered	CDRL1	1CFV	L	RSSQSLVSNNRRNYLH	house mouse	IGKV1-110
Unclustered	CDRL1	1MCR	A	TGTSSDVGGYNYVS	human	IGLV2-8
Unclustered	CDRL1	1MCD	B	TGTSSDVGGYNYVS	human	IGLV2-8
Unclustered	CDRL1	1NAK	M	RSSQSLGHSSGNTYLH	house mouse	IGKV1-110
Unclustered	CDRL1	1LHZ	B	TGVSSIVGSYNLVS	human	IGLV2-23
Unclustered	CDRL1	2MCG	2	TGTSSDVGGYNYVS	human	IGLV2-8
Unclustered	CDRL1	1NAK	L	RSSQSLGHSSGNTYLH	house mouse	IGKV1-110
Unclustered	CDRL1	3QNZ	A	RSSQSLLRRDGHNDLE	human	IGKV2-28
Unclustered	CDRL1	4FZE	L	SGDNLRDGYAS	human	IGLV3-1
Unclustered	CDRL1	4G6F	D	RGDSLRSHYAS	human	IGLV3-19

Unclustered	CDRL1	4JO1	L	QSSQNVYSNNNLA	rabbit	IGKV1S5
Unclustered	CDRL1	1EK3	B	KSSQNLLDSSFDTNTLA	human	IGKV4-1
Unclustered	CDRL1	3BDY	L	RASQDIPRSISGYVA	human	IGKV1-39
Unclustered	CDRL1	3TCL	B	RASQSVHSRYFA	human	IGKV3-20
Unclustered	CDRL1	3KYM	O	RASQSVSSYLA	human	IGKV3-15
Unclustered	CDRL1	3SE9	L	TAASYGHMT	human	IGKV3-20
Unclustered	CDRL1	3FO9	A	RSSQSLVHSYGNTFLN	house mouse	IGKV1-110
Unclustered	CDRL1	4LSS	L	RTSQYGSLA	human	IGKV3-20
Unclustered	CDRL1	1CLY	L	RSSQIIVHNNGNTYLE	house mouse	IGKV1-117
Unclustered	CDRL1	3ULS	A	SGDNIGSYYVH	human	IGLV3-21
Unclustered	CDRL1	1BFV	L	RSSQSLVSNNRRNYLH	house mouse	IGKV1-110
Unclustered	CDRL1	1TZH	A	RASQASYSSVA	house mouse	IGKV12-44
Unclustered	CDRL1	1IGF	L	RSNQTILLSDGDTYLE	house mouse	IGKV1-117
Unclustered	CDRL1	1DCL	A	TGTSSNVGGYNYVS	human	IGLV2-8
Unclustered	CDRL1	1MCK	B	TGTSSDVGGYNYVS	human	IGLV2-8
Unclustered	CDRL1	1MCL	B	TGTSSDVGGYNYVS	human	IGLV2-8
Unclustered	CDRL1	3MCG	1	TGTSSDVGGYNYVS	human	IGLV2-8
Unclustered	CDRL1	4FQ2	L	GRQALGSRAVQ	human	IGLV3-21
Unclustered	CDRL1	3U1S	L	KCSHSLQHSTGANYLA	human	IGKV2-28
Unclustered	CDRL1	2H3N	C	TLRNDHDIGVYSVY	human	VPREB1
Unclustered	CDRL1	3QO0	A	RSSQSLLRRDGHNDLE	human	IGKV2-28
Unclustered	CDRL1	3QO1	A	RSSQSLLRRDGHNDLE	human	IGKV2-28
Unclustered	CDRL1	4HIH	A	RASQSVTNYLA	human	IGKV3-11
Unclustered	CDRL1	2GK0	A	RSSQSIVHSNGNTYLE	house mouse	IGKV1-117
Unclustered	CDRL1	1JVK	B	TGVSSIVGSYNLVS	human	IGLV2-23
Unclustered	CDRL1	1NSN	L	RASQSVSTSSFRYMH	house mouse	IGKV3-7
Unclustered	CDRL1	1H3P	L	RSSQSLLNTRTRKSYLA	house mouse	IGKV8-21
Unclustered	CDRL1	1LGV	B	TGVSSIVGSYNLVS	human	IGLV2-23
Unclustered	CDRL1	1UCB	L	RSSQIIVHNNGNTYLE	house mouse	IGKV1-117
Unclustered	CDRL1	1MCQ	A	TGTSSDVGGYNYVS	human	IGLV2-8
Unclustered	CDRL1	1KEM	L	RFSQSIVHSNGNTYLE	house mouse	IGKV1-117
Unclustered	CDRL1	1CBV	L	RSSQSLVHSNGNTYLH	house mouse	IGKV1-110
Unclustered	CDRL1	3C5S	A	RSSKSLLHSDGITYLY	house mouse	IGKV2-109
Unclustered	CDRL1	4JDV	B	RASQSVSSYLA	human	IGKV3-11
Unclustered	CDRL1	3MCG	2	TGTSSDVGGYNYVS	human	IGLV2-8
Unclustered	CDRL1	1AXT	L	RSSQSLVHSYGNTFLN	house mouse	IGKV1-110
Unclustered	CDRL1	1MCL	A	TGTSSDVGGYNYVS	human	IGLV2-8
Unclustered	CDRL1	3RPI	B	QANGYLN	human	IGKV1-33
Unclustered	CDRL1	2RHE	A	TGSATDIGSNSVI	human	IGLV1-36
Unclustered	CDRL1	4HIJ	C	RASQSVTNYLA	human	IGKV3-11
Unclustered	CDRL1	1LHZ	A	TGVSSIVGSYNLVS	human	IGLV2-23
Unclustered	CDRL1	1MCB	B	TGTSSDVGGYNYVS	human	IGLV2-8
Unclustered	CDRL1	3FO9	L	RSSQSLVHSYGNTFLN	house mouse	IGKV1-110
Unclustered	CDRL1	2Y6S	L	RASKSVSTSGYSYMH	house mouse	IGKV3-12
Unclustered	CDRL1	1MCR	B	TGTSSDVGGYNYVS	human	IGLV2-8
Unclustered	CDRL1	4JDV	L	RASQSVSSYLA	human	IGKV3-11
Unclustered	CDRL1	2MCG	1	TGTSSDVGGYNYVS	human	IGLV2-8
Unclustered	CDRL1	1MCE	B	TGTSSDVGGYNYVS	human	IGLV2-8
Unclustered	CDRL1	1NBV	L	RSSQSLVHSNGNTYLH	house mouse	IGKV1-110
Unclustered	CDRL1	3SE8	L	KASQGGNAMT	human	IGKV3-11

Unclustered	CDRL1	2Y6S	C	RASKSVSTSGYSYMH	house mouse	IGKV3-12
Unclustered	CDRL1	1BLN	C	RSSQSIVHSTGNTYLE	house mouse	IGKV1-117
L2-7-A	CDRL2	3QNZ	A	LGSTRAS	human	IGKV2-28
L2-7-A	CDRL2	4MSW	B	YASESIS	house mouse	IGKV5-48
L2-7-A	CDRL2	3FO9	A	KVSNRFS	house mouse	IGKV1-110
L2-7-A	CDRL2	2YMX	L	AATNLAD	house mouse	IGKV12-46
L2-7-A	CDRL2	2VXS	N	ANNQRPS	human	IGLV6-57
L2-7-A	CDRL2	3BJL	A	EDNSRAS	human	IGLV1-44
L2-7-A	CDRL2	2VXV	L	TASTRAT	human	IGKV3-15
L2-7-A	CDRL2	1I9J	L	KVSNRFS	house mouse	IGKV1-117
L2-7-A	CDRL2	3LDB	B	YASTLFT	Norway rat	IGKV8S6
L2-7-A	CDRL2	4HII	A	GASNRAT	human	IGKV3-11
L2-7-A	CDRL2	2BX5	B	AASSLQS	human	IGKV1-39
L2-7-A	CDRL2	4CAD	G	NAKTLAD	house mouse	IGKV12-41
L2-7-A	CDRL2	4NZT	L	SNDQRPS	human	IGLV1-44
L2-7-A	CDRL2	1CD0	B	EDNQRPS	human	IGLV6-57
L2-7-A	CDRL2	1CGS	L	RVSNRFS	house mouse	IGKV1-110
L2-7-A	CDRL2	2W0L	B	EDNQRPS	human	IGLV6-57
L2-7-A	CDRL2	4AMK	L	GTNNRAP	house mouse	IGLV1
L2-7-A	CDRL2	4NCC	L	STSNLAS	house mouse	IGKV4-57
L2-7-A	CDRL2	3DIF	C	SASYRYT	house mouse	IGKV6-17
L2-7-A	CDRL2	1UZ8	A	QMSNLAS	house mouse	IGKV2-109
L2-7-A	CDRL2	1TJI	L	DASSLES	human	IGKV1D-13
L2-7-A	CDRL2	1YMH	C	WASTRES	house mouse	IGKV8-21
L2-7-A	CDRL2	4JY5	L	NNNDRPS	human	IGLV3-21
L2-7-A	CDRL2	1EFQ	A	WASTRES	human	IGKV4-1
L2-7-A	CDRL2	4JRE	C	YTSTLES	house mouse	IGKV19-93
L2-7-A	CDRL2	2V7N	A	DSSSRAT	human	IGKV3-11
L2-7-A	CDRL2	4FQH	B	SNDQRPS	human	IGLV1-44
L2-7-A	CDRL2	1CIC	C	YTTTLAD	house mouse	IGKV12-41
L2-7-A	CDRL2	3UYR	L	KVSNRFS	house mouse	IGKV1-110
L2-7-A	CDRL2	3D9A	L	YASQSIS	house mouse	IGKV5-43
L2-7-A	CDRL2	1YY9	C	YASESIS	house mouse	IGKV5-48
L2-7-A	CDRL2	1OAZ	L	GTNNRAP	house mouse	IGLV1
L2-7-A	CDRL2	3SGE	L	LVSKLDS	house mouse	IGKV1-135
L2-7-A	CDRL2	3BDX	B	EDNQRPS	human	IGLV6-57
L2-7-A	CDRL2	3TNN	B	EVSERPS	human	IGLV2-23
L2-7-A	CDRL2	4JZO	F	QDNKRPS	human	IGLV3-1
L2-7-A	CDRL2	3GHB	L	GNNKRPS	human	IGLV1-51
L2-7-A	CDRL2	4I2X	A	SASYRYT	human	IGKV6-17
L2-7-A	CDRL2	2VXS	M	ANNQRPS	human	IGLV6-57
L2-7-A	CDRL2	1CR9	L	LVSKRDS	house mouse	IGKV1-135
L2-7-A	CDRL2	3EOA	A	SGSTLQS	human	IGKV1-16
L2-7-A	CDRL2	2ORB	L	AISNRGS	house mouse	IGKV3-2
L2-7-A	CDRL2	3CFJ	L	GTNKRAP	house mouse	IGLV1
L2-7-A	CDRL2	3CDF	C	DASNLET	human	IGKV1-33
L2-7-A	CDRL2	3SO3	B	GASTRAT	human	IGKV3-11
L2-7-A	CDRL2	1ZLU	K	KASTLKT	human	IGKV1-5
L2-7-A	CDRL2	3T0W	B	NDDRRSS	human	IGLV1-47
L2-7-A	CDRL2	2ZKH	L	STSNLAS	house mouse	IGKV4-57

L2-7-A	CDRL2	1ORS	A	STSNLAS	house mouse	IGKV4-74
L2-7-A	CDRL2	2H9G	L	SASFLYS	human	IGKV1-39
L2-7-A	CDRL2	3QNX	A	LGSTRAS	human	IGKV2-28
L2-7-A	CDRL2	4ERS	L	AASSLES	human	IGKV1-17
L2-7-A	CDRL2	1DL7	L	GTKHRTP	house mouse	IGLV1
L2-7-A	CDRL2	4GW5	C	YASESIS	house mouse	IGKV5-48
L2-7-A	CDRL2	3W9E	B	GASNRAT	house mouse	IGKV1-117
L2-7-A	CDRL2	4M43	L	YASESIS	house mouse	IGKV5-48
L2-7-A	CDRL2	1F4W	L	ATNNRAA	house mouse	IGLV1
L2-7-A	CDRL2	1QKZ	L	TVSNRFS	house mouse	IGKV1-110
L2-7-A	CDRL2	2B2X	L	LTSYLAS	house mouse	IGKV4-68
L2-7-A	CDRL2	3HC0	B	SASYRYS	human	IGKV1-16
L2-7-A	CDRL2	2HFF	L	SASFLYS	human	IGKV1-39
L2-7-A	CDRL2	1WC7	A	STSNLAS	house mouse	IGKV4-57
L2-7-A	CDRL2	2R2H	A	WASTRES	house mouse	IGKV8-21
L2-7-A	CDRL2	2A1W	M	KVSNRFS	house mouse	IGKV1-117
L2-7-A	CDRL2	3L7F	L	SGSTLQS	human	IGKV3-11
L2-7-A	CDRL2	3TNN	D	EVSERPS	human	IGLV2-23
L2-7-A	CDRL2	1MCD	A	EVNKRPS	human	IGLV2-8
L2-7-A	CDRL2	3KS0	J	GTNKRAP	house mouse	IGLV1
L2-7-A	CDRL2	3LS4	L	RTSNLAS	house mouse	IGKV4-91
L2-7-A	CDRL2	1U91	A	DASSLES	human	IGKV1D-13
L2-7-A	CDRL2	4K94	L	SASSLYS	human	IGKV1-39
L2-7-A	CDRL2	3C2A	L	GNNKRPS	human	IGLV1-51
L2-7-A	CDRL2	1TPX	C	LVSRLDS	house mouse	IGKV1-135
L2-7-A	CDRL2	1LIL	A	QDNRRPS	human	IGLV3-1
L2-7-A	CDRL2	3D85	A	YASQSIS	house mouse	IGKV5-39
L2-7-A	CDRL2	1DCL	B	EVNKRPS	human	IGLV2-8
L2-7-A	CDRL2	3SE8	L	DTSRRAS	human	IGKV3-11
L2-7-A	CDRL2	1A6V	M	GTNNRAP	house mouse	IGLV1
L2-7-A	CDRL2	2YC1	E	DASNRAT	human	IGKV3D-11
L2-7-A	CDRL2	1LO2	L	KVSNRFS	house mouse	IGKV1-117
L2-7-A	CDRL2	3CDF	D	DASNLET	human	IGKV1-33
L2-7-A	CDRL2	3IET	C	KVSNRFS	house mouse	IGKV1-110
L2-7-A	CDRL2	4AT6	D	GTNNRAP	house mouse	IGLV1
L2-7-A	CDRL2	1OAR	N	GTNNRAP	house mouse	IGLV1
L2-7-A	CDRL2	1YEG	L	LVSKLDS	house mouse	IGKV1-133
L2-7-A	CDRL2	1ZTX	L	WASTRHT	house mouse	IGKV6-25
L2-7-A	CDRL2	1KCS	L	GASNRYT	house mouse	IGKV6-20
L2-7-A	CDRL2	3C08	L	DTSNLAS	human	IGKV1-39
L2-7-A	CDRL2	4HFW	A	AATSLQS	human	IGKV1-6
L2-7-A	CDRL2	1YEI	L	LVSKLDS	house mouse	IGKV1-133
L2-7-A	CDRL2	2BX5	D	AASSLQS	human	IGKV1-39
L2-7-A	CDRL2	1CIC	A	WASTRHT	house mouse	IGKV6-23
L2-7-A	CDRL2	2E27	L	YTSTLKS	house mouse	IGKV19-93
L2-7-A	CDRL2	1Q9O	A	WAATRES	house mouse	IGKV8-21
L2-7-A	CDRL2	1F58	L	AASTLES	house mouse	IGKV3-4
L2-7-A	CDRL2	1BRE	D	DASTLET	human	IGKV1-33
L2-7-A	CDRL2	1NCA	L	WASTRHI	house mouse	IGKV6-25
L2-7-A	CDRL2	3IFO	B	KVSNRFS	house mouse	IGKV1-117

L2-7-A	CDRL2	1MCQ	A	EVNKRPS	human	IGLV2-8
L2-7-A	CDRL2	1MJJ	L	RMSNLAS	house mouse	IGKV2-137
L2-7-A	CDRL2	4HK0	B	DDSDRPS	human	IGLV3-21
L2-7-A	CDRL2	4F33	G	DTSKLAS	house mouse	IGKV4-59
L2-7-A	CDRL2	3ZKM	L	LASNLES	house mouse	IGKV3-10
L2-7-A	CDRL2	3IF1	C	KVSNRFS	house mouse	IGKV1-110
L2-7-A	CDRL2	2JB5	L	GGSNRPS	human	IGLV2-23
L2-7-A	CDRL2	3L95	L	SASFLYS	human	IGKV1-39
L2-7-A	CDRL2	3MOD	L	DASSLES	human	IGKV1-13
L2-7-A	CDRL2	3FO1	A	KVSNRFS	house mouse	IGKV1-117
L2-7-A	CDRL2	2R56	L	AASSLQS	human	IGKV1D-16
L2-7-A	CDRL2	1BFV	L	KVSNRFS	house mouse	IGKV1-110
L2-7-A	CDRL2	2BX5	N	AASSLQS	human	IGKV1-39
L2-7-A	CDRL2	1MIM	L	DTSKLAS	human	IGKV1-5
L2-7-A	CDRL2	2NY6	C	GASTRAT	human	IGKV3-15
L2-7-A	CDRL2	1FNS	L	YTSSLHS	house mouse	IGKV10-94
L2-7-A	CDRL2	3IF1	A	KVSNRFS	house mouse	IGKV1-110
L2-7-A	CDRL2	2F5A	L	DASSLES	human	IGKV1D-13
L2-7-A	CDRL2	4AT6	N	GTNNRAP	house mouse	IGLV1
L2-7-A	CDRL2	2IGF	L	KVSNRFS	house mouse	IGKV1-117
L2-7-A	CDRL2	2R2B	A	WASTRES	house mouse	IGKV8-21
L2-7-A	CDRL2	2IPU	K	KVSNRFS	house mouse	IGKV1-117
L2-7-A	CDRL2	3HC4	L	SASYRYS	human	IGKV1-16
L2-7-A	CDRL2	3PHO	A	LASNLES	house mouse	IGKV3-12
L2-7-A	CDRL2	4LLU	D	SASYRYT	human	IGKV1-16
L2-7-A	CDRL2	1TQC	C	LVSRLDS	house mouse	IGKV1-135
L2-7-A	CDRL2	3GRW	L	SASFLYS	human	IGKV1-39
L2-7-A	CDRL2	1I9I	L	KVSNRFS	house mouse	IGKV1-117
L2-7-A	CDRL2	3V0V	B	KASNLHT	house mouse	IGKV11-125
L2-7-A	CDRL2	1OAR	L	GTNNRAP	house mouse	IGLV1
L2-7-A	CDRL2	4G6A	L	LASNLNS	house mouse	IGKV3
L2-7-A	CDRL2	1MCN	A	EVNKRPS	human	IGLV2-8
L2-7-A	CDRL2	2AJY	L	LMSTRAS	house mouse	IGKV2-112
L2-7-A	CDRL2	2OZ4	L	YASESIS	house mouse	IGKV5-48
L2-7-A	CDRL2	3GI8	L	FASTRES	house mouse	IGKV8-24
L2-7-A	CDRL2	2HKF	L	KVSNRFS	house mouse	IGKV1-110
L2-7-A	CDRL2	4K3H	C	ETDKKYS	human	IGLV7-46
L2-7-A	CDRL2	2VXQ	L	AASSLQS	human	IGKV1-6
L2-7-A	CDRL2	4LF3	D	AASSLES	house mouse	IGKV11-125
L2-7-A	CDRL2	1FN4	A	NTNSLQT	Norway rat	IGKV22S2
L2-7-A	CDRL2	3MLR	L	QDFKRPS	human	IGLV3-1
L2-7-A	CDRL2	3ZKN	D	LASNLES	house mouse	IGKV3-10
L2-7-A	CDRL2	3Q6G	L	ADDERSS	human	IGLV3-21
L2-7-A	CDRL2	1LO3	L	KVSNRFS	house mouse	IGKV1-117
L2-7-A	CDRL2	2AJZ	A	LMSTRAS	house mouse	IGKV2-112
L2-7-A	CDRL2	3CFK	E	GTNKRAP	house mouse	IGLV1
L2-7-A	CDRL2	4K3D	M	GDTSRAS	sheep	IGLV1S8
L2-7-A	CDRL2	1ETZ	L	GTNNRVP	house mouse	IGLV1
L2-7-A	CDRL2	4D9L	O	GNSNRPS	human	IGLV1-40
L2-7-A	CDRL2	3DRQ	A	DASSLES	human	IGKV1D-13

L2-7-A	CDRL2	3EYS	L	KVSNRFS	house mouse	IGKV1-117
L2-7-A	CDRL2	4F3F	A	DTSKLAS	house mouse	IGKV4-59
L2-7-A	CDRL2	1N64	L	WASTRES	house mouse	IGKV8-21
L2-7-A	CDRL2	3LEX	L	LVSKLAS	house mouse	IGKV1-135
L2-7-A	CDRL2	1KN2	L	LVSKLDS	house mouse	IGKV1-133
L2-7-A	CDRL2	1IBG	L	LASILES	house mouse	IGKV3-12
L2-7-A	CDRL2	4JHA	L	VASNLET	human	IGKV1-33
L2-7-A	CDRL2	1RU9	L	LGTKLDS	house mouse	IGKV1-133
L2-7-A	CDRL2	1GAF	L	AASTLHS	house mouse	IGKV9-124
L2-7-A	CDRL2	1J1O	L	FASQSIS	house mouse	IGKV5-43
L2-7-A	CDRL2	3DUR	A	WASTRES	house mouse	IGKV8-21
L2-7-A	CDRL2	2FR4	A	NAKTLAE	house mouse	IGKV12-44
L2-7-A	CDRL2	1GIG	L	GTNNRAP	house mouse	IGLV1
L2-7-A	CDRL2	3ULS	A	EDSERPS	human	IGLV3-21
L2-7-A	CDRL2	3UC0	L	DASTLES	human	IGKV1-13
L2-7-A	CDRL2	3RVU	C	RANRLIT	house mouse	IGKV14-111
L2-7-A	CDRL2	2UZI	L	SASVLQS	human	IGKV1-39
L2-7-A	CDRL2	3T3M	L	YGTNLVD	house mouse	IGKV14-100
L2-7-A	CDRL2	1A4K	L	KVSNRFS	house mouse	IGKV1-110
L2-7-A	CDRL2	1MCF	A	EVNKRPS	human	IGLV2-8
L2-7-A	CDRL2	1D5B	A	RTNRLVD	house mouse	IGKV14-111
L2-7-A	CDRL2	1HEZ	C	AASSLQS	human	IGKV1-39
L2-7-A	CDRL2	1Q9K	A	WASTRES	house mouse	IGKV8-21
L2-7-A	CDRL2	3UPA	B	DADDLQS	human	IGKV1-39
L2-7-A	CDRL2	4NPY	L	DDSDRPS	human	IGLV3-21
L2-7-A	CDRL2	1Q9W	A	WAATRES	house mouse	IGKV8-21
L2-7-A	CDRL2	3FCT	C	SASNRYT	house mouse	IGKV6-13
L2-7-A	CDRL2	1Q1J	M	GNNKRPS	human	IGLV1-51
L2-7-A	CDRL2	1B0W	C	DASTLET	human	IGKV1-33
L2-7-A	CDRL2	2YC1	B	DASNRAT	human	IGKV3D-11
L2-7-A	CDRL2	1WCB	L	STSNLAS	house mouse	IGKV4-57
L2-7-A	CDRL2	1SY6	L	DTSKLAS	house mouse	IGKV4-59
L2-7-A	CDRL2	1OP3	K	KASTLKT	human	IGKV1-5
L2-7-A	CDRL2	2BMK	L	STSNLAS	house mouse	IGKV4-57
L2-7-A	CDRL2	1Y18	L	GTNKRAP	house mouse	IGLV1
L2-7-A	CDRL2	3FZU	L	VASSLQS	human	IGKV1-39
L2-7-A	CDRL2	3C5S	A	HLSNLAS	house mouse	IGKV2-109
L2-7-A	CDRL2	1BVK	D	YTTTLAD	human	IGKV1-27
L2-7-A	CDRL2	4N9G	N	YTSHLES	human	IGKV1-16
L2-7-A	CDRL2	1MJ7	L	RMSNLAS	house mouse	IGKV2-137
L2-7-A	CDRL2	1FGN	L	YATSLAD	house mouse	IGKV14-126
L2-7-A	CDRL2	4F33	E	DTSKLAS	house mouse	IGKV4-59
L2-7-A	CDRL2	1REI	B	EASNLQA	human	IGKV1-33
L2-7-A	CDRL2	1IGT	C	KASNLHT	house mouse	IGKV10-94
L2-7-A	CDRL2	1F8T	L	LVSKLDS	house mouse	IGKV1-133
L2-7-A	CDRL2	4ALA	L	LASNRHT	house mouse	IGKV6-14
L2-7-A	CDRL2	1N7M	H	SASNRYT	house mouse	IGKV6-13
L2-7-A	CDRL2	1E4X	M	YTSTLHS	house mouse	IGKV10-96
L2-7-A	CDRL2	1Y0L	C	GTNKRAP	house mouse	IGLV1
L2-7-A	CDRL2	1YEJ	L	LVSKLDS	house mouse	IGKV1-133

L2-7-A	CDRL2	3C6S	E	HLSNLAS	house mouse	IGKV2-109
L2-7-A	CDRL2	1V7M	M	STSNLAS	house mouse	IGKV4-57
L2-7-A	CDRL2	2HKH	L	KVSNRFS	house mouse	IGKV1-110
L2-7-A	CDRL2	3MCL	L	DSSRLAS	house mouse	IGKV4-59
L2-7-A	CDRL2	1QAC	A	WASTRES	human	IGKV4-1
L2-7-A	CDRL2	4LSS	L	SGSTRAA	human	IGKV3-20
L2-7-A	CDRL2	1CE1	L	NTNNLQT	human	IGKV1-33
L2-7-A	CDRL2	2FJF	C	SASFLYS	human	IGKV1-39
L2-7-A	CDRL2	1XGY	M	RMSNLAS	house mouse	IGKV2-109
L2-7-A	CDRL2	3CFC	L	QMSNLAS	house mouse	IGKV2-109
L2-7-A	CDRL2	3U4E	B	DVSKRPS	human	IGLV2-14
L2-7-A	CDRL2	3DSF	L	AATNLAD	house mouse	IGKV12-46
L2-7-A	CDRL2	1OAY	N	GTNNRAP	house mouse	IGLV1
L2-7-A	CDRL2	3NH7	M	DDSNRPS	human	IGLV3-1
L2-7-A	CDRL2	1EAP	A	YTSTLLP	house mouse	IGKV19-93
L2-7-A	CDRL2	2XZQ	L	GTNNRAP	house mouse	IGLV1
L2-7-A	CDRL2	3P0Y	L	SASFLYS	human	IGKV1-5
L2-7-A	CDRL2	3EYQ	C	DASNRAT	human	IGKV3-11
L2-7-A	CDRL2	2G5B	C	WASTRES	house mouse	IGKV8-21
L2-7-A	CDRL2	4NRX	L	AASTLQS	human	IGKV1-39
L2-7-A	CDRL2	4J1U	E	WASTRES	house mouse	IGKV8-21
L2-7-A	CDRL2	2I5Y	L	GASTRAT	human	IGKV3-15
L2-7-A	CDRL2	4K3H	D	ETDKKYS	human	IGLV7-46
L2-7-A	CDRL2	3CDF	A	DASNLET	human	IGKV1-33
L2-7-A	CDRL2	1OAX	O	GTNNRAP	house mouse	IGLV1
L2-7-A	CDRL2	1MCK	B	EVNKRPS	human	IGLV2-8
L2-7-A	CDRL2	3O0R	L	SGSTLQF	house mouse	IGKV16-104
L2-7-A	CDRL2	1A7Q	L	YTTTLAA	house mouse	IGKV12-41
L2-7-A	CDRL2	2IQA	L	KVSNRFS	house mouse	IGKV1-117
L2-7-A	CDRL2	3U0T	A	QISRLDP	house mouse	IGKV
L2-7-A	CDRL2	1RZ7	L	AASTLQS	human	IGKV1-12
L2-7-A	CDRL2	1C5D	A	YTSILVL	Norway rat	IGKV19S2
L2-7-A	CDRL2	1NCB	L	WASTRHI	house mouse	IGKV6-25
L2-7-A	CDRL2	3HI5	L	AASSLQS	human	IGKV1-39
L2-7-A	CDRL2	1CLY	L	KVSNRFS	house mouse	IGKV1-117
L2-7-A	CDRL2	1NMC	L	YTSNLHS	house mouse	IGKV10-96
L2-7-A	CDRL2	2AK1	L	LMSTRAS	house mouse	IGKV2-112
L2-7-A	CDRL2	3OKM	A	WASTRES	house mouse	IGKV8-21
L2-7-A	CDRL2	4HH9	C	DASNRAT	human	IGKV3-11
L2-7-A	CDRL2	1ZLV	K	KASTLKT	human	IGKV1-5
L2-7-A	CDRL2	1RUP	L	LGTKLDS	house mouse	IGKV1-133
L2-7-A	CDRL2	2R0W	L	KVSNRFS	house mouse	IGKV1-117
L2-7-A	CDRL2	4K3H	F	ETDKKYS	human	IGLV7-46
L2-7-A	CDRL2	3B2U	L	DASNRAT	human	IGKV3D-11
L2-7-A	CDRL2	1K4D	B	YASESIS	house mouse	IGKV5-48
L2-7-A	CDRL2	3OAZ	L	KASTLKT	human	IGKV1-5
L2-7-A	CDRL2	4NRZ	L	GALNLQS	human	IGKV1-39
L2-7-A	CDRL2	1KEL	L	KVSNRFS	house mouse	IGKV1-117
L2-7-A	CDRL2	1XGP	A	YASQSIS	house mouse	IGKV5-43
L2-7-A	CDRL2	1L7I	L	SASYRYT	human	IGKV1-16

L2-7-A	CDRL2	1PLG	L	RVSNRFS	house mouse	IGKV1-110
L2-7-A	CDRL2	3MLV	M	QDFKRPS	human	IGLV3-1
L2-7-A	CDRL2	1IGM	L	DASNLET	human	IGKV1-33
L2-7-A	CDRL2	3A6C	L	YASQSIS	house mouse	IGKV5-43
L2-7-A	CDRL2	4J8R	A	WASTRHT	house mouse	IGKV6-23
L2-7-A	CDRL2	3NAC	L	AASSLQS	human	IGKV1-39
L2-7-A	CDRL2	2W0L	C	EDNQRPS	human	IGLV6-57
L2-7-A	CDRL2	1B0W	A	DASTLET	human	IGKV1-33
L2-7-A	CDRL2	4HS6	A	LASNLNS	house mouse	IGKV3
L2-7-A	CDRL2	3FCT	A	SASNRYT	house mouse	IGKV6-13
L2-7-A	CDRL2	3SQO	L	SASYRYT	human	IGKV1D-13
L2-7-A	CDRL2	2CJU	L	KVSNRFS	house mouse	IGKV1-117
L2-7-A	CDRL2	1TET	L	KVSNRFS	house mouse	IGKV1-117
L2-7-A	CDRL2	2OK0	L	KVSNRFS	house mouse	IGKV1-117
L2-7-A	CDRL2	1A4J	A	KVSNRFS	house mouse	IGKV1-110
L2-7-A	CDRL2	4NX3	A	FASTRES	house mouse	IGKV8-24
L2-7-A	CDRL2	3LRH	G	DDDLLAP	human	IGLV1-36
L2-7-A	CDRL2	4HPY	L	EDSKRPS	human	IGLV3-10
L2-7-A	CDRL2	1NGZ	A	SASNRYT	house mouse	IGKV6-13
L2-7-A	CDRL2	2G60	L	KVANRFS	house mouse	IGKV1-117
L2-7-A	CDRL2	1MCB	A	EVNKRPS	human	IGLV2-8
L2-7-A	CDRL2	1YJD	L	KASNLHT	house mouse	IGKV10-94
L2-7-A	CDRL2	3HC0	L	SASYRYS	human	IGKV1-16
L2-7-A	CDRL2	3Q3G	J	WASTRES	house mouse	IGKV8-30
L2-7-A	CDRL2	4GW4	B	DGSKLER	human	IGKV1-33
L2-7-A	CDRL2	3B2U	X	DASNRAT	human	IGKV3D-11
L2-7-A	CDRL2	3BPC	A	WASTRES	house mouse	IGKV8-21
L2-7-A	CDRL2	3U1S	L	LATHRAS	human	IGKV2-28
L2-7-A	CDRL2	1MLB	A	YVSQSSS	house mouse	IGKV5-43
L2-7-A	CDRL2	3SE9	L	ATSKRAS	human	IGKV3-20
L2-7-A	CDRL2	3MNW	A	FASIRES	house mouse	IGKV8-24
L2-7-A	CDRL2	2W0L	A	EDNQRPS	human	IGLV6-57
L2-7-A	CDRL2	2I5Y	Q	GASTRAT	human	IGKV3-15
L2-7-A	CDRL2	3G5Y	A	HGTNLED	house mouse	IGKV14-100
L2-7-A	CDRL2	1PZ5	A	KVSNRFS	house mouse	IGKV1-110
L2-7-A	CDRL2	4KI5	D	DTSNLAS	house mouse	IGKV4-55
L2-7-A	CDRL2	1JVK	A	EVNKRPS	human	IGLV2-23
L2-7-A	CDRL2	1A0Q	L	YTSTLLP	house mouse	IGKV19-93
L2-7-A	CDRL2	4IMK	D	DDSDRPS	human	IGLV3-21
L2-7-A	CDRL2	1DQJ	A	YASQSIS	house mouse	IGKV5-43
L2-7-A	CDRL2	2VXT	L	ATSSLDS	house mouse	IGKV9-120
L2-7-A	CDRL2	1R0A	L	YTSSLHS	house mouse	IGKV10-94
L2-7-A	CDRL2	4CAD	D	NAKTLAD	house mouse	IGKV12-41
L2-7-A	CDRL2	4HIH	A	GASNRAT	human	IGKV3-11
L2-7-A	CDRL2	3CMO	X	YTSRLQS	house mouse	IGKV10-94
L2-7-A	CDRL2	3IDN	A	DASSLES	human	IGKV1-13
L2-7-A	CDRL2	4AJ0	B	QDSKRPS	human	IGLV3-1
L2-7-A	CDRL2	1MCI	B	EVNKRPS	human	IGLV2-8
L2-7-A	CDRL2	4F33	A	DTSKLAS	house mouse	IGKV4-59
L2-7-A	CDRL2	2V17	L	SGSTLQS	house mouse	IGKV16-104

L2-7-A	CDRL2	1RUK	L	LGTKLDS	house mouse	IGKV1-133
L2-7-A	CDRL2	2XA8	L	AASYLES	house mouse	IGKV3
L2-7-A	CDRL2	2R1Y	A	WASTRES	house mouse	IGKV8-21
L2-7-A	CDRL2	2VDP	L	YGTNLVD	house mouse	IGKV14-100
L2-7-A	CDRL2	2H9G	A	SASFLYS	human	IGKV1-39
L2-7-A	CDRL2	4M61	A	WASTRES	house mouse	IGKV8-27
L2-7-A	CDRL2	3CDF	B	DASNLET	human	IGKV1-33
L2-7-A	CDRL2	3RVT	C	RANRLIT	house mouse	IGKV14-111
L2-7-A	CDRL2	3MAC	L	GDNNRPS	human	IGLV3-1
L2-7-A	CDRL2	1UYW	N	GASNRYT	house mouse	IGKV6-20
L2-7-A	CDRL2	3G5X	A	HGTNLDD	house mouse	IGKV14-100
L2-7-A	CDRL2	1HIL	A	WASTRES	house mouse	IGKV8-19
L2-7-A	CDRL2	4KPH	L	TTSNLAS	house mouse	IGKV4-80
L2-7-A	CDRL2	4J6R	L	GASTRAS	human	IGKV3D-7
L2-7-A	CDRL2	2B2X	M	LTSYLAS	house mouse	IGKV4-68
L2-7-A	CDRL2	1Q9W	C	WAATRES	house mouse	IGKV8-21
L2-7-A	CDRL2	5LVE	A	WASTRES	human	IGKV4-1
L2-7-A	CDRL2	1N0X	L	GVSNRAS	human	IGKV3-20
L2-7-A	CDRL2	3TNN	L	EVSERPS	human	IGLV2-23
L2-7-A	CDRL2	1T3F	A	GASNRYT	human	IGKV1-5
L2-7-A	CDRL2	3CFK	G	GTNKRAP	house mouse	IGLV1
L2-7-A	CDRL2	3QEH	B	LGSNRAT	house mouse	IGKV
L2-7-A	CDRL2	2B0S	L	RNDHRSS	human	IGLV1-47
L2-7-A	CDRL2	3M8O	L	LGSTRAS	human	IGKV2-28
L2-7-A	CDRL2	3EO0	A	GASSRAP	human	IGKV3-20
L2-7-A	CDRL2	2NY0	C	GASTRAT	human	IGKV3-15
L2-7-A	CDRL2	1RUR	L	QMSKLAS	house mouse	IGKV2-109
L2-7-A	CDRL2	4DKF	L	GASSRAS	human	IGKV1-39
L2-7-A	CDRL2	2IPU	L	KVSNRFS	house mouse	IGKV1-117
L2-7-A	CDRL2	1XGQ	A	YASQSIS	house mouse	IGKV5-43
L2-7-A	CDRL2	1MAM	L	YTSRLHS	house mouse	IGKV10-96
L2-7-A	CDRL2	1C5D	L	YTSILVL	Norway rat	IGKV19S2
L2-7-A	CDRL2	1ND0	E	LGTKLDS	house mouse	IGKV1-133
L2-7-A	CDRL2	3KYM	E	DASNRAT	human	IGKV3-15
L2-7-A	CDRL2	1A6T	A	STSNLAS	house mouse	IGKV4-72
L2-7-A	CDRL2	3V52	L	KVSNRFS	house mouse	IGKV1-110
L2-7-A	CDRL2	1S3K	L	KVSNRFS	house mouse	IGKV
L2-7-A	CDRL2	2C1O	A	LVSKLDS	house mouse	IGKV1-133
L2-7-A	CDRL2	1YEC	L	LVSKLDS	house mouse	IGKV1-133
L2-7-A	CDRL2	2G5B	G	WASTRES	house mouse	IGKV8-21
L2-7-A	CDRL2	1A4K	A	KVSNRFS	house mouse	IGKV1-110
L2-7-A	CDRL2	2V7N	C	DSSSRAT	human	IGKV3-11
L2-7-A	CDRL2	2VQ1	A	KVSNRFS	house mouse	IGKV1-110
L2-7-A	CDRL2	4LAR	L	STSNLAS	house mouse	IGKV4-79
L2-7-A	CDRL2	2YBR	B	DASNRAT	human	IGKV3D-11
L2-7-A	CDRL2	1NCC	L	WASTRHI	house mouse	IGKV6-25
L2-7-A	CDRL2	4AT6	B	GTNNRAP	house mouse	IGLV1
L2-7-A	CDRL2	3QG7	L	KLSSRFS	house mouse	IGKV1-110
L2-7-A	CDRL2	1F3D	L	KVSKRFS	house mouse	IGKV1-117
L2-7-A	CDRL2	2BX5	K	AASSLQS	human	IGKV1-39

L2-7-A	CDRL2	2AJ3	A	SASTLQS	human	IGKV1-9
L2-7-A	CDRL2	4J1U	C	WASTRES	house mouse	IGKV8-21
L2-7-A	CDRL2	4FQQ	C	DDSDRPS	human	IGLV3-21
L2-7-A	CDRL2	3OZ9	L	STSNLAS	house mouse	IGKV4-80
L2-7-A	CDRL2	4JZN	B	DDSDRPS	human	IGLV3-21
L2-7-A	CDRL2	3OJD	A	NASTLAD	house mouse	IGKV12-41
L2-7-A	CDRL2	4BJL	B	EDNSRAS	human	IGLV1-44
L2-7-A	CDRL2	3UC0	M	DASTLES	human	IGKV1-13
L2-7-A	CDRL2	2DWE	B	YASESIS	house mouse	IGKV5-48
L2-7-A	CDRL2	3EO0	C	GASSRAP	human	IGKV3-20
L2-7-A	CDRL2	3IDI	A	DASSLES	human	IGKV1-13
L2-7-A	CDRL2	3IU4	L	SASYRYT	house mouse	IGKV6-17
L2-7-A	CDRL2	3EYO	C	DASIRAT	human	IGKV3-11
L2-7-A	CDRL2	4DN3	L	DASSRAT	human	IGKV3-11
L2-7-A	CDRL2	3HNS	L	YTSRLHS	house mouse	IGKV10-96
L2-7-A	CDRL2	1R3J	A	YASESIS	house mouse	IGKV5-48
L2-7-A	CDRL2	3IJS	C	WASTRES	house mouse	IGKV8-21
L2-7-A	CDRL2	1Y18	A	GTNKRAP	house mouse	IGLV1
L2-7-A	CDRL2	3SKJ	L	KASNLHT	human	IGKV1-5
L2-7-A	CDRL2	2R2E	A	WASTRES	house mouse	IGKV8-21
L2-7-A	CDRL2	1NGX	A	SASNRYT	house mouse	IGKV6-13
L2-7-A	CDRL2	1PG7	L	YATSLAE	human	IGKV1-39
L2-7-A	CDRL2	2VDR	L	YGTNLVD	house mouse	IGKV14-100
L2-7-A	CDRL2	4HJG	A	SASFLYS	human	IGKV1-39
L2-7-A	CDRL2	8FAB	A	KDTQRPS	human	IGLV3-25
L2-7-A	CDRL2	3FO2	A	KVSNRFS	house mouse	IGKV1-117
L2-7-A	CDRL2	3S35	L	RASNLES	house mouse	IGKV3-5
L2-7-A	CDRL2	2OMN	A	EVTNRPS	human	IGLV2-14
L2-7-A	CDRL2	1CFT	A	RANRLMI	house mouse	IGKV14-111
L2-7-A	CDRL2	1LO4	L	KVSNRLS	house mouse	IGKV1-117
L2-7-A	CDRL2	4K3D	L	GDTSRAS	sheep	IGLV1S8
L2-7-A	CDRL2	1DQQ	A	YASQSIS	house mouse	IGKV5-43
L2-7-A	CDRL2	4M5Y	M	DDSDRPS	human	IGLV3-21
L2-7-A	CDRL2	3GBM	M	DNNKRPS	human	IGLV1-51
L2-7-A	CDRL2	2Q1E	B	DASTLET	human	IGKV1-33
L2-7-A	CDRL2	2P8P	A	DASSLES	human	IGKV1D-13
L2-7-A	CDRL2	1OAY	L	GTNNRAP	house mouse	IGLV1
L2-7-A	CDRL2	2HVJ	B	YASESIS	house mouse	IGKV5-48
L2-7-A	CDRL2	3IJS	A	WASTRES	house mouse	IGKV8-21
L2-7-A	CDRL2	4DGY	L	DASNRAT	human	IGKV3-11
L2-7-A	CDRL2	1VFA	A	YTTTLAD	house mouse	IGKV12-41
L2-7-A	CDRL2	3EYO	A	DASIRAT	human	IGKV3-11
L2-7-A	CDRL2	4HKZ	A	SASFLYS	human	IGKV1-39
L2-7-A	CDRL2	3DET	F	DTSKLTS	house mouse	IGKV4-59
L2-7-A	CDRL2	2ITD	B	YASESIS	house mouse	IGKV5-48
L2-7-A	CDRL2	1LK3	M	KASNLAS	Norway rat	IGKV3S11
L2-7-A	CDRL2	1Y0L	E	GTNKRAP	house mouse	IGLV1
L2-7-A	CDRL2	43C9	C	FASTRES	house mouse	IGKV8-24
L2-7-A	CDRL2	3C5S	C	HLSNLAS	house mouse	IGKV2-109
L2-7-A	CDRL2	1RZF	L	ENNERPS	human	IGLV1-51

L2-7-A	CDRL2	3EYV	A	KVSNRFS	house mouse	IGKV1-117
L2-7-A	CDRL2	1HI6	A	RANRLMI	house mouse	IGKV14-111
L2-7-A	CDRL2	3O6M	L	LVSKLDS	house mouse	IGKV1-135
L2-7-A	CDRL2	4AIZ	B	KDSERPS	human	IGLV3-25
L2-7-A	CDRL2	2Y6S	C	LVSNLES	house mouse	IGKV3-12
L2-7-A	CDRL2	4HFW	L	AATSLQS	human	IGKV1-6
L2-7-A	CDRL2	3B5G	B	EDNQRPS	human	IGLV6-57
L2-7-A	CDRL2	2NXZ	C	GASTRAT	human	IGKV3-15
L2-7-A	CDRL2	4LLV	B	GASSRPS	human	IGKV3-20
L2-7-A	CDRL2	1RMF	L	KVSNRFS	house mouse	IGKV1-110
L2-7-A	CDRL2	4JO4	L	KASTLAS	rabbit	IGKV1S3
L2-7-A	CDRL2	4FQL	L	GASTRAT	human	IGKV3-20
L2-7-A	CDRL2	4GAY	L	LASNLNS	house mouse	IGKV3-10
L2-7-A	CDRL2	3HZV	A	WASTRES	house mouse	IGKV8-21
L2-7-A	CDRL2	1AXS	A	RTNRLVD	house mouse	IGKV14-111
L2-7-A	CDRL2	4F37	K	KVSKRFS	house mouse	IGKV1-117
L2-7-A	CDRL2	3R06	E	YTNKLAD	house mouse	IGKV14-111
L2-7-A	CDRL2	4JZO	H	QDNKRPS	human	IGLV3-1
L2-7-A	CDRL2	2VDO	L	YGTNLVD	house mouse	IGKV14-100
L2-7-A	CDRL2	1FJ1	A	YTSTLQP	house mouse	IGKV19-93
L2-7-A	CDRL2	4JY6	C	NNQDRPA	human	IGLV3-21
L2-7-A	CDRL2	3HR5	A	KVSNRFS	house mouse	IGKV
L2-7-A	CDRL2	4N9G	L	YTSHLES	human	IGKV1-16
L2-7-A	CDRL2	1IGJ	C	KVSNRFS	house mouse	IGKV1-110
L2-7-A	CDRL2	1I7Z	C	LASNLAS	house mouse	IGKV3-12
L2-7-A	CDRL2	3CX5	K	YTSRLHA	house mouse	IGKV10-94
L2-7-A	CDRL2	3UJT	L	FASTRES	house mouse	IGKV8-24
L2-7-A	CDRL2	1BRE	C	DASTLET	human	IGKV1-33
L2-7-A	CDRL2	4G5Z	L	YASQSFS	human	IGKV1-39
L2-7-A	CDRL2	3H3P	M	GASSRPS	human	IGKV3-20
L2-7-A	CDRL2	1OAX	N	GTNNRAP	house mouse	IGLV1
L2-7-A	CDRL2	1U95	A	DASSLES	human	IGKV1D-13
L2-7-A	CDRL2	4NM4	M	WSSTRES	human	IGKV4-1
L2-7-A	CDRL2	3IDG	A	DASSLES	human	IGKV1-13
L2-7-A	CDRL2	2Y36	L	GTNNRAP	house mouse	IGLV1
L2-7-A	CDRL2	1F6L	L	NAKTLAE	human	IGKV1-39
L2-7-A	CDRL2	3MCG	1	EVNKRPS	human	IGLV2-8
L2-7-A	CDRL2	3GGW	A	HLSNLAS	house mouse	IGKV2-109
L2-7-A	CDRL2	1MFC	L	DTNNRAP	house mouse	IGLV1
L2-7-A	CDRL2	3C2A	M	GNNKRPS	human	IGLV1-51
L2-7-A	CDRL2	1YEF	L	LVSKLDS	house mouse	IGKV1-133
L2-7-A	CDRL2	1IFH	L	WASTRES	house mouse	IGKV8-19
L2-7-A	CDRL2	2G2R	L	LVSKLDS	house mouse	IGKV1-133
L2-7-A	CDRL2	2FX7	L	GASSRPS	human	IGKV3-20
L2-7-A	CDRL2	2BOB	B	YASESIS	house mouse	IGKV5-48
L2-7-A	CDRL2	4HXA	L	DTSNLAS	house mouse	IGKV4-63
L2-7-A	CDRL2	1IVL	A	YASQSIS	house mouse	IGKV5-43
L2-7-A	CDRL2	3NA9	L	AASSLQS	human	IGKV1-39
L2-7-A	CDRL2	1K6Q	L	YATSLAD	house mouse	IGKV14-126
L2-7-A	CDRL2	1BJM	A	EDNSRAS	human	IGLV1-44

L2-7-A	CDRL2	3MUG	E	DVSHRPS	human	IGLV2-14
L2-7-A	CDRL2	1XF3	L	NAKTLAE	house mouse	IGKV12-44
L2-7-A	CDRL2	3O41	L	AASNPES	house mouse	IGKV3-3
L2-7-A	CDRL2	3MLW	L	RDDQRPS	human	IGLV1-47
L2-7-A	CDRL2	1FVC	C	SASFLYS	human	IGKV1-39
L2-7-A	CDRL2	1YYL	Q	GASTRAT	human	IGKV3-15
L2-7-A	CDRL2	1AXS	L	RTNRLVD	house mouse	IGKV14-111
L2-7-A	CDRL2	1U8L	A	DASSLES	human	IGKV1D-13
L2-7-A	CDRL2	1J05	A	RASNLES	house mouse	IGKV3-5
L2-7-A	CDRL2	3R1G	L	SASFLYS	human	IGKV1-13
L2-7-A	CDRL2	1NAK	L	KVSNRFS	house mouse	IGKV1-110
L2-7-A	CDRL2	1J05	L	RASNLES	house mouse	IGKV3-5
L2-7-A	CDRL2	2IMN	A	GASTRES	house mouse	IGKV4-1
L2-7-A	CDRL2	2DLF	L	KVSNRFS	house mouse	IGKV1-110
L2-7-A	CDRL2	4JPI	L	DASNRAT	human	IGKV3-11
L2-7-A	CDRL2	1BLN	A	KISNRFS	house mouse	IGKV1-117
L2-7-A	CDRL2	1IC4	L	YASQSIS	house mouse	IGKV5-43
L2-7-A	CDRL2	1T66	C	KVSNRFS	house mouse	IGKV1-110
L2-7-A	CDRL2	2OQJ	G	KASTLKT	human	IGKV1-5
L2-7-A	CDRL2	3UTZ	A	KVSNRFS	house mouse	IGKV1-117
L2-7-A	CDRL2	4G6K	L	YTSKLHS	human	IGKV1-39
L2-7-A	CDRL2	1MCH	A	EVNKRPS	human	IGLV2-8
L2-7-A	CDRL2	2W0K	A	EDNQRPS	human	IGLV6-57
L2-7-A	CDRL2	3GO1	L	KDTKRPS	human	IGLV3-25
L2-7-A	CDRL2	4I77	L	LASNLES	house mouse	IGKV3
L2-7-A	CDRL2	1LVE	A	WASTRES	human	IGKV4-1
L2-7-A	CDRL2	1Y0L	A	GTNKRAP	house mouse	IGLV1
L2-7-A	CDRL2	3QWO	L	DTSKLAS	human	IGKV1-5
L2-7-A	CDRL2	3BKM	L	QVSTRFS	house mouse	IGKV1-117
L2-7-A	CDRL2	1XIW	C	YTSRLHS	house mouse	IGKV10-96
L2-7-A	CDRL2	3WKM	M	LASNLES	house mouse	IGKV3-10
L2-7-A	CDRL2	1Q9O	C	WAATRES	house mouse	IGKV8-21
L2-7-A	CDRL2	2Q20	A	DASNLET	human	IGKV1-33
L2-7-A	CDRL2	3LS5	L	RTSNLAS	house mouse	IGKV4-91
L2-7-A	CDRL2	4GXV	L	SLNQRPS	human	IGLV1-44
L2-7-A	CDRL2	3PP3	L	QMSNLVS	house mouse	IGKV
L2-7-A	CDRL2	1BWW	A	EASNLQA	human	IGKV1-33
L2-7-A	CDRL2	4FNL	L	DASNLQR	human	IGKV1-33
L2-7-A	CDRL2	1ND0	A	LGTKLDS	house mouse	IGKV1-133
L2-7-A	CDRL2	2Q20	B	DASNLET	human	IGKV1-33
L2-7-A	CDRL2	1REI	A	EASNLQA	human	IGKV1-33
L2-7-A	CDRL2	3Q3G	C	WASTRES	house mouse	IGKV8-30
L2-7-A	CDRL2	2FJF	S	SASFLYS	human	IGKV1-39
L2-7-A	CDRL2	4AEI	M	KVSNRFS	house mouse	IGKV1-117
L2-7-A	CDRL2	3DGG	A	LASNLES	house mouse	IGKV3-12
L2-7-A	CDRL2	1AY1	L	DSTNLAS	house mouse	IGKV4-55
L2-7-A	CDRL2	3MUG	K	DVSHRPS	human	IGLV2-14
L2-7-A	CDRL2	4OD1	L	ENNKRPS	human	IGLV1-51
L2-7-A	CDRL2	1RFD	L	RVSNLAS	house mouse	IGKV2-137
L2-7-A	CDRL2	2RHE	A	YNDLLPS	human	IGLV1-36

L2-7-A	CDRL2	1IQD	A	GASTRAT	human	IGKV3-20
L2-7-A	CDRL2	2P8L	A	DASSLES	human	IGKV1D-13
L2-7-A	CDRL2	4F33	C	DTSKLAS	house mouse	IGKV4-59
L2-7-A	CDRL2	1CZ8	X	FTSSLHS	human	IGKV1-16
L2-7-A	CDRL2	2NXY	C	GASTRAT	human	IGKV3-15
L2-7-A	CDRL2	4G3Y	L	YASESMS	house mouse	IGKV5-48
L2-7-A	CDRL2	2FJF	U	SASFLYS	human	IGKV1-39
L2-7-A	CDRL2	4JO1	M	DASKLAS	rabbit	IGKV1S5
L2-7-A	CDRL2	1MCE	B	EVNKRPS	human	IGLV2-8
L2-7-A	CDRL2	3L7E	L	SGSTLQS	house mouse	IGKV16-104
L2-7-A	CDRL2	4F58	N	DVTNRPS	human	IGLV2-14
L2-7-A	CDRL2	3A6B	L	YASQSIS	house mouse	IGKV5-43
L2-7-A	CDRL2	2DQI	L	AASQSIS	house mouse	IGKV5-43
L2-7-A	CDRL2	2NY1	C	GASTRAT	human	IGKV3-15
L2-7-A	CDRL2	1GPO	L	YASQSIS	house mouse	IGKV5-43
L2-7-A	CDRL2	4IJ3	B	SASYRYT	house mouse	IGKV6-17
L2-7-A	CDRL2	1A8J	H	EVNKRPS	human	IGLV2-8
L2-7-A	CDRL2	3QCT	L	KVSNLFS	house mouse	IGKV
L2-7-A	CDRL2	2F58	L	AASTLES	house mouse	IGKV3-4
L2-7-A	CDRL2	4AIZ	D	KDSERPS	human	IGLV3-25
L2-7-A	CDRL2	1XF4	L	NAKTLAE	house mouse	IGKV12-44
L2-7-A	CDRL2	3F58	L	AASTLES	house mouse	IGKV3-4
L2-7-A	CDRL2	1MCB	B	EVNKRPS	human	IGLV2-8
L2-7-A	CDRL2	2BDN	L	GATSLET	house mouse	IGKV13-84
L2-7-A	CDRL2	1E4X	L	YTSTLHS	house mouse	IGKV10-96
L2-7-A	CDRL2	3ULS	L	EDSERPS	human	IGLV3-21
L2-7-A	CDRL2	3BDX	C	EDNQRPS	human	IGLV6-57
L2-7-A	CDRL2	2Z92	B	DTSKLPS	house mouse	IGKV4-59
L2-7-A	CDRL2	3DUU	C	WASTRES	house mouse	IGKV8-21
L2-7-A	CDRL2	1DEE	E	AASSLQS	human	IGKV1-39
L2-7-A	CDRL2	3LD8	B	YASTLFT	Norway rat	IGKV8S6
L2-7-A	CDRL2	1U92	A	DASSLES	human	IGKV1D-13
L2-7-A	CDRL2	1JRH	L	GATSLET	house mouse	IGKV13-84
L2-7-A	CDRL2	3NTC	L	WASTGES	house mouse	IGKV8-19
L2-7-A	CDRL2	1WT5	D	KVSDRFS	house mouse	IGKV
L2-7-A	CDRL2	2FX9	L	GASSRPS	human	IGKV3-20
L2-7-A	CDRL2	3CFK	L	GTNKRAP	house mouse	IGLV1
L2-7-A	CDRL2	4NM4	L	WSSTRES	human	IGKV4-1
L2-7-A	CDRL2	3D0L	A	DASSLES	human	IGKV1D-13
L2-7-A	CDRL2	1DN0	C	DASSRAT	human	IGKV3D-20
L2-7-A	CDRL2	3EO9	L	SGSTLQS	human	IGKV1-16
L2-7-A	CDRL2	3BDX	A	EDNQRPS	human	IGLV6-57
L2-7-A	CDRL2	1FL6	A	KVSNRFS	house mouse	IGKV1-117
L2-7-A	CDRL2	2HG5	B	YASESIS	house mouse	IGKV5-48
L2-7-A	CDRL2	3E8U	L	AASNLES	house mouse	IGKV3-4
L2-7-A	CDRL2	4JDV	B	DASNRAT	human	IGKV3-11
L2-7-A	CDRL2	1NFD	E	MDNKRPS	hamster	IGLV
L2-7-A	CDRL2	1BBD	L	WASTRES	house mouse	IGKV8-19
L2-7-A	CDRL2	3BZ4	E	HLSNLAS	house mouse	IGKV2-109
L2-7-A	CDRL2	2G5B	A	WASTRES	house mouse	IGKV8-21

L2-7-A	CDRL2	1Q72	L	RVSNLAS	house mouse	IGKV2-137
L2-7-A	CDRL2	1AI1	L	IASNLES	house mouse	IGKV3-10
L2-7-A	CDRL2	2BRR	X	STSNLAS	house mouse	IGKV4-57-1
L2-7-A	CDRL2	1EEQ	B	WASTRES	human	IGKV4-1
L2-7-A	CDRL2	2XTJ	B	NGSTLQS	human	IGKV1-6
L2-7-A	CDRL2	2W0K	B	EDNQRPS	human	IGLV6-57
L2-7-A	CDRL2	1AD0	C	ATSNLAS	human	IGKV1-16
L2-7-A	CDRL2	3U30	E	SAKFLYS	human	IGKV1-39
L2-7-A	CDRL2	3CDY	A	DASTLET	human	IGKV1-33
L2-7-A	CDRL2	4LMQ	L	NAVKLES	human	IGKV1-5
L2-7-A	CDRL2	2VQ1	E	KVSNRFS	house mouse	IGKV1-110
L2-7-A	CDRL2	3T0V	A	NDDRRSS	human	IGLV1-47
L2-7-A	CDRL2	1YYL	L	GASTRAT	human	IGKV3-15
L2-7-A	CDRL2	3T65	A	WASTRES	house mouse	IGKV8-21
L2-7-A	CDRL2	1I8M	A	NAKTLAE	house mouse	IGKV12-44
L2-7-A	CDRL2	1TJG	L	DASSLES	human	IGKV1D-13
L2-7-A	CDRL2	1Y0L	L	GTNKRAP	house mouse	IGLV1
L2-7-A	CDRL2	4LEX	L	KVSNRFS	house mouse	IGKV1-110
L2-7-A	CDRL2	3PP4	L	QMSNLVS	house mouse	IGKV
L2-7-A	CDRL2	4JPK	L	DASNRAT	human	IGKV3-11
L2-7-A	CDRL2	3RA7	M	YSSTLLS	house mouse	IGKV10-96
L2-7-A	CDRL2	2C1O	L	LVSKLDS	house mouse	IGKV1-133
L2-7-A	CDRL2	2HRP	M	AASNQGS	house mouse	IGKV3-2
L2-7-A	CDRL2	1NCW	L	LGTKLDS	house mouse	IGKV1-133
L2-7-A	CDRL2	1CZ8	L	FTSSLHS	human	IGKV1-16
L2-7-A	CDRL2	1U8Q	A	DASSLES	human	IGKV1D-13
L2-7-A	CDRL2	4AL8	L	LASNRHT	human	IGKV1-17
L2-7-A	CDRL2	3TNM	L	EVNNRPS	human	IGLV2-8
L2-7-A	CDRL2	3UPA	A	DADDLQS	human	IGKV1-39
L2-7-A	CDRL2	1FE8	M	YTSRLHS	house mouse	IGKV10-96
L2-7-A	CDRL2	2CMR	L	KASSLAS	human	IGKV1-5
L2-7-A	CDRL2	1RIV	L	RVSNLAS	house mouse	IGKV2-137
L2-7-A	CDRL2	2DQF	D	YASQSIS	house mouse	IGKV5-43
L2-7-A	CDRL2	3CFK	A	GTNKRAP	house mouse	IGLV1
L2-7-A	CDRL2	3DGG	C	LASNLES	house mouse	IGKV3-12
L2-7-A	CDRL2	3IJH	A	WASTRES	house mouse	IGKV8-21
L2-7-A	CDRL2	2P8M	A	DASSLES	human	IGKV1D-13
L2-7-A	CDRL2	3Q6G	M	ADDERSS	human	IGLV3-21
L2-7-A	CDRL2	2ZPK	L	GTNNRVP	house mouse	IGLV1
L2-7-A	CDRL2	4FZE	L	RDDKRPS	human	IGLV3-1
L2-7-A	CDRL2	1PSK	L	STSTLAS	house mouse	IGKV4-57
L2-7-A	CDRL2	2QQN	L	GASSRAS	human	IGKV1-5
L2-7-A	CDRL2	3FO1	L	KVSNRFS	house mouse	IGKV1-117
L2-7-A	CDRL2	3GI9	L	FASTRES	house mouse	IGKV8-24
L2-7-A	CDRL2	2DQF	A	YASQSIS	house mouse	IGKV5-43
L2-7-A	CDRL2	3CFK	M	GTNKRAP	house mouse	IGLV1
L2-7-A	CDRL2	3MLS	L	QDFKRPS	human	IGLV3-1
L2-7-A	CDRL2	3BN9	E	AASSLQS	human	IGKV1-12
L2-7-A	CDRL2	3NZH	L	YASNRYT	house mouse	IGKV6-32
L2-7-A	CDRL2	3KYM	C	DASNRAT	human	IGKV3-15

L2-7-A	CDRL2	2DDQ	L	KVSNRFS	house mouse	IGKV1-117
L2-7-A	CDRL2	4LVE	A	WASTRES	human	IGKV4-1
L2-7-A	CDRL2	1INE	L	GTNNRAP	house mouse	IGLV1
L2-7-A	CDRL2	2FX8	L	GASSRPS	human	IGKV3-20
L2-7-A	CDRL2	2VXU	M	ATSSLDS	house mouse	IGKV9-120
L2-7-A	CDRL2	2XZC	L	DNNKRPS	human	IGLV1-51
L2-7-A	CDRL2	1UYW	L	GASNRYT	house mouse	IGKV6-20
L2-7-A	CDRL2	1WTL	B	GASILET	human	IGKV1-33
L2-7-A	CDRL2	1A3R	L	WASTRES	house mouse	IGKV8-19
L2-7-A	CDRL2	2V7N	E	DSSSRAT	human	IGKV3-11
L2-7-A	CDRL2	3RPI	L	DGSKLER	human	IGKV1-33
L2-7-A	CDRL2	2ZPK	M	GTNNRVP	house mouse	IGLV1
L2-7-A	CDRL2	2AEP	L	SASYRYS	house mouse	IGKV6-15
L2-7-A	CDRL2	4NPY	A	DDSDRPS	human	IGLV3-21
L2-7-A	CDRL2	1AJ7	L	AASTLDS	house mouse	IGKV9-124
L2-7-A	CDRL2	1I8M	L	NAKTLAE	house mouse	IGKV12-44
L2-7-A	CDRL2	3NH7	N	DDSNRPS	human	IGLV3-1
L2-7-A	CDRL2	1P2C	A	YTSQSMS	house mouse	IGKV5-43
L2-7-A	CDRL2	2J88	L	NAKTLAE	house mouse	IGKV12-44
L2-7-A	CDRL2	4DGI	L	YASESIS	house mouse	IGKV5-48
L2-7-A	CDRL2	3B9K	C	YTSTLVS	Norway rat	IGKV19S2
L2-7-A	CDRL2	1MFB	L	DTNNRAP	house mouse	IGLV1
L2-7-A	CDRL2	1XGU	A	YASQSIS	house mouse	IGKV5-43
L2-7-A	CDRL2	3OKE	A	WASTRES	house mouse	IGKV8-21
L2-7-A	CDRL2	3QPQ	C	NAKTLAD	house mouse	IGKV12-41
L2-7-A	CDRL2	4MA8	L	YASESIS	house mouse	IGKV5-48
L2-7-A	CDRL2	1MCS	B	EVNKRPS	human	IGLV2-8
L2-7-A	CDRL2	4FQQ	E	DDSDRPS	human	IGLV3-21
L2-7-A	CDRL2	1MRD	L	KVSNRFS	house mouse	IGKV1-110
L2-7-A	CDRL2	4IMK	C	DDSDRPS	human	IGLV3-21
L2-7-A	CDRL2	2AJS	L	LMSTRAS	house mouse	IGKV2-112
L2-7-A	CDRL2	3OKL	A	WASTRES	house mouse	IGKV8-21
L2-7-A	CDRL2	1Q1J	L	GNNKRPS	human	IGLV1-51
L2-7-A	CDRL2	4JO2	M	DASKLAS	rabbit	IGKV1S5
L2-7-A	CDRL2	1J1X	L	YASQSIS	house mouse	IGKV5-43
L2-7-A	CDRL2	1C1E	L	KVSNRFS	house mouse	IGKV1-110
L2-7-A	CDRL2	1MCD	B	EVNKRPS	human	IGLV2-8
L2-7-A	CDRL2	1RHH	C	GASTRAT	human	IGKV3-20
L2-7-A	CDRL2	1EJO	L	RASNLES	house mouse	IGKV3-5
L2-7-A	CDRL2	3EYF	C	DASIRAT	human	IGKV3-11
L2-7-A	CDRL2	1TZI	A	DASYLYS	house mouse	IGKV6-17
L2-7-A	CDRL2	3B2U	K	DASNRAT	human	IGKV3D-11
L2-7-A	CDRL2	4G7Y	L	SASSLYS	human	IGKV1-5
L2-7-A	CDRL2	1G7M	A	YTTTLAD	house mouse	IGKV12-41
L2-7-A	CDRL2	1DCL	A	EVNKRPS	human	IGLV2-8
L2-7-A	CDRL2	1AD9	L	RMSNLAS	house mouse	IGKV
L2-7-A	CDRL2	3IFL	L	KVSNRFS	house mouse	IGKV1-117
L2-7-A	CDRL2	1FLR	L	KVSNRFS	house mouse	IGKV1-110
L2-7-A	CDRL2	4G6M	L	YTSKLHS	house mouse	IGKV10-96
L2-7-A	CDRL2	3U79	E	DASNLET	human	IGKV1-33

L2-7-A	CDRL2	3HZK	A	WASTRES	house mouse	IGKV8-21
L2-7-A	CDRL2	1HEZ	A	AASSLQS	human	IGKV1-39
L2-7-A	CDRL2	3HR5	P	KVSNRFS	house mouse	IGKV
L2-7-A	CDRL2	2V7N	G	DSSSRAT	human	IGKV3-11
L2-7-A	CDRL2	1CBV	L	KVSNRFS	house mouse	IGKV1-110
L2-7-A	CDRL2	3QPQ	E	NAKTLAD	house mouse	IGKV12-41
L2-7-A	CDRL2	1A14	L	YTSNLHS	house mouse	IGKV10-96
L2-7-A	CDRL2	2VH5	L	SASVLQS	human	IGKV1-39
L2-7-A	CDRL2	4K3H	E	ETDKKYS	human	IGLV7-46
L2-7-A	CDRL2	3RVV	C	RANRLIT	house mouse	IGKV14-111
L2-7-A	CDRL2	1U8J	A	DASSLES	human	IGKV1D-13
L2-7-A	CDRL2	3DVI	A	DASNLET	human	IGKV1-33
L2-7-A	CDRL2	3UTZ	D	KVSNRFS	house mouse	IGKV1-117
L2-7-A	CDRL2	1OAX	L	GTNNRAP	house mouse	IGLV1
L2-7-A	CDRL2	1XIW	G	YTSRLHS	house mouse	IGKV10-96
L2-7-A	CDRL2	1T2Q	L	KVSNRFS	house mouse	IGKV1-117
L2-7-A	CDRL2	3U79	C	DASNLET	human	IGKV1-33
L2-7-A	CDRL2	3L5W	A	SGSTLQS	house mouse	IGKV16-104
L2-7-A	CDRL2	2FD6	L	EISKLAS	house mouse	IGKV4-86
L2-7-A	CDRL2	2DQC	L	YASQSIS	house mouse	IGKV5-43
L2-7-A	CDRL2	1UWG	X	SASYRYS	house mouse	IGKV6-15
L2-7-A	CDRL2	1FN4	C	NTNSLQT	Norway rat	IGKV22S2
L2-7-A	CDRL2	3LHP	M	GASSRPS	human	IGKV3-20
L2-7-A	CDRL2	2Y5T	B	RASNLES	house mouse	IGKV3-5
L2-7-A	CDRL2	2I60	L	GASTRAT	human	IGKV3-15
L2-7-A	CDRL2	2AJZ	L	LMSTRAS	house mouse	IGKV2-112
L2-7-A	CDRL2	1UB5	L	QMSNLAS	house mouse	IGKV2-109
L2-7-A	CDRL2	1UWE	L	STSYRYS	house mouse	IGKV6-15
L2-7-A	CDRL2	1FVE	A	SASFLES	human	IGKV1-39
L2-7-A	CDRL2	1KIR	A	STTTLAD	house mouse	IGKV12-41
L2-7-A	CDRL2	4HWE	L	GASSRAT	human	IGKV3-20
L2-7-A	CDRL2	4JZN	P	DDSDRPS	human	IGLV3-21
L2-7-A	CDRL2	4F37	L	KVSKRFS	house mouse	IGKV1-117
L2-7-A	CDRL2	4HCR	N	EVSNRFS	house mouse	IGKV
L2-7-A	CDRL2	2D03	L	KVSNRFS	house mouse	IGKV1-110
L2-7-A	CDRL2	3GJF	L	DVIERSS	human	IGLV2-11
L2-7-A	CDRL2	3WKM	L	LASNLES	house mouse	IGKV3-10
L2-7-A	CDRL2	3DUS	A	WASTRES	house mouse	IGKV8-21
L2-7-A	CDRL2	2BX5	J	AASSLQS	human	IGKV1-39
L2-7-A	CDRL2	2FX8	N	GASSRPS	human	IGKV3-20
L2-7-A	CDRL2	1DQD	L	TTSNLAS	house mouse	IGKV4-57
L2-7-A	CDRL2	1A6V	N	GTNNRAP	house mouse	IGLV1
L2-7-A	CDRL2	3G5V	A	HGTNLDD	house mouse	IGKV14-100
L2-7-A	CDRL2	1GGC	L	RSSNLIS	house mouse	IGKV3-5
L2-7-A	CDRL2	1XF2	L	NAKTLAE	house mouse	IGKV12-44
L2-7-A	CDRL2	1GGI	M	RSSNLIS	house mouse	IGKV3-5
L2-7-A	CDRL2	3I02	A	WASTRES	house mouse	IGKV8-21
L2-7-A	CDRL2	1MRE	L	KVSNRFS	house mouse	IGKV1-110
L2-7-A	CDRL2	1QBM	L	NAKTLAD	house mouse	IGKV12-41
L2-7-A	CDRL2	3MUG	I	DVSHRPS	human	IGLV2-14

L2-7-A	CDRL2	3MNV	C	FASIRES	house mouse	IGKV8-24
L2-7-A	CDRL2	1MCR	B	EVNKRPS	human	IGLV2-8
L2-7-A	CDRL2	2Q8B	L	YASIRYT	house mouse	IGKV6-32
L2-7-A	CDRL2	4HIJ	A	GASNRAT	human	IGKV3-11
L2-7-A	CDRL2	1KFA	M	KVSSRFS	house mouse	IGKV1-110
L2-7-A	CDRL2	3OKD	A	WASTRES	house mouse	IGKV8-21
L2-7-A	CDRL2	1MHH	A	WASTRES	house mouse	IGKV8-21
L2-7-A	CDRL2	3MUG	G	DVSHRPS	human	IGLV2-14
L2-7-A	CDRL2	3PNW	V	SASSLYS	human	IGKV1-39
L2-7-A	CDRL2	3QEH	H	LGSNRAT	house mouse	IGKV
L2-7-A	CDRL2	1XGT	A	YASQSIS	house mouse	IGKV5-43
L2-7-A	CDRL2	3MLW	M	RDDQRPS	human	IGLV1-47
L2-7-A	CDRL2	1A6T	C	STSNLAS	house mouse	IGKV4-72
L2-7-A	CDRL2	1PG7	M	YATSLAE	human	IGKV1-39
L2-7-A	CDRL2	3VW3	L	TVSNRFS	house mouse	IGKV1-117
L2-7-A	CDRL2	1A2Y	A	YTTTLAD	house mouse	IGKV12-41
L2-7-A	CDRL2	1UWX	L	AATSLAD	house mouse	IGKV12-98
L2-7-A	CDRL2	4M1D	L	GNNKRPS	human	IGLV1-51
L2-7-A	CDRL2	4K3J	L	WASTRES	human	IGKV1D-13
L2-7-A	CDRL2	3AAZ	L	QVSTRFS	house mouse	IGKV
L2-7-A	CDRL2	2DTM	L	KVSNRFS	house mouse	IGKV1-117
L2-7-A	CDRL2	4HS6	L	LASNLNS	house mouse	IGKV3
L2-7-A	CDRL2	4F58	O	DVTNRPS	human	IGLV2-14
L2-7-A	CDRL2	1H3P	L	WASTRES	house mouse	IGKV8-21
L2-7-A	CDRL2	1VPO	L	KAYKRFP	house mouse	IGKV1-117
L2-7-A	CDRL2	4AIX	B	QDSKRPS	human	IGLV3-1
L2-7-A	CDRL2	1UCB	L	KVSNRFS	house mouse	IGKV1-117
L2-7-A	CDRL2	3GJE	A	DVIERSS	human	IGLV2-11
L2-7-A	CDRL2	3CFB	L	QMSNLAS	house mouse	IGKV2-109
L2-7-A	CDRL2	2YK1	L	RNNQRPS	human	IGLV1-47
L2-7-A	CDRL2	1MCP	L	GASTRES	house mouse	IGKV8-28
L2-7-A	CDRL2	3IET	A	KVSNRFS	house mouse	IGKV1-110
L2-7-A	CDRL2	4DVB	L	ATSNLAS	house mouse	IGKV4-57-1
L2-7-A	CDRL2	1NSN	L	YASNLES	house mouse	IGKV3-7
L2-7-A	CDRL2	2YSS	A	YASQSIS	human	IGKV3-15
L2-7-A	CDRL2	1AR2	A	EASNLQA	human	IGKV1-33
L2-7-A	CDRL2	1FJ1	C	YTSTLQP	house mouse	IGKV19-93
L2-7-A	CDRL2	4EOW	L	RNNRRPS	human	IGLV1-47
L2-7-A	CDRL2	3HI6	Y	AASSLQS	human	IGKV1-39
L2-7-A	CDRL2	3T0X	A	NDDRRPS	human	IGLV1-47
L2-7-A	CDRL2	1HQ4	C	STSNLAS	house mouse	IGKV4-79
L2-7-A	CDRL2	3DV6	A	WASTRES	house mouse	IGKV8-21
L2-7-A	CDRL2	1JV5	A	YTSRLHS	human	IGKV1-33
L2-7-A	CDRL2	3OAU	L	KASTLKT	human	IGKV1-5
L2-7-A	CDRL2	3RKD	L	SATNLAE	house mouse	IGKV12-46
L2-7-A	CDRL2	3GHE	L	DVSKRPS	human	IGLV2-14
L2-7-A	CDRL2	1UWG	L	SASYRYS	house mouse	IGKV6-15
L2-7-A	CDRL2	3EYU	L	KVSNRFS	house mouse	IGKV1-117
L2-7-A	CDRL2	2CD0	A	EDDHRPS	human	IGLV6-57
L2-7-A	CDRL2	3FZU	D	VASSLQS	human	IGKV1-39

L2-7-A	CDRL2	1CFS	A	RANRLMI	house mouse	IGKV14-111
L2-7-A	CDRL2	1CF8	L	STSNLAS	house mouse	IGKV4-79
L2-7-A	CDRL2	4IOI	A	SASFLYS	human	IGKV1-39
L2-7-A	CDRL2	1TZG	L	GASSRPS	human	IGKV3-20
L2-7-A	CDRL2	4GAY	B	LASNLNS	house mouse	IGKV3-10
L2-7-A	CDRL2	4EBQ	L	DTSNLAS	house mouse	IGKV4-55
L2-7-A	CDRL2	2UYL	V	KVSNRFS	house mouse	IGKV1-117
L2-7-A	CDRL2	2DQJ	L	YASQSIS	house mouse	IGKV5-43
L2-7-A	CDRL2	1ZA6	G	WASARES	house mouse	IGKV
L2-7-A	CDRL2	1NL0	L	DVSKRPS	human	IGLV1-51
L2-7-A	CDRL2	2JEL	L	KISNRFS	house mouse	IGKV1-117
L2-7-A	CDRL2	3ZL4	L	DNNKRPS	human	IGLV1-51
L2-7-A	CDRL2	1P7K	A	NAKTLAE	house mouse	IGKV12-44
L2-7-A	CDRL2	43CA	A	FASTRES	house mouse	IGKV8-24
L2-7-A	CDRL2	1S5H	A	YASESIS	house mouse	IGKV5-48
L2-7-A	CDRL2	1EEU	B	WASTRES	human	IGKV4-1
L2-7-A	CDRL2	3FO0	L	KVSNRFS	house mouse	IGKV1-117
L2-7-A	CDRL2	1MCQ	B	EVNKRPS	human	IGLV2-8
L2-7-A	CDRL2	4DN4	L	DASSRAT	human	IGKV3-11
L2-7-A	CDRL2	4JZO	B	QDNKRPS	human	IGLV3-1
L2-7-A	CDRL2	4JN2	L	LVSKLDS	house mouse	IGKV1-133
L2-7-A	CDRL2	4NRX	B	AASTLQS	human	IGKV1-39
L2-7-A	CDRL2	4NCC	1	STSNLAS	house mouse	IGKV4-57
L2-7-A	CDRL2	3U0T	C	QISRLDP	house mouse	IGKV
L2-7-A	CDRL2	1MHH	C	WASTRES	house mouse	IGKV8-21
L2-7-A	CDRL2	3CDY	B	DASTLET	human	IGKV1-33
L2-7-A	CDRL2	4NZR	L	ETYSKIA	human	IGKV3-15
L2-7-A	CDRL2	1LK3	L	KASNLAS	Norway rat	IGKV3S11
L2-7-A	CDRL2	3OKN	A	WASTRES	house mouse	IGKV8-21
L2-7-A	CDRL2	3CFD	L	YTSRLHS	house mouse	IGKV10-96
L2-7-A	CDRL2	3O6L	L	LVSKLDS	house mouse	IGKV1-135
L2-7-A	CDRL2	2Y07	L	GTNNRAP	house mouse	IGLV1
L2-7-A	CDRL2	1CT8	C	FASQSIP	house mouse	IGKV5-43
L2-7-A	CDRL2	4AT6	F	GTNNRAP	house mouse	IGLV1
L2-7-A	CDRL2	2NY3	C	GASTRAT	human	IGKV3-15
L2-7-A	CDRL2	1BM3	L	YASQSIS	house mouse	IGKV5-43
L2-7-A	CDRL2	2IQA	A	KVSNRFS	house mouse	IGKV1-117
L2-7-A	CDRL2	1TZG	M	GASSRPS	human	IGKV3-20
L2-7-A	CDRL2	3PGF	L	SASSLYS	human	IGKV1-39
L2-7-A	CDRL2	2AJ3	C	SASTLQS	human	IGKV1-9
L2-7-A	CDRL2	4AEI	L	KVSNRFS	house mouse	IGKV1-117
L2-7-A	CDRL2	1XGY	L	RMSNLAS	house mouse	IGKV2-109
L2-7-A	CDRL2	3LH2	L	GASSRPS	human	IGKV3-20
L2-7-A	CDRL2	1D6V	L	RANRLVD	house mouse	IGKV14-111
L2-7-A	CDRL2	4DTG	L	LVSILDS	house mouse	IGKV
L2-7-A	CDRL2	1N4X	M	KVSNRFS	house mouse	IGKV1-117
L2-7-A	CDRL2	1MCR	A	EVNKRPS	human	IGLV2-8
L2-7-A	CDRL2	1ND0	G	LGTKLDS	house mouse	IGKV1-133
L2-7-A	CDRL2	1DEE	C	AASSLQS	human	IGKV1-39
L2-7-A	CDRL2	4GMT	L	SASNRYS	house mouse	IGKV6-15

L2-7-A	CDRL2	1RZG	B	KASSLKS	human	IGKV1-5
L2-7-A	CDRL2	4JR9	L	YTSTLES	house mouse	IGKV19-93
L2-7-A	CDRL2	3OAZ	K	KASTLKT	human	IGKV1-5
L2-7-A	CDRL2	4G6J	L	YASQSFS	human	IGKV1-39
L2-7-A	CDRL2	3B2U	R	DASNRAT	human	IGKV3D-11
L2-7-A	CDRL2	1F90	L	LVSKLDS	house mouse	IGKV1-133
L2-7-A	CDRL2	2R1X	A	WASTRES	house mouse	IGKV8-21
L2-7-A	CDRL2	3LRG	A	DDDLLAP	human	IGLV1-36
L2-7-A	CDRL2	1TJH	L	DASSLES	human	IGKV1D-13
L2-7-A	CDRL2	2NY5	L	GASTRAT	human	IGKV3-15
L2-7-A	CDRL2	1AQK	L	ANTNRPS	human	IGLV1-40
L2-7-A	CDRL2	3HC3	L	SASYRYS	human	IGKV1-16
L2-7-A	CDRL2	2F5B	L	DASSLES	human	IGKV1D-13
L2-7-A	CDRL2	3U79	D	DASNLET	human	IGKV1-33
L2-7-A	CDRL2	1U8I	A	DASSLES	human	IGKV1D-13
L2-7-A	CDRL2	3G6A	L	DDNDRPS	human	IGLV3-1
L2-7-A	CDRL2	1KCV	L	GASNRYT	house mouse	IGKV6-20
L2-7-A	CDRL2	4HJJ	L	TASTRAT	human	IGKV4-1
L2-7-A	CDRL2	2GCY	A	RASNLEP	house mouse	IGKV3-5
L2-7-A	CDRL2	1DN0	A	DASSRAT	human	IGKV3D-20
L2-7-A	CDRL2	4LVE	B	WASTRES	human	IGKV4-1
L2-7-A	CDRL2	2VL5	B	RASNLES	house mouse	IGKV3-5
L2-7-A	CDRL2	3S34	L	DASNLDT	human	IGKV1-12
L2-7-A	CDRL2	1DBA	L	KVSNRFY	house mouse	IGKV1-110
L2-7-A	CDRL2	3KR3	L	TASTLQS	human	IGKV1-39
L2-7-A	CDRL2	2C1P	A	LVSKLDS	house mouse	IGKV1-133
L2-7-A	CDRL2	3ZKM	D	LASNLES	house mouse	IGKV3-10
L2-7-A	CDRL2	3HZM	A	WASTRES	house mouse	IGKV8-21
L2-7-A	CDRL2	1FL5	L	KVSNRFS	house mouse	IGKV1-117
L2-7-A	CDRL2	4JLR	L	DTSKLAS	human	IGKV1-5
L2-7-A	CDRL2	4LF3	A	AASSLES	house mouse	IGKV11-125
L2-7-A	CDRL2	2BX5	O	AASSLQS	human	IGKV1-39
L2-7-A	CDRL2	3STZ	B	YASESIS	house mouse	IGKV5-48
L2-7-A	CDRL2	4HIH	C	GASNRAT	human	IGKV3-11
L2-7-A	CDRL2	1EEQ	A	WASTRES	human	IGKV4-1
L2-7-A	CDRL2	1MCE	A	EVNKRPS	human	IGLV2-8
L2-7-A	CDRL2	2MPA	L	KVSNRFS	house mouse	IGKV1-110
L2-7-A	CDRL2	1JHK	L	NAKTLAD	house mouse	IGKV12-41
L2-7-A	CDRL2	3SOB	L	SASFLYS	human	IGKV1-39
L2-7-A	CDRL2	1N8Z	A	SASFLYS	human	IGKV1-39
L2-7-A	CDRL2	2BRR	L	STSNLAS	house mouse	IGKV4-57-1
L2-7-A	CDRL2	2R23	A	WASTRES	house mouse	IGKV8-21
L2-7-A	CDRL2	4K2U	M	YTSRLHS	house mouse	IGKV10-96
L2-7-A	CDRL2	1LIL	B	QDNRRPS	human	IGLV3-1
L2-7-A	CDRL2	3PNW	A	SASSLYS	human	IGKV1-39
L2-7-A	CDRL2	3LRS	D	DVSHRPS	human	IGLV2-14
L2-7-A	CDRL2	1DLF	L	KVSNRFS	house mouse	IGKV1-110
L2-7-A	CDRL2	2HFG	L	SASFLYS	human	IGKV1-5
L2-7-A	CDRL2	3EFD	L	SASSLYS	human	IGKV1-39
L2-7-A	CDRL2	3IFO	L	KVSNRFS	house mouse	IGKV1-117

L2-7-A	CDRL2	3C6S	C	HLSNLAS	house mouse	IGKV2-109
L2-7-A	CDRL2	3BKY	L	APSNLAS	house mouse	IGKV4-72
L2-7-A	CDRL2	3U6R	L	DASNRAT	human	IGKV3-11
L2-7-A	CDRL2	1NGX	L	SASNRYT	house mouse	IGKV6-13
L2-7-A	CDRL2	1XGR	A	YASQSIS	house mouse	IGKV5-43
L2-7-A	CDRL2	3EYF	A	DASIRAT	human	IGKV3-11
L2-7-A	CDRL2	3EYV	L	KVSNRFS	house mouse	IGKV1-117
L2-7-A	CDRL2	4K9E	L	SASSLYS	house mouse	IGKV1-12
L2-7-A	CDRL2	1DVF	C	YTSRLHS	house mouse	IGKV10-96
L2-7-A	CDRL2	4LMQ	I	NAVKLES	human	IGKV1-5
L2-7-A	CDRL2	1MCH	B	EVNKRPS	human	IGLV2-8
L2-7-A	CDRL2	1MEX	L	SASYRYS	house mouse	IGKV6-15
L2-7-A	CDRL2	1AD0	A	ATSNLAS	human	IGKV1-16
L2-7-A	CDRL2	3MLS	N	QDFKRPS	human	IGLV3-1
L2-7-A	CDRL2	2PCP	C	KVTNRFS	house mouse	IGKV1-117
L2-7-A	CDRL2	4M1D	M	GNNKRPS	human	IGLV1-51
L2-7-A	CDRL2	3NH7	O	DDSNRPS	human	IGLV3-1
L2-7-A	CDRL2	2FJF	O	SASFLYS	human	IGKV1-39
L2-7-A	CDRL2	1PG7	W	GTNNRAP	house mouse	IGLV1
L2-7-A	CDRL2	3R06	L	YTNKLAD	house mouse	IGKV14-111
L2-7-A	CDRL2	4FFY	L	LASNLES	house mouse	IGKV3-10
L2-7-A	CDRL2	1OAQ	L	GTNNRAP	house mouse	IGLV1
L2-7-A	CDRL2	1ZLS	L	KASTLKT	human	IGKV1-5
L2-7-A	CDRL2	1R3L	A	YASESIS	house mouse	IGKV5-48
L2-7-A	CDRL2	3MLV	L	QDFKRPS	human	IGLV3-1
L2-7-A	CDRL2	2FJF	G	SASFLYS	human	IGKV1-39
L2-7-A	CDRL2	1NGY	A	SASNRYT	house mouse	IGKV6-13
L2-7-A	CDRL2	4F58	M	DVTNRPS	human	IGLV2-14
L2-7-A	CDRL2	1EHL	L	KVSNRFS	house mouse	IGKV1-117
L2-7-A	CDRL2	4D9L	N	GNSNRPS	human	IGLV1-40
L2-7-A	CDRL2	3GGW	C	HLSNLAS	house mouse	IGKV2-109
L2-7-A	CDRL2	1OAU	L	GTNNRAP	house mouse	IGLV1
L2-7-A	CDRL2	2YBR	H	DASNRAT	human	IGKV3D-11
L2-7-A	CDRL2	2VDQ	L	YGTNLVD	house mouse	IGKV14-100
L2-7-A	CDRL2	2BX5	G	AASSLQS	human	IGKV1-39
L2-7-A	CDRL2	3T3P	F	YGTNLVD	house mouse	IGKV14-100
L2-7-A	CDRL2	1NBV	L	KVSNRFS	house mouse	IGKV1-110
L2-7-A	CDRL2	1OCW	L	GTNNRAP	house mouse	IGLV1
L2-7-A	CDRL2	3UJI	L	EDTKRPS	human	IGLV3-1
L2-7-A	CDRL2	2AI0	L	KVSNRFS	house mouse	IGKV1-117
L2-7-A	CDRL2	1E4W	L	YTSTLHS	house mouse	IGKV10-96
L2-7-A	CDRL2	3RPI	B	DGSKLER	human	IGKV1-33
L2-7-A	CDRL2	3IJH	C	WASTRES	house mouse	IGKV8-21
L2-7-A	CDRL2	1A4J	L	KVSNRFS	house mouse	IGKV1-110
L2-7-A	CDRL2	2OJZ	L	KVSNRFS	house mouse	IGKV1-117
L2-7-A	CDRL2	3RU8	L	GVSNRAS	human	IGKV3-20
L2-7-A	CDRL2	3CFK	O	GTNKRAP	house mouse	IGLV1
L2-7-A	CDRL2	2PW1	A	DASSLES	human	IGKV1D-13
L2-7-A	CDRL2	1Q9L	A	WASTRES	house mouse	IGKV8-21
L2-7-A	CDRL2	2C1P	L	LVSKLDS	house mouse	IGKV1-133

L2-7-A	CDRL2	1KCU	L	GASNRYT	house mouse	IGKV6-20
L2-7-A	CDRL2	1M71	A	KVSNRFS	house mouse	IGKV1-110
L2-7-A	CDRL2	3C6S	G	HLSNLAS	house mouse	IGKV2-109
L2-7-A	CDRL2	2I60	Q	GASTRAT	human	IGKV3-15
L2-7-A	CDRL2	3INU	N	WASTRES	human	IGKV4-1
L2-7-A	CDRL2	2FJF	A	SASFLYS	human	IGKV1-39
L2-7-A	CDRL2	4JPI	B	DASNRAT	human	IGKV3-11
L2-7-A	CDRL2	2IQ9	L	KVSNRFS	house mouse	IGKV1-117
L2-7-A	CDRL2	1MCC	B	EVNKRPS	human	IGLV2-8
L2-7-A	CDRL2	3LEX	B	LVSKLAS	house mouse	IGKV1-135
L2-7-A	CDRL2	1IT9	L	AASNLES	house mouse	IGKV3-4
L2-7-A	CDRL2	1BJ1	L	FTSSLHS	human	IGKV1-33
L2-7-A	CDRL2	2BX5	I	AASSLQS	human	IGKV1-39
L2-7-A	CDRL2	3H3P	L	GASSRPS	human	IGKV3-20
L2-7-A	CDRL2	2B1H	L	RNDHRSS	human	IGLV1-47
L2-7-A	CDRL2	3CMO	L	YTSRLQS	house mouse	IGKV10-94
L2-7-A	CDRL2	4HPO	L	EDNQRPS	human	IGLV6-57
L2-7-A	CDRL2	4M5Y	L	DDSDRPS	human	IGLV3-21
L2-7-A	CDRL2	3BT2	L	EISKLAS	house mouse	IGKV4-86
L2-7-A	CDRL2	12E8	M	SASNRYT	house mouse	IGKV6-13
L2-7-A	CDRL2	1KIP	A	YTTTLAD	house mouse	IGKV12-41
L2-7-A	CDRL2	1RIH	L	YASQSIS	house mouse	IGKV5-43
L2-7-A	CDRL2	2B1A	L	RNDHRSS	human	IGLV1-47
L2-7-A	CDRL2	1RJL	A	MASNLHT	house mouse	IGKV11-125
L2-7-A	CDRL2	1G7L	A	YTTTLAD	house mouse	IGKV12-41
L2-7-A	CDRL2	1XF5	C	WASTRES	house mouse	IGKV8-21
L2-7-A	CDRL2	2F19	L	YTSRLHS	house mouse	IGKV10-96
L2-7-A	CDRL2	3O2D	L	WASTRES	human	IGKV4-1
L2-7-A	CDRL2	3HR5	Q	KVSNRFS	house mouse	IGKV
L2-7-A	CDRL2	1L7T	L	KAYKRFP	house mouse	IGKV1-117
L2-7-A	CDRL2	3QWO	B	DTSKLAS	human	IGKV1-5
L2-7-A	CDRL2	4I2X	C	SASYRYT	human	IGKV6-17
L2-7-A	CDRL2	2OLD	A	EVTNRPS	human	IGLV2-14
L2-7-A	CDRL2	2W9D	L	DTSKLAS	house mouse	IGKV4-59
L2-7-A	CDRL2	1AXT	L	KVSNRFS	house mouse	IGKV1-110
L2-7-A	CDRL2	1Y18	E	GTNKRAP	house mouse	IGLV1
L2-7-A	CDRL2	1QAC	B	WASTRES	human	IGKV4-1
L2-7-A	CDRL2	1KN4	L	LVSKLDS	house mouse	IGKV1-133
L2-7-A	CDRL2	3D0V	A	DASSLES	human	IGKV1D-13
L2-7-A	CDRL2	3PNW	P	SASSLYS	human	IGKV1-39
L2-7-A	CDRL2	1ZA6	E	WASARES	house mouse	IGKV
L2-7-A	CDRL2	3MLS	M	QDFKRPS	human	IGLV3-1
L2-7-A	CDRL2	1ZAN	L	NTDTLHT	Norway rat	IGKV12S34
L2-7-A	CDRL2	3L95	A	SASFLYS	human	IGKV1-39
L2-7-A	CDRL2	4LLY	D	SASYRYT	human	IGKV1D-13
L2-7-A	CDRL2	4H20	L	SASYRYT	house mouse	IGKV6-17
L2-7-A	CDRL2	3L7F	D	SGSTLQS	human	IGKV3-11
L2-7-A	CDRL2	3CFK	J	GTNKRAP	house mouse	IGLV1
L2-7-A	CDRL2	3RVW	C	RANRLIT	house mouse	IGKV14-111
L2-7-A	CDRL2	4H88	L	YASESIS	house mouse	IGKV5-48

L2-7-A	CDRL2	3NAA	L	AASSLQS	human	IGKV1-39
L2-7-A	CDRL2	2ATK	B	YASESIS	house mouse	IGKV5-48
L2-7-A	CDRL2	1W72	M	DDSDRPS	human	IGLV3-21
L2-7-A	CDRL2	4D9L	L	GNSNRPS	human	IGLV1-40
L2-7-A	CDRL2	1MJJ	A	RMSNLAS	house mouse	IGKV2-137
L2-7-A	CDRL2	3H0T	A	EDSQRPS	human	IGLV6-57
L2-7-A	CDRL2	4GW1	C	YASESIS	house mouse	IGKV5-48
L2-7-A	CDRL2	3IKC	A	WASTRES	house mouse	IGKV8-21
L2-7-A	CDRL2	4LLY	B	SASYRYT	human	IGKV1D-13
L2-7-A	CDRL2	1LGV	B	EVNKRPS	human	IGLV2-23
L2-7-A	CDRL2	1RZ8	A	GASTRAT	human	IGKV3-15
L2-7-A	CDRL2	2NY2	C	GASTRAT	human	IGKV3-15
L2-7-A	CDRL2	1IC5	L	YASQSIS	house mouse	IGKV5-43
L2-7-A	CDRL2	4JRE	L	YTSTLES	house mouse	IGKV19-93
L2-7-A	CDRL2	2NY4	C	GASTRAT	human	IGKV3-15
L2-7-A	CDRL2	1YEH	L	LVSKLDS	house mouse	IGKV1-133
L2-7-A	CDRL2	3B5G	A	EDNQRPS	human	IGLV6-57
L2-7-A	CDRL2	4JO3	M	KASTLAS	rabbit	IGKV1S3
L2-7-A	CDRL2	1ZA6	C	WASARES	house mouse	IGKV
L2-7-A	CDRL2	1WCB	A	STSNLAS	house mouse	IGKV4-57
L2-7-A	CDRL2	3V6O	F	LASNRHT	house mouse	IGKV6-14
L2-7-A	CDRL2	1IC7	L	YASQSIS	house mouse	IGKV5-43
L2-7-A	CDRL2	2FJG	A	SASFLYS	human	IGKV1-39
L2-7-A	CDRL2	1GPO	M	YASQSIS	house mouse	IGKV5-43
L2-7-A	CDRL2	1FVC	A	SASFLYS	human	IGKV1-39
L2-7-A	CDRL2	3OKK	A	WASTRES	house mouse	IGKV8-21
L2-7-A	CDRL2	3NIF	L	YGTNLVD	house mouse	IGKV14-100
L2-7-A	CDRL2	2AJX	L	LMSTRAS	house mouse	IGKV2-112
L2-7-A	CDRL2	1FGV	L	YTSTLES	human	IGKV1-33
L2-7-A	CDRL2	1FVD	C	SASFLES	human	IGKV1-39
L2-7-A	CDRL2	3NID	F	YGTNLVD	house mouse	IGKV14-100
L2-7-A	CDRL2	1FL3	B	QMSNLAS	house mouse	IGKV2-109
L2-7-A	CDRL2	1OSP	L	GATSLET	house mouse	IGKV13-84
L2-7-A	CDRL2	1OPG	L	YASQSIS	house mouse	IGKV5-43
L2-7-A	CDRL2	2W65	B	LVSKLDS	house mouse	IGKV1-135
L2-7-A	CDRL2	3U2S	B	DVSKRPS	human	IGLV2-14
L2-7-A	CDRL2	4K3H	H	ETDKKYS	human	IGLV7-46
L2-7-A	CDRL2	3U79	G	DASNLET	human	IGKV1-33
L2-7-A	CDRL2	1JVK	B	EVNKRPS	human	IGLV2-23
L2-7-A	CDRL2	1FL3	L	QMSNLAS	house mouse	IGKV2-109
L2-7-A	CDRL2	2UYL	M	KVSNRFS	house mouse	IGKV1-117
L2-7-A	CDRL2	2R8S	L	SASSLYS	human	IGKV1-39
L2-7-A	CDRL2	1A6W	L	GTNNRAP	house mouse	IGLV1
L2-7-A	CDRL2	4G6A	D	LASNLNS	house mouse	IGKV3
L2-7-A	CDRL2	3LRH	K	DDDLLAP	human	IGLV1-36
L2-7-A	CDRL2	2DQG	L	YASQSIS	house mouse	IGKV5-43
L2-7-A	CDRL2	2GK0	L	KVSNRFS	house mouse	IGKV1-117
L2-7-A	CDRL2	4HBC	L	ETSTLAS	rabbit	IGKV1S3
L2-7-A	CDRL2	3L7F	A	SGSTLQS	human	IGKV3-11
L2-7-A	CDRL2	1MLC	C	YVSQSSS	house mouse	IGKV5-43

L2-7-A	CDRL2	2FX9	M	GASSRPS	human	IGKV3-20
L2-7-A	CDRL2	1U93	A	DASSLES	human	IGKV1D-13
L2-7-A	CDRL2	2ORB	M	AISNRGS	house mouse	IGKV3-2
L2-7-A	CDRL2	3MLX	L	ENSKRPS	human	IGLV1-51
L2-7-A	CDRL2	1UM4	L	AGSTLDS	house mouse	IGKV9-124
L2-7-A	CDRL2	1YNK	L	RVSNRFS	house mouse	IGKV1-110
L2-7-A	CDRL2	2CGR	L	RVSNRFS	house mouse	IGKV1-110
L2-7-A	CDRL2	3QRG	L	AASNPES	house mouse	IGKV3
L2-7-A	CDRL2	3GIZ	L	DASNRAT	human	IGKV3-11
L2-7-A	CDRL2	3V0W	L	KASNLHT	house mouse	IGKV11-125
L2-7-A	CDRL2	1MJ8	L	RMSNLAS	house mouse	IGKV2-137
L2-7-A	CDRL2	3U7A	A	DASTLET	human	IGKV1-33
L2-7-A	CDRL2	3RA7	L	YSSTLLS	house mouse	IGKV10-96
L2-7-A	CDRL2	3LRH	E	DDDLLAP	human	IGLV1-36
L2-7-A	CDRL2	2BFV	L	KVSNRFS	house mouse	IGKV1-110
L2-7-A	CDRL2	1SBS	L	WASTRES	house mouse	IGKV8-30
L2-7-A	CDRL2	3MCK	A	KVSNRFS	house mouse	IGKV1-117
L2-7-A	CDRL2	1YEK	L	LVSKLDS	house mouse	IGKV1-133
L2-7-A	CDRL2	1A7O	L	YTTTLAD	house mouse	IGKV12-41
L2-7-A	CDRL2	8FAB	C	KDTQRPS	human	IGLV3-25
L2-7-A	CDRL2	2H8P	B	YASESIS	house mouse	IGKV5-48
L2-7-A	CDRL2	3A67	L	YASQSIS	house mouse	IGKV5-43
L2-7-A	CDRL2	2OR9	L	AISNRGS	house mouse	IGKV3-2
L2-7-A	CDRL2	1UB6	B	QMSNLAS	house mouse	IGKV2-109
L2-7-A	CDRL2	4FQQ	A	DDSDRPS	human	IGLV3-21
L2-7-A	CDRL2	1ZA6	A	WASARES	house mouse	IGKV
L2-7-A	CDRL2	1RUA	L	LGTKLDS	house mouse	IGKV1-133
L2-7-A	CDRL2	1NGW	L	SASNRYT	house mouse	IGKV6-13
L2-7-A	CDRL2	3HNT	L	YTSRLHS	house mouse	IGKV10-96
L2-7-A	CDRL2	4KI5	F	AASTLDS	house mouse	IGKV9-124
L2-7-A	CDRL2	2OSL	B	ATSNLAS	house mouse	IGKV4-72
L2-7-A	CDRL2	43CA	C	FASTRES	house mouse	IGKV8-24
L2-7-A	CDRL2	1HQ4	A	STSNLAS	house mouse	IGKV4-79
L2-7-A	CDRL2	3QG6	L	KLSSRFS	house mouse	IGKV1-110
L2-7-A	CDRL2	3B2U	U	DASNRAT	human	IGKV3D-11
L2-7-A	CDRL2	4NRX	E	AASTLQS	human	IGKV1-39
L2-7-A	CDRL2	2BX5	L	AASSLQS	human	IGKV1-39
L2-7-A	CDRL2	2XKN	A	YTSRLHS	house mouse	IGKV10-96
L2-7-A	CDRL2	1GGI	L	RSSNLIS	house mouse	IGKV3-5
L2-7-A	CDRL2	2JK5	B	YASESIS	house mouse	IGKV5-48
L2-7-A	CDRL2	1CLO	L	ATSNLAS	house mouse	IGKV4-72
L2-7-A	CDRL2	3BJL	B	EDNSRAS	human	IGLV1-44
L2-7-A	CDRL2	1MCL	A	EVNKRPS	human	IGLV2-8
L2-7-A	CDRL2	3L5W	L	SGSTLQS	house mouse	IGKV16-104
L2-7-A	CDRL2	1BJM	B	EDNSRAS	human	IGLV1-44
L2-7-A	CDRL2	4JO3	L	KASTLAS	rabbit	IGKV1S3
L2-7-A	CDRL2	1EMT	L	YTSSLRS	house mouse	IGKV10-94
L2-7-A	CDRL2	4AT6	K	GTNNRAP	house mouse	IGLV1
L2-7-A	CDRL2	2PW2	A	DASSLES	human	IGKV1D-13
L2-7-A	CDRL2	3HR5	L	KVSNRFS	house mouse	IGKV

L2-7-A	CDRL2	3OKO	A	WASTRES	house mouse	IGKV8-21
L2-7-A	CDRL2	3QEG	L	GASSRAT	human	IGKV3-20
L2-7-A	CDRL2	3S37	L	DASNLDT	human	IGKV1-12
L2-7-A	CDRL2	3VFG	L	SASNRYS	house mouse	IGKV6-32
L2-7-A	CDRL2	1U8K	A	DASSLES	human	IGKV1D-13
L2-7-A	CDRL2	2NY7	L	GVSNRAS	human	IGKV3-20
L2-7-A	CDRL2	3CLF	L	LASYRYS	house mouse	IGKV6-15
L2-7-A	CDRL2	3QCV	L	KVSNLFS	house mouse	IGKV
L2-7-A	CDRL2	3U30	B	SAKFLYS	human	IGKV1-39
L2-7-A	CDRL2	3CDC	B	DASNLET	human	IGKV1-33
L2-7-A	CDRL2	4FQC	L	NNQDRPS	human	IGLV3-21
L2-7-A	CDRL2	3B2U	D	DASNRAT	human	IGKV3D-11
L2-7-A	CDRL2	3BDY	L	WGSYLYS	human	IGKV1-39
L2-7-A	CDRL2	1B0W	B	DASTLET	human	IGKV1-33
L2-7-A	CDRL2	2FJF	Q	SASFLYS	human	IGKV1-39
L2-7-A	CDRL2	4ETQ	L	DTSNLAS	house mouse	IGKV4-55
L2-7-A	CDRL2	1MRC	L	KVSNRFS	house mouse	IGKV1-110
L2-7-A	CDRL2	1CFV	L	KVSNRFS	house mouse	IGKV1-110
L2-7-A	CDRL2	1HZH	M	GVSNRAS	human	IGKV3-20
L2-7-A	CDRL2	4FQ2	L	NNQDRPS	human	IGLV3-21
L2-7-A	CDRL2	2BX5	H	AASSLQS	human	IGKV1-39
L2-7-A	CDRL2	3BZ4	G	HLSNLAS	house mouse	IGKV2-109
L2-7-A	CDRL2	3NCJ	L	AASSLQS	human	IGKV1-39
L2-7-A	CDRL2	4N9G	F	YTSHLES	human	IGKV1-16
L2-7-A	CDRL2	3QEH	F	LGSNRAT	house mouse	IGKV
L2-7-A	CDRL2	1WC7	L	STSNLAS	house mouse	IGKV4-57
L2-7-A	CDRL2	1IND	L	GTNNRAP	house mouse	IGLV1
L2-7-A	CDRL2	1OP3	L	KASTLKT	human	IGKV1-5
L2-7-A	CDRL2	2Q8A	L	YASIRYT	house mouse	IGKV6-32
L2-7-A	CDRL2	1OAX	M	GTNNRAP	house mouse	IGLV1
L2-7-A	CDRL2	1ND0	C	LGTKLDS	house mouse	IGKV1-133
L2-7-A	CDRL2	4F57	L	DVTNRPS	human	IGLV2-14
L2-7-A	CDRL2	1BFO	A	NTNNLQT	Norway rat	IGKV22S7
L2-7-A	CDRL2	1DQM	L	YASQSIS	house mouse	IGKV5-43
L2-7-A	CDRL2	1UZ6	E	QMSNLAS	house mouse	IGKV2-109
L2-7-A	CDRL2	1CD0	A	EDNQRPS	human	IGLV6-57
L2-7-A	CDRL2	2G5B	E	WASTRES	house mouse	IGKV8-21
L2-7-A	CDRL2	1MF2	M	AASNQGS	house mouse	IGKV3-2
L2-7-A	CDRL2	2Q76	C	YTSQSMS	house mouse	IGKV5-43
L2-7-A	CDRL2	1QYG	L	RVSNLAS	house mouse	IGKV2-137
L2-7-A	CDRL2	1PG7	Y	GTNNRAP	house mouse	IGLV1
L2-7-A	CDRL2	4JN2	A	LVSKLDS	house mouse	IGKV1-133
L2-7-A	CDRL2	1F4Y	L	ATNNRAA	house mouse	IGLV1
L2-7-A	CDRL2	3DUS	C	WASTRES	house mouse	IGKV8-21
L2-7-A	CDRL2	1FOR	L	SSSNLAS	house mouse	IGKV4-57
L2-7-A	CDRL2	1QFU	L	KVSNRFS	house mouse	IGKV1-110
L2-7-A	CDRL2	1D5I	L	RANRLVD	house mouse	IGKV14-111
L2-7-A	CDRL2	1IQW	L	AASNLES	house mouse	IGKV3-4
L2-7-A	CDRL2	1UZ6	M	QMSNLAS	house mouse	IGKV2-109
L2-7-A	CDRL2	1CFN	A	RANRLMI	house mouse	IGKV14-111

L2-7-A	CDRL2	1HH9	A	RANRLMI	house mouse	IGKV14-111
L2-7-A	CDRL2	3Q3G	A	WASTRES	house mouse	IGKV8-30
L2-7-A	CDRL2	2IFF	L	DTSKLAS	house mouse	IGKV4-59
L2-7-A	CDRL2	3SGE	I	LVSKLDS	house mouse	IGKV1-135
L2-7-A	CDRL2	3H42	L	GNSNRPS	human	IGLV1-40
L2-7-A	CDRL2	1I7Z	A	LASNLAS	house mouse	IGKV3-12
L2-7-A	CDRL2	1RZ8	C	GASTRAT	human	IGKV3-15
L2-7-A	CDRL2	15C8	L	STSNLAS	house mouse	IGKV4-74
L2-7-A	CDRL2	1SEQ	L	TTSNLAS	house mouse	IGKV4-80
L2-7-A	CDRL2	3O2W	L	KVSNRFS	house mouse	IGKV1-110
L2-7-A	CDRL2	3MXV	L	YASNRYT	house mouse	IGKV6-32
L2-7-A	CDRL2	2GK0	A	KVSNRFS	house mouse	IGKV1-117
L2-7-A	CDRL2	2WUC	L	SASFLYS	human	IGKV1D-13
L2-7-A	CDRL2	1I3G	L	WASTRHT	house mouse	IGKV6-23
L2-7-A	CDRL2	1CK0	L	WASTRGS	house mouse	IGKV8-21
L2-7-A	CDRL2	1MCS	A	EVNKRPS	human	IGLV2-8
L2-7-A	CDRL2	1RZG	D	KASSLKS	human	IGKV1-5
L2-7-A	CDRL2	4JO4	M	KASTLAS	rabbit	IGKV1S3
L2-7-A	CDRL2	25C8	L	STSNLAS	house mouse	IGKV4-74
L2-7-A	CDRL2	1PW3	B	EDNQRPS	human	IGLV6-57
L2-7-A	CDRL2	1XF3	A	NAKTLAE	house mouse	IGKV12-44
L2-7-A	CDRL2	2Q1E	A	DASTLET	human	IGKV1-33
L2-7-A	CDRL2	1JGL	L	NAKTLAD	house mouse	IGKV12-41
L2-7-A	CDRL2	3R06	C	YTNKLAD	house mouse	IGKV14-111
L2-7-A	CDRL2	3CFB	A	QMSNLAS	house mouse	IGKV2-109
L2-7-A	CDRL2	4LLU	B	SASYRYT	human	IGKV1-16
L2-7-A	CDRL2	1EK3	B	WASSRES	human	IGKV4-1
L2-7-A	CDRL2	1BWW	B	EASNLQA	human	IGKV1-33
L2-7-A	CDRL2	4AEH	L	AATNLAA	house mouse	IGKV12-46
L2-7-A	CDRL2	1PW3	A	EDNQRPS	human	IGLV6-57
L2-7-A	CDRL2	2DQE	L	YASQSIS	house mouse	IGKV5-43
L2-7-A	CDRL2	1MLC	A	YVSQSSS	house mouse	IGKV5-43
L2-7-A	CDRL2	2AJV	L	LMSTRAS	house mouse	IGKV2-112
L2-7-A	CDRL2	4AEI	N	KVSNRFS	house mouse	IGKV1-117
L2-7-A	CDRL2	2XRA	L	DASNLAT	human	IGKV1-33
L2-7-A	CDRL2	2IPT	L	KVSNRFS	house mouse	IGKV1-117
L2-7-A	CDRL2	4DQO	L	DVSHRPS	human	IGLV2-14
L2-7-A	CDRL2	4DKF	M	GASSRAS	human	IGKV1-39
L2-7-A	CDRL2	3QHZ	L	GNNERPS	human	IGLV1-44
L2-7-A	CDRL2	2FJF	J	SASFLYS	human	IGKV1-39
L2-7-A	CDRL2	1RUM	L	LGTKLDS	house mouse	IGKV1-133
L2-7-A	CDRL2	4HII	C	GASNRAT	human	IGKV3-11
L2-7-A	CDRL2	3RVX	C	RANRLIT	house mouse	IGKV14-111
L2-7-A	CDRL2	3C6S	A	HLSNLAS	house mouse	IGKV2-109
L2-7-A	CDRL2	4LEO	B	WASTRES	human	IGKV4-1
L2-7-A	CDRL2	4HGW	A	WASTRES	house mouse	IGKV8-21
L2-7-A	CDRL2	1KIQ	A	YTTTLAD	house mouse	IGKV12-41
L2-7-A	CDRL2	2R56	M	AASSLQS	human	IGKV1D-16
L2-7-A	CDRL2	3BN9	C	AASSLQS	human	IGKV1-12
L2-7-A	CDRL2	3GBM	L	DNNKRPS	human	IGLV1-51

L2-7-A	CDRL2	2FAT	L	EISKLAS	house mouse	IGKV4-86
L2-7-A	CDRL2	3EOA	L	SGSTLQS	human	IGKV1-16
L2-7-A	CDRL2	4LLW	B	SASYRYT	human	IGKV1-16
L2-7-A	CDRL2	1MFA	L	DTNNRAP	house mouse	IGLV1
L2-7-A	CDRL2	1JFQ	L	FTSRSQS	house mouse	IGKV10-96
L2-7-A	CDRL2	3ZE2	F	YGTNLVD	house mouse	IGKV14-100
L2-7-A	CDRL2	1BFO	C	NTNNLQT	Norway rat	IGKV22S7
L2-7-A	CDRL2	4GMT	M	SASNRYS	house mouse	IGKV6-15
L2-7-A	CDRL2	1YEE	L	LVSKLDS	house mouse	IGKV1-133
L2-7-A	CDRL2	1ZLV	L	KASTLKT	human	IGKV1-5
L2-7-A	CDRL2	2YKL	L	RNNQRPS	human	IGLV1-47
L2-7-A	CDRL2	1UJ3	A	YATSLAD	human	IGKV1D-16
L2-7-A	CDRL2	4GAG	L	LASNLNS	house mouse	IGKV3-10
L2-7-A	CDRL2	1MH5	A	RMSNLAS	house mouse	IGKV2-137
L2-7-A	CDRL2	4JY6	A	NNQDRPA	human	IGLV3-21
L2-7-A	CDRL2	4DVB	B	ATSNLAS	house mouse	IGKV4-57-1
L2-7-A	CDRL2	3SY0	A	WASTRES	house mouse	IGKV8-21
L2-7-A	CDRL2	4GW4	L	DGSKLER	human	IGKV1-33
L2-7-A	CDRL2	3MNZ	A	FASIRES	house mouse	IGKV8-24
L2-7-A	CDRL2	2XKN	C	YTSRLHS	house mouse	IGKV10-96
L2-7-A	CDRL2	3LIZ	L	RADRLVD	house mouse	IGKV14-111
L2-7-A	CDRL2	1OAU	N	GTNNRAP	house mouse	IGLV1
L2-7-A	CDRL2	4OCS	L	ENTRRPS	human	IGLV1-51
L2-7-A	CDRL2	4NZU	L	DASNLAI	human	IGKV1-33
L2-7-A	CDRL2	4AJ0	A	QDSKRPS	human	IGLV3-1
L2-7-A	CDRL2	3I75	A	YTSILRP	house mouse	IGKV19-93
L2-7-A	CDRL2	3U79	F	DASNLET	human	IGKV1-33
L2-7-A	CDRL2	3MLX	M	ENSKRPS	human	IGLV1-51
L2-7-A	CDRL2	3MCK	L	KVSNRFS	house mouse	IGKV1-117
L2-7-A	CDRL2	1RUQ	L	QMSKLAS	house mouse	IGKV2-109
L2-7-A	CDRL2	2HFF	A	SASFLYS	human	IGKV1-39
L2-7-A	CDRL2	1TQB	C	LVSRLDS	house mouse	IGKV1-135
L2-7-A	CDRL2	1QP1	A	DASTLET	human	IGKV1-33
L2-7-A	CDRL2	3I02	C	WASTRES	house mouse	IGKV8-21
L2-7-A	CDRL2	4ETQ	B	DTSNLAS	house mouse	IGKV4-55
L2-7-A	CDRL2	2OLD	B	EVTNRPS	human	IGLV2-14
L2-7-A	CDRL2	3GJE	L	DVIERSS	human	IGLV2-11
L2-7-A	CDRL2	2XQY	K	LASNLES	house mouse	IGKV3-12
L2-7-A	CDRL2	35C8	L	STSNLAS	house mouse	IGKV4-74
L2-7-A	CDRL2	3QPQ	I	NAKTLAD	house mouse	IGKV12-41
L2-7-A	CDRL2	4AIX	A	QDSKRPS	human	IGLV3-1
L2-7-A	CDRL2	3MUG	A	DVSHRPS	human	IGLV2-14
L2-7-A	CDRL2	1U8H	A	DASSLES	human	IGKV1D-13
L2-7-A	CDRL2	3QOT	L	WASTRES	human	IGKV4-1
L2-7-A	CDRL2	1Q0X	L	GTNNRAP	house mouse	IGLV1
L2-7-A	CDRL2	4HIE	A	GASNRAT	human	IGKV3-11
L2-7-A	CDRL2	1QP1	B	DASTLET	human	IGKV1-33
L2-7-A	CDRL2	1UA6	L	YASQSIS	house mouse	IGKV5-43
L2-7-A	CDRL2	2O5X	L	KVSNRFS	house mouse	IGKV1-110
L2-7-A	CDRL2	1EO8	L	DTSKLAS	house mouse	IGKV4-70

L2-7-A	CDRL2	3ZKN	L	LASNLES	house mouse	IGKV3-10
L2-7-A	CDRL2	1UZ6	V	QMSNLAS	house mouse	IGKV2-109
L2-7-A	CDRL2	2BX5	C	AASSLQS	human	IGKV1-39
L2-7-A	CDRL2	1YY8	A	YASESIS	house mouse	IGKV5-48
L2-7-A	CDRL2	3GK8	L	SASNRYT	house mouse	IGKV6-17
L2-7-A	CDRL2	4D9L	M	GNSNRPS	human	IGLV1-40
L2-7-A	CDRL2	3U79	A	DASNLET	human	IGKV1-33
L2-7-A	CDRL2	1DBM	L	KVSNRFY	house mouse	IGKV1-110
L2-7-A	CDRL2	1OM3	L	KASTLKT	human	IGKV1-5
L2-7-A	CDRL2	1Q9L	C	WASTRES	house mouse	IGKV8-21
L2-7-A	CDRL2	3K2U	L	SASFLYS	human	IGKV1D-13
L2-7-A	CDRL2	4JY4	A	NNQDRPS	human	IGLV3-21
L2-7-A	CDRL2	1ETZ	A	GTNNRVP	house mouse	IGLV1
L2-7-A	CDRL2	2DQU	L	KVSNRFS	house mouse	IGKV1-117
L2-7-A	CDRL2	4FNL	M	DASNLQR	human	IGKV1-33
L2-7-A	CDRL2	1C12	A	HGTNLED	house mouse	IGKV14-100
L2-7-A	CDRL2	1UZ6	L	QMSNLAS	house mouse	IGKV2-109
L2-7-A	CDRL2	3TCL	L	GGSTRAT	human	IGKV3-20
L2-7-A	CDRL2	3CVI	L	GATSLET	house mouse	IGKV13-84
L2-7-A	CDRL2	3KS0	L	GTNKRAP	house mouse	IGLV1
L2-7-A	CDRL2	4CAD	J	NAKTLAD	house mouse	IGKV12-41
L2-7-A	CDRL2	1EGJ	L	LASNLAS	house mouse	IGKV3-10
L2-7-A	CDRL2	1MRF	L	KVSNRFS	house mouse	IGKV1-110
L2-7-A	CDRL2	43C9	A	FASTRES	house mouse	IGKV8-24
L2-7-A	CDRL2	3QQ9	C	AASNPES	house mouse	IGKV3-3
L2-7-A	CDRL2	1MCJ	A	EVNKRPS	human	IGLV2-8
L2-7-A	CDRL2	1DVF	A	YTTTLAD	house mouse	IGKV12-41
L2-7-A	CDRL2	4AIZ	A	KDSERPS	human	IGLV3-25
L2-7-A	CDRL2	3G04	A	YDDQLPS	human	IGLV1-36
L2-7-A	CDRL2	2FJF	E	SASFLYS	human	IGKV1-39
L2-7-A	CDRL2	1FAI	L	YTSRLHS	house mouse	IGKV10-96
L2-7-A	CDRL2	4AT6	P	GTNNRAP	house mouse	IGLV1
L2-7-A	CDRL2	2DQD	L	YASQSIS	house mouse	IGKV5-43
L2-7-A	CDRL2	1A6U	L	GTNNRAP	house mouse	IGLV1
L2-7-A	CDRL2	1MPA	L	KVSNRFS	house mouse	IGKV1-110
L2-7-A	CDRL2	3L5Y	L	SGSTLQS	house mouse	IGKV12-44
L2-7-A	CDRL2	4GAJ	L	LASNLNS	house mouse	IGKV3-10
L2-7-A	CDRL2	2IH3	B	YASESIS	house mouse	IGKV5-48
L2-7-A	CDRL2	3CFD	A	YTSRLHS	house mouse	IGKV10-96
L2-7-A	CDRL2	3AAZ	B	QVSTRFS	house mouse	IGKV
L2-7-A	CDRL2	1OAZ	N	GTNNRAP	house mouse	IGLV1
L2-7-A	CDRL2	3UTZ	E	KVSNRFS	house mouse	IGKV1-117
L2-7-A	CDRL2	3OAY	K	KASTLKT	human	IGKV1-5
L2-7-A	CDRL2	3NH7	L	DDSNRPS	human	IGLV3-1
L2-7-A	CDRL2	2CK0	L	WASTRGS	house mouse	IGKV8-21
L2-7-A	CDRL2	1OAR	M	GTNNRAP	house mouse	IGLV1
L2-7-A	CDRL2	3GJF	K	DVIERSS	human	IGLV2-11
L2-7-A	CDRL2	3FO9	L	KVSNRFS	house mouse	IGKV1-110
L2-7-A	CDRL2	3T0W	A	NDDRRSS	human	IGLV1-47
L2-7-A	CDRL2	3PNW	M	SASSLYS	human	IGKV1-39

L2-7-A	CDRL2	2BX5	F	AASSLQS	human	IGKV1-39
L2-7-A	CDRL2	1YY8	C	YASESIS	house mouse	IGKV5-48
L2-7-A	CDRL2	2VXU	L	ATSSLDS	house mouse	IGKV9-120
L2-7-A	CDRL2	1M7D	A	KVSNRFS	house mouse	IGKV1-110
L2-7-A	CDRL2	4LAS	L	STSNLAS	house mouse	IGKV4-79
L2-7-A	CDRL2	1ZLU	L	KASTLKT	human	IGKV1-5
L2-7-A	CDRL2	1BJ1	J	FTSSLHS	human	IGKV1-33
L2-7-A	CDRL2	2DQT	L	KVSNRFS	house mouse	IGKV1-117
L2-7-A	CDRL2	3STL	B	YASESIS	house mouse	IGKV5-48
L2-7-A	CDRL2	2NLJ	A	YASESIS	house mouse	IGKV5-48
L2-7-A	CDRL2	1KB5	L	NAKTLGE	house mouse	IGKV12-44
L2-7-A	CDRL2	2VL5	D	RASNLES	house mouse	IGKV3-5
L2-7-A	CDRL2	3QQ9	L	AASNPES	house mouse	IGKV3-3
L2-7-A	CDRL2	1UWX	K	AATSLAD	house mouse	IGKV12-98
L2-7-A	CDRL2	3NIG	L	YGTNLVD	house mouse	IGKV14-100
L2-7-A	CDRL2	2AI0	O	KVSNRFS	house mouse	IGKV1-117
L2-7-A	CDRL2	3BZ4	C	HLSNLAS	house mouse	IGKV2-109
L2-7-A	CDRL2	3CLE	L	LASYRYS	house mouse	IGKV6-15
L2-7-A	CDRL2	1C5B	L	DATKLDS	house mouse	IGKV9-124
L2-7-A	CDRL2	4JDV	L	DASNRAT	human	IGKV3-11
L2-7-A	CDRL2	4K3H	B	ETDKKYS	human	IGLV7-46
L2-7-A	CDRL2	4JZO	E	QDNKRPS	human	IGLV3-1
L2-7-A	CDRL2	3CFJ	C	GTNKRAP	house mouse	IGLV1
L2-7-A	CDRL2	1G9M	L	GASTRAT	human	IGKV3-15
L2-7-A	CDRL2	1IGT	A	KASNLHT	house mouse	IGKV10-94
L2-7-A	CDRL2	2VDL	L	YGTNLVD	house mouse	IGKV14-100
L2-7-A	CDRL2	3G5Z	A	HGTNLSD	house mouse	IGKV14-100
L2-7-A	CDRL2	1FE8	N	YTSRLHS	house mouse	IGKV10-96
L2-7-A	CDRL2	3HI6	L	AASSLQS	human	IGKV1-39
L2-7-A	CDRL2	2R0L	L	SASFLYS	human	IGKV1-39
L2-7-A	CDRL2	1BRE	E	DASTLET	human	IGKV1-33
L2-7-A	CDRL2	3NPS	C	DNNQRPS	human	IGLV1-44
L2-7-A	CDRL2	3DVF	A	DASNLET	human	IGKV1-33
L2-7-A	CDRL2	1MCL	B	EVNKRPS	human	IGLV2-8
L2-7-A	CDRL2	1BOG	A	RANRLMI	house mouse	IGKV14-111
L2-7-A	CDRL2	3CFJ	A	GTNKRAP	house mouse	IGLV1
L2-7-A	CDRL2	1BVK	A	YTTTLAD	human	IGKV1-27
L2-7-A	CDRL2	4AIZ	C	KDSERPS	human	IGLV3-25
L2-7-A	CDRL2	4AJ0	C	QDSKRPS	human	IGLV3-1
L2-7-A	CDRL2	2FR4	L	NAKTLAE	house mouse	IGKV12-44
L2-7-A	CDRL2	1JGV	L	KVSNRFS	house mouse	IGKV1-110
L2-7-A	CDRL2	2EIZ	A	YASQSIS	human	IGKV3-15
L2-7-A	CDRL2	3LEY	L	LVSKLAS	house mouse	IGKV1-135
L2-7-A	CDRL2	3V4U	L	KVSNRFS	house mouse	IGKV1-110
L2-7-A	CDRL2	1NMC	C	YTSNLHS	house mouse	IGKV10-96
L2-7-A	CDRL2	2LVE	A	WASTRES	human	IGKV4-1
L2-7-A	CDRL2	3PNW	G	SASSLYS	human	IGKV1-39
L2-7-A	CDRL2	4JZN	D	DDSDRPS	human	IGLV3-21
L2-7-A	CDRL2	1UB5	B	QMSNLAS	house mouse	IGKV2-109
L2-7-A	CDRL2	1A3L	L	QMSKLAS	house mouse	IGKV2-109

L2-7-A	CDRL2	2HVK	B	YASESIS	house mouse	IGKV5-48
L2-7-A	CDRL2	1UWE	U	STSYRYS	house mouse	IGKV6-15
L2-7-A	CDRL2	1LHZ	B	EVNKRPS	human	IGLV2-23
L2-7-A	CDRL2	3MLT	L	QDFKRPS	human	IGLV3-1
L2-7-A	CDRL2	2RCS	L	AASTLDS	house mouse	IGKV9-124
L2-7-A	CDRL2	3QNY	A	LGSTRAS	human	IGKV2-28
L2-7-A	CDRL2	2MCG	1	EVNKRPS	human	IGLV2-8
L2-7-A	CDRL2	3U79	B	DASNLET	human	IGKV1-33
L2-7-A	CDRL2	1TZH	A	AASYLYS	house mouse	IGKV12-44
L2-7-A	CDRL2	12E8	L	SASNRYT	house mouse	IGKV6-13
L2-7-A	CDRL2	1NMB	L	YTSNLHS	house mouse	IGKV10-96
L2-7-A	CDRL2	1G7H	A	YTTTLAD	house mouse	IGKV12-41
L2-7-A	CDRL2	1NGW	A	SASNRYT	house mouse	IGKV6-13
L2-7-A	CDRL2	1IGF	L	KVSNRFS	house mouse	IGKV1-117
L2-7-A	CDRL2	2XQY	L	LASNLES	house mouse	IGKV3-12
L2-7-A	CDRL2	1CFQ	A	RANRLMI	house mouse	IGKV14-111
L2-7-A	CDRL2	1IGI	L	KVSNRFS	house mouse	IGKV1-110
L2-7-A	CDRL2	2FJF	L	SASFLYS	human	IGKV1-39
L2-7-A	CDRL2	1LHZ	A	EVNKRPS	human	IGLV2-23
L2-7-A	CDRL2	3TNM	B	EVNNRPS	human	IGLV2-8
L2-7-A	CDRL2	3U6R	B	DASNRAT	human	IGKV3-11
L2-7-A	CDRL2	2W0L	D	EDNQRPS	human	IGLV6-57
L2-7-A	CDRL2	1IL1	B	WASTRES	house mouse	IGKV8-30
L2-7-A	CDRL2	2DWD	B	YASESIS	house mouse	IGKV5-48
L2-7-A	CDRL2	1YYM	Q	GASTRAT	human	IGKV3-15
L2-7-A	CDRL2	1FE8	L	YTSRLHS	house mouse	IGKV10-96
L2-7-A	CDRL2	3PNW	J	SASSLYS	human	IGKV1-39
L2-7-A	CDRL2	4MA7	L	YASESIS	house mouse	IGKV5-48
L2-7-A	CDRL2	3MXW	L	YASNRYT	human	IGKV1-33
L2-7-A	CDRL2	4AT6	I	GTNNRAP	house mouse	IGLV1
L2-7-A	CDRL2	1NBZ	A	YASQSIS	house mouse	IGKV5-43
L2-7-A	CDRL2	3SGD	L	LVSKLDS	house mouse	IGKV1-135
L2-7-A	CDRL2	2FB4	L	RDAMRPS	human	IGLV1-44
L2-7-A	CDRL2	3B2U	G	DASNRAT	human	IGKV3D-11
L2-7-A	CDRL2	2IMM	A	GASTRES	house mouse	IGKV8-28
L2-7-A	CDRL2	3OAY	L	KASTLKT	human	IGKV1-5
L2-7-A	CDRL2	1A7P	L	YTTTLAD	house mouse	IGKV12-41
L2-7-A	CDRL2	2Q1E	C	DASTLET	human	IGKV1-33
L2-7-A	CDRL2	3U9P	M	YTSTLES	Norway rat	IGKV19S2
L2-7-A	CDRL2	2FJF	M	SASFLYS	human	IGKV1-39
L2-7-A	CDRL2	4HHA	A	DASNRAT	human	IGKV3-11
L2-7-A	CDRL2	3BKC	L	QVSTRFS	house mouse	IGKV1-117
L2-7-A	CDRL2	1NAK	M	KVSNRFS	house mouse	IGKV1-110
L2-7-A	CDRL2	3L5X	L	SGSTLQS	human	IGKV3-11
L2-7-A	CDRL2	3PP3	K	QMSNLVS	house mouse	IGKV
L2-7-A	CDRL2	3QG6	A	KLSSRFS	house mouse	IGKV1-110
L2-7-A	CDRL2	1WEJ	L	NAKTLAD	house mouse	IGKV12-41
L2-7-A	CDRL2	2FBJ	L	EISKLAS	house mouse	IGKV4-86
L2-7-A	CDRL2	3BZ4	A	HLSNLAS	house mouse	IGKV2-109
L2-7-A	CDRL2	1MCN	B	EVNKRPS	human	IGLV2-8

L2-7-A	CDRL2	1DSF	L	KVSNRFS	house mouse	IGKV1-110
L2-7-A	CDRL2	1FDL	L	YTTTLAD	house mouse	IGKV12-41
L2-7-A	CDRL2	2OSL	L	ATSNLAS	house mouse	IGKV4-72
L2-7-A	CDRL2	1FL5	A	KVSNRFS	house mouse	IGKV1-117
L2-7-A	CDRL2	2XQB	L	RDRRRPS	human	IGLV1-47
L2-7-A	CDRL2	1F3D	J	KVSKRFS	house mouse	IGKV1-117
L2-7-A	CDRL2	4OCR	L	KNDNRPS	human	IGLV1-51
L2-7-A	CDRL2	1LO3	X	KVSNRFS	house mouse	IGKV1-117
L2-7-A	CDRL2	1KEM	L	KVSNRFS	house mouse	IGKV1-117
L2-7-A	CDRL2	3EHB	D	NAKTLGE	house mouse	IGKV12-44
L2-7-A	CDRL2	1A6V	L	GTNNRAP	house mouse	IGLV1
L2-7-A	CDRL2	1FL6	L	KVSNRFS	house mouse	IGKV1-117
L2-7-A	CDRL2	1N0X	M	GVSNRAS	human	IGKV3-20
L2-7-A	CDRL2	4M5Z	L	DDSDRPS	human	IGLV3-21
L2-7-A	CDRL2	1A5F	L	WASTRES	house mouse	IGKV8-19
L2-7-A	CDRL2	4N9G	B	YTSHLES	human	IGKV1-16
L2-7-A	CDRL2	4AIX	C	QDSKRPS	human	IGLV3-1
L2-7-A	CDRL2	4FQH	L	SNDQRPS	human	IGLV1-44
L2-7-A	CDRL2	3QOS	L	WASTRES	human	IGKV4-1
L2-7-A	CDRL2	3LRH	O	DDDLLAP	human	IGLV1-36
L2-7-A	CDRL2	2YPV	L	RANRLVD	house mouse	IGKV14-111
L2-7-A	CDRL2	1NFD	G	MDNKRPS	hamster	IGLV
L2-7-A	CDRL2	2OR9	M	AISNRGS	house mouse	IGKV3-2
L2-7-A	CDRL2	2XWT	B	DNNKRPS	human	IGLV1-51
L2-7-A	CDRL2	1RZJ	L	GASTRAT	human	IGKV3-15
L2-7-A	CDRL2	3T2N	L	DTNNRAP	human	IGLV7-43
L2-7-A	CDRL2	3LRH	A	DDDLLAP	human	IGLV1-36
L2-7-A	CDRL2	1E6O	L	EISKLAS	house mouse	IGKV4-86
L2-7-A	CDRL2	4HCR	L	EVSNRFS	house mouse	IGKV
L2-7-A	CDRL2	1A8J	L	EVNKRPS	human	IGLV2-8
L2-7-A	CDRL2	3FO2	L	KVSNRFS	house mouse	IGKV1-117
L2-7-A	CDRL2	4AG4	L	LTSNLAS	house mouse	IGKV4-68
L2-7-A	CDRL2	2Z91	B	DTSKLPS	house mouse	IGKV4-59
L2-7-A	CDRL2	3W9D	B	GASNRAT	house mouse	IGKV1-117
L2-7-A	CDRL2	3ZDX	L	YGTNLVD	house mouse	IGKV14-100
L2-7-A	CDRL2	1MCJ	B	EVNKRPS	human	IGLV2-8
L2-7-A	CDRL2	3GBN	L	DNNKRPS	human	IGLV1-51
L2-7-A	CDRL2	3B2U	O	DASNRAT	human	IGKV3D-11
L2-7-A	CDRL2	2QSC	L	TASNLET	human	IGKV1-33
L2-7-A	CDRL2	3RKD	C	SATNLAE	house mouse	IGKV12-46
L2-7-A	CDRL2	1OAK	L	YTSSLHS	house mouse	IGKV10-94
L2-7-A	CDRL2	2AJU	L	LMSTRAS	house mouse	IGKV2-112
L2-7-A	CDRL2	1DQQ	C	YASQSIS	house mouse	IGKV5-43
L2-7-A	CDRL2	4LLW	D	SASYRYT	human	IGKV1-16
L2-7-A	CDRL2	4HXB	L	KVSNRFP	house mouse	IGKV1-117
L2-7-A	CDRL2	2Z4Q	A	KVSDRFS	house mouse	IGKV1-117
L2-7-A	CDRL2	2PR4	L	DASSLES	human	IGKV1D-13
L2-7-A	CDRL2	4AIX	D	QDSKRPS	human	IGLV3-1
L2-7-A	CDRL2	1YNL	L	RVSNRFS	house mouse	IGKV1-110
L2-7-A	CDRL2	1KEG	L	KVSNRFS	house mouse	IGKV1-117

L2-7-A	CDRL2	3MLY	L	ENSKRPS	human	IGLV1-51
L2-7-A	CDRL2	1Y18	C	GTNKRAP	house mouse	IGLV1
L2-7-A	CDRL2	3OR6	B	YASESIS	house mouse	IGKV5-48
L2-7-A	CDRL2	4HIX	L	LVSKLDS	house mouse	IGKV
L2-7-A	CDRL2	3MLS	O	QDFKRPS	human	IGLV3-1
L2-7-A	CDRL2	3DV4	A	WASTRES	house mouse	IGKV8-21
L2-7-A	CDRL2	1YYM	L	GASTRAT	human	IGKV3-15
L2-7-A	CDRL2	2FJF	W	SASFLYS	human	IGKV1-39
L2-7-A	CDRL2	2R0Z	L	KVSNRFS	house mouse	IGKV1-117
L2-7-A	CDRL2	3NFS	L	TTSNLAS	human	IGKV1-5
L2-7-A	CDRL2	3DUU	A	WASTRES	house mouse	IGKV8-21
L2-7-A	CDRL2	1IGF	M	KVSNRFS	house mouse	IGKV1-117
L2-7-A	CDRL2	2BX5	A	AASSLQS	human	IGKV1-39
L2-7-A	CDRL2	1QBL	L	NAKTLAD	house mouse	IGKV12-41
L2-7-A	CDRL2	43C9	G	FASTRES	house mouse	IGKV8-24
L2-7-A	CDRL2	3UO1	L	KVSNRFS	house mouse	IGKV1-110
L2-7-A	CDRL2	4HH9	A	DASNRAT	human	IGKV3-11
L2-7-A	CDRL2	1VFB	A	YTTTLAD	house mouse	IGKV12-41
L2-7-A	CDRL2	2Q1E	D	DASTLET	human	IGKV1-33
L2-7-A	CDRL2	2NYY	C	RASNLEP	house mouse	IGKV3-5
L2-7-A	CDRL2	3DIF	A	SASYRYT	house mouse	IGKV6-17
L2-7-A	CDRL2	3VG9	B	AATNLAD	house mouse	IGKV12-46
L2-7-A	CDRL2	1RIU	L	RVSNLAS	house mouse	IGKV2-137
L2-7-A	CDRL2	3U7W	L	SGSTRAA	human	IGKV3-11
L2-7-A	CDRL2	3V6F	L	WASTRES	house mouse	IGKV8-27
L2-7-A	CDRL2	2W60	B	LVSKLDS	house mouse	IGKV1-135
L2-7-A	CDRL2	3LH2	O	GASSRPS	human	IGKV3-20
L2-7-A	CDRL2	2Z91	D	DTSKLPS	house mouse	IGKV4-59
L2-7-A	CDRL2	1UB6	L	QMSNLAS	house mouse	IGKV2-109
L2-7-A	CDRL2	4FQQ	L	DDSDRPS	human	IGLV3-21
L2-7-A	CDRL2	3MLT	D	QDFKRPS	human	IGLV3-1
L2-7-A	CDRL2	4K23	L	LVSKLDS	house mouse	IGKV1-135
L2-7-A	CDRL2	1HIL	C	WASTRES	house mouse	IGKV8-19
L2-7-A	CDRL2	1JPS	L	YATSLAE	human	IGKV1-39
L2-7-A	CDRL2	4LAQ	L	STSNLAS	house mouse	IGKV4-79
L2-7-A	CDRL2	2EH8	L	LVSKLDS	house mouse	IGKV1-133
L2-7-A	CDRL2	2CD0	B	EDDHRPS	human	IGLV6-57
L2-7-A	CDRL2	3QCU	L	KVSNLFS	house mouse	IGKV
L2-7-A	CDRL2	4KUC	D	LGSNLES	house mouse	IGKV3-12
L2-7-A	CDRL2	2ADF	L	YTSTLQP	house mouse	IGKV19-93
L2-7-A	CDRL2	2H1P	L	KVSNRFS	house mouse	IGKV1-110
L2-7-A	CDRL2	3IDJ	A	DASSLES	human	IGKV1-13
L2-7-A	CDRL2	4JO2	L	DASKLAS	rabbit	IGKV1S5
L2-7-A	CDRL2	1MFD	L	DTNNRAP	house mouse	IGLV1
L2-7-A	CDRL2	1DQL	L	AASSLQS	human	IGKV1-6
L2-7-A	CDRL2	3IFN	L	KVSNRFS	house mouse	IGKV1-117
L2-7-A	CDRL2	43C9	E	FASTRES	house mouse	IGKV8-24
L2-7-A	CDRL2	3QPQ	L	NAKTLAD	house mouse	IGKV12-41
L2-7-A	CDRL2	1IGJ	A	KVSNRFS	house mouse	IGKV1-110
L2-7-A	CDRL2	2OMN	B	EVTNRPS	human	IGLV2-14

L2-7-A	CDRL2	4HT1	L	TASYLAS	rabbit	IGKV1S3
L2-7-A	CDRL2	3T77	A	WASTRES	house mouse	IGKV8-21
L2-7-A	CDRL2	3MOB	L	DASSLES	human	IGKV1-13
L2-7-A	CDRL2	3LRG	B	DDDLLAP	human	IGLV1-36
L2-7-A	CDRL2	3U2S	L	DVSKRPS	human	IGLV2-14
L2-7-A	CDRL2	1A7N	L	YTTTLAD	house mouse	IGKV12-41
L2-7-A	CDRL2	1UWE	X	STSYRYS	house mouse	IGKV6-15
L2-7-A	CDRL2	1MFE	L	DTNNRAP	house mouse	IGLV1
L2-7-A	CDRL2	4JAM	B	ENYKRPS	human	IGLV3-1
L2-7-A	CDRL2	1LO2	X	KVSNRFS	house mouse	IGKV1-117
L2-7-A	CDRL2	3BAE	L	QVSTRFS	house mouse	IGKV1-117
L2-7-A	CDRL2	1DBJ	L	KVSNRFY	house mouse	IGKV1-110
L2-7-A	CDRL2	1F4X	L	ATNNRAA	house mouse	IGLV1
L2-7-A	CDRL2	3OR7	B	YASESIS	house mouse	IGKV5-48
L2-7-A	CDRL2	1UAC	L	YASQSIS	house mouse	IGKV5-43
L2-7-A	CDRL2	2BX5	M	AASSLQS	human	IGKV1-39
L2-7-A	CDRL2	4LKC	A	DASSLES	human	IGKV1-13
L2-7-A	CDRL2	1J1P	L	YASQSIS	house mouse	IGKV5-43
L2-7-A	CDRL2	1H0D	A	AASNQGS	house mouse	IGKV3-2
L2-7-A	CDRL2	1MNU	L	KVSNRFS	house mouse	IGKV1-110
L2-7-A	CDRL2	3V6O	E	LASNRHT	house mouse	IGKV6-14
L2-7-A	CDRL2	4AT6	L	GTNNRAP	house mouse	IGLV1
L2-7-A	CDRL2	4FQI	L	SNDQRPS	human	IGLV1-44
L2-7-A	CDRL2	4AJ0	D	QDSKRPS	human	IGLV3-1
L2-7-A	CDRL2	1RHH	A	GASTRAT	human	IGKV3-20
L2-7-A	CDRL2	1HH6	A	RANRLMI	house mouse	IGKV14-111
L2-7-A	CDRL2	1CT8	A	FASQSIP	house mouse	IGKV5-43
L2-7-A	CDRL2	3SGD	I	LVSKLDS	house mouse	IGKV1-135
L2-7-A	CDRL2	3GNM	L	KVSNRFS	house mouse	IGKV1-117
L2-7-A	CDRL2	4CAD	A	NAKTLAD	house mouse	IGKV12-41
L2-7-A	CDRL2	1Q0Y	L	GTNNRAP	house mouse	IGLV1
L2-7-A	CDRL2	2AAB	L	AASNVES	house mouse	IGKV3-1
L2-7-A	CDRL2	3LMJ	L	DDTQRPS	human	IGLV1-51
L2-7-A	CDRL2	2Y06	L	GTNNRAP	house mouse	IGLV1
L2-7-A	CDRL2	1G7I	A	YTTTLAD	house mouse	IGKV12-41
L2-7-A	CDRL2	3MLT	A	QDFKRPS	human	IGLV3-1
L2-7-A	CDRL2	1BRE	F	DASTLET	human	IGKV1-33
L2-7-A	CDRL2	4K3H	A	ETDKKYS	human	IGLV7-46
L2-7-A	CDRL2	1FRG	L	WASTRES	house mouse	IGKV8-19
L2-7-A	CDRL2	3V6F	D	WASTRES	house mouse	IGKV8-27
L2-7-A	CDRL2	2DQH	L	YASQSIS	house mouse	IGKV5-43
L2-7-A	CDRL2	3QOS	A	WASTRES	human	IGKV4-1
L2-7-A	CDRL2	3TCL	B	GGSTRAT	human	IGKV3-20
L2-7-A	CDRL2	3KYM	A	DASNRAT	human	IGKV3-15
L2-7-A	CDRL2	4M61	C	WASTRES	house mouse	IGKV8-27
L2-7-A	CDRL2	4JAM	L	ENYKRPS	human	IGLV3-1
L2-7-A	CDRL2	1NGQ	L	GTNNRAP	house mouse	IGLV1
L2-7-A	CDRL2	1OAR	O	GTNNRAP	house mouse	IGLV1
L2-7-A	CDRL2	1MJU	L	RMSNLAS	house mouse	IGKV2-137
L2-7-A	CDRL2	1GHF	L	YTSRLHS	house mouse	IGKV10-96

L2-7-A	CDRL2	1BZ7	A	YTSRLQS	house mouse	IGKV10-96
L2-7-A	CDRL2	3NID	L	YGTNLVD	house mouse	IGKV14-100
L2-7-A	CDRL2	4GW1	A	YASESIS	house mouse	IGKV5-48
L2-7-A	CDRL2	2OQJ	D	KASTLKT	human	IGKV1-5
L2-7-A	CDRL2	1XF5	A	WASTRES	house mouse	IGKV8-21
L2-7-A	CDRL2	3CXD	L	AATNLAD	house mouse	IGKV12-46
L2-7-A	CDRL2	1BRE	A	DASTLET	human	IGKV1-33
L2-7-A	CDRL2	3V0V	L	KASNLHT	house mouse	IGKV11-125
L2-7-A	CDRL2	3HB3	D	NAKTLGE	house mouse	IGKV12-44
L2-7-A	CDRL2	2MCG	2	EVNKRPS	human	IGLV2-8
L2-7-A	CDRL2	1C08	A	YASQSIS	house mouse	IGKV5-43
L2-7-A	CDRL2	3QO1	A	LGSTRAS	human	IGKV2-28
L2-7-A	CDRL2	2HFE	B	YASESIS	house mouse	IGKV5-48
L2-7-A	CDRL2	3QO0	A	LGSTRAS	human	IGKV2-28
L2-7-A	CDRL2	4LLV	L	GASSRPS	human	IGKV3-20
L2-7-A	CDRL2	1WZ1	L	KVSNRFS	house mouse	IGKV1-110
L2-7-A	CDRL2	2AI0	M	KVSNRFS	house mouse	IGKV1-117
L2-7-A	CDRL2	1U8N	A	DASSLES	human	IGKV1D-13
L2-7-A	CDRL2	1BQL	L	DTSKLAS	house mouse	IGKV4-59
L2-7-A	CDRL2	3V6F	F	WASTRES	house mouse	IGKV8-27
L2-7-A	CDRL2	2Q76	A	YTSQSMS	house mouse	IGKV5-43
L2-7-A	CDRL2	3CDF	F	DASNLET	human	IGKV1-33
L2-7-A	CDRL2	1SM3	L	GTNNRAP	house mouse	IGLV1
L2-7-A	CDRL2	1R3K	A	YASESIS	house mouse	IGKV5-48
L2-7-A	CDRL2	2PCP	A	KVTNRFS	house mouse	IGKV1-117
L2-7-A	CDRL2	4J8R	C	WASTRHT	house mouse	IGKV6-23
L2-7-A	CDRL2	1MCF	B	EVNKRPS	human	IGLV2-8
L2-7-A	CDRL2	3QEH	D	LGSNRAT	house mouse	IGKV
L2-7-A	CDRL2	1BFO	E	NTNNLQT	Norway rat	IGKV22S7
L2-7-A	CDRL2	2D7T	L	AASNLQG	human	IGKV1-6
L2-7-A	CDRL2	3QCU	M	KVSNLFS	house mouse	IGKV
L2-7-A	CDRL2	3T2N	M	DTNNRAP	human	IGLV7-43
L2-7-A	CDRL2	3T3P	L	YGTNLVD	house mouse	IGKV14-100
L2-7-A	CDRL2	2OQJ	A	KASTLKT	human	IGKV1-5
L2-7-A	CDRL2	2BMK	A	STSNLAS	house mouse	IGKV4-57
L2-7-A	CDRL2	3U0W	L	YTSSLHS	house mouse	IGKV10-94
L2-7-A	CDRL2	1EEU	A	WASTRES	human	IGKV4-1
L2-7-A	CDRL2	2HWZ	L	DTSKLAS	house mouse	IGKV1-117
L2-7-A	CDRL2	3GHB	M	GNNKRPS	human	IGLV1-51
L2-7-A	CDRL2	3MLY	M	ENSKRPS	human	IGLV1-51
L2-7-A	CDRL2	4K3G	A	ETDKKYS	human	IGLV7-46
L2-7-A	CDRL2	2AJ3	E	SASTLQS	human	IGKV1-9
L2-7-A	CDRL2	1OM3	M	KASTLKT	human	IGKV1-5
L2-7-A	CDRL2	1N4X	L	KVSNRFS	house mouse	IGKV1-117
L2-7-A	CDRL2	3CDC	A	DASNLET	human	IGKV1-33
L2-7-A	CDRL2	1LO0	X	KVSNRFS	house mouse	IGKV1-117
L2-7-A	CDRL2	1JHL	L	SGSTLQS	house mouse	IGKV16-104
L2-7-A	CDRL2	3LRH	I	DDDLLAP	human	IGLV1-36
L2-7-A	CDRL2	2HRP	L	AASNQGS	house mouse	IGKV3-2
L2-7-A	CDRL2	1K4C	B	YASESIS	house mouse	IGKV5-48

L2-7-A	CDRL2	3MLT	G	QDFKRPS	human	IGLV3-1
L2-7-A	CDRL2	1QP1	C	DASTLET	human	IGKV1-33
L2-7-A	CDRL2	4JO1	L	DASKLAS	rabbit	IGKV1S5
L2-7-A	CDRL2	2OQJ	J	KASTLKT	human	IGKV1-5
L2-7-A	CDRL2	1JGU	L	KVSNRFS	house mouse	IGKV1-110
L2-7-A	CDRL2	4HIJ	C	GASNRAT	human	IGKV3-11
L2-7-A	CDRL2	3QPX	L	YTDRLQT	Norway rat	IGKV22S9
L2-7-A	CDRL2	1XF4	A	NAKTLAE	house mouse	IGKV12-44
L2-7-A	CDRL2	1HKL	L	AASTLHS	house mouse	IGKV9-124
L2-7-A	CDRL2	4JM4	L	ETYSKIA	human	IGKV3-15
L2-7-A	CDRL2	1WT5	C	KVSDRFS	house mouse	IGKV
L2-7-A	CDRL2	1MCC	A	EVNKRPS	human	IGLV2-8
L2-7-A	CDRL2	1IKF	L	YTSRLRS	house mouse	IGKV10-96
L2-7-A	CDRL2	1M7I	A	KVSNRFS	house mouse	IGKV1-110
L2-7-A	CDRL2	4G7V	L	SASSLYS	human	IGKV1-5
L2-7-A	CDRL2	2UYL	X	KVSNRFS	house mouse	IGKV1-117
L2-7-A	CDRL2	2Y6S	L	LVSNLES	house mouse	IGKV3-12
L2-7-A	CDRL2	2VXS	O	ANNQRPS	human	IGLV6-57
L2-7-A	CDRL2	1FVD	A	SASFLES	human	IGKV1-39
L2-7-A	CDRL2	1UM5	L	AGSTLDS	house mouse	IGKV9-124
L2-7-A	CDRL2	1KC5	L	GASNRYT	house mouse	IGKV6-20
L2-7-A	CDRL2	2UYL	A	KVSNRFS	house mouse	IGKV1-117
L2-7-A	CDRL2	1LGV	A	EVNKRPS	human	IGLV2-23
L2-7-A	CDRL2	2VXS	L	ANNQRPS	human	IGLV6-57
L2-7-A	CDRL2	4F58	L	DVTNRPS	human	IGLV2-14
L2-7-A	CDRL2	1U8M	A	DASSLES	human	IGKV1D-13
L2-7-A	CDRL2	3CFK	C	GTNKRAP	house mouse	IGLV1
L2-7-A	CDRL2	3LH2	M	GASSRPS	human	IGKV3-20
L2-7-A	CDRL2	3KYM	M	DASNRAT	human	IGKV3-15
L2-7-A	CDRL2	2W0F	B	YASESIS	house mouse	IGKV5-48
L2-7-A	CDRL2	1T66	L	KVSNRFS	house mouse	IGKV1-110
L2-7-A	CDRL2	3TNN	F	EVSERPS	human	IGLV2-23
L2-7-A	CDRL2	3KYM	K	DASNRAT	human	IGKV3-15
L2-7-A	CDRL2	3U4E	L	DVSKRPS	human	IGLV2-14
L2-7-A	CDRL2	3O41	B	AASNPES	house mouse	IGKV3-3
L2-7-A	CDRL2	3ZDY	L	YGTNLVD	house mouse	IGKV14-100
L2-7-A	CDRL2	2FJG	L	SASFLYS	human	IGKV1-39
L2-7-A	CDRL2	1IGC	L	GASNRYT	house mouse	IGKV6-20
L2-7-A	CDRL2	1NBY	A	YASQSIS	house mouse	IGKV5-43
L2-7-A	CDRL2	3MNV	A	FASIRES	house mouse	IGKV8-24
L2-7-A	CDRL2	1G7J	A	YTTTLAD	house mouse	IGKV12-41
L2-7-A	CDRL2	4BJL	A	EDNSRAS	human	IGLV1-44
L2-7-A	CDRL2	4NRZ	B	GALNLQS	human	IGKV1-39
L2-7-A	CDRL2	4HK0	D	DDSDRPS	human	IGLV3-21
L2-7-A	CDRL2	1MQK	L	NAKTLGE	house mouse	IGKV12-44
L2-7-A	CDRL2	3L7E	A	SGSTLQS	house mouse	IGKV16-104
L2-7-A	CDRL2	2HMI	C	YTSSLHS	house mouse	IGKV10-94
L2-7-A	CDRL2	3KYM	I	DASNRAT	human	IGKV3-15
L2-7-A	CDRL2	4K3E	M	GDTSRAS	sheep	IGLV1S8
L2-7-A	CDRL2	3IKC	C	WASTRES	house mouse	IGKV8-21

L2-7-A	CDRL2	1VGE	L	DASNLES	human	IGKV1-13
L2-7-A	CDRL2	2IH1	B	YASESIS	house mouse	IGKV5-48
L2-7-A	CDRL2	1MHP	Y	LTSNLAS	human	IGKV1D-13
L2-7-A	CDRL2	1BLN	C	KISNRFS	house mouse	IGKV1-117
L2-7-A	CDRL2	3LRH	M	DDDLLAP	human	IGLV1-36
L2-7-A	CDRL2	2P7T	B	YASESIS	house mouse	IGKV5-48
L2-7-A	CDRL2	1CLZ	L	KVSNRFS	house mouse	IGKV1-117
L2-7-A	CDRL2	4HS8	L	SASNHAS	house mouse	IGKV3
L2-7-A	CDRL2	3GKW	L	KVSNRFS	house mouse	IGKV
L2-7-A	CDRL2	3CFJ	E	GTNKRAP	house mouse	IGLV1
L2-7-A	CDRL2	1JPT	L	YATSLAE	human	IGKV1-39
L2-7-A	CDRL2	3LRS	F	DVSHRPS	human	IGLV2-14
L2-7-A	CDRL2	1NLB	L	WASTRES	house mouse	IGKV8-21
L2-7-A	CDRL2	1IVL	B	YASQSIS	house mouse	IGKV5-43
L2-7-A	CDRL2	2J4W	L	ATSNLAS	house mouse	IGKV4-72
L2-7-A	CDRL2	2VDK	L	YGTNLVD	house mouse	IGKV14-100
L2-7-A	CDRL2	1GC1	L	GASTRAT	human	IGKV3-15
L2-7-A	CDRL2	3MOA	L	DASSLES	human	IGKV1-13
L2-7-A	CDRL2	3MO1	A	FASIRES	house mouse	IGKV8-24
L2-7-A	CDRL2	1BFO	G	NTNNLQT	Norway rat	IGKV22S7
L2-7-A	CDRL2	3LVE	A	WASTRES	human	IGKV4-1
L2-7-A	CDRL2	2R1W	A	WASTRES	house mouse	IGKV8-21
L2-7-A	CDRL2	1B6D	A	AASSLET	human	IGKV1-33
L2-7-A	CDRL2	1W72	L	DDSDRPS	human	IGLV3-21
L2-7-A	CDRL2	2OJZ	M	KVSNRFS	house mouse	IGKV1-117
L2-7-A	CDRL2	1P2C	D	YTSQSMS	house mouse	IGKV5-43
L2-7-A	CDRL2	1AD9	A	RMSNLAS	house mouse	IGKV
L2-7-A	CDRL2	3L1O	L	SGSTLQS	house mouse	IGKV16-104
L2-7-A	CDRL2	1BRE	B	DASTLET	human	IGKV1-33
L2-7-A	CDRL2	3QCV	M	KVSNLFS	house mouse	IGKV
L2-7-A	CDRL2	4HQQ	L	EDNQRPS	human	IGLV6-57
L2-7-A	CDRL2	2FX8	M	GASSRPS	human	IGKV3-20
L2-7-A	CDRL2	43CA	E	FASTRES	house mouse	IGKV8-24
L2-7-A	CDRL2	1A7R	L	YTTTLAD	house mouse	IGKV12-41
L2-7-A	CDRL2	3IDM	A	DASSLES	human	IGKV1-13
L2-7-A	CDRL2	3PHQ	A	LASNLES	house mouse	IGKV3-12
L2-7-A	CDRL2	4KVC	L	GASNRYT	house mouse	IGKV6-20
L2-7-A	CDRL2	1B6D	B	AASSLET	human	IGKV1-33
L2-7-A	CDRL2	3O2V	L	KVSNRFS	house mouse	IGKV1-110
L2-7-A	CDRL2	3KYM	G	DASNRAT	human	IGKV3-15
L2-7-A	CDRL2	1UZ8	L	QMSNLAS	house mouse	IGKV2-109
L2-7-A	CDRL2	1ZEA	L	KVSNRFS	house mouse	IGKV1-117
L2-7-A	CDRL2	1EK3	A	WASSRES	human	IGKV4-1
L2-7-A	CDRL2	1NDG	A	YASQSIS	house mouse	IGKV5-43
L2-7-A	CDRL2	3MLU	L	QDFKRPS	human	IGLV3-1
L2-7-A	CDRL2	2BJM	L	GTNNRAP	house mouse	IGLV1
L2-7-A	CDRL2	3U9P	L	YTSTLES	Norway rat	IGKV19S2
L2-7-A	CDRL2	3B9K	L	YTSTLVS	Norway rat	IGKV19S2
L2-7-A	CDRL2	1R3I	L	YASESIS	house mouse	IGKV5-48
L2-7-A	CDRL2	1V7M	L	STSNLAS	house mouse	IGKV4-57

L2-7-A	CDRL2	4K3G	B	ETDKKYS	human	IGLV7-46
L2-7-A	CDRL2	4ENE	F	DTSKLTS	house mouse	IGKV4-59
L2-7-A	CDRL2	3T4Y	A	WASTRES	house mouse	IGKV8-21
L2-7-A	CDRL2	1NGP	L	GTNNRAP	house mouse	IGLV1
L2-7-A	CDRL2	1MF2	L	AASNQGS	house mouse	IGKV3-2
L2-7-A	CDRL2	2YBR	E	DASNRAT	human	IGKV3D-11
L2-7-A	CDRL2	3O6K	L	LVSKLDS	house mouse	IGKV1-135
L2-7-A	CDRL2	2FX8	O	GASSRPS	human	IGKV3-20
L2-7-A	CDRL2	3LHP	L	GASSRPS	human	IGKV3-20
L2-7-A	CDRL2	2XZA	L	DNNKRPS	human	IGLV1-51
L2-7-A	CDRL2	1DBB	L	KVSNRFY	house mouse	IGKV1-110
L2-7-A	CDRL2	3LH2	N	GASSRPS	human	IGKV3-20
L2-7-A	CDRL2	4K3E	L	GDTSRAS	sheep	IGLV1S8
L2-7-A	CDRL2	2G2R	A	LVSKLDS	house mouse	IGKV1-133
L2-7-A	CDRL2	1DEE	A	AASSLQS	human	IGKV1-39
L2-7-A	CDRL2	1S5I	L	LVSKLDS	house mouse	IGKV1-133
L2-7-A	CDRL2	1YMH	A	WASTRES	house mouse	IGKV8-21
L2-7-A	CDRL2	2EH7	L	LVSKLDS	house mouse	IGKV1-133
L2-7-A	CDRL2	3MBX	L	WASTRES	house mouse	IGKV8-30
L2-7-A	CDRL2	4FQJ	L	RSYQRPS	human	IGLV1-47
L2-7-A	CDRL2	3G5X	C	HGTNLDD	house mouse	IGKV14-100
L2-7-A	CDRL2	4GW5	A	YASESIS	house mouse	IGKV5-48
L2-7-A	CDRL2	3N9G	L	GNNQRPS	human	IGLV1-44
L2-7-A	CDRL2	1WTL	A	GASILET	human	IGKV1-33
L2-7-A	CDRL2	1TZH	L	AASYLYS	house mouse	IGKV12-44
L2-7-A	CDRL2	2O5Z	L	KVSNRFS	house mouse	IGKV1-110
L2-7-A	CDRL2	1FVE	C	SASFLES	human	IGKV1-39
L2-7-A	CDRL2	3U79	H	DASNLET	human	IGKV1-33
L2-7-A	CDRL2	1C5C	L	DATKLDS	house mouse	IGKV9-124
L2-7-A	CDRL2	3UJT	M	FASTRES	house mouse	IGKV8-24
L2-7-A	CDRL2	2AI0	N	KVSNRFS	house mouse	IGKV1-117
L2-7-A	CDRL2	2BX5	E	AASSLQS	human	IGKV1-39
L2-7-A	CDRL2	1UM6	L	AGSTLDS	house mouse	IGKV9-124
L2-7-A	CDRL2	1XF2	A	NAKTLAE	house mouse	IGKV12-44
L2-7-A	CDRL2	1DFB	L	KASSLES	human	IGKV1-5
L2-7-A	CDRL2	3UJJ	L	EDSKRPS	human	IGLV3-10
L2-7-A	CDRL2	2A77	L	KVSNRFS	house mouse	IGKV1-117
L2-7-A	CDRL2	1RUL	L	LGTKLDS	house mouse	IGKV1-133
L2-7-A	CDRL2	4FAB	L	KVSNRFS	house mouse	IGKV1-110
L2-7-A	CDRL2	3CDF	E	DASNLET	human	IGKV1-33
L2-7-A	CDRL2	3DUR	C	WASTRES	house mouse	IGKV8-21
L2-7-A	CDRL2	3DVG	A	SARSLYS	human	IGKV1-13
L2-7-A	CDRL2	2W65	D	LVSKLDS	house mouse	IGKV1-135
L2-7-A	CDRL2	1NDM	A	YASQSIS	house mouse	IGKV5-43
L2-7-A	CDRL2	2A1W	L	KVSNRFS	house mouse	IGKV1-117
L2-7-A	CDRL2	1YQV	L	DTSKLAS	house mouse	IGKV4-59
L2-7-A	CDRL2	3W9D	D	GASNRAT	human	IGKV3
L2-7-A	CDRL2	3BKJ	L	QVSTRFS	house mouse	IGKV1-117
L2-7-A	CDRL2	3HZY	A	WASTRES	house mouse	IGKV8-21
L2-7-A	CDRL2	4JN1	L	LVSKLDS	house mouse	IGKV1-133

L2-7-A	CDRL2	3HNV	L	YTSRLHS	house mouse	IGKV10-96
L2-7-A	CDRL2	1OTS	F	DTSKLTS	house mouse	IGKV4-59
L2-7-A	CDRL2	2EKS	A	YASQSIS	human	IGKV3-15
L2-7-A	CDRL2	4DGV	L	DASNRAT	human	IGKV3-11
L2-7-A	CDRL2	1P7K	L	NAKTLAE	house mouse	IGKV12-44
L2-7-A	CDRL2	3LRH	C	DDDLLAP	human	IGLV1-36
L2-7-A	CDRL2	1LO0	L	KVSNRFS	house mouse	IGKV1-117
L2-7-A	CDRL2	3SKJ	M	KASNLHT	human	IGKV1-5
L2-7-A	CDRL2	3Q1S	L	DASNRAT	human	IGKV3D-11
L2-7-A	CDRL2	4ENE	D	DTSKLTS	house mouse	IGKV4-59
L2-7-B	CDRL2	4JFX	L	EGNTLRP	human	IGKV3-11
L2-7-B	CDRL2	1NC2	C	GNNNRPP	house mouse	IGLV1
L2-7-B	CDRL2	2ADI	A	EGNTLRP	house mouse	IGKV17-121
L2-7-B	CDRL2	2ADG	A	EGNTLRP	house mouse	IGKV17-121
L2-7-B	CDRL2	1NC2	A	GNNNRPP	house mouse	IGLV1
L2-7-B	CDRL2	4D9R	L	GGNTLRP	human	IGKV1-27
L2-7-B	CDRL2	4JG1	L	EGNTLRP	human	IGKV3-11
L2-7-B	CDRL2	4G6F	D	GKNNRPS	human	IGLV3-19
L2-7-B	CDRL2	4JG0	L	EGNTLRP	human	IGKV3-11
L2-7-B	CDRL2	3MA9	L	GDNNRPS	human	IGLV3-1
L2-7-B	CDRL2	1NC4	C	GNNNRPP	house mouse	IGLV1
L2-7-B	CDRL2	4JFZ	L	EGNTLRP	human	IGKV3-11
L2-7-B	CDRL2	4L5F	L	EGNTLRP	house mouse	IGKV17-127
L2-7-B	CDRL2	1NC4	A	GNNNRPP	house mouse	IGLV1
L2-7-B	CDRL2	3I9G	L	EGNILRP	house mouse	IGKV17-121
L2-7-B	CDRL2	4D9R	D	GGNTLRP	human	IGKV1-27
L2-7-B	CDRL2	1I8K	A	EGNTLRP	house mouse	IGKV17-127
L2-7-B	CDRL2	4JFX	A	EGNTLRP	human	IGKV3-11
L2-7-B	CDRL2	4D9Q	L	GGNTLRP	human	IGKV1-27
L2-7-B	CDRL2	1I8I	A	EGNTLRP	house mouse	IGKV17-127
L2-7-B	CDRL2	4D9Q	D	GGNTLRP	human	IGKV1-27
L2-7-C	CDRL2	2V7H	A	YTSNLHS	house mouse	IGKV10-96
L2-7-C	CDRL2	2V7H	L	YTSNLHS	house mouse	IGKV10-96
L2-7-C	CDRL2	2A6J	A	YTSRLHS	house mouse	IGKV10-96
L2-7-C	CDRL2	2A6I	A	YTSRLHS	house mouse	IGKV10-96
L2-7-C	CDRL2	2A6J	L	YTSRLHS	house mouse	IGKV10-96
L2-7-C	CDRL2	6FAB	L	FTSRSQS	house mouse	IGKV10-96
L2-11-A	CDRL2	4JJ5	A	LKKDGSHSTGD	house mouse	IGLV3
L2-11-A	CDRL2	4DCQ	A	LKKDGSHSTGD	house mouse	IGLV3
L2-11-A	CDRL2	3FFD	B	LKQDGSHSTGD	house mouse	IGLV3
L2-11-A	CDRL2	2QHR	L	LKKDGSHSTGD	house mouse	IGLV3
L2-11-A	CDRL2	2OTU	C	LKKDGSHSTGD	house mouse	IGLV3
L2-11-A	CDRL2	3S96	D	LKKDGSHSTGD	house mouse	IGLV3
L2-11-A	CDRL2	2OTW	A	LKKDGSHSTGD	house mouse	IGLV3
L2-11-A	CDRL2	2OTU	A	LKKDGSHSTGD	house mouse	IGLV3
L2-11-A	CDRL2	2OTU	G	LKKDGSHSTGD	house mouse	IGLV3
L2-11-A	CDRL2	4ISV	A	LKKDGSHSTGD	house mouse	IGLV3
L2-11-A	CDRL2	2OTU	E	LKKDGSHSTGD	house mouse	IGLV3
L2-11-A	CDRL2	2GSG	A	LKKDGSHSTGD	house mouse	IGLV3
L2-11-A	CDRL2	3S96	B	LKKDGSHSTGD	house mouse	IGLV3

L2-11-B	CDRL2	2H3N	A	YFSQSDKSQGP	human	VPREB1
L2-11-B	CDRL2	4KTD	L	YFSDSSQHQGS	rhesus monkey	IGLV
L2-11-B	CDRL2	2H3N	C	YFSQSDKSQGP	human	VPREB1
L2-11-B	CDRL2	4KTE	L	YYSDSSKYQGS	rhesus monkey	IGLV
L2-11-B	CDRL2	3BJ9	1	YFSQSDKSQGP	human	VPREB1
Unclustered	CDRL2	3MCG	2	EVNKRPS	human	IGLV2-8
Unclustered	CDRL2	1BGX	L	DSTNLAS	house mouse	IGKV4-55
Unclustered	CDRL2	1MCK	A	EVNKRPS	human	IGLV2-8
Unclustered	CDRL2	1NJ9	A	VNNARPP	house mouse	IGLV1
Unclustered	CDRL2	4LKX	B	KVSNRFS	house mouse	IGKV
Unclustered	CDRL2	1MCI	A	EVNKRPS	human	IGLV2-8
Unclustered	CDRL2	1NJ9	L	VNNARPP	house mouse	IGLV1
Unclustered	CDRL2	3KYM	O	DASNRAT	human	IGKV3-15
Unclustered	CDRL2	3EOT	L	LTSNLAS	house mouse	IGKV4-68
L3-5-A	CDRL3	4JPI	L	QQYEF	human	IGKV3-11
L3-5-A	CDRL3	3U7W	L	QQYEF	human	IGKV3-11
L3-5-A	CDRL3	3RPI	L	QVYEF	human	IGKV1-33
L3-5-A	CDRL3	4LSP	L	QQYET	human	IGKV1-NL1
L3-5-A	CDRL3	4HWE	L	QQYET	human	IGKV3-20
L3-5-A	CDRL3	4LSU	L	NAFEF	human	IGLV2-14
L3-5-A	CDRL3	4LSS	L	QQYEF	human	IGKV3-20
L3-5-A	CDRL3	4JDV	L	QQYEF	human	IGKV3-11
L3-5-A	CDRL3	3U7Y	L	QQYEF	human	IGKV3-11
L3-5-A	CDRL3	3SE9	L	QQLEF	human	IGKV3-20
L3-5-A	CDRL3	3SE8	L	QQFEF	human	IGKV3-11
L3-5-A	CDRL3	4JPK	L	QQYEF	human	IGKV3-11
L3-5-A	CDRL3	4HWB	L	QQYET	human	IGKV3-20
L3-5-A	CDRL3	4JDV	B	QQYEF	human	IGKV3-11
L3-5-A	CDRL3	4JPI	B	QQYEF	human	IGKV3-11
L3-5-A	CDRL3	3RPI	B	QVYEF	human	IGKV1-33
L3-5-A	CDRL3	4J6R	L	QQYET	human	IGKV3D-7
L3-7-A	CDRL3	1DFB	L	QQYNSYS	human	IGKV1-5
L3-7-A	CDRL3	1MIM	L	HQRSSYT	human	IGKV1-5
L3-8-A	CDRL3	2G5B	G	KQSYNLRT	house mouse	IGKV8-21
L3-8-A	CDRL3	1EAP	A	LQYYNLRT	house mouse	IGKV19-93
L3-8-A	CDRL3	3HZY	A	KQSYNLRT	house mouse	IGKV8-21
L3-8-A	CDRL3	2R1W	A	KQSYNLRT	house mouse	IGKV8-21
L3-8-A	CDRL3	4DGV	L	QQRSNWIT	human	IGKV3-11
L3-8-A	CDRL3	1PZ5	A	SQTTHVPT	house mouse	IGKV1-110
L3-8-A	CDRL3	4M61	A	HQHLSSWT	house mouse	IGKV8-27
L3-8-A	CDRL3	2R1Y	A	KQSYNLRT	house mouse	IGKV8-21
L3-8-A	CDRL3	1Q9O	C	KQSYNLRT	house mouse	IGKV8-21
L3-8-A	CDRL3	2R1X	A	KQSYNLRT	house mouse	IGKV8-21
L3-8-A	CDRL3	1IL1	B	QQYYHYRT	house mouse	IGKV8-30
L3-8-A	CDRL3	4JR9	L	LQYNSLLT	house mouse	IGKV19-93
L3-8-A	CDRL3	3V52	L	SQSTHVPT	house mouse	IGKV1-110
L3-8-A	CDRL3	1Q9W	A	KQSYNLRT	house mouse	IGKV8-21
L3-8-A	CDRL3	2XTJ	B	QQFDGDPT	human	IGKV1-6
L3-8-A	CDRL3	3IF1	C	SQSTHVPT	house mouse	IGKV1-110
L3-8-A	CDRL3	3OKM	A	KQSYNLRT	house mouse	IGKV8-21

L3-8-A	CDRL3	4JRE	C	LQYNSLLT	house mouse	IGKV19-93
L3-8-A	CDRL3	2R2E	A	KQSYNLRT	house mouse	IGKV8-21
L3-8-A	CDRL3	1QKZ	L	SQSTHFPT	house mouse	IGKV1-110
L3-8-A	CDRL3	3DUS	A	KQSYNLRT	house mouse	IGKV8-21
L3-8-A	CDRL3	2R2H	A	KQSYNLRT	house mouse	IGKV8-21
L3-8-A	CDRL3	3QEH	F	MQAKESPT	house mouse	IGKV
L3-8-A	CDRL3	4J8R	A	QQYSSYPT	house mouse	IGKV6-23
L3-8-A	CDRL3	3QEH	B	MQAKESPT	house mouse	IGKV
L3-8-A	CDRL3	1Q9L	A	KQSYNLRT	house mouse	IGKV8-21
L3-8-A	CDRL3	3HI6	L	QQSYSTPS	human	IGKV1-39
L3-8-A	CDRL3	3DUS	C	KQSYNLRT	house mouse	IGKV8-21
L3-8-A	CDRL3	3OKE	A	KQSYNLRT	house mouse	IGKV8-21
L3-8-A	CDRL3	4DGY	L	QQRSNWIT	human	IGKV3-11
L3-8-A	CDRL3	1C5D	L	LQYGNLYT	Norway rat	IGKV19S2
L3-8-A	CDRL3	3OKD	A	KQSYNLRT	house mouse	IGKV8-21
L3-8-A	CDRL3	3U6R	L	QQRSDWVT	human	IGKV3-11
L3-8-A	CDRL3	3OKO	A	KQSYNLRT	house mouse	IGKV8-21
L3-8-A	CDRL3	3IJS	A	KQSNNLRT	house mouse	IGKV8-21
L3-8-A	CDRL3	3IKC	A	KQSNNLRT	house mouse	IGKV8-21
L3-8-A	CDRL3	3HZM	A	KQSYNLRT	house mouse	IGKV8-21
L3-8-A	CDRL3	1CK0	L	KQSYNLYT	house mouse	IGKV8-21
L3-8-A	CDRL3	1M7I	A	SQTTHVPT	house mouse	IGKV1-110
L3-8-A	CDRL3	3T65	A	KQSYNLRT	house mouse	IGKV8-21
L3-8-A	CDRL3	3HZV	A	KQSYNLRT	house mouse	IGKV8-21
L3-8-A	CDRL3	3DUU	C	KQSYNLRT	house mouse	IGKV8-21
L3-8-A	CDRL3	3DUR	C	KQSYNLRT	house mouse	IGKV8-21
L3-8-A	CDRL3	3HI6	Y	QQSYSTPS	human	IGKV1-39
L3-8-A	CDRL3	3T4Y	A	KQSYNLRT	house mouse	IGKV8-21
L3-8-A	CDRL3	3DUR	A	KQSYNLRT	house mouse	IGKV8-21
L3-8-A	CDRL3	1Q9O	A	KQSYNLRT	house mouse	IGKV8-21
L3-8-A	CDRL3	3B9K	C	LQYDTLYT	Norway rat	IGKV19S2
L3-8-A	CDRL3	3QEH	D	MQAKESPT	house mouse	IGKV
L3-8-A	CDRL3	4M61	C	HQHLSSWT	house mouse	IGKV8-27
L3-8-A	CDRL3	2CK0	L	KQSYNLYT	house mouse	IGKV8-21
L3-8-A	CDRL3	3IF1	A	SQSTHVPT	house mouse	IGKV1-110
L3-8-A	CDRL3	3IJH	C	KQSNNLRT	house mouse	IGKV8-21
L3-8-A	CDRL3	1JRH	L	QQYWSTWT	house mouse	IGKV13-84
L3-8-A	CDRL3	3T77	A	KQSYNLRT	house mouse	IGKV8-21
L3-8-A	CDRL3	1Q9W	C	KQSYNLRT	house mouse	IGKV8-21
L3-8-A	CDRL3	3UO1	L	SQSTHVPT	house mouse	IGKV1-110
L3-8-A	CDRL3	1M71	A	SQTTHVPT	house mouse	IGKV1-110
L3-8-A	CDRL3	4J8R	C	QQYSSYPT	house mouse	IGKV6-23
L3-8-A	CDRL3	4J1U	C	KQSYDLPT	house mouse	IGKV8-21
L3-8-A	CDRL3	1Q9L	C	KQSYNLRT	house mouse	IGKV8-21
L3-8-A	CDRL3	1RZ7	L	QQANSFFT	human	IGKV1-12
L3-8-A	CDRL3	1T4K	C	KQSYDLPT	house mouse	IGKV8-21
L3-8-A	CDRL3	3I02	C	KQSYNLRT	house mouse	IGKV8-21
L3-8-A	CDRL3	1M7D	A	SQTTHVPT	house mouse	IGKV1-110
L3-8-A	CDRL3	3IET	C	SQSTHVPT	house mouse	IGKV1-110
L3-8-A	CDRL3	2G5B	A	KQSYNLRT	house mouse	IGKV8-21

L3-8-A	CDRL3	1C5D	A	LQYGNLYT	Norway rat	IGKV19S2
L3-8-A	CDRL3	3OKK	A	KQSYNLRT	house mouse	IGKV8-21
L3-8-A	CDRL3	3QPX	L	QQYNSRDT	Norway rat	IGKV22S9
L3-8-A	CDRL3	3CMO	L	QQYSKLFT	house mouse	IGKV10-94
L3-8-A	CDRL3	3HI5	L	QQSYSTPS	human	IGKV1-39
L3-8-A	CDRL3	1H3P	L	KQSYSLYT	house mouse	IGKV8-21
L3-8-A	CDRL3	1Q9K	A	KQSYNLRT	house mouse	IGKV8-21
L3-8-A	CDRL3	3BPC	A	KQSYNLRT	house mouse	IGKV8-21
L3-8-A	CDRL3	3CMO	X	QQYSKLFT	house mouse	IGKV10-94
L3-8-A	CDRL3	3DV4	A	KQSYNLRT	house mouse	IGKV8-21
L3-8-A	CDRL3	3IKC	C	KQSNNLRT	house mouse	IGKV8-21
L3-8-A	CDRL3	3OKN	A	KQSYNLRT	house mouse	IGKV8-21
L3-8-A	CDRL3	3DUU	A	KQSYNLRT	house mouse	IGKV8-21
L3-8-A	CDRL3	4JRE	L	LQYNSLLT	house mouse	IGKV19-93
L3-8-A	CDRL3	3QEH	H	MQAKESPT	house mouse	IGKV
L3-8-A	CDRL3	2R2B	A	KQSYNLRT	house mouse	IGKV8-21
L3-8-A	CDRL3	3I02	A	KQSYNLRT	house mouse	IGKV8-21
L3-8-A	CDRL3	1DQL	L	LQQNSNWT	human	IGKV1-6
L3-8-A	CDRL3	2G5B	C	KQSYNLRT	house mouse	IGKV8-21
L3-8-A	CDRL3	3IJS	C	KQSNNLRT	house mouse	IGKV8-21
L3-8-A	CDRL3	3V4U	L	SQSTHVPT	house mouse	IGKV1-110
L3-8-A	CDRL3	3DV6	A	KQSYNLRT	house mouse	IGKV8-21
L3-8-A	CDRL3	1A7O	L	QHFWSTPT	house mouse	IGKV12-41
L3-8-A	CDRL3	4HGW	A	KQSYNLRT	house mouse	IGKV8-21
L3-8-A	CDRL3	3IJH	A	KQSNNLRT	house mouse	IGKV8-21
L3-8-A	CDRL3	2G5B	E	KQSYNLRT	house mouse	IGKV8-21
L3-8-A	CDRL3	3SY0	A	KQSYNLRT	house mouse	IGKV8-21
L3-8-A	CDRL3	3B9K	L	LQYDTLYT	Norway rat	IGKV19S2
L3-8-A	CDRL3	2ADF	L	LQYDNLRT	house mouse	IGKV19-93
L3-8-A	CDRL3	2R23	A	KQSYNLRT	house mouse	IGKV8-21
L3-8-A	CDRL3	3HZK	A	KQSYNLRT	house mouse	IGKV8-21
L3-8-A	CDRL3	3U6R	B	QQRSDWVT	human	IGKV3-11
L3-8-A	CDRL3	3IET	A	SQSTHVPT	house mouse	IGKV1-110
L3-8-A	CDRL3	3UYR	L	SQSTHVPT	house mouse	IGKV1-110
L3-8-A	CDRL3	4FZ8	L	MQALQAVG	human	IGKV2-28
L3-8-A	CDRL3	3O2D	L	QQYYSYRT	human	IGKV4-1
L3-8-A	CDRL3	1A0Q	L	LQYYNLRT	house mouse	IGKV19-93
L3-8-A	CDRL3	3OKL	A	KQSYNLRT	house mouse	IGKV8-21
L3-8-A	CDRL3	1T4K	A	KQSYDLPT	house mouse	IGKV8-21
L3-8-B	CDRL3	3W9D	D	QQYGSSPT	human	IGKV3
L3-8-B	CDRL3	1YQV	L	QQWGRNPT	house mouse	IGKV4-59
L3-8-B	CDRL3	1KEG	L	FQGSLVPT	house mouse	IGKV1-117
L3-8-B	CDRL3	3W9D	B	QQYGSSPT	house mouse	IGKV1-117
L3-8-B	CDRL3	3VW3	L	FRGSHVPT	house mouse	IGKV1-117
L3-8-B	CDRL3	2IFF	L	QQWGRNPT	house mouse	IGKV4-59
L3-8-B	CDRL3	3W9E	B	QQYGSSPT	house mouse	IGKV1-117
L3-8-B	CDRL3	1BQL	L	QQWGRNPT	house mouse	IGKV4-59
L3-8-B	CDRL3	1EHL	L	FQGSLVPT	house mouse	IGKV1-117
L3-8-C	CDRL3	2FD6	L	QQWNYPFT	house mouse	IGKV4-86
L3-8-C	CDRL3	3PHO	A	QHSRELRT	house mouse	IGKV3-12

L3-8-C	CDRL3	2FAT	L	QQWNYPFT	house mouse	IGKV4-86
L3-8-C	CDRL3	3PHQ	A	QHSRELRT	house mouse	IGKV3-12
L3-8-C	CDRL3	1E6O	L	QQWNYPFT	house mouse	IGKV4-86
L3-8-C	CDRL3	3BT2	L	QQWNYPFT	house mouse	IGKV4-86
L3-8-D	CDRL3	1TZH	L	QSSASPAT	house mouse	IGKV12-44
L3-8-D	CDRL3	1TZH	A	QSSASPAT	house mouse	IGKV12-44
L3-8-D	CDRL3	3L5Y	L	QQHDYPYT	house mouse	IGKV12-44
L3-9,10-A	CDRL3	2G60	L	FQGAHAPYT	house mouse	IGKV1-117
L3-9,10-A	CDRL3	4M43	L	QQSNSWPVT	house mouse	IGKV5-48
L3-9,10-A	CDRL3	2W60	B	WQGTHFPLT	house mouse	IGKV1-135
L3-9,10-A	CDRL3	3VG9	B	QHFYGSTWA	house mouse	IGKV12-46
L3-9,10-A	CDRL3	1TPX	C	WQGSHFPQT	house mouse	IGKV1-135
L3-9,10-A	CDRL3	4GW1	A	QQNNNWPTT	house mouse	IGKV5-48
L3-9,10-A	CDRL3	3QEG	L	QQYGSSPET	human	IGKV3-20
L3-9,10-A	CDRL3	2HRP	M	QQSKEVPWT	house mouse	IGKV3-2
L3-9,10-A	CDRL3	1RUQ	L	AQNLELPYT	house mouse	IGKV2-109
L3-9,10-A	CDRL3	3MBX	L	QQYYSYPFT	house mouse	IGKV8-30
L3-9,10-A	CDRL3	1MRC	L	SQSTHVPRT	house mouse	IGKV1-110
L3-9,10-A	CDRL3	3NCJ	L	QQGNTLPYT	human	IGKV1-39
L3-9,10-A	CDRL3	3SKJ	M	QQYNSYSRT	human	IGKV1-5
L3-9,10-A	CDRL3	3EOA	A	QQHNEYPLT	human	IGKV1-16
L3-9,10-A	CDRL3	1AXS	L	LQYDEFPYT	house mouse	IGKV14-111
L3-9,10-A	CDRL3	2AJ3	E	QHLKRYPYT	human	IGKV1-9
L3-9,10-A	CDRL3	2AJ3	A	QHLKRYPYT	human	IGKV1-9
L3-9,10-A	CDRL3	3I2C	L	QHSGELPFT	house mouse	IGKV3-12
L3-9,10-A	CDRL3	3OR6	B	QQSNRWPFT	house mouse	IGKV5-48
L3-9,10-A	CDRL3	1LO2	L	FQGSHFPLA	house mouse	IGKV1-117
L3-9,10-A	CDRL3	3CFC	L	AQNLELPPT	house mouse	IGKV2-109
L3-9,10-A	CDRL3	3LH2	M	QQYGQSLST	human	IGKV3-20
L3-9,10-A	CDRL3	2V7N	G	HQYSDISPT	human	IGKV3-11
L3-9,10-A	CDRL3	1UB5	B	AQNLELPPT	house mouse	IGKV2-109
L3-9,10-A	CDRL3	3L7F	A	QQHNEYPYT	human	IGKV3-11
L3-9,10-A	CDRL3	3SGD	L	WQGSHFPYT	house mouse	IGKV1-135
L3-9,10-A	CDRL3	1A7Q	L	QHFWSTPRS	house mouse	IGKV12-41
L3-9,10-A	CDRL3	2Z91	D	QQWSSNPPT	house mouse	IGKV4-59
L3-9,10-A	CDRL3	1FL5	L	FQGSHVPRT	house mouse	IGKV1-117
L3-9,10-A	CDRL3	2B2X	M	QQWSGNPWT	house mouse	IGKV4-68
L3-9,10-A	CDRL3	3RVX	C	LQYDEFPYT	house mouse	IGKV14-111
L3-9,10-A	CDRL3	1NDG	A	QQSNSWPYT	house mouse	IGKV5-43
L3-9,10-A	CDRL3	1XF3	L	QHHYGTPLT	house mouse	IGKV12-44
L3-9,10-A	CDRL3	2H9G	A	QQSYTTPPT	human	IGKV1-39
L3-9,10-A	CDRL3	1HIL	A	QNDYSNPLT	house mouse	IGKV8-19
L3-9,10-A	CDRL3	4G6K	L	LQGKMLPWT	human	IGKV1-39
L3-9,10-A	CDRL3	3EO0	C	QQYADSPIT	human	IGKV3-20
L3-9,10-A	CDRL3	1PSK	L	QQRSGYPFT	house mouse	IGKV4-57
L3-9,10-A	CDRL3	1XF2	L	QHHYGTPLT	house mouse	IGKV12-44
L3-9,10-A	CDRL3	3TCL	L	QQYGRSPYT	human	IGKV3-20
L3-9,10-A	CDRL3	1I3G	L	QQYSSYPLT	house mouse	IGKV6-23
L3-9,10-A	CDRL3	1GAF	L	LQYASYPRT	house mouse	IGKV9-124
L3-9,10-A	CDRL3	1P7K	L	QHHYGTPLT	house mouse	IGKV12-44

L3-9,10-A	CDRL3	1A6T	C	QQYSSHPLT	house mouse	IGKV4-72
L3-9,10-A	CDRL3	3H3P	L	QQYGQSLST	human	IGKV3-20
L3-9,10-A	CDRL3	3IFN	L	FQSSHVPLT	house mouse	IGKV1-117
L3-9,10-A	CDRL3	4MA7	L	QQSNTWPYT	house mouse	IGKV5-48
L3-9,10-A	CDRL3	1R3K	A	QQSNRWPFT	house mouse	IGKV5-48
L3-9,10-A	CDRL3	1RIV	L	MQHLEYPFT	house mouse	IGKV2-137
L3-9,10-A	CDRL3	3V6O	E	LQHWNYPLT	house mouse	IGKV6-14
L3-9,10-A	CDRL3	1HEZ	A	QQSYSTPRT	human	IGKV1-39
L3-9,10-A	CDRL3	1XIW	G	QQGNTLPWT	house mouse	IGKV10-96
L3-9,10-A	CDRL3	1LO0	L	FQGSHFPLA	house mouse	IGKV1-117
L3-9,10-A	CDRL3	1JHK	L	HHFWSTPWT	house mouse	IGKV12-41
L3-9,10-A	CDRL3	2R0L	L	QQSYTTPPT	human	IGKV1-39
L3-9,10-A	CDRL3	4NZU	L	QHYDDFPIS	human	IGKV1-33
L3-9,10-A	CDRL3	1OPG	L	QQSNSWPLT	house mouse	IGKV5-43
L3-9,10-A	CDRL3	3RVU	C	LQYDEFPYT	house mouse	IGKV14-111
L3-9,10-A	CDRL3	2BFV	L	SQSSHVPLT	house mouse	IGKV1-110
L3-9,10-A	CDRL3	1U8H	A	QQLHFYPHT	human	IGKV1D-13
L3-9,10-A	CDRL3	3CDY	A	QQYDNLPYT	human	IGKV1-33
L3-9,10-A	CDRL3	1VGE	L	QQFNSYPLT	human	IGKV1-13
L3-9,10-A	CDRL3	3FCT	A	QQYSSYPLT	house mouse	IGKV6-13
L3-9,10-A	CDRL3	3LH2	N	QQYGQSLST	human	IGKV3-20
L3-9,10-A	CDRL3	1TQB	C	WQGSHFPQT	house mouse	IGKV1-135
L3-9,10-A	CDRL3	3QCV	L	SQSTHFPFT	house mouse	IGKV
L3-9,10-A	CDRL3	1DBB	L	SQSSHVPPT	house mouse	IGKV1-110
L3-9,10-A	CDRL3	1G7H	A	QHFASTPRT	house mouse	IGKV12-41
L3-9,10-A	CDRL3	2W9D	L	HQWRSNPYT	house mouse	IGKV4-59
L3-9,10-A	CDRL3	3ZDY	L	VQYAQLPYT	house mouse	IGKV14-100
L3-9,10-A	CDRL3	1FDL	L	QHFWSTPRT	house mouse	IGKV12-41
L3-9,10-A	CDRL3	3QO1	A	MQNKQTPLT	human	IGKV2-28
L3-9,10-A	CDRL3	1KB5	L	QHHYGTPYT	house mouse	IGKV12-44
L3-9,10-A	CDRL3	3BAE	L	FQASLVPLT	house mouse	IGKV1-117
L3-9,10-A	CDRL3	2VDK	L	VQYAQLPYT	house mouse	IGKV14-100
L3-9,10-A	CDRL3	1VPO	L	FDGSTVPPK	house mouse	IGKV1-117
L3-9,10-A	CDRL3	3G5X	C	VQYAQFPWT	house mouse	IGKV14-100
L3-9,10-A	CDRL3	2W65	B	WQGTHFPLT	house mouse	IGKV1-135
L3-9,10-A	CDRL3	1ND0	A	WQGTHFPYT	house mouse	IGKV1-133
L3-9,10-A	CDRL3	1UM5	L	LQYASYPRT	house mouse	IGKV9-124
L3-9,10-A	CDRL3	3C6S	G	AHNVELPRT	house mouse	IGKV2-109
L3-9,10-A	CDRL3	3SOB	L	QQSYTTPPT	human	IGKV1-39
L3-9,10-A	CDRL3	4IJ3	B	QQHYSIPLT	house mouse	IGKV6-17
L3-9,10-A	CDRL3	1BFO	A	LQHISRPRT	Norway rat	IGKV22S7
L3-9,10-A	CDRL3	1IGJ	C	SQTTHVPPT	house mouse	IGKV1-110
L3-9,10-A	CDRL3	1T66	C	SQSTHVPWT	house mouse	IGKV1-110
L3-9,10-A	CDRL3	1IGT	A	QQGQSYPLT	house mouse	IGKV10-94
L3-9,10-A	CDRL3	1KIQ	A	QHFWSTPRT	house mouse	IGKV12-41
L3-9,10-A	CDRL3	1RUR	L	AQNLELPYT	house mouse	IGKV2-109
L3-9,10-A	CDRL3	2F5B	L	QQLHFYPHT	human	IGKV1D-13
L3-9,10-A	CDRL3	12E8	M	QQYSSYPLT	house mouse	IGKV6-13
L3-9,10-A	CDRL3	1NDM	A	QQSNSWPYT	house mouse	IGKV5-43
L3-9,10-A	CDRL3	1D5B	A	LQYDEFPYT	house mouse	IGKV14-111

L3-9,10-A	CDRL3	4MWF	L	QQYYRSPLT	human	IGKV3-15
L3-9,10-A	CDRL3	1EMT	L	QQYSRLPFT	house mouse	IGKV10-94
L3-9,10-A	CDRL3	3FO9	L	SQGTHVPYT	house mouse	IGKV1-110
L3-9,10-A	CDRL3	1AR2	A	QQYQSLPYT	human	IGKV1-33
L3-9,10-A	CDRL3	1UB5	L	AQNLELPPT	house mouse	IGKV2-109
L3-9,10-A	CDRL3	2OJZ	M	FQGSHIPLT	house mouse	IGKV1-117
L3-9,10-A	CDRL3	1DN0	A	QQYGSSPLT	human	IGKV3D-20
L3-9,10-A	CDRL3	4LEO	B	QSDYSYPYT	human	IGKV4-1
L3-9,10-A	CDRL3	1S5I	L	VQGTHFPRT	house mouse	IGKV1-133
L3-9,10-A	CDRL3	35C8	L	HQYHRSPYT	house mouse	IGKV4-74
L3-9,10-A	CDRL3	3Q3G	J	QQYYSYPLT	house mouse	IGKV8-30
L3-9,10-A	CDRL3	1MNU	L	SQSTHVPRT	house mouse	IGKV1-110
L3-9,10-A	CDRL3	4N9G	L	QQHNSYPRT	human	IGKV1-16
L3-9,10-A	CDRL3	2IMN	A	QNDHSYPLT	house mouse	IGKV4-1
L3-9,10-A	CDRL3	3IU4	L	QQHYSTPWT	house mouse	IGKV6-17
L3-9,10-A	CDRL3	2YBR	B	QQYRYSPRT	human	IGKV3D-11
L3-9,10-A	CDRL3	4EBQ	L	QQWTSYPLT	house mouse	IGKV4-55
L3-9,10-A	CDRL3	3BZ4	A	AHNVELPRT	house mouse	IGKV2-109
L3-9,10-A	CDRL3	2VQ1	E	SQTTHVPWT	house mouse	IGKV1-110
L3-9,10-A	CDRL3	2AAB	L	QQSRKIPYT	house mouse	IGKV3-1
L3-9,10-A	CDRL3	2YC1	E	QQYRYSPRT	human	IGKV3D-11
L3-9,10-A	CDRL3	2UYL	X	FQGSHVPRT	house mouse	IGKV1-117
L3-9,10-A	CDRL3	4MA8	L	QQSNTWPYT	house mouse	IGKV5-48
L3-9,10-A	CDRL3	3NID	F	VQYAQLPYT	house mouse	IGKV14-100
L3-9,10-A	CDRL3	4FAB	L	SQSTHVPWT	house mouse	IGKV1-110
L3-9,10-A	CDRL3	3EYF	C	QQRSMWPPVT	human	IGKV3-11
L3-9,10-A	CDRL3	1A7R	L	QHFWSTPRT	house mouse	IGKV12-41
L3-9,10-A	CDRL3	1FE8	M	QNGGTNPWT	house mouse	IGKV10-96
L3-9,10-A	CDRL3	2FJF	C	QQSYTTPPT	human	IGKV1-39
L3-9,10-A	CDRL3	1YEC	L	VQGTHFPYT	house mouse	IGKV1-133
L3-9,10-A	CDRL3	3U0T	C	LQGTHYPVL	house mouse	IGKV
L3-9,10-A	CDRL3	3CDC	A	QQYDNLPYT	human	IGKV1-33
L3-9,10-A	CDRL3	1FJ1	A	LQYDNLQRT	house mouse	IGKV19-93
L3-9,10-A	CDRL3	2HFF	A	QQSRITPPT	human	IGKV1-39
L3-9,10-A	CDRL3	1QP1	A	QQYDDLPYT	human	IGKV1-33
L3-9,10-A	CDRL3	1FVD	C	QQHYTTPPT	human	IGKV1-39
L3-9,10-A	CDRL3	2BX5	E	QQSYSTPNT	human	IGKV1-39
L3-9,10-A	CDRL3	1TET	L	FQGSHIPFT	house mouse	IGKV1-117
L3-9,10-A	CDRL3	4ETQ	L	QQWTSYPLT	house mouse	IGKV4-55
L3-9,10-A	CDRL3	1IVL	A	QQVSEWPFT	house mouse	IGKV5-43
L3-9,10-A	CDRL3	1WTL	A	QQYDTLPLT	human	IGKV1-33
L3-9,10-A	CDRL3	3CDC	B	QQYDNLPYT	human	IGKV1-33
L3-9,10-A	CDRL3	3GNM	L	FQGSHVPFT	house mouse	IGKV1-117
L3-9,10-A	CDRL3	1JGL	L	HHFWSTPWT	house mouse	IGKV12-41
L3-9,10-A	CDRL3	2HVK	B	QQSNRWPFT	house mouse	IGKV5-48
L3-9,10-A	CDRL3	1CT8	A	HQTHGRPLT	house mouse	IGKV5-43
L3-9,10-A	CDRL3	1RUA	L	WQGTHFPYT	house mouse	IGKV1-133
L3-9,10-A	CDRL3	2UYL	V	FQGSHVPRT	house mouse	IGKV1-117
L3-9,10-A	CDRL3	1SY6	L	QQWSSNPFT	house mouse	IGKV4-59
L3-9,10-A	CDRL3	1KIR	A	QHFWSTPRT	house mouse	IGKV12-41

L3-9,10-A	CDRL3	1RHH	C	QQYGTSPYT	human	IGKV3-20
L3-9,10-A	CDRL3	3ZKM	D	QQNNEDPWT	house mouse	IGKV3-10
L3-9,10-A	CDRL3	2FJF	G	QQSYTTPPT	human	IGKV1-39
L3-9,10-A	CDRL3	3O6L	L	WQGTHFPRT	house mouse	IGKV1-135
L3-9,10-A	CDRL3	1IBG	L	QHSREYPLT	house mouse	IGKV3-12
L3-9,10-A	CDRL3	3LDB	B	QQGISNPYT	Norway rat	IGKV8S6
L3-9,10-A	CDRL3	1T3F	A	GQSYNYPFT	human	IGKV1-5
L3-9,10-A	CDRL3	2XQY	K	QHSRELPWT	house mouse	IGKV3-12
L3-9,10-A	CDRL3	4GAY	B	QQNNVDPWT	house mouse	IGKV3-10
L3-9,10-A	CDRL3	1MHH	A	KQAYIPPLT	house mouse	IGKV8-21
L3-9,10-A	CDRL3	3QG6	L	SQTTHVPYT	house mouse	IGKV1-110
L3-9,10-A	CDRL3	2A77	L	FQGSHIPWT	house mouse	IGKV1-117
L3-9,10-A	CDRL3	2Y6S	C	QHIAELTRT	house mouse	IGKV3-12
L3-9,10-A	CDRL3	1PG7	L	LQHGESPWT	human	IGKV1-39
L3-9,10-A	CDRL3	1C5C	L	LQYASFPRT	house mouse	IGKV9-124
L3-9,10-A	CDRL3	3MO1	A	LQHYNTPWT	house mouse	IGKV8-24
L3-9,10-A	CDRL3	2AI0	M	FQGSHIPWT	house mouse	IGKV1-117
L3-9,10-A	CDRL3	2NLJ	A	QQSNRWPFT	house mouse	IGKV5-48
L3-9,10-A	CDRL3	1E4W	L	QQGGALPFT	house mouse	IGKV10-96
L3-9,10-A	CDRL3	4JFX	A	LQSFNVPLT	human	IGKV3-11
L3-9,10-A	CDRL3	1U8J	A	QQLHFYPHT	human	IGKV1D-13
L3-9,10-A	CDRL3	1RU9	L	WQGTHFPYT	house mouse	IGKV1-133
L3-9,10-A	CDRL3	3GIZ	L	QQRSNWPIT	human	IGKV3-11
L3-9,10-A	CDRL3	3LH2	L	QQYGQSLST	human	IGKV3-20
L3-9,10-A	CDRL3	1AI1	L	QQNNEDPPT	house mouse	IGKV3-10
L3-9,10-A	CDRL3	3UTZ	A	FQASHVPPT	house mouse	IGKV1-117
L3-9,10-A	CDRL3	2PCP	A	FQGTHAPYT	house mouse	IGKV1-117
L3-9,10-A	CDRL3	1QAC	A	LQYYSTPYS	human	IGKV4-1
L3-9,10-A	CDRL3	2BMK	L	QQRSTYPFT	house mouse	IGKV4-57
L3-9,10-A	CDRL3	2FR4	L	QHHYGTPLT	house mouse	IGKV12-44
L3-9,10-A	CDRL3	1YEH	L	VQGTHFPYT	house mouse	IGKV1-133
L3-9,10-A	CDRL3	1RUL	L	WQGTHFPYT	house mouse	IGKV1-133
L3-9,10-A	CDRL3	3UTZ	D	FQASHVPPT	house mouse	IGKV1-117
L3-9,10-A	CDRL3	2FJF	A	QQSYTTPPT	human	IGKV1-39
L3-9,10-A	CDRL3	4JM4	L	QQYEEWPRT	human	IGKV3-15
L3-9,10-A	CDRL3	2F19	L	QQGSTLPRT	house mouse	IGKV10-96
L3-9,10-A	CDRL3	1T66	L	SQSTHVPWT	house mouse	IGKV1-110
L3-9,10-A	CDRL3	3I75	A	LQYDDLLLT	house mouse	IGKV19-93
L3-9,10-A	CDRL3	1IGM	L	QQYQNLPLT	human	IGKV1-33
L3-9,10-A	CDRL3	2Q1E	C	QQYDNLPYT	human	IGKV1-33
L3-9,10-A	CDRL3	2F5A	L	QQLHFYPHT	human	IGKV1D-13
L3-9,10-A	CDRL3	3CDF	D	QQYDNLPYT	human	IGKV1-33
L3-9,10-A	CDRL3	3SGE	I	WQGSHFPYT	house mouse	IGKV1-135
L3-9,10-A	CDRL3	2HFF	L	QQSRITPPT	human	IGKV1-39
L3-9,10-A	CDRL3	3MNV	A	LQHYNTPWT	house mouse	IGKV8-24
L3-9,10-A	CDRL3	2VDO	L	VQYAQLPYT	house mouse	IGKV14-100
L3-9,10-A	CDRL3	2C1O	A	VQGSHFPPT	house mouse	IGKV1-133
L3-9,10-A	CDRL3	1FRG	L	QNDYSHPLT	house mouse	IGKV8-19
L3-9,10-A	CDRL3	4HCR	N	MQNIQLPWT	house mouse	IGKV
L3-9,10-A	CDRL3	3UC0	M	QQFNSYPLT	human	IGKV1-13

L3-9,10-A	CDRL3	3LD8	B	QQGISNPYT	Norway rat	IGKV8S6
L3-9,10-A	CDRL3	3AAZ	B	FQASLVPLT	house mouse	IGKV
L3-9,10-A	CDRL3	4L5F	L	LQSDNLPLT	house mouse	IGKV17-127
L3-9,10-A	CDRL3	2DDQ	L	FQGSHVPLT	house mouse	IGKV1-117
L3-9,10-A	CDRL3	1F58	L	QQSHEDPLT	house mouse	IGKV3-4
L3-9,10-A	CDRL3	1YMH	A	KQAYIPPLT	house mouse	IGKV8-21
L3-9,10-A	CDRL3	3CFD	L	QQGTTLPPT	house mouse	IGKV10-96
L3-9,10-A	CDRL3	2O5X	L	FQSTHFFPT	house mouse	IGKV1-110
L3-9,10-A	CDRL3	1L7I	L	QQYYIYPYT	human	IGKV1-16
L3-9,10-A	CDRL3	3B2U	D	HQYGSTPLT	human	IGKV3D-11
L3-9,10-A	CDRL3	1R3J	A	QQSNRWPFT	house mouse	IGKV5-48
L3-9,10-A	CDRL3	1AXT	L	SQGTHVPYT	house mouse	IGKV1-110
L3-9,10-A	CDRL3	1IGJ	A	SQTTHVPPT	house mouse	IGKV1-110
L3-9,10-A	CDRL3	1CFV	L	SQSSHVPLT	house mouse	IGKV1-110
L3-9,10-A	CDRL3	43CA	E	QQHYRAPRT	house mouse	IGKV8-24
L3-9,10-A	CDRL3	3INU	L	QQYYSAPLT	human	IGKV4-1
L3-9,10-A	CDRL3	1I8I	A	LQSFNVPLT	house mouse	IGKV17-127
L3-9,10-A	CDRL3	1EEQ	B	QQYYSHPYS	human	IGKV4-1
L3-9,10-A	CDRL3	1MLC	A	QQSNSWPRT	house mouse	IGKV5-43
L3-9,10-A	CDRL3	2AJZ	A	QQFVEYPFT	house mouse	IGKV2-112
L3-9,10-A	CDRL3	1UZ8	A	AQNLEVPWT	house mouse	IGKV2-109
L3-9,10-A	CDRL3	1U93	A	QQLHFYPHT	human	IGKV1D-13
L3-9,10-A	CDRL3	4G6J	L	HQSSSLPFT	human	IGKV1-39
L3-9,10-A	CDRL3	3LS4	L	QQGSSIPLT	house mouse	IGKV4-91
L3-9,10-A	CDRL3	2A6I	A	QQGNTLPRT	house mouse	IGKV10-96
L3-9,10-A	CDRL3	4AEI	N	FQGSHVPLT	house mouse	IGKV1-117
L3-9,10-A	CDRL3	2ATK	B	QQSNRWPFT	house mouse	IGKV5-48
L3-9,10-A	CDRL3	4I2X	C	QQHYSTPWT	human	IGKV6-17
L3-9,10-A	CDRL3	3MNW	A	LQHYNTPWT	house mouse	IGKV8-24
L3-9,10-A	CDRL3	4LLW	B	QQYYIYPYT	human	IGKV1-16
L3-9,10-A	CDRL3	2HFG	L	QQSQISPPT	human	IGKV1-5
L3-9,10-A	CDRL3	1BOG	A	LQYDDFPLT	house mouse	IGKV14-111
L3-9,10-A	CDRL3	1MRE	L	SQSTHVPRT	house mouse	IGKV1-110
L3-9,10-A	CDRL3	1B0W	C	QQYDDLPYT	human	IGKV1-33
L3-9,10-A	CDRL3	1YEG	L	VQGTHFPYT	house mouse	IGKV1-133
L3-9,10-A	CDRL3	1UB6	L	AQNLELPPT	house mouse	IGKV2-109
L3-9,10-A	CDRL3	2A1W	M	FQGSHIPWT	house mouse	IGKV1-117
L3-9,10-A	CDRL3	1FL6	L	FQGSHVPRT	house mouse	IGKV1-117
L3-9,10-A	CDRL3	1I9I	L	FQGSHVPPT	house mouse	IGKV1-117
L3-9,10-A	CDRL3	1BFV	L	SQSSHVPLT	house mouse	IGKV1-110
L3-9,10-A	CDRL3	3IDJ	A	QQLHFYPHT	human	IGKV1-13
L3-9,10-A	CDRL3	3RVW	C	LQYDEFPYT	house mouse	IGKV14-111
L3-9,10-A	CDRL3	1NMC	C	QQDFTLPFT	house mouse	IGKV10-96
L3-9,10-A	CDRL3	4AL8	L	LQHWTYPYT	human	IGKV1-17
L3-9,10-A	CDRL3	3IDN	A	QQLHFYPHT	human	IGKV1-13
L3-9,10-A	CDRL3	4DTG	L	LQATHFPQT	house mouse	IGKV
L3-9,10-A	CDRL3	3FO0	L	FQGSHLPPT	house mouse	IGKV1-117
L3-9,10-A	CDRL3	2FJG	A	QQSYTTPPT	human	IGKV1-39
L3-9,10-A	CDRL3	1QYG	L	MQHLEYPFT	house mouse	IGKV2-137
L3-9,10-A	CDRL3	4JLR	L	FQGSGYPFT	human	IGKV1-5

L3-9,10-A	CDRL3	1DQQ	C	QQSNSWPYT	house mouse	IGKV5-43
L3-9,10-A	CDRL3	1R3L	A	QQSNRWPFT	house mouse	IGKV5-48
L3-9,10-A	CDRL3	4G7V	L	QQYFYWPIT	human	IGKV1-5
L3-9,10-A	CDRL3	3CDF	E	QQYDNLPYT	human	IGKV1-33
L3-9,10-A	CDRL3	2IH3	B	QQSNRWPFT	house mouse	IGKV5-48
L3-9,10-A	CDRL3	1FL6	A	FQGSHVPRT	house mouse	IGKV1-117
L3-9,10-A	CDRL3	3EYF	A	QQRSMWPPVT	human	IGKV3-11
L3-9,10-A	CDRL3	1YEE	L	VQGTHFPYT	house mouse	IGKV1-133
L3-9,10-A	CDRL3	4MSW	B	QQSNRWPFT	house mouse	IGKV5-48
L3-9,10-A	CDRL3	4AEI	M	FQGSHVPLT	house mouse	IGKV1-117
L3-9,10-A	CDRL3	12E8	L	QQYSSYPLT	house mouse	IGKV6-13
L3-9,10-A	CDRL3	1KN2	L	VQGTHFPYT	house mouse	IGKV1-133
L3-9,10-A	CDRL3	1N8Z	A	QQHYTTPPT	human	IGKV1-39
L3-9,10-A	CDRL3	3SKJ	L	QQYNSYSRT	human	IGKV1-5
L3-9,10-A	CDRL3	3WKM	M	QQNNEDPWT	house mouse	IGKV3-10
L3-9,10-A	CDRL3	3O6K	L	WQGTHFPRT	house mouse	IGKV1-135
L3-9,10-A	CDRL3	3CFB	L	AQNLELPPT	house mouse	IGKV2-109
L3-9,10-A	CDRL3	25C8	L	HQYHRSPYT	house mouse	IGKV4-74
L3-9,10-A	CDRL3	4ENE	D	QQWSSHPQT	house mouse	IGKV4-59
L3-9,10-A	CDRL3	3CLF	L	QQYSSFPLT	house mouse	IGKV6-15
L3-9,10-A	CDRL3	1HI6	A	LQYDDFPLT	house mouse	IGKV14-111
L3-9,10-A	CDRL3	3STL	B	QQSNRWPFT	house mouse	IGKV5-48
L3-9,10-A	CDRL3	1D6V	L	LQYDEFPYT	house mouse	IGKV14-111
L3-9,10-A	CDRL3	2DQH	L	QQSNSWPYT	house mouse	IGKV5-43
L3-9,10-A	CDRL3	2HKH	L	SQSTHVPFT	house mouse	IGKV1-110
L3-9,10-A	CDRL3	1DQQ	A	QQSNSWPYT	house mouse	IGKV5-43
L3-9,10-A	CDRL3	2C1P	A	VQGSHFPPT	house mouse	IGKV1-133
L3-9,10-A	CDRL3	4GW1	C	QQNNNWPTT	house mouse	IGKV5-48
L3-9,10-A	CDRL3	1FVD	A	QQHYTTPPT	human	IGKV1-39
L3-9,10-A	CDRL3	4F37	K	FQGSRVPLT	house mouse	IGKV1-117
L3-9,10-A	CDRL3	3QOS	A	QQYYSTPLT	human	IGKV4-1
L3-9,10-A	CDRL3	1PLG	L	FQGTHVPYT	house mouse	IGKV1-110
L3-9,10-A	CDRL3	4FNL	L	QQYDGLPFT	human	IGKV1-33
L3-9,10-A	CDRL3	4LKX	B	FQGSHVPPT	house mouse	IGKV
L3-9,10-A	CDRL3	3QCU	L	SQSTHFPFT	house mouse	IGKV
L3-9,10-A	CDRL3	4K2U	L	QQGSTFPWT	house mouse	IGKV10-96
L3-9,10-A	CDRL3	1FAI	L	QQGSTLPRT	house mouse	IGKV10-96
L3-9,10-A	CDRL3	2BX5	J	QQSYSTPNT	human	IGKV1-39
L3-9,10-A	CDRL3	3L5W	L	QQHNEYPYT	house mouse	IGKV16-104
L3-9,10-A	CDRL3	4HDI	L	SQSTHVPWT	house mouse	IGKV1-110
L3-9,10-A	CDRL3	2A6J	L	QQGNTLPRT	house mouse	IGKV10-96
L3-9,10-A	CDRL3	1NLB	L	KQAYIPPLT	house mouse	IGKV8-21
L3-9,10-A	CDRL3	3GI8	L	QQHYSTPYT	house mouse	IGKV8-24
L3-9,10-A	CDRL3	3U7A	A	QQYDNLPFT	human	IGKV1-33
L3-9,10-A	CDRL3	1JPS	L	LQHGESPWT	human	IGKV1-39
L3-9,10-A	CDRL3	1VFB	A	QHFWSTPRT	house mouse	IGKV12-41
L3-9,10-A	CDRL3	4GMT	L	QQYNSYPYT	house mouse	IGKV6-15
L3-9,10-A	CDRL3	43CA	G	QQHYRAPRT	house mouse	IGKV8-24
L3-9,10-A	CDRL3	2Z92	B	QQWSSNPPT	house mouse	IGKV4-59
L3-9,10-A	CDRL3	3BZ4	E	AHNVELPRT	house mouse	IGKV2-109

L3-9,10-A	CDRL3	1KFA	M	SQSTHVPFT	house mouse	IGKV1-110
L3-9,10-A	CDRL3	1N4X	M	FQGSHFPPT	house mouse	IGKV1-117
L3-9,10-A	CDRL3	3QPQ	C	QHFWSTPFT	house mouse	IGKV12-41
L3-9,10-A	CDRL3	3F58	L	QQSHEDPLT	house mouse	IGKV3-4
L3-9,10-A	CDRL3	2ADG	A	LQSDTLPLT	house mouse	IGKV17-121
L3-9,10-A	CDRL3	3Q3G	A	QQYYSYPLT	house mouse	IGKV8-30
L3-9,10-A	CDRL3	43CA	A	QQHYRAPRT	house mouse	IGKV8-24
L3-9,10-A	CDRL3	1GPO	L	QQITDWPFT	house mouse	IGKV5-43
L3-9,10-A	CDRL3	1FJ1	C	LQYDNLQRT	house mouse	IGKV19-93
L3-9,10-A	CDRL3	1XIW	C	QQGNTLPWT	house mouse	IGKV10-96
L3-9,10-A	CDRL3	1XF2	A	QHHYGTPLT	house mouse	IGKV12-44
L3-9,10-A	CDRL3	3V0W	L	LQGQSYPRT	house mouse	IGKV11-125
L3-9,10-A	CDRL3	2AI0	L	FQGSHIPWT	house mouse	IGKV1-117
L3-9,10-A	CDRL3	1XF5	A	KQAYIPPLT	house mouse	IGKV8-21
L3-9,10-A	CDRL3	4DKF	M	QQYWSEPVT	human	IGKV1-39
L3-9,10-A	CDRL3	1RUP	L	WQGTHFPYT	house mouse	IGKV1-133
L3-9,10-A	CDRL3	3L5X	L	QQHNEYPYT	human	IGKV3-11
L3-9,10-A	CDRL3	3R06	C	QQYYNYPWT	house mouse	IGKV14-111
L3-9,10-A	CDRL3	2UZI	L	QQSVMIPMT	human	IGKV1-39
L3-9,10-A	CDRL3	3IFL	L	FQSSHVPLT	house mouse	IGKV1-117
L3-9,10-A	CDRL3	3NA9	L	QQGNTLSYT	human	IGKV1-39
L3-9,10-A	CDRL3	2BX5	F	QQSYSTPNT	human	IGKV1-39
L3-9,10-A	CDRL3	3B2U	U	HQYGSTPLT	human	IGKV3D-11
L3-9,10-A	CDRL3	1B6D	A	QQYDSLPLT	human	IGKV1-33
L3-9,10-A	CDRL3	3VG0	L	LQHWNYPLT	house mouse	IGKV6-14
L3-9,10-A	CDRL3	2IPT	L	FQGSHVPLT	house mouse	IGKV1-117
L3-9,10-A	CDRL3	1DEE	A	QQSYSAPRT	human	IGKV1-39
L3-9,10-A	CDRL3	2C1P	L	VQGSHFPPT	house mouse	IGKV1-133
L3-9,10-A	CDRL3	3ZKN	D	QQNNEDPWT	house mouse	IGKV3-10
L3-9,10-A	CDRL3	2VDP	L	VQYAQLPYT	house mouse	IGKV14-100
L3-9,10-A	CDRL3	1OAK	L	QQYEKLPWT	house mouse	IGKV10-94
L3-9,10-A	CDRL3	4JG0	L	LQSFNVPLT	human	IGKV3-11
L3-9,10-A	CDRL3	1FGV	L	QQGNTLPPT	human	IGKV1-33
L3-9,10-A	CDRL3	1DBA	L	SQSSHVPPT	house mouse	IGKV1-110
L3-9,10-A	CDRL3	2FX8	N	QQYGQSLST	human	IGKV3-20
L3-9,10-A	CDRL3	2AK1	L	QQFVEYPFT	house mouse	IGKV2-112
L3-9,10-A	CDRL3	1UZ6	V	AQNLEVPWT	house mouse	IGKV2-109
L3-9,10-A	CDRL3	2GK0	A	FQGSHLPPT	house mouse	IGKV1-117
L3-9,10-A	CDRL3	4CAD	A	QHFWSTPWT	house mouse	IGKV12-41
L3-9,10-A	CDRL3	3LHP	M	QQYGQSLST	human	IGKV3-20
L3-9,10-A	CDRL3	1UM6	L	LQYASYPRT	house mouse	IGKV9-124
L3-9,10-A	CDRL3	3CDF	B	QQYDNLPYT	human	IGKV1-33
L3-9,10-A	CDRL3	2DQT	L	FQGSHVPPT	house mouse	IGKV1-117
L3-9,10-A	CDRL3	4AEI	L	FQGSHVPLT	house mouse	IGKV1-117
L3-9,10-A	CDRL3	3EYV	L	FQGSHVPFT	house mouse	IGKV1-117
L3-9,10-A	CDRL3	3HR5	P	SQNTLVPWT	house mouse	IGKV
L3-9,10-A	CDRL3	2JEL	L	FQGSHVPYT	house mouse	IGKV1-117
L3-9,10-A	CDRL3	4H20	L	QQHYGTPLT	house mouse	IGKV6-17
L3-9,10-A	CDRL3	1YJD	L	QQGQTYPYT	house mouse	IGKV10-94
L3-9,10-A	CDRL3	43C9	G	QQHYRAPRT	house mouse	IGKV8-24

L3-9,10-A	CDRL3	3T3M	L	VQYAQLPYT	house mouse	IGKV14-100
L3-9,10-A	CDRL3	2VXT	L	LQYASSPYT	house mouse	IGKV9-120
L3-9,10-A	CDRL3	1YNL	L	SQGTHVPYT	house mouse	IGKV1-110
L3-9,10-A	CDRL3	1LVE	A	QQYYSTPYS	human	IGKV4-1
L3-9,10-A	CDRL3	1YMH	C	KQAYIPPLT	house mouse	IGKV8-21
L3-9,10-A	CDRL3	3GGW	A	AHNVELPRT	house mouse	IGKV2-109
L3-9,10-A	CDRL3	3HR5	A	SQNTLVPWT	house mouse	IGKV
L3-9,10-A	CDRL3	1P2C	A	QQSGSWPRT	house mouse	IGKV5-43
L3-9,10-A	CDRL3	3P0Y	L	QQSEPEPYT	human	IGKV1-5
L3-9,10-A	CDRL3	1BFO	G	LQHISRPRT	Norway rat	IGKV22S7
L3-9,10-A	CDRL3	1XGR	A	QQSNSWPYT	house mouse	IGKV5-43
L3-9,10-A	CDRL3	1EEU	B	DQYYSHPYS	human	IGKV4-1
L3-9,10-A	CDRL3	2AJY	L	QQFVEYPFT	house mouse	IGKV2-112
L3-9,10-A	CDRL3	1AD0	C	QHWSSKPPT	human	IGKV1-16
L3-9,10-A	CDRL3	2QQN	L	QQYLGSPPT	human	IGKV1-5
L3-9,10-A	CDRL3	1UYW	N	GQGYSYPYT	house mouse	IGKV6-20
L3-9,10-A	CDRL3	1C08	A	QQSNSWPYT	house mouse	IGKV5-43
L3-9,10-A	CDRL3	1J05	A	QQTNEDPYT	house mouse	IGKV3-5
L3-9,10-A	CDRL3	2XKN	C	QQGNTLPWT	house mouse	IGKV10-96
L3-9,10-A	CDRL3	4HXB	L	FQASHDPPT	house mouse	IGKV1-117
L3-9,10-A	CDRL3	2YBR	H	QQYRYSPRT	human	IGKV3D-11
L3-9,10-A	CDRL3	1G7L	A	QHFSSTPRT	house mouse	IGKV12-41
L3-9,10-A	CDRL3	2HKF	L	SQSTHVPFT	house mouse	IGKV1-110
L3-9,10-A	CDRL3	4LLV	L	QQYGQSLST	human	IGKV3-20
L3-9,10-A	CDRL3	2J4W	L	QQWSSHPPT	house mouse	IGKV4-72
L3-9,10-A	CDRL3	3UPA	A	QQSYSTPNT	human	IGKV1-39
L3-9,10-A	CDRL3	1UWX	L	QQLSSTPYT	house mouse	IGKV12-98
L3-9,10-A	CDRL3	4GMT	M	QQYNSYPYT	house mouse	IGKV6-15
L3-9,10-A	CDRL3	2Q8A	L	QQGFSSPRT	house mouse	IGKV6-32
L3-9,10-A	CDRL3	4K3J	L	QQYYAYPWT	human	IGKV1D-13
L3-9,10-A	CDRL3	3QCT	L	SQSTHFPFT	house mouse	IGKV
L3-9,10-A	CDRL3	2IMM	A	QNDHSYPLT	house mouse	IGKV8-28
L3-9,10-A	CDRL3	2OK0	L	FQGSHIPLT	house mouse	IGKV1-117
L3-9,10-A	CDRL3	1BRE	B	QQYDDLPYT	human	IGKV1-33
L3-9,10-A	CDRL3	1T2Q	L	FQGSHVPLT	house mouse	IGKV1-117
L3-9,10-A	CDRL3	1FNS	L	QQYEKLPWT	house mouse	IGKV10-94
L3-9,10-A	CDRL3	2XA8	L	QQSHEDPYT	house mouse	IGKV3
L3-9,10-A	CDRL3	4LAS	L	HQWSSFPFT	house mouse	IGKV4-79
L3-9,10-A	CDRL3	1J05	L	QQTNEDPYT	house mouse	IGKV3-5
L3-9,10-A	CDRL3	2VL5	D	QQSNEDPYT	house mouse	IGKV3-5
L3-9,10-A	CDRL3	3HC0	B	QQYDTYPFT	human	IGKV1-16
L3-9,10-A	CDRL3	2FX8	O	QQYGQSLST	human	IGKV3-20
L3-9,10-A	CDRL3	1GHF	L	QQGNTLPYT	house mouse	IGKV10-96
L3-9,10-A	CDRL3	3EOT	L	QQISGNPWT	house mouse	IGKV4-68
L3-9,10-A	CDRL3	4GAG	L	QQNNVDPWT	house mouse	IGKV3-10
L3-9,10-A	CDRL3	1AD9	A	MQHLEYPFT	house mouse	IGKV
L3-9,10-A	CDRL3	3LIZ	L	LQYDELPYT	house mouse	IGKV14-111
L3-9,10-A	CDRL3	3BKJ	L	FQASLVPLT	house mouse	IGKV1-117
L3-9,10-A	CDRL3	3EYU	L	FQGSHVPLT	house mouse	IGKV1-117
L3-9,10-A	CDRL3	4LEX	L	SQSTHVPWT	house mouse	IGKV1-110

L3-9,10-A	CDRL3	3U30	B	QQSYTTPPT	human	IGKV1-39
L3-9,10-A	CDRL3	3IDG	A	QQLHFYPHT	human	IGKV1-13
L3-9,10-A	CDRL3	1ZA6	E	QQYYSYPLT	house mouse	IGKV
L3-9,10-A	CDRL3	1J1O	L	QQSNSWPYT	house mouse	IGKV5-43
L3-9,10-A	CDRL3	4LF3	D	LQHNSNPLT	house mouse	IGKV11-125
L3-9,10-A	CDRL3	3LEX	B	WQGTHFPWT	house mouse	IGKV1-135
L3-9,10-A	CDRL3	1OTS	F	QQWSSHPQT	house mouse	IGKV4-59
L3-9,10-A	CDRL3	1NSN	L	QHSWEIPYT	house mouse	IGKV3-7
L3-9,10-A	CDRL3	4HS6	L	QQNNVDPWT	house mouse	IGKV3
L3-9,10-A	CDRL3	1S3K	L	FQGSHVPFT	house mouse	IGKV
L3-9,10-A	CDRL3	1OSP	L	QQYWSPPPT	house mouse	IGKV13-84
L3-9,10-A	CDRL3	3O41	L	QQIIEDPWT	house mouse	IGKV3-3
L3-9,10-A	CDRL3	1U8L	A	QQLHFYPHT	human	IGKV1D-13
L3-9,10-A	CDRL3	1XGT	A	QQSNSWPYT	house mouse	IGKV5-43
L3-9,10-A	CDRL3	4HCR	L	MQNIQLPWT	house mouse	IGKV
L3-9,10-A	CDRL3	1UAC	L	QQSNSWPYT	house mouse	IGKV5-43
L3-9,10-A	CDRL3	1MLC	C	QQSNSWPRT	house mouse	IGKV5-43
L3-9,10-A	CDRL3	3KYM	G	QQYDKWPLT	human	IGKV3-15
L3-9,10-A	CDRL3	4HH9	A	QQRGNWPPIT	human	IGKV3-11
L3-9,10-A	CDRL3	3V0V	L	LQGQSYPRT	house mouse	IGKV11-125
L3-9,10-A	CDRL3	4JN1	L	LQSTHFPHT	house mouse	IGKV1-133
L3-9,10-A	CDRL3	3C5S	A	AHNVELPRT	house mouse	IGKV2-109
L3-9,10-A	CDRL3	2V7H	L	QQGNTLPRT	house mouse	IGKV10-96
L3-9,10-A	CDRL3	3A6B	L	QQSNSWPYT	house mouse	IGKV5-43
L3-9,10-A	CDRL3	3U9P	L	LQYDNLPYM	Norway rat	IGKV19S2
L3-9,10-A	CDRL3	3EYV	A	FQGSHVPFT	house mouse	IGKV1-117
L3-9,10-A	CDRL3	3FCT	C	QQYSSYPLT	house mouse	IGKV6-13
L3-9,10-A	CDRL3	1C12	A	VQYVQFPFT	house mouse	IGKV14-100
L3-9,10-A	CDRL3	1MF2	M	QQSKEVPWT	house mouse	IGKV3-2
L3-9,10-A	CDRL3	2VH5	L	QQSVMIPMT	human	IGKV1-39
L3-9,10-A	CDRL3	3OJD	A	QHFWSTPFT	house mouse	IGKV12-41
L3-9,10-A	CDRL3	1IGF	L	FQGSHVPPT	house mouse	IGKV1-117
L3-9,10-A	CDRL3	3UJT	L	QQHYSTPFT	house mouse	IGKV8-24
L3-9,10-A	CDRL3	4CAD	G	QHFWSTPWT	house mouse	IGKV12-41
L3-9,10-A	CDRL3	1RHH	A	QQYGTSPYT	human	IGKV3-20
L3-9,10-A	CDRL3	1F90	L	VQGTHFPRT	house mouse	IGKV1-133
L3-9,10-A	CDRL3	3QCU	M	SQSTHFPFT	house mouse	IGKV
L3-9,10-A	CDRL3	1QP1	B	QQYDDLPYT	human	IGKV1-33
L3-9,10-A	CDRL3	4HS6	A	QQNNVDPWT	house mouse	IGKV3
L3-9,10-A	CDRL3	2VDR	L	VQYAQLPYT	house mouse	IGKV14-100
L3-9,10-A	CDRL3	1P2C	D	QQSGSWPRT	house mouse	IGKV5-43
L3-9,10-A	CDRL3	2DLF	L	SQSTHVPFT	house mouse	IGKV1-110
L3-9,10-A	CDRL3	3Q3G	F	QQYYSYPLT	house mouse	IGKV8-30
L3-9,10-A	CDRL3	1XGY	M	GQMLEHPLT	house mouse	IGKV2-109
L3-9,10-A	CDRL3	1XGQ	A	QQSNSWPYT	house mouse	IGKV5-43
L3-9,10-A	CDRL3	2H1P	L	SQSTHVPWT	house mouse	IGKV1-110
L3-9,10-A	CDRL3	3U0T	A	LQGTHYPVL	house mouse	IGKV
L3-9,10-A	CDRL3	1BFO	E	LQHISRPRT	Norway rat	IGKV22S7
L3-9,10-A	CDRL3	1RZG	B	QQHDSSPYT	human	IGKV1-5
L3-9,10-A	CDRL3	1RMF	L	SQSTHVPLT	house mouse	IGKV1-110

L3-9,10-A	CDRL3	1GGI	M	QQSNEDPLT	house mouse	IGKV3-5
L3-9,10-A	CDRL3	1IC4	L	QQSNSWPYT	house mouse	IGKV5-43
L3-9,10-A	CDRL3	3GGW	C	AHNVELPRT	house mouse	IGKV2-109
L3-9,10-A	CDRL3	2R56	M	QQYHSYPWT	human	IGKV1D-16
L3-9,10-A	CDRL3	3L7E	A	QQHNEYPYT	house mouse	IGKV16-104
L3-9,10-A	CDRL3	3G5X	A	VQYAQFPWT	house mouse	IGKV14-100
L3-9,10-A	CDRL3	1V7M	L	QQRSGYPRT	house mouse	IGKV4-57
L3-9,10-A	CDRL3	2P8L	A	QQLHFYPHT	human	IGKV1D-13
L3-9,10-A	CDRL3	3L1O	L	QQHNDYPLT	house mouse	IGKV16-104
L3-9,10-A	CDRL3	1CIC	A	QHCGSYPFT	house mouse	IGKV6-23
L3-9,10-A	CDRL3	4ETQ	B	QQWTSYPLT	house mouse	IGKV4-55
L3-9,10-A	CDRL3	3FZU	L	LQVYSTPRT	human	IGKV1-39
L3-9,10-A	CDRL3	4F33	A	QQWSKHPLT	house mouse	IGKV4-59
L3-9,10-A	CDRL3	1QFU	L	SQNTHVPYT	house mouse	IGKV1-110
L3-9,10-A	CDRL3	1NCB	L	QQHYSPPWT	house mouse	IGKV6-25
L3-9,10-A	CDRL3	2P8M	A	QQLHFYPHT	human	IGKV1D-13
L3-9,10-A	CDRL3	1IQW	L	QQSNEDPRT	house mouse	IGKV3-4
L3-9,10-A	CDRL3	1XF3	A	QHHYGTPLT	house mouse	IGKV12-44
L3-9,10-A	CDRL3	3LS5	L	QQGSSIPLT	house mouse	IGKV4-91
L3-9,10-A	CDRL3	2FJF	J	QQSYTTPPT	human	IGKV1-39
L3-9,10-A	CDRL3	3IDM	A	QQLHFYPHT	human	IGKV1-13
L3-9,10-A	CDRL3	3BKY	L	QQWSFNPPT	house mouse	IGKV4-72
L3-9,10-A	CDRL3	1YY8	C	QQNNNWPTT	house mouse	IGKV5-48
L3-9,10-A	CDRL3	4LLU	D	QQYYIYPYT	human	IGKV1-16
L3-9,10-A	CDRL3	1XF4	A	QHHYGTPLT	house mouse	IGKV12-44
L3-9,10-A	CDRL3	2CGR	L	SQGTHVPYT	house mouse	IGKV1-110
L3-9,10-A	CDRL3	3LHP	L	QQYGQSLST	human	IGKV3-20
L3-9,10-A	CDRL3	2DQI	L	QQSNSWPYT	house mouse	IGKV5-43
L3-9,10-A	CDRL3	1RZG	D	QQHDSSPYT	human	IGKV1-5
L3-9,10-A	CDRL3	1R3I	L	QQSNRWPFT	house mouse	IGKV5-48
L3-9,10-A	CDRL3	2A1W	L	FQGSHIPWT	house mouse	IGKV1-117
L3-9,10-A	CDRL3	1N64	L	KQAYIPPLT	house mouse	IGKV8-21
L3-9,10-A	CDRL3	3QPQ	L	QHFWSTPFT	house mouse	IGKV12-41
L3-9,10-A	CDRL3	4FQL	L	QQYGSSPWT	human	IGKV3-20
L3-9,10-A	CDRL3	3U9P	M	LQYDNLPYM	Norway rat	IGKV19S2
L3-9,10-A	CDRL3	2HVJ	B	QQSNRWPFT	house mouse	IGKV5-48
L3-9,10-A	CDRL3	3UTZ	E	FQASHVPPT	house mouse	IGKV1-117
L3-9,10-A	CDRL3	1XGU	A	QQSNSWPYT	house mouse	IGKV5-43
L3-9,10-A	CDRL3	1MHH	C	KQAYIPPLT	house mouse	IGKV8-21
L3-9,10-A	CDRL3	1ZA6	A	QQYYSYPLT	house mouse	IGKV
L3-9,10-A	CDRL3	1MCP	L	QNDHSYPLT	house mouse	IGKV8-28
L3-9,10-A	CDRL3	1F3D	J	FQGSHVPYT	house mouse	IGKV1-117
L3-9,10-A	CDRL3	4K2U	M	QQGSTFPWT	house mouse	IGKV10-96
L3-9,10-A	CDRL3	1DEE	E	QQSYSAPRT	human	IGKV1-39
L3-9,10-A	CDRL3	2YBR	E	QQYRYSPRT	human	IGKV3D-11
L3-9,10-A	CDRL3	1ZAN	L	QHYFGYPRT	Norway rat	IGKV12S34
L3-9,10-A	CDRL3	1MJ8	L	LQHLEYPFT	house mouse	IGKV2-137
L3-9,10-A	CDRL3	3MXV	L	QQDYGSPPT	house mouse	IGKV6-32
L3-9,10-A	CDRL3	2FX9	M	QQYGQSLST	human	IGKV3-20
L3-9,10-A	CDRL3	2DQF	A	QQSNSWPYT	house mouse	IGKV5-43

L3-9,10-A	CDRL3	2Q8B	L	QQGFSSPRT	house mouse	IGKV6-32
L3-9,10-A	CDRL3	1IC5	L	QQSNSWPYT	house mouse	IGKV5-43
L3-9,10-A	CDRL3	3L5W	A	QQHNEYPYT	house mouse	IGKV16-104
L3-9,10-A	CDRL3	1NGX	A	QQYSSYPLT	house mouse	IGKV6-13
L3-9,10-A	CDRL3	1J1P	L	QQANSWPYT	house mouse	IGKV5-43
L3-9,10-A	CDRL3	1ZA6	C	QQYYSYPLT	house mouse	IGKV
L3-9,10-A	CDRL3	1A7N	L	QHFWSTPRT	house mouse	IGKV12-41
L3-9,10-A	CDRL3	1YEK	L	VQGTHFPYT	house mouse	IGKV1-133
L3-9,10-A	CDRL3	3EOA	L	QQHNEYPLT	human	IGKV1-16
L3-9,10-A	CDRL3	3NID	L	VQYAQLPYT	house mouse	IGKV14-100
L3-9,10-A	CDRL3	2V7H	A	QQGNTLPRT	house mouse	IGKV10-96
L3-9,10-A	CDRL3	3EYS	L	FQGSHVPLT	house mouse	IGKV1-117
L3-9,10-A	CDRL3	1UZ8	L	AQNLEVPWT	house mouse	IGKV2-109
L3-9,10-A	CDRL3	3NTC	L	QNDYSYPWT	house mouse	IGKV8-19
L3-9,10-A	CDRL3	4G7Y	L	QQYFYWPIT	human	IGKV1-5
L3-9,10-A	CDRL3	3RA7	M	QQSITLPPT	house mouse	IGKV10-96
L3-9,10-A	CDRL3	1TJH	L	QQLHFYPHT	human	IGKV1D-13
L3-9,10-A	CDRL3	1YEJ	L	VQGTHFPYT	house mouse	IGKV1-133
L3-9,10-A	CDRL3	2CMR	L	QQYSNYPLT	human	IGKV1-5
L3-9,10-A	CDRL3	2NYY	C	QQGNEVPFT	house mouse	IGKV3-5
L3-9,10-A	CDRL3	1BWW	A	QQYQSLPYT	human	IGKV1-33
L3-9,10-A	CDRL3	1MPA	L	SQSTHVPRT	house mouse	IGKV1-110
L3-9,10-A	CDRL3	1LO3	L	FQGSHFPLA	house mouse	IGKV1-117
L3-9,10-A	CDRL3	2BX5	C	QQSYSTPNT	human	IGKV1-39
L3-9,10-A	CDRL3	2QQK	L	QQAWAYLPT	human	IGKV1D-13
L3-9,10-A	CDRL3	1BLN	A	FQASHAPRT	house mouse	IGKV1-117
L3-9,10-A	CDRL3	1KN4	L	VQGTHFPYT	house mouse	IGKV1-133
L3-9,10-A	CDRL3	2PCP	C	FQGTHAPYT	house mouse	IGKV1-117
L3-9,10-A	CDRL3	3AAZ	L	FQASLVPLT	house mouse	IGKV
L3-9,10-A	CDRL3	3B2U	O	HQYGSTPLT	human	IGKV3D-11
L3-9,10-A	CDRL3	1QAC	B	LQYYSTPYS	human	IGKV4-1
L3-9,10-A	CDRL3	1I8K	A	LQSFNVPLT	house mouse	IGKV17-127
L3-9,10-A	CDRL3	1DVF	A	QHFWSTPRT	house mouse	IGKV12-41
L3-9,10-A	CDRL3	15C8	L	HQYHRSPYT	house mouse	IGKV4-74
L3-9,10-A	CDRL3	1U8M	A	QQLHFYPHT	human	IGKV1D-13
L3-9,10-A	CDRL3	2Q1E	D	QQYDNLPYT	human	IGKV1-33
L3-9,10-A	CDRL3	1R0A	L	QQYSKFPWT	house mouse	IGKV10-94
L3-9,10-A	CDRL3	1CLO	L	QHWSSKPPT	house mouse	IGKV4-72
L3-9,10-A	CDRL3	2AJS	L	QQFVEYPFT	house mouse	IGKV2-112
L3-9,10-A	CDRL3	2DWD	B	QQSNRWPFT	house mouse	IGKV5-48
L3-9,10-A	CDRL3	2OJZ	L	FQGSHIPLT	house mouse	IGKV1-117
L3-9,10-A	CDRL3	3HC3	L	QQYDTYPFT	human	IGKV1-16
L3-9,10-A	CDRL3	3D0V	A	QQLHFYPHT	human	IGKV1D-13
L3-9,10-A	CDRL3	1WEJ	L	QHFWSTPWT	house mouse	IGKV12-41
L3-9,10-A	CDRL3	1A2Y	A	QHFWSTPRT	house mouse	IGKV12-41
L3-9,10-A	CDRL3	3V0V	B	LQGQSYPRT	house mouse	IGKV11-125
L3-9,10-A	CDRL3	2BX5	N	QQSYSTPNT	human	IGKV1-39
L3-9,10-A	CDRL3	3BKC	L	FQASLVPLT	house mouse	IGKV1-117
L3-9,10-A	CDRL3	2BX5	G	QQSYSTPNT	human	IGKV1-39
L3-9,10-A	CDRL3	3NIF	L	VQYAQLPYT	house mouse	IGKV14-100

L3-9,10-A	CDRL3	1BBD	L	QNNYNYPLT	house mouse	IGKV8-19
L3-9,10-A	CDRL3	3DRQ	A	QQLHFYPHT	human	IGKV1D-13
L3-9,10-A	CDRL3	2BX5	D	QQSYSTPNT	human	IGKV1-39
L3-9,10-A	CDRL3	4GAY	L	QQNNVDPWT	house mouse	IGKV3-10
L3-9,10-A	CDRL3	1I8M	L	QHHYGTPLT	house mouse	IGKV12-44
L3-9,10-A	CDRL3	1IGI	L	SQTTHVPPT	house mouse	IGKV1-110
L3-9,10-A	CDRL3	1C1E	L	FQSTHFFPT	house mouse	IGKV1-110
L3-9,10-A	CDRL3	1BJ1	J	QQYSTVPWT	human	IGKV1-33
L3-9,10-A	CDRL3	3L7F	L	QQHNEYPYT	human	IGKV3-11
L3-9,10-A	CDRL3	1BJ1	L	QQYSTVPWT	human	IGKV1-33
L3-9,10-A	CDRL3	2FJF	E	QQSYTTPPT	human	IGKV1-39
L3-9,10-A	CDRL3	1FVC	C	QQHYTTPPT	human	IGKV1-39
L3-9,10-A	CDRL3	1EJO	L	QQSNEDPLT	house mouse	IGKV3-5
L3-9,10-A	CDRL3	2FX8	L	QQYGQSLST	human	IGKV3-20
L3-9,10-A	CDRL3	2AJU	L	QQFVEYPFT	house mouse	IGKV2-112
L3-9,10-A	CDRL3	1K4D	B	QQSNRWPFT	house mouse	IGKV5-48
L3-9,10-A	CDRL3	3LH2	O	QQYGQSLST	human	IGKV3-20
L3-9,10-A	CDRL3	1UJ3	A	LQHGESPYT	human	IGKV1D-16
L3-9,10-A	CDRL3	1G7J	A	QHFHSTPRT	house mouse	IGKV12-41
L3-9,10-A	CDRL3	2BX5	A	QQSYSTPNT	human	IGKV1-39
L3-9,10-A	CDRL3	3L95	A	QQFYTTPST	human	IGKV1-39
L3-9,10-A	CDRL3	3PP3	K	AQNLELPYT	house mouse	IGKV
L3-9,10-A	CDRL3	3E8U	L	QQSNEDPFT	house mouse	IGKV3-4
L3-9,10-A	CDRL3	2G2R	L	LQSTHFPLT	house mouse	IGKV1-133
L3-9,10-A	CDRL3	1REI	A	QQYQSLPYT	human	IGKV1-33
L3-9,10-A	CDRL3	3MCK	L	FQGSRVPLT	house mouse	IGKV1-117
L3-9,10-A	CDRL3	4F33	G	QQWSKHPLT	house mouse	IGKV4-59
L3-9,10-A	CDRL3	1WCB	A	QQRSTYPFT	house mouse	IGKV4-57
L3-9,10-A	CDRL3	3EFD	L	QQYSYSLLT	human	IGKV1-39
L3-9,10-A	CDRL3	4NRX	B	QQSYSTPFT	human	IGKV1-39
L3-9,10-A	CDRL3	2PW1	A	QQLHFYPHT	human	IGKV1D-13
L3-9,10-A	CDRL3	1DSF	L	SQSTHVPLT	house mouse	IGKV1-110
L3-9,10-A	CDRL3	1KIP	A	QHFWSTPRT	house mouse	IGKV12-41
L3-9,10-A	CDRL3	3MOB	L	QQLHFYPHT	human	IGKV1-13
L3-9,10-A	CDRL3	2FR4	A	QHHYGTPLT	house mouse	IGKV12-44
L3-9,10-A	CDRL3	2YPV	L	LQYDEFPPT	house mouse	IGKV14-111
L3-9,10-A	CDRL3	2BX5	B	QQSYSTPNT	human	IGKV1-39
L3-9,10-A	CDRL3	3RKD	C	QHFWGNPWT	house mouse	IGKV12-46
L3-9,10-A	CDRL3	1XGP	A	QQSNSWPYT	house mouse	IGKV5-43
L3-9,10-A	CDRL3	1FL5	A	FQGSHVPRT	house mouse	IGKV1-117
L3-9,10-A	CDRL3	4LLV	B	QQYGQSLST	human	IGKV3-20
L3-9,10-A	CDRL3	2IPU	L	FQGSHVPLT	house mouse	IGKV1-117
L3-9,10-A	CDRL3	4LAQ	L	HQWSSFPFT	house mouse	IGKV4-79
L3-9,10-A	CDRL3	1F3D	L	FQGSHVPYT	house mouse	IGKV1-117
L3-9,10-A	CDRL3	1FOR	L	QQRSSYPIT	house mouse	IGKV4-57
L3-9,10-A	CDRL3	2WUC	L	QQSNRAPAT	human	IGKV1D-13
L3-9,10-A	CDRL3	2AEP	L	QQFNRYPLT	house mouse	IGKV6-15
L3-9,10-A	CDRL3	3H3P	M	QQYGQSLST	human	IGKV3-20
L3-9,10-A	CDRL3	4N9G	N	QQHNSYPRT	human	IGKV1-16
L3-9,10-A	CDRL3	1WT5	D	FQGSHIPPT	house mouse	IGKV

L3-9,10-A	CDRL3	1DQM	L	QQSNSWPYT	house mouse	IGKV5-43
L3-9,10-A	CDRL3	1I8M	A	QHHYGTPLT	house mouse	IGKV12-44
L3-9,10-A	CDRL3	2FJF	Q	QQSYTTPPT	human	IGKV1-39
L3-9,10-A	CDRL3	1LO4	L	FQGSHSPWT	house mouse	IGKV1-117
L3-9,10-A	CDRL3	1NMC	L	QQDFTLPFT	house mouse	IGKV10-96
L3-9,10-A	CDRL3	3D0L	A	QQLHFYPHT	human	IGKV1D-13
L3-9,10-A	CDRL3	3MXW	L	QQDYGSPPT	human	IGKV1-33
L3-9,10-A	CDRL3	3MCK	A	FQGSRVPLT	house mouse	IGKV1-117
L3-9,10-A	CDRL3	3EYO	C	QQRSMWPPVT	human	IGKV3-11
L3-9,10-A	CDRL3	3HR5	Q	SQNTLVPWT	house mouse	IGKV
L3-9,10-A	CDRL3	1MLB	A	QQSNSWPRT	house mouse	IGKV5-43
L3-9,10-A	CDRL3	1CBV	L	SQSTHVPLT	house mouse	IGKV1-110
L3-9,10-A	CDRL3	4LMQ	L	KQYWNTPFT	human	IGKV1-5
L3-9,10-A	CDRL3	1E4X	M	QQGGALPFT	house mouse	IGKV10-96
L3-9,10-A	CDRL3	1C5B	L	LQYASFPRT	house mouse	IGKV9-124
L3-9,10-A	CDRL3	1DQD	L	QQRSTYPPT	house mouse	IGKV4-57
L3-9,10-A	CDRL3	3KR3	L	QQSYNSPWT	human	IGKV1-39
L3-9,10-A	CDRL3	3NAC	L	QQGNTLSYT	human	IGKV1-39
L3-9,10-A	CDRL3	1EEU	A	DQYYSHPYS	human	IGKV4-1
L3-9,10-A	CDRL3	2EH7	L	VQGTHFPQT	house mouse	IGKV1-133
L3-9,10-A	CDRL3	3QNZ	A	MQNKQTPLT	human	IGKV2-28
L3-9,10-A	CDRL3	1MH5	L	LQHLEYPFT	house mouse	IGKV2-137
L3-9,10-A	CDRL3	2FJF	U	QQSYTTPPT	human	IGKV1-39
L3-9,10-A	CDRL3	1WTL	B	QQYDTLPLT	human	IGKV1-33
L3-9,10-A	CDRL3	1TJI	L	QQLHFYPHT	human	IGKV1D-13
L3-9,10-A	CDRL3	3CXD	L	QHFWGTPFT	house mouse	IGKV12-46
L3-9,10-A	CDRL3	1S5H	A	QQSNRWPFT	house mouse	IGKV5-48
L3-9,10-A	CDRL3	1BVK	A	QHFWSTPRT	human	IGKV1-27
L3-9,10-A	CDRL3	1FE8	N	QNGGTNPWT	house mouse	IGKV10-96
L3-9,10-A	CDRL3	4JFX	L	LQSFNVPLT	human	IGKV3-11
L3-9,10-A	CDRL3	2IQA	L	FQGSHVPLT	house mouse	IGKV1-117
L3-9,10-A	CDRL3	4NRX	E	QQSYSTPFT	human	IGKV1-39
L3-9,10-A	CDRL3	2VXU	M	LQYASSPYT	house mouse	IGKV9-120
L3-9,10-A	CDRL3	3L7E	L	QQHNEYPYT	house mouse	IGKV16-104
L3-9,10-A	CDRL3	1BRE	D	QQYDDLPYT	human	IGKV1-33
L3-9,10-A	CDRL3	1A4K	L	SQVTHVPPT	house mouse	IGKV1-110
L3-9,10-A	CDRL3	3R06	L	QQYYNYPWT	house mouse	IGKV14-111
L3-9,10-A	CDRL3	3BZ4	G	AHNVELPRT	house mouse	IGKV2-109
L3-9,10-A	CDRL3	2OSL	B	QQWTSNPPT	house mouse	IGKV4-72
L3-9,10-A	CDRL3	3ZKN	L	QQNNEDPWT	house mouse	IGKV3-10
L3-9,10-A	CDRL3	1HQ4	C	LQWSSFPYT	house mouse	IGKV4-79
L3-9,10-A	CDRL3	2Q20	A	QQYDNLPYT	human	IGKV1-33
L3-9,10-A	CDRL3	3D9A	L	QQSNSWPYT	house mouse	IGKV5-43
L3-9,10-A	CDRL3	1IGF	M	FQGSHVPPT	house mouse	IGKV1-117
L3-9,10-A	CDRL3	1IT9	L	QQSNEDPRT	house mouse	IGKV3-4
L3-9,10-A	CDRL3	1U8K	A	QQLHFYPHT	human	IGKV1D-13
L3-9,10-A	CDRL3	1UM4	L	LQYASYPRT	house mouse	IGKV9-124
L3-9,10-A	CDRL3	3RVT	C	LQYDEFPYT	house mouse	IGKV14-111
L3-9,10-A	CDRL3	1CLY	L	FQGSHVPFT	house mouse	IGKV1-117
L3-9,10-A	CDRL3	3CDY	B	QQYDNLPYT	human	IGKV1-33

L3-9,10-A	CDRL3	1FGN	L	LQHGESPYT	house mouse	IGKV14-126
L3-9,10-A	CDRL3	2ZKH	L	QQRSGYPRT	house mouse	IGKV4-57
L3-9,10-A	CDRL3	2UYL	A	FQGSHVPRT	house mouse	IGKV1-117
L3-9,10-A	CDRL3	1MHP	Y	QQWSGNPWT	human	IGKV1D-13
L3-9,10-A	CDRL3	3PP4	L	AQNLELPYT	house mouse	IGKV
L3-9,10-A	CDRL3	1MH5	A	LQHLEYPFT	house mouse	IGKV2-137
L3-9,10-A	CDRL3	2V7N	E	HQYSDISPT	human	IGKV3-11
L3-9,10-A	CDRL3	1LO2	X	FQGSHFPLA	house mouse	IGKV1-117
L3-9,10-A	CDRL3	1LO3	X	FQGSHFPLA	house mouse	IGKV1-117
L3-9,10-A	CDRL3	43C9	A	QQHYRAPRT	house mouse	IGKV8-24
L3-9,10-A	CDRL3	3OR7	B	QQSNRWPFT	house mouse	IGKV5-48
L3-9,10-A	CDRL3	3M8O	L	MQNKQTPLT	human	IGKV2-28
L3-9,10-A	CDRL3	3FO1	A	FQGSHLPPT	house mouse	IGKV1-117
L3-9,10-A	CDRL3	4JN2	L	LQSTHFPHT	house mouse	IGKV1-133
L3-9,10-A	CDRL3	1I9J	L	FQGSHVPPT	house mouse	IGKV1-117
L3-9,10-A	CDRL3	1K6Q	L	LQHGESPFT	house mouse	IGKV14-126
L3-9,10-A	CDRL3	2BX5	K	QQSYSTPNT	human	IGKV1-39
L3-9,10-A	CDRL3	4JHA	L	QQYDNLPLT	human	IGKV1-33
L3-9,10-A	CDRL3	1MJU	L	LQHLEYPFT	house mouse	IGKV2-137
L3-9,10-A	CDRL3	3QRG	L	QQIIEDPWT	house mouse	IGKV3
L3-9,10-A	CDRL3	3FO1	L	FQGSHLPPT	house mouse	IGKV1-117
L3-9,10-A	CDRL3	2BX5	L	QQSYSTPNT	human	IGKV1-39
L3-9,10-A	CDRL3	3IXT	L	FQGSGYPFT	human	IGKV1-5
L3-9,10-A	CDRL3	3A6C	L	QQSDSWPYT	house mouse	IGKV5-43
L3-9,10-A	CDRL3	1DLF	L	SQSTHVPFT	house mouse	IGKV1-110
L3-9,10-A	CDRL3	2Q76	C	QQSGSWPRT	house mouse	IGKV5-43
L3-9,10-A	CDRL3	1NMB	L	QQDFTLPFT	house mouse	IGKV10-96
L3-9,10-A	CDRL3	3SGD	I	WQGSHFPYT	house mouse	IGKV1-135
L3-9,10-A	CDRL3	3MOA	L	QQLHFYPHT	human	IGKV1-13
L3-9,10-A	CDRL3	3IXT	B	FQGSGYPFT	human	IGKV1-5
L3-9,10-A	CDRL3	1GGI	L	QQSNEDPLT	house mouse	IGKV3-5
L3-9,10-A	CDRL3	1IC7	L	QQSNSWPYT	house mouse	IGKV5-43
L3-9,10-A	CDRL3	4GW5	C	QQNNNWPTT	house mouse	IGKV5-48
L3-9,10-A	CDRL3	4G6A	D	QQNNVDPWT	house mouse	IGKV3
L3-9,10-A	CDRL3	2IH1	B	QQSNRWPFT	house mouse	IGKV5-48
L3-9,10-A	CDRL3	1UZ6	L	AQNLEVPWT	house mouse	IGKV2-109
L3-9,10-A	CDRL3	1NGX	L	QQYSSYPLT	house mouse	IGKV6-13
L3-9,10-A	CDRL3	4JG1	L	LQSFNVPLT	human	IGKV3-11
L3-9,10-A	CDRL3	4NRZ	B	QQSYSTPFT	human	IGKV1-39
L3-9,10-A	CDRL3	2DQC	L	QQSNSWPYT	house mouse	IGKV5-43
L3-9,10-A	CDRL3	1NAK	M	FQTTHDPYT	house mouse	IGKV1-110
L3-9,10-A	CDRL3	3B2U	K	HQYGSTPLT	human	IGKV3D-11
L3-9,10-A	CDRL3	2XQY	L	QHSRELPWT	house mouse	IGKV3-12
L3-9,10-A	CDRL3	1BRE	C	QQYDDLPYT	human	IGKV1-33
L3-9,10-A	CDRL3	1B6D	B	QQYDSLPLT	human	IGKV1-33
L3-9,10-A	CDRL3	3B2U	G	HQYGSTPLT	human	IGKV3D-11
L3-9,10-A	CDRL3	1CFS	A	LQYDDFPLT	house mouse	IGKV14-111
L3-9,10-A	CDRL3	3QO0	A	MQNKQTPLT	human	IGKV2-28
L3-9,10-A	CDRL3	2FJF	L	QQSYTTPPT	human	IGKV1-39
L3-9,10-A	CDRL3	1A4J	L	SQSTHVPPT	house mouse	IGKV1-110

L3-9,10-A	CDRL3	1A14	L	QQDFTLPFT	house mouse	IGKV10-96
L3-9,10-A	CDRL3	4LVE	B	QQYYSTPYS	human	IGKV4-1
L3-9,10-A	CDRL3	1RIH	L	QQSNRWPLT	house mouse	IGKV5-43
L3-9,10-A	CDRL3	1FVC	A	QQHYTTPPT	human	IGKV1-39
L3-9,10-A	CDRL3	3FZU	D	LQVYSTPRT	human	IGKV1-39
L3-9,10-A	CDRL3	4DKF	L	QQYWSEPVT	human	IGKV1-39
L3-9,10-A	CDRL3	3LEY	L	WQGTHFPWT	house mouse	IGKV1-135
L3-9,10-A	CDRL3	4KI5	F	LQYASYPYT	house mouse	IGKV9-124
L3-9,10-A	CDRL3	1XF5	C	KQAYIPPLT	house mouse	IGKV8-21
L3-9,10-A	CDRL3	3STZ	B	QQSNRWPFT	house mouse	IGKV5-48
L3-9,10-A	CDRL3	3FO2	A	FQGSHLPPT	house mouse	IGKV1-117
L3-9,10-A	CDRL3	1TJG	L	QQLHFYPHT	human	IGKV1D-13
L3-9,10-A	CDRL3	3DSF	L	QHFWGTPFT	house mouse	IGKV12-46
L3-9,10-A	CDRL3	4HHA	A	QQRGNWPPIT	human	IGKV3-11
L3-9,10-A	CDRL3	1CZ8	X	QQYSTVPWT	human	IGKV1-16
L3-9,10-A	CDRL3	4D9R	L	LQSDSLPYT	human	IGKV1-27
L3-9,10-A	CDRL3	2JK5	B	QQSNRWPFT	house mouse	IGKV5-48
L3-9,10-A	CDRL3	4LBE	B	QQSNRWPFT	house mouse	IGKV5-48
L3-9,10-A	CDRL3	3CFB	A	AQNLELPPT	house mouse	IGKV2-109
L3-9,10-A	CDRL3	3CDF	F	QQYDNLPYT	human	IGKV1-33
L3-9,10-A	CDRL3	1RJL	A	QQGQSFPLT	house mouse	IGKV11-125
L3-9,10-A	CDRL3	4CAD	J	QHFWSTPWT	house mouse	IGKV12-41
L3-9,10-A	CDRL3	4ENE	F	QQWSSHPQT	house mouse	IGKV4-59
L3-9,10-A	CDRL3	1UWG	X	QQYNLFPVT	house mouse	IGKV6-15
L3-9,10-A	CDRL3	4FFY	L	QQNNEDPYT	house mouse	IGKV3-10
L3-9,10-A	CDRL3	3PGF	L	QQSSYIPVT	human	IGKV1-39
L3-9,10-A	CDRL3	2AJZ	L	QQFVEYPFT	house mouse	IGKV2-112
L3-9,10-A	CDRL3	1F6L	L	QHHYGTPFT	human	IGKV1-39
L3-9,10-A	CDRL3	1MJJ	A	LQHLEYPFT	house mouse	IGKV2-137
L3-9,10-A	CDRL3	3HC0	L	QQYDTYPFT	human	IGKV1-16
L3-9,10-A	CDRL3	2YMX	L	QHYSTTPWT	house mouse	IGKV12-46
L3-9,10-A	CDRL3	2AJX	L	QQFVEYPFT	house mouse	IGKV2-112
L3-9,10-A	CDRL3	1MJ7	L	MQHLEYPFT	house mouse	IGKV2-137
L3-9,10-A	CDRL3	2W65	D	WQGTHFPLT	house mouse	IGKV1-135
L3-9,10-A	CDRL3	4FNL	M	QQYDGLPFT	human	IGKV1-33
L3-9,10-A	CDRL3	4N9G	F	QQHNSYPRT	human	IGKV1-16
L3-9,10-A	CDRL3	1BFO	C	LQHISRPRT	Norway rat	IGKV22S7
L3-9,10-A	CDRL3	1TQC	C	WQGSHFPQT	house mouse	IGKV1-135
L3-9,10-A	CDRL3	1NGW	A	QQYSSYPLT	house mouse	IGKV6-13
L3-9,10-A	CDRL3	4NX3	A	QQHYSTPYT	house mouse	IGKV8-24
L3-9,10-A	CDRL3	3ZDX	F	VQYAQLPYT	house mouse	IGKV14-100
L3-9,10-A	CDRL3	3MOD	L	QQLHFYPHT	human	IGKV1-13
L3-9,10-A	CDRL3	3QPQ	E	QHFWSTPFT	house mouse	IGKV12-41
L3-9,10-A	CDRL3	1HKL	L	LQYASYPRT	house mouse	IGKV9-124
L3-9,10-A	CDRL3	2G2R	A	LQSTHFPLT	house mouse	IGKV1-133
L3-9,10-A	CDRL3	3O6M	L	WQGTHFPRT	house mouse	IGKV1-135
L3-9,10-A	CDRL3	1B0W	A	QQYDDLPYT	human	IGKV1-33
L3-9,10-A	CDRL3	3QCV	M	SQSTHFPFT	house mouse	IGKV
L3-9,10-A	CDRL3	4G5Z	L	HQSSSLPFT	human	IGKV1-39
L3-9,10-A	CDRL3	3UC0	L	QQFNSYPLT	human	IGKV1-13

L3-9,10-A	CDRL3	2MPA	L	SQSTHVPRT	house mouse	IGKV1-110
L3-9,10-A	CDRL3	1JPT	L	LQHGESPWT	human	IGKV1-39
L3-9,10-A	CDRL3	2R0W	L	FQGSHVPLT	house mouse	IGKV1-117
L3-9,10-A	CDRL3	3KYM	K	QQYDKWPLT	human	IGKV3-15
L3-9,10-A	CDRL3	3WKM	L	QQNNEDPWT	house mouse	IGKV3-10
L3-9,10-A	CDRL3	3O41	B	QQIIEDPWT	house mouse	IGKV3-3
L3-9,10-A	CDRL3	3BDY	L	QQHYTTPPT	human	IGKV1-39
L3-9,10-A	CDRL3	1RUM	L	WQGTHFPYT	house mouse	IGKV1-133
L3-9,10-A	CDRL3	3KYM	M	QQYDKWPLT	human	IGKV3-15
L3-9,10-A	CDRL3	2HG5	B	QQSNRWPFT	house mouse	IGKV5-48
L3-9,10-A	CDRL3	1GGB	L	QQSNEDPLT	house mouse	IGKV3-5
L3-9,10-A	CDRL3	1U8I	A	QQLHFYPHT	human	IGKV1D-13
L3-9,10-A	CDRL3	3C6S	C	AHNVELPRT	house mouse	IGKV2-109
L3-9,10-A	CDRL3	3GK8	L	QQHYTTPWT	house mouse	IGKV6-17
L3-9,10-A	CDRL3	2AJV	L	QQFVEYPFT	house mouse	IGKV2-112
L3-9,10-A	CDRL3	3D85	A	QNGHSFPFT	house mouse	IGKV5-39
L3-9,10-A	CDRL3	4NM4	M	HQYYTAPWT	human	IGKV4-1
L3-9,10-A	CDRL3	1JGU	L	SQSTHVPPLT	house mouse	IGKV1-110
L3-9,10-A	CDRL3	1SEQ	L	HQWSSYPWT	house mouse	IGKV4-80
L3-9,10-A	CDRL3	1DN0	C	QQYGSSPLT	human	IGKV3D-20
L3-9,10-A	CDRL3	1UYW	L	GQGYSYPYT	house mouse	IGKV6-20
L3-9,10-A	CDRL3	1WZ1	L	SQSTHVPFT	house mouse	IGKV1-110
L3-9,10-A	CDRL3	3EO0	A	QQYADSPIT	human	IGKV3-20
L3-9,10-A	CDRL3	3G5Y	A	VQYGQFPWT	house mouse	IGKV14-100
L3-9,10-A	CDRL3	2V17	L	QQHNDYPLT	house mouse	IGKV16-104
L3-9,10-A	CDRL3	1PG7	M	LQHGESPWT	human	IGKV1-39
L3-9,10-A	CDRL3	2BOB	B	QQSNRWPFT	house mouse	IGKV5-48
L3-9,10-A	CDRL3	3NAA	L	QQGNTLSYT	human	IGKV1-39
L3-9,10-A	CDRL3	1BM3	L	QQSNSWPLT	house mouse	IGKV5-43
L3-9,10-A	CDRL3	2AI0	O	FQGSHIPWT	house mouse	IGKV1-117
L3-9,10-A	CDRL3	1UWE	X	QQYNIYPVT	house mouse	IGKV6-15
L3-9,10-A	CDRL3	1SBS	L	QQYHSYPFT	house mouse	IGKV8-30
L3-9,10-A	CDRL3	4LF3	A	LQHNSNPLT	house mouse	IGKV11-125
L3-9,10-A	CDRL3	2ITD	B	QQSNRWPFT	house mouse	IGKV5-48
L3-9,10-A	CDRL3	4HXA	L	FQGSGYPLT	house mouse	IGKV4-63
L3-9,10-A	CDRL3	1UB6	B	AQNLELPPT	house mouse	IGKV2-109
L3-9,10-A	CDRL3	1V7M	M	QQRSGYPRT	house mouse	IGKV4-57
L3-9,10-A	CDRL3	2D03	L	FQSSHVPLT	house mouse	IGKV1-110
L3-9,10-A	CDRL3	1MIE	L	MQHLEYPFT	house mouse	IGKV2-137
L3-9,10-A	CDRL3	2EIZ	A	QQSNSWPYT	human	IGKV3-15
L3-9,10-A	CDRL3	4G6A	L	QQNNVDPWT	house mouse	IGKV3
L3-9,10-A	CDRL3	3HNS	L	HQDTKPPYT	house mouse	IGKV10-96
L3-9,10-A	CDRL3	4G3Y	L	QQSHSWPFT	house mouse	IGKV5-48
L3-9,10-A	CDRL3	1BRE	F	QQYDDLPYT	human	IGKV1-33
L3-9,10-A	CDRL3	2FJF	W	QQSYTTPPT	human	IGKV1-39
L3-9,10-A	CDRL3	43C9	E	QQHYRAPRT	house mouse	IGKV8-24
L3-9,10-A	CDRL3	2H8P	B	QQSNRWPFT	house mouse	IGKV5-48
L3-9,10-A	CDRL3	2DQF	D	QQSNSWPYT	house mouse	IGKV5-43
L3-9,10-A	CDRL3	4HKZ	A	QQHYTTPPT	human	IGKV1-39
L3-9,10-A	CDRL3	2V7N	A	HQYSDISPT	human	IGKV3-11

L3-9,10-A	CDRL3	1H0D	A	QQSKEVPLT	house mouse	IGKV3-2
L3-9,10-A	CDRL3	4LLY	D	QQYYIYPYT	human	IGKV1D-13
L3-9,10-A	CDRL3	3KYM	E	QQYDKWPLT	human	IGKV3-15
L3-9,10-A	CDRL3	2R8S	L	QQSYSSPIT	human	IGKV1-39
L3-9,10-A	CDRL3	1NCW	L	WQGTHFPYT	house mouse	IGKV1-133
L3-9,10-A	CDRL3	2DWE	B	QQSNRWPFT	house mouse	IGKV5-48
L3-9,10-A	CDRL3	1ND0	E	WQGTHFPYT	house mouse	IGKV1-133
L3-9,10-A	CDRL3	2VQ1	A	SQTTHVPWT	house mouse	IGKV1-110
L3-9,10-A	CDRL3	3C5S	C	AHNVELPRT	house mouse	IGKV2-109
L3-9,10-A	CDRL3	2B2X	L	QQWSGNPWT	house mouse	IGKV4-68
L3-9,10-A	CDRL3	4N9G	B	QQHNSYPRT	human	IGKV1-16
L3-9,10-A	CDRL3	6FAB	L	QQGNALPRT	house mouse	IGKV10-96
L3-9,10-A	CDRL3	4ERS	L	LQHNSNPLT	human	IGKV1-17
L3-9,10-A	CDRL3	3BN9	C	QQHGNLPYT	human	IGKV1-12
L3-9,10-A	CDRL3	1UZ6	E	AQNLEVPWT	house mouse	IGKV2-109
L3-9,10-A	CDRL3	2EH8	L	VQGTHFPQT	house mouse	IGKV1-133
L3-9,10-A	CDRL3	1JV5	A	QQGNTLPWT	human	IGKV1-33
L3-9,10-A	CDRL3	2FJF	M	QQSYTTPPT	human	IGKV1-39
L3-9,10-A	CDRL3	3EYQ	C	QQRSNWPPIT	human	IGKV3-11
L3-9,10-A	CDRL3	1LO0	X	FQGSHFPLA	house mouse	IGKV1-117
L3-9,10-A	CDRL3	2DQG	L	QQSNSWPYT	house mouse	IGKV5-43
L3-9,10-A	CDRL3	2PR4	L	QQLHFYPHT	human	IGKV1D-13
L3-9,10-A	CDRL3	4NM4	L	HQYYTAPWT	human	IGKV4-1
L3-9,10-A	CDRL3	1XGY	L	GQMLEHPLT	house mouse	IGKV2-109
L3-9,10-A	CDRL3	2Z91	B	QQWSSNPPT	house mouse	IGKV4-59
L3-9,10-A	CDRL3	1CIC	C	QHFWSTPRT	house mouse	IGKV12-41
L3-9,10-A	CDRL3	3QQ9	C	QQIIEDPWT	house mouse	IGKV3-3
L3-9,10-A	CDRL3	1IFH	L	QNDYSNPLT	house mouse	IGKV8-19
L3-9,10-A	CDRL3	3IFO	B	FQGSHVPLT	house mouse	IGKV1-117
L3-9,10-A	CDRL3	1YY9	C	QQNNNWPTT	house mouse	IGKV5-48
L3-9,10-A	CDRL3	5LVE	A	AQYYSTPYS	human	IGKV4-1
L3-9,10-A	CDRL3	2P7T	B	QQSNRWPFT	house mouse	IGKV5-48
L3-9,10-A	CDRL3	3SQO	L	QQRYSLWRT	human	IGKV1D-13
L3-9,10-A	CDRL3	1Q72	L	MQHLEYPFT	house mouse	IGKV2-137
L3-9,10-A	CDRL3	3UJT	M	QQHYSTPFT	house mouse	IGKV8-24
L3-9,10-A	CDRL3	3NFS	L	HQRSTYPLT	human	IGKV1-5
L3-9,10-A	CDRL3	1LK3	M	QQSWNGPLT	Norway rat	IGKV3S11
L3-9,10-A	CDRL3	3DVF	A	QEYDYLPQT	human	IGKV1-33
L3-9,10-A	CDRL3	2R56	L	QQYHSYPWT	human	IGKV1D-16
L3-9,10-A	CDRL3	1U8Q	A	QQLHFYPHT	human	IGKV1D-13
L3-9,10-A	CDRL3	1CFQ	A	LQYDDFPLT	house mouse	IGKV14-111
L3-9,10-A	CDRL3	1UWE	L	QQYNIYPVT	house mouse	IGKV6-15
L3-9,10-A	CDRL3	1J1X	L	QQSNAWPYT	house mouse	IGKV5-43
L3-9,10-A	CDRL3	4LLY	B	QQYYIYPYT	human	IGKV1D-13
L3-9,10-A	CDRL3	1G7M	A	QHFVSTPRT	house mouse	IGKV12-41
L3-9,10-A	CDRL3	1QBL	L	QHFWSTPWT	house mouse	IGKV12-41
L3-9,10-A	CDRL3	3QG7	L	SQTTHVPYT	house mouse	IGKV1-110
L3-9,10-A	CDRL3	1DVF	C	QQGNTLPWT	house mouse	IGKV10-96
L3-9,10-A	CDRL3	4LLW	D	QQYYIYPYT	human	IGKV1-16
L3-9,10-A	CDRL3	2DQU	L	FQGSHVPPT	house mouse	IGKV1-117

L3-9,10-A	CDRL3	4FFV	L	QQWSNHPPT	house mouse	IGKV4-72
L3-9,10-A	CDRL3	1G7I	A	QHFFSTPRT	house mouse	IGKV12-41
L3-9,10-A	CDRL3	3U30	E	QQSYTTPPT	human	IGKV1-39
L3-9,10-A	CDRL3	2Q1E	A	QQYDNLPYT	human	IGKV1-33
L3-9,10-A	CDRL3	3KYM	C	QQYDKWPLT	human	IGKV3-15
L3-9,10-A	CDRL3	1NCA	L	QQHYSPPWT	house mouse	IGKV6-25
L3-9,10-A	CDRL3	2Q20	B	QQYDNLPYT	human	IGKV1-33
L3-9,10-A	CDRL3	4LMQ	I	KQYWNTPFT	human	IGKV1-5
L3-9,10-A	CDRL3	3L7F	D	QQHNEYPYT	human	IGKV3-11
L3-9,10-A	CDRL3	1JGV	L	SQSTHVPPLT	house mouse	IGKV1-110
L3-9,10-A	CDRL3	3ZDX	L	VQYAQLPYT	house mouse	IGKV14-100
L3-9,10-A	CDRL3	4F33	E	QQWSKHPLT	house mouse	IGKV4-59
L3-9,10-A	CDRL3	1HEZ	C	QQSYSTPRT	human	IGKV1-39
L3-9,10-A	CDRL3	1D5B	L	LQYDEFPYT	house mouse	IGKV14-111
L3-9,10-A	CDRL3	3S35	L	QQSNEDPLT	house mouse	IGKV3-5
L3-9,10-A	CDRL3	1E4X	L	QQGGALPFT	house mouse	IGKV10-96
L3-9,10-A	CDRL3	1D5I	L	LQYDEFPYT	house mouse	IGKV14-111
L3-9,10-A	CDRL3	2DTM	L	FQGSHVPPT	house mouse	IGKV1-117
L3-9,10-A	CDRL3	4GW5	A	QQNNNWPTT	house mouse	IGKV5-48
L3-9,10-A	CDRL3	2OR9	L	QQTKEVPWT	house mouse	IGKV3-2
L3-9,10-A	CDRL3	2VL5	B	QQSNEDPYT	house mouse	IGKV3-5
L3-9,10-A	CDRL3	3ZKM	L	QQNNEDPWT	house mouse	IGKV3-10
L3-9,10-A	CDRL3	1JHL	L	QQHNEYPWT	house mouse	IGKV16-104
L3-9,10-A	CDRL3	1CLZ	L	FQGSHVPFT	house mouse	IGKV1-117
L3-9,10-A	CDRL3	3HR5	L	SQNTLVPWT	house mouse	IGKV
L3-9,10-A	CDRL3	1P7K	A	QHHYGTPLT	house mouse	IGKV12-44
L3-9,10-A	CDRL3	1TZG	M	QQYGQSLST	human	IGKV3-20
L3-9,10-A	CDRL3	1ND0	G	WQGTHFPYT	house mouse	IGKV1-133
L3-9,10-A	CDRL3	1YY8	A	QQNNNWPTT	house mouse	IGKV5-48
L3-9,10-A	CDRL3	3FO2	L	FQGSHLPPT	house mouse	IGKV1-117
L3-9,10-A	CDRL3	3NIG	L	VQYAQLPYT	house mouse	IGKV14-100
L3-9,10-A	CDRL3	2OR9	M	QQTKEVPWT	house mouse	IGKV3-2
L3-9,10-A	CDRL3	3QG6	A	SQTTHVPYT	house mouse	IGKV1-110
L3-9,10-A	CDRL3	4ALA	L	LQHWTYPYT	house mouse	IGKV6-14
L3-9,10-A	CDRL3	4H88	L	QQSNTWPYT	house mouse	IGKV5-48
L3-9,10-A	CDRL3	3GI9	L	QQHYSTPYT	house mouse	IGKV8-24
L3-9,10-A	CDRL3	1WT5	C	FQGSHIPPT	house mouse	IGKV
L3-9,10-A	CDRL3	2O5Z	L	FQSTHFFPT	house mouse	IGKV1-110
L3-9,10-A	CDRL3	3O0R	L	QQHNEYPLT	house mouse	IGKV16-104
L3-9,10-A	CDRL3	2BX5	O	QQSYSTPNT	human	IGKV1-39
L3-9,10-A	CDRL3	2W0F	B	QQSNRWPFT	house mouse	IGKV5-48
L3-9,10-A	CDRL3	2ORB	L	QQTKEVPWT	house mouse	IGKV3-2
L3-9,10-A	CDRL3	2HRP	L	QQSKEVPWT	house mouse	IGKV3-2
L3-9,10-A	CDRL3	2GK0	L	FQGSHLPPT	house mouse	IGKV1-117
L3-9,10-A	CDRL3	2OSL	L	QQWTSNPPT	house mouse	IGKV4-72
L3-9,10-A	CDRL3	3B2U	R	HQYGSTPLT	human	IGKV3D-11
L3-9,10-A	CDRL3	4D9R	D	LQSDSLPYT	human	IGKV1-27
L3-9,10-A	CDRL3	2ADI	A	LQSDTLPLT	house mouse	IGKV17-121
L3-9,10-A	CDRL3	2PW2	A	QQLHFYPHT	human	IGKV1D-13
L3-9,10-A	CDRL3	2P8P	A	QQLHFYPHT	human	IGKV1D-13

L3-9,10-A	CDRL3	4I77	L	QQNNEDPRT	house mouse	IGKV3
L3-9,10-A	CDRL3	3LEX	L	WQGTHFPWT	house mouse	IGKV1-135
L3-9,10-A	CDRL3	4NRX	L	QQSYSTPFT	human	IGKV1-39
L3-9,10-A	CDRL3	2FX9	L	QQYGQSLST	human	IGKV3-20
L3-9,10-A	CDRL3	3RVV	C	LQYDEFPYT	house mouse	IGKV14-111
L3-9,10-A	CDRL3	3EYO	A	QQRSMWPPVT	human	IGKV3-11
L3-9,10-A	CDRL3	1KFA	L	SQSTHVPFT	house mouse	IGKV1-110
L3-9,10-A	CDRL3	3K2U	L	QQSNRAPAT	human	IGKV1D-13
L3-9,10-A	CDRL3	1FE8	L	QNGGTNPWT	house mouse	IGKV10-96
L3-9,10-A	CDRL3	2D7T	L	LQTHAYPLT	human	IGKV1-6
L3-9,10-A	CDRL3	3HC4	L	QQYDTYPFT	human	IGKV1-16
L3-9,10-A	CDRL3	1UWG	L	QQYNLFPVT	house mouse	IGKV6-15
L3-9,10-A	CDRL3	3I9G	L	LQSDNLPFT	house mouse	IGKV17-121
L3-9,10-A	CDRL3	3CLE	L	QQYSSFPLT	house mouse	IGKV6-15
L3-9,10-A	CDRL3	3HNV	L	HQDTKPPYT	house mouse	IGKV10-96
L3-9,10-A	CDRL3	43C9	C	QQHYRAPRT	house mouse	IGKV8-24
L3-9,10-A	CDRL3	1A3L	L	AQNLELPYT	house mouse	IGKV2-109
L3-9,10-A	CDRL3	1MJJ	L	LQHLEYPFT	house mouse	IGKV2-137
L3-9,10-A	CDRL3	3L95	L	QQFYTTPST	human	IGKV1-39
L3-9,10-A	CDRL3	1EGJ	L	QQNNEDPWT	house mouse	IGKV3-10
L3-9,10-A	CDRL3	3SGE	L	WQGSHFPYT	house mouse	IGKV1-135
L3-9,10-A	CDRL3	1CGS	L	SQGTHVPYT	house mouse	IGKV1-110
L3-9,10-A	CDRL3	1NBV	L	SQSTHVPLT	house mouse	IGKV1-110
L3-9,10-A	CDRL3	1QP1	C	QQYDDLPYT	human	IGKV1-33
L3-9,10-A	CDRL3	3KYM	A	QQYDKWPLT	human	IGKV3-15
L3-9,10-A	CDRL3	3IDI	A	QQLHFYPHT	human	IGKV1-13
L3-9,10-A	CDRL3	1HH9	A	LQYDDFPLT	house mouse	IGKV14-111
L3-9,10-A	CDRL3	1HH6	A	LQYDDFPLT	house mouse	IGKV14-111
L3-9,10-A	CDRL3	3PP3	L	AQNLELPYT	house mouse	IGKV
L3-9,10-A	CDRL3	2IQA	A	FQGSHVPLT	house mouse	IGKV1-117
L3-9,10-A	CDRL3	1CR9	L	WQGTHFPHT	house mouse	IGKV1-135
L3-9,10-A	CDRL3	1DBJ	L	SQSSHVPPT	house mouse	IGKV1-110
L3-9,10-A	CDRL3	1JFQ	L	QQGNALPRT	house mouse	IGKV10-96
L3-9,10-A	CDRL3	1BZ7	A	QQGKTLPYT	house mouse	IGKV10-96
L3-9,10-A	CDRL3	1CZ8	L	QQYSTVPWT	human	IGKV1-16
L3-9,10-A	CDRL3	3GRW	L	QQSYTTPPT	human	IGKV1-39
L3-9,10-A	CDRL3	1RUK	L	WQGTHFPYT	house mouse	IGKV1-133
L3-9,10-A	CDRL3	1DBM	L	SQSSHVPPT	house mouse	IGKV1-110
L3-9,10-A	CDRL3	1DEE	C	QQSYSAPRT	human	IGKV1-39
L3-9,10-A	CDRL3	4HIX	L	WQGTHFPRT	house mouse	IGKV
L3-9,10-A	CDRL3	1WCB	L	QQRSTYPFT	house mouse	IGKV4-57
L3-9,10-A	CDRL3	2UYL	M	FQGSHVPRT	house mouse	IGKV1-117
L3-9,10-A	CDRL3	2IPU	K	FQGSHVPLT	house mouse	IGKV1-117
L3-9,10-A	CDRL3	1LK3	L	QQSWNGPLT	Norway rat	IGKV3S11
L3-9,10-A	CDRL3	4NCC	1	QQRSSYPFT	house mouse	IGKV4-57
L3-9,10-A	CDRL3	3Q3G	C	QQYYSYPLT	house mouse	IGKV8-30
L3-9,10-A	CDRL3	2VDL	L	VQYAQLPYT	house mouse	IGKV14-100
L3-9,10-A	CDRL3	1NGY	A	QQYSSYPLT	house mouse	IGKV6-13
L3-9,10-A	CDRL3	1HIL	C	QNDYSNPLT	house mouse	IGKV8-19
L3-9,10-A	CDRL3	2CJU	L	FQGSHVPYT	house mouse	IGKV1-117

L3-9,10-A	CDRL3	2Y6S	L	QHIAELTRT	house mouse	IGKV3-12
L3-9,10-A	CDRL3	2YSS	A	QQSNSWPYT	human	IGKV3-15
L3-9,10-A	CDRL3	4D9Q	D	LQSDSLPYT	human	IGKV1-27
L3-9,10-A	CDRL3	4JN2	A	LQSTHFPHT	house mouse	IGKV1-133
L3-9,10-A	CDRL3	1YEF	L	VQGTHFPYT	house mouse	IGKV1-133
L3-9,10-A	CDRL3	1YEI	L	VQGTHFPYT	house mouse	IGKV1-133
L3-9,10-A	CDRL3	1AXS	A	LQYDEFPYT	house mouse	IGKV14-111
L3-9,10-A	CDRL3	3MNV	C	LQHYNTPWT	house mouse	IGKV8-24
L3-9,10-A	CDRL3	4LAR	L	HQWSSFPFT	house mouse	IGKV4-79
L3-9,10-A	CDRL3	2Y5T	B	QQSNEDPYT	house mouse	IGKV3-5
L3-9,10-A	CDRL3	1AD0	A	QHWSSKPPT	human	IGKV1-16
L3-9,10-A	CDRL3	1XF4	L	QHHYGTPLT	house mouse	IGKV12-44
L3-9,10-A	CDRL3	3DIF	C	QQHYSTPWT	house mouse	IGKV6-17
L3-9,10-A	CDRL3	2FJF	S	QQSYTTPPT	human	IGKV1-39
L3-9,10-A	CDRL3	2AJ3	C	QHLKRYPYT	human	IGKV1-9
L3-9,10-A	CDRL3	1BLN	C	FQASHAPRT	house mouse	IGKV1-117
L3-9,10-A	CDRL3	2FX7	L	QQYGQSLST	human	IGKV3-20
L3-9,10-A	CDRL3	2VXU	L	LQYASSPYT	house mouse	IGKV9-120
L3-9,10-A	CDRL3	1MEX	L	QQYNSYPYT	house mouse	IGKV6-15
L3-9,10-A	CDRL3	1A5F	L	QNNYNYPLT	house mouse	IGKV8-19
L3-9,10-A	CDRL3	2V7N	C	HQYSDISPT	human	IGKV3-11
L3-9,10-A	CDRL3	3U1S	L	MQGLHSPWT	human	IGKV2-28
L3-9,10-A	CDRL3	4NCC	L	QQRSSYPFT	house mouse	IGKV4-57
L3-9,10-A	CDRL3	1ZTX	L	QQHYTTPLT	house mouse	IGKV6-25
L3-9,10-A	CDRL3	4D9Q	L	LQSDSLPYT	human	IGKV1-27
L3-9,10-A	CDRL3	1CFN	A	LQYDDFPLT	house mouse	IGKV14-111
L3-9,10-A	CDRL3	2A6J	A	QQGNTLPRT	house mouse	IGKV10-96
L3-9,10-A	CDRL3	1MRD	L	SQSTHVPRT	house mouse	IGKV1-110
L3-9,10-A	CDRL3	4CAD	D	QHFWSTPWT	house mouse	IGKV12-41
L3-9,10-A	CDRL3	3RKD	L	QHFWGNPWT	house mouse	IGKV12-46
L3-9,10-A	CDRL3	2Z4Q	A	FQGSHIPPT	house mouse	IGKV1-117
L3-9,10-A	CDRL3	1MAM	L	QQGNTLPFT	house mouse	IGKV10-96
L3-9,10-A	CDRL3	1CFT	A	LQYDDFPLT	house mouse	IGKV14-111
L3-9,10-A	CDRL3	2HFE	B	QQSNRWPFT	house mouse	IGKV5-48
L3-9,10-A	CDRL3	1FVE	A	QQHYTTPPT	human	IGKV1-39
L3-9,10-A	CDRL3	1NGZ	A	QQYSSYPLT	house mouse	IGKV6-13
L3-9,10-A	CDRL3	1U92	A	QQLHFYPHT	human	IGKV1D-13
L3-9,10-A	CDRL3	2IGF	L	FQGSHVPPT	house mouse	IGKV1-117
L3-9,10-A	CDRL3	1MHP	L	QQWSGNPWT	human	IGKV1D-13
L3-9,10-A	CDRL3	2IQ9	L	FQGSHVPLT	house mouse	IGKV1-117
L3-9,10-A	CDRL3	1QBM	L	QHFWSTPWT	house mouse	IGKV12-41
L3-9,10-A	CDRL3	3U0W	L	QQYRKLPYT	house mouse	IGKV10-94
L3-9,10-A	CDRL3	1UA6	L	QQSNSWPYT	house mouse	IGKV5-43
L3-9,10-A	CDRL3	1A3R	L	QNNYNYPLT	house mouse	IGKV8-19
L3-9,10-A	CDRL3	1U91	A	QQLHFYPHT	human	IGKV1D-13
L3-9,10-A	CDRL3	1NBY	A	QQSNSWPYT	house mouse	IGKV5-43
L3-9,10-A	CDRL3	4LKC	A	QQFNSYLIT	human	IGKV1-13
L3-9,10-A	CDRL3	1TZG	L	QQYGQSLST	human	IGKV3-20
L3-9,10-A	CDRL3	2C1O	L	VQGSHFPPT	house mouse	IGKV1-133
L3-9,10-A	CDRL3	3EO9	L	QQHNEYPLT	human	IGKV1-16

L3-9,10-A	CDRL3	3RA7	L	QQSITLPPT	house mouse	IGKV10-96
L3-9,10-A	CDRL3	1A6T	A	QQYSSHPLT	house mouse	IGKV4-72
L3-9,10-A	CDRL3	2VDQ	L	VQYAQLPYT	house mouse	IGKV14-100
L3-9,10-A	CDRL3	1NCC	L	QQHYSPPWT	house mouse	IGKV6-25
L3-9,10-A	CDRL3	1MRF	L	SQSTHVPRT	house mouse	IGKV1-110
L3-9,10-A	CDRL3	2Q76	A	QQSGSWPRT	house mouse	IGKV5-43
L3-9,10-A	CDRL3	3BZ4	C	AHNVELPRT	house mouse	IGKV2-109
L3-9,10-A	CDRL3	3TCL	B	QQYGRSPYT	human	IGKV3-20
L3-9,10-A	CDRL3	3V6O	F	LQHWNYPLT	house mouse	IGKV6-14
L3-9,10-A	CDRL3	1GPO	M	QQITDWPFT	house mouse	IGKV5-43
L3-9,10-A	CDRL3	2EKS	A	QQSNSWPYT	human	IGKV3-15
L3-9,10-A	CDRL3	1IVL	B	QQVSEWPFT	house mouse	IGKV5-43
L3-9,10-A	CDRL3	4KVC	L	GQGYSYPYT	house mouse	IGKV6-20
L3-9,10-A	CDRL3	1CE1	L	LQHISRPRT	human	IGKV1-33
L3-9,10-A	CDRL3	1BRE	A	QQYDDLPYT	human	IGKV1-33
L3-9,10-A	CDRL3	4AEH	L	QHFWNIPFT	house mouse	IGKV12-46
L3-9,10-A	CDRL3	1GGC	L	QQSNEDPLT	house mouse	IGKV3-5
L3-9,10-A	CDRL3	1MF2	L	QQSKEVPWT	house mouse	IGKV3-2
L3-9,10-A	CDRL3	4KUC	D	QHKREYPPT	house mouse	IGKV3-12
L3-9,10-A	CDRL3	1EFQ	A	QQYYSTPYS	human	IGKV4-1
L3-9,10-A	CDRL3	1ND0	C	WQGTHFPYT	house mouse	IGKV1-133
L3-9,10-A	CDRL3	1VFA	A	QHFWSTPRT	house mouse	IGKV12-41
L3-9,10-A	CDRL3	2AI0	N	FQGSHIPWT	house mouse	IGKV1-117
L3-9,10-A	CDRL3	3QPQ	I	QHFWSTPFT	house mouse	IGKV12-41
L3-9,10-A	CDRL3	2FJG	L	QQSYTTPPT	human	IGKV1-39
L3-9,10-A	CDRL3	1CF8	L	LQWSSFPYT	house mouse	IGKV4-79
L3-9,10-A	CDRL3	3T3P	L	VQYAQLPYT	house mouse	IGKV14-100
L3-9,10-A	CDRL3	3CDF	C	QQYDNLPYT	human	IGKV1-33
L3-9,10-A	CDRL3	4NRZ	L	QQSYSTPFT	human	IGKV1-39
L3-9,10-A	CDRL3	3C6S	A	AHNVELPRT	house mouse	IGKV2-109
L3-9,10-A	CDRL3	4K23	L	WQGTHFPLT	house mouse	IGKV1-135
L3-9,10-A	CDRL3	4IOI	A	QQHYTTPPT	human	IGKV1-39
L3-9,10-A	CDRL3	1NGW	L	QQYSSYPLT	house mouse	IGKV6-13
L3-9,10-A	CDRL3	3QNX	A	MQNKQTPLT	human	IGKV2-28
L3-9,10-A	CDRL3	1ZA6	G	QQYYSYPLT	house mouse	IGKV
L3-9,10-A	CDRL3	1N7M	H	QQYSSYPLT	house mouse	IGKV6-13
L3-9,10-A	CDRL3	2HMI	C	QQYSKFPWT	house mouse	IGKV10-94
L3-9,10-A	CDRL3	3UPA	B	QQSYSTPNT	human	IGKV1-39
L3-9,10-A	CDRL3	1IGT	C	QQGQSYPLT	house mouse	IGKV10-94
L3-9,10-A	CDRL3	1A4J	A	SQSTHVPPT	house mouse	IGKV1-110
L3-9,10-A	CDRL3	4NZR	L	QQYEEWPRT	human	IGKV3-15
L3-9,10-A	CDRL3	3DIF	A	QQHYSTPWT	house mouse	IGKV6-17
L3-9,10-A	CDRL3	4LCU	B	QQSNRWPFT	house mouse	IGKV5-48
L3-9,10-A	CDRL3	1N4X	L	FQGSHFPPT	house mouse	IGKV1-117
L3-9,10-A	CDRL3	2Q1E	B	QQYDNLPYT	human	IGKV1-33
L3-9,10-A	CDRL3	1KCS	L	GQSYSSPLT	house mouse	IGKV6-20
L3-9,10-A	CDRL3	4KI5	D	QHWSSYPLT	house mouse	IGKV4-55
L3-9,10-A	CDRL3	4HS8	L	HQSKEAPYA	house mouse	IGKV3
L3-9,10-A	CDRL3	3G5Z	A	VQYAQFPWT	house mouse	IGKV14-100
L3-9,10-A	CDRL3	3FB5	B	QQSNRWPFT	house mouse	IGKV5-48

L3-9,10-A	CDRL3	1WC7	A	QQRSTYPFT	house mouse	IGKV4-57
L3-9,10-A	CDRL3	2BX5	M	QQSYSTPNT	human	IGKV1-39
L3-9,10-A	CDRL3	1DQJ	A	QQSNSWPYT	house mouse	IGKV5-43
L3-9,10-A	CDRL3	2RCS	L	LQYASYPRT	house mouse	IGKV9-124
L3-9,10-A	CDRL3	3B2U	L	HQYGSTPLT	human	IGKV3D-11
L3-9,10-A	CDRL3	2GCY	A	QQSNEAPFT	house mouse	IGKV3-5
L3-9,10-A	CDRL3	1EEQ	A	QQYYSHPYS	human	IGKV4-1
L3-9,10-A	CDRL3	1BRE	E	QQYDDLPYT	human	IGKV1-33
L3-9,10-A	CDRL3	3BN9	E	QQHGNLPYT	human	IGKV1-12
L3-9,10-A	CDRL3	4F3F	A	QQWSKHPLT	house mouse	IGKV4-59
L3-9,10-A	CDRL3	1U95	A	QQLHFYPHT	human	IGKV1D-13
L3-9,10-A	CDRL3	3MNZ	A	LQHYNTPWT	house mouse	IGKV8-24
L3-9,10-A	CDRL3	1NAK	L	FQTTHDPYT	house mouse	IGKV1-110
L3-9,10-A	CDRL3	3C6S	E	AHNVELPRT	house mouse	IGKV2-109
L3-9,10-A	CDRL3	1UZ6	M	AQNLEVPWT	house mouse	IGKV2-109
L3-9,10-A	CDRL3	3QOS	L	QQYYSTPLT	human	IGKV4-1
L3-9,10-A	CDRL3	1RIU	L	MQHLEYPFT	house mouse	IGKV2-137
L3-9,10-A	CDRL3	4AG4	L	QQWSSNPYT	house mouse	IGKV4-68
L3-9,10-A	CDRL3	1REI	B	QQYQSLPYT	human	IGKV1-33
L3-9,10-A	CDRL3	3CFD	A	QQGTTLPPT	house mouse	IGKV10-96
L3-9,10-A	CDRL3	1B0W	B	QQYDDLPYT	human	IGKV1-33
L3-9,10-A	CDRL3	3S34	L	QQAKAFPPT	human	IGKV1-12
L3-9,10-A	CDRL3	2R0Z	L	FQGSHVPLT	house mouse	IGKV1-117
L3-9,10-A	CDRL3	2FX8	M	QQYGQSLST	human	IGKV3-20
L3-9,10-A	CDRL3	4GAJ	L	QQNNVDPWT	house mouse	IGKV3-10
L3-9,10-A	CDRL3	4DGI	L	QQSNTWPYT	house mouse	IGKV5-48
L3-9,10-A	CDRL3	1HQ4	A	LQWSSFPYT	house mouse	IGKV4-79
L3-9,10-A	CDRL3	3BKM	L	FQASLVPLT	house mouse	IGKV1-117
L3-9,10-A	CDRL3	4F37	L	FQGSRVPLT	house mouse	IGKV1-117
L3-9,10-A	CDRL3	1AD9	L	MQHLEYPFT	house mouse	IGKV
L3-9,10-A	CDRL3	3B2U	X	HQYGSTPLT	human	IGKV3D-11
L3-9,10-A	CDRL3	2BMK	A	QQRSTYPFT	house mouse	IGKV4-57
L3-9,10-A	CDRL3	3GKW	L	FQYSHVPWT	house mouse	IGKV
L3-9,10-A	CDRL3	3CDF	A	QQYDNLPYT	human	IGKV1-33
L3-9,10-A	CDRL3	1FVE	C	QQHYTTPPT	human	IGKV1-39
L3-9,10-A	CDRL3	3LVE	A	QQYYSTPYS	human	IGKV4-1
L3-9,10-A	CDRL3	1F8T	L	VQGTHFPRT	house mouse	IGKV1-133
L3-9,10-A	CDRL3	4HJG	A	QQHYTTPPT	human	IGKV1-39
L3-9,10-A	CDRL3	2FJF	O	QQSYTTPPT	human	IGKV1-39
L3-9,10-A	CDRL3	1ZWI	B	QQSNRWPFT	house mouse	IGKV5-48
L3-9,10-A	CDRL3	3O2V	L	SQSTHFFPT	house mouse	IGKV1-110
L3-9,10-A	CDRL3	3QWO	B	FQGSGYPFT	human	IGKV1-5
L3-9,10-A	CDRL3	1NBZ	A	QQSNSWPYT	house mouse	IGKV5-43
L3-9,10-A	CDRL3	1U8N	A	QQLHFYPHT	human	IGKV1D-13
L3-9,10-A	CDRL3	3NZH	L	QQDYSSPYT	house mouse	IGKV6-32
L3-9,10-A	CDRL3	2DQJ	L	QQSNSWPYT	house mouse	IGKV5-43
L3-9,10-A	CDRL3	1IKF	L	QQGSRIPPT	house mouse	IGKV10-96
L3-9,10-A	CDRL3	2BDN	L	QQFWSAPYT	house mouse	IGKV13-84
L3-9,10-A	CDRL3	4LLU	B	QQYYIYPYT	human	IGKV1-16
L3-9,10-A	CDRL3	4G6M	L	LQGKMLPWT	house mouse	IGKV10-96

L3-9,10-A	CDRL3	3CVI	L	QQYWSTPLT	house mouse	IGKV13-84
L3-9,10-A	CDRL3	3KYM	I	QQYDKWPLT	human	IGKV3-15
L3-9,10-A	CDRL3	3ZE2	F	VQYAQLPYT	house mouse	IGKV14-100
L3-9,10-A	CDRL3	1ZEA	L	FQGSHIPFT	house mouse	IGKV1-117
L3-9,10-A	CDRL3	43CA	C	QQHYRAPRT	house mouse	IGKV8-24
L3-9,10-A	CDRL3	1UWX	K	QQLSSTPYT	house mouse	IGKV12-98
L3-9,10-A	CDRL3	3IGA	B	QQSNRWPFT	house mouse	IGKV5-48
L3-9,10-A	CDRL3	1K4C	B	QQSNRWPFT	house mouse	IGKV5-48
L3-9,10-A	CDRL3	3QQ9	L	QQIIEDPWT	house mouse	IGKV3-3
L3-9,10-A	CDRL3	1A4K	A	SQVTHVPPT	house mouse	IGKV1-110
L3-9,10-A	CDRL3	3QWO	L	FQGSGYPFT	human	IGKV1-5
L3-9,10-A	CDRL3	2DQE	L	QQSNSWPYT	house mouse	IGKV5-43
L3-9,10-A	CDRL3	3G5V	A	VQYAQFPWT	house mouse	IGKV14-100
L3-9,10-A	CDRL3	2YC1	B	QQYRYSPRT	human	IGKV3D-11
L3-9,10-A	CDRL3	1L7T	L	FDGSTVPPK	house mouse	IGKV1-117
L3-9,10-A	CDRL3	1RFD	L	MQHLEYPFT	house mouse	IGKV2-137
L3-9,10-A	CDRL3	4LVE	A	QQYYSTPYS	human	IGKV4-1
L3-9,10-A	CDRL3	2H9G	L	QQSYTTPPT	human	IGKV1-39
L3-9,10-A	CDRL3	4HH9	C	QQRGNWPPIT	human	IGKV3-11
L3-9,10-A	CDRL3	1WC7	L	QQRSTYPFT	house mouse	IGKV4-57
L3-9,10-A	CDRL3	2DQD	L	QQSNSWPYT	house mouse	IGKV5-43
L3-9,10-A	CDRL3	1BVK	D	QHFWSTPRT	human	IGKV1-27
L3-9,10-A	CDRL3	2XKN	A	QQGNTLPWT	house mouse	IGKV10-96
L3-9,10-A	CDRL3	3HNT	L	HQDTKPPYT	house mouse	IGKV10-96
L3-9,10-A	CDRL3	3QOT	L	QQYYSTPLT	human	IGKV4-1
L3-9,10-A	CDRL3	3T3P	F	VQYAQLPYT	house mouse	IGKV14-100
L3-9,10-A	CDRL3	1UWE	U	QQYNIYPVT	house mouse	IGKV6-15
L3-9,10-A	CDRL3	3FO9	A	SQGTHVPYT	house mouse	IGKV1-110
L3-9,10-A	CDRL3	1IGC	L	GQGNSYPYT	house mouse	IGKV6-20
L3-9,10-A	CDRL3	1KCV	L	GQSYSSPLT	house mouse	IGKV6-20
L3-9,10-A	CDRL3	2F58	L	QQSHEDPLT	house mouse	IGKV3-4
L3-9,10-A	CDRL3	1YNK	L	SQGTHVPYT	house mouse	IGKV1-110
L3-9,10-A	CDRL3	3A67	L	QQSNSWPYT	house mouse	IGKV5-43
L3-9,10-A	CDRL3	1CT8	C	HQTHGRPLT	house mouse	IGKV5-43
L3-9,10-A	CDRL3	4I2X	A	QQHYSTPWT	human	IGKV6-17
L3-9,10-A	CDRL3	2LVE	A	QQYYSTPYS	human	IGKV4-1
L3-9,10-A	CDRL3	1BWW	B	QQYQSLPYT	human	IGKV1-33
L3-9,10-A	CDRL3	1AJ7	L	LQYASYPRT	house mouse	IGKV9-124
L3-9,10-A	CDRL3	3IFO	L	FQGSHVPLT	house mouse	IGKV1-117
L3-9,10-A	CDRL3	4JFZ	L	LQSFNVPLT	human	IGKV3-11
L3-9-A	CDRL3	1Y18	C	ALWNSNHLV	house mouse	IGLV1
L3-9-A	CDRL3	3CFK	O	ALWNSNHLV	house mouse	IGLV1
L3-9-A	CDRL3	4AT6	B	ALWYSNHLV	house mouse	IGLV1
L3-9-A	CDRL3	1A6W	L	ALWYSNHWV	house mouse	IGLV1
L3-9-A	CDRL3	2FBJ	L	QQWTYPLIT	house mouse	IGKV4-86
L3-9-A	CDRL3	1Y18	A	ALWNSNHLV	house mouse	IGLV1
L3-9-A	CDRL3	3GJE	L	WSFAGSYYV	human	IGLV2-11
L3-9-A	CDRL3	2Y36	L	ALWYSNHWV	house mouse	IGLV1
L3-9-A	CDRL3	2CD0	A	QSYDHNNQV	human	IGLV6-57
L3-9-A	CDRL3	2CD0	B	QSYDHNNQV	human	IGLV6-57

L3-9-A	CDRL3	3T2N	M	ALWYSNHFI	human	IGLV7-43
L3-9-A	CDRL3	2ZPK	L	ALWYSNHWV	house mouse	IGLV1
L3-9-A	CDRL3	3CFJ	A	ALWNSNHLV	house mouse	IGLV1
L3-9-A	CDRL3	1OAX	N	ALWYSNHLV	house mouse	IGLV1
L3-9-A	CDRL3	2Y06	L	ALWYSNHWV	house mouse	IGLV1
L3-9-A	CDRL3	1OAY	L	ALWYSNHLV	house mouse	IGLV1
L3-9-A	CDRL3	1OAZ	L	ALWYSNHLV	house mouse	IGLV1
L3-9-A	CDRL3	1PW3	B	QSYDARNVV	human	IGLV6-57
L3-9-A	CDRL3	4AMK	L	ALWYSNHLV	house mouse	IGLV1
L3-9-A	CDRL3	2XZQ	L	ALWYSNHWV	house mouse	IGLV1
L3-9-A	CDRL3	3CFJ	C	ALWNSNHLV	house mouse	IGLV1
L3-9-A	CDRL3	1OAY	N	ALWYSNHLV	house mouse	IGLV1
L3-9-A	CDRL3	1A6V	N	ALWYSNHWV	house mouse	IGLV1
L3-9-A	CDRL3	1MFD	L	ALWCNNHWI	house mouse	IGLV1
L3-9-A	CDRL3	1INE	L	ALWYSNLWV	house mouse	IGLV1
L3-9-A	CDRL3	1Q0Y	L	ALWSNNKLV	house mouse	IGLV1
L3-9-A	CDRL3	4AT6	N	ALWYSNHLV	house mouse	IGLV1
L3-9-A	CDRL3	4KTE	L	AIWHNFACV	rhesus monkey	IGLV
L3-9-A	CDRL3	4JZO	E	QAWDSNTGV	human	IGLV3-1
L3-9-A	CDRL3	1Y0L	C	ALWNSNHLV	house mouse	IGLV1
L3-9-A	CDRL3	1OAR	O	ALWYSNHLV	house mouse	IGLV1
L3-9-A	CDRL3	1ETZ	A	ALWYSNHWV	house mouse	IGLV1
L3-9-A	CDRL3	1NC2	C	ALWYSNHWV	house mouse	IGLV1
L3-9-A	CDRL3	1OAU	N	ALWYSNHLV	house mouse	IGLV1
L3-9-A	CDRL3	4JZO	H	QAWDSNTGV	human	IGLV3-1
L3-9-A	CDRL3	2Y07	L	ALWYSNHWV	house mouse	IGLV1
L3-9-A	CDRL3	3KS0	J	ALWDSNHLV	house mouse	IGLV1
L3-9-A	CDRL3	1GIG	L	ALWYSNHWV	house mouse	IGLV1
L3-9-A	CDRL3	1OAX	L	ALWYSNHLV	house mouse	IGLV1
L3-9-A	CDRL3	1Y0L	L	ALWNSNHLV	house mouse	IGLV1
L3-9-A	CDRL3	4JZO	F	QAWDSNTGV	human	IGLV3-1
L3-9-A	CDRL3	1ETZ	L	ALWYSNHWV	house mouse	IGLV1
L3-9-A	CDRL3	1Q0X	L	ALWSNNKLV	house mouse	IGLV1
L3-9-A	CDRL3	1A6V	L	ALWYSNHWV	house mouse	IGLV1
L3-9-A	CDRL3	7FAB	L	QSYDRSLRV	human	IGLV1-40
L3-9-A	CDRL3	1JN6	A	ALWFSNQFI	house mouse	IGLV1
L3-9-A	CDRL3	4JZN	B	QVWDSSSVV	human	IGLV3-21
L3-9-A	CDRL3	3CFJ	E	ALWNSNHLV	house mouse	IGLV1
L3-9-A	CDRL3	3GJF	K	WSFAGSYYV	human	IGLV2-11
L3-9-A	CDRL3	3CFK	C	ALWNSNHLV	house mouse	IGLV1
L3-9-A	CDRL3	1OAZ	N	ALWYSNHLV	house mouse	IGLV1
L3-9-A	CDRL3	1OAU	L	ALWYSNHLV	house mouse	IGLV1
L3-9-A	CDRL3	1NGP	L	ALWYSNHWV	house mouse	IGLV1
L3-9-A	CDRL3	3CFK	A	ALWNSNHLV	house mouse	IGLV1
L3-9-A	CDRL3	1Y18	E	ALWNSNHLV	house mouse	IGLV1
L3-9-A	CDRL3	4AT6	D	ALWYSNHLV	house mouse	IGLV1
L3-9-A	CDRL3	3KS0	L	ALWDSNHLV	house mouse	IGLV1
L3-9-A	CDRL3	4KTD	L	AIWHSGAWV	rhesus monkey	IGLV
L3-9-A	CDRL3	1Y0L	E	ALWNSNHLV	house mouse	IGLV1
L3-9-A	CDRL3	1NGQ	L	ALWYSNHWV	house mouse	IGLV1

L3-9-A	CDRL3	3CFK	G	ALWNSNHLV	house mouse	IGLV1
L3-9-A	CDRL3	1NJ9	L	ALWYSNHWV	house mouse	IGLV1
L3-9-A	CDRL3	4JZN	P	QVWDSSSVV	human	IGLV3-21
L3-9-A	CDRL3	3GJE	A	WSFAGSYYV	human	IGLV2-11
L3-9-A	CDRL3	1OAQ	L	ALWYSNHLV	house mouse	IGLV1
L3-9-A	CDRL3	4JZN	D	QVWDSSSVV	human	IGLV3-21
L3-9-A	CDRL3	1OAX	M	ALWYSNHLV	house mouse	IGLV1
L3-9-A	CDRL3	3CFK	J	ALWNSNHLV	house mouse	IGLV1
L3-9-A	CDRL3	1MFC	L	ALWCNNHWI	house mouse	IGLV1
L3-9-A	CDRL3	1PG7	W	ALWYSNHWV	house mouse	IGLV1
L3-9-A	CDRL3	3NPS	C	QSRDISQYV	human	IGLV1-44
L3-9-A	CDRL3	1OAR	N	ALWYSNHLV	house mouse	IGLV1
L3-9-A	CDRL3	3GJF	L	WSFAGSYYV	human	IGLV2-11
L3-9-A	CDRL3	1OAR	L	ALWYSNHLV	house mouse	IGLV1
L3-9-A	CDRL3	1NJ9	A	ALWYSNHWV	house mouse	IGLV1
L3-9-A	CDRL3	1MFB	L	ALWCNNHWI	house mouse	IGLV1
L3-9-A	CDRL3	3CFK	L	ALWNSNHLV	house mouse	IGLV1
L3-9-A	CDRL3	1SM3	L	ALWYSNHWV	house mouse	IGLV1
L3-9-A	CDRL3	1F4X	L	ALWYSGHWV	house mouse	IGLV1
L3-9-A	CDRL3	1MFE	L	ALWCNNHWI	house mouse	IGLV1
L3-9-A	CDRL3	4AT6	F	ALWYSNHLV	house mouse	IGLV1
L3-9-A	CDRL3	3CFK	M	ALWNSNHLV	house mouse	IGLV1
L3-9-A	CDRL3	2ZPK	M	ALWYSNHWV	house mouse	IGLV1
L3-9-A	CDRL3	4AT6	K	ALWYSNHLV	house mouse	IGLV1
L3-9-A	CDRL3	1MFA	L	ALWSNNHWI	house mouse	IGLV1
L3-9-A	CDRL3	1IND	L	ALWYSNLWV	house mouse	IGLV1
L3-9-A	CDRL3	1CD0	B	QSYDARNVV	human	IGLV6-57
L3-9-A	CDRL3	1F4Y	L	ALWYSGHWV	house mouse	IGLV1
L3-9-A	CDRL3	1OAR	M	ALWYSNHLV	house mouse	IGLV1
L3-9-A	CDRL3	1NC2	A	ALWYSNHWV	house mouse	IGLV1
L3-9-A	CDRL3	4AT6	P	ALWYSNHLV	house mouse	IGLV1
L3-9-A	CDRL3	1PG7	Y	ALWYSNHWV	house mouse	IGLV1
L3-9-A	CDRL3	1CD0	A	QSYDARNVV	human	IGLV6-57
L3-9-A	CDRL3	1Y18	L	ALWNSNHLV	house mouse	IGLV1
L3-9-A	CDRL3	1PW3	A	QSYDARNVV	human	IGLV6-57
L3-9-A	CDRL3	3CFK	E	ALWNSNHLV	house mouse	IGLV1
L3-9-A	CDRL3	3CFJ	L	ALWNSNHLV	house mouse	IGLV1
L3-9-A	CDRL3	3T2N	L	ALWYSNHFI	human	IGLV7-43
L3-9-A	CDRL3	4HPO	L	QSYDNSSWV	human	IGLV6-57
L3-9-A	CDRL3	1F4W	L	ALWYSGHWV	house mouse	IGLV1
L3-9-A	CDRL3	1NC4	C	ALWYSNHWV	house mouse	IGLV1
L3-9-A	CDRL3	1A6V	M	ALWYSNHWV	house mouse	IGLV1
L3-9-A	CDRL3	1Y0L	A	ALWNSNHLV	house mouse	IGLV1
L3-9-A	CDRL3	4AT6	I	ALWYSNHLV	house mouse	IGLV1
L3-9-A	CDRL3	4JZO	B	QAWDSNTGV	human	IGLV3-1
L3-9-A	CDRL3	1NC4	A	ALWYSNHWV	house mouse	IGLV1
L3-9-A	CDRL3	1A6U	L	ALWYSNHWV	house mouse	IGLV1
L3-9-B	CDRL3	2VXS	N	QTYDPYSVV	human	IGLV6-57
L3-9-B	CDRL3	2VXS	M	QTYDPYSVV	human	IGLV6-57
L3-9-B	CDRL3	3H0T	A	QSYDSSNVV	human	IGLV6-57

L3-9-B	CDRL3	2VXS	L	QTYDPYSVV	human	IGLV6-57
L3-9-B	CDRL3	2VXS	O	QTYDPYSVV	human	IGLV6-57
L3-9-C	CDRL3	2HWZ	L	FQGSGYPFT	house mouse	IGKV1-117
L3-9-C	CDRL3	1UCB	L	FQGSHVPFT	house mouse	IGKV1-117
L3-10,11-A	CDRL3	1BJM	B	AAWDDSLDVAV	human	IGLV1-44
L3-10,11-A	CDRL3	4D9L	O	QSYDSSLSGYV	human	IGLV1-40
L3-10,11-A	CDRL3	3T0V	A	LSWDDSLNGWV	human	IGLV1-47
L3-10,11-A	CDRL3	3LRH	E	ATWDDSLNGWV	human	IGLV1-36
L3-10,11-A	CDRL3	4IMK	C	QVWDSSSDHYV	human	IGLV3-21
L3-10,11-A	CDRL3	1RZF	L	GTWDSSLSAVV	human	IGLV1-51
L3-10,11-A	CDRL3	3G04	A	TSWDDSLDSQL	human	IGLV1-36
L3-10,11-A	CDRL3	3LRH	C	ATWDDSLNGWV	human	IGLV1-36
L3-10,11-A	CDRL3	3BJL	B	AAWDDSLDVAV	human	IGLV1-44
L3-10,11-A	CDRL3	3LRH	A	ATWDDSLNGWV	human	IGLV1-36
L3-10,11-A	CDRL3	4FQH	L	AAWDDSLKGAV	human	IGLV1-44
L3-10,11-A	CDRL3	4FQI	L	AAWDDSLKGAV	human	IGLV1-44
L3-10,11-A	CDRL3	4IMK	D	QVWDSSSDHYV	human	IGLV3-21
L3-10,11-A	CDRL3	3LRH	K	ATWDDSLNGWV	human	IGLV1-36
L3-10,11-A	CDRL3	3T0X	A	LSWDDSLNGWV	human	IGLV1-47
L3-10,11-A	CDRL3	4NZT	L	AAWDDSLKGAV	human	IGLV1-44
L3-10,11-A	CDRL3	3LRH	O	ATWDDSLNGWV	human	IGLV1-36
L3-10,11-A	CDRL3	1NL0	L	AAWDDSLSEFL	human	IGLV1-51
L3-10,11-A	CDRL3	3BJL	A	AAWDDSLDVAV	human	IGLV1-44
L3-10,11-A	CDRL3	4D9L	M	QSYDSSLSGYV	human	IGLV1-40
L3-10,11-A	CDRL3	4M5Y	L	QVWDISTDQAV	human	IGLV3-21
L3-10,11-A	CDRL3	4FQH	B	AAWDDSLKGAV	human	IGLV1-44
L3-10,11-A	CDRL3	3LRH	M	ATWDDSLNGWV	human	IGLV1-36
L3-10,11-A	CDRL3	3T0X	B	LSWDDSLNGWV	human	IGLV1-47
L3-10,11-A	CDRL3	2XWT	B	GTWDSRLGIAV	human	IGLV1-51
L3-10,11-A	CDRL3	4FQJ	L	ATWDDSLDGWV	human	IGLV1-47
L3-10,11-A	CDRL3	3LRH	G	ATWDDSLNGWV	human	IGLV1-36
L3-10,11-A	CDRL3	2RHE	A	AAWNDSLDEPG	human	IGLV1-36
L3-10,11-A	CDRL3	4HK0	B	QVWDSSSDHVV	human	IGLV3-21
L3-10,11-A	CDRL3	2YKL	L	AAWDDSLSAWV	human	IGLV1-47
L3-10,11-A	CDRL3	4D9L	L	QSYDSSLSGYV	human	IGLV1-40
L3-10,11-A	CDRL3	2XQB	L	AWYDRELSEWV	human	IGLV1-47
L3-10,11-A	CDRL3	1W72	L	QVWDSRTDHWV	human	IGLV3-21
L3-10,11-A	CDRL3	3LRH	I	ATWDDSLNGWV	human	IGLV1-36
L3-10,11-A	CDRL3	3H42	L	QSYDSSLSGSV	human	IGLV1-40
L3-10,11-A	CDRL3	4HK0	D	QVWDSSSDHVV	human	IGLV3-21
L3-10,11-A	CDRL3	1W72	M	QVWDSRTDHWV	human	IGLV3-21
L3-10,11-A	CDRL3	3N9G	L	AAWDDSLNGPV	human	IGLV1-44
L3-10,11-A	CDRL3	3LRG	A	ATWDDSLNGWV	human	IGLV1-36
L3-10,11-A	CDRL3	3MLX	M	ATWDGSLRTV	human	IGLV1-51
L3-10,11-A	CDRL3	4BJL	B	AAWDDSLDVAV	human	IGLV1-44
L3-10,11-A	CDRL3	2YK1	L	AAWDDSLSAWV	human	IGLV1-47
L3-10,11-A	CDRL3	4D9L	N	QSYDSSLSGYV	human	IGLV1-40
L3-10,11-A	CDRL3	3MLX	L	ATWDGSLRTV	human	IGLV1-51
L3-10,11-A	CDRL3	4M5Y	M	QVWDISTDQAV	human	IGLV3-21
L3-10,11-A	CDRL3	3MLY	L	ATWDGSLRTV	human	IGLV1-51

L3-10,11-A	CDRL3	2FB4	L	AAWDVSLNAYV	human	IGLV1-44
L3-10,11-A	CDRL3	4HKX	B	QVWDSDSDHVV	human	IGLV3-21
L3-10,11-A	CDRL3	3T0W	A	LSWDDSLNGWV	human	IGLV1-47
L3-10,11-A	CDRL3	3MLY	M	ATWDGSLRTV	human	IGLV1-51
L3-10,11-A	CDRL3	3T0W	B	LSWDDSLNGWV	human	IGLV1-47
L3-10,11-A	CDRL3	3LRG	B	ATWDDSLNGWV	human	IGLV1-36
L3-10,11-A	CDRL3	3Q6G	L	QVWDSSSHHML	human	IGLV3-21
L3-10-A	CDRL3	4F58	L	AAYTVASTLL	human	IGLV2-14
L3-10-A	CDRL3	3U2S	L	KSLTSTRRRV	human	IGLV2-14
L3-10-A	CDRL3	4F57	L	AAYTVASTLL	human	IGLV2-14
L3-10-A	CDRL3	3MUG	I	SSLTDRSHRI	human	IGLV2-14
L3-10-A	CDRL3	4F58	M	AAYTVASTLL	human	IGLV2-14
L3-10-A	CDRL3	3LRS	B	SSLTDRSHRI	human	IGLV2-14
L3-10-A	CDRL3	2W0L	A	QSYDSSNHVV	human	IGLV6-57
L3-10-A	CDRL3	3U2S	B	KSLTSTRRRV	human	IGLV2-14
L3-10-A	CDRL3	3MUG	G	SSLTDRSHRI	human	IGLV2-14
L3-10-A	CDRL3	2W0L	D	QSYDSSNHVV	human	IGLV6-57
L3-10-A	CDRL3	3U4E	B	KSLTSTRRRV	human	IGLV2-14
L3-10-A	CDRL3	3B5G	B	QSYDSSNHVV	human	IGLV6-57
L3-10-A	CDRL3	4F58	O	AAYTVASTLL	human	IGLV2-14
L3-10-A	CDRL3	3MUG	A	SSLTDRSHRI	human	IGLV2-14
L3-10-A	CDRL3	4F58	N	AAYTVASTLL	human	IGLV2-14
L3-10-A	CDRL3	2XZC	L	GTWDSSLNPV	human	IGLV1-51
L3-10-A	CDRL3	3BDX	B	QSYDSSNHVV	human	IGLV6-57
L3-10-A	CDRL3	3LRS	L	SSLTDRSHRI	human	IGLV2-14
L3-10-A	CDRL3	2XZA	L	GTWDSSLNPV	human	IGLV1-51
L3-10-A	CDRL3	3BDX	A	QSYDSSNHVV	human	IGLV6-57
L3-10-A	CDRL3	3LRS	D	SSLTDRSHRI	human	IGLV2-14
L3-10-A	CDRL3	3B5G	A	QSYDSSNHVV	human	IGLV6-57
L3-10-A	CDRL3	4DQO	L	SSLTDRSHRI	human	IGLV2-14
L3-10-A	CDRL3	3BDX	C	QSYDSSNHVV	human	IGLV6-57
L3-10-A	CDRL3	2W0L	B	QSYDSSNHVV	human	IGLV6-57
L3-10-A	CDRL3	3LRS	F	SSLTDRSHRI	human	IGLV2-14
L3-10-A	CDRL3	2W0L	C	QSYDSSNHVV	human	IGLV6-57
L3-10-A	CDRL3	2W0K	A	QSYDSSNHVV	human	IGLV6-57
L3-10-A	CDRL3	2W0K	B	QSYDSSNHVV	human	IGLV6-57
L3-10-A	CDRL3	3U4E	L	KSLTSTRRRV	human	IGLV2-14
L3-10-A	CDRL3	3MUG	K	SSLTDRSHRI	human	IGLV2-14
L3-10-B	CDRL3	3U79	F	QQYHNLPPFT	human	IGKV1-33
L3-10-B	CDRL3	3U79	D	QQYHNLPPFT	human	IGKV1-33
L3-10-B	CDRL3	3U79	G	QQYHNLPPFT	human	IGKV1-33
L3-10-B	CDRL3	3U79	H	QQYHNLPPFT	human	IGKV1-33
L3-10-B	CDRL3	3U79	E	QQYHNLPPFT	human	IGKV1-33
L3-10-B	CDRL3	3U79	A	QQYHNLPPFT	human	IGKV1-33
L3-10-B	CDRL3	3U79	B	QQYHNLPPFT	human	IGKV1-33
L3-10-B	CDRL3	3DVI	A	QQYHNLPPYT	human	IGKV1-33
L3-10-B	CDRL3	3U79	C	QQYHNLPPFT	human	IGKV1-33
L3-10-C	CDRL3	2DD8	L	QVWDSSSDYV	human	IGLV3-21
L3-10-C	CDRL3	4K94	L	QQWAVHSLIT	human	IGKV1-39
L3-10-C	CDRL3	3G6A	A	GTWDMVTNNV	human	IGLV3-1

L3-10-C	CDRL3	3G6A	L	GTWDMVTNNV	human	IGLV3-1
L3-10-D	CDRL3	3ULS	A	SSYDDPNFQV	human	IGLV3-21
L3-10-D	CDRL3	4JAM	L	QVWDSFSTFV	human	IGLV3-1
L3-10-D	CDRL3	4JAM	B	QVWDSFSTFV	human	IGLV3-1
L3-11-A	CDRL3	3DVG	A	QQYSSYSSLFT	human	IGKV1-13
L3-11-A	CDRL3	3Q6G	M	QVWDSSSHHML	human	IGLV3-21
L3-11-A	CDRL3	3MA9	L	ASWDSMTVDGV	human	IGLV3-1
L3-12-A	CDRL3	3QHF	L	AAWDDSLNGFWV	human	IGLV1-44
L3-12-A	CDRL3	3QHZ	M	AAWDDSLNGFWV	human	IGLV1-44
L3-12-A	CDRL3	3QHZ	L	AAWDDSLNGFWV	human	IGLV1-44
L3-12-A	CDRL3	4GXV	M	AAWDDSLSAHVV	human	IGLV1-44
L3-12-B	CDRL3	4JY5	L	HIWDSRRPTNWV	human	IGLV3-21
L3-12-B	CDRL3	4FQC	L	HIWDSRVPTKWV	human	IGLV3-21
L3-13-A	CDRL3	2B1H	L	AAWDDSRGGPDWV	human	IGLV1-47
L3-13-A	CDRL3	3MLW	L	ASWDDSRGGPDYV	human	IGLV1-47
L3-13-A	CDRL3	2B0S	L	AAWDDSRGGPDWV	human	IGLV1-47
L3-13-A	CDRL3	2B1A	L	AAWDDSRGGPDWV	human	IGLV1-47
L3-13-A	CDRL3	3MLW	M	ASWDDSRGGPDYV	human	IGLV1-47
Unclustered	CDRL3	3MCL	L	QNWRSSPT	house mouse	IGKV4-59
Unclustered	CDRL3	3GHB	M	ATWDSGLSADWV	human	IGLV1-51
Unclustered	CDRL3	2VXV	L	QQYNNWPSIT	human	IGKV3-15
Unclustered	CDRL3	4BJL	A	AAWDDSLDVAV	human	IGLV1-44
Unclustered	CDRL3	1MCR	A	SSYEGSDNFV	human	IGLV2-8
Unclustered	CDRL3	1ORS	A	HQFHRSLT	house mouse	IGKV4-74
Unclustered	CDRL3	3OAZ	K	QHYAGYSAT	human	IGKV1-5
Unclustered	CDRL3	4GXV	L	AAWDDSLSAHVV	human	IGLV1-44
Unclustered	CDRL3	1MCQ	A	SSYEGSDNFV	human	IGLV2-8
Unclustered	CDRL3	2OQJ	A	QHYAGYSAT	human	IGKV1-5
Unclustered	CDRL3	3C2A	L	ATWDSGLSADWV	human	IGLV1-51
Unclustered	CDRL3	1EK3	A	QQYYSTPPT	human	IGKV4-1
Unclustered	CDRL3	2OQJ	J	QHYAGYSAT	human	IGKV1-5
Unclustered	CDRL3	1ZLU	K	QHYAGYSAT	human	IGKV1-5
Unclustered	CDRL3	3OAZ	L	QHYAGYSAT	human	IGKV1-5
Unclustered	CDRL3	4K3H	G	SLSDVDGYL	human	IGLV7-46
Unclustered	CDRL3	1Q1J	L	ATWDSGLSADWV	human	IGLV1-51
Unclustered	CDRL3	3PNW	S	QQHGPFYWLFT	human	IGKV1-39
Unclustered	CDRL3	3OAY	K	QHYAGYSAT	human	IGKV1-5
Unclustered	CDRL3	4HBC	L	QSTYENPTYVS	rabbit	IGKV1S3
Unclustered	CDRL3	1ZLV	K	QHYAGYSAT	human	IGKV1-5
Unclustered	CDRL3	2OQJ	D	QHYAGYSAT	human	IGKV1-5
Unclustered	CDRL3	2E27	L	LQYDNFTWT	house mouse	IGKV19-93
Unclustered	CDRL3	4AJ0	B	QAADSSTAVV	human	IGLV3-1
Unclustered	CDRL3	1MCN	B	SSYEGSDNFV	human	IGLV2-8
Unclustered	CDRL3	1MCJ	A	SSYEGSDNFV	human	IGLV2-8
Unclustered	CDRL3	2NY3	C	QQYNNWPPRYT	human	IGKV3-15
Unclustered	CDRL3	1MCL	A	SSYEGSDNFV	human	IGLV2-8
Unclustered	CDRL3	4DN4	L	HQYIQLHSFT	human	IGKV3-11
Unclustered	CDRL3	1MCL	B	SSYEGSDNFV	human	IGLV2-8
Unclustered	CDRL3	3TNN	B	SSYAGSTTFRV	human	IGLV2-23
Unclustered	CDRL3	1MCR	B	SSYEGSDNFV	human	IGLV2-8

Unclustered	CDRL3	1MCS	A	SSYEGSDNFV	human	IGLV2-8
Unclustered	CDRL3	1MCN	A	SSYEGSDNFV	human	IGLV2-8
Unclustered	CDRL3	1OP3	K	QHYAGYSAT	human	IGKV1-5
Unclustered	CDRL3	4JO3	L	QQGYSISDIDNS	rabbit	IGKV1S3
Unclustered	CDRL3	4HIJ	C	QQRDNWPPDAT	human	IGKV3-11
Unclustered	CDRL3	2NY1	C	QQYNNWPPRYT	human	IGKV3-15
Unclustered	CDRL3	2NY4	C	QQYNNWPPRYT	human	IGKV3-15
Unclustered	CDRL3	3UJJ	L	YSTNSGGTFFV	human	IGLV3-10
Unclustered	CDRL3	4AIZ	C	QSADSSGTYVV	human	IGLV3-25
Unclustered	CDRL3	4JO1	M	LGGYDCSSGDCAA	rabbit	IGKV1S5
Unclustered	CDRL3	1MCD	B	SSYEGSDNFV	human	IGLV2-8
Unclustered	CDRL3	2OTW	C	GVGDTIKEQFVYV	house mouse	IGLV3
Unclustered	CDRL3	4KPH	M	HQWSSYPT	house mouse	IGKV4-80
Unclustered	CDRL3	3V6F	L	HQYLSSYMYT	house mouse	IGKV8-27
Unclustered	CDRL3	8FAB	C	QAWDNSASI	human	IGLV3-25
Unclustered	CDRL3	4AIX	B	QAWDSSTAVV	human	IGLV3-1
Unclustered	CDRL3	3R1G	L	QQFPTYLPT	human	IGKV1-13
Unclustered	CDRL3	1MCE	B	SSYEGSDNFV	human	IGLV2-8
Unclustered	CDRL3	4K3E	M	ASAEDSSSNAV	sheep	IGLV1S8
Unclustered	CDRL3	3PNW	V	QQHGPFYWLFT	human	IGKV1-39
Unclustered	CDRL3	4JO4	M	QQGYSISDIDNS	rabbit	IGKV1S3
Unclustered	CDRL3	3KYM	O	QQYDKWPLT	human	IGKV3-15
Unclustered	CDRL3	1MCC	A	SSYEGSDNFV	human	IGLV2-8
Unclustered	CDRL3	2OLD	B	SSYASGSTPRI	human	IGLV2-14
Unclustered	CDRL3	1MCD	A	SSYEGSDNFV	human	IGLV2-8
Unclustered	CDRL3	1KEL	L	FQGSHVPRT	house mouse	IGKV1-117
Unclustered	CDRL3	4DVB	L	QHYSAYPRT	house mouse	IGKV4-57-1
Unclustered	CDRL3	4JJ5	A	GVGDTIKEQFVYV	house mouse	IGLV3
Unclustered	CDRL3	1OM3	L	QHYAGYSAT	human	IGKV1-5
Unclustered	CDRL3	1ZLU	L	QHYAGYSAT	human	IGKV1-5
Unclustered	CDRL3	1MCB	B	SSYEGSDNFV	human	IGLV2-8
Unclustered	CDRL3	4HII	A	QQRDNWPPDAT	human	IGKV3-11
Unclustered	CDRL3	1MCF	B	SSYEGSDNFV	human	IGLV2-8
Unclustered	CDRL3	4HFW	L	QKTLRTWT	human	IGKV1-6
Unclustered	CDRL3	3FFD	B	GVGDTIKEQFVYV	house mouse	IGLV3
Unclustered	CDRL3	3MCG	1	SSYEGSDNFV	human	IGLV2-8
Unclustered	CDRL3	4KPH	L	HQWSSYPT	house mouse	IGKV4-80
Unclustered	CDRL3	1DCL	A	SSYEGSDNFV	human	IGLV2-8
Unclustered	CDRL3	3TNN	D	SSYAGSTTFRV	human	IGLV2-23
Unclustered	CDRL3	3PNW	D	QQHGPFYWLFT	human	IGKV1-39
Unclustered	CDRL3	1LHZ	A	SSYDGSSTSVV	human	IGLV2-23
Unclustered	CDRL3	3EHB	D	QHHYGTPPLT	house mouse	IGKV12-44
Unclustered	CDRL3	1NFD	G	LSSYGDNNDLV	hamster	IGLV
Unclustered	CDRL3	1HZH	M	QVYGASSYT	human	IGKV3-20
Unclustered	CDRL3	4JO2	M	LGGYDCSSGDCAA	rabbit	IGKV1S5
Unclustered	CDRL3	4K3H	B	SLSDVDGYL	human	IGLV7-46
Unclustered	CDRL3	1LIL	B	QVWDSNASVV	human	IGLV3-1
Unclustered	CDRL3	2OTU	A	GVGDTIKEQFVYV	house mouse	IGLV3
Unclustered	CDRL3	1GC1	L	QQYNNWPPRYT	human	IGKV3-15
Unclustered	CDRL3	4JO2	L	LGGYDCSSGDCAA	rabbit	IGKV1S5

Unclustered	CDRL3	1AQK	L	QSYDSSLSAR	human	IGLV1-40
Unclustered	CDRL3	4M1D	M	ATWDSGLSADWV	human	IGLV1-51
Unclustered	CDRL3	1BJM	A	AAWDDSLDVAV	human	IGLV1-44
Unclustered	CDRL3	3BJ9	1	AMGARSTHV	human	VPREB1
Unclustered	CDRL3	1YYM	L	QQYNNWPPRYT	human	IGKV3-15
Unclustered	CDRL3	3GHE	L	SSYTSSTPYVL	human	IGLV2-14
Unclustered	CDRL3	3NH7	O	STFTMSGNGTV	human	IGLV3-1
Unclustered	CDRL3	4HJJ	L	QQYNNWPSIT	human	IGKV4-1
Unclustered	CDRL3	3PNW	G	QQHGPFYWLFT	human	IGKV1-39
Unclustered	CDRL3	4JO1	L	LGGYDCSSGDCAA	rabbit	IGKV1S5
Unclustered	CDRL3	2OLD	A	SSYASGSTPRI	human	IGLV2-14
Unclustered	CDRL3	1YYL	L	QQYNNWPPRYT	human	IGKV3-15
Unclustered	CDRL3	1MCJ	B	SSYEGSDNFV	human	IGLV2-8
Unclustered	CDRL3	3TNN	F	SSYAGSTTFRV	human	IGLV2-23
Unclustered	CDRL3	4HIJ	A	QQRDNWPPDAT	human	IGKV3-11
Unclustered	CDRL3	2J88	L	QHHYGTRT	house mouse	IGKV12-44
Unclustered	CDRL3	4JY6	A	HIYDARGGTNWV	human	IGLV3-21
Unclustered	CDRL3	1EK3	B	QQYYSTPPT	human	IGKV4-1
Unclustered	CDRL3	2NY6	C	QQYNNWPPRYT	human	IGKV3-15
Unclustered	CDRL3	3MLV	M	QAWDASTGVSGGGTKLT	human	IGLV3-1
Unclustered	CDRL3	4K3H	A	SLSDVDGYL	human	IGLV7-46
Unclustered	CDRL3	1MCK	A	SSYEGSDNFV	human	IGLV2-8
Unclustered	CDRL3	4DN3	L	HQYIQLHSFT	human	IGKV3-11
Unclustered	CDRL3	4C83	D	QQWNGYPPLT	house mouse	IGKV4-59
Unclustered	CDRL3	3HB3	D	QHHYGTPPLT	house mouse	IGKV12-44
Unclustered	CDRL3	3MLS	N	QAWDASTGVSGGGTKLT	human	IGLV3-1
Unclustered	CDRL3	1MCS	B	SSYEGSDNFV	human	IGLV2-8
Unclustered	CDRL3	3MLR	L	QAWDASTGVSGGGTKLT	human	IGLV3-1
Unclustered	CDRL3	3SO3	B	QQRSNWPPGYT	human	IGKV3-11
Unclustered	CDRL3	2QSC	L	QQYDNLGDLS	human	IGKV1-33
Unclustered	CDRL3	3MLS	M	QAWDASTGVSGGGTKLT	human	IGLV3-1
Unclustered	CDRL3	3FN0	L	QARLLLPQT	human	IGKV3D-11
Unclustered	CDRL3	4JY4	A	HIWDSRVPTKWV	human	IGLV3-21
Unclustered	CDRL3	2OTU	G	GVGDTIKEQFVYV	house mouse	IGLV3
Unclustered	CDRL3	1AY1	L	QQWSTYPLT	house mouse	IGKV4-55
Unclustered	CDRL3	4K3E	L	ASAEDSSSNAV	sheep	IGLV1S8
Unclustered	CDRL3	1MCH	A	SSYEGSDNFV	human	IGLV2-8
Unclustered	CDRL3	3MLV	L	QAWDASTGVSGGGTKLT	human	IGLV3-1
Unclustered	CDRL3	1IQD	A	QKYGTSAIT	human	IGKV3-20
Unclustered	CDRL3	4K3D	M	ASAEDSSSNAV	sheep	IGLV1S8
Unclustered	CDRL3	3RU8	L	QVYGASSYT	human	IGKV3-20
Unclustered	CDRL3	1OM3	M	QHYAGYSAT	human	IGKV1-5
Unclustered	CDRL3	1BGX	L	QQWSTYPLT	house mouse	IGKV4-55
Unclustered	CDRL3	1DCL	B	SSYEGSDNFV	human	IGLV2-8
Unclustered	CDRL3	2MCG	1	SSYEGSDNFV	human	IGLV2-8
Unclustered	CDRL3	1LHZ	B	SSYDGSSTSVV	human	IGLV2-23
Unclustered	CDRL3	4FZE	L	QAWASTTVI	human	IGLV3-1
Unclustered	CDRL3	1ZLS	L	QHYAGYSAT	human	IGKV1-5
Unclustered	CDRL3	3GO1	L	QSADSSGDYV	human	IGLV3-25
Unclustered	CDRL3	8FAB	A	QAWDNSASI	human	IGLV3-25

Unclustered	CDRL3	1MCE	A	SSYEGSDNFV	human	IGLV2-8
Unclustered	CDRL3	1I7Z	A	LYSREFPPWT	house mouse	IGKV3-12
Unclustered	CDRL3	3MLS	O	QAWDASTGVSGGGTKLT	human	IGLV3-1
Unclustered	CDRL3	1HZH	L	QVYGASSYT	human	IGKV3-20
Unclustered	CDRL3	1I7Z	C	LYSREFPPWT	house mouse	IGKV3-12
Unclustered	CDRL3	3TNN	L	SSYAGSTTFRV	human	IGLV2-23
Unclustered	CDRL3	4AIX	D	QAWDSSTAVV	human	IGLV3-1
Unclustered	CDRL3	3UJI	L	QAWDSTLGVV	human	IGLV3-1
Unclustered	CDRL3	2NXY	C	QQYNNWPPRYT	human	IGKV3-15
Unclustered	CDRL3	2OTU	C	GVGDTIKEQFVYV	house mouse	IGLV3
Unclustered	CDRL3	1KEM	L	FQGSHVPRT	house mouse	IGKV1-117
Unclustered	CDRL3	3MLU	L	QAWDASTGVSGGGTKLT	human	IGLV3-1
Unclustered	CDRL3	1Q1J	M	ATWDSGLSADWV	human	IGLV1-51
Unclustered	CDRL3	1AR1	D	QHHYGTPPLT	house mouse	IGKV12-44
Unclustered	CDRL3	2NY7	L	QVYGASSYT	human	IGKV3-20
Unclustered	CDRL3	3LMJ	L	GTWDSSLSTGQL	human	IGLV1-51
Unclustered	CDRL3	2OMN	A	SSYASGSTPRI	human	IGLV2-14
Unclustered	CDRL3	1MCI	B	SSYEGSDNFV	human	IGLV2-8
Unclustered	CDRL3	1FN4	A	YQYNNGYT	Norway rat	IGKV22S2
Unclustered	CDRL3	4C83	B	QQWNGYPPLT	house mouse	IGKV4-59
Unclustered	CDRL3	1A8J	L	SSYEGSDNFV	human	IGLV2-8
Unclustered	CDRL3	4HIH	A	QQRDNWPPDAT	human	IGKV3-11
Unclustered	CDRL3	4DCQ	A	GVGDTIKEQFVYV	house mouse	IGLV3
Unclustered	CDRL3	2MCG	2	SSYEGSDNFV	human	IGLV2-8
Unclustered	CDRL3	3OZ9	L	HQWSGFYT	house mouse	IGKV4-80
Unclustered	CDRL3	1MCI	A	SSYEGSDNFV	human	IGLV2-8
Unclustered	CDRL3	2QHR	L	GVGDTIKEQFVYV	house mouse	IGLV3
Unclustered	CDRL3	2I60	Q	QQYNNWPPRYT	human	IGKV3-15
Unclustered	CDRL3	2VXQ	L	QESLSASYT	human	IGKV1-6
Unclustered	CDRL3	3S96	D	GVGDTIKEQFVYV	house mouse	IGLV3
Unclustered	CDRL3	4FQ2	L	HMWDSRSGFSWS	human	IGLV3-21
Unclustered	CDRL3	3S96	B	GVGDTIKEQFVYV	house mouse	IGLV3
Unclustered	CDRL3	1N0X	L	QVYGASSYT	human	IGKV3-20
Unclustered	CDRL3	1N0X	M	QVYGASSYT	human	IGKV3-20
Unclustered	CDRL3	3VFG	L	QQDYSS	house mouse	IGKV6-32
Unclustered	CDRL3	3GHB	L	ATWDSGLSADWV	human	IGLV1-51
Unclustered	CDRL3	4K3G	B	SLSDVDGYL	human	IGLV7-46
Unclustered	CDRL3	3PNW	A	QQHGPFYWLFT	human	IGKV1-39
Unclustered	CDRL3	4HT1	L	QTAYYNSRPDTVA	rabbit	IGKV1S3
Unclustered	CDRL3	2OTW	A	GVGDTIKEQFVYV	house mouse	IGLV3
Unclustered	CDRL3	4HIH	C	QQRDNWPPDAT	human	IGKV3-11
Unclustered	CDRL3	1A7P	L	QHFWSTSRT	house mouse	IGKV12-41
Unclustered	CDRL3	1OCW	L	ALWYSNHLV	house mouse	IGLV1
Unclustered	CDRL3	2NY0	C	QQYNNWPPRYT	human	IGKV3-15
Unclustered	CDRL3	1MCK	B	SSYEGSDNFV	human	IGLV2-8
Unclustered	CDRL3	1MCH	B	SSYEGSDNFV	human	IGLV2-8
Unclustered	CDRL3	1G9M	L	QQYNNWPPRYT	human	IGKV3-15
Unclustered	CDRL3	3NH7	M	STFTMSGNGTV	human	IGLV3-1
Unclustered	CDRL3	1MCB	A	SSYEGSDNFV	human	IGLV2-8
Unclustered	CDRL3	4HII	C	QQRDNWPPDAT	human	IGKV3-11

Unclustered	CDRL3	1YYM	Q	QQYNNWPPRYT	human	IGKV3-15
Unclustered	CDRL3	1TZI	A	QQAYSSPDT	house mouse	IGKV6-17
Unclustered	CDRL3	4JO3	M	QQGYSISDIDNS	rabbit	IGKV1S3
Unclustered	CDRL3	3MLS	L	QAWDASTGVSGGGTKLT	human	IGLV3-1
Unclustered	CDRL3	4AIZ	D	QSADSSGTYVV	human	IGLV3-25
Unclustered	CDRL3	3Q1S	L	QARLLLPQT	human	IGKV3D-11
Unclustered	CDRL3	4K9E	L	QQWAVHSLIT	house mouse	IGKV1-12
Unclustered	CDRL3	2BRR	X	QQYSGYPYT	house mouse	IGKV4-57-1
Unclustered	CDRL3	3DGG	A	QHSRELLT	house mouse	IGKV3-12
Unclustered	CDRL3	2I60	L	QQYNNWPPRYT	human	IGKV3-15
Unclustered	CDRL3	2I5Y	Q	QQYNNWPPRYT	human	IGKV3-15
Unclustered	CDRL3	2I5Y	L	QQYNNWPPRYT	human	IGKV3-15
Unclustered	CDRL3	1JVK	A	SSYDGSSTSVV	human	IGLV2-23
Unclustered	CDRL3	4HIE	A	QQRDNWPPDAT	human	IGKV3-11
Unclustered	CDRL3	2BJM	L	ALWYSNHLV	house mouse	IGLV1
Unclustered	CDRL3	4K3D	L	ASAEDSSSNAV	sheep	IGLV1S8
Unclustered	CDRL3	1YYL	Q	QQYNNWPPRYT	human	IGKV3-15
Unclustered	CDRL3	1LIL	A	QVWDSNASVV	human	IGLV3-1
Unclustered	CDRL3	2OTU	E	GVGDTIKEQFVYV	house mouse	IGLV3
Unclustered	CDRL3	1RZ8	C	QQYNNWPPRYT	human	IGKV3-15
Unclustered	CDRL3	1A8J	H	SSYEGSDNFV	human	IGLV2-8
Unclustered	CDRL3	1MCQ	B	SSYEGSDNFV	human	IGLV2-8
Unclustered	CDRL3	4DVB	B	QHYSAYPRT	house mouse	IGKV4-57-1
Unclustered	CDRL3	2NY5	L	QQYNNWPPRYT	human	IGKV3-15
Unclustered	CDRL3	3ZL4	L	GTWDSSLNPV	human	IGLV1-51
Unclustered	CDRL3	4AIZ	B	QSADSSGTYVV	human	IGLV3-25
Unclustered	CDRL3	1RZJ	L	QQYNNWPPRYT	human	IGKV3-15
Unclustered	CDRL3	1MCF	A	SSYEGSDNFV	human	IGLV2-8
Unclustered	CDRL3	2BRR	L	QQYSGYPYT	house mouse	IGKV4-57-1
Unclustered	CDRL3	3OAY	L	QHYAGYSAT	human	IGKV1-5
Unclustered	CDRL3	3GBN	L	ATWDRRPTAYVV	human	IGLV1-51
Unclustered	CDRL3	4OCR	L	GTWDTSLSGGGV	human	IGLV1-51
Unclustered	CDRL3	1LGV	B	SSYDGSSTSVV	human	IGLV2-23
Unclustered	CDRL3	1RZ8	A	QQYNNWPPRYT	human	IGKV3-15
Unclustered	CDRL3	1NFD	E	LSSYGDNNDLV	house mouse	IGKV10-94
Unclustered	CDRL3	4G6F	D	SSRDKSGSRLSV	human	IGLV3-19
Unclustered	CDRL3	3PNW	P	QQHGPFYWLFT	human	IGKV1-39
Unclustered	CDRL3	4AIZ	A	QSADSSGTYVV	human	IGLV3-25
Unclustered	CDRL3	4M1D	L	ATWDSGLSADWV	human	IGLV1-51
Unclustered	CDRL3	1ZLV	L	QHYAGYSAT	human	IGKV1-5
Unclustered	CDRL3	4HFW	A	QKTLRTWT	human	IGKV1-6
Unclustered	CDRL3	1MCC	B	SSYEGSDNFV	human	IGLV2-8
Unclustered	CDRL3	4HPY	L	YSTDSSGNHRV	human	IGLV3-10
Unclustered	CDRL3	4JO4	L	QQGYSISDIDNS	rabbit	IGKV1S3
Unclustered	CDRL3	4NPY	A	QVWDSRGPTNWV	human	IGLV3-21
Unclustered	CDRL3	3MCG	2	SSYEGSDNFV	human	IGLV2-8
Unclustered	CDRL3	3PNW	J	QQHGPFYWLFT	human	IGKV1-39
Unclustered	CDRL3	4K3H	C	SLSDVDGYL	human	IGLV7-46
Unclustered	CDRL3	1OP3	L	QHYAGYSAT	human	IGKV1-5
Unclustered	CDRL3	3PNW	M	QQHGPFYWLFT	human	IGKV1-39

Unclustered	CDRL3	1JVK	B	SSYDGSSTSVV	human	IGLV2-23
Unclustered	CDRL3	4K3H	D	SLSDVDGYL	human	IGLV7-46
Unclustered	CDRL3	3OAU	L	QHYAGYSAT	human	IGKV1-5
Unclustered	CDRL3	2XRA	L	QHYDDLPRIT	human	IGKV1-33
Unclustered	CDRL3	2NY2	C	QQYNNWPPRYT	human	IGKV3-15
Unclustered	CDRL3	3C08	L	QQWSSHIFT	human	IGKV1-39
Unclustered	CDRL3	2OMN	B	SSYASGSTPRI	human	IGLV2-14
Unclustered	CDRL3	4G6F	L	SSRDKSGSRLSV	human	IGLV3-19
Unclustered	CDRL3	2JB5	L	RSWDSNLSYSV	human	IGLV2-23
Unclustered	CDRL3	2BX5	I	QQSYSTPNT	human	IGKV1-39
Unclustered	CDRL3	1MQK	L	QHHYGTPPLT	house mouse	IGKV12-44
Unclustered	CDRL3	2OQJ	G	QHYAGYSAT	human	IGKV1-5
Unclustered	CDRL3	2NXZ	C	QQYNNWPPRYT	human	IGKV3-15
Unclustered	CDRL3	1FN4	C	YQYNNGYT	Norway rat	IGKV22S2
Unclustered	CDRL3	4ISV	A	GVGDTIKEQFVYV	house mouse	IGLV3
Unclustered	CDRL3	4K3G	A	SLSDVDGYL	human	IGLV7-46
H1-4-A	CDRH1	1KXQ	G	TYTD	Arabian camel	IGHV1S45
H1-4-A	CDRH1	1OP9	A	GYTY	Arabian camel	IGHV1S45
H1-4-A	CDRH1	1KXQ	E	TYTD	Arabian camel	IGHV1S45
H1-4-A	CDRH1	1KXQ	H	TYTD	Arabian camel	IGHV1S45
H1-4-A	CDRH1	3EBA	A	GYTY	Arabian camel	IGHV1S50
H1-4-A	CDRH1	4C58	B	EYTS	llama	IGHV
H1-4-A	CDRH1	1KXQ	F	TYTD	Arabian camel	IGHV1S45
H1-4-A	CDRH1	4C59	B	EYTS	llama	IGHV
H1-6-A	CDRH1	2QTE	B	KVTNKD	channel catfish	NITR11
H1-6-A	CDRH1	2QQQ	D	KVTNKN	channel catfish	NITR11
H1-6-A	CDRH1	2QQQ	C	KVTNKN	channel catfish	NITR11
H1-6-A	CDRH1	2QQQ	A	KVTNKN	channel catfish	NITR11
H1-6-A	CDRH1	2QTE	C	KVTNKD	channel catfish	NITR11
H1-6-A	CDRH1	2QTE	A	KVTNKD	channel catfish	NITR11
H1-6-A	CDRH1	2QQQ	B	KVTNKN	channel catfish	NITR11
H1-7-A	CDRH1	2XKN	D	GFTFSRY	house mouse	IGHV5S9
H1-7-A	CDRH1	3V6F	A	GFTFSSY	house mouse	IGHV5-6-1
H1-7-A	CDRH1	1AD9	H	GYTFTDY	house mouse	IGHV1-2
H1-7-A	CDRH1	4DKF	H	GFSFTSY	human	IGHV3-23
H1-7-A	CDRH1	1BLN	D	GFTFSSY	house mouse	IGHV5-9
H1-7-A	CDRH1	1WC7	H	GFTFTDY	house mouse	IGHV7-3
H1-7-A	CDRH1	4FZE	H	RGTLSSY	human	IGHV1-69
H1-7-A	CDRH1	4D9Q	H	GYTFTNY	human	IGHV7-4-1
H1-7-A	CDRH1	1YY9	D	GFSLTNY	house mouse	IGHV2-2
H1-7-A	CDRH1	1VFA	B	GFSLTGY	house mouse	IGHV2-6-7
H1-7-A	CDRH1	1OAU	J	GYTFTSY	house mouse	IGHV1-72
H1-7-A	CDRH1	2J4W	H	GFIFSDY	house mouse	IGHV5-4
H1-7-A	CDRH1	1DQL	H	GFTFSSY	human	IGHV3-30
H1-7-A	CDRH1	4GMT	H	GYTFSSY	house mouse	IGHV1-9
H1-7-A	CDRH1	3MLR	H	GYNFLDS	human	IGHV5-51
H1-7-A	CDRH1	4HKZ	B	GFNIKDT	human	IGHV3-66
H1-7-A	CDRH1	3UTZ	C	GFAFSTY	house mouse	IGHV5-9
H1-7-A	CDRH1	1XGP	B	GDSVTSD	house mouse	IGHV3-8
H1-7-A	CDRH1	1F4Y	H	GYVFSTY	house mouse	IGHV5-12-1

H1-7-A	CDRH1	3TCL	H	GFIFENF	human	IGHV3-20
H1-7-A	CDRH1	2XKN	B	GFTFSRY	house mouse	IGHV5S9
H1-7-A	CDRH1	1NCA	H	GYTFTNY	house mouse	IGHV9-3
H1-7-A	CDRH1	2XQY	G	GYSFTSY	house mouse	IGHV1S126
H1-7-A	CDRH1	1J1P	H	GDSITSD	house mouse	IGHV3-8
H1-7-A	CDRH1	1UZ6	H	GFDFSGY	house mouse	IGHV4-1
H1-7-A	CDRH1	1DEE	B	GFTFSGY	human	IGHV3-30
H1-7-A	CDRH1	1YEJ	H	GYIFTSY	house mouse	IGHV1-76
H1-7-A	CDRH1	3SGD	J	GFSFNTN	house mouse	IGHV10S3
H1-7-A	CDRH1	1OAU	H	GYTFTSY	house mouse	IGHV1-72
H1-7-A	CDRH1	3NH7	I	GFTFSNY	human	IGHV3-48
H1-7-A	CDRH1	2V7N	D	GFTFRNS	human	IGHV3-30
H1-7-A	CDRH1	1E4W	H	GFTFTNY	house mouse	IGHV1S81
H1-7-A	CDRH1	4G6J	H	GFTFSVY	human	IGHV3-33
H1-7-A	CDRH1	7FAB	H	GTSFDDY	human	IGHV4-59
H1-7-A	CDRH1	4HIX	H	GFTFSNY	house mouse	IGHV3-7
H1-7-A	CDRH1	4F58	J	GFSFSDF	human	IGHV3-30
H1-7-A	CDRH1	3S34	H	GFTFSSY	human	IGHV3-21
H1-7-A	CDRH1	4D9L	H	GGNFNTY	human	IGHV1-69
H1-7-A	CDRH1	1I8K	B	GFTFRKF	house mouse	IGHV5-6
H1-7-A	CDRH1	3BZ4	B	GLTFSNY	house mouse	IGHV6-3
H1-7-A	CDRH1	1F4W	H	GYVFSTY	house mouse	IGHV5-12-1
H1-7-A	CDRH1	2Y06	H	GYTFTSY	house mouse	IGHV1-72
H1-7-A	CDRH1	1N8Z	B	GFNIKDT	human	IGHV3-66
H1-7-A	CDRH1	1K6Q	H	GFNIKDY	house mouse	IGHV14-1
H1-7-A	CDRH1	3HZV	B	GFTFTDY	house mouse	IGHV7-3
H1-7-A	CDRH1	2FJG	H	GFTISDY	human	IGHV3-23
H1-7-A	CDRH1	3CLE	H	GYRFTSY	house mouse	IGHV1-12
H1-7-A	CDRH1	2GK0	B	GFSLTNY	house mouse	IGHV2-9
H1-7-A	CDRH1	3IF1	D	GFTFSDA	house mouse	IGHV6-6
H1-7-A	CDRH1	2OTU	F	GFTFRDY	house mouse	IGHV5-12
H1-7-A	CDRH1	2AI0	K	GFTFRNY	house mouse	IGHV5S4
H1-7-A	CDRH1	2FJF	F	GFTISDY	human	IGHV3-23
H1-7-A	CDRH1	4DVB	A	GFTFSRY	house mouse	IGHV5S9
H1-7-A	CDRH1	3QPQ	H	GYIFTTY	house mouse	IGHV1S81
H1-7-A	CDRH1	4F33	B	GYSFTGY	house mouse	IGHV1-37
H1-7-A	CDRH1	4JG1	H	GFTFRKF	human	IGHV3-23
H1-7-A	CDRH1	3M8O	H	GFTLSGS	human	IGHV3-73
H1-7-A	CDRH1	3ZHK	B	GFTFSSY	human	IGHV3-23
H1-7-A	CDRH1	1ZLU	H	NFRISAH	human	IGHV3-21
H1-7-A	CDRH1	2VDR	H	GFNIKDT	house mouse	IGHV14-3
H1-7-A	CDRH1	3TNN	H	GFTFSTY	human	IGHV3-23
H1-7-A	CDRH1	43C9	B	GISLSRY	house mouse	IGHV2-6-4
H1-7-A	CDRH1	3EOA	H	GYSFTGH	human	IGHV3-7
H1-7-A	CDRH1	2VL5	C	GYTFTSY	house mouse	IGHV1-61
H1-7-A	CDRH1	1OHQ	A	GFRISDE	human	IGHV3-23
H1-7-A	CDRH1	1TQB	B	GYTFTNY	house mouse	IGHV9-3-1
H1-7-A	CDRH1	1W72	I	GFTFDDY	human	IGHV3-9
H1-7-A	CDRH1	1TZH	B	GFDIYDD	house mouse	IGHV5-17
H1-7-A	CDRH1	3OGO	G	GFPVNRY	Arabian camel	IGHV1S26

H1-7-A	CDRH1	1DQQ	B	GDSVTSD	house mouse	IGHV3-8
H1-7-A	CDRH1	3C2A	I	GFTFSDV	human	IGHV3-15
H1-7-A	CDRH1	4LLY	C	GFTFTDY	human	IGHV3-66
H1-7-A	CDRH1	1BJ1	H	GYTFTNY	human	IGHV7-4-1
H1-7-A	CDRH1	4LSS	H	GYEFIDC	human	IGHV1-18
H1-7-A	CDRH1	1IND	H	GFTLSGE	house mouse	IGHV5-9
H1-7-A	CDRH1	2FJF	P	GFTISDY	human	IGHV3-23
H1-7-A	CDRH1	3T77	B	GFTFTDY	house mouse	IGHV7-3
H1-7-A	CDRH1	4FQ2	H	GDSMNNY	human	IGHV4-59
H1-7-A	CDRH1	1NC2	D	GFSLTDY	house mouse	IGHV2-2
H1-7-A	CDRH1	1HH9	B	GYIFTDY	house mouse	IGHV1-15
H1-7-A	CDRH1	2FJF	I	GFTISDY	human	IGHV3-23
H1-7-A	CDRH1	2NY6	D	GDTFIRY	human	IGHV1-69
H1-7-A	CDRH1	2Y36	H	GYTFTSY	house mouse	IGHV1-72
H1-7-A	CDRH1	4J6R	H	GYSFTDY	human	IGHV1-2
H1-7-A	CDRH1	3OKN	B	GFTFTDY	house mouse	IGHV7-3
H1-7-A	CDRH1	2B2X	I	GFTFSRY	house mouse	IGHV5-9
H1-7-A	CDRH1	3OKD	B	GFTFTDY	house mouse	IGHV7-3
H1-7-A	CDRH1	3S96	C	GYTFTTY	house mouse	IGHV9-3
H1-7-A	CDRH1	3IKC	B	GFTFTDY	house mouse	IGHV7-3
H1-7-A	CDRH1	1F2X	L	GYTVSTY	Arabian camel	IGHV1S45
H1-7-A	CDRH1	1CFQ	B	GYIFTDY	house mouse	IGHV1S52
H1-7-A	CDRH1	3KYM	N	GFTFSIY	human	IGHV3-23
H1-7-A	CDRH1	2I60	R	GDTFIRY	human	IGHV1-69
H1-7-A	CDRH1	4JPI	A	GYTFTGY	human	IGHV1-2
H1-7-A	CDRH1	4NCC	2	GFDFSRY	house mouse	IGHV4-1
H1-7-A	CDRH1	3E8U	H	GYTFTHY	house mouse	IGHV9-1
H1-7-A	CDRH1	3HI6	H	GFTFSRY	human	IGHV3-23
H1-7-A	CDRH1	3GNM	H	GYTFTTY	house mouse	IGHV1-7
H1-7-A	CDRH1	3CFB	B	GITFSRY	house mouse	IGHV5-9
H1-7-A	CDRH1	3C5S	D	GLTFSNY	house mouse	IGHV6-3
H1-7-A	CDRH1	2OJZ	H	GYTFTDY	house mouse	IGHV1-84
H1-7-A	CDRH1	4G7V	H	GFNVSSS	human	IGHV3-66
H1-7-A	CDRH1	3CFJ	D	GFDFRRY	house mouse	IGHV4-1
H1-7-A	CDRH1	1ZAN	H	GFSLTNN	Norway rat	IGHV2S41
H1-7-A	CDRH1	1N64	H	GYTFTDF	house mouse	IGHV9-2-1
H1-7-A	CDRH1	4HXB	H	GYTFTNY	house mouse	IGHV9-3
H1-7-A	CDRH1	3DUS	B	GFTFTDY	house mouse	IGHV7-3
H1-7-A	CDRH1	1FL3	A	GITFSRY	house mouse	IGHV5-9
H1-7-A	CDRH1	1MPA	H	GYSFTSY	house mouse	IGHV1-5
H1-7-A	CDRH1	4AL8	H	GYTFSSY	human	IGHV1-69
H1-7-A	CDRH1	1Q1J	I	GFTFSDV	human	IGHV3-15
H1-7-A	CDRH1	4JPK	H	GYTFTGY	human	IGHV1-2
H1-7-A	CDRH1	2A1W	H	GFTFRNY	house mouse	IGHV5S4
H1-7-A	CDRH1	1ZA6	H	GYTFTDH	house mouse	IGHV1
H1-7-A	CDRH1	4HCR	M	GYTFTSY	house mouse	IGHV1-18
H1-7-A	CDRH1	1OAX	H	GYTFTSY	house mouse	IGHV1-72
H1-7-A	CDRH1	4M1D	H	GFTFSDV	human	IGHV3-15
H1-7-A	CDRH1	2DTM	H	GFTFSNY	house mouse	IGHV5S9
H1-7-A	CDRH1	1NLB	H	GYTFTDF	house mouse	IGHV9-2-1

H1-7-A	CDRH1	1G9M	H	GDTFIRY	human	IGHV1-69
H1-7-A	CDRH1	1BFO	B	GFTFTDF	Norway rat	IGHV7S6
H1-7-A	CDRH1	4LMQ	H	GFSLTVY	human	IGHV3-23
H1-7-A	CDRH1	1LO4	H	GFTLRTY	house mouse	IGHV5S4
H1-7-A	CDRH1	2QHR	H	GFAFSSY	house mouse	IGHV5-12-1
H1-7-A	CDRH1	4NCC	H	GFDFSRY	house mouse	IGHV4-1
H1-7-A	CDRH1	3PNW	T	GFNLSSS	human	IGHV3-66
H1-7-A	CDRH1	1F3D	H	GYSFIDY	house mouse	IGHV1S135
H1-7-A	CDRH1	3ZKQ	D	GFTFSSA	llama	IGHV1S6
H1-7-A	CDRH1	3U9P	H	GFSLTSN	Norway rat	IGHV2S5
H1-7-A	CDRH1	3MLT	I	GYNFLDS	human	IGHV5-51
H1-7-A	CDRH1	3A6B	H	GDSITSD	house mouse	IGHV3-8
H1-7-A	CDRH1	3GJF	H	GFTFSTY	human	IGHV3-23
H1-7-A	CDRH1	3HC4	H	GYTFTTY	human	IGHV1-3
H1-7-A	CDRH1	3CFI	F	GFAFSGY	llama	IGHV1S6
H1-7-A	CDRH1	2A6I	B	GYTFTSY	house mouse	IGHV1-58
H1-7-A	CDRH1	3LS5	H	GFTFNNY	house mouse	IGHV5-9-3
H1-7-A	CDRH1	4EIG	B	GIIFSVY	llama	IGHV1S3
H1-7-A	CDRH1	1MFB	H	GYTFTNY	house mouse	IGHV1-5
H1-7-A	CDRH1	3HNS	H	GYSFTNY	house mouse	IGHV1-5
H1-7-A	CDRH1	1NC2	B	GFSLTDY	house mouse	IGHV2-2
H1-7-A	CDRH1	3MCL	H	GYTFSTS	house mouse	IGHV1-9
H1-7-A	CDRH1	1NGP	H	GYTFTSY	house mouse	IGHV1-72
H1-7-A	CDRH1	3CFI	C	GFAFSGY	llama	IGHV1S6
H1-7-A	CDRH1	2A1W	I	GFTFRNY	house mouse	IGHV5S4
H1-7-A	CDRH1	1Y18	H	GFDFRRY	house mouse	IGHV4-1
H1-7-A	CDRH1	1LO3	Y	GFSFRNY	house mouse	IGHV5S4
H1-7-A	CDRH1	1A6V	I	GYTFTSY	house mouse	IGHV1-72
H1-7-A	CDRH1	3O6M	H	GFAFSSY	house mouse	IGHV5-9
H1-7-A	CDRH1	2O5X	H	GYMFTNY	house mouse	IGHV9-3-1
H1-7-A	CDRH1	1MJJ	H	GYTFTNY	house mouse	IGHV1S61
H1-7-A	CDRH1	3CMO	Y	GYTFTSY	house mouse	IGHV1-5
H1-7-A	CDRH1	3GK8	H	GYTFTNY	house mouse	IGHV1-85
H1-7-A	CDRH1	3DIF	B	GYSFTSY	house mouse	IGHV1-66
H1-7-A	CDRH1	1YJD	H	GYTFTSY	house mouse	IGHV1S12
H1-7-A	CDRH1	3S96	A	GYTFTTY	house mouse	IGHV9-3
H1-7-A	CDRH1	1Y0L	F	GFDFRRY	house mouse	IGHV4-1
H1-7-A	CDRH1	4F33	F	GYSFTGY	house mouse	IGHV1-37
H1-7-A	CDRH1	1RUQ	H	GYTFTNY	house mouse	IGHV1-63
H1-7-A	CDRH1	1RMF	H	GYTFIDY	house mouse	IGHV1-67
H1-7-A	CDRH1	3LN9	A	GYTFSHR	Arabian camel	IGHV1S42
H1-7-A	CDRH1	1QKZ	H	GFTFSDY	house mouse	IGHV5-4
H1-7-A	CDRH1	43CA	D	GISLSRY	house mouse	IGHV2-6-4
H1-7-A	CDRH1	3H3P	H	GGSFSTY	human	IGHV1-69
H1-7-A	CDRH1	2FJF	K	GFTISDY	human	IGHV3-23
H1-7-A	CDRH1	4G5Z	H	GFTFSVY	human	IGHV3-33
H1-7-A	CDRH1	3VFG	H	GFSVTNY	house mouse	IGHV2-9
H1-7-A	CDRH1	3NH7	K	GFTFSNY	human	IGHV3-48
H1-7-A	CDRH1	3MXW	H	GYTFIDE	human	IGHV1-2
H1-7-A	CDRH1	3NFS	H	GYTFTSY	human	IGHV1-46

H1-7-A	CDRH1	2VYR	F	GFTFEEY	human	IGHV3-23
H1-7-A	CDRH1	1UB6	A	GITFSRY	house mouse	IGHV5-9
H1-7-A	CDRH1	3T3M	H	GFNIKDT	house mouse	IGHV14-3
H1-7-A	CDRH1	3MNV	D	GYTFTDY	house mouse	IGHV9-2-1
H1-7-A	CDRH1	3FFD	A	GFTFSSY	house mouse	IGHV5-6-1
H1-7-A	CDRH1	2VH5	H	GFTFSTF	human	IGHV3-48
H1-7-A	CDRH1	4FHB	D	GRTFSSY	llama	IGHV
H1-7-A	CDRH1	3NID	H	GFNIKDT	house mouse	IGHV14-3
H1-7-A	CDRH1	3WKM	H	GFNIKDT	house mouse	IGHV14-3
H1-7-A	CDRH1	1C5C	H	GYSFTSY	house mouse	IGHV1-53
H1-7-A	CDRH1	3PGF	H	GFNFSSS	human	IGHV3-66
H1-7-A	CDRH1	3T2N	I	GYTFTDY	human	IGHV7-4-1
H1-7-A	CDRH1	1QYG	H	GFTFSDA	house mouse	IGHV6-6
H1-7-A	CDRH1	1IGT	B	GFTFSDY	house mouse	IGHV5-12
H1-7-A	CDRH1	4LEO	A	GYTFRSS	human	IGHV1-18
H1-7-A	CDRH1	4M61	B	GYSFTGY	house mouse	IGHV1-39
H1-7-A	CDRH1	1J1O	H	GDSITSD	house mouse	IGHV3-8
H1-7-A	CDRH1	4H20	H	GYTFTDY	house mouse	IGHV1-19
H1-7-A	CDRH1	15C8	H	GFNIKDT	house mouse	IGHV14-3
H1-7-A	CDRH1	1N0X	H	GYRFSNF	human	IGHV1-3
H1-7-A	CDRH1	3IKC	D	GFTFTDY	house mouse	IGHV7-3
H1-7-A	CDRH1	2DQI	H	GDSITSD	house mouse	IGHV3-8
H1-7-A	CDRH1	1JPS	H	GFNIKEY	human	IGHV3-66
H1-7-A	CDRH1	1WT5	B	GYTFTSY	house mouse	IGHV1-2
H1-7-A	CDRH1	2YBR	D	GFTFDNY	human	IGHV3-9
H1-7-A	CDRH1	2OR9	I	GFTFSHY	house mouse	IGHV5-6
H1-7-A	CDRH1	1A3R	H	GFNIKDI	house mouse	IGHV14-3
H1-7-A	CDRH1	2V7N	H	GFTFRNS	human	IGHV3-30
H1-7-A	CDRH1	1Q0Y	H	GYTFSSY	house mouse	IGHV1-9
H1-7-A	CDRH1	3P9W	H	GFNIKDT	human	IGHV3-66
H1-7-A	CDRH1	2ADG	B	GFTFSSY	house mouse	IGHV5-6-1
H1-7-A	CDRH1	1Y0L	D	GFDFRRY	house mouse	IGHV4-1
H1-7-A	CDRH1	3GI9	H	GYTFTSY	house mouse	IGHV1-69
H1-7-A	CDRH1	3V6F	H	GFTFSSY	house mouse	IGHV5-6-1
H1-7-A	CDRH1	3ZKN	C	GFSLTRY	house mouse	IGHV2-9
H1-7-A	CDRH1	3CFK	H	GFDFRRY	house mouse	IGHV4-1
H1-7-A	CDRH1	3R06	H	GFTFSGY	house mouse	IGHV5-17
H1-7-A	CDRH1	3O6L	H	GFAFSSY	house mouse	IGHV5-9
H1-7-A	CDRH1	3B9V	D	GFNIKDT	human	IGHV3-66
H1-7-A	CDRH1	2FD6	H	GYSFTNF	house mouse	IGHV1-66
H1-7-A	CDRH1	1DBA	H	GYAFTNY	house mouse	IGHV9-3-1
H1-7-A	CDRH1	2CJU	H	GFTFTNY	house mouse	IGHV7-3
H1-7-A	CDRH1	4CAD	E	GYKFSSY	house mouse	IGHV1-9
H1-7-A	CDRH1	3V6F	E	GFTFSSY	house mouse	IGHV5-6-1
H1-7-A	CDRH1	25C8	H	GFNIKDT	house mouse	IGHV14-3
H1-7-A	CDRH1	1FN4	B	GFSLTSF	Norway rat	IGHV2S85
H1-7-A	CDRH1	3Q3G	B	GFNIKDI	house mouse	IGHV14-3
H1-7-A	CDRH1	3L95	H	GFTFSSY	human	IGHV3-74
H1-7-A	CDRH1	2GK0	H	GFSLTNY	house mouse	IGHV2-9
H1-7-A	CDRH1	1A2Y	B	GFSLTGY	house mouse	IGHV2-6-7

H1-7-A	CDRH1	3ZHK	A	GFTFSSY	human	IGHV3-23
H1-7-A	CDRH1	1A6T	B	GYSFSTY	house mouse	IGHV1-26
H1-7-A	CDRH1	2DQG	H	GDSITSD	house mouse	IGHV3-8
H1-7-A	CDRH1	1FVE	B	GFNIKDT	human	IGHV3-66
H1-7-A	CDRH1	2VDO	H	GFNIKDT	house mouse	IGHV14-3
H1-7-A	CDRH1	2EH8	H	GYAFSSS	house mouse	IGHV1-82
H1-7-A	CDRH1	1T4K	D	GFSLSDY	house mouse	IGHV2-6-5
H1-7-A	CDRH1	4GW4	A	GYKISDH	human	IGHV1-2
H1-7-A	CDRH1	2DQC	H	GDSITSD	house mouse	IGHV3-8
H1-7-A	CDRH1	3BPC	B	GFTFTDY	house mouse	IGHV7-3
H1-7-A	CDRH1	2XQB	H	GYSFSSF	human	IGHV1-18
H1-7-A	CDRH1	2YC1	D	GFTFDNY	human	IGHV3-9
H1-7-A	CDRH1	2AJ3	B	GASVNNY	human	IGHV4-59
H1-7-A	CDRH1	1A7Q	H	GFSLTGY	house mouse	IGHV2-6-7
H1-7-A	CDRH1	3OJD	B	GYIFTNY	house mouse	IGHV9-1
H1-7-A	CDRH1	2OQJ	K	NFRISAH	human	IGHV3-21
H1-7-A	CDRH1	3Q3G	D	GFNIKDI	house mouse	IGHV14-3
H1-7-A	CDRH1	2A77	H	GFTFRNY	house mouse	IGHV5S4
H1-7-A	CDRH1	1QBM	H	GFNIKDT	house mouse	IGHV14-3
H1-7-A	CDRH1	1YNK	H	GYTFSEY	house mouse	IGHV1-9
H1-7-A	CDRH1	3MLT	B	GYNFLDS	human	IGHV5-51
H1-7-A	CDRH1	2Q76	D	GYTFTTY	house mouse	IGHV1-9
H1-7-A	CDRH1	3MLS	H	GYNFLDS	human	IGHV5-51
H1-7-A	CDRH1	1YMH	B	GYTFTDF	house mouse	IGHV9-2-1
H1-7-A	CDRH1	4JFX	B	GFTFRKF	human	IGHV3-66
H1-7-A	CDRH1	3I9G	H	GYIFIDH	house mouse	IGHV1-78
H1-7-A	CDRH1	3ZHL	A	GFTFSSY	human	IGHV3-23
H1-7-A	CDRH1	1MHP	X	GFTFSRY	human	IGHV3-23
H1-7-A	CDRH1	2R23	B	GFTFTDY	house mouse	IGHV7-3
H1-7-A	CDRH1	1DLF	H	GFTFSDA	house mouse	IGHV6-6
H1-7-A	CDRH1	4F58	K	GFSFSDF	human	IGHV3-30
H1-7-A	CDRH1	1MF2	N	GFTFMRF	house mouse	IGHV5-17
H1-7-A	CDRH1	1NFD	H	GFTFSDF	hamster	IGHV6-6
H1-7-A	CDRH1	3OKO	B	GFTFTDY	house mouse	IGHV7-3
H1-7-A	CDRH1	4G6F	B	GFDFDNA	human	IGHV3-15
H1-7-A	CDRH1	2R1Y	B	GFTFTDY	house mouse	IGHV7-3
H1-7-A	CDRH1	1XF5	B	GYTFTDF	house mouse	IGHV9-2-1
H1-7-A	CDRH1	2NY2	D	GDTFIRY	human	IGHV1-69
H1-7-A	CDRH1	3P0Y	H	GFTLSGD	human	IGHV3-66
H1-7-A	CDRH1	1OAZ	H	GYTFTSY	house mouse	IGHV1-72
H1-7-A	CDRH1	3CFK	N	GFDFRRY	house mouse	IGHV4-1
H1-7-A	CDRH1	1UA6	H	GDSITSD	house mouse	IGHV3-8
H1-7-A	CDRH1	3MLS	K	GYNFLDS	human	IGHV5-51
H1-7-A	CDRH1	2BFV	H	GFTFNTY	house mouse	IGHV5-6-2
H1-7-A	CDRH1	3ZKN	H	GFSLTRY	house mouse	IGHV2-9
H1-7-A	CDRH1	1ZTX	H	GYTFSDY	house mouse	IGHV1-9
H1-7-A	CDRH1	3UTZ	B	GFAFSTY	house mouse	IGHV5-9
H1-7-A	CDRH1	3B9K	H	GFSLISD	Norway rat	IGHV2S5
H1-7-A	CDRH1	2D03	H	GFSLTSY	house mouse	IGHV2-2
H1-7-A	CDRH1	2DDQ	H	GYTFTSY	house mouse	IGHV1-14

H1-7-A	CDRH1	1EHL	H	GYSFTSF	house mouse	IGHV1-5
H1-7-A	CDRH1	4HII	B	GFTFSRY	human	IGHV3-30
H1-7-A	CDRH1	1MH5	H	GYTFTSN	house mouse	IGHV1S61
H1-7-A	CDRH1	2NY4	D	GDTFIRY	human	IGHV1-69
H1-7-A	CDRH1	4JG0	H	GFTFRKF	human	IGHV3-23
H1-7-A	CDRH1	2OQJ	E	NFRISAH	human	IGHV3-21
H1-7-A	CDRH1	2NY5	H	GDTFIRY	human	IGHV1-69
H1-7-A	CDRH1	1KIP	B	GFSLTGA	house mouse	IGHV2-6-7
H1-7-A	CDRH1	1AQK	H	GFTFNNY	human	IGHV3-30
H1-7-A	CDRH1	4LLY	A	GFTFTDY	human	IGHV3-66
H1-7-A	CDRH1	1MHH	D	GYTFTDF	house mouse	IGHV9-2-1
H1-7-A	CDRH1	3PNW	E	GFNLSSS	human	IGHV3-66
H1-7-A	CDRH1	3NTC	H	GYTFTNS	house mouse	IGHV1-63
H1-7-A	CDRH1	4NM4	H	GYTFTAY	human	IGHV1-3
H1-7-A	CDRH1	1RFD	H	GFTFSDA	house mouse	IGHV6-6
H1-7-A	CDRH1	4JY6	D	GASINDA	human	IGHV4-59
H1-7-A	CDRH1	4F58	H	GFSFSDF	human	IGHV3-30
H1-7-A	CDRH1	1NGY	B	GYTFTSY	house mouse	IGHV1S36
H1-7-A	CDRH1	2R1X	B	GFTFTDY	house mouse	IGHV7-3
H1-7-A	CDRH1	3MUG	F	GFTFHKY	human	IGHV3-30
H1-7-A	CDRH1	4NRX	A	GYNFASE	human	IGHV5-51
H1-7-A	CDRH1	3QPQ	D	GYIFTTY	house mouse	IGHV1S81
H1-7-A	CDRH1	2DQE	H	GDSITSD	house mouse	IGHV3-8
H1-7-A	CDRH1	1UZ8	H	GFDFSGY	house mouse	IGHV4-1
H1-7-A	CDRH1	2DQD	H	GDSITSD	house mouse	IGHV3-8
H1-7-A	CDRH1	3FO1	H	GFSLTNY	house mouse	IGHV2-9
H1-7-A	CDRH1	1FJ1	B	GYTFTDY	house mouse	IGHV9-2-1
H1-7-A	CDRH1	3FO2	B	GFSLTNY	house mouse	IGHV2-9
H1-7-A	CDRH1	2EKS	B	GDSIRSD	human	IGHV4-59
H1-7-A	CDRH1	1NDG	B	GDSIIRD	house mouse	IGHV3-8
H1-7-A	CDRH1	3MUG	B	GFTFHKY	human	IGHV3-30
H1-7-A	CDRH1	2UYL	B	GYTFTSN	house mouse	IGHV1S61
H1-7-A	CDRH1	3PNW	N	GFNLSSS	human	IGHV3-66
H1-7-A	CDRH1	4OCS	H	QFTFSGH	human	IGHV3-30
H1-7-A	CDRH1	1MJJ	B	GYTFTNY	house mouse	IGHV1S61
H1-7-A	CDRH1	4HII	D	GFTFSRY	human	IGHV3-30
H1-7-A	CDRH1	3HC0	H	GYTFTTY	human	IGHV1-69
H1-7-A	CDRH1	1PG7	H	GFNIKEY	human	IGHV3-66
H1-7-A	CDRH1	1CZ8	Y	GYDFTHY	human	IGHV3-23
H1-7-A	CDRH1	1LO0	H	GFSFRNY	house mouse	IGHV5S4
H1-7-A	CDRH1	3MNZ	B	GYTFTDY	house mouse	IGHV9-2-1
H1-7-A	CDRH1	2BMK	B	GFTFTDY	house mouse	IGHV7-3
H1-7-A	CDRH1	4D9L	J	GGNFNTY	human	IGHV1-69
H1-7-A	CDRH1	2I5Y	R	GDTFIRY	human	IGHV1-69
H1-7-A	CDRH1	3UPC	J	GFTFSDE	human	IGHV3-23
H1-7-A	CDRH1	2XWT	A	GYSLTDN	human	IGHV5-51
H1-7-A	CDRH1	1NDM	B	GDSITSD	house mouse	IGHV3-8
H1-7-A	CDRH1	1FL3	H	GITFSRY	house mouse	IGHV5-9
H1-7-A	CDRH1	1M7D	B	GFTFSNY	house mouse	IGHV6-6
H1-7-A	CDRH1	4G6F	H	GFDFDNA	human	IGHV3-15

H1-7-A	CDRH1	1FE8	I	GFSLTTY	house mouse	IGHV2-3
H1-7-A	CDRH1	1OM3	H	NFRISAH	human	IGHV3-21
H1-7-A	CDRH1	2R56	H	GFTFRHH	human	IGHV3-23
H1-7-A	CDRH1	1N4X	I	NYAFTDY	house mouse	IGHV9-2-1
H1-7-A	CDRH1	1VPO	H	GFTFSRY	house mouse	IGHV5S9
H1-7-A	CDRH1	3CXD	H	GFTFNIY	house mouse	IGHV10-1
H1-7-A	CDRH1	2FR4	B	GYTFTSY	house mouse	IGHV1-14
H1-7-A	CDRH1	1UZ8	B	GFDFSGY	house mouse	IGHV4-1
H1-7-A	CDRH1	2YBR	A	GFTFDNY	human	IGHV3-9
H1-7-A	CDRH1	1IFH	H	GFSFSSY	house mouse	IGHV5-6-1
H1-7-A	CDRH1	1AD0	D	GFTFTDY	human	IGHV3-72
H1-7-A	CDRH1	1KIR	B	GFSLTGY	house mouse	IGHV2-6-7
H1-7-A	CDRH1	4J8R	B	GYAFTNY	house mouse	IGHV1-54
H1-7-A	CDRH1	4OCR	H	GFTFSNF	human	IGHV3-30-3
H1-7-A	CDRH1	3TNN	E	GFTFSTY	human	IGHV3-23
H1-7-A	CDRH1	1F2X	K	GYTVSTY	Arabian camel	IGHV1S45
H1-7-A	CDRH1	2NYY	D	GFTFKYD	house mouse	IGHV5-4
H1-7-A	CDRH1	3DUU	B	GFTFTDY	house mouse	IGHV7-3
H1-7-A	CDRH1	3UJI	H	GYSFSNY	human	IGHV5-51
H1-7-A	CDRH1	2OJZ	I	GYTFTDY	house mouse	IGHV1-84
H1-7-A	CDRH1	2VXV	H	GYTVTSY	human	IGHV5-51
H1-7-A	CDRH1	4K3J	H	GYTFTSY	human	IGHV3-74
H1-7-A	CDRH1	1KB5	H	GYSFTGY	house mouse	IGHV1-39
H1-7-A	CDRH1	1EO8	H	GYSFSTY	house mouse	IGHV1-9
H1-7-A	CDRH1	4HJG	B	GFNIKDT	human	IGHV3-66
H1-7-A	CDRH1	1Y18	B	GFDFRRY	house mouse	IGHV4-1
H1-7-A	CDRH1	4ENE	E	GFDYSRY	house mouse	IGHV4-1
H1-7-A	CDRH1	1TPX	B	GYTFTNY	house mouse	IGHV9-3-1
H1-7-A	CDRH1	4KFZ	C	GFSFSHS	human	IGHV3-23
H1-7-A	CDRH1	43C9	D	GISLSRY	house mouse	IGHV2-6-4
H1-7-A	CDRH1	35C8	H	GFNIKDT	house mouse	IGHV14-3
H1-7-A	CDRH1	1YEG	H	GYIFTSY	house mouse	IGHV1-76
H1-7-A	CDRH1	3QO0	B	GFTLSGS	human	IGHV3-73
H1-7-A	CDRH1	1FGN	H	GFNIKDY	house mouse	IGHV14-1
H1-7-A	CDRH1	3FO2	H	GFSLTNY	house mouse	IGHV2-9
H1-7-A	CDRH1	3A67	H	GDSITSD	house mouse	IGHV3-8
H1-7-A	CDRH1	2VXS	I	GFTFSSY	human	IGHV3-23
H1-7-A	CDRH1	3CLF	H	GYRFTSY	house mouse	IGHV1-12
H1-7-A	CDRH1	3ZKX	B	GFTFSSA	llama	IGHV1S6
H1-7-A	CDRH1	4K94	H	GFNISSY	human	IGHV3-66
H1-7-A	CDRH1	3V52	H	GFTFSDY	house mouse	IGHV5-16
H1-7-A	CDRH1	1WT5	A	GYTFTSY	house mouse	IGHV1-2
H1-7-A	CDRH1	1TZG	H	GGSFSTY	human	IGHV1-69
H1-7-A	CDRH1	4ETQ	H	GYSFNFY	house mouse	IGHV1S126
H1-7-A	CDRH1	2XQY	J	GYSFTSY	house mouse	IGHV1S126
H1-7-A	CDRH1	1C5B	H	GYSFTSY	house mouse	IGHV1-53
H1-7-A	CDRH1	1NC4	B	GFSLTDY	house mouse	IGHV2-2
H1-7-A	CDRH1	1OAX	J	GYTFTSY	house mouse	IGHV1-72
H1-7-A	CDRH1	43CA	H	GISLSRY	house mouse	IGHV2-6-4
H1-7-A	CDRH1	2B0S	H	GYSFSDY	human	IGHV5-51

H1-7-A	CDRH1	1IQW	H	GYTFTSY	house mouse	IGHV1-50
H1-7-A	CDRH1	1A6W	H	GYTFTSY	house mouse	IGHV1-72
H1-7-A	CDRH1	1A3L	H	GYTFTNY	house mouse	IGHV1-63
H1-7-A	CDRH1	3IET	D	GFTFSDA	house mouse	IGHV6-6
H1-7-A	CDRH1	3CFI	I	GFAFSGY	llama	IGHV1S6
H1-7-A	CDRH1	12E8	P	GFNIKDY	house mouse	IGHV14-4
H1-7-A	CDRH1	2FX8	I	GGSFSTY	human	IGHV1-69
H1-7-A	CDRH1	1GIG	H	GFLLISN	house mouse	IGHV2-9
H1-7-A	CDRH1	1Q9K	B	GFTFTDY	house mouse	IGHV7-3
H1-7-A	CDRH1	2UYL	W	GYTFTSN	house mouse	IGHV1S61
H1-7-A	CDRH1	2OTU	B	GFTFRDY	house mouse	IGHV5-12
H1-7-A	CDRH1	2R2B	B	GFTFTDY	house mouse	IGHV7-3
H1-7-A	CDRH1	2VDQ	H	GFNIKDT	house mouse	IGHV14-3
H1-7-A	CDRH1	3Q6G	I	GGSISNN	human	IGHV4-59
H1-7-A	CDRH1	3UJJ	H	GYSFSSY	human	IGHV5-51
H1-7-A	CDRH1	2EIZ	B	GDSIRSD	human	IGHV4-59
H1-7-A	CDRH1	2OTU	D	GFTFRDY	house mouse	IGHV5-12
H1-7-A	CDRH1	1W72	H	GFTFDDY	human	IGHV3-9
H1-7-A	CDRH1	3NAC	H	GYSFTNY	human	IGHV5-10-1
H1-7-A	CDRH1	3DUR	B	GFTFTDY	house mouse	IGHV7-3
H1-7-A	CDRH1	1ZA6	B	GYTFTDH	house mouse	IGHV1
H1-7-A	CDRH1	1DFB	H	GFTFNDY	human	IGHV3-9
H1-7-A	CDRH1	3FO9	H	GLTFSRF	house mouse	IGHV6-3
H1-7-A	CDRH1	2GSG	D	GFTFRDY	house mouse	IGHV5-12
H1-7-A	CDRH1	4NZU	H	GFTFGSY	human	IGHV3-30
H1-7-A	CDRH1	3O2V	H	GYMFTNY	house mouse	IGHV9-3-1
H1-7-A	CDRH1	1MEX	H	GYTFTDH	house mouse	IGHV1S53
H1-7-A	CDRH1	1UB6	H	GITFSRY	house mouse	IGHV5-9
H1-7-A	CDRH1	3LH2	H	GGSFSTY	human	IGHV1-69
H1-7-A	CDRH1	3V0V	A	GFTFSDY	house mouse	IGHV7-3
H1-7-A	CDRH1	2AI0	J	GFTFRNY	house mouse	IGHV5S4
H1-7-A	CDRH1	1RJL	B	GFTFTSS	house mouse	IGHV1S130
H1-7-A	CDRH1	1FL5	H	GFTFTDY	house mouse	IGHV3-72
H1-7-A	CDRH1	2H9G	B	GFSIGKS	human	IGHV3-66
H1-7-A	CDRH1	4GMT	I	GYTFSSY	house mouse	IGHV1-9
H1-7-A	CDRH1	2NY7	H	GYRFSNF	human	IGHV1-3
H1-7-A	CDRH1	3K1K	C	GFPVNRY	llama	IGHV1S1
H1-7-A	CDRH1	4GW1	D	GFSLTNY	house mouse	IGHV2-2
H1-7-A	CDRH1	1AD9	B	GYTFTDY	house mouse	IGHV1-2
H1-7-A	CDRH1	3DVG	B	GFNVKTG	human	IGHV3-66
H1-7-A	CDRH1	2QQN	H	GFTFSSY	human	IGHV3-23
H1-7-A	CDRH1	2C1P	H	GYSFTNY	house mouse	IGHV1-61
H1-7-A	CDRH1	2VYR	J	GFTFEEY	human	IGHV3-23
H1-7-A	CDRH1	4HHA	B	GFTFSNH	human	IGHV3-30
H1-7-A	CDRH1	1LO0	Y	GFSFRNY	house mouse	IGHV5S4
H1-7-A	CDRH1	3KYM	D	GFTFSIY	human	IGHV3-23
H1-7-A	CDRH1	4M1D	I	GFTFSDV	human	IGHV3-15
H1-7-A	CDRH1	1A7R	H	GFSLTGY	house mouse	IGHV2-6-7
H1-7-A	CDRH1	3OAY	M	NFRISAH	human	IGHV3-21
H1-7-A	CDRH1	3PP3	I	GYAFSYS	house mouse	IGHV1-69

H1-7-A	CDRH1	2FX8	K	GGSFSTY	human	IGHV1-69
H1-7-A	CDRH1	3HI6	X	GFTFSRY	human	IGHV3-23
H1-7-A	CDRH1	4D9L	K	GGNFNTY	human	IGHV1-69
H1-7-A	CDRH1	4JJ5	B	GYSFTTY	house mouse	IGHV9-3
H1-7-A	CDRH1	3G6A	B	GFTFNSY	human	IGHV3-30
H1-7-A	CDRH1	3MLV	N	GYNFLDS	human	IGHV5-51
H1-7-A	CDRH1	1IGT	D	GFTFSDY	house mouse	IGHV5-12
H1-7-A	CDRH1	3PNW	Q	GFNLSSS	human	IGHV3-66
H1-7-A	CDRH1	4GXV	I	GFTFSSY	human	IGHV3-30
H1-7-A	CDRH1	1CFV	H	GFTFNTY	house mouse	IGHV5-6-2
H1-7-A	CDRH1	4HKX	A	GYTFTDN	human	IGHV1-2
H1-7-A	CDRH1	3EO9	H	GYSFTGH	human	IGHV3-7
H1-7-A	CDRH1	1PG7	X	GYSFTGH	house mouse	IGHV1-37
H1-7-A	CDRH1	1E6O	H	GYTFTSY	house mouse	IGHV1-4
H1-7-A	CDRH1	4HH9	D	GFTFSNH	human	IGHV3-30
H1-7-A	CDRH1	3MNV	B	GYTFTDY	house mouse	IGHV9-2-1
H1-7-A	CDRH1	1Q9W	B	GFTFTDY	house mouse	IGHV7-3
H1-7-A	CDRH1	3BZ4	H	GLTFSNY	house mouse	IGHV6-3
H1-7-A	CDRH1	1MHH	B	GYTFTDF	house mouse	IGHV9-2-1
H1-7-A	CDRH1	1UJ3	B	GFNIKDY	human	IGHV1-46
H1-7-A	CDRH1	1MFA	H	GYTFTNY	house mouse	IGHV1-5
H1-7-A	CDRH1	1IBG	H	GFSLTTY	house mouse	IGHV2-9
H1-7-A	CDRH1	1UB5	A	GITFSRY	house mouse	IGHV5-9
H1-7-A	CDRH1	3CFJ	H	GFDFRRY	house mouse	IGHV4-1
H1-7-A	CDRH1	1CLO	H	GFTFTDY	house mouse	IGHV7-3
H1-7-A	CDRH1	1DVF	D	GFNIKDT	house mouse	IGHV14-3
H1-7-A	CDRH1	1NGQ	H	GYTFTSY	house mouse	IGHV1-72
H1-7-A	CDRH1	3P9W	F	GFNIKDT	human	IGHV3-66
H1-7-A	CDRH1	3UPC	F	GFTFSDE	human	IGHV3-23
H1-7-A	CDRH1	2B1A	H	GYSFSDY	human	IGHV5-51
H1-7-A	CDRH1	1RZJ	H	GDTFIRY	human	IGHV1-69
H1-7-A	CDRH1	4N9G	M	GGSFSSY	human	IGHV4-34
H1-7-A	CDRH1	1UWX	H	GYTFTNF	house mouse	IGHV9-3-1
H1-7-A	CDRH1	2VXU	H	GYSFTDY	house mouse	IGHV1S135
H1-7-A	CDRH1	2BRR	Y	GYTFTNY	house mouse	IGHV9-3-1
H1-7-A	CDRH1	1KN2	H	GYIFTSY	house mouse	IGHV1-76
H1-7-A	CDRH1	3BZ4	F	GLTFSNY	house mouse	IGHV6-3
H1-7-A	CDRH1	3LH2	I	GGSFSTY	human	IGHV1-69
H1-7-A	CDRH1	4DVB	H	GFTFSRY	house mouse	IGHV5S9
H1-7-A	CDRH1	1NCC	H	GYTFTNY	house mouse	IGHV9-3
H1-7-A	CDRH1	1DBB	H	GYAFTNY	house mouse	IGHV9-3-1
H1-7-A	CDRH1	1IGF	J	GFTFSRC	house mouse	IGHV5-9-3
H1-7-A	CDRH1	2FJG	B	GFTISDY	human	IGHV3-23
H1-7-A	CDRH1	4LLU	A	GFTFTDY	human	IGHV3-66
H1-7-A	CDRH1	1FVD	B	GFNIKDT	human	IGHV3-66
H1-7-A	CDRH1	2D7T	H	GYTFTGN	human	IGHV1-2
H1-7-A	CDRH1	3OKE	B	GFTFTDY	house mouse	IGHV7-3
H1-7-A	CDRH1	2V7H	B	GYTFSNY	house mouse	IGHV1-9
H1-7-A	CDRH1	4F58	I	GFSFSDF	human	IGHV3-30
H1-7-A	CDRH1	1SEQ	H	GFTFSTY	house mouse	IGHV5-12-2

H1-7-A	CDRH1	1KN4	H	GYIFTSY	house mouse	IGHV1-76
H1-7-A	CDRH1	1RZ7	H	GYTFSDF	human	IGHV1-69-2
H1-7-A	CDRH1	3D9A	H	GDSITSD	house mouse	IGHV3-8
H1-7-A	CDRH1	1INE	H	GFTLSGE	house mouse	IGHV5-9
H1-7-A	CDRH1	1MRE	H	GYTFTSY	house mouse	IGHV1-50
H1-7-A	CDRH1	4JY5	H	GTLVRDN	human	IGHV4-59
H1-7-A	CDRH1	2ADI	B	GFTFSSY	house mouse	IGHV5-6-1
H1-7-A	CDRH1	12E8	H	GFNIKDY	house mouse	IGHV14-4
H1-7-A	CDRH1	3KS0	H	GDSITSG	house mouse	IGHV3-8
H1-7-A	CDRH1	3SOB	H	GFTFTNS	human	IGHV3-11
H1-7-A	CDRH1	43CA	F	GISLSRY	house mouse	IGHV2-6-4
H1-7-A	CDRH1	2Y07	H	GYTFTSY	house mouse	IGHV1-72
H1-7-A	CDRH1	2FJF	V	GFTISDY	human	IGHV3-23
H1-7-A	CDRH1	3UPC	H	GFTFSDE	human	IGHV3-23
H1-7-A	CDRH1	2Y6S	D	GYTFTDY	house mouse	IGHV1-77
H1-7-A	CDRH1	1WZ1	H	GFTFSDA	house mouse	IGHV6-6
H1-7-A	CDRH1	1YY8	B	GFSLTNY	house mouse	IGHV2-2
H1-7-A	CDRH1	3V6F	C	GFTFSSY	house mouse	IGHV5-6-1
H1-7-A	CDRH1	1LK3	H	GYTFTDF	Norway rat	IGHV1S29
H1-7-A	CDRH1	3EYO	D	GFKFGDH	human	IGHV3-30
H1-7-A	CDRH1	3WKM	I	GFNIKDT	house mouse	IGHV14-3
H1-7-A	CDRH1	1ZA6	D	GYTFTDH	house mouse	IGHV1
H1-7-A	CDRH1	3TCL	A	GFIFENF	human	IGHV3-20
H1-7-A	CDRH1	1A7P	H	GFSLTGY	house mouse	IGHV2-6-7
H1-7-A	CDRH1	2G5B	F	GFTFIDN	house mouse	IGHV7-3
H1-7-A	CDRH1	1NMC	H	GYTFTNY	house mouse	IGHV1-12
H1-7-A	CDRH1	2FBJ	H	GFDFSKY	house mouse	IGHV4-1
H1-7-A	CDRH1	1BBD	H	GFNIKDI	house mouse	IGHV14-3
H1-7-A	CDRH1	4M43	H	GYTFTDY	house mouse	IGHV1-18
H1-7-A	CDRH1	1KFA	H	GFTFSSY	house mouse	IGHV5S9
H1-7-A	CDRH1	3MAC	H	GGTFNSY	human	IGHV1-69
H1-7-A	CDRH1	1C5D	B	GFPLTTN	Norway rat	IGHV2S8
H1-7-A	CDRH1	3KS0	K	GDSITSG	house mouse	IGHV3-8
H1-7-A	CDRH1	3PNW	H	GFNLSSS	human	IGHV3-66
H1-7-A	CDRH1	4H88	H	GYTFTDY	house mouse	IGHV1S127
H1-7-A	CDRH1	3MLV	H	GYNFLDS	human	IGHV5-51
H1-7-A	CDRH1	1BFV	H	GFTFNTY	house mouse	IGHV5-6-2
H1-7-A	CDRH1	1N7M	L	GYTFTSY	house mouse	IGHV1S36
H1-7-A	CDRH1	1XF4	B	GYTFTSY	house mouse	IGHV1-14
H1-7-A	CDRH1	4NRZ	A	GYNFASE	human	IGHV5-51
H1-7-A	CDRH1	1MRF	H	GYTFTSY	house mouse	IGHV1-50
H1-7-A	CDRH1	3UYR	H	GFTFSDY	house mouse	IGHV5-16
H1-7-A	CDRH1	4HH9	B	GFTFSNH	human	IGHV3-30
H1-7-A	CDRH1	1XGU	B	GDSVTSD	house mouse	IGHV3-8
H1-7-A	CDRH1	1Y0L	B	GFDFRRY	house mouse	IGHV4-1
H1-7-A	CDRH1	3KYM	B	GFTFSIY	human	IGHV3-23
H1-7-A	CDRH1	4DKF	I	GFSFTSY	human	IGHV3-23
H1-7-A	CDRH1	4GRW	G	GFTLDDY	llama	IGHV1S1
H1-7-A	CDRH1	3DUR	D	GFTFTDY	house mouse	IGHV7-3
H1-7-A	CDRH1	1NAK	H	GVSITSG	house mouse	IGHV3-8

H1-7-A	CDRH1	1CT8	B	GFTFSSS	house mouse	IGHV1-66
H1-7-A	CDRH1	3S35	H	GYIFTEY	house mouse	IGHV1-71
H1-7-A	CDRH1	2ZPK	I	GYTFTTA	house mouse	IGHV9-4
H1-7-A	CDRH1	3UO1	H	GFTFSDY	house mouse	IGHV5-16
H1-7-A	CDRH1	3R06	F	GFTFSGY	house mouse	IGHV5-17
H1-7-A	CDRH1	3UJT	I	GYTFTSS	house mouse	IGHV1S130
H1-7-A	CDRH1	2ORB	I	GFTFSHY	house mouse	IGHV5-6
H1-7-A	CDRH1	2B1H	H	GYSFSDY	human	IGHV5-51
H1-7-A	CDRH1	4BEL	E	GFTFSSA	llama	IGHV1S6
H1-7-A	CDRH1	1AD0	B	GFTFTDY	human	IGHV3-72
H1-7-A	CDRH1	3PNW	K	GFNLSSS	human	IGHV3-66
H1-7-A	CDRH1	3HNT	H	GYSFTNY	house mouse	IGHV1-5
H1-7-A	CDRH1	1KEG	H	GYSFTSF	house mouse	IGHV1-5
H1-7-A	CDRH1	1HIL	D	GFSFSSY	house mouse	IGHV5-6-1
H1-7-A	CDRH1	4NRX	D	GYNFASE	human	IGHV5-51
H1-7-A	CDRH1	2HRP	N	GFTFMRF	house mouse	IGHV5-17
H1-7-A	CDRH1	2OR9	H	GFTFSHY	house mouse	IGHV5-6
H1-7-A	CDRH1	1ZLS	H	NFRISAH	human	IGHV3-21
H1-7-A	CDRH1	3OAZ	H	NFRISAH	human	IGHV3-21
H1-7-A	CDRH1	1NMB	H	GYTFTNY	house mouse	IGHV1-12
H1-7-A	CDRH1	2F19	H	GYTFTSY	house mouse	IGHV1-58
H1-7-A	CDRH1	4KI5	E	GYTFSSY	house mouse	IGHV1-9
H1-7-A	CDRH1	3DV6	B	GFTFTDY	house mouse	IGHV7-3
H1-7-A	CDRH1	3H3P	I	GGSFSTY	human	IGHV1-69
H1-7-A	CDRH1	3UPC	I	GFTFSDE	human	IGHV3-23
H1-7-A	CDRH1	1XGY	H	GYTFTDY	house mouse	IGHV1-75
H1-7-A	CDRH1	1CFT	B	GYIFTDY	house mouse	IGHV1S52
H1-7-A	CDRH1	3O0R	H	GYSFTSY	house mouse	IGHV1-5
H1-7-A	CDRH1	4FQL	H	GFSFDEY	human	IGHV3-9
H1-7-A	CDRH1	3U9P	K	GFSLTSN	Norway rat	IGHV2S5
H1-7-A	CDRH1	3K2U	H	GFTINGT	human	IGHV3-66
H1-7-A	CDRH1	1NGZ	B	GYTFTSY	house mouse	IGHV1S36
H1-7-A	CDRH1	2R2E	B	GFTFTDY	house mouse	IGHV7-3
H1-7-A	CDRH1	4HIH	D	GFTFSRY	human	IGHV3-30
H1-7-A	CDRH1	4HGW	B	GFTFTDY	house mouse	IGHV7-3
H1-7-A	CDRH1	3QEH	G	GYTFVNF	house mouse	IGHV1-46
H1-7-A	CDRH1	1CLZ	H	GFTFSDY	house mouse	IGHV5-12
H1-7-A	CDRH1	3U0W	H	GFDFSRY	house mouse	IGHV4-1
H1-7-A	CDRH1	4JR9	H	GYSFTDY	house mouse	IGHV9-3
H1-7-A	CDRH1	1AR1	C	GFTFSSY	house mouse	IGHV5-12-2
H1-7-A	CDRH1	3OGO	E	GFPVNRY	Arabian camel	IGHV1S26
H1-7-A	CDRH1	3UPC	C	GFTFSDE	human	IGHV3-23
H1-7-A	CDRH1	3MA9	H	GGTFNSY	human	IGHV1-69
H1-7-A	CDRH1	1M71	B	GFTFSNY	house mouse	IGHV6-6
H1-7-A	CDRH1	4HCR	H	GYTFTSY	house mouse	IGHV1-18
H1-7-A	CDRH1	2NXZ	D	GDTFIRY	human	IGHV1-69
H1-7-A	CDRH1	1NJ9	H	GYSFTDY	house mouse	IGHV1S135
H1-7-A	CDRH1	3C6S	F	GLTFSNY	house mouse	IGHV6-3
H1-7-A	CDRH1	3UPC	A	GFTFSDE	human	IGHV3-23
H1-7-A	CDRH1	1MLC	B	GYTFSTY	house mouse	IGHV1-9

H1-7-A	CDRH1	4HS8	H	GYTFTNY	house mouse	IGHV3-23
H1-7-A	CDRH1	3K74	B	GFTFNNY	Arabian camel	IGHV1S23
H1-7-A	CDRH1	3EYO	B	GFKFGDH	human	IGHV3-30
H1-7-A	CDRH1	4LF3	E	GFTFSSY	house mouse	IGHV5-17
H1-7-A	CDRH1	1Y0L	H	GFDFRRY	house mouse	IGHV4-1
H1-7-A	CDRH1	3V0V	H	GFTFSDY	house mouse	IGHV7-3
H1-7-A	CDRH1	1ZLV	M	NFRISAH	human	IGHV3-21
H1-7-A	CDRH1	1EGJ	H	GYTFTDY	house mouse	IGHV1-26
H1-7-A	CDRH1	1A6T	D	GYSFSTY	house mouse	IGHV1-26
H1-7-A	CDRH1	3TNN	A	GFTFSTY	human	IGHV3-23
H1-7-A	CDRH1	2O5Z	H	GYMFTNY	house mouse	IGHV9-3-1
H1-7-A	CDRH1	1YEC	H	GYIFTSY	house mouse	IGHV1-76
H1-7-A	CDRH1	2H1P	H	GFTFSSY	house mouse	IGHV5-6-2
H1-7-A	CDRH1	2Q8A	H	GFKIKDT	house mouse	IGHV14-3
H1-7-A	CDRH1	1WCB	H	GFTFTDY	house mouse	IGHV7-3
H1-7-A	CDRH1	2YMX	H	GYSFTGY	house mouse	IGHV1-42
H1-7-A	CDRH1	1D6V	H	GYTFSSY	house mouse	IGHV1-9
H1-7-A	CDRH1	4J8R	D	GYAFTNY	house mouse	IGHV1-54
H1-7-A	CDRH1	3G6A	H	GFTFNSY	human	IGHV3-30
H1-7-A	CDRH1	4ISV	B	GYSFTTY	house mouse	IGHV9-3
H1-7-A	CDRH1	3UPC	D	GFTFSDE	human	IGHV3-23
H1-7-A	CDRH1	4JPI	H	GYTFTGY	human	IGHV1-2
H1-7-A	CDRH1	4HXA	H	GFTFSNY	house mouse	IGHV13-2
H1-7-A	CDRH1	1MCP	H	GFTFSDF	house mouse	IGHV7-1
H1-7-A	CDRH1	1CR9	H	GFNIKDY	house mouse	IGHV14-4
H1-7-A	CDRH1	2AEP	H	GFTFIDY	house mouse	IGHV7-3
H1-7-A	CDRH1	1XF4	H	GYTFTSY	house mouse	IGHV1-14
H1-7-A	CDRH1	1TET	H	GYTFTTY	house mouse	IGHV9-3
H1-7-A	CDRH1	1IC5	H	GDSITSD	house mouse	IGHV3-8
H1-7-A	CDRH1	3DGG	D	GYTFTSY	house mouse	IGHV1-14
H1-7-A	CDRH1	1FVE	D	GFNIKDT	human	IGHV3-66
H1-7-A	CDRH1	3B9V	A	GFNIKDT	human	IGHV3-66
H1-7-A	CDRH1	3TNN	C	GFTFSTY	human	IGHV3-23
H1-7-A	CDRH1	6FAB	H	GYTFTSN	house mouse	IGHV1-58
H1-7-A	CDRH1	2G2R	B	GYTFTAY	house mouse	IGHV1-19
H1-7-A	CDRH1	3Q6G	H	GGSISNN	human	IGHV4-59
H1-7-A	CDRH1	1SM3	H	GFTFSNY	house mouse	IGHV6-6
H1-7-A	CDRH1	1HH6	B	GYIFTDY	house mouse	IGHV1-15
H1-7-A	CDRH1	1P7K	H	GYTFTSY	house mouse	IGHV1-14
H1-7-A	CDRH1	3INU	H	GFTLINY	human	IGHV3-21
H1-7-A	CDRH1	3RU8	H	GYRFSNF	human	IGHV1-3
H1-7-A	CDRH1	4JFX	H	GFTFRKF	human	IGHV3-66
H1-7-A	CDRH1	4CAD	H	GYKFSSY	house mouse	IGHV1-9
H1-7-A	CDRH1	3HC0	A	GYTFTTY	human	IGHV1-69
H1-7-A	CDRH1	1F4X	H	GYVFSTY	house mouse	IGHV5-12-1
H1-7-A	CDRH1	1CFN	B	GYIFTDY	house mouse	IGHV1S52
H1-7-A	CDRH1	3NA9	H	GYSFTNY	human	IGHV5-10-1
H1-7-A	CDRH1	3UPC	B	GFTFSDE	human	IGHV3-23
H1-7-A	CDRH1	1PG7	I	GFNIKEY	human	IGHV3-66
H1-7-A	CDRH1	1BM3	H	GFTFSSY	house mouse	IGHV5S4

H1-7-A	CDRH1	3EOA	B	GYSFTGH	human	IGHV3-7
H1-7-A	CDRH1	3ZLQ	C	GFTFSSA	llama	IGHV1S6
H1-7-A	CDRH1	1NJ9	B	GYSFTDY	house mouse	IGHV1S135
H1-7-A	CDRH1	1FVC	B	GFNIKDT	human	IGHV3-66
H1-7-A	CDRH1	3NZH	H	GFTFSNY	house mouse	IGHV6-3
H1-7-A	CDRH1	1EJO	H	GFTFSSY	house mouse	IGHV5-6-4
H1-7-A	CDRH1	3ZDX	H	GFNIKDT	house mouse	IGHV14-3
H1-7-A	CDRH1	4ETQ	A	GYSFNFY	house mouse	IGHV1S126
H1-7-A	CDRH1	3O2W	H	GYMFTNY	house mouse	IGHV9-3-1
H1-7-A	CDRH1	1MNU	H	GYSFTSY	house mouse	IGHV1-5
H1-7-A	CDRH1	1WC7	B	GFTFTDY	house mouse	IGHV7-3
H1-7-A	CDRH1	4JHA	H	GGPLRNY	human	IGHV1-69
H1-7-A	CDRH1	3VW3	H	GYTFTNY	house mouse	IGHV1-5
H1-7-A	CDRH1	1G7J	B	GFSLTGY	house mouse	IGHV2-6-7
H1-7-A	CDRH1	1Q9W	D	GFTFTDY	house mouse	IGHV7-3
H1-7-A	CDRH1	4IJ3	C	GFSLTGY	house mouse	IGHV2-6-7
H1-7-A	CDRH1	3GJE	H	GFTFSTY	human	IGHV3-23
H1-7-A	CDRH1	1Q1J	H	GFTFSDV	human	IGHV3-15
H1-7-A	CDRH1	1PLG	H	GYTFTDY	house mouse	IGHV1-84
H1-7-A	CDRH1	3QNZ	B	GFTLSGS	human	IGHV3-73
H1-7-A	CDRH1	3OAZ	M	NFRISAH	human	IGHV3-21
H1-7-A	CDRH1	3SGE	H	GFSFNTN	house mouse	IGHV10S3
H1-7-A	CDRH1	4KVC	H	GFTFSSF	house mouse	IGHV5-17
H1-7-A	CDRH1	1XIW	H	GYSFTGY	house mouse	IGHV1-37
H1-7-A	CDRH1	3R06	B	GFTFSGY	house mouse	IGHV5-17
H1-7-A	CDRH1	1TZI	B	GFAIYDY	house mouse	IGHV5-17
H1-7-A	CDRH1	3GJE	B	GFTFSTY	human	IGHV3-23
H1-7-A	CDRH1	4K9E	H	GFNISVY	house mouse	IGHV3-66
H1-7-A	CDRH1	3T4Y	B	GFTFTDY	house mouse	IGHV7-3
H1-7-A	CDRH1	1UAC	H	GDSITSD	house mouse	IGHV3-8
H1-7-A	CDRH1	2VDL	H	GFNIKDT	house mouse	IGHV14-3
H1-7-A	CDRH1	1HI6	B	GYIFTDY	house mouse	IGHV1-15
H1-7-A	CDRH1	3QEH	E	GYTFVNF	house mouse	IGHV1-46
H1-7-A	CDRH1	4HWE	H	GYSFTSY	human	IGHV5-51
H1-7-A	CDRH1	2MPA	H	GYSFTSY	house mouse	IGHV1-5
H1-7-A	CDRH1	3OZ9	H	GYRFSSY	house mouse	IGHV1-9
H1-7-A	CDRH1	1FGV	H	GYTFTEY	human	IGHV3-74
H1-7-A	CDRH1	2OTU	H	GFTFRDY	house mouse	IGHV5-12
H1-7-A	CDRH1	2BMK	H	GFTFTDY	house mouse	IGHV7-3
H1-7-A	CDRH1	1J05	H	GFNIKDT	house mouse	IGHV14-3
H1-7-A	CDRH1	1FE8	J	GFSLTTY	house mouse	IGHV2-3
H1-7-A	CDRH1	3FO1	B	GFSLTNY	house mouse	IGHV2-9
H1-7-A	CDRH1	3U2S	A	GFDFSRQ	human	IGHV3-30
H1-7-A	CDRH1	2ZPK	H	GYTFTTA	house mouse	IGHV9-4
H1-7-A	CDRH1	4GXV	H	GFTFSSY	human	IGHV3-30
H1-7-A	CDRH1	3MUG	H	GFTFHKY	human	IGHV3-30
H1-7-A	CDRH1	1XGT	B	GDSVTSD	house mouse	IGHV3-8
H1-7-A	CDRH1	1YEI	H	GYIFTSY	house mouse	IGHV1-76
H1-7-A	CDRH1	3CFJ	B	GFDFRRY	house mouse	IGHV4-1
H1-7-A	CDRH1	3C6S	H	GLTFSNY	house mouse	IGHV6-3

H1-7-A	CDRH1	1CT8	D	GFTFSSS	house mouse	IGHV1-66
H1-7-A	CDRH1	1MRC	H	GYTFTSY	house mouse	IGHV1-50
H1-7-A	CDRH1	3I2C	H	GFNFNTY	house mouse	IGHV10-3
H1-7-A	CDRH1	1BFO	D	GFTFTDF	Norway rat	IGHV7S6
H1-7-A	CDRH1	3L95	B	GFTFSSY	human	IGHV3-74
H1-7-A	CDRH1	1NAK	I	GVSITSG	house mouse	IGHV3-8
H1-7-A	CDRH1	3OGO	H	GFPVNRY	Arabian camel	IGHV1S26
H1-7-A	CDRH1	3CFK	F	GFDFRRY	house mouse	IGHV4-1
H1-7-A	CDRH1	1QFU	H	GYTLTTY	house mouse	IGHV1-52
H1-7-A	CDRH1	1Q0X	H	GYTFSSY	house mouse	IGHV1-9
H1-7-A	CDRH1	1DQJ	B	GDSVTSD	house mouse	IGHV3-8
H1-7-A	CDRH1	2QQK	H	GFTISGY	human	IGHV3-66
H1-7-A	CDRH1	4G7Y	H	GFNVSSS	human	IGHV3-66
H1-7-A	CDRH1	3SGE	J	GFSFNTN	house mouse	IGHV10S3
H1-7-A	CDRH1	1CIC	B	GYTFTNY	house mouse	IGHV1S5
H1-7-A	CDRH1	4GW5	D	GFSLTNY	house mouse	IGHV2-2
H1-7-A	CDRH1	3MNW	B	GYTFTDY	house mouse	IGHV9-2-1
H1-7-A	CDRH1	4C83	C	GFTFRRF	house mouse	IGHV5-17
H1-7-A	CDRH1	2R1W	B	GFTFTDY	house mouse	IGHV7-3
H1-7-A	CDRH1	3RA7	I	GFTFSDY	house mouse	IGHV5-9-3
H1-7-A	CDRH1	1OAY	H	GYTFTSY	house mouse	IGHV1-72
H1-7-A	CDRH1	1RIV	H	GFTFSDA	house mouse	IGHV6-6
H1-7-A	CDRH1	2BDN	H	GLNIKDT	house mouse	IGHV14-3
H1-7-A	CDRH1	4NRX	H	GYNFASE	human	IGHV5-51
H1-7-A	CDRH1	2VYR	K	GFTFEEY	human	IGHV3-23
H1-7-A	CDRH1	3L1O	H	GFTFTDY	house mouse	IGHV7-3
H1-7-A	CDRH1	4FQJ	H	GYIFTES	human	IGHV1-18
H1-7-A	CDRH1	1KFA	I	GFTFSSY	house mouse	IGHV5S9
H1-7-A	CDRH1	1Y18	F	GFDFRRY	house mouse	IGHV4-1
H1-7-A	CDRH1	1L7I	H	GFTFTDY	human	IGHV3-66
H1-7-A	CDRH1	4HPO	H	GYRFTSY	human	IGHV5-51
H1-7-A	CDRH1	1JPT	H	GFNIKEY	human	IGHV3-66
H1-7-A	CDRH1	2PCP	B	GYTFTDY	house mouse	IGHV1-34
H1-7-A	CDRH1	1MFC	H	GYTFTNY	house mouse	IGHV1-5
H1-7-A	CDRH1	3CFB	H	GITFSRY	house mouse	IGHV5-9
H1-7-A	CDRH1	2I60	H	GDTFIRY	human	IGHV1-69
H1-7-A	CDRH1	1Q9O	D	GFTFTDY	house mouse	IGHV7-3
H1-7-A	CDRH1	1ZLV	H	NFRISAH	human	IGHV3-21
H1-7-A	CDRH1	3U4E	H	GFDFSRQ	human	IGHV3-30
H1-7-A	CDRH1	3LS4	H	GFTFNNY	house mouse	IGHV5-9-3
H1-7-A	CDRH1	43CA	B	GISLSRY	house mouse	IGHV2-6-4
H1-7-A	CDRH1	2R56	I	GFTFRHH	human	IGHV3-23
H1-7-A	CDRH1	3V6O	D	GFSLTDD	house mouse	IGHV2-6-5
H1-7-A	CDRH1	1DBJ	H	GYAFTNY	house mouse	IGHV9-3-1
H1-7-A	CDRH1	1UZ6	W	GFDFSGY	house mouse	IGHV4-1
H1-7-A	CDRH1	2W65	A	GYTFTDY	house mouse	IGHV9-2-1
H1-7-A	CDRH1	4BFB	E	GFTFSSA	llama	IGHV1S6
H1-7-A	CDRH1	1DBM	H	GYAFTNY	house mouse	IGHV9-3-1
H1-7-A	CDRH1	2HFF	H	GFTISSN	human	IGHV3-66
H1-7-A	CDRH1	3P9W	B	GFNIKDT	human	IGHV3-66

H1-7-A	CDRH1	1DQM	H	GDSVTSD	house mouse	IGHV3-8
H1-7-A	CDRH1	1IC7	H	GDSITSA	house mouse	IGHV3-8
H1-7-A	CDRH1	1T3F	B	GYIFTSS	human	IGHV1-69
H1-7-A	CDRH1	4D9L	I	GGNFNTY	human	IGHV1-69
H1-7-A	CDRH1	4AEI	I	GFTFSGY	house mouse	IGHV5-4
H1-7-A	CDRH1	8FAB	D	GFTFSNY	human	IGHV3-33
H1-7-A	CDRH1	2CK0	H	GFTFNTD	house mouse	IGHV10-1
H1-7-A	CDRH1	1UWX	M	GYTFTNF	house mouse	IGHV9-3-1
H1-7-A	CDRH1	1J05	B	GFNIKDT	house mouse	IGHV14-3
H1-7-A	CDRH1	4JY6	B	GASINDA	human	IGHV4-59
H1-7-A	CDRH1	4OD1	H	RFSFNRY	human	IGHV3-30
H1-7-A	CDRH1	1XF2	B	GYTFTSY	house mouse	IGHV1-14
H1-7-A	CDRH1	1TQC	B	GYTFTNY	house mouse	IGHV9-3-1
H1-7-A	CDRH1	1UYW	H	GYTFTEY	house mouse	IGHV1-26
H1-7-A	CDRH1	2FJF	D	GFTISDY	human	IGHV3-23
H1-7-A	CDRH1	2R2H	B	GFTFTDY	house mouse	IGHV7-3
H1-7-A	CDRH1	1HIL	B	GFSFSSY	house mouse	IGHV5-6-1
H1-7-A	CDRH1	3ZHD	B	GFTFSSY	human	IGHV3-23
H1-7-A	CDRH1	1DL7	H	GFSLTGY	house mouse	IGHV2-6-7
H1-7-A	CDRH1	3KR3	H	GFTFSNY	human	IGHV3-23
H1-7-A	CDRH1	1NCB	H	GYTFTNY	house mouse	IGHV9-3
H1-7-A	CDRH1	2YPV	H	GYSFSDY	house mouse	IGHV1-39
H1-7-A	CDRH1	1L7T	H	GFTFSRY	house mouse	IGHV5S9
H1-7-A	CDRH1	3HI5	H	GFTFSRY	human	IGHV3-23
H1-7-A	CDRH1	1PSK	H	GYTFTKY	house mouse	IGHV1-26
H1-7-A	CDRH1	1LO2	H	GFSFRNY	house mouse	IGHV5S4
H1-7-A	CDRH1	1GPO	I	GDSITSD	house mouse	IGHV3-8
H1-7-A	CDRH1	2GSG	B	GFTFRDY	house mouse	IGHV5-12
H1-7-A	CDRH1	2NY0	D	GDTFIRY	human	IGHV1-69
H1-7-A	CDRH1	3EHB	C	GFTFSSY	house mouse	IGHV5-12-2
H1-7-A	CDRH1	2C1O	B	GYSFTNY	house mouse	IGHV1-61
H1-7-A	CDRH1	3BKY	H	GYTFTSY	house mouse	IGHV1-12
H1-7-A	CDRH1	2FJF	H	GFTISDY	human	IGHV3-23
H1-7-A	CDRH1	3LH2	J	GGSFSTY	human	IGHV1-69
H1-7-A	CDRH1	3MLS	J	GYNFLDS	human	IGHV5-51
H1-7-A	CDRH1	2OQJ	B	NFRISAH	human	IGHV3-21
H1-7-A	CDRH1	1NMC	B	GYTFTNY	house mouse	IGHV1-12
H1-7-A	CDRH1	1IT9	H	GYTFTSY	house mouse	IGHV1-50
H1-7-A	CDRH1	2VDP	H	GFNIKDT	house mouse	IGHV14-3
H1-7-A	CDRH1	3NZ8	A	GYTFTTY	house mouse	IGHV1-85
H1-7-A	CDRH1	4FQI	H	GGTSNNY	human	IGHV1-69
H1-7-A	CDRH1	1T66	D	GFTFSDY	house mouse	IGHV6-7
H1-7-A	CDRH1	2NXY	D	GDTFIRY	human	IGHV1-69
H1-7-A	CDRH1	3O11	B	GYTFSTS	house mouse	IGHV1-9
H1-7-A	CDRH1	3RA7	H	GFTFSDY	house mouse	IGHV5-9-3
H1-7-A	CDRH1	1GPO	H	GDSITSD	house mouse	IGHV3-8
H1-7-A	CDRH1	2R8S	H	GFNLYSS	human	IGHV3-48
H1-7-A	CDRH1	3OKL	B	GFTFTDY	house mouse	IGHV7-3
H1-7-A	CDRH1	2OK0	H	GYTFTDY	house mouse	IGHV1-84
H1-7-A	CDRH1	3O2D	H	GYTFTSY	human	IGHV1-46

H1-7-A	CDRH1	3GJF	M	GFTFSTY	human	IGHV3-23
H1-7-A	CDRH1	1MFD	H	GYTFTNY	house mouse	IGHV1-5
H1-7-A	CDRH1	2YC1	A	GFTFDNY	human	IGHV3-9
H1-7-A	CDRH1	1OP3	M	NFRISAH	human	IGHV3-21
H1-7-A	CDRH1	1YYL	R	GDTFIRY	human	IGHV1-69
H1-7-A	CDRH1	4AG4	H	GYTFSIS	house mouse	IGHV1-61
H1-7-A	CDRH1	3KYM	L	GFTFSIY	human	IGHV3-23
H1-7-A	CDRH1	1FOR	H	GYAFSSF	house mouse	IGHV1-80
H1-7-A	CDRH1	3IJS	D	GFTFTDY	house mouse	IGHV7-3
H1-7-A	CDRH1	1D5I	H	GYTFSSY	house mouse	IGHV1-9
H1-7-A	CDRH1	2FX8	J	GGSFSTY	human	IGHV1-69
H1-7-A	CDRH1	1DEE	D	GFTFSGY	human	IGHV3-30
H1-7-A	CDRH1	2E27	H	GYTFTTY	house mouse	IGHV1-7
H1-7-A	CDRH1	1T66	H	GFTFSDY	house mouse	IGHV6-7
H1-7-A	CDRH1	2EH7	H	GYAFSSS	house mouse	IGHV1-82
H1-7-A	CDRH1	3CMO	H	GYTFTSY	house mouse	IGHV1-5
H1-7-A	CDRH1	2FJF	R	GFTISDY	human	IGHV3-23
H1-7-A	CDRH1	3HR5	J	GFTFSDY	house mouse	IGHV3-48
H1-7-A	CDRH1	4K23	H	GYSFTSY	house mouse	IGHV1-43
H1-7-A	CDRH1	1G7I	B	GFSLTGY	house mouse	IGHV2-6-7
H1-7-A	CDRH1	3UPC	G	GFTFSDE	human	IGHV3-23
H1-7-A	CDRH1	1N0X	K	GYRFSNF	human	IGHV1-3
H1-7-A	CDRH1	1H0D	B	GFTFSSY	house mouse	IGHV5-9
H1-7-A	CDRH1	3KYM	F	GFTFSIY	human	IGHV3-23
H1-7-A	CDRH1	1BZ7	B	GFTFSNF	house mouse	IGHV5-17
H1-7-A	CDRH1	1Q9L	D	GFTFTDY	house mouse	IGHV7-3
H1-7-A	CDRH1	3UC0	H	GGSISDF	human	IGHV4-59
H1-7-A	CDRH1	4J1U	F	GFSLTDY	house mouse	IGHV2-6-5
H1-7-A	CDRH1	1XIW	D	GYSFTGY	house mouse	IGHV1-37
H1-7-A	CDRH1	2W60	A	GYTFTDY	house mouse	IGHV9-2-1
H1-7-A	CDRH1	3FO0	H	GFSLTNY	house mouse	IGHV2-9
H1-7-A	CDRH1	3HR5	B	GFTFSDY	house mouse	IGHV3-48
H1-7-A	CDRH1	4NRZ	H	GYNFASE	human	IGHV5-51
H1-7-A	CDRH1	2AJ3	F	GASVNNY	human	IGHV4-59
H1-7-A	CDRH1	1FVD	D	GFNIKDT	human	IGHV3-66
H1-7-A	CDRH1	3R1G	H	GFTFLGY	human	IGHV3-23
H1-7-A	CDRH1	4D9R	E	GYTFTNY	human	IGHV7-4-1
H1-7-A	CDRH1	2JEL	H	GYTFTTY	house mouse	IGHV1-67
H1-7-A	CDRH1	1BJ1	K	GYTFTNY	human	IGHV7-4-1
H1-7-A	CDRH1	1AXS	B	GYTFSSF	house mouse	IGHV1-9
H1-7-A	CDRH1	1DVF	B	GFSLTGY	house mouse	IGHV2-6-7
H1-7-A	CDRH1	3GI8	H	GYTFTSY	house mouse	IGHV1-69
H1-7-A	CDRH1	1MJU	H	GYTFTNY	house mouse	IGHV1S61
H1-7-A	CDRH1	2OSL	A	GYTFTSY	house mouse	IGHV1-12
H1-7-A	CDRH1	1C1E	H	GYMFTNY	house mouse	IGHV9-1
H1-7-A	CDRH1	4HT1	H	GFDFSTY	rabbit	IGHV1S7
H1-7-A	CDRH1	3U2S	H	GFDFSRQ	human	IGHV3-30
H1-7-A	CDRH1	3V6O	C	GFSLTDD	house mouse	IGHV2-6-5
H1-7-A	CDRH1	2OTW	B	GFTFRDY	house mouse	IGHV5-12
H1-7-A	CDRH1	3CFK	D	GFDFRRY	house mouse	IGHV4-1

H1-7-A	CDRH1	1DSF	H	GFIFSDN	house mouse	IGHV5-4
H1-7-A	CDRH1	3MXV	H	GYTFIDE	house mouse	IGHV1-67
H1-7-A	CDRH1	3DGG	B	GYTFTSY	house mouse	IGHV1-14
H1-7-A	CDRH1	4EOW	H	GYSFSRY	human	IGHV7-4-1
H1-7-A	CDRH1	4HDI	H	GFTFSNY	house mouse	IGHV5-6-2
H1-7-A	CDRH1	1G7M	B	GFSLTGY	house mouse	IGHV2-6-7
H1-7-A	CDRH1	3QOS	B	GFTFSSY	human	IGHV3-23
H1-7-A	CDRH1	1WCB	B	GFTFTDY	house mouse	IGHV7-3
H1-7-A	CDRH1	1YEE	H	GYIFTSY	house mouse	IGHV1-76
H1-7-A	CDRH1	1MIM	H	GYSFTRY	human	IGHV1-5
H1-7-A	CDRH1	2C1O	H	GYSFTNY	house mouse	IGHV1-61
H1-7-A	CDRH1	1V7M	H	GFTFSDA	house mouse	IGHV6-6
H1-7-A	CDRH1	1FJ1	D	GYTFTDY	house mouse	IGHV9-2-1
H1-7-A	CDRH1	3KYM	P	GFTFSIY	human	IGHV3-23
H1-7-A	CDRH1	3QPQ	F	GYIFTTY	house mouse	IGHV1S81
H1-7-A	CDRH1	3GIZ	H	GFTFNDY	human	IGHV3-9
H1-7-A	CDRH1	4AMK	H	GYTFTSY	house mouse	IGHV1-12
H1-7-A	CDRH1	1XGQ	B	GDSVTSD	house mouse	IGHV3-8
H1-7-A	CDRH1	1AXT	H	GLTFSRF	house mouse	IGHV6-3
H1-7-A	CDRH1	1IQD	B	GYTLTEL	human	IGHV1-24
H1-7-A	CDRH1	2DQT	H	GFTFSNY	house mouse	IGHV5S9
H1-7-A	CDRH1	3HB3	C	GFTFSSY	house mouse	IGHV5-12-2
H1-7-A	CDRH1	3LHP	I	GGSFSTY	human	IGHV1-69
H1-7-A	CDRH1	4DGI	H	GYTFTDY	house mouse	IGHV1S127
H1-7-A	CDRH1	3VG0	H	GFSLTDD	house mouse	IGHV2-6-5
H1-7-A	CDRH1	3IF1	B	GFTFSDA	house mouse	IGHV6-6
H1-7-A	CDRH1	3I75	B	GYTFTDF	house mouse	IGHV1-15
H1-7-A	CDRH1	2IFF	H	GYTFSDY	house mouse	IGHV1-9
H1-7-A	CDRH1	2BRR	H	GYTFTNY	house mouse	IGHV9-3-1
H1-7-A	CDRH1	1MRD	H	GYTFTSY	house mouse	IGHV1-50
H1-7-A	CDRH1	1A5F	H	GFTIKDA	house mouse	IGHV14-3
H1-7-A	CDRH1	2HFG	H	GFTISSS	human	IGHV3-66
H1-7-A	CDRH1	2Q76	B	GYTFTTY	house mouse	IGHV1-9
H1-7-A	CDRH1	1A14	H	GYTFTNY	house mouse	IGHV1-12
H1-7-A	CDRH1	2IGF	H	GFTFSRC	house mouse	IGHV5-9-3
H1-7-A	CDRH1	3SKJ	H	GFTFSHY	human	IGHV3-23
H1-7-A	CDRH1	3QOS	H	GFTFSSY	human	IGHV3-23
H1-7-A	CDRH1	2FX9	H	GGSFSTY	human	IGHV1-69
H1-7-A	CDRH1	3T65	B	GFTFTDY	house mouse	IGHV7-3
H1-7-A	CDRH1	1MQK	H	GFTFSSY	house mouse	IGHV5-12-2
H1-7-A	CDRH1	1YYM	R	GDTFIRY	human	IGHV1-69
H1-7-A	CDRH1	2VXS	H	GFTFSSY	human	IGHV3-23
H1-7-A	CDRH1	3CFC	H	GITFSRY	house mouse	IGHV5-9
H1-7-A	CDRH1	3OAY	H	NFRISAH	human	IGHV3-21
H1-7-A	CDRH1	2PCP	D	GYTFTDY	house mouse	IGHV1-34
H1-7-A	CDRH1	3V4U	H	GFTFSDY	house mouse	IGHV5-16
H1-7-A	CDRH1	3BN9	F	GFTFSSY	human	IGHV3-23
H1-7-A	CDRH1	3ZKS	D	GFTFSSA	llama	IGHV1S6
H1-7-A	CDRH1	1I3G	H	GYTFTSY	house mouse	IGHV1-61
H1-7-A	CDRH1	1LO2	Y	GFSFRNY	house mouse	IGHV5S4

H1-7-A	CDRH1	1FL5	B	GFTFTDY	house mouse	IGHV3-72
H1-7-A	CDRH1	2VXU	I	GYSFTDY	house mouse	IGHV1S135
H1-7-A	CDRH1	3EYF	B	GFKFGDH	human	IGHV3-30
H1-7-A	CDRH1	4LLW	C	GFTFTDY	human	IGHV3-66
H1-7-A	CDRH1	4G3Y	H	GFIFSNH	house mouse	IGHV6-6
H1-7-A	CDRH1	3GGW	B	GLTFSNY	house mouse	IGHV6-3
H1-7-A	CDRH1	3MLS	I	GYNFLDS	human	IGHV5-51
H1-7-A	CDRH1	1G7L	B	GFSLTGY	house mouse	IGHV2-6-7
H1-7-A	CDRH1	3H42	H	GFTFSSY	human	IGHV3-21
H1-7-A	CDRH1	3OKM	B	GFTFTDY	house mouse	IGHV7-3
H1-7-A	CDRH1	3P9W	D	GFNIKDT	human	IGHV3-66
H1-7-A	CDRH1	1YY8	D	GFSLTNY	house mouse	IGHV2-2
H1-7-A	CDRH1	2G5B	H	GFTFIDN	house mouse	IGHV7-3
H1-7-A	CDRH1	2DQF	E	GDSITSD	house mouse	IGHV3-8
H1-7-A	CDRH1	3SE8	H	GYNFRDY	human	IGHV1-2
H1-7-A	CDRH1	3PHQ	B	GFTFIDY	house mouse	IGHV7-3
H1-7-A	CDRH1	2UYL	N	GYTFTSN	house mouse	IGHV1S61
H1-7-A	CDRH1	2XZQ	H	GYTFTSY	house mouse	IGHV1-72
H1-7-A	CDRH1	3EOT	H	GFTFSRY	house mouse	IGHV5-9
H1-7-A	CDRH1	4LKC	B	GFTFSSY	human	IGHV3-13
H1-7-A	CDRH1	3CFK	B	GFDFRRY	house mouse	IGHV4-1
H1-7-A	CDRH1	3ZE2	E	GFNIKDT	house mouse	IGHV14-3
H1-7-A	CDRH1	3LIZ	H	GFTFSSF	house mouse	IGHV5-6-3
H1-7-A	CDRH1	4F57	H	GFSFSDF	human	IGHV3-30
H1-7-A	CDRH1	1J1X	H	GDSITSD	house mouse	IGHV3-8
H1-7-A	CDRH1	3MLW	H	GYTFTDH	human	IGHV5-51
H1-7-A	CDRH1	1C5D	H	GFPLTTN	Norway rat	IGHV2S8
H1-7-A	CDRH1	2ZKH	H	GFTFSDA	house mouse	IGHV6-6
H1-7-A	CDRH1	2A6J	H	GYTFTSY	house mouse	IGHV1-58
H1-7-A	CDRH1	4HIH	B	GFTFSRY	human	IGHV3-30
H1-7-A	CDRH1	2UZI	H	GFTFSTF	human	IGHV3-48
H1-7-A	CDRH1	1I8I	B	GFTFRKF	house mouse	IGHV5-6
H1-7-A	CDRH1	3HNV	H	GYSFTNY	house mouse	IGHV1-5
H1-7-A	CDRH1	1V7M	I	GFTFSDA	house mouse	IGHV6-6
H1-7-A	CDRH1	3HR5	H	GFTFSDY	house mouse	IGHV3-48
H1-7-A	CDRH1	1BQL	H	GYTFSDY	house mouse	IGHV1-9
H1-7-A	CDRH1	1IGM	H	GFTFNIF	human	IGHV3-23
H1-7-A	CDRH1	2FX8	H	GGSFSTY	human	IGHV1-69
H1-7-A	CDRH1	3PNW	W	GFNLSSS	human	IGHV3-66
H1-7-A	CDRH1	3R06	D	GFTFSGY	house mouse	IGHV5-17
H1-7-A	CDRH1	1HEZ	B	GFTFSGY	human	IGHV3-30
H1-7-A	CDRH1	2V7N	B	GFTFRNS	human	IGHV3-30
H1-7-A	CDRH1	1RZ8	B	GDTFIRY	human	IGHV1-69
H1-7-A	CDRH1	3Q3G	H	GFNIKDI	house mouse	IGHV14-3
H1-7-A	CDRH1	3MLW	I	GYTFTDH	human	IGHV5-51
H1-7-A	CDRH1	1LO3	H	GFSFRNY	house mouse	IGHV5S4
H1-7-A	CDRH1	2AAB	H	GFTFSSF	house mouse	IGHV5-17
H1-7-A	CDRH1	2VYR	I	GFTFEEY	human	IGHV3-23
H1-7-A	CDRH1	1FN4	D	GFSLTSF	Norway rat	IGHV2S85
H1-7-A	CDRH1	4AEI	J	GFTFSGY	house mouse	IGHV5-4

H1-7-A	CDRH1	4DGY	H	GFTFNNY	human	IGHV3-33
H1-7-A	CDRH1	4K3E	I	GFSLSDK	sheep	IGHV1S3
H1-7-A	CDRH1	1CLY	H	GFTFSDY	house mouse	IGHV5-12
H1-7-A	CDRH1	2VDK	H	GFNIKDT	house mouse	IGHV14-3
H1-7-A	CDRH1	4LF3	B	GFTFSSY	house mouse	IGHV5-17
H1-7-A	CDRH1	3MLT	E	GYNFLDS	human	IGHV5-51
H1-7-A	CDRH1	1IKF	H	GFTFSDY	house mouse	IGHV5-12
H1-7-A	CDRH1	1OP3	H	NFRISAH	human	IGHV3-21
H1-7-A	CDRH1	4LSU	H	GYTFSDY	human	IGHV1-2
H1-7-A	CDRH1	1I8M	B	GYTFTSY	house mouse	IGHV1-14
H1-7-A	CDRH1	1KEL	H	GFTFTDY	house mouse	IGHV7-3
H1-7-A	CDRH1	2DQJ	H	GDSITSD	house mouse	IGHV3-8
H1-7-A	CDRH1	4HK0	C	GYTFTGY	human	IGHV1-2
H1-7-A	CDRH1	1MH5	B	GYTFTSN	house mouse	IGHV1S61
H1-7-A	CDRH1	2WUC	H	GFTINGT	human	IGHV3-66
H1-7-A	CDRH1	2OQJ	H	NFRISAH	human	IGHV3-21
H1-7-A	CDRH1	2VYR	E	GFTFEEY	human	IGHV3-23
H1-7-A	CDRH1	1RIU	H	GFTFSDA	house mouse	IGHV6-6
H1-7-A	CDRH1	4AEH	H	GYSFTSN	house mouse	IGHV1S61
H1-7-A	CDRH1	1OAQ	H	GYTFTSY	house mouse	IGHV1-72
H1-7-A	CDRH1	3HZK	B	GFTFTDY	house mouse	IGHV7-3
H1-7-A	CDRH1	1E4X	I	GFTFTNY	house mouse	IGHV1S81
H1-7-A	CDRH1	2ADF	H	GYTFINY	house mouse	IGHV9-3
H1-7-A	CDRH1	1CZ8	H	GYDFTHY	human	IGHV3-23
H1-7-A	CDRH1	1A0Q	H	GYTFTDH	house mouse	IGHV1S53
H1-7-A	CDRH1	1EMT	H	GYVFSSS	house mouse	IGHV1-82
H1-7-A	CDRH1	4AEI	H	GFTFSGY	house mouse	IGHV5-4
H1-7-A	CDRH1	3CFK	K	GFDFRRY	house mouse	IGHV4-1
H1-7-A	CDRH1	2DQH	H	GDSITSD	house mouse	IGHV3-8
H1-7-A	CDRH1	4HFW	H	GYSFTSY	human	IGHV1-46
H1-7-A	CDRH1	1NFD	F	GFTFSDF	hamster	IGHV6-6
H1-7-A	CDRH1	3A6C	H	GDSITSD	house mouse	IGHV3-8
H1-7-A	CDRH1	2VYR	H	GFTFEEY	human	IGHV3-23
H1-7-A	CDRH1	2NY1	D	GDTFIRY	human	IGHV1-69
H1-7-A	CDRH1	2CGR	H	GYTFSEY	house mouse	IGHV1-9
H1-7-A	CDRH1	1FDL	H	GFSLTGY	house mouse	IGHV2-6-7
H1-7-A	CDRH1	4HIJ	B	GFTFSRY	human	IGHV3-30
H1-7-A	CDRH1	2FJF	T	GFTISDY	human	IGHV3-23
H1-7-A	CDRH1	3G04	B	GYRFTSY	human	IGHV5-10-1
H1-7-A	CDRH1	1NL0	H	GFTFSTY	human	IGHV3-30
H1-7-A	CDRH1	3NCJ	H	GYSFTNY	human	IGHV5-10-1
H1-7-A	CDRH1	3QPX	H	GLTFSAN	Norway rat	IGHV5S47
H1-7-A	CDRH1	1T2Q	H	GFSLTSY	house mouse	IGHV2-2
H1-7-A	CDRH1	2C1P	B	GYSFTNY	house mouse	IGHV1-61
H1-7-A	CDRH1	4IOI	B	GFNIKDT	human	IGHV3-66
H1-7-A	CDRH1	2VXS	K	GFTFSSY	human	IGHV3-23
H1-7-A	CDRH1	4JFZ	H	GFTFRKF	human	IGHV3-66
H1-7-A	CDRH1	1BFO	H	GFTFTDF	Norway rat	IGHV7S6
H1-7-A	CDRH1	3QEH	C	GYTFVNF	house mouse	IGHV1-46
H1-7-A	CDRH1	1RUR	H	GYTFTNY	house mouse	IGHV1-63

H1-7-A	CDRH1	3HZM	B	GFTFTDY	house mouse	IGHV7-3
H1-7-A	CDRH1	1H3P	H	GFTFSSY	house mouse	IGHV5-9-4
H1-7-A	CDRH1	1FE8	H	GFSLTTY	house mouse	IGHV2-3
H1-7-A	CDRH1	3PP3	H	GYAFSYS	house mouse	IGHV1-69
H1-7-A	CDRH1	2G60	H	GYTFTAY	house mouse	IGHV1-4
H1-7-A	CDRH1	1DQQ	D	GDSVTSD	house mouse	IGHV3-8
H1-7-A	CDRH1	4C83	A	GFTFRRF	house mouse	IGHV5-17
H1-7-A	CDRH1	1YQV	H	GYTFSDY	house mouse	IGHV1-9
H1-7-A	CDRH1	3V0W	H	GFTFSDY	house mouse	IGHV7-3
H1-7-A	CDRH1	4GW1	B	GFSLTNY	house mouse	IGHV2-2
H1-7-A	CDRH1	1CE1	H	GFTFTDF	human	IGHV4-59
H1-7-A	CDRH1	1I9I	H	GFTFSTY	house mouse	IGHV5S9
H1-7-A	CDRH1	4JRE	B	GYSFTDY	house mouse	IGHV9-3
H1-7-A	CDRH1	3SKJ	I	GFTFSHY	human	IGHV3-23
H1-7-A	CDRH1	1A6V	H	GYTFTSY	house mouse	IGHV1-72
H1-7-A	CDRH1	4MA8	H	GYTFTDY	house mouse	IGHV1S127
H1-7-A	CDRH1	4CAD	K	GYKFSSY	house mouse	IGHV1-9
H1-7-A	CDRH1	3DV4	B	GFTFTDY	house mouse	IGHV7-3
H1-7-A	CDRH1	1S3K	H	GFTFSDY	house mouse	IGHV3-30
H1-7-A	CDRH1	1JN6	B	GYSFTTY	house mouse	IGHV1-5
H1-7-A	CDRH1	3Q3G	K	GFNIKDI	house mouse	IGHV14-3
H1-7-A	CDRH1	1TZG	I	GGSFSTY	human	IGHV1-69
H1-7-A	CDRH1	3BZ4	D	GLTFSNY	house mouse	IGHV6-3
H1-7-A	CDRH1	1PG7	Z	GYSFTGH	house mouse	IGHV1-37
H1-7-A	CDRH1	4I77	H	GFSLSAY	house mouse	IGHV2-70
H1-7-A	CDRH1	3INU	M	GFTLINY	human	IGHV3-21
H1-7-A	CDRH1	1SBS	H	GFTFSNY	house mouse	IGHV6-6
H1-7-A	CDRH1	4NC2	B	GLTFSRY	llama	IGHV1S3
H1-7-A	CDRH1	2FR4	H	GYTFTSY	house mouse	IGHV1-14
H1-7-A	CDRH1	4GW4	H	GYKISDH	human	IGHV1-2
H1-7-A	CDRH1	4GW5	B	GFSLTNY	house mouse	IGHV2-2
H1-7-A	CDRH1	1MLC	D	GYTFSTY	house mouse	IGHV1-9
H1-7-A	CDRH1	2Y5T	A	GYTFTSY	house mouse	IGHV1-61
H1-7-A	CDRH1	1JHL	H	GYTFISY	house mouse	IGHV1-61
H1-7-A	CDRH1	1BVK	B	GFSLTGY	human	IGHV4-59
H1-7-A	CDRH1	3LDB	C	GDSITSY	Norway rat	IGHV3S3
H1-7-A	CDRH1	4HFW	B	GYSFTSY	human	IGHV1-46
H1-7-A	CDRH1	4F3F	B	GYSFTGY	house mouse	IGHV1-37
H1-7-A	CDRH1	4LMQ	E	GFSLTVY	human	IGHV3-23
H1-7-A	CDRH1	3GHB	H	GFTFSDV	human	IGHV3-15
H1-7-A	CDRH1	1NBZ	B	GDSVTSD	house mouse	IGHV3-8
H1-7-A	CDRH1	1OAR	H	GYTFTSY	house mouse	IGHV1-72
H1-7-A	CDRH1	3PNW	B	GFNLSSS	human	IGHV3-66
H1-7-A	CDRH1	1XGR	B	GDSVTSD	house mouse	IGHV3-8
H1-7-A	CDRH1	3BT2	H	GYSFTNF	house mouse	IGHV1-66
H1-7-A	CDRH1	4HWB	H	GYSFTSY	human	IGHV5-51
H1-7-A	CDRH1	3CFK	P	GFDFRRY	house mouse	IGHV4-1
H1-7-A	CDRH1	4ENE	C	GFDYSRY	house mouse	IGHV4-1
H1-7-A	CDRH1	2DQU	H	GFTFSNY	house mouse	IGHV5S9
H1-7-A	CDRH1	1OAZ	J	GYTFTSY	house mouse	IGHV1-72

H1-7-A	CDRH1	4KUC	H	GYTFTSY	house mouse	IGHV1S61
H1-7-A	CDRH1	1A6U	H	GYTFTSY	house mouse	IGHV1-72
H1-7-A	CDRH1	3B9K	D	GFSLISD	Norway rat	IGHV2S5
H1-7-A	CDRH1	1A7N	H	GFSLTGY	house mouse	IGHV2-6-7
H1-7-A	CDRH1	1T4K	B	GFSLSDY	house mouse	IGHV2-6-5
H1-7-A	CDRH1	3CVI	H	GYTFTDY	house mouse	IGHV1-18
H1-7-A	CDRH1	3SY0	B	GFTFTDY	house mouse	IGHV7-3
H1-7-A	CDRH1	2FX9	I	GGSFSTY	human	IGHV1-69
H1-7-A	CDRH1	4I2X	B	GYSFTSY	human	IGHV1-66
H1-7-A	CDRH1	1DEE	F	GFTFSGY	human	IGHV3-30
H1-7-A	CDRH1	1JV5	B	GYNFTSY	human	IGHV1-8
H1-7-A	CDRH1	1P2C	E	GYTFTTY	house mouse	IGHV1-9
H1-7-A	CDRH1	4HK0	A	GYTFTGY	human	IGHV1-2
H1-7-A	CDRH1	3T2N	H	GYTFTDY	human	IGHV7-4-1
H1-7-A	CDRH1	1XF5	D	GYTFTDF	house mouse	IGHV9-2-1
H1-7-A	CDRH1	4HBC	H	GFSLNTY	rabbit	IGHV1S69
H1-7-A	CDRH1	3QNX	B	GFTLSGS	human	IGHV3-73
H1-7-A	CDRH1	2G5B	D	GFTFIDN	house mouse	IGHV7-3
H1-7-A	CDRH1	1UZ6	P	GFDFSGY	house mouse	IGHV4-1
H1-7-A	CDRH1	1VGE	H	GYSFTSY	human	IGHV1-3
H1-7-A	CDRH1	3DSF	H	GFTFNIY	house mouse	IGHV10-1
H1-7-A	CDRH1	2AI0	H	GFTFRNY	house mouse	IGHV5S4
H1-7-A	CDRH1	2FX7	H	GGSFSTY	human	IGHV1-69
H1-7-A	CDRH1	1I9J	H	GFTFSTY	house mouse	IGHV5S9
H1-7-A	CDRH1	3ZKM	C	GFSLTRY	house mouse	IGHV2-9
H1-7-A	CDRH1	2OZ4	H	GYTFSEF	house mouse	IGHV1-26
H1-7-A	CDRH1	3FO9	B	GLTFSRF	house mouse	IGHV6-3
H1-7-A	CDRH1	3UTZ	F	GFAFSTY	house mouse	IGHV5-9
H1-7-A	CDRH1	1FL6	H	GFTFTDY	house mouse	IGHV3-72
H1-7-A	CDRH1	2OTW	D	GFTFRDY	house mouse	IGHV5-12
H1-7-A	CDRH1	3NH7	J	GFTFSNY	human	IGHV3-48
H1-7-A	CDRH1	1FVC	D	GFNIKDT	human	IGHV3-66
H1-7-A	CDRH1	2RCS	H	GFNIKDT	house mouse	IGHV1-3
H1-7-A	CDRH1	1RIH	H	GYSFTSY	house mouse	IGHV1-7
H1-7-A	CDRH1	4K2U	I	GFNIKDN	house mouse	IGHV14-3
H1-7-A	CDRH1	4BFB	D	GFTFSSA	llama	IGHV1S6
H1-7-A	CDRH1	2OSL	H	GYTFTSY	house mouse	IGHV1-12
H1-7-A	CDRH1	4DCQ	B	GYTFTTY	house mouse	IGHV9-3
H1-7-A	CDRH1	1BFO	F	GFTFTDF	Norway rat	IGHV7S6
H1-7-A	CDRH1	2G5B	B	GFTFIDN	house mouse	IGHV7-3
H1-7-A	CDRH1	4D9Q	E	GYTFTNY	human	IGHV7-4-1
H1-7-A	CDRH1	3CFI	L	GFAFSGY	llama	IGHV1S6
H1-7-A	CDRH1	2VL5	A	GYTFTSY	house mouse	IGHV1-61
H1-7-A	CDRH1	2FJF	X	GFTISDY	human	IGHV3-23
H1-7-A	CDRH1	3MO1	B	GYTFTDY	house mouse	IGHV9-2-1
H1-7-A	CDRH1	1FL6	B	GFTFTDY	house mouse	IGHV3-72
H1-7-A	CDRH1	2NY3	D	GDTFIRY	human	IGHV1-69
H1-7-A	CDRH1	1Q9L	B	GFTFTDY	house mouse	IGHV7-3
H1-7-A	CDRH1	4FFY	H	GYTFTNW	house mouse	IGHV1-72
H1-7-A	CDRH1	2ORB	H	GFTFSHY	house mouse	IGHV5-6

H1-7-A	CDRH1	2I5Y	H	GDTFIRY	human	IGHV1-69
H1-7-A	CDRH1	1XF3	H	GYTFTSY	house mouse	IGHV1-14
H1-7-A	CDRH1	1OAR	J	GYTFTSY	house mouse	IGHV1-72
H1-7-A	CDRH1	3HR5	I	GFTFSDY	house mouse	IGHV3-48
H1-7-A	CDRH1	4GRW	E	GFTLDDY	llama	IGHV1S1
H1-7-A	CDRH1	1OHQ	B	GFRISDE	human	IGHV3-23
H1-7-A	CDRH1	3PP4	H	GYAFSYS	house mouse	IGHV1-69
H1-7-A	CDRH1	8FAB	B	GFTFSNY	human	IGHV3-33
H1-7-A	CDRH1	1ZLU	M	NFRISAH	human	IGHV3-21
H1-7-A	CDRH1	1BLN	B	GFTFSSY	house mouse	IGHV5-9
H1-7-A	CDRH1	4DQO	H	GFTFHKY	human	IGHV3-30
H1-7-A	CDRH1	1FAI	H	GYTFTSY	house mouse	IGHV1-58
H1-7-A	CDRH1	3U4E	A	GFDFSRQ	human	IGHV3-30
H1-7-A	CDRH1	1IGF	H	GFTFSRC	house mouse	IGHV5-9-3
H1-7-A	CDRH1	1UCB	H	GFTFSDY	house mouse	IGHV5-12
H1-7-A	CDRH1	3FCT	B	GYTFTSY	house mouse	IGHV1S36
H1-7-A	CDRH1	4HQQ	H	GYRFTSY	human	IGHV5-51
H1-7-A	CDRH1	4JDV	A	GYTFTGY	human	IGHV1-2
H1-7-A	CDRH1	2HRP	H	GFTFMRF	house mouse	IGHV5-17
H1-7-A	CDRH1	1JFQ	H	GYTFTSN	house mouse	IGHV1-58
H1-7-A	CDRH1	1NBY	B	GDSVTSD	house mouse	IGHV3-8
H1-7-A	CDRH1	3NIG	H	GFNIKDT	house mouse	IGHV14-3
H1-7-A	CDRH1	3IJH	B	GFTFTDY	house mouse	IGHV7-3
H1-7-A	CDRH1	3UJT	H	GYTFTSS	house mouse	IGHV1S130
H1-7-A	CDRH1	4MA7	H	GYTFTDY	house mouse	IGHV1S127
H1-7-A	CDRH1	2Q8B	H	GFKIKDT	house mouse	IGHV14-3
H1-7-A	CDRH1	4HIE	B	GFTFSRY	human	IGHV3-30
H1-7-A	CDRH1	3GHB	I	GFTFSDV	human	IGHV3-15
H1-7-A	CDRH1	2V7N	F	GFTFRNS	human	IGHV3-30
H1-7-A	CDRH1	3DUU	D	GFTFTDY	house mouse	IGHV7-3
H1-7-A	CDRH1	3HC3	H	GYTFTTY	human	IGHV1-3
H1-7-A	CDRH1	3MLT	H	GYNFLDS	human	IGHV5-51
H1-7-A	CDRH1	1AXS	H	GYTFSSF	house mouse	IGHV1-9
H1-7-A	CDRH1	1FNS	H	GFSLTDY	house mouse	IGHV2-6-7
H1-7-A	CDRH1	4LLW	A	GFTFTDY	human	IGHV3-66
H1-7-A	CDRH1	3QO1	B	GFTLSGS	human	IGHV3-73
H1-7-A	CDRH1	1P2C	B	GYTFTTY	house mouse	IGHV1-9
H1-7-A	CDRH1	1SY6	H	GYTFTRY	house mouse	IGHV1-4
H1-7-A	CDRH1	1EAP	B	GYISTDH	house mouse	IGHV1S53
H1-7-A	CDRH1	2VXS	J	GFTFSSY	human	IGHV3-23
H1-7-A	CDRH1	1N4X	H	NYAFTDY	house mouse	IGHV9-2-1
H1-7-A	CDRH1	2QSC	H	GFNFSSY	human	IGHV3-64
H1-7-A	CDRH1	4KTD	H	GGSISTN	rhesus monkey	IGHV4-2
H1-7-A	CDRH1	4FQC	H	GASISDS	human	IGHV4-59
H1-7-A	CDRH1	2Y6S	H	GYTFTDY	house mouse	IGHV1-77
H1-7-A	CDRH1	4BEL	D	GFTFSSA	llama	IGHV1S6
H1-7-A	CDRH1	3K1K	D	GFPVNRY	llama	IGHV1S1
H1-7-A	CDRH1	1UZ6	F	GFDFSGY	house mouse	IGHV4-1
H1-7-A	CDRH1	1IGC	H	GFTFSSF	house mouse	IGHV5-17
H1-7-A	CDRH1	3MLU	H	GYNFLDS	human	IGHV5-51

H1-7-A	CDRH1	1KIQ	B	GFSLTGY	house mouse	IGHV2-6-7
H1-7-A	CDRH1	1PZ5	B	GFTFSNY	house mouse	IGHV6-6
H1-7-A	CDRH1	4M61	D	GYSFTGY	house mouse	IGHV1-39
H1-7-A	CDRH1	3BN9	D	GFTFSSY	human	IGHV3-23
H1-7-A	CDRH1	1I8M	H	GYTFTSY	house mouse	IGHV1-14
H1-7-A	CDRH1	3QPQ	J	GYIFTTY	house mouse	IGHV1S81
H1-7-A	CDRH1	2UYL	Y	GYTFTSN	house mouse	IGHV1S61
H1-7-A	CDRH1	1KEM	H	GFTFTDY	house mouse	IGHV7-3
H1-7-A	CDRH1	2FJF	N	GFTISDY	human	IGHV3-23
H1-7-A	CDRH1	4L5F	H	GYSFIGY	house mouse	IGHV1-42
H1-7-A	CDRH1	1ZA6	F	GYTFTDH	house mouse	IGHV1
H1-7-A	CDRH1	3O6K	H	GFAFSSY	house mouse	IGHV5-9
H1-7-A	CDRH1	1YMH	D	GYTFTDF	house mouse	IGHV9-2-1
H1-7-A	CDRH1	4IMK	A	GYTFTGY	human	IGHV1-2
H1-7-A	CDRH1	4D9R	H	GYTFTNY	human	IGHV7-4-1
H1-7-A	CDRH1	1YEK	H	GYIFTSY	house mouse	IGHV1-76
H1-7-A	CDRH1	3OGO	F	GFPVNRY	Arabian camel	IGHV1S26
H1-7-A	CDRH1	4K2U	H	GFNIKDN	house mouse	IGHV14-3
H1-7-A	CDRH1	3GHE	H	GFTFNNY	human	IGHV3-7
H1-7-A	CDRH1	2V7H	H	GYTFSNY	house mouse	IGHV1-9
H1-7-A	CDRH1	3OKK	B	GFTFTDY	house mouse	IGHV7-3
H1-7-A	CDRH1	1XF2	H	GYTFTSY	house mouse	IGHV1-14
H1-7-A	CDRH1	4N9G	H	GGSFSSY	human	IGHV4-34
H1-7-A	CDRH1	3BDY	H	GFNIKDT	human	IGHV3-66
H1-7-A	CDRH1	1GC1	H	GDTFIRY	human	IGHV1-69
H1-7-A	CDRH1	2VYR	G	GFTFEEY	human	IGHV3-23
H1-7-A	CDRH1	3T3P	H	GFNIKDT	house mouse	IGHV14-3
H1-7-A	CDRH1	1P7K	B	GYTFTSY	house mouse	IGHV1-14
H1-7-A	CDRH1	4I2X	D	GYSFTSY	human	IGHV1-66
H1-7-A	CDRH1	1MFE	H	GYTFTNY	house mouse	IGHV1-5
H1-7-A	CDRH1	1NC4	D	GFSLTDY	house mouse	IGHV2-2
H1-7-A	CDRH1	3C6S	B	GLTFSNY	house mouse	IGHV6-3
H1-7-A	CDRH1	1MHP	H	GFTFSRY	human	IGHV3-23
H1-7-A	CDRH1	1M7I	B	GFTFSNY	house mouse	IGHV6-6
H1-7-A	CDRH1	1QBL	H	GFNIKDT	house mouse	IGHV14-3
H1-7-A	CDRH1	4DTG	H	GFTFSNY	house mouse	IGHV3-21
H1-7-A	CDRH1	1UYW	M	GYTFTEY	house mouse	IGHV1-26
H1-7-A	CDRH1	3EYV	H	GFTFSDY	house mouse	IGHV5-12
H1-7-A	CDRH1	2V17	H	GFTFTDY	house mouse	IGHV7-3
H1-7-A	CDRH1	3QYC	A	GVRLSAY	human	IGHV3-23
H1-7-A	CDRH1	3B9V	B	GFNIKDT	human	IGHV3-66
H1-7-A	CDRH1	1OM3	K	NFRISAH	human	IGHV3-21
H1-7-A	CDRH1	2H9G	H	GFSIGKS	human	IGHV3-66
H1-7-A	CDRH1	3I02	D	GFTFTDY	house mouse	IGHV7-3
H1-7-A	CDRH1	1HEZ	D	GFTFSGY	human	IGHV3-30
H1-7-A	CDRH1	4FAB	H	GFTFSDY	house mouse	IGHV6-7
H1-7-A	CDRH1	2AJ3	D	GASVNNY	human	IGHV4-59
H1-7-A	CDRH1	1MF2	H	GFTFMRF	house mouse	IGHV5-17
H1-7-A	CDRH1	1T2J	A	GFTFSNS	human	IGHV3-23
H1-7-A	CDRH1	4LLU	C	GFTFTDY	human	IGHV3-66

H1-7-A	CDRH1	1BOG	B	GYIFTDY	house mouse	IGHV1S52
H1-7-A	CDRH1	3EFD	H	GFNLSYS	human	IGHV3-66
H1-7-A	CDRH1	2VXT	H	GYSFTDY	house mouse	IGHV1S135
H1-7-A	CDRH1	3NH7	H	GFTFSNY	human	IGHV3-48
H1-7-A	CDRH1	4F33	H	GYSFTGY	house mouse	IGHV1-37
H1-7-A	CDRH1	4ALA	H	GYTFSSY	house mouse	IGHV1-55
H1-7-A	CDRH1	3FCT	D	GYTFTSY	house mouse	IGHV1S36
H1-7-A	CDRH1	1YNL	H	GYTFSFY	house mouse	IGHV1-9
H1-7-A	CDRH1	3OAU	H	NFRISAH	human	IGHV3-21
H1-7-A	CDRH1	3PHO	B	GFTFIDY	house mouse	IGHV7-3
H1-7-A	CDRH1	1MLB	B	GYTFSTY	house mouse	IGHV1-9
H1-7-A	CDRH1	3SO3	C	GFTFSSY	human	IGHV3-23
H1-7-A	CDRH1	2GCY	B	GFTFSDY	house mouse	IGHV5-4
H1-7-A	CDRH1	1E4X	H	GFTFTNY	house mouse	IGHV1S81
H1-7-A	CDRH1	1YYL	H	GDTFIRY	human	IGHV1-69
H1-7-A	CDRH1	3LD8	C	GDSITSY	Norway rat	IGHV3S3
H1-7-A	CDRH1	1YEH	H	GYIFTSY	house mouse	IGHV1-76
H1-7-A	CDRH1	3I02	B	GFTFTDY	house mouse	IGHV7-3
H1-7-A	CDRH1	1D5B	H	GYTFSSF	house mouse	IGHV1-9
H1-7-A	CDRH1	3CFJ	F	GFDFRRY	house mouse	IGHV4-1
H1-7-A	CDRH1	1CK0	H	GFTFNTD	house mouse	IGHV10S3
H1-7-A	CDRH1	1WEJ	H	GFNIKDT	house mouse	IGHV14-3
H1-7-A	CDRH1	1FRG	H	GFTFSSF	house mouse	IGHV5-6
H1-7-A	CDRH1	3DIF	D	GYSFTSY	house mouse	IGHV1-66
H1-7-A	CDRH1	3LHP	H	GGSFSTY	human	IGHV1-69
H1-7-A	CDRH1	1C08	B	GDSITSD	house mouse	IGHV3S1
H1-7-A	CDRH1	3DUS	D	GFTFTDY	house mouse	IGHV7-3
H1-7-A	CDRH1	2W65	C	GYTFTDY	house mouse	IGHV9-2-1
H1-7-A	CDRH1	3ZHD	A	GFTFSSY	human	IGHV3-23
H1-7-A	CDRH1	1Q9O	B	GFTFTDY	house mouse	IGHV7-3
H1-7-A	CDRH1	1YEF	H	GYIFTSY	house mouse	IGHV1-76
H1-7-A	CDRH1	1CFS	B	GYIFTDY	house mouse	IGHV1-15
H1-7-A	CDRH1	3IJS	B	GFTFTDY	house mouse	IGHV7-3
H1-7-A	CDRH1	1OPG	H	GFTFSSY	house mouse	IGHV5S4
H1-7-A	CDRH1	1XGY	I	GYTFTDY	house mouse	IGHV1-75
H1-7-A	CDRH1	1UB5	H	GITFSRY	house mouse	IGHV5-9
H1-7-A	CDRH1	2A6J	B	GYTFTSY	house mouse	IGHV1-58
H1-7-A	CDRH1	3C6S	D	GLTFSNY	house mouse	IGHV6-3
H1-7-A	CDRH1	2DLF	H	GFTFSDA	house mouse	IGHV6-6
H1-7-A	CDRH1	3SGD	H	GFSFNTN	house mouse	IGHV10S3
H1-7-A	CDRH1	3EYF	D	GFKFGDH	human	IGHV3-30
H1-7-A	CDRH1	3GGW	D	GLTFSNY	house mouse	IGHV6-3
H1-7-A	CDRH1	1VFB	B	GFSLTGY	house mouse	IGHV2-6-7
H1-7-A	CDRH1	3C2A	H	GFTFSDV	human	IGHV3-15
H1-7-A	CDRH1	3ZLQ	D	GFTFSSA	llama	IGHV1S6
H1-7-A	CDRH1	3IET	B	GFTFSDA	house mouse	IGHV6-6
H1-7-A	CDRH1	3QEH	A	GYTFVNF	house mouse	IGHV1-46
H1-7-A	CDRH1	2YBR	G	GFTFDNY	human	IGHV3-9
H1-7-A	CDRH1	3B9V	C	GFNIKDT	human	IGHV3-66
H1-7-A	CDRH1	2FJF	B	GFTISDY	human	IGHV3-23

H1-7-A	CDRH1	3U7W	H	GYEFLNC	human	IGHV1-2
H1-7-A	CDRH1	2VYR	L	GFTFEEY	human	IGHV3-23
H1-7-A	CDRH1	3LH2	K	GGSFSTY	human	IGHV1-69
H1-7-A	CDRH1	4HIJ	D	GFTFSRY	human	IGHV3-30
H1-7-A	CDRH1	3D85	B	GYTFTSN	house mouse	IGHV1-14
H1-7-A	CDRH1	3MUG	L	GFTFHKY	human	IGHV3-30
H1-7-A	CDRH1	3HZY	B	GFTFTDY	house mouse	IGHV7-3
H1-7-A	CDRH1	1YYM	H	GDTFIRY	human	IGHV1-69
H1-7-A	CDRH1	4NX3	B	GFNIKDT	house mouse	IGHV14-3
H1-7-A	CDRH1	1G7H	B	GFSLTGY	house mouse	IGHV2-6-7
H1-7-A	CDRH1	3LRS	C	GFTFHKY	human	IGHV3-30
H1-7-A	CDRH1	1MAM	H	GFTFTDY	house mouse	IGHV7-3
H1-7-A	CDRH1	4EBQ	H	GYSFNFY	house mouse	IGHV1S126
H1-7-A	CDRH1	4JDV	H	GYTFTGY	human	IGHV1-2
H1-7-A	CDRH1	3EYQ	D	GFTFSSY	human	IGHV3-30
H1-7-A	CDRH1	2G2R	H	GYTFTAY	house mouse	IGHV1-19
H1-7-A	CDRH1	2FB4	H	GFIFSSY	human	IGHV3-30
H1-7-A	CDRH1	1CIC	D	GFSLTGY	house mouse	IGHV2-6-7
H1-7-A	CDRH1	3NAA	H	GYSFTNY	human	IGHV5-10-1
H1-7-A	CDRH1	3IJH	D	GFTFTDY	house mouse	IGHV7-3
H1-7-A	CDRH1	2DQF	B	GDSITSD	house mouse	IGHV3-8
H1-7-A	CDRH1	1OAY	J	GYTFTSY	house mouse	IGHV1-72
H1-7-A	CDRH1	4JY4	B	GASISDS	human	IGHV4-59
H1-7-A	CDRH1	3NIF	H	GFNIKDT	house mouse	IGHV14-3
H1-7-A	CDRH1	1TZH	H	GFDIYDD	house mouse	IGHV5-17
H1-7-A	CDRH1	1ZEA	H	GYTFTTY	house mouse	IGHV9-3
H1-7-A	CDRH1	2HFF	B	GFTISSN	human	IGHV3-66
H1-7-A	CDRH1	4ERS	H	GFTFSSY	human	IGHV3-30
H1-7-A	CDRH1	2AI0	I	GFTFRNY	house mouse	IGHV5S4
H1-7-A	CDRH1	3KYM	J	GFTFSIY	human	IGHV3-23
H1-7-A	CDRH1	3N9G	H	GYTFTHY	human	IGHV1-46
H1-7-A	CDRH1	3KYM	H	GFTFSIY	human	IGHV3-23
H1-7-A	CDRH1	3EYV	B	GFTFSDY	house mouse	IGHV5-12
H1-7-A	CDRH1	1A7O	H	GFSLTGY	house mouse	IGHV2-6-7
H1-7-A	CDRH1	1F3D	K	GYSFIDY	house mouse	IGHV1S135
H1-7-A	CDRH1	1LK3	I	GYTFTDF	Norway rat	IGHV1S29
H1-7-A	CDRH1	2B2X	H	GFTFSRY	house mouse	IGHV5-9
H1-7-A	CDRH1	1BVK	E	GFSLTGY	human	IGHV4-59
H1-7-A	CDRH1	4JRE	H	GYSFTDY	house mouse	IGHV9-3
H1-7-A	CDRH1	3IU4	H	GFSLSRY	house mouse	IGHV2-6-4
H1-7-A	CDRH1	1Y18	D	GFDFRRY	house mouse	IGHV4-1
H1-7-A	CDRH1	1XF3	B	GYTFTSY	house mouse	IGHV1-14
H1-7-A	CDRH1	4DGV	H	GFTFNNY	human	IGHV3-33
H1-7-A	CDRH1	1Q72	H	GFTFSDA	house mouse	IGHV6-6
H1-7-A	CDRH1	3CFK	I	GFDFRRY	house mouse	IGHV4-1
H1-7-A	CDRH1	1RZ8	D	GDTFIRY	human	IGHV1-69
H1-7-A	CDRH1	3ZKM	H	GFSLTRY	house mouse	IGHV2-9
H1-7-A	CDRH1	1IL1	A	DFNIKDY	house mouse	IGHV14-4
H1-7-B	CDRH1	3MLX	H	GGSISGF	human	IGHV4-59
H1-7-B	CDRH1	3MLY	I	GGSISGF	human	IGHV4-59

H1-7-B	CDRH1	4JZO	A	GGTLSNY	human	IGHV1-69
H1-7-B	CDRH1	1JGU	H	GFTFSGY	house mouse	IGHV5S21
H1-7-B	CDRH1	3MLY	H	GGSISGF	human	IGHV4-59
H1-7-B	CDRH1	4FQQ	H	GGSISSY	human	IGHV4-59
H1-7-B	CDRH1	4NPY	B	GGSISSY	human	IGHV4-59
H1-7-B	CDRH1	3U30	C	GFTFSNT	human	IGHV3-23
H1-7-B	CDRH1	4JZO	D	GGTLSNY	human	IGHV1-69
H1-7-B	CDRH1	1JGV	H	GFTFSGY	house mouse	IGHV5S21
H1-7-B	CDRH1	4NPY	H	GGSISSY	human	IGHV4-59
H1-7-B	CDRH1	4FQQ	F	GGSISSY	human	IGHV4-59
H1-7-B	CDRH1	3Q1S	H	GGSMINY	human	IGHV4-59
H1-7-B	CDRH1	4FQQ	B	GGSISSY	human	IGHV4-59
H1-7-B	CDRH1	4JZO	C	GGTLSNY	human	IGHV1-69
H1-7-B	CDRH1	3U30	F	GFTFSNT	human	IGHV3-23
H1-7-B	CDRH1	4FQQ	D	GGSISSY	human	IGHV4-59
H1-7-B	CDRH1	3MLX	I	GGSISGF	human	IGHV4-59
H1-7-C	CDRH1	1OSP	H	GEPITSG	house mouse	IGHV3-8
H1-7-C	CDRH1	4KPH	I	GDSITSG	house mouse	IGHV3-8
H1-7-C	CDRH1	4KPH	H	GDSITSG	house mouse	IGHV3-8
H1-7-C	CDRH1	4LEX	H	GDSITSG	house mouse	IGHV3-8
H1-7-C	CDRH1	4G6A	C	GDSITSG	house mouse	IGHV4-30-4
H1-7-C	CDRH1	4GAY	H	GDSITSG	house mouse	IGHV3-8
H1-7-C	CDRH1	4GAG	H	GDSITSG	house mouse	IGHV3-8
H1-7-C	CDRH1	4HS6	B	GDSITSG	house mouse	IGHV4-59
H1-7-C	CDRH1	4G6A	H	GDSITSG	house mouse	IGHV4-30-4
H1-7-C	CDRH1	1DQD	H	GDSITSG	house mouse	IGHV3-8
H1-7-D	CDRH1	1BZQ	K	GYAYTYI	Arabian camel	IGHV1S45
H1-7-D	CDRH1	1BZQ	L	GYAYTYI	Arabian camel	IGHV1S45
H1-7-D	CDRH1	1BZQ	M	GYAYTYI	Arabian camel	IGHV1S45
H1-7-D	CDRH1	2P4A	D	GYPWTYI	Arabian camel	IGHV1S45
H1-7-D	CDRH1	3QSK	B	GYHHPYI	Arabian camel	IGHV1S45
H1-7-D	CDRH1	2P49	B	GYAYTYI	Arabian camel	IGHV1S45
H1-7-D	CDRH1	1BZQ	N	GYAYTYI	Arabian camel	IGHV1S45
H1-7-D	CDRH1	2P4A	B	GYPWTYI	Arabian camel	IGHV1S45
H1-7-E	CDRH1	4DK3	B	GRTSSLY	llama	IGHV1S1
H1-7-E	CDRH1	1SJX	A	GNIFRIN	llama	IGHV1S3
H1-7-E	CDRH1	4DK6	B	GRTSSLY	llama	IGHV1S1
H1-7-E	CDRH1	4DKA	B	GRTSSLY	llama	IGHV1S1
H1-7-E	CDRH1	4DKA	A	GRTSSLY	llama	IGHV1S1
H1-7-E	CDRH1	4DK6	A	GRTSSLY	llama	IGHV1S1
H1-7-E	CDRH1	4DK3	A	GRTSSLY	llama	IGHV1S1
H1-7-F	CDRH1	3QXW	A	RRSSRSW	llama	IGHV1S3
H1-7-F	CDRH1	4KI5	C	DYTFTDY	house mouse	IGHV9-2-1
H1-7-F	CDRH1	3QXW	B	RRSSRSW	llama	IGHV1S3
H1-7-F	CDRH1	4NBZ	B	ERTFSRY	llama	IGHV1S1
H1-7-F	CDRH1	4NBZ	D	ERTFSRY	llama	IGHV1S1
H1-7-F	CDRH1	4NC0	B	ERTFSRY	llama	IGHV1S1
H1-7-G	CDRH1	3EZJ	H	GSIFSIN	llama	IGHV1S3
H1-7-G	CDRH1	1MVF	A	GFTYSRK	Arabian camel	IGHV1S45
H1-7-G	CDRH1	3EZJ	D	GSIFSIN	llama	IGHV1S3

H1-7-G	CDRH1	4IDL	A	GNTASIR	llama	IGHV1S3
H1-7-G	CDRH1	3EZJ	B	GSIFSIN	llama	IGHV1S3
H1-7-G	CDRH1	3EZJ	F	GSIFSIN	llama	IGHV1S3
H1-8-A	CDRH1	3G5X	D	GYSITSDF	house mouse	IGHV3-2
H1-8-A	CDRH1	3G5V	B	GYSITSDF	house mouse	IGHV3-2
H1-8-A	CDRH1	1CF8	H	GYSITSGY	house mouse	IGHV3-2
H1-8-A	CDRH1	2Z91	C	GYSITSGY	house mouse	IGHV3-1
H1-8-A	CDRH1	2Z92	A	GYSITSGY	house mouse	IGHV3-1
H1-8-A	CDRH1	1C12	B	GYSITSDY	house mouse	IGHV3-2
H1-8-A	CDRH1	1F8T	H	GYSITSDY	house mouse	IGHV3-2
H1-8-A	CDRH1	3LEX	H	GYSITSDY	house mouse	IGHV3-2
H1-8-A	CDRH1	3CXH	J	GYSITSGY	house mouse	IGHV3-6
H1-8-A	CDRH1	1KCU	H	GYSITSDY	house mouse	IGHV3-2
H1-8-A	CDRH1	1KC5	H	GYSITSDY	house mouse	IGHV3-2
H1-8-A	CDRH1	1HQ4	B	GYSITSGY	house mouse	IGHV3-2
H1-8-A	CDRH1	3RVV	D	GYSITSDY	house mouse	IGHV3-2
H1-8-A	CDRH1	3LEX	A	GYSITSDY	house mouse	IGHV3-2
H1-8-A	CDRH1	4M5Y	H	GYSISSNY	human	IGHV4-38-2
H1-8-A	CDRH1	3CFD	B	GYSITSDY	house mouse	IGHV3-2
H1-8-A	CDRH1	3G5X	B	GYSITSDF	house mouse	IGHV3-2
H1-8-A	CDRH1	1F90	H	GYSITSDY	house mouse	IGHV3-2
H1-8-A	CDRH1	3CX5	J	GYSITSGY	house mouse	IGHV3-6
H1-8-A	CDRH1	1ORS	B	GYSITNNY	house mouse	IGHV3-2
H1-8-A	CDRH1	1KCS	H	SYSITSDY	house mouse	IGHV3-2
H1-8-A	CDRH1	4LKX	A	GYSITSDY	house mouse	IGHV4-38-2
H1-8-A	CDRH1	1KCV	H	SYSITSDY	house mouse	IGHV3-2
H1-8-A	CDRH1	3RVW	D	GYSITSDY	house mouse	IGHV3-2
H1-8-A	CDRH1	3QG7	H	GYSITSNY	house mouse	IGHV3-2
H1-8-A	CDRH1	3CFD	H	GYSITSDY	house mouse	IGHV3-2
H1-8-A	CDRH1	3CXH	U	GYSITSGY	house mouse	IGHV3-6
H1-8-A	CDRH1	1AY1	H	GYSITSDY	house mouse	IGHV3-2
H1-8-A	CDRH1	1HQ4	D	GYSITSGY	house mouse	IGHV3-2
H1-8-A	CDRH1	3G5Y	B	GYSITSDY	house mouse	IGHV3-2
H1-8-A	CDRH1	3RVT	D	GYSITSDY	house mouse	IGHV3-2
H1-8-A	CDRH1	3LEY	H	GYLITTDY	house mouse	IGHV3-2
H1-8-A	CDRH1	3RVX	D	GYSITSDY	house mouse	IGHV3-2
H1-8-A	CDRH1	3G5Z	B	GYSITSDF	house mouse	IGHV3-2
H1-8-A	CDRH1	4M5Y	I	GYSISSNY	human	IGHV4-38-2
H1-8-A	CDRH1	1S5I	H	GYSITSDY	house mouse	IGHV3-2
H1-8-A	CDRH1	2Z91	A	GYSITSGY	house mouse	IGHV3-1
H1-8-B	CDRH1	1ND0	B	GYSITSDF	house mouse	IGHV3-2
H1-8-B	CDRH1	1RUP	H	GYSITSDF	house mouse	IGHV3-2
H1-8-B	CDRH1	1RUM	H	GYSITSDF	house mouse	IGHV3-2
H1-8-B	CDRH1	3QG6	B	GYSITSNY	house mouse	IGHV3-2
H1-8-B	CDRH1	1RUL	H	GYSITSDF	house mouse	IGHV3-2
H1-8-B	CDRH1	2AJU	H	GYSITTNY	house mouse	IGHV3-2
H1-8-B	CDRH1	1RUA	H	GYSITSDF	house mouse	IGHV3-2
H1-8-B	CDRH1	1ND0	F	GYSITSDF	house mouse	IGHV3-2
H1-8-B	CDRH1	1RU9	H	GYSITSDF	house mouse	IGHV3-2
H1-8-B	CDRH1	1NCW	H	GYSITSDF	house mouse	IGHV3-2

H1-8-B	CDRH1	3QG6	H	GYSITSNY	house mouse	IGHV3-2
H1-8-B	CDRH1	1ND0	H	GYSITSDF	house mouse	IGHV3-2
H1-8-B	CDRH1	1ND0	D	GYSITSDF	house mouse	IGHV3-2
H1-8-B	CDRH1	1RUK	H	GYSITSDF	house mouse	IGHV3-2
H1-8-C	CDRH1	3F58	H	GYSITSGY	house mouse	IGHV3-1
H1-8-C	CDRH1	1F58	H	GYSITSGY	house mouse	IGHV3-1
H1-8-C	CDRH1	2F58	H	GYSITSGY	house mouse	IGHV3-1
H1-8-C	CDRH1	2XZC	H	GYSISSGY	human	IGHV4-38-2
H1-8-C	CDRH1	2XA8	H	GYSITSGY	house mouse	IGHV3-66
H1-8-C	CDRH1	3ZL4	H	GYSISSGY	human	IGHV4-38-2
H1-8-C	CDRH1	2XZA	H	GYSISSGY	human	IGHV4-38-2
H1-9-A	CDRH1	2IPU	H	GFSLSTSGM	house mouse	IGHV8-12
H1-9-A	CDRH1	3IFN	H	GFSLSTSGM	house mouse	IGHV8-12
H1-9-A	CDRH1	3MOB	H	GFSLSDFGV	human	IGHV2-5
H1-9-A	CDRH1	3IDN	B	GFSLSDFGV	human	IGHV2-5
H1-9-A	CDRH1	3QHF	H	GFSLSTSGM	human	IGHV2-70
H1-9-A	CDRH1	1U8M	B	GFSLSDFGV	human	IGHV2-5
H1-9-A	CDRH1	3IDM	B	GFSLSDFGV	human	IGHV2-5
H1-9-A	CDRH1	1U8I	B	GFSLSDFGV	human	IGHV2-5
H1-9-A	CDRH1	2IQA	H	GFSLRTSGM	house mouse	IGHV8-12
H1-9-A	CDRH1	2VQ1	F	GFSLSSSAM	house mouse	IGHV8-8
H1-9-A	CDRH1	1R0A	H	GFSLSTSGI	house mouse	IGHV8-12
H1-9-A	CDRH1	3MOD	H	GFSLSDFGV	human	IGHV2-5
H1-9-A	CDRH1	3IFO	A	GFSLSTSGM	house mouse	IGHV8-12
H1-9-A	CDRH1	1U95	B	GFSLSDFGV	human	IGHV2-5
H1-9-A	CDRH1	2IQA	B	GFSLRTSGM	house mouse	IGHV8-12
H1-9-A	CDRH1	1U8Q	B	GFSLSDFGV	human	IGHV2-5
H1-9-A	CDRH1	1U8K	B	GFSLSDFGV	human	IGHV2-5
H1-9-A	CDRH1	2P8P	B	GFSLSDFGV	human	IGHV2-5
H1-9-A	CDRH1	1U8J	B	GFSLSDFGV	human	IGHV2-5
H1-9-A	CDRH1	3EYU	H	GFSLSTSGM	house mouse	IGHV8-12
H1-9-A	CDRH1	2IPT	H	GFSLSTSGM	house mouse	IGHV8-12
H1-9-A	CDRH1	1TJG	H	GFSLSDFGV	human	IGHV2-5
H1-9-A	CDRH1	1ETZ	H	GFSLSTSGM	house mouse	IGHV8-5
H1-9-A	CDRH1	1GGC	H	GFSLSTYGM	house mouse	IGHV8-12
H1-9-A	CDRH1	2PW2	B	GFSLSDFGV	human	IGHV2-5
H1-9-A	CDRH1	3MBX	H	GFSLSTYGM	house mouse	IGHV8-8
H1-9-A	CDRH1	4G6K	H	GFSLSTSGM	human	IGHV8-12
H1-9-A	CDRH1	4JLR	H	GFSLSTAGM	human	IGHV2-70
H1-9-A	CDRH1	2F5A	H	GFSLSDFGV	human	IGHV2-5
H1-9-A	CDRH1	1TJI	H	GFSLSDFGV	human	IGHV2-5
H1-9-A	CDRH1	3RKD	D	GFSLSTSGM	house mouse	IGHV8-8
H1-9-A	CDRH1	3L7F	H	GFSLSTYGM	human	IGHV2-5
H1-9-A	CDRH1	1TJH	H	GFSLSDFGV	human	IGHV2-5
H1-9-A	CDRH1	3QQ9	D	GFSLSTSGM	house mouse	IGHV8-12
H1-9-A	CDRH1	2HWZ	H	GFSLSTSGM	house mouse	IGHV2-70
H1-9-A	CDRH1	3L7F	B	GFSLSTYGM	human	IGHV2-5
H1-9-A	CDRH1	2R0Z	H	GFSLSTSGM	house mouse	IGHV8-12
H1-9-A	CDRH1	3D0L	B	GFSLSDFGV	human	IGHV2-5
H1-9-A	CDRH1	3MCK	B	GFSLSTSGM	house mouse	IGHV8-12

H1-9-A	CDRH1	1GGB	H	GFSLSTYGM	house mouse	IGHV8-12
H1-9-A	CDRH1	3IDI	B	GFSLSDFGV	human	IGHV2-5
H1-9-A	CDRH1	3QQ9	H	GFSLSTSGM	house mouse	IGHV8-12
H1-9-A	CDRH1	1U8H	B	GFSLSDFGV	human	IGHV2-5
H1-9-A	CDRH1	3O41	A	GFSLSTSGM	house mouse	IGHV8-12
H1-9-A	CDRH1	3L5Y	H	GFSLSTYGV	house mouse	IGHV2-5
H1-9-A	CDRH1	3L5X	H	GFSLSTYGM	human	IGHV2-5
H1-9-A	CDRH1	2VQ1	B	GFSLSSSAM	house mouse	IGHV8-8
H1-9-A	CDRH1	1U91	B	GFSLSDFGV	human	IGHV2-5
H1-9-A	CDRH1	2P8L	B	GFSLSDFGV	human	IGHV2-5
H1-9-A	CDRH1	3RKD	H	GFSLSTSGM	house mouse	IGHV8-8
H1-9-A	CDRH1	2F5B	H	GFSLSDFGV	human	IGHV2-5
H1-9-A	CDRH1	3L5W	B	GFSLSTYGM	house mouse	IGHV8-8
H1-9-A	CDRH1	3LEV	H	GFSLSDFGV	human	IGHV2-5
H1-9-A	CDRH1	3MOA	H	GFSLSDFGV	human	IGHV2-5
H1-9-A	CDRH1	3QRG	H	GFSLSTSGM	house mouse	IGHV2-5
H1-9-A	CDRH1	1GGI	H	GFSLSTYGM	house mouse	IGHV8-12
H1-9-A	CDRH1	2PR4	H	GFSLSDFGV	human	IGHV2-5
H1-9-A	CDRH1	2IPU	G	GFSLSTSGM	house mouse	IGHV8-12
H1-9-A	CDRH1	3IFO	H	GFSLSTSGM	house mouse	IGHV8-12
H1-9-A	CDRH1	3L7F	E	GFSLSTYGM	human	IGHV2-5
H1-9-A	CDRH1	3QWO	A	GFSLSTAGM	human	IGHV2-70
H1-9-A	CDRH1	2IQ9	H	GFSLRTSGM	house mouse	IGHV8-12
H1-9-A	CDRH1	1U8L	B	GFSLSDFGV	human	IGHV2-5
H1-9-A	CDRH1	3L7E	H	GFSLSTYGM	house mouse	IGHV8-8
H1-9-A	CDRH1	3IDG	B	GFSLSDFGV	human	IGHV2-5
H1-9-A	CDRH1	1ETZ	B	GFSLSTSGM	house mouse	IGHV8-5
H1-9-A	CDRH1	3IFL	H	GFSLSTSGM	house mouse	IGHV8-12
H1-9-A	CDRH1	3IDJ	B	GFSLSDFGV	human	IGHV2-5
H1-9-A	CDRH1	1JRH	H	GFSLTTYGM	house mouse	IGHV8-12
H1-9-A	CDRH1	1U92	B	GFSLSDFGV	human	IGHV2-5
H1-9-A	CDRH1	2J88	H	GFSLSTSGM	house mouse	IGHV8-12
H1-9-A	CDRH1	3O41	H	GFSLSTSGM	house mouse	IGHV8-12
H1-9-A	CDRH1	2R0W	H	GFSLRTSGM	house mouse	IGHV8-12
H1-9-A	CDRH1	3MCK	H	GFSLSTSGM	house mouse	IGHV8-12
H1-9-A	CDRH1	3L7E	B	GFSLSTYGM	house mouse	IGHV8-8
H1-9-A	CDRH1	2PW1	B	GFSLSDFGV	human	IGHV2-5
H1-9-A	CDRH1	4G6M	H	GFSLSTSGM	house mouse	IGHV8-12
H1-9-A	CDRH1	3QWO	H	GFSLSTAGM	human	IGHV2-70
H1-9-A	CDRH1	1U93	B	GFSLSDFGV	human	IGHV2-5
H1-9-A	CDRH1	3L5W	H	GFSLSTYGM	house mouse	IGHV8-8
H1-9-A	CDRH1	3D0V	B	GFSLSDFGV	human	IGHV2-5
H1-9-A	CDRH1	1U8N	B	GFSLSDFGV	human	IGHV2-5
H1-9-A	CDRH1	2P8M	B	GFSLSDFGV	human	IGHV2-5
H1-9-A	CDRH1	1GGI	J	GFSLSTYGM	house mouse	IGHV8-12
H1-9-A	CDRH1	3DRQ	B	GFSLSDFGV	human	IGHV2-5
H1-9-A	CDRH1	3EYS	H	GFSLSTSGM	house mouse	IGHV8-12
H1-9-B	CDRH1	3BAE	H	GFSIRTSKV	house mouse	IGHV8-12
H1-9-B	CDRH1	4F37	F	GFSLRTSRV	house mouse	IGHV8-12
H1-9-B	CDRH1	3BKC	H	GFSIRTSKV	house mouse	IGHV8-12

H1-9-B	CDRH1	3BKJ	H	GFSIRTSKV	house mouse	IGHV8-12
H1-9-B	CDRH1	3BKM	H	GFSIRTSKV	house mouse	IGHV8-12
Unclustered	CDRH1	4LLV	A	GGSFSTY	human	IGHV1-69
Unclustered	CDRH1	3QXW	E	RRSSRSW	llama	IGHV1S3
Unclustered	CDRH1	3EO0	B	GYTFSSN	human	IGHV1-69
Unclustered	CDRH1	4JN2	H	GFSLTSY	house mouse	IGHV2-9
Unclustered	CDRH1	1YZZ	A	GSTYSPC	Arabian camel	IGHV1S17
Unclustered	CDRH1	4B5E	A	GRSFSRD	llama	IGHV1S3
Unclustered	CDRH1	4I1N	B	ERLTVDY	llama	IGHV1S1
Unclustered	CDRH1	4KRM	F	GRTSRSY	llama	IGHV1S1
Unclustered	CDRH1	1UM5	H	EYTLTSY	house mouse	IGHV1-14
Unclustered	CDRH1	4NZR	H	GDSIRGGEWGDKDY	human	IGHV4-39
Unclustered	CDRH1	3QXU	D	RRSSRSW	llama	IGHV1S5
Unclustered	CDRH1	2QJD	B	DKN	channel catfish	NITR11
Unclustered	CDRH1	3SE9	H	EDIFERTE	human	IGHV1-2
Unclustered	CDRH1	3STL	A	GYTFTSD	house mouse	IGHV1S81
Unclustered	CDRH1	4NBY	C	GRTFSMD	llama	IGHV1S3
Unclustered	CDRH1	1HKL	H	GFNIKDT	house mouse	IGHV14-3
Unclustered	CDRH1	1GHF	H	YTFTDY	house mouse	IGHV9-3
Unclustered	CDRH1	2IH1	A	GYTFTSD	house mouse	IGHV1S81
Unclustered	CDRH1	2CMR	H	GDTFSSY	human	IGHV1-69
Unclustered	CDRH1	2YK1	H	GGS	human	IGHV4-39
Unclustered	CDRH1	3OR6	A	GYTFTSD	house mouse	IGHV1S81
Unclustered	CDRH1	4JO4	H	GFSFTNNY	rabbit	IGHV1S40
Unclustered	CDRH1	3QXU	A	RRSSRSW	llama	IGHV1S5
Unclustered	CDRH1	4MSW	A	GYTFTSD	house mouse	IGHV1S81
Unclustered	CDRH1	3B2U	N	GGSISSGDY	human	IGHV4-30-4
Unclustered	CDRH1	2NLJ	B	GYTFTSD	house mouse	IGHV1S81
Unclustered	CDRH1	3TPK	A	GYTFSQE	Arabian camel	IGHV1S50
Unclustered	CDRH1	3U0T	D	GYYTEAY	house mouse	IGHV1-46
Unclustered	CDRH1	2AJV	H	GYSITTNY	house mouse	IGHV3-2
Unclustered	CDRH1	3ZKX	C	GFTFSRA	llama	IGHV1S5
Unclustered	CDRH1	2HKF	H	GSTLNNY	house mouse	IGHV10-1
Unclustered	CDRH1	2HVJ	A	GYTFTSD	house mouse	IGHV1S81
Unclustered	CDRH1	1IGI	H	GYIFTDF	house mouse	IGHV1-34
Unclustered	CDRH1	3FN0	H	GGSMINY	human	IGHV4-59
Unclustered	CDRH1	2W0F	A	GYTFTSD	house mouse	IGHV1S81
Unclustered	CDRH1	4K3D	H	GFSLSDK	sheep	IGHV1S2
Unclustered	CDRH1	3QXW	D	RRSSRSW	llama	IGHV1S3
Unclustered	CDRH1	2QTE	D	KVTNKD	channel catfish	NITR11
Unclustered	CDRH1	3U0T	B	GYYTEAY	house mouse	IGHV1-46
Unclustered	CDRH1	4LLV	H	GGSFSTY	human	IGHV1-69
Unclustered	CDRH1	2JB5	H	GGTFSNY	human	IGHV1-69
Unclustered	CDRH1	3R0M	B	GRISSSY	human	IGHV3-23
Unclustered	CDRH1	3QCU	I	GYGFINY	house mouse	IGHV5-51
Unclustered	CDRH1	4LAR	H	GDSVTSG	house mouse	IGHV3-8
Unclustered	CDRH1	2BOB	A	GYTFTSD	house mouse	IGHV1S81
Unclustered	CDRH1	2XV6	D	GSFFMSN	alpaca	IGHV
Unclustered	CDRH1	3QXT	B	RRSSRSW	Arabian camel	IGHV1S72
Unclustered	CDRH1	1I7Z	B	GYSFTNY	house mouse	IGHV9-3-1

Unclustered	CDRH1	3B2U	W	GGSISSGDY	human	IGHV4-30-4
Unclustered	CDRH1	4KDT	A	GYTDSRY	Arabian camel	IGHV1S47
Unclustered	CDRH1	4KML	B	GRTFSSY	llama	IGHV1S1
Unclustered	CDRH1	3W9D	C	GGTLRTY	human	IGHV1-69
Unclustered	CDRH1	3QXV	B	RRSSRSW	llama	IGHV1S3
Unclustered	CDRH1	2X89	C	GYTDSRY	Arabian camel	IGHV1S47
Unclustered	CDRH1	1SHM	A	GATGSTY	llama	IGHV1S3
Unclustered	CDRH1	4FQH	H	GGTSNNY	human	IGHV1-69
Unclustered	CDRH1	1A4J	B	GYTFTNY	house mouse	IGHV9-3-1
Unclustered	CDRH1	2AJX	H	GYSITTNY	house mouse	IGHV3-2
Unclustered	CDRH1	2BJM	H	GYTFTSY	house mouse	IGHV1-72
Unclustered	CDRH1	3GKW	H	GYTFTNY	house mouse	IGHV1-69
Unclustered	CDRH1	4KRM	B	GRTSRSY	llama	IGHV1S1
Unclustered	CDRH1	1R3L	B	GYTFTSD	house mouse	IGHV1S81
Unclustered	CDRH1	4GRW	I	GRTFSWS	llama	IGHV1S3
Unclustered	CDRH1	2YKL	H	GGS	human	IGHV4-39
Unclustered	CDRH1	2BSE	D	RRTGSNW	llama	IGHV1S3
Unclustered	CDRH1	1OCW	H	GYTFTSY	house mouse	IGHV1-72
Unclustered	CDRH1	1I7Z	D	GYSFTNY	house mouse	IGHV9-3-1
Unclustered	CDRH1	2HMI	D	GFSLSTSGI	house mouse	IGHV8-12
Unclustered	CDRH1	4DN4	H	GGTFSSY	human	IGHV1-69
Unclustered	CDRH1	3EAK	A	GGSEYSYSTF	Arabian camel	IGHV1S27
Unclustered	CDRH1	3EAK	B	GGSEYSYSTF	Arabian camel	IGHV1S27
Unclustered	CDRH1	1K4D	A	GYTFTSD	house mouse	IGHV1S81
Unclustered	CDRH1	3B2U	T	GGSISSGDY	human	IGHV4-30-4
Unclustered	CDRH1	3K7U	A	GRTFSNN	llama	IGHV1S3
Unclustered	CDRH1	4JO3	I	GFSFTNNY	rabbit	IGHV1S40
Unclustered	CDRH1	3B2U	F	GGSISSGDY	human	IGHV4-30-4
Unclustered	CDRH1	4KRM	J	GRTSRSY	llama	IGHV1S1
Unclustered	CDRH1	3ULS	B	GDSVSSNSA	human	IGHV6-1
Unclustered	CDRH1	3NPS	B	GGTFSSY	human	IGHV1-69
Unclustered	CDRH1	2XT1	B	GSFFMSN	llama	IGHV1S4
Unclustered	CDRH1	2P7T	A	GYTFTSD	house mouse	IGHV1S81
Unclustered	CDRH1	2DWE	A	GYTFTSD	house mouse	IGHV1S81
Unclustered	CDRH1	4N9O	B	GRTFSSY	llama	IGHV1S1
Unclustered	CDRH1	3W9D	A	GGTLRTY	house mouse	IGHV1-69
Unclustered	CDRH1	3STZ	A	GYTFTSD	house mouse	IGHV1S81
Unclustered	CDRH1	3GBN	H	GGPFRSY	human	IGHV1-69
Unclustered	CDRH1	4JO1	I	GFSFGNNY	rabbit	IGHV1S40
Unclustered	CDRH1	3QXV	A	RRSSRSW	llama	IGHV1S3
Unclustered	CDRH1	2AJY	H	GYSITTNY	house mouse	IGHV3-2
Unclustered	CDRH1	4AT6	A	GYSFSSY	house mouse	IGHV1-12
Unclustered	CDRH1	3EO0	D	GYTFSSN	human	IGHV1-69
Unclustered	CDRH1	4FNL	I	GSSFGESTLSYY	human	IGHV3-23
Unclustered	CDRH1	3QXU	C	RRSSRSW	llama	IGHV1S5
Unclustered	CDRH1	2X1O	A	GRNLRMY	llama	IGHV1S1
Unclustered	CDRH1	3U1S	H	GNSFSNH	human	IGHV1-8
Unclustered	CDRH1	4EJ1	D	GRTFSSY	llama	IGHV1S3
Unclustered	CDRH1	2VXQ	H	GGSIRSGGY	human	IGHV4-31
Unclustered	CDRH1	4JZN	I	GGTLSNY	human	IGHV1-69

Unclustered	CDRH1	4KDT	B	GYTDSRY	Arabian camel	IGHV1S47
Unclustered	CDRH1	1UM4	H	EYTLTSY	house mouse	IGHV1-14
Unclustered	CDRH1	3QCV	H	GYGFINY	house mouse	IGHV5-51
Unclustered	CDRH1	3U6R	A	GDTFSRY	human	IGHV1-69
Unclustered	CDRH1	1RZF	H	GATLNSH	human	IGHV1-69
Unclustered	CDRH1	1RHH	B	GGTFSMY	human	IGHV1-69
Unclustered	CDRH1	1QD0	A	GRAASGHGHY	llama	IGHV1S3
Unclustered	CDRH1	1RZG	A	GGTFSNY	human	IGHV1-69
Unclustered	CDRH1	1RHH	D	GGTFSMY	human	IGHV1-69
Unclustered	CDRH1	4B5E	B	GRSFSRD	llama	IGHV1S3
Unclustered	CDRH1	4KRM	D	GRTSRSY	llama	IGHV1S1
Unclustered	CDRH1	3QHZ	H	GFSLSTSGM	human	IGHV2-70
Unclustered	CDRH1	1HCV	A	GRTGSTY	llama	IGHV
Unclustered	CDRH1	2IH3	A	GYTFTSD	house mouse	IGHV1S81
Unclustered	CDRH1	1UM6	H	EYTLTSY	house mouse	IGHV1-14
Unclustered	CDRH1	4HPY	H	GFTFDDG	human	IGHV3-9
Unclustered	CDRH1	1U0Q	A	GRTFSTY	llama	IGHV1S1
Unclustered	CDRH1	2XTJ	D	GGTFNSH	human	IGHV1-69
Unclustered	CDRH1	4F37	H	GFSLRTSRV	house mouse	IGHV8-12
Unclustered	CDRH1	4LLV	C	GGSFSTY	human	IGHV1-69
Unclustered	CDRH1	4HJJ	H	GFSLSKSVM	human	IGHV2-70
Unclustered	CDRH1	4KRM	L	GRTSRSY	llama	IGHV1S1
Unclustered	CDRH1	1YC7	A	GSTYSPC	Arabian camel	IGHV1S55
Unclustered	CDRH1	4JO2	H	GFSFGNNY	rabbit	IGHV1S40
Unclustered	CDRH1	2H8P	A	GYTFTSD	house mouse	IGHV1S81
Unclustered	CDRH1	1UWE	V	AYSITDF	house mouse	IGHV1S135
Unclustered	CDRH1	3H0T	B	GDSVSSNSA	human	IGHV6-1
Unclustered	CDRH1	1CGS	H	GYTFSEY	house mouse	IGHV1-9
Unclustered	CDRH1	3FZU	C	GFRFTFNNY	human	IGHV3-11
Unclustered	CDRH1	2BSE	F	RRTGSNW	llama	IGHV1S3
Unclustered	CDRH1	2HFE	A	GYTFTSD	house mouse	IGHV1S81
Unclustered	CDRH1	2JK5	A	GYTFTSD	house mouse	IGHV1S81
Unclustered	CDRH1	2X89	A	GYTDSRY	Arabian camel	IGHV1S47
Unclustered	CDRH1	1SHM	B	GATGSTY	llama	IGHV1S3
Unclustered	CDRH1	1AI1	H	GFSITRTNY	house mouse	IGHV12-1-1
Unclustered	CDRH1	3QYC	B	GVRLSAY	human	IGHV3-23
Unclustered	CDRH1	1KXV	C	GNTLCTY	Arabian camel	IGHV1S52
Unclustered	CDRH1	2AJS	H	GYSITTNY	house mouse	IGHV3-2
Unclustered	CDRH1	2XXC	B	GSFFMSN	alpaca	IGHV
Unclustered	CDRH1	3B2U	J	GGSISSGDY	human	IGHV4-30-4
Unclustered	CDRH1	3GBM	I	GGPFRSY	human	IGHV1-69
Unclustered	CDRH1	1UWG	H	AYSITDF	house mouse	IGHV1S135
Unclustered	CDRH1	1IGJ	B	GYIFTDF	house mouse	IGHV1-34
Unclustered	CDRH1	4NC1	C	GRTFSMD	llama	IGHV1S3
Unclustered	CDRH1	1DN0	B	GGSFSDY	human	IGHV4-34
Unclustered	CDRH1	4B41	B	RSIISNN	llama	IGHV1S3
Unclustered	CDRH1	3GO1	H	GGPINNA	human	IGHV4-59
Unclustered	CDRH1	1SHM	E	GATGSTY	llama	IGHV1S3
Unclustered	CDRH1	1UWG	Y	AYSITDF	house mouse	IGHV1S135
Unclustered	CDRH1	4NBX	B	GRTFSMD	llama	IGHV1S3

Unclustered	CDRH1	2XRA	H	GGTFNRL	human	IGHV1-69
Unclustered	CDRH1	2R0L	H	GFTISNS	human	IGHV3-66
Unclustered	CDRH1	2AJZ	H	GYSITTNY	house mouse	IGHV3-2
Unclustered	CDRH1	2YSS	B	GDSIRSD	human	IGHV4-59
Unclustered	CDRH1	3TNM	H	GGSSSSGAH	human	IGHV4-30-4
Unclustered	CDRH1	1IGJ	D	GYIFTDF	house mouse	IGHV1-34
Unclustered	CDRH1	2G75	A	GGTFSSY	human	IGHV1-69
Unclustered	CDRH1	4JO1	H	GFSFGNNY	rabbit	IGHV1S40
Unclustered	CDRH1	2BSE	E	RRTGSNW	llama	IGHV1S3
Unclustered	CDRH1	3U6R	H	GDTFSRY	human	IGHV1-69
Unclustered	CDRH1	3CX5	U	GYSITSGY	house mouse	IGHV3-6
Unclustered	CDRH1	3ULS	H	GDSVSSNSA	human	IGHV6-1
Unclustered	CDRH1	4JN1	H	GFSLTSY	house mouse	IGHV2-9
Unclustered	CDRH1	1RI8	A	GYKDRNY	Arabian camel	IGHV1S47
Unclustered	CDRH1	3FZU	H	GFRFTFNNY	human	IGHV3-11
Unclustered	CDRH1	3B2U	H	GGSISSGDY	human	IGHV4-30-4
Unclustered	CDRH1	1IC4	H	GDSITSA	house mouse	IGHV3-8
Unclustered	CDRH1	4GFT	B	PERAFSNY	llama	IGHV
Unclustered	CDRH1	1KXV	D	GNTLCTY	Arabian camel	IGHV1S52
Unclustered	CDRH1	4N9G	A	GGSFSSY	human	IGHV4-34
Unclustered	CDRH1	3QXT	A	RRSSRSW	Arabian camel	IGHV1S72
Unclustered	CDRH1	4FNL	H	GSSFGESTLSYY	human	IGHV3-23
Unclustered	CDRH1	2AJZ	B	GYSITTNY	house mouse	IGHV3-2
Unclustered	CDRH1	4KRM	H	GRTSRSY	llama	IGHV1S1
Unclustered	CDRH1	2W9D	H	RNTFTDY	house mouse	IGHV1-34
Unclustered	CDRH1	1A4J	H	GYTFTNY	house mouse	IGHV9-3-1
Unclustered	CDRH1	4B41	A	RSIISNN	llama	IGHV1S3
Unclustered	CDRH1	1R3I	H	GYTFTSD	house mouse	IGHV1S81
Unclustered	CDRH1	3QOT	H	GGTFSSY	human	IGHV1-69
Unclustered	CDRH1	3B2U	Q	GGSISSGDY	human	IGHV4-30-4
Unclustered	CDRH1	4NBY	B	GRTFSMD	llama	IGHV1S3
Unclustered	CDRH1	3B2U	C	GGSISSGDY	human	IGHV4-30-4
Unclustered	CDRH1	1A4K	B	GYTFTNY	house mouse	IGHV9-3-1
Unclustered	CDRH1	2HVK	A	GYTFTSD	house mouse	IGHV1S81
Unclustered	CDRH1	2ITD	A	GYTFTSD	house mouse	IGHV1S81
Unclustered	CDRH1	2X89	B	GYTDSRY	Arabian camel	IGHV1S47
Unclustered	CDRH1	1UWE	H	AYSITDF	house mouse	IGHV1S135
Unclustered	CDRH1	3QCU	H	GYGFINY	house mouse	IGHV5-51
Unclustered	CDRH1	4JO4	I	GFSFTNNY	rabbit	IGHV1S40
Unclustered	CDRH1	1R3J	B	GYTFTSD	house mouse	IGHV1S81
Unclustered	CDRH1	1NBV	H	GFSFNTN	house mouse	IGHV10S3
Unclustered	CDRH1	3QXV	D	RRSSRSW	llama	IGHV1S3
Unclustered	CDRH1	4JO3	H	GFSFTNNY	rabbit	IGHV1S40
Unclustered	CDRH1	3QCV	I	GYGFINY	house mouse	IGHV5-51
Unclustered	CDRH1	2X1O	B	GRNLRMY	llama	IGHV1S1
Unclustered	CDRH1	1ZVY	A	GSTDSIE	Arabian camel	IGHV1S45
Unclustered	CDRH1	1U0Q	B	GRTFSTY	llama	IGHV1S1
Unclustered	CDRH1	2XV6	B	GSFFMSN	alpaca	IGHV
Unclustered	CDRH1	4HS6	H	GDSITSG	house mouse	IGHV4-59
Unclustered	CDRH1	4GRW	F	GFTLDYL	llama	IGHV1S1

Unclustered	CDRH1	2HKH	H	GSTLNNY	house mouse	IGHV10-1
Unclustered	CDRH1	3GBM	H	GGPFRSY	human	IGHV1-69
Unclustered	CDRH1	1BGX	H	GYSITSDY	house mouse	IGHV3-2
Unclustered	CDRH1	4JM4	H	GDSIRGGEWGDKDY	human	IGHV4-39
Unclustered	CDRH1	2XA3	A	ARTSSSH	llama	IGHV1S3
Unclustered	CDRH1	3QCT	H	GYGFINY	house mouse	IGHV5-51
Unclustered	CDRH1	1ZV5	A	GVTYKNY	Arabian camel	IGHV1S47
Unclustered	CDRH1	2G75	C	GGTFSSY	human	IGHV1-69
Unclustered	CDRH1	2HG5	A	GYTFTSD	house mouse	IGHV1S81
Unclustered	CDRH1	1A6V	J	GYTFTSY	house mouse	IGHV1-72
Unclustered	CDRH1	3AAZ	H	GFSLRTSRV	house mouse	IGHV2-5
Unclustered	CDRH1	1YZZ	B	GSTYSPC	Arabian camel	IGHV1S17
Unclustered	CDRH1	2XXM	B	GSFFMSN	llama	IGHV1S4
Unclustered	CDRH1	1S5H	B	GYTFTSD	house mouse	IGHV1S81
Unclustered	CDRH1	4NC1	D	GRTFSMD	llama	IGHV1S3
Unclustered	CDRH1	4AT6	E	GYSFSSY	house mouse	IGHV1-12
Unclustered	CDRH1	2AK1	H	GYSITTNY	house mouse	IGHV3-2
Unclustered	CDRH1	3OR7	A	GYTFTSD	house mouse	IGHV1S81
Unclustered	CDRH1	3W9E	A	GGTLRTY	house mouse	IGHV1-69
Unclustered	CDRH1	1NSN	H	GYSITSDY	house mouse	IGHV3-2
Unclustered	CDRH1	2QJD	A	DKN	channel catfish	NITR11
Unclustered	CDRH1	1CBV	H	GFSFNTN	house mouse	IGHV10S3
Unclustered	CDRH1	3G9A	B	GDTFSSY	llama	IGHV1S3
Unclustered	CDRH1	2DWD	A	GYTFTSD	house mouse	IGHV1S81
Unclustered	CDRH1	4JAM	H	GGSMGGT	human	IGHV4-61
Unclustered	CDRH1	3GRW	H	GFTFTST	human	IGHV3-66
Unclustered	CDRH1	1K4C	A	GYTFTSD	house mouse	IGHV1S81
Unclustered	CDRH1	4KTE	H	GGSISGGY	rhesus monkey	IGHV4-2
Unclustered	CDRH1	4GRW	H	GRTFSWS	llama	IGHV1S3
Unclustered	CDRH1	1A4K	H	GYTFTNY	house mouse	IGHV9-3-1
Unclustered	CDRH1	3AAZ	A	GFSLRTSRV	house mouse	IGHV2-5
Unclustered	CDRH1	4JO2	I	GFSFGNNY	rabbit	IGHV1S40
Unclustered	CDRH1	1RZG	C	GGTFSNY	human	IGHV1-69
Unclustered	CDRH1	4AT6	H	GYSFSSY	house mouse	IGHV1-12
Unclustered	CDRH1	4KRN	A	GRTFSSY	llama	IGHV1S1
Unclustered	CDRH1	1UWE	Y	AYSITDF	house mouse	IGHV1S135
Unclustered	CDRH1	1AJ7	H	GFNIKDT	house mouse	IGHV14-3
Unclustered	CDRH1	3QXV	C	RRSSRSW	llama	IGHV1S3
Unclustered	CDRH1	3QEG	H	RGTFSSY	human	IGHV1-69
Unclustered	CDRH1	1MJ8	H	GYTFTSN	house mouse	IGHV1S61
Unclustered	CDRH1	3LMJ	H	GFTLTGL	human	IGHV1-24
Unclustered	CDRH1	1NGX	B	GYTFTSY	house mouse	IGHV1S36
Unclustered	CDRH1	1DN0	D	GGSFSDY	human	IGHV4-34
Unclustered	CDRH1	3R0M	A	GRISSSY	human	IGHV3-23
Unclustered	CDRH1	4K3D	I	GFSLSDK	sheep	IGHV1S2
Unclustered	CDRH1	4I13	B	ERLTVDY	llama	IGHV1S1
Unclustered	CDRH1	4LAS	H	GDSVTSG	house mouse	IGHV3-8
Unclustered	CDRH1	4CDG	D	GIWFSIN	llama	IGHV
Unclustered	CDRH1	1NGX	H	GYTFTSY	house mouse	IGHV1S36
Unclustered	CDRH1	4AQ1	D	GRTSSAY	llama	IGHV1S1

H2-7-A	CDRH2	1F90	H	YITYSGS	house mouse	IGHV3-2
H2-7-A	CDRH2	3B2U	T	YIYYSGS	human	IGHV4-30-4
H2-7-A	CDRH2	43CA	D	MIWGGGS	house mouse	IGHV2-6-4
H2-7-A	CDRH2	2DQU	H	SISSGGS	house mouse	IGHV5S9
H2-7-A	CDRH2	2GK0	H	VIWSGGS	house mouse	IGHV2-9
H2-7-A	CDRH2	2XZC	H	SIYHSGS	human	IGHV4-38-2
H2-7-A	CDRH2	4DVB	A	SITNGGS	house mouse	IGHV5S9
H2-7-A	CDRH2	1NC2	D	VIWSGGG	house mouse	IGHV2-2
H2-7-A	CDRH2	3CX5	U	YISNVGD	house mouse	IGHV3-6
H2-7-A	CDRH2	1KXV	C	GIDNDGT	Arabian camel	IGHV1S52
H2-7-A	CDRH2	1UB5	A	SISSGGI	house mouse	IGHV5-9
H2-7-A	CDRH2	1I9J	H	SIVSGGN	house mouse	IGHV5S9
H2-7-A	CDRH2	3QRG	H	HIYWDDD	house mouse	IGHV2-5
H2-7-A	CDRH2	1XGP	B	YISYSGS	house mouse	IGHV3-8
H2-7-A	CDRH2	2VQ1	F	HIWWNDD	house mouse	IGHV8-8
H2-7-A	CDRH2	1NBZ	B	YISYSGS	house mouse	IGHV3-8
H2-7-A	CDRH2	3FO2	B	VIWSGGS	house mouse	IGHV2-9
H2-7-A	CDRH2	1BVK	E	MIWGDGN	human	IGHV4-59
H2-7-A	CDRH2	4JAM	A	YIFHTGE	human	IGHV4-61
H2-7-A	CDRH2	3G5V	B	YISYSGN	house mouse	IGHV3-2
H2-7-A	CDRH2	1A7P	H	MIWGDGN	house mouse	IGHV2-6-7
H2-7-A	CDRH2	3VFG	H	VIWAGGI	house mouse	IGHV2-9
H2-7-A	CDRH2	3IDN	B	IIYSDDD	human	IGHV2-5
H2-7-A	CDRH2	3A6C	H	YVSYSGS	house mouse	IGHV3-8
H2-7-A	CDRH2	1RUA	H	YINYSGF	house mouse	IGHV3-2
H2-7-A	CDRH2	3LEX	A	YINYSGY	house mouse	IGHV3-2
H2-7-A	CDRH2	1GGI	J	HIFWDGD	house mouse	IGHV8-12
H2-7-A	CDRH2	4GAY	H	YISYSGS	house mouse	IGHV3-8
H2-7-A	CDRH2	1L7T	H	SIVSGGN	house mouse	IGHV5S9
H2-7-A	CDRH2	3KS0	K	YISYGGS	house mouse	IGHV3-8
H2-7-A	CDRH2	2DTM	H	SISSGGS	house mouse	IGHV5S9
H2-7-A	CDRH2	2Z92	A	YIHYRGT	house mouse	IGHV3-1
H2-7-A	CDRH2	3MOB	H	IIYSDDD	human	IGHV2-5
H2-7-A	CDRH2	1YY8	D	VIWSGGN	house mouse	IGHV2-2
H2-7-A	CDRH2	1J1X	H	YVSYSGS	house mouse	IGHV3-8
H2-7-A	CDRH2	3EZJ	F	TITSGGS	llama	IGHV1S3
H2-7-A	CDRH2	3Q1S	H	HIIYGGT	human	IGHV4-59
H2-7-A	CDRH2	3IFL	H	HIWWDDD	house mouse	IGHV8-12
H2-7-A	CDRH2	3IXT	A	DIWWDDK	human	IGHV2-70
H2-7-A	CDRH2	2B2X	H	VISGGGH	house mouse	IGHV5-9
H2-7-A	CDRH2	1KXT	D	RIFSDGS	Arabian camel	IGHV1S72
H2-7-A	CDRH2	1U91	B	IIYSDDD	human	IGHV2-5
H2-7-A	CDRH2	1TJH	H	IIYSDDD	human	IGHV2-5
H2-7-A	CDRH2	1FL3	A	SISSGGI	house mouse	IGHV5-9
H2-7-A	CDRH2	1CIC	D	MIWGDGN	house mouse	IGHV2-6-7
H2-7-A	CDRH2	3CFD	B	YISYSGR	house mouse	IGHV3-2
H2-7-A	CDRH2	3V6O	D	VIWGGGS	house mouse	IGHV2-6-5
H2-7-A	CDRH2	4JAM	H	YIFHTGE	human	IGHV4-61
H2-7-A	CDRH2	3IDJ	B	IIYSDDD	human	IGHV2-5
H2-7-A	CDRH2	4JN1	H	VIWAGGS	house mouse	IGHV2-9

H2-7-A	CDRH2	3RVV	D	YISYSGT	house mouse	IGHV3-2
H2-7-A	CDRH2	2DQD	H	FVSYSGS	house mouse	IGHV3-8
H2-7-A	CDRH2	1U8L	B	IIYSDDD	human	IGHV2-5
H2-7-A	CDRH2	4KPH	I	YINYSGN	house mouse	IGHV3-8
H2-7-A	CDRH2	3L5W	H	HIWWDDV	house mouse	IGHV8-8
H2-7-A	CDRH2	1NDG	B	YISFSGN	house mouse	IGHV3-8
H2-7-A	CDRH2	4I77	H	MIWGDGK	house mouse	IGHV2-70
H2-7-A	CDRH2	1A7O	H	MIWGDGN	house mouse	IGHV2-6-7
H2-7-A	CDRH2	3MOA	H	IIYSDDD	human	IGHV2-5
H2-7-A	CDRH2	1T4K	B	VIWGDGS	house mouse	IGHV2-6-5
H2-7-A	CDRH2	4M5Y	I	SIYHSGS	human	IGHV4-38-2
H2-7-A	CDRH2	3IXT	H	DIWWDDK	human	IGHV2-70
H2-7-A	CDRH2	1AI1	H	RICYEGS	house mouse	IGHV12-1-1
H2-7-A	CDRH2	1RUK	H	YINYSGF	house mouse	IGHV3-2
H2-7-A	CDRH2	3ZL4	H	SIYHSGS	human	IGHV4-38-2
H2-7-A	CDRH2	1YY9	D	VIWSGGN	house mouse	IGHV2-2
H2-7-A	CDRH2	1LO3	H	SISYGGL	house mouse	IGHV5S4
H2-7-A	CDRH2	3VG0	H	VIWGGGS	house mouse	IGHV2-6-5
H2-7-A	CDRH2	43C9	H	MIWGGGS	house mouse	IGHV2-6-4
H2-7-A	CDRH2	1CF8	H	YIRYSGD	house mouse	IGHV3-2
H2-7-A	CDRH2	2VXQ	H	YIYHSGN	human	IGHV4-31
H2-7-A	CDRH2	3B2U	H	YIYYSGS	human	IGHV4-30-4
H2-7-A	CDRH2	1DL7	H	MIWGDGS	house mouse	IGHV2-6-7
H2-7-A	CDRH2	2F5B	H	IIYSDDD	human	IGHV2-5
H2-7-A	CDRH2	7FAB	H	YVFYTGT	human	IGHV4-59
H2-7-A	CDRH2	1J1O	H	YVSYSGS	house mouse	IGHV3-8
H2-7-A	CDRH2	3U9P	K	GIWGDGR	Norway rat	IGHV2S5
H2-7-A	CDRH2	1U8N	B	IIYSDDD	human	IGHV2-5
H2-7-A	CDRH2	1MHP	X	TISGGGH	human	IGHV3-23
H2-7-A	CDRH2	3RVT	D	YISYSGT	house mouse	IGHV3-2
H2-7-A	CDRH2	4EIG	B	LITTNNN	llama	IGHV1S3
H2-7-A	CDRH2	3B9K	H	GIWADGS	Norway rat	IGHV2S5
H2-7-A	CDRH2	1FDL	H	MIWGDGN	house mouse	IGHV2-6-7
H2-7-A	CDRH2	1ORS	B	YINYSGT	house mouse	IGHV3-2
H2-7-A	CDRH2	1GGI	H	HIFWDGD	house mouse	IGHV8-12
H2-7-A	CDRH2	1GIG	H	VIWAGGN	house mouse	IGHV2-9
H2-7-A	CDRH2	3IU4	H	MIWGGGS	house mouse	IGHV2-6-4
H2-7-A	CDRH2	2YK1	H	SIYSSGS	human	IGHV4-39
H2-7-A	CDRH2	4NC2	B	AITWGGT	llama	IGHV1S3
H2-7-A	CDRH2	1KFA	H	TITTRGY	house mouse	IGHV5S9
H2-7-A	CDRH2	1U8H	B	IIYSDDD	human	IGHV2-5
H2-7-A	CDRH2	1F58	H	YIHYSAG	house mouse	IGHV3-1
H2-7-A	CDRH2	43C9	F	MIWGGGS	house mouse	IGHV2-6-4
H2-7-A	CDRH2	1FL3	H	SISSGGI	house mouse	IGHV5-9
H2-7-A	CDRH2	4LKX	A	SISYSGI	house mouse	IGHV4-38-2
H2-7-A	CDRH2	1NAK	I	YISKSGS	house mouse	IGHV3-8
H2-7-A	CDRH2	1GPO	H	FVQYSGE	house mouse	IGHV3-8
H2-7-A	CDRH2	1IC5	H	YVSYSGS	house mouse	IGHV3-8
H2-7-A	CDRH2	2DQT	H	SISSGGS	house mouse	IGHV5S9
H2-7-A	CDRH2	3D0L	B	IIYSDDD	human	IGHV2-5

H2-7-A	CDRH2	1KCV	H	YISYSGS	house mouse	IGHV3-2
H2-7-A	CDRH2	4F37	H	HIYWDDD	house mouse	IGHV8-12
H2-7-A	CDRH2	1KXV	D	GIDNDGT	Arabian camel	IGHV1S52
H2-7-A	CDRH2	3B2U	Q	YIYYSGS	human	IGHV4-30-4
H2-7-A	CDRH2	2DQG	H	YVSFSGS	house mouse	IGHV3-8
H2-7-A	CDRH2	3BAE	H	HIYWDDD	house mouse	IGHV8-12
H2-7-A	CDRH2	3FO1	H	VIWSGGS	house mouse	IGHV2-9
H2-7-A	CDRH2	1C08	B	YVSYSGS	house mouse	IGHV3S1
H2-7-A	CDRH2	1XGQ	B	YISYSGS	house mouse	IGHV3-8
H2-7-A	CDRH2	1A7Q	H	MIWGDGN	house mouse	IGHV2-6-7
H2-7-A	CDRH2	1HQ4	D	YIRYSGD	house mouse	IGHV3-2
H2-7-A	CDRH2	4KPH	H	YINYSGN	house mouse	IGHV3-8
H2-7-A	CDRH2	1DVF	B	MIWGDGN	house mouse	IGHV2-6-7
H2-7-A	CDRH2	43CA	F	MIWGGGS	house mouse	IGHV2-6-4
H2-7-A	CDRH2	4M5Y	H	SIYHSGS	human	IGHV4-38-2
H2-7-A	CDRH2	4K3D	H	SIDTGGN	sheep	IGHV1S2
H2-7-A	CDRH2	3B2U	N	YIYYSGS	human	IGHV4-30-4
H2-7-A	CDRH2	1U8M	B	IIYSDDD	human	IGHV2-5
H2-7-A	CDRH2	1DN0	D	EINHSGS	human	IGHV4-34
H2-7-A	CDRH2	1BVK	B	MIWGDGN	human	IGHV4-59
H2-7-A	CDRH2	3L7E	H	HIWWDDV	house mouse	IGHV8-8
H2-7-A	CDRH2	43CA	B	MIWGGGS	house mouse	IGHV2-6-4
H2-7-A	CDRH2	2DQF	E	YVSASGS	house mouse	IGHV3-8
H2-7-A	CDRH2	4LAS	H	YISYRGS	house mouse	IGHV3-8
H2-7-A	CDRH2	4DVB	H	SITNGGS	house mouse	IGHV5S9
H2-7-A	CDRH2	1UA6	H	YVSSFGS	house mouse	IGHV3-8
H2-7-A	CDRH2	2DQJ	H	YVSYSGS	house mouse	IGHV3-8
H2-7-A	CDRH2	1U8Q	B	IIYSDDD	human	IGHV2-5
H2-7-A	CDRH2	1F8T	H	YITYSGS	house mouse	IGHV3-2
H2-7-A	CDRH2	3QQ9	D	HIYWDDD	house mouse	IGHV8-12
H2-7-A	CDRH2	4G6A	C	YISYSGS	house mouse	IGHV4-30-4
H2-7-A	CDRH2	3BKC	H	HIYWDDD	house mouse	IGHV8-12
H2-7-A	CDRH2	1UB6	H	SISSGGI	house mouse	IGHV5-9
H2-7-A	CDRH2	1GGB	H	HIFWDGD	house mouse	IGHV8-12
H2-7-A	CDRH2	1G7H	B	MIWGDGN	house mouse	IGHV2-6-7
H2-7-A	CDRH2	3B2U	J	YIYYSGS	human	IGHV4-30-4
H2-7-A	CDRH2	1SJX	A	AITSGGS	llama	IGHV1S3
H2-7-A	CDRH2	3MLX	H	YIYYSGS	human	IGHV4-59
H2-7-A	CDRH2	2PW2	B	IIYSDDD	human	IGHV2-5
H2-7-A	CDRH2	3IFN	H	HIWWDDD	house mouse	IGHV8-12
H2-7-A	CDRH2	1U92	B	IIYSDDD	human	IGHV2-5
H2-7-A	CDRH2	1NC4	D	VIWSGGG	house mouse	IGHV2-2
H2-7-A	CDRH2	2IPU	H	HIWWDDD	house mouse	IGHV8-12
H2-7-A	CDRH2	3O41	A	HIYWDDD	house mouse	IGHV8-12
H2-7-A	CDRH2	4GAJ	H	YISYSGS	house mouse	IGHV3-8
H2-7-A	CDRH2	1T4K	D	VIWGDGS	house mouse	IGHV2-6-5
H2-7-A	CDRH2	1DQJ	B	YISYSGS	house mouse	IGHV3-8
H2-7-A	CDRH2	4FQQ	B	YIYYSGS	human	IGHV4-59
H2-7-A	CDRH2	3RKD	D	HIWWDDV	house mouse	IGHV8-8
H2-7-A	CDRH2	3CFB	H	SISSGGI	house mouse	IGHV5-9

H2-7-A	CDRH2	3LS5	H	SISRGGS	house mouse	IGHV5-9-3
H2-7-A	CDRH2	1LO2	H	SISYGGL	house mouse	IGHV5S4
H2-7-A	CDRH2	1A7N	H	MIWGDGN	house mouse	IGHV2-6-7
H2-7-A	CDRH2	1RU9	H	YINYSGF	house mouse	IGHV3-2
H2-7-A	CDRH2	4G6A	H	YISYSGS	house mouse	IGHV4-30-4
H2-7-A	CDRH2	3QWO	H	DIWWDDK	human	IGHV2-70
H2-7-A	CDRH2	2F58	H	YIHYSAG	house mouse	IGHV3-1
H2-7-A	CDRH2	2P8P	B	IIYSDDD	human	IGHV2-5
H2-7-A	CDRH2	1MHP	H	TISGGGH	human	IGHV3-23
H2-7-A	CDRH2	4HBC	H	IISNFGV	rabbit	IGHV1S69
H2-7-A	CDRH2	1IC4	H	YVSYSGS	house mouse	IGHV3-8
H2-7-A	CDRH2	1U8K	B	IIYSDDD	human	IGHV2-5
H2-7-A	CDRH2	3L7F	H	HIWWDDV	human	IGHV2-5
H2-7-A	CDRH2	1KCS	H	YISYSGS	house mouse	IGHV3-2
H2-7-A	CDRH2	3AAZ	H	HIYWDDD	house mouse	IGHV2-5
H2-7-A	CDRH2	3CFI	F	GINRDGS	llama	IGHV1S6
H2-7-A	CDRH2	43C9	B	MIWGGGS	house mouse	IGHV2-6-4
H2-7-A	CDRH2	1KFA	I	TITTRGY	house mouse	IGHV5S9
H2-7-A	CDRH2	1HQ4	B	YIRYSGD	house mouse	IGHV3-2
H2-7-A	CDRH2	3L5X	H	HIWWDDV	human	IGHV2-5
H2-7-A	CDRH2	2DQI	H	YVSYSGS	house mouse	IGHV3-8
H2-7-A	CDRH2	3B2U	W	YIYYSGS	human	IGHV4-30-4
H2-7-A	CDRH2	2XZA	H	SIYHSGS	human	IGHV4-38-2
H2-7-A	CDRH2	1UB5	H	SISSGGI	house mouse	IGHV5-9
H2-7-A	CDRH2	4B41	A	RISSGGR	llama	IGHV1S3
H2-7-A	CDRH2	3CX5	J	YISNVGD	house mouse	IGHV3-6
H2-7-A	CDRH2	1KXT	B	RIFSDGS	Arabian camel	IGHV1S72
H2-7-A	CDRH2	2XA8	H	SITYDGS	house mouse	IGHV3-66
H2-7-A	CDRH2	43CA	H	MIWGGGS	house mouse	IGHV2-6-4
H2-7-A	CDRH2	3FO2	H	VIWSGGS	house mouse	IGHV2-9
H2-7-A	CDRH2	1NC2	B	VIWSGGG	house mouse	IGHV2-2
H2-7-A	CDRH2	1KCU	H	YISYSGS	house mouse	IGHV3-2
H2-7-A	CDRH2	1DQQ	D	YISYSGS	house mouse	IGHV3-8
H2-7-A	CDRH2	1NC4	B	VIWSGGG	house mouse	IGHV2-2
H2-7-A	CDRH2	2YKL	H	SIYSSGS	human	IGHV4-39
H2-7-A	CDRH2	1G7M	B	MIWGDGN	house mouse	IGHV2-6-7
H2-7-A	CDRH2	1OAK	H	MIWGDGS	house mouse	IGHV2-6-7
H2-7-A	CDRH2	3QQ9	H	HIYWDDD	house mouse	IGHV8-12
H2-7-A	CDRH2	4FQQ	H	YIYYSGS	human	IGHV4-59
H2-7-A	CDRH2	3IFO	A	HIYWDDD	house mouse	IGHV8-12
H2-7-A	CDRH2	3CFI	I	GINRDGS	llama	IGHV1S6
H2-7-A	CDRH2	1J1P	H	YVSYSGS	house mouse	IGHV3-8
H2-7-A	CDRH2	1NDM	B	YISYSGS	house mouse	IGHV3-8
H2-7-A	CDRH2	3MLY	H	YIYYSGS	human	IGHV4-59
H2-7-A	CDRH2	2PW1	B	IIYSDDD	human	IGHV2-5
H2-7-A	CDRH2	2Z91	C	YIHYRGT	house mouse	IGHV3-1
H2-7-A	CDRH2	3FO1	B	VIWSGGS	house mouse	IGHV2-9
H2-7-A	CDRH2	1DQQ	B	YISYSGS	house mouse	IGHV3-8
H2-7-A	CDRH2	3B2U	F	YIYYSGS	human	IGHV4-30-4
H2-7-A	CDRH2	4K3E	I	SIDTGGS	sheep	IGHV1S3

H2-7-A	CDRH2	3MLX	I	YIYYSGS	human	IGHV4-59
H2-7-A	CDRH2	3G5X	B	YISYSGN	house mouse	IGHV3-2
H2-7-A	CDRH2	1T2Q	H	VIWSGGS	house mouse	IGHV2-2
H2-7-A	CDRH2	3G5Z	B	YISYSGN	house mouse	IGHV3-2
H2-7-A	CDRH2	1GPO	I	FVQYSGE	house mouse	IGHV3-8
H2-7-A	CDRH2	1DQM	H	YISYSGS	house mouse	IGHV3-8
H2-7-A	CDRH2	1NBY	B	YISYSGS	house mouse	IGHV3-8
H2-7-A	CDRH2	3LS4	H	SISRGGS	house mouse	IGHV5-9-3
H2-7-A	CDRH2	3IDM	B	IIYSDDD	human	IGHV2-5
H2-7-A	CDRH2	1U93	B	IIYSDDD	human	IGHV2-5
H2-7-A	CDRH2	1U8J	B	IIYSDDD	human	IGHV2-5
H2-7-A	CDRH2	1G7I	B	MIWGDGN	house mouse	IGHV2-6-7
H2-7-A	CDRH2	1G7J	B	MIWGDGN	house mouse	IGHV2-6-7
H2-7-A	CDRH2	3MLY	I	YIYYSGS	human	IGHV4-59
H2-7-A	CDRH2	3KS0	H	YISYGGS	house mouse	IGHV3-8
H2-7-A	CDRH2	3TNM	A	YIHYSGN	human	IGHV4-30-4
H2-7-A	CDRH2	1ND0	H	YINYSGF	house mouse	IGHV3-2
H2-7-A	CDRH2	3GO1	H	YVYHTGV	human	IGHV4-59
H2-7-A	CDRH2	4IJ3	C	MIWGDGR	house mouse	IGHV2-6-7
H2-7-A	CDRH2	1JRH	H	HIWWDDD	house mouse	IGHV8-12
H2-7-A	CDRH2	3EYU	H	HIWWDDD	house mouse	IGHV8-12
H2-7-A	CDRH2	1LO4	H	SISYGGL	house mouse	IGHV5S4
H2-7-A	CDRH2	4G6K	H	HIWWDGD	human	IGHV8-12
H2-7-A	CDRH2	1A2Y	B	MIWGDGN	house mouse	IGHV2-6-7
H2-7-A	CDRH2	1RUL	H	YINYSGF	house mouse	IGHV3-2
H2-7-A	CDRH2	1UB6	A	SISSGGI	house mouse	IGHV5-9
H2-7-A	CDRH2	3A67	H	YVSYSGS	house mouse	IGHV3-8
H2-7-A	CDRH2	2IQA	B	HIWWDDD	house mouse	IGHV8-12
H2-7-A	CDRH2	1G7L	B	MIWGDGN	house mouse	IGHV2-6-7
H2-7-A	CDRH2	4LKC	B	AIGTAGD	human	IGHV3-13
H2-7-A	CDRH2	1VFA	B	MIWGDGN	house mouse	IGHV2-6-7
H2-7-A	CDRH2	3CFD	H	YISYSGR	house mouse	IGHV3-2
H2-7-A	CDRH2	1ND0	F	YINYSGF	house mouse	IGHV3-2
H2-7-A	CDRH2	3V6O	C	VIWGGGS	house mouse	IGHV2-6-5
H2-7-A	CDRH2	3RKD	H	HIWWDDV	house mouse	IGHV8-8
H2-7-A	CDRH2	1LO0	H	SISYGGL	house mouse	IGHV5S4
H2-7-A	CDRH2	1ND0	B	YINYSGF	house mouse	IGHV3-2
H2-7-A	CDRH2	1U8I	B	IIYSDDD	human	IGHV2-5
H2-7-A	CDRH2	1RUP	H	YINYSGF	house mouse	IGHV3-2
H2-7-A	CDRH2	3L7E	B	HIWWDDV	house mouse	IGHV8-8
H2-7-A	CDRH2	2DQC	H	YVSYSGS	house mouse	IGHV3-8
H2-7-A	CDRH2	3EZJ	B	TITSGGS	llama	IGHV1S3
H2-7-A	CDRH2	2IPT	H	HIWWDDD	house mouse	IGHV8-12
H2-7-A	CDRH2	2IPU	G	HIWWDDD	house mouse	IGHV8-12
H2-7-A	CDRH2	1C12	B	YISYSGS	house mouse	IGHV3-2
H2-7-A	CDRH2	1FN4	D	RMWYDGY	Norway rat	IGHV2S85
H2-7-A	CDRH2	1VFB	B	MIWGDGN	house mouse	IGHV2-6-7
H2-7-A	CDRH2	3IFO	H	HIYWDDD	house mouse	IGHV8-12
H2-7-A	CDRH2	43C9	D	MIWGGGS	house mouse	IGHV2-6-4
H2-7-A	CDRH2	3STB	B	AINRSGS	llama	IGHV1S1

H2-7-A	CDRH2	3EYS	H	HIWWDDD	house mouse	IGHV8-12
H2-7-A	CDRH2	4NPY	B	YIYYSGS	human	IGHV4-59
H2-7-A	CDRH2	3G9A	B	NILRDGT	llama	IGHV1S3
H2-7-A	CDRH2	2F5A	H	IIYSDDD	human	IGHV2-5
H2-7-A	CDRH2	1ETZ	B	DIWWNDK	house mouse	IGHV8-5
H2-7-A	CDRH2	3LD8	C	YIYNSGG	Norway rat	IGHV3S3
H2-7-A	CDRH2	1FN4	B	RMWYDGY	Norway rat	IGHV2S85
H2-7-A	CDRH2	4K3D	I	SIDTGGN	sheep	IGHV1S2
H2-7-A	CDRH2	2D03	H	VIWSGGS	house mouse	IGHV2-2
H2-7-A	CDRH2	3LDB	C	YIYNSGG	Norway rat	IGHV3S3
H2-7-A	CDRH2	4LAR	H	YISYRGS	house mouse	IGHV3-8
H2-7-A	CDRH2	3ZKM	C	VIWAGGT	house mouse	IGHV2-9
H2-7-A	CDRH2	1NAK	H	YISKSGS	house mouse	IGHV3-8
H2-7-A	CDRH2	4J1U	F	VIWGGGS	house mouse	IGHV2-6-5
H2-7-A	CDRH2	1A7R	H	MIWGDGN	house mouse	IGHV2-6-7
H2-7-A	CDRH2	3G5X	D	YISYSGN	house mouse	IGHV3-2
H2-7-A	CDRH2	1KIR	B	MIWGDGN	house mouse	IGHV2-6-7
H2-7-A	CDRH2	3L7F	E	HIWWDDV	human	IGHV2-5
H2-7-A	CDRH2	1YZZ	A	SISSPGT	Arabian camel	IGHV1S17
H2-7-A	CDRH2	2DQF	B	YVSASGS	house mouse	IGHV3-8
H2-7-A	CDRH2	3ZKN	C	VIWAGGT	house mouse	IGHV2-9
H2-7-A	CDRH2	3A6B	H	YVSYSGS	house mouse	IGHV3-8
H2-7-A	CDRH2	4G6M	H	HIWWDGD	house mouse	IGHV8-12
H2-7-A	CDRH2	4GW5	D	VIWSGGN	house mouse	IGHV2-2
H2-7-A	CDRH2	2DQE	H	YVSASGS	house mouse	IGHV3-8
H2-7-A	CDRH2	1FE8	J	VIWGDGN	house mouse	IGHV2-3
H2-7-A	CDRH2	3D9A	H	YVSYSGS	house mouse	IGHV3-8
H2-7-A	CDRH2	3B9K	D	GIWADGS	Norway rat	IGHV2S5
H2-7-A	CDRH2	2B2X	I	VISGGGH	house mouse	IGHV5-9
H2-7-A	CDRH2	3EZJ	D	TITSGGS	llama	IGHV1S3
H2-7-A	CDRH2	2EIZ	B	YVSYSGS	human	IGHV4-59
H2-7-A	CDRH2	1FE8	H	VIWGDGN	house mouse	IGHV2-3
H2-7-A	CDRH2	3BKJ	H	HIYWDDD	house mouse	IGHV8-12
H2-7-A	CDRH2	4K3E	H	SIDTGGS	sheep	IGHV1S3
H2-7-A	CDRH2	3F58	H	YIHYSAG	house mouse	IGHV3-1
H2-7-A	CDRH2	3G5Y	B	YISYSAN	house mouse	IGHV3-2
H2-7-A	CDRH2	3RVX	D	YISYSGT	house mouse	IGHV3-2
H2-7-A	CDRH2	3QWO	A	DIWWDDK	human	IGHV2-70
H2-7-A	CDRH2	3MCK	B	HIYWDDD	house mouse	IGHV8-12
H2-7-A	CDRH2	3B2U	C	YIYYSGS	human	IGHV4-30-4
H2-7-A	CDRH2	3DRQ	B	IIYSDDD	human	IGHV2-5
H2-7-A	CDRH2	3O41	H	HIYWDDD	house mouse	IGHV8-12
H2-7-A	CDRH2	3FO0	H	VIWSGGS	house mouse	IGHV2-9
H2-7-A	CDRH2	1FE8	I	VIWGDGN	house mouse	IGHV2-3
H2-7-A	CDRH2	1IBG	H	LIWAGGN	house mouse	IGHV2-9
H2-7-A	CDRH2	3MCK	H	HIYWDDD	house mouse	IGHV8-12
H2-7-A	CDRH2	1LO2	Y	SISYGGL	house mouse	IGHV5S4
H2-7-A	CDRH2	1KXT	F	RIFSDGS	Arabian camel	IGHV1S72
H2-7-A	CDRH2	3QG6	H	FISSYGT	house mouse	IGHV3-2
H2-7-A	CDRH2	1KIQ	B	MIWGDGN	house mouse	IGHV2-6-7

H2-7-A	CDRH2	4FQ2	H	YISDRES	human	IGHV4-59
H2-7-A	CDRH2	1TJG	H	IIYSDDD	human	IGHV2-5
H2-7-A	CDRH2	1S5I	H	YITYSGS	house mouse	IGHV3-2
H2-7-A	CDRH2	4FQQ	D	YIYYSGS	human	IGHV4-59
H2-7-A	CDRH2	1ND0	D	YINYSGF	house mouse	IGHV3-2
H2-7-A	CDRH2	1TJI	H	IIYSDDD	human	IGHV2-5
H2-7-A	CDRH2	1LO3	Y	SISYGGL	house mouse	IGHV5S4
H2-7-A	CDRH2	3ZKN	H	VIWAGGT	house mouse	IGHV2-9
H2-7-A	CDRH2	3EZJ	H	TITSGGS	llama	IGHV1S3
H2-7-A	CDRH2	1C5D	H	AISSGGS	Norway rat	IGHV2S8
H2-7-A	CDRH2	4NZR	H	SIHWRGT	human	IGHV4-39
H2-7-A	CDRH2	3L5W	B	HIWWDDV	house mouse	IGHV8-8
H2-7-A	CDRH2	3RVW	D	YISYSGT	house mouse	IGHV3-2
H2-7-A	CDRH2	2R0W	H	HIWWDDD	house mouse	IGHV8-12
H2-7-A	CDRH2	1FNS	H	MIWGDGS	house mouse	IGHV2-6-7
H2-7-A	CDRH2	4HS6	B	YISYSGS	house mouse	IGHV4-59
H2-7-A	CDRH2	1GGC	H	HIFWDGD	house mouse	IGHV8-12
H2-7-A	CDRH2	3CFC	H	SISSGGI	house mouse	IGHV5-9
H2-7-A	CDRH2	3STB	A	AINRSGS	llama	IGHV1S1
H2-7-A	CDRH2	3U9P	H	GIWGDGR	Norway rat	IGHV2S5
H2-7-A	CDRH2	4M5Z	H	SIYHSGS	human	IGHV4-38-2
H2-7-A	CDRH2	3BKM	H	HIYWDDD	house mouse	IGHV8-12
H2-7-A	CDRH2	3MBX	H	HIWWDDV	house mouse	IGHV8-8
H2-7-A	CDRH2	3LEY	H	YISYSGF	house mouse	IGHV3-2
H2-7-A	CDRH2	4JM4	H	SIHWRGT	human	IGHV4-39
H2-7-A	CDRH2	3CFB	B	SISSGGI	house mouse	IGHV5-9
H2-7-A	CDRH2	2P8M	B	IIYSDDD	human	IGHV2-5
H2-7-A	CDRH2	4JLR	H	DIWWDDK	human	IGHV2-70
H2-7-A	CDRH2	1IC7	H	YVSYSGS	house mouse	IGHV3-8
H2-7-A	CDRH2	1UAC	H	YVSSFGS	house mouse	IGHV3-8
H2-7-A	CDRH2	1KC5	H	YISYSGS	house mouse	IGHV3-2
H2-7-A	CDRH2	4GW1	B	VIWSGGN	house mouse	IGHV2-2
H2-7-A	CDRH2	4GAG	H	YISYSGS	house mouse	IGHV3-8
H2-7-A	CDRH2	4FQQ	F	YIYYSGS	human	IGHV4-59
H2-7-A	CDRH2	2IQ9	H	HIWWDDD	house mouse	IGHV8-12
H2-7-A	CDRH2	3L5Y	H	HIWWDDV	house mouse	IGHV2-5
H2-7-A	CDRH2	1NCW	H	YINYSGF	house mouse	IGHV3-2
H2-7-A	CDRH2	1LO0	Y	SISYGGL	house mouse	IGHV5S4
H2-7-A	CDRH2	4B41	B	RISSGGR	llama	IGHV1S3
H2-7-A	CDRH2	3EOT	H	TISGGGH	house mouse	IGHV5-9
H2-7-A	CDRH2	3FN0	H	HIIYGGT	human	IGHV4-59
H2-7-A	CDRH2	1NSN	H	YITYSGT	house mouse	IGHV3-2
H2-7-A	CDRH2	2PR4	H	IIYSDDD	human	IGHV2-5
H2-7-A	CDRH2	1ETZ	H	DIWWNDK	house mouse	IGHV8-5
H2-7-A	CDRH2	2VQ1	B	HIWWNDD	house mouse	IGHV8-8
H2-7-A	CDRH2	3L7F	B	HIWWDDV	human	IGHV2-5
H2-7-A	CDRH2	3QG7	H	FISSYGT	house mouse	IGHV3-2
H2-7-A	CDRH2	3CFI	C	GINRDGS	llama	IGHV1S6
H2-7-A	CDRH2	3ZKM	H	VIWAGGT	house mouse	IGHV2-9
H2-7-A	CDRH2	3MOD	H	IIYSDDD	human	IGHV2-5

H2-7-A	CDRH2	1C5D	B	AISSGGS	Norway rat	IGHV2S8
H2-7-A	CDRH2	3AAZ	A	HIYWDDD	house mouse	IGHV2-5
H2-7-A	CDRH2	3IDG	B	IIYSDDD	human	IGHV2-5
H2-7-A	CDRH2	3CFI	L	GINRDGS	llama	IGHV1S6
H2-7-A	CDRH2	1XGR	B	YISYSGS	house mouse	IGHV3-8
H2-7-A	CDRH2	2J88	H	HIYWDDD	house mouse	IGHV8-12
H2-7-A	CDRH2	1XGU	B	YISYSGS	house mouse	IGHV3-8
H2-7-A	CDRH2	1YY8	B	VIWSGGN	house mouse	IGHV2-2
H2-7-A	CDRH2	1KIP	B	MIWGDGN	house mouse	IGHV2-6-7
H2-7-A	CDRH2	2EKS	B	YVSYSGS	human	IGHV4-59
H2-7-A	CDRH2	4GAY	A	YISYSGS	house mouse	IGHV3-8
H2-7-A	CDRH2	4IDL	A	SLTTTGT	llama	IGHV1S3
H2-7-A	CDRH2	1DN0	B	EINHSGS	human	IGHV4-34
H2-7-A	CDRH2	1XGT	B	YISYSGS	house mouse	IGHV3-8
H2-7-A	CDRH2	2YSS	B	YVSYSGS	human	IGHV4-59
H2-7-A	CDRH2	3D0V	B	IIYSDDD	human	IGHV2-5
H2-7-A	CDRH2	1I9I	H	SIVSGGN	house mouse	IGHV5S9
H2-7-A	CDRH2	3QHF	H	LIDWDDD	human	IGHV2-70
H2-7-A	CDRH2	1SJV	A	TINSRGI	llama	IGHV1S3
H2-7-A	CDRH2	2DQH	H	YVSYSGS	house mouse	IGHV3-8
H2-7-A	CDRH2	2P8L	B	IIYSDDD	human	IGHV2-5
H2-7-A	CDRH2	1VPO	H	SIVSGGN	house mouse	IGHV5S9
H2-7-A	CDRH2	4GW1	D	VIWSGGN	house mouse	IGHV2-2
H2-7-A	CDRH2	3LEX	H	YINYSGY	house mouse	IGHV3-2
H2-7-A	CDRH2	1DQD	H	YISYSGS	house mouse	IGHV3-8
H2-7-A	CDRH2	4GW5	B	VIWSGGN	house mouse	IGHV2-2
H2-7-A	CDRH2	2R0Z	H	HIWWDDD	house mouse	IGHV8-12
H2-7-A	CDRH2	1ZAN	H	GVWAGGA	Norway rat	IGHV2S41
H2-7-A	CDRH2	1RUM	H	YINYSGF	house mouse	IGHV3-2
H2-7-A	CDRH2	2IQA	H	HIWWDDD	house mouse	IGHV8-12
H2-7-A	CDRH2	2HWZ	H	DIWWDDK	house mouse	IGHV2-70
H2-7-A	CDRH2	1RI8	A	VIDSSGR	Arabian camel	IGHV1S47
H2-7-A	CDRH2	3IDI	B	IIYSDDD	human	IGHV2-5
H2-7-A	CDRH2	1U95	B	IIYSDDD	human	IGHV2-5
H2-7-A	CDRH2	3QG6	B	FISSYGT	house mouse	IGHV3-2
H2-7-A	CDRH2	4NPY	H	YIYYSGS	human	IGHV4-59
H2-7-A	CDRH2	4HS6	H	YISYSGS	house mouse	IGHV4-59
H2-7-B	CDRH2	4JY5	H	YVHDSGD	human	IGHV4-59
H2-7-B	CDRH2	4JY6	B	YVHHSGD	human	IGHV4-59
H2-7-B	CDRH2	4JY4	B	YVHKSGD	human	IGHV4-59
H2-7-B	CDRH2	4FQC	H	YVHKSGD	human	IGHV4-59
H2-8-A	CDRH2	1I8M	H	YINPYNDG	house mouse	IGHV1-14
H2-8-A	CDRH2	1DBA	H	WINIYTGE	house mouse	IGHV9-3-1
H2-8-A	CDRH2	3HC0	A	WIYPGNVH	human	IGHV1-69
H2-8-A	CDRH2	1SHM	B	AINWGSAG	llama	IGHV1S3
H2-8-A	CDRH2	3I75	B	TLDPETGG	house mouse	IGHV1-15
H2-8-A	CDRH2	3B9V	C	SIYPTNGY	human	IGHV3-66
H2-8-A	CDRH2	2VXV	H	FIYPGDSE	human	IGHV5-51
H2-8-A	CDRH2	1KXQ	H	AIYRRTGY	Arabian camel	IGHV1S45
H2-8-A	CDRH2	1HCV	A	AINWDSAR	llama	IGHV

H2-8-A	CDRH2	2O5Z	H	WINPYTGE	house mouse	IGHV9-3-1
H2-8-A	CDRH2	1KXQ	G	AIYRRTGY	Arabian camel	IGHV1S45
H2-8-A	CDRH2	2HG5	A	EIIPSYGR	house mouse	IGHV1S81
H2-8-A	CDRH2	4HXB	H	WIKTNTGE	house mouse	IGHV9-3
H2-8-A	CDRH2	2Q8B	H	RIDPANDN	house mouse	IGHV14-3
H2-8-A	CDRH2	3QEH	G	VINPFRGD	house mouse	IGHV1-46
H2-8-A	CDRH2	3MAC	H	SIIPLFGF	human	IGHV1-69
H2-8-A	CDRH2	2Y5T	A	MIHPSDSE	house mouse	IGHV1-61
H2-8-A	CDRH2	1OP9	A	AIRSSDGT	Arabian camel	IGHV1S45
H2-8-A	CDRH2	1OAY	H	RIDPNGGG	house mouse	IGHV1-72
H2-8-A	CDRH2	2DWE	A	EIIPSYGR	house mouse	IGHV1S81
H2-8-A	CDRH2	3MLU	H	IIYPDDSD	human	IGHV5-51
H2-8-A	CDRH2	4HCR	H	WISVYSGN	house mouse	IGHV1-18
H2-8-A	CDRH2	1UYW	H	GIDPNSGG	house mouse	IGHV1-26
H2-8-A	CDRH2	2G60	H	YIAPAAGA	house mouse	IGHV1-4
H2-8-A	CDRH2	1OAU	H	RIDPNGGG	house mouse	IGHV1-72
H2-8-A	CDRH2	2XQY	G	RIDPSDNE	house mouse	IGHV1S126
H2-8-A	CDRH2	1ZVY	A	ALYTHTGN	Arabian camel	IGHV1S45
H2-8-A	CDRH2	2UYL	W	HISPGSSS	house mouse	IGHV1S61
H2-8-A	CDRH2	3NIF	H	RIDPANGY	house mouse	IGHV14-3
H2-8-A	CDRH2	2FX8	I	GVIPLLTI	human	IGHV1-69
H2-8-A	CDRH2	3S35	H	WLYPESNI	house mouse	IGHV1-71
H2-8-A	CDRH2	3CVI	H	DINPNNGG	house mouse	IGHV1-18
H2-8-A	CDRH2	3S96	A	WINTYSGV	house mouse	IGHV9-3
H2-8-A	CDRH2	2D7T	H	WINPKSGD	human	IGHV1-2
H2-8-A	CDRH2	1MEX	H	YISPGNGD	house mouse	IGHV1S53
H2-8-A	CDRH2	2G75	A	GITPILGI	human	IGHV1-69
H2-8-A	CDRH2	2YMX	H	EMSPSTGR	house mouse	IGHV1-42
H2-8-A	CDRH2	4I1N	B	AISWGGGL	llama	IGHV1S1
H2-8-A	CDRH2	1CZ8	Y	WINTYTGE	human	IGHV3-23
H2-8-A	CDRH2	2VXU	I	DIDPYNGD	house mouse	IGHV1S135
H2-8-A	CDRH2	1YEF	H	RIYPGTGS	house mouse	IGHV1-76
H2-8-A	CDRH2	15C8	H	QIDPANGN	house mouse	IGHV14-3
H2-8-A	CDRH2	3LH2	K	GVIPLLTI	human	IGHV1-69
H2-8-A	CDRH2	3MLW	H	MIYPGDSD	human	IGHV5-51
H2-8-A	CDRH2	1A14	H	IFYPGNGD	house mouse	IGHV1-12
H2-8-A	CDRH2	3BDY	H	RIYPTNGY	human	IGHV3-66
H2-8-A	CDRH2	2NLJ	B	EIIPSYGR	house mouse	IGHV1S81
H2-8-A	CDRH2	3T3M	H	RIDPANGY	house mouse	IGHV14-3
H2-8-A	CDRH2	2VDK	H	RIDPANGY	house mouse	IGHV14-3
H2-8-A	CDRH2	3MLW	I	MIYPGDSD	human	IGHV5-51
H2-8-A	CDRH2	4HKZ	B	RIYPTNGY	human	IGHV3-66
H2-8-A	CDRH2	1YYM	R	RIITILDV	human	IGHV1-69
H2-8-A	CDRH2	1NJ9	B	YIDPSNGD	house mouse	IGHV1S135
H2-8-A	CDRH2	1IT9	H	EIDPSDSY	house mouse	IGHV1-50
H2-8-A	CDRH2	3P9W	F	RIYPTNGY	human	IGHV3-66
H2-8-A	CDRH2	4MA8	H	SIDPSDSY	house mouse	IGHV1S127
H2-8-A	CDRH2	2B1H	H	IFYPGDSD	human	IGHV5-51
H2-8-A	CDRH2	3NFS	H	YINPSTGY	human	IGHV1-46
H2-8-A	CDRH2	1UM5	H	YIYPYNGG	house mouse	IGHV1-14

H2-8-A	CDRH2	1OAR	H	RIDPNGGG	house mouse	IGHV1-72
H2-8-A	CDRH2	2VDQ	H	RIDPANGY	house mouse	IGHV14-3
H2-8-A	CDRH2	4L5F	H	EINPRTGD	house mouse	IGHV1-42
H2-8-A	CDRH2	3WKM	I	RIDPANGN	house mouse	IGHV14-3
H2-8-A	CDRH2	4KI5	C	WINTETGD	house mouse	IGHV9-2-1
H2-8-A	CDRH2	4LLV	E	GVIPLLTI	human	IGHV1-69
H2-8-A	CDRH2	4N9G	H	EMNGNSGY	human	IGHV4-34
H2-8-A	CDRH2	2BOB	A	EIIPSYGR	house mouse	IGHV1S81
H2-8-A	CDRH2	2Y6S	H	EFYPGTDS	house mouse	IGHV1-77
H2-8-A	CDRH2	1RIH	H	YIDPATAY	house mouse	IGHV1-7
H2-8-A	CDRH2	1AD9	B	WIDPGSGN	house mouse	IGHV1-2
H2-8-A	CDRH2	2Y06	H	RIDPNSGG	house mouse	IGHV1-72
H2-8-A	CDRH2	3CMO	H	AIYPGNSD	house mouse	IGHV1-5
H2-8-A	CDRH2	4JZO	C	GFIPTFRT	human	IGHV1-69
H2-8-A	CDRH2	2C1O	B	MIHPSDSE	house mouse	IGHV1-61
H2-8-A	CDRH2	3CLE	H	AIYPGNGD	house mouse	IGHV1-12
H2-8-A	CDRH2	1MHH	D	WVNTETGE	house mouse	IGHV9-2-1
H2-8-A	CDRH2	2YPV	H	IIDPKYGT	house mouse	IGHV1-39
H2-8-A	CDRH2	3QPQ	F	EINPNNGR	house mouse	IGHV1S81
H2-8-A	CDRH2	1OAU	J	RIDPNGGG	house mouse	IGHV1-72
H2-8-A	CDRH2	3K2U	H	GIYPAGGA	human	IGHV3-66
H2-8-A	CDRH2	1AD9	H	WIDPGSGN	house mouse	IGHV1-2
H2-8-A	CDRH2	4D9Q	E	WINTYTGE	human	IGHV7-4-1
H2-8-A	CDRH2	4NZT	H	GISPIFGS	human	IGHV1-69
H2-8-A	CDRH2	1YEG	H	RIYPGTGS	house mouse	IGHV1-76
H2-8-A	CDRH2	1KXQ	E	AIYRRTGY	Arabian camel	IGHV1S45
H2-8-A	CDRH2	1N8Z	B	RIYPTNGY	human	IGHV3-66
H2-8-A	CDRH2	1MRE	H	EIDPSDSY	house mouse	IGHV1-50
H2-8-A	CDRH2	3SE8	H	WIKPLWGA	human	IGHV1-2
H2-8-A	CDRH2	4GW4	H	WINPKTGQ	human	IGHV1-2
H2-8-A	CDRH2	1FGV	H	GINPKNGG	human	IGHV3-74
H2-8-A	CDRH2	1PLG	H	WIYPGSGN	house mouse	IGHV1-84
H2-8-A	CDRH2	4NRZ	H	IIYPGDSD	human	IGHV5-51
H2-8-A	CDRH2	4M61	D	KINPYYGS	house mouse	IGHV1-39
H2-8-A	CDRH2	1N64	H	WVNTETGE	house mouse	IGHV9-2-1
H2-8-A	CDRH2	4JZO	G	GFIPTFRT	human	IGHV1-69
H2-8-A	CDRH2	1MVF	A	AIFIDNGN	Arabian camel	IGHV1S45
H2-8-A	CDRH2	3NAA	H	FIDPSDSY	human	IGHV5-10-1
H2-8-A	CDRH2	2UYL	N	HISPGSSS	house mouse	IGHV1S61
H2-8-A	CDRH2	1TQC	B	WINTFTGE	house mouse	IGHV9-3-1
H2-8-A	CDRH2	3U7Y	H	WLKPRGGA	human	IGHV1-2
H2-8-A	CDRH2	3MLR	H	IIYPDDSD	human	IGHV5-51
H2-8-A	CDRH2	1FVD	B	RIYPTNGY	human	IGHV3-66
H2-8-A	CDRH2	4JPI	H	WINPNSGG	human	IGHV1-2
H2-8-A	CDRH2	2H9G	B	VIYPHDGN	human	IGHV3-66
H2-8-A	CDRH2	1CT8	D	WIYAGSGG	house mouse	IGHV1-66
H2-8-A	CDRH2	4AT6	A	VIYPGNGD	house mouse	IGHV1-12
H2-8-A	CDRH2	3HNV	H	SIYPGNSD	house mouse	IGHV1-5
H2-8-A	CDRH2	3MNV	D	WINTETGE	house mouse	IGHV9-2-1
H2-8-A	CDRH2	4FFV	D	VVIPKYGT	house mouse	IGHV1-39

H2-8-A	CDRH2	1UYW	M	GIDPNSGG	house mouse	IGHV1-26
H2-8-A	CDRH2	2EH7	H	RIYPGDGD	house mouse	IGHV1-82
H2-8-A	CDRH2	2OSL	A	AIYPGNGD	house mouse	IGHV1-12
H2-8-A	CDRH2	1I8M	B	YINPYNDG	house mouse	IGHV1-14
H2-8-A	CDRH2	4LSS	H	WLKPRGGA	human	IGHV1-18
H2-8-A	CDRH2	2W0F	A	EIIPSYGR	house mouse	IGHV1S81
H2-8-A	CDRH2	3MLS	K	IIYPDDSD	human	IGHV5-51
H2-8-A	CDRH2	2NY0	D	RIITILDV	human	IGHV1-69
H2-8-A	CDRH2	2DDQ	H	YINPYNDG	house mouse	IGHV1-14
H2-8-A	CDRH2	1N0X	H	WINPYNGN	human	IGHV1-3
H2-8-A	CDRH2	1A3R	H	RLDPANGY	house mouse	IGHV14-3
H2-8-A	CDRH2	1UWX	M	WINTYTGE	house mouse	IGHV9-3-1
H2-8-A	CDRH2	35C8	H	QIDPANGN	house mouse	IGHV14-3
H2-8-A	CDRH2	1YMH	D	WVNTETGE	house mouse	IGHV9-2-1
H2-8-A	CDRH2	1LK3	H	YINPNSGY	Norway rat	IGHV1S29
H2-8-A	CDRH2	4MSW	A	EIIPSYGR	house mouse	IGHV1S81
H2-8-A	CDRH2	2HVJ	A	EIIPSYGR	house mouse	IGHV1S81
H2-8-A	CDRH2	2BRR	Y	WINTYTGE	house mouse	IGHV9-3-1
H2-8-A	CDRH2	3B9V	A	SIYPTNGY	human	IGHV3-66
H2-8-A	CDRH2	4D9L	K	RIIPIFGI	human	IGHV1-69
H2-8-A	CDRH2	4NM4	I	WINTAIGN	human	IGHV1-3
H2-8-A	CDRH2	4J6R	H	WIKPERGA	human	IGHV1-2
H2-8-A	CDRH2	4N9G	E	EMNGNSGY	human	IGHV4-34
H2-8-A	CDRH2	2Y36	H	RIDPNSGG	house mouse	IGHV1-72
H2-8-A	CDRH2	3QEH	A	VINPFRGD	house mouse	IGHV1-46
H2-8-A	CDRH2	1FJ1	D	WINTETGE	house mouse	IGHV9-2-1
H2-8-A	CDRH2	2PCP	D	YIYPNNGG	house mouse	IGHV1-34
H2-8-A	CDRH2	4AT6	C	VIYPGNGD	house mouse	IGHV1-12
H2-8-A	CDRH2	4F33	H	LITPYNGA	house mouse	IGHV1-37
H2-8-A	CDRH2	4KI5	E	EILPGSGS	house mouse	IGHV1-9
H2-8-A	CDRH2	1FVD	D	RIYPTNGY	human	IGHV3-66
H2-8-A	CDRH2	3UJI	H	IVYPDDSD	human	IGHV5-51
H2-8-A	CDRH2	3RU8	H	WINPYNGN	human	IGHV1-3
H2-8-A	CDRH2	2HVK	A	EIIPSYGR	house mouse	IGHV1S81
H2-8-A	CDRH2	2XQY	J	RIDPSDNE	house mouse	IGHV1S126
H2-8-A	CDRH2	1TPX	B	WINTFTGE	house mouse	IGHV9-3-1
H2-8-A	CDRH2	2HFF	B	WITPSDGN	human	IGHV3-66
H2-8-A	CDRH2	1KN2	H	RIYPGTGS	house mouse	IGHV1-76
H2-8-A	CDRH2	3EO9	H	MIHPSDSE	human	IGHV3-7
H2-8-A	CDRH2	1CFS	B	GIHPGSSG	house mouse	IGHV1-15
H2-8-A	CDRH2	4JZN	I	GFIPTFRT	human	IGHV1-69
H2-8-A	CDRH2	4H20	H	LIIPSNGG	house mouse	IGHV1-19
H2-8-A	CDRH2	3EOA	H	MIHPSDSE	human	IGHV3-7
H2-8-A	CDRH2	4FFY	H	RIDPNSDV	house mouse	IGHV1-72
H2-8-A	CDRH2	1GC1	H	RIITILDV	human	IGHV1-69
H2-8-A	CDRH2	4G7Y	H	SISSYYGY	human	IGHV3-66
H2-8-A	CDRH2	1JV5	B	DIYPGSGI	human	IGHV1-8
H2-8-A	CDRH2	3GI9	H	EIDPSDSY	house mouse	IGHV1-69
H2-8-A	CDRH2	3D85	B	YINPYNDG	house mouse	IGHV1-14
H2-8-A	CDRH2	3ZDZ	H	RIDPANGY	house mouse	IGHV14-3

H2-8-A	CDRH2	3UJJ	H	FIYPADSD	human	IGHV5-51
H2-8-A	CDRH2	3O2D	H	YINPYNDG	human	IGHV1-46
H2-8-A	CDRH2	2JK5	A	EIIPSYGR	house mouse	IGHV1S81
H2-8-A	CDRH2	1E4X	I	EILPSNGR	house mouse	IGHV1S81
H2-8-A	CDRH2	1A6U	H	RIDPNSGG	house mouse	IGHV1-72
H2-8-A	CDRH2	3UJT	H	EIHPNSGN	house mouse	IGHV1S130
H2-8-A	CDRH2	1RHH	B	GIIPIFGT	human	IGHV1-69
H2-8-A	CDRH2	1MFE	H	AIYPGNSA	house mouse	IGHV1-5
H2-8-A	CDRH2	1OAZ	H	RIDPNGGG	house mouse	IGHV1-72
H2-8-A	CDRH2	1FVC	B	RIYPTNGY	human	IGHV3-66
H2-8-A	CDRH2	4IOI	B	RIYPTNGY	human	IGHV3-66
H2-8-A	CDRH2	3MLT	E	IIYPDDSD	human	IGHV5-51
H2-8-A	CDRH2	1DVF	D	RIDPANGN	house mouse	IGHV14-3
H2-8-A	CDRH2	1A6V	H	RIDPNSGG	house mouse	IGHV1-72
H2-8-A	CDRH2	2DD8	H	GITPILGI	human	IGHV1-69
H2-8-A	CDRH2	3PNW	B	SISSSYGS	human	IGHV3-66
H2-8-A	CDRH2	4N9G	A	EMNGNSGY	human	IGHV4-34
H2-8-A	CDRH2	1HH6	B	GIHPGSSG	house mouse	IGHV1-15
H2-8-A	CDRH2	1MFB	H	AIYPGNSA	house mouse	IGHV1-5
H2-8-A	CDRH2	2VDL	H	RIDPANGY	house mouse	IGHV14-3
H2-8-A	CDRH2	4LLV	C	GVIPLLTI	human	IGHV1-69
H2-8-A	CDRH2	3LH2	J	GVIPLLTI	human	IGHV1-69
H2-8-A	CDRH2	1WT5	B	NIYPGSGG	house mouse	IGHV1-2
H2-8-A	CDRH2	3OR7	A	EIIPSYGR	house mouse	IGHV1S81
H2-8-A	CDRH2	1MPA	H	GIYPGNRD	house mouse	IGHV1-5
H2-8-A	CDRH2	2ITD	A	EIIPSYGR	house mouse	IGHV1S81
H2-8-A	CDRH2	1NGP	H	RIDPNSGG	house mouse	IGHV1-72
H2-8-A	CDRH2	1HKL	H	RIDPANVD	house mouse	IGHV14-3
H2-8-A	CDRH2	4AT6	E	VIYPGNGD	house mouse	IGHV1-12
H2-8-A	CDRH2	1EMT	H	RIYPGNGN	house mouse	IGHV1-82
H2-8-A	CDRH2	2HFG	H	WVLPSVGF	human	IGHV3-66
H2-8-A	CDRH2	1U0Q	B	YFGTRGGR	llama	IGHV1S1
H2-8-A	CDRH2	1CR9	H	WIDPENGN	house mouse	IGHV14-4
H2-8-A	CDRH2	1K4C	A	EIIPSYGR	house mouse	IGHV1S81
H2-8-A	CDRH2	4HFW	H	MINPSDGS	human	IGHV1-46
H2-8-A	CDRH2	1GAF	H	RIDPANVD	house mouse	IGHV14-3
H2-8-A	CDRH2	2Q8A	H	RIDPANDN	house mouse	IGHV14-3
H2-8-A	CDRH2	1YEE	H	RIYPGTGS	house mouse	IGHV1-76
H2-8-A	CDRH2	1NGY	B	MIDPNSGG	house mouse	IGHV1S36
H2-8-A	CDRH2	4HFW	B	MINPSDGS	human	IGHV1-46
H2-8-A	CDRH2	4HCR	M	WISVYSGN	house mouse	IGHV1-18
H2-8-A	CDRH2	1MJU	H	NIYPGSSY	house mouse	IGHV1S61
H2-8-A	CDRH2	3PNW	K	SISSSYGS	human	IGHV3-66
H2-8-A	CDRH2	4K9E	H	SIYPYSGY	house mouse	IGHV3-66
H2-8-A	CDRH2	3WKM	H	RIDPANGN	house mouse	IGHV14-3
H2-8-A	CDRH2	1R3J	B	EIIPSYGR	house mouse	IGHV1S81
H2-8-A	CDRH2	4LSR	H	WMNPTNGA	human	IGHV1-2
H2-8-A	CDRH2	4D9R	E	WINTYTGE	human	IGHV7-4-1
H2-8-A	CDRH2	4K3J	H	MIDPSNSD	human	IGHV3-74
H2-8-A	CDRH2	1FGN	H	LIDPENGN	house mouse	IGHV14-1

H2-8-A	CDRH2	3HNT	H	SIYPGNSD	house mouse	IGHV1-5
H2-8-A	CDRH2	4D9L	H	RIIPIFGI	human	IGHV1-69
H2-8-A	CDRH2	2NY4	D	RIITILDV	human	IGHV1-69
H2-8-A	CDRH2	1XF2	B	YINPYNDG	house mouse	IGHV1-14
H2-8-A	CDRH2	1JHL	H	NIYPSDSY	house mouse	IGHV1-61
H2-8-A	CDRH2	4HPO	H	IIYPGDFD	human	IGHV5-51
H2-8-A	CDRH2	3P9W	H	RIYPTNGY	human	IGHV3-66
H2-8-A	CDRH2	1AJ7	H	RIDPANGN	house mouse	IGHV14-3
H2-8-A	CDRH2	3MLT	I	IIYPDDSD	human	IGHV5-51
H2-8-A	CDRH2	1R3K	B	EIIPSYGR	house mouse	IGHV1S81
H2-8-A	CDRH2	3U30	F	SITPSSGQ	human	IGHV3-23
H2-8-A	CDRH2	1A6T	D	RVDPDNGG	house mouse	IGHV1-26
H2-8-A	CDRH2	2I60	H	RIITILDV	human	IGHV1-69
H2-8-A	CDRH2	2W60	A	WINTETGE	house mouse	IGHV9-2-1
H2-8-A	CDRH2	3L95	H	RINPPNRS	human	IGHV3-74
H2-8-A	CDRH2	1OAX	H	RIDPNGGG	house mouse	IGHV1-72
H2-8-A	CDRH2	1MJJ	H	NIYPGSSY	house mouse	IGHV1S61
H2-8-A	CDRH2	1YYM	H	RIITILDV	human	IGHV1-69
H2-8-A	CDRH2	1C5B	H	LIDPSNGR	house mouse	IGHV1-53
H2-8-A	CDRH2	1MH5	H	NIYPDSYR	house mouse	IGHV1S61
H2-8-A	CDRH2	3G04	B	RIDPTDSY	human	IGHV5-10-1
H2-8-A	CDRH2	2E27	H	AIYPGNGD	house mouse	IGHV1-7
H2-8-A	CDRH2	1T3F	B	RIDPSDGE	human	IGHV1-69
H2-8-A	CDRH2	1R3L	B	EIIPSYGR	house mouse	IGHV1S81
H2-8-A	CDRH2	4HWB	H	VIYPGDSY	human	IGHV5-51
H2-8-A	CDRH2	3L95	B	RINPPNRS	human	IGHV3-74
H2-8-A	CDRH2	3MLV	N	IIYPDDSD	human	IGHV5-51
H2-8-A	CDRH2	1N0X	K	WINPYNGN	human	IGHV1-3
H2-8-A	CDRH2	1BJ1	H	WINTYTGE	human	IGHV7-4-1
H2-8-A	CDRH2	1RZJ	H	RIITILDV	human	IGHV1-69
H2-8-A	CDRH2	4DGI	H	SIDPSDSY	house mouse	IGHV1S127
H2-8-A	CDRH2	2H8P	A	EIIPSYGR	house mouse	IGHV1S81
H2-8-A	CDRH2	1JGL	H	WLNTQSGV	house mouse	IGHV9-4
H2-8-A	CDRH2	1BBD	H	RLDPANGY	house mouse	IGHV14-3
H2-8-A	CDRH2	1A4J	H	WINTYTGE	house mouse	IGHV9-3-1
H2-8-A	CDRH2	4C58	B	CIHRQSNL	llama	IGHV
H2-8-A	CDRH2	3PNW	N	SISSSYGS	human	IGHV3-66
H2-8-A	CDRH2	1RJL	B	EIHPNSGN	house mouse	IGHV1S130
H2-8-A	CDRH2	1QFU	H	RIDPYDSE	house mouse	IGHV1-52
H2-8-A	CDRH2	4NRZ	A	IIYPGDSD	human	IGHV5-51
H2-8-A	CDRH2	4JRE	B	WINTSNGY	house mouse	IGHV9-3
H2-8-A	CDRH2	4JZO	D	GFIPTFRT	human	IGHV1-69
H2-8-A	CDRH2	3N9G	H	IINPSGGS	human	IGHV1-46
H2-8-A	CDRH2	1RUR	H	DIYPGGVY	house mouse	IGHV1-63
H2-8-A	CDRH2	1Q0Y	H	EILPGSGD	house mouse	IGHV1-9
H2-8-A	CDRH2	3EFD	H	YISPSYGY	human	IGHV3-66
H2-8-A	CDRH2	12E8	H	WIDPEIGD	house mouse	IGHV14-4
H2-8-A	CDRH2	4AT6	H	VIYPGNGD	house mouse	IGHV1-12
H2-8-A	CDRH2	4EBQ	H	MIDPSESE	house mouse	IGHV1S126
H2-8-A	CDRH2	4LLY	C	DVNPNSGG	human	IGHV3-66

H2-8-A	CDRH2	2DWD	A	EIIPSYGR	house mouse	IGHV1S81
H2-8-A	CDRH2	1YEI	H	RIYPGTGS	house mouse	IGHV1-76
H2-8-A	CDRH2	1F3D	H	YIVPYSGG	house mouse	IGHV1S135
H2-8-A	CDRH2	1JHK	H	WLNTQSGV	house mouse	IGHV9-4
H2-8-A	CDRH2	4GMT	I	EILPEIGM	house mouse	IGHV1-9
H2-8-A	CDRH2	4JJ5	B	WINTYSGV	house mouse	IGHV9-3
H2-8-A	CDRH2	2NY2	D	RIITILDV	human	IGHV1-69
H2-8-A	CDRH2	1P7K	H	YINPYNDG	house mouse	IGHV1-14
H2-8-A	CDRH2	4FZE	H	GITPLLGT	human	IGHV1-69
H2-8-A	CDRH2	1FVC	D	RIYPTNGY	human	IGHV3-66
H2-8-A	CDRH2	3B9V	B	SIYPTNGY	human	IGHV3-66
H2-8-A	CDRH2	1RZ7	H	LIDPEDAD	human	IGHV1-69-2
H2-8-A	CDRH2	4LLW	C	DVNPNSGG	human	IGHV3-66
H2-8-A	CDRH2	4G7V	H	SISSYYGY	human	IGHV3-66
H2-8-A	CDRH2	4GW4	A	WINPKTGQ	human	IGHV1-2
H2-8-A	CDRH2	1YEJ	H	RIYPGTGS	house mouse	IGHV1-76
H2-8-A	CDRH2	2UYL	Y	HISPGSSS	house mouse	IGHV1S61
H2-8-A	CDRH2	1XF4	B	YINPYNDG	house mouse	IGHV1-14
H2-8-A	CDRH2	2HFF	H	WITPSDGN	human	IGHV3-66
H2-8-A	CDRH2	4J8R	D	VINPGSGD	house mouse	IGHV1-54
H2-8-A	CDRH2	3DGG	D	YINPYNDD	house mouse	IGHV1-14
H2-8-A	CDRH2	4NX3	B	RIDPANGH	house mouse	IGHV14-3
H2-8-A	CDRH2	3MNZ	B	WINTETGE	house mouse	IGHV9-2-1
H2-8-A	CDRH2	2NY1	D	RIITILDV	human	IGHV1-69
H2-8-A	CDRH2	3QEH	C	VINPFRGD	house mouse	IGHV1-46
H2-8-A	CDRH2	4LSQ	H	WMNPTNGA	human	IGHV1-2
H2-8-A	CDRH2	2ZPK	I	WINTRSGV	house mouse	IGHV9-4
H2-8-A	CDRH2	2IFF	H	EILPGSGS	house mouse	IGHV1-9
H2-8-A	CDRH2	1VGE	H	WISAGTGN	human	IGHV1-3
H2-8-A	CDRH2	3Q3G	D	RIDPANDK	house mouse	IGHV14-3
H2-8-A	CDRH2	3GNM	H	FISPNTDY	house mouse	IGHV1-7
H2-8-A	CDRH2	1OAX	J	RIDPNGGG	house mouse	IGHV1-72
H2-8-A	CDRH2	1NGX	H	RIDPNSGG	house mouse	IGHV1S36
H2-8-A	CDRH2	4HK0	C	WINPNSGG	human	IGHV1-2
H2-8-A	CDRH2	1FVE	B	RIYPTNGY	human	IGHV3-66
H2-8-A	CDRH2	1BQL	H	EILPGSGS	house mouse	IGHV1-9
H2-8-A	CDRH2	3EO0	D	GVIPIVDI	human	IGHV1-69
H2-8-A	CDRH2	1IQD	B	SFDPESGE	human	IGHV1-24
H2-8-A	CDRH2	3CLF	H	AIYPGNGD	house mouse	IGHV1-12
H2-8-A	CDRH2	4FQH	A	GISPIFGS	human	IGHV1-69
H2-8-A	CDRH2	1J05	H	RIDPANGN	house mouse	IGHV14-3
H2-8-A	CDRH2	1DBJ	H	WINIYTGE	house mouse	IGHV9-3-1
H2-8-A	CDRH2	1XIW	H	LINPYKGV	house mouse	IGHV1-37
H2-8-A	CDRH2	3HNS	H	SIYPGNSD	house mouse	IGHV1-5
H2-8-A	CDRH2	4M61	B	KINPYYGS	house mouse	IGHV1-39
H2-8-A	CDRH2	3CMO	Y	AIYPGNSD	house mouse	IGHV1-5
H2-8-A	CDRH2	1NGX	B	RIDPNSGG	house mouse	IGHV1S36
H2-8-A	CDRH2	4C59	B	CIHRQSNL	llama	IGHV
H2-8-A	CDRH2	1PG7	Z	LVHPHNGA	house mouse	IGHV1-37
H2-8-A	CDRH2	2NY5	H	RIITILDV	human	IGHV1-69

H2-8-A	CDRH2	4K94	H	SIYPYSGY	human	IGHV3-66
H2-8-A	CDRH2	1TZH	B	YIAPSYGY	house mouse	IGHV5-17
H2-8-A	CDRH2	1RHH	D	GIIPIFGT	human	IGHV1-69
H2-8-A	CDRH2	1TZG	H	GVIPLLTI	human	IGHV1-69
H2-8-A	CDRH2	4HQQ	H	IIYPGDFD	human	IGHV5-51
H2-8-A	CDRH2	1RZF	H	GIIPIFGS	human	IGHV1-69
H2-8-A	CDRH2	4M43	H	DISPYYGS	house mouse	IGHV1-18
H2-8-A	CDRH2	1S5H	B	EIIPSYGR	house mouse	IGHV1S81
H2-8-A	CDRH2	1CZ8	H	WINTYTGE	human	IGHV3-23
H2-8-A	CDRH2	1A4K	H	WINTYTGE	house mouse	IGHV9-3-1
H2-8-A	CDRH2	1A3L	H	DIYPGGVY	house mouse	IGHV1-63
H2-8-A	CDRH2	3P9W	B	RIYPTNGY	human	IGHV3-66
H2-8-A	CDRH2	2VL5	C	MIHPSDSE	house mouse	IGHV1-61
H2-8-A	CDRH2	2XWT	A	IIYPGDSD	human	IGHV5-51
H2-8-A	CDRH2	3LH2	H	GVIPLLTI	human	IGHV1-69
H2-8-A	CDRH2	3MXW	H	VIRPYSGE	human	IGHV1-2
H2-8-A	CDRH2	1FJ1	B	WINTETGE	house mouse	IGHV9-2-1
H2-8-A	CDRH2	1UWX	H	WINTYTGE	house mouse	IGHV9-3-1
H2-8-A	CDRH2	4N9G	M	EMNGNSGY	human	IGHV4-34
H2-8-A	CDRH2	4D9R	H	WINTYTGE	human	IGHV7-4-1
H2-8-A	CDRH2	3PGF	H	YISSYSGY	human	IGHV3-66
H2-8-A	CDRH2	3HC3	H	WIYPGNVH	human	IGHV1-3
H2-8-A	CDRH2	1JPS	H	LIDPEQGN	human	IGHV3-66
H2-8-A	CDRH2	3U7W	H	WLKPRGGA	human	IGHV1-2
H2-8-A	CDRH2	2NXZ	D	RIITILDV	human	IGHV1-69
H2-8-A	CDRH2	3EOA	B	MIHPSDSE	human	IGHV3-7
H2-8-A	CDRH2	3FCT	B	MIDPNSGG	house mouse	IGHV1S36
H2-8-A	CDRH2	2R0L	H	WIYPTGGA	human	IGHV3-66
H2-8-A	CDRH2	3DGG	B	YINPYNDD	house mouse	IGHV1-14
H2-8-A	CDRH2	3NAC	H	RIDPSDSY	human	IGHV5-10-1
H2-8-A	CDRH2	3ZDY	H	RIDPANGY	house mouse	IGHV14-3
H2-8-A	CDRH2	3R0M	B	AISWSGGT	human	IGHV3-23
H2-8-A	CDRH2	3NIG	H	RIDPANGY	house mouse	IGHV14-3
H2-8-A	CDRH2	2NY6	D	RIITILDV	human	IGHV1-69
H2-8-A	CDRH2	1TQB	B	WINTFTGE	house mouse	IGHV9-3-1
H2-8-A	CDRH2	2XA3	A	AISWSGGT	llama	IGHV1S3
H2-8-A	CDRH2	3Q3G	B	RIDPANDK	house mouse	IGHV14-3
H2-8-A	CDRH2	1NGQ	H	RIDPNSGG	house mouse	IGHV1-72
H2-8-A	CDRH2	1NLB	H	WVNTETGE	house mouse	IGHV9-2-1
H2-8-A	CDRH2	4ETQ	A	MIDPSESE	house mouse	IGHV1S126
H2-8-A	CDRH2	4NRX	A	IIYPGDSD	human	IGHV5-51
H2-8-A	CDRH2	2UYL	B	HISPGSSS	house mouse	IGHV1S61
H2-8-A	CDRH2	2A6I	B	YINPGNGY	house mouse	IGHV1-58
H2-8-A	CDRH2	4F3F	B	LITPYNGA	house mouse	IGHV1-37
H2-8-A	CDRH2	1HI6	B	GIHPGSSG	house mouse	IGHV1-15
H2-8-A	CDRH2	4HJG	B	RIYPTNGY	human	IGHV3-66
H2-8-A	CDRH2	2NXY	D	RIITILDV	human	IGHV1-69
H2-8-A	CDRH2	3VW3	H	TIYPGNSD	house mouse	IGHV1-5
H2-8-A	CDRH2	4JPK	H	WINPNSGG	human	IGHV1-2
H2-8-A	CDRH2	3I9G	H	AISPRHDI	house mouse	IGHV1-78

H2-8-A	CDRH2	2ZPK	H	WINTRSGV	house mouse	IGHV9-4
H2-8-A	CDRH2	3EO0	B	GVIPIVDI	human	IGHV1-69
H2-8-A	CDRH2	1NCA	H	WINTNTGE	house mouse	IGHV9-3
H2-8-A	CDRH2	2I60	R	RIITILDV	human	IGHV1-69
H2-8-A	CDRH2	1N4X	I	WINTETDE	house mouse	IGHV9-2-1
H2-8-A	CDRH2	3QCV	I	LINPGSDY	house mouse	IGHV5-51
H2-8-A	CDRH2	1A4J	B	WINTYTGE	house mouse	IGHV9-3-1
H2-8-A	CDRH2	1UJ3	B	GNDPANGH	human	IGHV1-46
H2-8-A	CDRH2	1MFA	H	AIYPGNSA	house mouse	IGHV1-5
H2-8-A	CDRH2	1IQW	H	EIDPSDSY	house mouse	IGHV1-50
H2-8-A	CDRH2	3P0Y	H	EISAAGGY	human	IGHV3-66
H2-8-A	CDRH2	2XQB	H	WISAFNGY	human	IGHV1-18
H2-8-A	CDRH2	4MA7	H	SIDPSDSY	house mouse	IGHV1S127
H2-8-A	CDRH2	4JR9	H	WINTSNGY	house mouse	IGHV9-3
H2-8-A	CDRH2	2XRA	H	GIMPIFDI	human	IGHV1-69
H2-8-A	CDRH2	3PNW	H	SISSSYGS	human	IGHV3-66
H2-8-A	CDRH2	4KUC	H	NIYPGSGR	house mouse	IGHV1S61
H2-8-A	CDRH2	1XIW	D	LINPYKGV	house mouse	IGHV1-37
H2-8-A	CDRH2	4JHA	H	GIIPVLGT	human	IGHV1-69
H2-8-A	CDRH2	3PP4	H	RIFPGDGD	house mouse	IGHV1-69
H2-8-A	CDRH2	3QEH	E	VINPFRGD	house mouse	IGHV1-46
H2-8-A	CDRH2	4IMK	A	WINPNSGG	human	IGHV1-2
H2-8-A	CDRH2	1MIM	H	AIYPGNSD	human	IGHV1-5
H2-8-A	CDRH2	3STL	A	EIIPSYGR	house mouse	IGHV1S81
H2-8-A	CDRH2	4LEO	A	WIYAGTGS	human	IGHV1-18
H2-8-A	CDRH2	4LLU	A	DVNPNSGG	human	IGHV3-66
H2-8-A	CDRH2	3B9V	D	SIYPTNGY	human	IGHV3-66
H2-8-A	CDRH2	3BKY	H	AIYPGNGD	house mouse	IGHV1-12
H2-8-A	CDRH2	2VDO	H	RIDPANGY	house mouse	IGHV14-3
H2-8-A	CDRH2	3MLT	B	IIYPDDSD	human	IGHV5-51
H2-8-A	CDRH2	2O5X	H	WINPYTGE	house mouse	IGHV9-3-1
H2-8-A	CDRH2	1MHH	B	WVNTETGE	house mouse	IGHV9-2-1
H2-8-A	CDRH2	2FR4	B	YINPYNDG	house mouse	IGHV1-14
H2-8-A	CDRH2	1L7I	H	DVNPNSGG	human	IGHV3-66
H2-8-A	CDRH2	2W65	A	WINTETGE	house mouse	IGHV9-2-1
H2-8-A	CDRH2	2C1P	H	MIHPSDSE	house mouse	IGHV1-61
H2-8-A	CDRH2	2NY3	D	RIITILDV	human	IGHV1-69
H2-8-A	CDRH2	2MPA	H	GIYPGNRD	house mouse	IGHV1-5
H2-8-A	CDRH2	1OAQ	H	RIDPNGGG	house mouse	IGHV1-72
H2-8-A	CDRH2	1A6T	B	RVDPDNGG	house mouse	IGHV1-26
H2-8-A	CDRH2	1A4K	B	WINTYTGE	house mouse	IGHV9-3-1
H2-8-A	CDRH2	4LSU	H	WMNPQWGQ	human	IGHV1-2
H2-8-A	CDRH2	3NCJ	H	FIDPSDSY	human	IGHV5-10-1
H2-8-A	CDRH2	2FX7	H	GVIPLLTI	human	IGHV1-69
H2-8-A	CDRH2	4KTE	H	SIYSSSGS	rhesus monkey	IGHV4-2
H2-8-A	CDRH2	1NCB	H	WINTNTGE	house mouse	IGHV9-3
H2-8-A	CDRH2	1KEG	H	TIYPGNSD	house mouse	IGHV1-5
H2-8-A	CDRH2	1OAR	J	RIDPNGGG	house mouse	IGHV1-72
H2-8-A	CDRH2	3PNW	T	SISSSYGS	human	IGHV3-66
H2-8-A	CDRH2	3NTC	H	DIYPGGGY	house mouse	IGHV1-63

H2-8-A	CDRH2	1HH9	B	GIHPGSSG	house mouse	IGHV1-15
H2-8-A	CDRH2	2QQK	H	YIYPDSGY	human	IGHV3-66
H2-8-A	CDRH2	1AXS	H	EILPGSGG	house mouse	IGHV1-9
H2-8-A	CDRH2	1MJJ	B	NIYPGSSY	house mouse	IGHV1S61
H2-8-A	CDRH2	1MFC	H	AIYPGNSA	house mouse	IGHV1-5
H2-8-A	CDRH2	1N4X	H	WINTETDE	house mouse	IGHV9-2-1
H2-8-A	CDRH2	4HWE	H	VIYPGDSY	human	IGHV5-51
H2-8-A	CDRH2	1TZI	B	DIAPYAGA	house mouse	IGHV5-17
H2-8-A	CDRH2	2FX8	J	GVIPLLTI	human	IGHV1-69
H2-8-A	CDRH2	4AL8	H	NIYPGSGT	human	IGHV1-69
H2-8-A	CDRH2	1Q0X	H	EILPGSGD	house mouse	IGHV1-9
H2-8-A	CDRH2	3W9D	C	RTIPLFGK	human	IGHV1-69
H2-8-A	CDRH2	2PCP	B	YIYPNNGG	house mouse	IGHV1-34
H2-8-A	CDRH2	1SHM	D	AINWGSAG	llama	IGHV1S3
H2-8-A	CDRH2	1JN6	B	AIYPGDDD	house mouse	IGHV1-5
H2-8-A	CDRH2	4F33	F	LITPYNGA	house mouse	IGHV1-37
H2-8-A	CDRH2	1RZ8	B	RIITILDV	human	IGHV1-69
H2-8-A	CDRH2	3U0T	B	RIDPATGN	house mouse	IGHV1-46
H2-8-A	CDRH2	4AMK	H	VIYPGNGD	house mouse	IGHV1-12
H2-8-A	CDRH2	4FQJ	H	WISGYSGD	human	IGHV1-18
H2-8-A	CDRH2	1CFQ	B	GIHPGSSG	house mouse	IGHV1S52
H2-8-A	CDRH2	1XGY	H	WIFPRNGN	house mouse	IGHV1-75
H2-8-A	CDRH2	4JZN	A	GFIPTFRT	human	IGHV1-69
H2-8-A	CDRH2	2P7T	A	EIIPSYGR	house mouse	IGHV1S81
H2-8-A	CDRH2	2BDN	H	RIDPANGN	house mouse	IGHV14-3
H2-8-A	CDRH2	4LLV	A	GVIPLLTI	human	IGHV1-69
H2-8-A	CDRH2	4LLY	A	DVNPNSGG	human	IGHV3-66
H2-8-A	CDRH2	2I5Y	R	RIITILDV	human	IGHV1-69
H2-8-A	CDRH2	1QBM	H	RIDPASGN	house mouse	IGHV14-3
H2-8-A	CDRH2	4JDV	A	WINPNSGG	human	IGHV1-2
H2-8-A	CDRH2	3T2N	I	WINTETGS	human	IGHV7-4-1
H2-8-A	CDRH2	3MXV	H	VIRPYSGE	house mouse	IGHV1-67
H2-8-A	CDRH2	2B0S	H	IFYPGDSD	human	IGHV5-51
H2-8-A	CDRH2	1YYL	H	RIITILDV	human	IGHV1-69
H2-8-A	CDRH2	2FX8	H	GVIPLLTI	human	IGHV1-69
H2-8-A	CDRH2	3EBA	A	AIRSSDGT	Arabian camel	IGHV1S50
H2-8-A	CDRH2	4FFV	H	VVIPKYGT	house mouse	IGHV1-39
H2-8-A	CDRH2	2C1P	B	MIHPSDSE	house mouse	IGHV1-61
H2-8-A	CDRH2	1NMC	H	IFYPGNGD	house mouse	IGHV1-12
H2-8-A	CDRH2	2CMR	H	GIIPIFGT	human	IGHV1-69
H2-8-A	CDRH2	1ZEA	H	WINTYSGV	house mouse	IGHV9-3
H2-8-A	CDRH2	1YYL	R	RIITILDV	human	IGHV1-69
H2-8-A	CDRH2	1XF5	D	WVNTETGE	house mouse	IGHV9-2-1
H2-8-A	CDRH2	3GI8	H	EIDPSDSY	house mouse	IGHV1-69
H2-8-A	CDRH2	1N7M	L	RIDPNSGG	house mouse	IGHV1S36
H2-8-A	CDRH2	1JPT	H	LIDPEQGN	human	IGHV3-66
H2-8-A	CDRH2	4LLU	C	DVNPNSGG	human	IGHV3-66
H2-8-A	CDRH2	1OAZ	J	RIDPNGGG	house mouse	IGHV1-72
H2-8-A	CDRH2	3PNW	E	SISSSYGS	human	IGHV3-66
H2-8-A	CDRH2	4JRE	H	WINTSNGY	house mouse	IGHV9-3

H2-8-A	CDRH2	1YEH	H	RIYPGTGS	house mouse	IGHV1-76
H2-8-A	CDRH2	4GRW	G	GIDSGDGS	llama	IGHV1S1
H2-8-A	CDRH2	4HS8	H	DIYPSDSF	house mouse	IGHV3-23
H2-8-A	CDRH2	4ETQ	H	MIDPSESE	house mouse	IGHV1S126
H2-8-A	CDRH2	1HZH	K	WINPYNGN	human	IGHV1-3
H2-8-A	CDRH2	3T3P	H	RIDPANGY	house mouse	IGHV14-3
H2-8-A	CDRH2	3S96	C	WINTYSGV	house mouse	IGHV9-3
H2-8-A	CDRH2	2HFE	A	EIIPSYGR	house mouse	IGHV1S81
H2-8-A	CDRH2	1MNU	H	GIYPGNRD	house mouse	IGHV1-5
H2-8-A	CDRH2	4JZO	A	GFIPTFRT	human	IGHV1-69
H2-8-A	CDRH2	4ISV	B	WINTYSGV	house mouse	IGHV9-3
H2-8-A	CDRH2	3Q3G	H	RIDPANDK	house mouse	IGHV14-3
H2-8-A	CDRH2	3NA9	H	FIDPSDSY	human	IGHV5-10-1
H2-8-A	CDRH2	3ZE2	E	RIDPANGY	house mouse	IGHV14-3
H2-8-A	CDRH2	3P9W	D	RIYPTNGY	human	IGHV3-66
H2-8-A	CDRH2	3U0T	D	RIDPATGN	house mouse	IGHV1-46
H2-8-A	CDRH2	4DCQ	B	WINTYSGV	house mouse	IGHV9-3
H2-8-A	CDRH2	3LHP	H	GVIPLLTI	human	IGHV1-69
H2-8-A	CDRH2	1NMC	B	IFYPGNGD	house mouse	IGHV1-12
H2-8-A	CDRH2	1FVE	D	RIYPTNGY	human	IGHV3-66
H2-8-A	CDRH2	1TZH	H	YIAPSYGY	house mouse	IGHV5-17
H2-8-A	CDRH2	3MNW	B	WINTETGE	house mouse	IGHV9-2-1
H2-8-A	CDRH2	4JDV	H	WINPNSGG	human	IGHV1-2
H2-8-A	CDRH2	1KN4	H	RIYPGTGS	house mouse	IGHV1-76
H2-8-A	CDRH2	2I5Y	H	RIITILDV	human	IGHV1-69
H2-8-A	CDRH2	2Y6S	D	EFYPGTDS	house mouse	IGHV1-77
H2-8-A	CDRH2	1GHF	H	WIQTNTEE	house mouse	IGHV9-3
H2-8-A	CDRH2	1J05	B	RIDPANGN	house mouse	IGHV14-3
H2-8-A	CDRH2	1SY6	H	YINPSRGY	house mouse	IGHV1-4
H2-8-A	CDRH2	1U0Q	A	YFGTRGGR	llama	IGHV1S1
H2-8-A	CDRH2	4GMT	H	EILPEIGM	house mouse	IGHV1-9
H2-8-A	CDRH2	1IL1	A	WLDPENGD	house mouse	IGHV14-4
H2-8-A	CDRH2	4AT6	J	VIYPGNGD	house mouse	IGHV1-12
H2-8-A	CDRH2	1XF2	H	YINPYNDG	house mouse	IGHV1-14
H2-8-A	CDRH2	1R3I	H	EIIPSYGR	house mouse	IGHV1S81
H2-8-A	CDRH2	1P7K	B	YINPYNDG	house mouse	IGHV1-14
H2-8-A	CDRH2	3PP3	I	RIFPGDGD	house mouse	IGHV1-69
H2-8-A	CDRH2	4KUC	F	NIYPGSGR	house mouse	IGHV1S61
H2-8-A	CDRH2	1WEJ	H	RIDPASGN	house mouse	IGHV14-3
H2-8-A	CDRH2	3MA9	H	SIIPIFGT	human	IGHV1-69
H2-8-A	CDRH2	3MO1	B	WINTETGE	house mouse	IGHV9-2-1
H2-8-A	CDRH2	1SHM	E	AINWGSAG	llama	IGHV1S3
H2-8-A	CDRH2	2VDR	H	RIDPANGY	house mouse	IGHV14-3
H2-8-A	CDRH2	3QCT	H	LINPGSDY	house mouse	IGHV5-51
H2-8-A	CDRH2	2VXU	H	DIDPYNGD	house mouse	IGHV1S135
H2-8-A	CDRH2	4AT6	O	VIYPGNGD	house mouse	IGHV1-12
H2-8-A	CDRH2	3E8U	H	WINTHTGE	house mouse	IGHV9-1
H2-8-A	CDRH2	2FR4	H	YINPYNDG	house mouse	IGHV1-14
H2-8-A	CDRH2	1NCC	H	WINTNTGE	house mouse	IGHV9-3
H2-8-A	CDRH2	3ZDX	H	RIDPANGY	house mouse	IGHV14-3

H2-8-A	CDRH2	4EOW	H	WINTRSGV	human	IGHV7-4-1
H2-8-A	CDRH2	1I3G	H	NIYPSDSY	house mouse	IGHV1-61
H2-8-A	CDRH2	1FOR	H	QIYPGDGD	house mouse	IGHV1-80
H2-8-A	CDRH2	4I13	B	AISWGGGL	llama	IGHV1S1
H2-8-A	CDRH2	1XGY	I	WIFPRNGN	house mouse	IGHV1-75
H2-8-A	CDRH2	3LH2	I	GVIPLLTI	human	IGHV1-69
H2-8-A	CDRH2	3LHP	I	GVIPLLTI	human	IGHV1-69
H2-8-A	CDRH2	2EH8	H	RIYPGDGD	house mouse	IGHV1-82
H2-8-A	CDRH2	3TPK	A	GISLRKGW	Arabian camel	IGHV1S50
H2-8-A	CDRH2	1MVF	B	AIFIDNGN	Arabian camel	IGHV1S45
H2-8-A	CDRH2	1A6V	I	RIDPNSGG	house mouse	IGHV1-72
H2-8-A	CDRH2	2XZQ	H	RIDPNSGG	house mouse	IGHV1-72
H2-8-A	CDRH2	2G2R	B	VINPYNGF	house mouse	IGHV1-19
H2-8-A	CDRH2	1XF5	B	WVNTETGE	house mouse	IGHV9-2-1
H2-8-A	CDRH2	3STZ	A	EIIPSYGR	house mouse	IGHV1S81
H2-8-A	CDRH2	2G75	C	GITPILGI	human	IGHV1-69
H2-8-A	CDRH2	3OR6	A	EIIPSYGR	house mouse	IGHV1S81
H2-8-A	CDRH2	3QPQ	H	EINPNNGR	house mouse	IGHV1S81
H2-8-A	CDRH2	1BOG	B	GIHPGSSG	house mouse	IGHV1S52
H2-8-A	CDRH2	2ATK	A	EIIPSYGR	house mouse	IGHV1S81
H2-8-A	CDRH2	2VDP	H	RIDPANGY	house mouse	IGHV14-3
H2-8-A	CDRH2	1D5B	H	EILPGSGG	house mouse	IGHV1-9
H2-8-A	CDRH2	3PNW	Q	SISSSYGS	human	IGHV3-66
H2-8-A	CDRH2	1YEK	H	RIYPGTGS	house mouse	IGHV1-76
H2-8-A	CDRH2	3T2N	H	WINTETGS	human	IGHV7-4-1
H2-8-A	CDRH2	2ADF	H	WKNTNTGE	house mouse	IGHV9-3
H2-8-A	CDRH2	4FQH	H	GISPIFGS	human	IGHV1-69
H2-8-A	CDRH2	3W9E	A	RTIPLFGK	house mouse	IGHV1-69
H2-8-A	CDRH2	1D5B	B	EILPGSGG	house mouse	IGHV1-9
H2-8-A	CDRH2	3PP3	H	RIFPGDGD	house mouse	IGHV1-69
H2-8-A	CDRH2	2VXT	H	DIDPYNGD	house mouse	IGHV1S135
H2-8-A	CDRH2	2V7H	H	EILPGSGS	house mouse	IGHV1-9
H2-8-A	CDRH2	1XF3	B	YINPYNDG	house mouse	IGHV1-14
H2-8-A	CDRH2	1WT5	A	NIYPGSGG	house mouse	IGHV1-2
H2-8-A	CDRH2	1NMB	H	IFYPGNGD	house mouse	IGHV1-12
H2-8-A	CDRH2	3QCU	I	LINPGSDY	house mouse	IGHV5-51
H2-8-A	CDRH2	3MLS	J	IIYPDDSD	human	IGHV5-51
H2-8-A	CDRH2	2B1A	H	IFYPGDSD	human	IGHV5-51
H2-8-A	CDRH2	3QPQ	J	EINPNNGR	house mouse	IGHV1S81
H2-8-A	CDRH2	3GKW	H	GINPTSGG	house mouse	IGHV1-69
H2-8-A	CDRH2	1MJ8	H	NIYPGSGY	house mouse	IGHV1S61
H2-8-A	CDRH2	3HC4	H	WIYPGNGH	human	IGHV1-3
H2-8-A	CDRH2	2BRR	H	WINTYTGE	house mouse	IGHV9-3-1
H2-8-A	CDRH2	3QOT	H	GIIPIFGT	human	IGHV1-69
H2-8-A	CDRH2	4AT6	M	VIYPGNGD	house mouse	IGHV1-12
H2-8-A	CDRH2	2FX8	K	GVIPLLTI	human	IGHV1-69
H2-8-A	CDRH2	1JFQ	H	YNNPGNGY	house mouse	IGHV1-58
H2-8-A	CDRH2	2IH1	A	EIIPSYGR	house mouse	IGHV1S81
H2-8-A	CDRH2	4F33	B	LITPYNGA	house mouse	IGHV1-37
H2-8-A	CDRH2	2JEL	H	LISTYSGY	house mouse	IGHV1-67

H2-8-A	CDRH2	1MFD	H	AIYPGNSA	house mouse	IGHV1-5
H2-8-A	CDRH2	4NRX	D	IIYPGDSD	human	IGHV5-51
H2-8-A	CDRH2	2W65	C	WINTETGE	house mouse	IGHV9-2-1
H2-8-A	CDRH2	1KB5	H	NIDPYYGG	house mouse	IGHV1-39
H2-8-A	CDRH2	3DVG	B	YITPYYGS	human	IGHV3-66
H2-8-A	CDRH2	3SOB	H	WITPYGGY	human	IGHV3-11
H2-8-A	CDRH2	2OSL	H	AIYPGNGD	house mouse	IGHV1-12
H2-8-A	CDRH2	3U30	C	SITPSSGQ	human	IGHV3-23
H2-8-A	CDRH2	1NGZ	B	RIDPNSGG	house mouse	IGHV1S36
H2-8-A	CDRH2	4HKX	A	WIHPNSGA	human	IGHV1-2
H2-8-A	CDRH2	1DBB	H	WINIYTGE	house mouse	IGHV9-3-1
H2-8-A	CDRH2	1E6O	H	YINPSSGY	house mouse	IGHV1-4
H2-8-A	CDRH2	1C5C	H	LIDPSNGR	house mouse	IGHV1-53
H2-8-A	CDRH2	3NID	H	RIDPANGY	house mouse	IGHV14-3
H2-8-A	CDRH2	3OJD	B	WINTYTGE	house mouse	IGHV9-1
H2-8-A	CDRH2	2FX9	H	GVIPLLTI	human	IGHV1-69
H2-8-A	CDRH2	3Q3G	K	RIDPANDK	house mouse	IGHV14-3
H2-8-A	CDRH2	1IGI	H	YISPYSGV	house mouse	IGHV1-34
H2-8-A	CDRH2	4NRX	H	IIYPGDSD	human	IGHV5-51
H2-8-A	CDRH2	1A6W	H	RIDPNSGG	house mouse	IGHV1-72
H2-8-A	CDRH2	1PG7	X	LVHPHNGA	house mouse	IGHV1-37
H2-8-A	CDRH2	4D9L	J	RIIPIFGI	human	IGHV1-69
H2-8-A	CDRH2	2H9G	H	VIYPHDGN	human	IGHV3-66
H2-8-A	CDRH2	3O2V	H	WINPYTGE	house mouse	IGHV9-3-1
H2-8-A	CDRH2	1XF3	H	YINPYNDG	house mouse	IGHV1-14
H2-8-A	CDRH2	4ALA	H	NIYPGSGT	house mouse	IGHV1-55
H2-8-A	CDRH2	1CT8	B	WIYAGSGG	house mouse	IGHV1-66
H2-8-A	CDRH2	1E4X	H	EILPSNGR	house mouse	IGHV1S81
H2-8-A	CDRH2	4LSP	H	WMNPTNGA	human	IGHV1-2
H2-8-A	CDRH2	1F3D	K	YIVPYSGG	house mouse	IGHV1S135
H2-8-A	CDRH2	4DN3	H	GIIPIFGT	human	IGHV1-69
H2-8-A	CDRH2	1EGJ	H	DINPSNGG	house mouse	IGHV1-26
H2-8-A	CDRH2	3QPQ	D	EINPNNGR	house mouse	IGHV1S81
H2-8-A	CDRH2	2Y07	H	RIDPNSGG	house mouse	IGHV1-72
H2-8-A	CDRH2	1EAP	B	YISPGNGD	house mouse	IGHV1S53
H2-8-A	CDRH2	4GRW	E	GIDSGDGS	llama	IGHV1S1
H2-8-A	CDRH2	2NY7	H	WINPYNGN	human	IGHV1-3
H2-8-A	CDRH2	4HT1	H	TVYVRQGT	rabbit	IGHV1S7
H2-8-A	CDRH2	3PNW	W	SISSSYGS	human	IGHV3-66
H2-8-A	CDRH2	1UM6	H	YIYPYNGG	house mouse	IGHV1-14
H2-8-A	CDRH2	1RZ8	D	RIITILDV	human	IGHV1-69
H2-8-A	CDRH2	1BJ1	K	WINTYTGE	human	IGHV7-4-1
H2-8-A	CDRH2	1MH5	B	NIYPDSYR	house mouse	IGHV1S61
H2-8-A	CDRH2	1G9M	H	RIITILDV	human	IGHV1-69
H2-8-A	CDRH2	4D9Q	H	WINTYTGE	human	IGHV7-4-1
H2-8-A	CDRH2	1UM4	H	YIYPYNGG	house mouse	IGHV1-14
H2-8-A	CDRH2	3MLT	H	IIYPDDSD	human	IGHV5-51
H2-8-A	CDRH2	1OAY	J	RIDPNGGG	house mouse	IGHV1-72
H2-8-A	CDRH2	1AXS	B	EILPGSGG	house mouse	IGHV1-9
H2-8-A	CDRH2	2WUC	H	GIYPAGGA	human	IGHV3-66

H2-8-A	CDRH2	25C8	H	QIDPANGN	house mouse	IGHV14-3
H2-8-A	CDRH2	2C1O	H	MIHPSDSE	house mouse	IGHV1-61
H2-8-A	CDRH2	2IH3	A	EIIPSYGR	house mouse	IGHV1S81
H2-8-A	CDRH2	1TET	H	WINTYSGV	house mouse	IGHV9-3
H2-8-A	CDRH2	1XF4	H	YINPYNDG	house mouse	IGHV1-14
H2-8-A	CDRH2	3MLV	H	IIYPDDSD	human	IGHV5-51
H2-8-A	CDRH2	4LLW	A	DVNPNSGG	human	IGHV3-66
H2-8-A	CDRH2	1QBL	H	RIDPASGN	house mouse	IGHV14-3
H2-8-A	CDRH2	3R0M	A	AISWSGGT	human	IGHV3-23
H2-8-A	CDRH2	2VL5	A	MIHPSDSE	house mouse	IGHV1-61
H2-8-A	CDRH2	1RUQ	H	DIYPGGVY	house mouse	IGHV1-63
H2-8-A	CDRH2	1YEC	H	RIYPGTGS	house mouse	IGHV1-76
H2-8-A	CDRH2	4K23	H	EINPYNGG	house mouse	IGHV1-43
H2-8-A	CDRH2	3NID	E	RIDPANGY	house mouse	IGHV14-3
H2-8-A	CDRH2	1I7Z	D	WINTYTGE	house mouse	IGHV9-3-1
H2-8-A	CDRH2	1CIC	B	NIYPGSGD	house mouse	IGHV1S5
H2-8-A	CDRH2	1E4W	H	EILPSNGR	house mouse	IGHV1S81
H2-8-A	CDRH2	3UJT	I	EIHPNSGN	house mouse	IGHV1S130
H2-8-A	CDRH2	4JPI	A	WINPNSGG	human	IGHV1-2
H2-8-A	CDRH2	3MLS	H	IIYPDDSD	human	IGHV5-51
H2-8-A	CDRH2	3MLS	I	IIYPDDSD	human	IGHV5-51
H2-8-A	CDRH2	1ZTX	H	DILCGTGR	house mouse	IGHV1-9
H2-8-A	CDRH2	3MNV	B	WINTETGE	house mouse	IGHV9-2-1
H2-8-A	CDRH2	1MLB	B	EILPGSGS	house mouse	IGHV1-9
H2-8-A	CDRH2	1LK3	I	YINPNSGY	Norway rat	IGHV1S29
H2-8-A	CDRH2	4D9L	I	RIIPIFGI	human	IGHV1-69
H2-8-A	CDRH2	1CFT	B	GIHPGSSG	house mouse	IGHV1S52
H2-8-A	CDRH2	1YMH	B	WVNTETGE	house mouse	IGHV9-2-1
H2-8-A	CDRH2	1SHM	A	AINWGSAG	llama	IGHV1S3
H2-8-A	CDRH2	2FX9	I	GVIPLLTI	human	IGHV1-69
H2-8-A	CDRH2	4H88	H	SIDPSDSY	house mouse	IGHV1S127
H2-8-A	CDRH2	4KTD	H	GISGSSGD	rhesus monkey	IGHV4-2
H2-8-A	CDRH2	4LLV	H	GVIPLLTI	human	IGHV1-69
H2-8-A	CDRH2	1CFN	B	GIHPGSSG	house mouse	IGHV1S52
H2-8-A	CDRH2	2RCS	H	RIDPANGN	house mouse	IGHV1-3
H2-8-A	CDRH2	12E8	P	WIDPEIGD	house mouse	IGHV14-4
H2-8-A	CDRH2	1K4D	A	EIIPSYGR	house mouse	IGHV1S81
H2-8-A	CDRH2	3T3P	E	RIDPANGY	house mouse	IGHV14-3
H2-8-A	CDRH2	2G2R	H	VINPYNGF	house mouse	IGHV1-19
H2-8-A	CDRH2	1KXQ	F	AIYRRTGY	Arabian camel	IGHV1S45
H2-8-A	CDRH2	1K6Q	H	WIDPENGN	house mouse	IGHV14-1
H2-8-B	CDRH2	3ZHL	A	LISGSGGS	human	IGHV3-23
H2-8-B	CDRH2	4NCC	H	QINPHSST	house mouse	IGHV4-1
H2-8-B	CDRH2	2X1P	B	SISRSGTL	llama	IGHV
H2-8-B	CDRH2	3ZHK	B	AISGSGGS	human	IGHV3-23
H2-8-B	CDRH2	3O6M	H	SISSGGSY	house mouse	IGHV5-9
H2-8-B	CDRH2	4HII	B	VVSSDGRT	human	IGHV3-30
H2-8-B	CDRH2	8FAB	B	VIWYNGSR	human	IGHV3-33
H2-8-B	CDRH2	2VYR	H	GINARGYT	human	IGHV3-23
H2-8-B	CDRH2	3CFJ	B	EINPDSRT	house mouse	IGHV4-1

H2-8-B	CDRH2	4JFX	H	SIVTGGRK	human	IGHV3-66
H2-8-B	CDRH2	1CLY	H	YISQGGDI	house mouse	IGHV5-12
H2-8-B	CDRH2	2AI0	K	AISGNSLY	house mouse	IGHV5S4
H2-8-B	CDRH2	1EJO	H	TISSGGAY	house mouse	IGHV5-6-4
H2-8-B	CDRH2	3ZKS	D	TIGSDGSI	llama	IGHV1S6
H2-8-B	CDRH2	3HB3	C	SINNGGGR	house mouse	IGHV5-12-2
H2-8-B	CDRH2	3EHB	C	SINNGGGR	house mouse	IGHV5-12-2
H2-8-B	CDRH2	3BN9	D	AISGSGGS	human	IGHV3-23
H2-8-B	CDRH2	1HIL	B	TISNGGGY	house mouse	IGHV5-6-1
H2-8-B	CDRH2	3ZLQ	C	TIGSDGSI	llama	IGHV1S6
H2-8-B	CDRH2	2X1Q	A	TISQSGAA	llama	IGHV1S2
H2-8-B	CDRH2	4C57	C	GIWEDSSA	llama	IGHV
H2-8-B	CDRH2	4HH9	B	VISYDGRH	human	IGHV3-30
H2-8-B	CDRH2	4NBZ	B	VISSTGTS	llama	IGHV1S1
H2-8-B	CDRH2	3QXW	E	KISGDGRL	llama	IGHV1S3
H2-8-B	CDRH2	1UZ6	W	EINPDSST	house mouse	IGHV4-1
H2-8-B	CDRH2	3U0W	H	EINPDSST	house mouse	IGHV4-1
H2-8-B	CDRH2	3QXV	B	KISGDGRL	llama	IGHV1S3
H2-8-B	CDRH2	3K74	B	MINPGGII	Arabian camel	IGHV1S23
H2-8-B	CDRH2	3RA7	I	SINIGATY	house mouse	IGHV5-9-3
H2-8-B	CDRH2	2UZI	H	YISRTSKT	human	IGHV3-48
H2-8-B	CDRH2	4JFZ	H	SIATGGHT	human	IGHV3-66
H2-8-B	CDRH2	3U4E	A	FIKYDGSE	human	IGHV3-30
H2-8-B	CDRH2	3V52	H	NINYDGSS	house mouse	IGHV5-16
H2-8-B	CDRH2	3UPC	I	AISGSGGS	human	IGHV3-23
H2-8-B	CDRH2	1T2J	A	SISGSGGN	human	IGHV3-23
H2-8-B	CDRH2	1H0D	B	TISSGGGN	house mouse	IGHV5-9
H2-8-B	CDRH2	4KDT	B	RINSGRDI	Arabian camel	IGHV1S47
H2-8-B	CDRH2	4G5Z	H	IIWYDGDN	human	IGHV3-33
H2-8-B	CDRH2	3O6K	H	SISSGGSY	house mouse	IGHV5-9
H2-8-B	CDRH2	3KYM	L	WIGPSGGI	human	IGHV3-23
H2-8-B	CDRH2	3HI6	X	YIWPSGGN	human	IGHV3-23
H2-8-B	CDRH2	2VYR	L	GINARGYT	human	IGHV3-23
H2-8-B	CDRH2	4GRW	F	CVSSSGQY	llama	IGHV1S1
H2-8-B	CDRH2	3KR3	H	VISSSGGM	human	IGHV3-23
H2-8-B	CDRH2	1UZ6	F	EINPDSST	house mouse	IGHV4-1
H2-8-B	CDRH2	1UZ6	P	EINPDSST	house mouse	IGHV4-1
H2-8-B	CDRH2	2OR9	I	TIGSRGTY	house mouse	IGHV5-6
H2-8-B	CDRH2	4DK3	A	AISRNGAN	llama	IGHV1S1
H2-8-B	CDRH2	3K80	A	AISRSGGL	llama	IGHV1S4
H2-8-B	CDRH2	3UTZ	F	TISSGGSY	house mouse	IGHV5-9
H2-8-B	CDRH2	2X1P	C	SISRSGTL	llama	IGHV
H2-8-B	CDRH2	4OCR	H	FISSDGSN	human	IGHV3-30-3
H2-8-B	CDRH2	4OCS	H	SISFAGTK	human	IGHV3-30
H2-8-B	CDRH2	1OL0	B	AVSGSGGS	human	IGHV3-23
H2-8-B	CDRH2	4KRM	H	GISWRGDS	llama	IGHV1S1
H2-8-B	CDRH2	3ZLQ	D	TIGSDGSI	llama	IGHV1S6
H2-8-B	CDRH2	1FRG	H	TISNGGGY	house mouse	IGHV5-6
H2-8-B	CDRH2	1CLZ	H	YISQGGDI	house mouse	IGHV5-12
H2-8-B	CDRH2	2X89	A	RINSGRDI	Arabian camel	IGHV1S47

H2-8-B	CDRH2	3HI5	H	YIWPSGGN	human	IGHV3-23
H2-8-B	CDRH2	3UPC	D	AISGSGGS	human	IGHV3-23
H2-8-B	CDRH2	3V6F	C	TISSGGNY	house mouse	IGHV5-6-1
H2-8-B	CDRH2	4HIJ	D	VVSSDGRT	human	IGHV3-30
H2-8-B	CDRH2	3QYC	A	AISSSGGS	human	IGHV3-23
H2-8-B	CDRH2	3MUG	B	LISDDGMR	human	IGHV3-30
H2-8-B	CDRH2	4NC1	E	VISSTGTS	llama	IGHV1S1
H2-8-B	CDRH2	3CFK	I	EINPDSRT	house mouse	IGHV4-1
H2-8-B	CDRH2	2X89	B	RINSGRDI	Arabian camel	IGHV1S47
H2-8-B	CDRH2	3UYR	H	NINYDGSS	house mouse	IGHV5-16
H2-8-B	CDRH2	2VYR	E	GINARGYT	human	IGHV3-23
H2-8-B	CDRH2	3QXW	D	KISGDGRL	llama	IGHV1S3
H2-8-B	CDRH2	2X89	C	RINSGRDI	Arabian camel	IGHV1S47
H2-8-B	CDRH2	2HRP	H	YISSGSST	house mouse	IGHV5-17
H2-8-B	CDRH2	3QXV	C	KISGDGRL	llama	IGHV1S3
H2-8-B	CDRH2	4ENE	E	EINPVSST	house mouse	IGHV4-1
H2-8-B	CDRH2	1IKF	H	FISNGGGS	house mouse	IGHV5-12
H2-8-B	CDRH2	3QXT	A	KISGDGRL	Arabian camel	IGHV1S72
H2-8-B	CDRH2	4HDI	H	VITSNGDN	house mouse	IGHV5-6-2
H2-8-B	CDRH2	2NYY	D	TISDGGSY	house mouse	IGHV5-4
H2-8-B	CDRH2	4DKA	A	AISRNGAN	llama	IGHV1S1
H2-8-B	CDRH2	4C83	A	YIGGGSST	house mouse	IGHV5-17
H2-8-B	CDRH2	1F4Y	H	FISSGGGR	house mouse	IGHV5-12-1
H2-8-B	CDRH2	4JFX	B	SIVTGGRK	human	IGHV3-66
H2-8-B	CDRH2	3V6F	H	TISSGGNY	house mouse	IGHV5-6-1
H2-8-B	CDRH2	4KML	B	SITSSGDK	llama	IGHV1S1
H2-8-B	CDRH2	1NL0	H	IISYDGSK	human	IGHV3-30
H2-8-B	CDRH2	2VXS	I	AISGSGGS	human	IGHV3-23
H2-8-B	CDRH2	4HH9	D	VISYDGRH	human	IGHV3-30
H2-8-B	CDRH2	1IGF	J	GISSGGSY	house mouse	IGHV5-9-3
H2-8-B	CDRH2	3KYM	D	WIGPSGGI	human	IGHV3-23
H2-8-B	CDRH2	2ADI	B	TISSGGSY	house mouse	IGHV5-6-1
H2-8-B	CDRH2	3U2S	H	FIKYDGSE	human	IGHV3-30
H2-8-B	CDRH2	3TCL	A	GTNWNGGD	human	IGHV3-20
H2-8-B	CDRH2	4DQO	H	LISDDGMR	human	IGHV3-30
H2-8-B	CDRH2	1OL0	A	AVSGSGGS	human	IGHV3-23
H2-8-B	CDRH2	4KRM	B	GISWRGDS	llama	IGHV1S1
H2-8-B	CDRH2	2XKN	B	TISSGGSY	house mouse	IGHV5S9
H2-8-B	CDRH2	3CFJ	H	EINPDSRT	house mouse	IGHV4-1
H2-8-B	CDRH2	3QXT	B	KISGDGRL	Arabian camel	IGHV1S72
H2-8-B	CDRH2	2VXS	J	AISGSGGS	human	IGHV3-23
H2-8-B	CDRH2	3SO3	C	AISGSGGS	human	IGHV3-23
H2-8-B	CDRH2	3KYM	B	WIGPSGGI	human	IGHV3-23
H2-8-B	CDRH2	1Y18	F	DINPDSRT	house mouse	IGHV4-1
H2-8-B	CDRH2	3QXW	C	KISGDGRL	llama	IGHV1S3
H2-8-B	CDRH2	3QXU	C	KISGDGRL	llama	IGHV1S5
H2-8-B	CDRH2	3QXU	A	KISGDGRL	llama	IGHV1S5
H2-8-B	CDRH2	3EYQ	D	VISYDGSN	human	IGHV3-30
H2-8-B	CDRH2	4NZU	H	FISYNGSS	human	IGHV3-30
H2-8-B	CDRH2	3TCL	H	GTNWNGGD	human	IGHV3-20

H2-8-B	CDRH2	1DEE	D	LISYDESN	human	IGHV3-30
H2-8-B	CDRH2	4BFB	E	TIGSDGSI	llama	IGHV1S6
H2-8-B	CDRH2	3EYF	B	VISSDGTD	human	IGHV3-30
H2-8-B	CDRH2	3V4U	H	NINYDGSS	house mouse	IGHV5-16
H2-8-B	CDRH2	2OTW	B	FISNGGGS	house mouse	IGHV5-12
H2-8-B	CDRH2	2OTU	D	FISNGGGS	house mouse	IGHV5-12
H2-8-B	CDRH2	3LRS	C	LISDDGMR	human	IGHV3-30
H2-8-B	CDRH2	4N9O	B	SITSSGDK	llama	IGHV1S1
H2-8-B	CDRH2	4HPY	H	GISWNSNI	human	IGHV3-9
H2-8-B	CDRH2	3ZHK	A	AISGSGGS	human	IGHV3-23
H2-8-B	CDRH2	1MF2	H	YISSGSST	house mouse	IGHV5-17
H2-8-B	CDRH2	2XKN	D	TISSGGSY	house mouse	IGHV5S9
H2-8-B	CDRH2	3INU	H	SISSSSSY	human	IGHV3-21
H2-8-B	CDRH2	1W72	H	GISWNSGS	human	IGHV3-9
H2-8-B	CDRH2	3HR5	J	FISDLAYT	house mouse	IGHV3-48
H2-8-B	CDRH2	3CFJ	F	EINPDSRT	house mouse	IGHV4-1
H2-8-B	CDRH2	3ZKQ	D	TIGSDGSI	llama	IGHV1S6
H2-8-B	CDRH2	2VYR	G	GINARGYT	human	IGHV3-23
H2-8-B	CDRH2	2GSG	B	FISNGGGS	house mouse	IGHV5-12
H2-8-B	CDRH2	1MQK	H	SINNGGGR	house mouse	IGHV5-12-2
H2-8-B	CDRH2	4DKA	B	AISRNGAN	llama	IGHV1S1
H2-8-B	CDRH2	3MUG	H	LISDDGMR	human	IGHV3-30
H2-8-B	CDRH2	4FHB	D	VISWSGSN	llama	IGHV
H2-8-B	CDRH2	2OTU	F	FISNGGGS	house mouse	IGHV5-12
H2-8-B	CDRH2	1IGF	H	GISSGGSY	house mouse	IGHV5-9-3
H2-8-B	CDRH2	2VH5	H	YISRTSKT	human	IGHV3-48
H2-8-B	CDRH2	3UPC	G	AISGSGGS	human	IGHV3-23
H2-8-B	CDRH2	1HEZ	B	LISYDESN	human	IGHV3-30
H2-8-B	CDRH2	3CFK	H	EINPDSRT	house mouse	IGHV4-1
H2-8-B	CDRH2	2OTU	H	FISNGGGS	house mouse	IGHV5-12
H2-8-B	CDRH2	1DEE	F	LISYDESN	human	IGHV3-30
H2-8-B	CDRH2	3CFK	N	EINPDSRT	house mouse	IGHV4-1
H2-8-B	CDRH2	4AEI	I	SISDGGSF	house mouse	IGHV5-4
H2-8-B	CDRH2	4NCC	2	QINPHSST	house mouse	IGHV4-1
H2-8-B	CDRH2	3HR5	B	FISDLAYT	house mouse	IGHV3-48
H2-8-B	CDRH2	3CFJ	D	EINPDSRT	house mouse	IGHV4-1
H2-8-B	CDRH2	3ZHD	B	LISGSGGS	human	IGHV3-23
H2-8-B	CDRH2	4AEI	J	SISDGGSF	house mouse	IGHV5-4
H2-8-B	CDRH2	2ADG	B	TISSGGSY	house mouse	IGHV5-6-1
H2-8-B	CDRH2	1Y18	D	DINPDSRT	house mouse	IGHV4-1
H2-8-B	CDRH2	4KRM	J	GISWRGDS	llama	IGHV1S1
H2-8-B	CDRH2	4BEL	D	TIGSDGSI	llama	IGHV1S6
H2-8-B	CDRH2	1Y18	H	DINPDSRT	house mouse	IGHV4-1
H2-8-B	CDRH2	3UPC	C	AISGSGGS	human	IGHV3-23
H2-8-B	CDRH2	3QXU	B	KISGDGRL	llama	IGHV1S5
H2-8-B	CDRH2	1IGC	H	YISSGSST	house mouse	IGHV5-17
H2-8-B	CDRH2	3QOS	H	AISGSGGS	human	IGHV3-23
H2-8-B	CDRH2	2QSC	H	AISSDGET	human	IGHV3-64
H2-8-B	CDRH2	4OD1	H	AISYDGTD	human	IGHV3-30
H2-8-B	CDRH2	4EJ1	C	AITWGGST	llama	IGHV1S3

H2-8-B	CDRH2	2OTU	B	FISNGGGS	house mouse	IGHV5-12
H2-8-B	CDRH2	3H42	H	SISSSSSY	human	IGHV3-21
H2-8-B	CDRH2	3G6A	H	GIAYDSSN	human	IGHV3-30
H2-8-B	CDRH2	2GSG	D	FISNGGGS	house mouse	IGHV5-12
H2-8-B	CDRH2	1Y0L	H	EINPDSRT	house mouse	IGHV4-1
H2-8-B	CDRH2	3QXU	D	KISGDGRL	llama	IGHV1S5
H2-8-B	CDRH2	3GHE	H	NIKQDGND	human	IGHV3-7
H2-8-B	CDRH2	2A77	H	AISGNSLY	house mouse	IGHV5S4
H2-8-B	CDRH2	3TNN	H	SINNSGRN	human	IGHV3-23
H2-8-B	CDRH2	3EYO	B	VISSDGTD	human	IGHV3-30
H2-8-B	CDRH2	3QXV	D	KISGDGRL	llama	IGHV1S3
H2-8-B	CDRH2	4KVC	H	YISSGSST	house mouse	IGHV5-17
H2-8-B	CDRH2	1OTS	E	EINPVSST	house mouse	IGHV4-1
H2-8-B	CDRH2	3CFK	F	EINPDSRT	house mouse	IGHV4-1
H2-8-B	CDRH2	1Y0L	B	EINPDSRT	house mouse	IGHV4-1
H2-8-B	CDRH2	3TNN	E	SINNSGRN	human	IGHV3-23
H2-8-B	CDRH2	3MUG	L	LISDDGMR	human	IGHV3-30
H2-8-B	CDRH2	2ORB	H	TIGSRGTY	house mouse	IGHV5-6
H2-8-B	CDRH2	4DK6	B	AISRNGAN	llama	IGHV1S1
H2-8-B	CDRH2	3BN9	F	AISGSGGS	human	IGHV3-23
H2-8-B	CDRH2	3UPC	B	AISGSGGS	human	IGHV3-23
H2-8-B	CDRH2	3INU	M	SISSSSSY	human	IGHV3-21
H2-8-B	CDRH2	3UTZ	B	TISSGGSY	house mouse	IGHV5-9
H2-8-B	CDRH2	3NH7	J	YTSSSGSL	human	IGHV3-48
H2-8-B	CDRH2	3QXV	A	KISGDGRL	llama	IGHV1S3
H2-8-B	CDRH2	2VYR	J	GINARGYT	human	IGHV3-23
H2-8-B	CDRH2	4AEI	H	SISDGGSF	house mouse	IGHV5-4
H2-8-B	CDRH2	3HR5	H	FISDLAYT	house mouse	IGHV3-48
H2-8-B	CDRH2	3UO1	H	NINYDGSS	house mouse	IGHV5-16
H2-8-B	CDRH2	3ZKX	B	TIGSDGSI	llama	IGHV1S6
H2-8-B	CDRH2	4EIZ	D	AITWGGST	llama	IGHV1S3
H2-8-B	CDRH2	1DFB	H	GISWDSSS	human	IGHV3-9
H2-8-B	CDRH2	4HIJ	B	VVSSDGRT	human	IGHV3-30
H2-8-B	CDRH2	2FB4	H	IIWDDGSD	human	IGHV3-30
H2-8-B	CDRH2	4ENE	C	EINPVSST	house mouse	IGHV4-1
H2-8-B	CDRH2	3LRS	A	LISDDGMR	human	IGHV3-30
H2-8-B	CDRH2	2FBJ	H	EIHPDSGT	house mouse	IGHV4-1
H2-8-B	CDRH2	3LIZ	H	TINSNGAS	house mouse	IGHV5-6-3
H2-8-B	CDRH2	2VYR	K	GINARGYT	human	IGHV3-23
H2-8-B	CDRH2	1QD0	A	AIRWSGKE	llama	IGHV1S3
H2-8-B	CDRH2	3NH7	H	YTSSSGSL	human	IGHV3-48
H2-8-B	CDRH2	4FQL	H	GINWKGNF	human	IGHV3-9
H2-8-B	CDRH2	3U4E	H	FIKYDGSE	human	IGHV3-30
H2-8-B	CDRH2	1UZ8	H	EINPDSST	house mouse	IGHV4-1
H2-8-B	CDRH2	1IGT	D	YISNGGGS	house mouse	IGHV5-12
H2-8-B	CDRH2	2HRP	N	YISSGSST	house mouse	IGHV5-17
H2-8-B	CDRH2	2X1Q	B	TISQSGAA	llama	IGHV1S2
H2-8-B	CDRH2	2VXS	H	AISGSGGS	human	IGHV3-23
H2-8-B	CDRH2	2AI0	H	AISGNSLY	house mouse	IGHV5S4
H2-8-B	CDRH2	3CFK	B	EINPDSRT	house mouse	IGHV4-1

H2-8-B	CDRH2	4G6J	H	IIWYDGDN	human	IGHV3-33
H2-8-B	CDRH2	3KYM	F	WIGPSGGI	human	IGHV3-23
H2-8-B	CDRH2	2A1W	H	AISGNSLY	house mouse	IGHV5S4
H2-8-B	CDRH2	1H3P	H	EVSSDGSY	house mouse	IGHV5-9-4
H2-8-B	CDRH2	3UPC	F	AISGSGGS	human	IGHV3-23
H2-8-B	CDRH2	4BFB	D	TIGSDGSI	llama	IGHV1S6
H2-8-B	CDRH2	4NBZ	D	VISSTGTS	llama	IGHV1S1
H2-8-B	CDRH2	3UPC	H	AISGSGGS	human	IGHV3-23
H2-8-B	CDRH2	1UZ8	B	EINPDSST	house mouse	IGHV4-1
H2-8-B	CDRH2	4KFZ	C	YISYNSSS	human	IGHV3-23
H2-8-B	CDRH2	3NH7	I	YTSSSGSL	human	IGHV3-48
H2-8-B	CDRH2	3GIZ	H	TISWNSGS	human	IGHV3-9
H2-8-B	CDRH2	2VYR	I	GINARGYT	human	IGHV3-23
H2-8-B	CDRH2	2R56	I	SLSGSGTK	human	IGHV3-23
H2-8-B	CDRH2	1IFH	H	TISNGGGY	house mouse	IGHV5-6-1
H2-8-B	CDRH2	3UPC	J	AISGSGGS	human	IGHV3-23
H2-8-B	CDRH2	4AQ1	D	GISSKGGS	llama	IGHV1S1
H2-8-B	CDRH2	3V6F	A	TISSGGNY	house mouse	IGHV5-6-1
H2-8-B	CDRH2	4KDT	A	RINSGRDI	Arabian camel	IGHV1S47
H2-8-B	CDRH2	4HHA	B	VISYDGRH	human	IGHV3-30
H2-8-B	CDRH2	3EYF	D	VISSDGTD	human	IGHV3-30
H2-8-B	CDRH2	2OR9	H	TIGSRGTY	house mouse	IGHV5-6
H2-8-B	CDRH2	3TNN	A	SINNSGRN	human	IGHV3-23
H2-8-B	CDRH2	2QHR	H	YISRGGGY	house mouse	IGHV5-12-1
H2-8-B	CDRH2	1DQL	H	VISSDGGN	human	IGHV3-30
H2-8-B	CDRH2	3CFK	D	EINPDSRT	house mouse	IGHV4-1
H2-8-B	CDRH2	3QXW	B	KISGDGRL	llama	IGHV1S3
H2-8-B	CDRH2	2A1W	I	AISGNSLY	house mouse	IGHV5S4
H2-8-B	CDRH2	3UPC	A	AISGSGGS	human	IGHV3-23
H2-8-B	CDRH2	4NC0	B	VISSTGTS	llama	IGHV1S1
H2-8-B	CDRH2	3NH7	K	YTSSSGSL	human	IGHV3-48
H2-8-B	CDRH2	1I8K	B	SISTGGYN	house mouse	IGHV5-6
H2-8-B	CDRH2	2X1P	A	SISRSGTL	llama	IGHV
H2-8-B	CDRH2	4EIZ	C	AITWGGST	llama	IGHV1S3
H2-8-B	CDRH2	3TNN	C	SINNSGRN	human	IGHV3-23
H2-8-B	CDRH2	2VXS	K	AISGSGGS	human	IGHV3-23
H2-8-B	CDRH2	4HIE	B	VVSSDGRT	human	IGHV3-30
H2-8-B	CDRH2	3S34	H	SISSSSSY	human	IGHV3-21
H2-8-B	CDRH2	3U2S	A	FIKYDGSE	human	IGHV3-30
H2-8-B	CDRH2	1I8I	B	SISTGGYN	house mouse	IGHV5-6
H2-8-B	CDRH2	3LN9	A	VISQSGMR	Arabian camel	IGHV1S42
H2-8-B	CDRH2	1DEE	B	LISYDESN	human	IGHV3-30
H2-8-B	CDRH2	3V6F	E	TISSGGNY	house mouse	IGHV5-6-1
H2-8-B	CDRH2	4KRM	D	GISWRGDS	llama	IGHV1S1
H2-8-B	CDRH2	1HEZ	D	LISYDESN	human	IGHV3-30
H2-8-B	CDRH2	1HIL	D	TISNGGGY	house mouse	IGHV5-6-1
H2-8-B	CDRH2	4GFT	B	GITGSGRS	llama	IGHV
H2-8-B	CDRH2	3QXW	A	KISGDGRL	llama	IGHV1S3
H2-8-B	CDRH2	1W72	I	GISWNSGS	human	IGHV3-9
H2-8-B	CDRH2	2AI0	J	AISGNSLY	house mouse	IGHV5S4

H2-8-B	CDRH2	3CFK	K	EINPDSRT	house mouse	IGHV4-1
H2-8-B	CDRH2	4DTG	H	TISRSGSY	house mouse	IGHV3-21
H2-8-B	CDRH2	3EYO	D	VISSDGTD	human	IGHV3-30
H2-8-B	CDRH2	1UZ6	H	EINPDSST	house mouse	IGHV4-1
H2-8-B	CDRH2	3QYC	B	AISSSGGS	human	IGHV3-23
H2-8-B	CDRH2	4DK3	B	AISRNGAN	llama	IGHV1S1
H2-8-B	CDRH2	4GXV	H	VISYDGRN	human	IGHV3-30
H2-8-B	CDRH2	1UCB	H	YISQGGDI	house mouse	IGHV5-12
H2-8-B	CDRH2	3ZHD	A	LISGSGGS	human	IGHV3-23
H2-8-B	CDRH2	2R56	H	SLSGSGTK	human	IGHV3-23
H2-8-B	CDRH2	2X1P	D	SISRSGTL	llama	IGHV
H2-8-B	CDRH2	3G6A	B	GIAYDSSN	human	IGHV3-30
H2-8-B	CDRH2	4HII	D	VVSSDGRT	human	IGHV3-30
H2-8-B	CDRH2	2ORB	I	TIGSRGTY	house mouse	IGHV5-6
H2-8-B	CDRH2	1SEQ	H	YISKGGGS	house mouse	IGHV5-12-2
H2-8-B	CDRH2	3K7U	A	AISWTGGL	llama	IGHV1S3
H2-8-B	CDRH2	4EJ1	D	AITWGGST	llama	IGHV1S3
H2-8-B	CDRH2	4BEL	E	TIGSDGSI	llama	IGHV1S6
H2-8-B	CDRH2	4KRM	L	GISWRGDS	llama	IGHV1S1
H2-8-B	CDRH2	3FFD	A	TISSGGSY	house mouse	IGHV5-6-1
H2-8-B	CDRH2	1Y0L	F	EINPDSRT	house mouse	IGHV4-1
H2-8-B	CDRH2	3HR5	I	FISDLAYT	house mouse	IGHV3-48
H2-8-B	CDRH2	4AQ1	B	GISSKGGS	llama	IGHV1S1
H2-8-B	CDRH2	4GXV	I	VISYDGRN	human	IGHV3-30
H2-8-B	CDRH2	2AI0	I	AISGNSLY	house mouse	IGHV5S4
H2-8-B	CDRH2	1QKZ	H	TISDGGAY	house mouse	IGHV5-4
H2-8-B	CDRH2	4NBX	B	AGSSTGRT	llama	IGHV1S3
H2-8-B	CDRH2	4KRM	F	GISWRGDS	llama	IGHV1S1
H2-8-B	CDRH2	2GCY	B	TISDGGSY	house mouse	IGHV5-4
H2-8-B	CDRH2	1AQK	H	FISYDGSK	human	IGHV3-30
H2-8-B	CDRH2	3LRS	H	LISDDGMR	human	IGHV3-30
H2-8-B	CDRH2	2IGF	H	GISSGGSY	house mouse	IGHV5-9-3
H2-8-B	CDRH2	3CFK	P	EINPDSRT	house mouse	IGHV4-1
H2-8-B	CDRH2	2OTW	D	FISNGGGS	house mouse	IGHV5-12
H2-8-B	CDRH2	4HIH	B	VVSSDGRT	human	IGHV3-30
H2-8-B	CDRH2	1Y18	B	DINPDSRT	house mouse	IGHV4-1
H2-8-B	CDRH2	2AAB	H	YISSDSSN	house mouse	IGHV5-17
H2-8-B	CDRH2	1MF2	N	YISSGSST	house mouse	IGHV5-17
H2-8-B	CDRH2	8FAB	D	VIWYNGSR	human	IGHV3-33
H2-8-B	CDRH2	4HIH	D	VVSSDGRT	human	IGHV3-30
H2-8-B	CDRH2	2VYR	F	GINARGYT	human	IGHV3-23
H2-8-B	CDRH2	3K80	B	AISRSGGL	llama	IGHV1S4
H2-8-B	CDRH2	3O6L	H	SISSGGSY	house mouse	IGHV5-9
H2-8-B	CDRH2	4DK6	A	AISRNGAN	llama	IGHV1S1
H2-8-B	CDRH2	1Y0L	D	EINPDSRT	house mouse	IGHV4-1
H2-8-B	CDRH2	4C83	C	YIGGGSST	house mouse	IGHV5-17
H2-8-C	CDRH2	2YBR	A	GISRSSGD	human	IGHV3-9
H2-8-C	CDRH2	3OAY	H	SISTSSTY	human	IGHV3-21
H2-8-C	CDRH2	2YBR	D	GISRSSGD	human	IGHV3-9
H2-8-C	CDRH2	3OAU	H	SISTSSTY	human	IGHV3-21

H2-8-C	CDRH2	3OAZ	H	SISTSSTY	human	IGHV3-21
H2-8-C	CDRH2	2OQJ	B	SISTSSTY	human	IGHV3-21
H2-8-C	CDRH2	3OAZ	M	SISTSSTY	human	IGHV3-21
H2-8-C	CDRH2	1ZLV	M	SISTSSTY	human	IGHV3-21
H2-8-C	CDRH2	2YC1	A	GISRSSGD	human	IGHV3-9
H2-8-C	CDRH2	2OQJ	K	SISTSSTY	human	IGHV3-21
H2-8-C	CDRH2	3OAY	M	SISTSSTY	human	IGHV3-21
H2-8-C	CDRH2	2OQJ	H	SISTSSTY	human	IGHV3-21
H2-8-C	CDRH2	1OP3	H	SISTSSTY	human	IGHV3-21
H2-8-C	CDRH2	2YBR	G	GISRSSGD	human	IGHV3-9
H2-8-C	CDRH2	1OM3	H	SISTSSTY	human	IGHV3-21
H2-8-C	CDRH2	2YC1	D	GISRSSGD	human	IGHV3-9
H2-8-C	CDRH2	1ZLU	H	SISTSSTY	human	IGHV3-21
H2-8-C	CDRH2	1ZLU	M	SISTSSTY	human	IGHV3-21
H2-8-C	CDRH2	1ZLV	H	SISTSSTY	human	IGHV3-21
H2-8-C	CDRH2	1ZLS	H	SISTSSTY	human	IGHV3-21
H2-8-C	CDRH2	1OM3	K	SISTSSTY	human	IGHV3-21
H2-8-C	CDRH2	2OQJ	E	SISTSSTY	human	IGHV3-21
H2-8-C	CDRH2	1OP3	M	SISTSSTY	human	IGHV3-21
H2-8-D	CDRH2	1MLC	B	EILPGSGS	house mouse	IGHV1-9
H2-8-D	CDRH2	1YNL	H	EILPGSGR	house mouse	IGHV1-9
H2-8-D	CDRH2	1YNK	H	EILPGSGR	house mouse	IGHV1-9
H2-8-D	CDRH2	1CGS	H	EILPGSGR	house mouse	IGHV1-9
H2-8-D	CDRH2	2CGR	H	EILPGSGR	house mouse	IGHV1-9
H2-8-D	CDRH2	3DIF	D	WVFPGSGN	house mouse	IGHV1-66
H2-8-D	CDRH2	1P2C	B	EILPGSDS	house mouse	IGHV1-9
H2-8-D	CDRH2	1PSK	H	DINPNNGG	house mouse	IGHV1-26
H2-8-D	CDRH2	3OZ9	H	KILPGIGS	house mouse	IGHV1-9
H2-8-D	CDRH2	1P2C	E	EILPGSDS	house mouse	IGHV1-9
H2-8-D	CDRH2	1D5I	H	EILPGSGS	house mouse	IGHV1-9
H2-8-D	CDRH2	1RZG	C	GIIPIFNI	human	IGHV1-69
H2-8-D	CDRH2	1RZG	A	GIIPIFNI	human	IGHV1-69
H2-8-D	CDRH2	1MLC	D	EILPGSGS	house mouse	IGHV1-9
H2-8-D	CDRH2	4AEH	H	NISPGGSN	house mouse	IGHV1S61
H2-8-D	CDRH2	1EHL	H	TIYPGNSD	house mouse	IGHV1-5
H2-8-D	CDRH2	4I2X	B	WVFPGSGN	human	IGHV1-66
H2-8-D	CDRH2	3DIF	B	WVFPGSGN	house mouse	IGHV1-66
H2-8-D	CDRH2	1YQV	H	EILPGSGS	house mouse	IGHV1-9
H2-8-E	CDRH2	3U1S	H	WMSHEGDK	human	IGHV1-8
H2-8-E	CDRH2	3K1K	C	GMSSAGDR	llama	IGHV1S1
H2-8-E	CDRH2	3OGO	F	GMSSAGDR	Arabian camel	IGHV1S26
H2-8-E	CDRH2	3K1K	D	GMSSAGDR	llama	IGHV1S1
H2-8-E	CDRH2	3OGO	E	GMSSAGDR	Arabian camel	IGHV1S26
H2-8-E	CDRH2	3OGO	H	GMSSAGDR	Arabian camel	IGHV1S26
H2-8-E	CDRH2	3QPX	H	SISPDGGS	Norway rat	IGHV5S47
H2-8-E	CDRH2	3OGO	G	GMSSAGDR	Arabian camel	IGHV1S26
H2-8-F	CDRH2	2XXM	B	AIRGGDMS	llama	IGHV1S4
H2-8-F	CDRH2	3GBM	H	GIIPIFGT	human	IGHV1-69
H2-8-F	CDRH2	3GBM	I	GIIPIFGT	human	IGHV1-69
H2-8-F	CDRH2	2XV6	B	AIRGGDMS	alpaca	IGHV

H2-8-F	CDRH2	2XT1	B	AIRGGDMS	llama	IGHV1S4
H2-8-F	CDRH2	3GBN	H	GIIPIFGT	human	IGHV1-69
H2-8-F	CDRH2	2XV6	D	AIRGGDMS	alpaca	IGHV
H2-8-G	CDRH2	1YJD	H	CIYPGNVN	house mouse	IGHV1S12
H2-8-G	CDRH2	1A0Q	H	YISPGNGD	house mouse	IGHV1S53
H2-8-G	CDRH2	1ZA6	F	YFSPGNDD	house mouse	IGHV1
H2-8-G	CDRH2	1ZA6	B	YFSPGNDD	house mouse	IGHV1
H2-8-G	CDRH2	1ZA6	D	YFSPGNDD	house mouse	IGHV1
H2-8-G	CDRH2	1ZA6	H	YFSPGNDD	house mouse	IGHV1
H2-8-H	CDRH2	3EYV	B	YMSNVGAI	house mouse	IGHV5-12
H2-8-H	CDRH2	1S3K	H	YMSNVGAI	house mouse	IGHV3-30
H2-8-H	CDRH2	3HI6	H	YIWPSGGN	human	IGHV3-23
H2-8-H	CDRH2	3EYV	H	YMSNVGAI	house mouse	IGHV5-12
H2-8-I	CDRH2	1BLN	D	TISSGGGN	house mouse	IGHV5-9
H2-8-I	CDRH2	3QOS	B	AISGSGGS	human	IGHV3-23
H2-8-I	CDRH2	3KYM	J	WIGPSGGI	human	IGHV3-23
H2-8-J	CDRH2	1DSF	H	TISDGGTY	house mouse	IGHV5-4
H2-8-J	CDRH2	1F4X	H	FISSGGGR	house mouse	IGHV5-12-1
H2-8-J	CDRH2	1F4W	H	FISSGGGR	house mouse	IGHV5-12-1
H2-10-A	CDRH2	3QNZ	B	RIKRNAESDA	human	IGHV3-73
H2-10-A	CDRH2	1SM3	H	EIRLKSNNYA	house mouse	IGHV6-6
H2-10-A	CDRH2	3DUS	D	FIRNKAKGYT	house mouse	IGHV7-3
H2-10-A	CDRH2	3C2A	I	RIKSRTDGGT	human	IGHV3-15
H2-10-A	CDRH2	3HZY	B	FIRNKAKGYT	house mouse	IGHV7-3
H2-10-A	CDRH2	4M1D	H	RIKSRTDGGT	human	IGHV3-15
H2-10-A	CDRH2	3OKD	B	FIRNKAKGYT	house mouse	IGHV7-3
H2-10-A	CDRH2	2R2E	B	FIRNKANGYT	house mouse	IGHV7-3
H2-10-A	CDRH2	3BZ4	H	EIRLKSDNYA	house mouse	IGHV6-3
H2-10-A	CDRH2	1WC7	H	LIRNKANGYT	house mouse	IGHV7-3
H2-10-A	CDRH2	2R1Y	B	FIRNKANGYT	house mouse	IGHV7-3
H2-10-A	CDRH2	3SGE	J	RIRSKINNYS	house mouse	IGHV10S3
H2-10-A	CDRH2	3PHQ	B	FIRNKGNGYT	house mouse	IGHV7-3
H2-10-A	CDRH2	3FO9	B	EIRLKSDNYA	house mouse	IGHV6-3
H2-10-A	CDRH2	3IJH	D	FIRNKAKGYT	house mouse	IGHV7-3
H2-10-A	CDRH2	3M8O	H	RIKRNAESDA	human	IGHV3-73
H2-10-A	CDRH2	2G5B	D	FIRNKVNGYT	house mouse	IGHV7-3
H2-10-A	CDRH2	1CE1	H	FIRDKAKGYT	human	IGHV4-59
H2-10-A	CDRH2	3DSF	H	RIRSQSNNYT	house mouse	IGHV10-1
H2-10-A	CDRH2	1Q9O	D	FIRNKPKGYT	house mouse	IGHV7-3
H2-10-A	CDRH2	3OKE	B	FIRNKAKGYT	house mouse	IGHV7-3
H2-10-A	CDRH2	1T66	H	QIRNKPYNYE	house mouse	IGHV6-7
H2-10-A	CDRH2	3OKN	B	FIRNKAKGYT	house mouse	IGHV7-3
H2-10-A	CDRH2	1FL5	B	FIRNKANGYT	house mouse	IGHV3-72
H2-10-A	CDRH2	3HZK	B	FIRNKAKGYT	house mouse	IGHV7-3
H2-10-A	CDRH2	3C2A	H	RIKSRTDGGT	human	IGHV3-15
H2-10-A	CDRH2	3QO0	B	RIKRNAESDA	human	IGHV3-73
H2-10-A	CDRH2	2V17	H	LIRNKAKGYT	house mouse	IGHV7-3
H2-10-A	CDRH2	1WC7	B	LIRNKANGYT	house mouse	IGHV7-3
H2-10-A	CDRH2	1T66	D	QIRNKPYNYE	house mouse	IGHV6-7
H2-10-A	CDRH2	1V7M	I	EIRSKVNNHA	house mouse	IGHV6-6

H2-10-A	CDRH2	1WCB	B	LIRNKANGYT	house mouse	IGHV7-3
H2-10-A	CDRH2	1KEL	H	FIRNKAKGYT	house mouse	IGHV7-3
H2-10-A	CDRH2	1BFO	F	FIRDKAKGYT	Norway rat	IGHV7S6
H2-10-A	CDRH2	3DV6	B	FIRNKAKGYT	house mouse	IGHV7-3
H2-10-A	CDRH2	3IET	B	EIRNKANNHE	house mouse	IGHV6-6
H2-10-A	CDRH2	3CXD	H	RIRSQSNNYT	house mouse	IGHV10-1
H2-10-A	CDRH2	1NFD	H	RIKNIPNNYA	hamster	IGHV6-6
H2-10-A	CDRH2	3T65	B	FIRNKANGYT	house mouse	IGHV7-3
H2-10-A	CDRH2	2CJU	H	SIRNKANGYT	house mouse	IGHV7-3
H2-10-A	CDRH2	1Q9O	B	FIRNKPKGYT	house mouse	IGHV7-3
H2-10-A	CDRH2	3T4Y	B	FIRNKANGYT	house mouse	IGHV7-3
H2-10-A	CDRH2	3IJH	B	FIRNKAKGYT	house mouse	IGHV7-3
H2-10-A	CDRH2	3OKL	B	FIRNKAKGYT	house mouse	IGHV7-3
H2-10-A	CDRH2	2G5B	F	FIRNKVNGYT	house mouse	IGHV7-3
H2-10-A	CDRH2	2DLF	H	EIRNKANNHA	house mouse	IGHV6-6
H2-10-A	CDRH2	4HGW	B	FIRNKANGYT	house mouse	IGHV7-3
H2-10-A	CDRH2	3IF1	D	EIRNKANNHE	house mouse	IGHV6-6
H2-10-A	CDRH2	1Q9L	D	FIRNKANGYT	house mouse	IGHV7-3
H2-10-A	CDRH2	3BZ4	D	EIRLKSDNYA	house mouse	IGHV6-3
H2-10-A	CDRH2	3DUU	B	FIRNKAKGYT	house mouse	IGHV7-3
H2-10-A	CDRH2	3BZ4	F	EIRLKSDNYA	house mouse	IGHV6-3
H2-10-A	CDRH2	1Q9W	D	FIRNKPKGYT	house mouse	IGHV7-3
H2-10-A	CDRH2	4G6F	H	RITGPGEGWS	human	IGHV3-15
H2-10-A	CDRH2	3SGD	J	RIRSKINNYS	house mouse	IGHV10S3
H2-10-A	CDRH2	1CK0	H	RIRSKGFNFA	house mouse	IGHV10S3
H2-10-A	CDRH2	1RIV	H	EIRNKANNHA	house mouse	IGHV6-6
H2-10-A	CDRH2	3BPC	B	FIRNKANGYT	house mouse	IGHV7-3
H2-10-A	CDRH2	3I02	D	FIRNKVKGYT	house mouse	IGHV7-3
H2-10-A	CDRH2	2G5B	H	FIRNKVNGYT	house mouse	IGHV7-3
H2-10-A	CDRH2	3IF1	B	EIRNKANNHE	house mouse	IGHV6-6
H2-10-A	CDRH2	3V0V	H	LIRNKRNGDT	house mouse	IGHV7-3
H2-10-A	CDRH2	1RIU	H	EIRNKANNHA	house mouse	IGHV6-6
H2-10-A	CDRH2	3FO9	H	EIRLKSDNYA	house mouse	IGHV6-3
H2-10-A	CDRH2	3QO1	B	RIKRNAESDA	human	IGHV3-73
H2-10-A	CDRH2	3BZ4	B	EIRLKSDNYA	house mouse	IGHV6-3
H2-10-A	CDRH2	4FAB	H	QIRNKPYNYE	house mouse	IGHV6-7
H2-10-A	CDRH2	1PZ5	B	EIRLKSNNYA	house mouse	IGHV6-6
H2-10-A	CDRH2	3DUU	D	FIRNKAKGYT	house mouse	IGHV7-3
H2-10-A	CDRH2	1FL5	H	FIRNKANGYT	house mouse	IGHV3-72
H2-10-A	CDRH2	2BMK	B	LIRNKANGYT	house mouse	IGHV7-3
H2-10-A	CDRH2	1Q1J	I	RIKSRTDGGT	human	IGHV3-15
H2-10-A	CDRH2	1NBV	H	RIRSKSNNYA	house mouse	IGHV10S3
H2-10-A	CDRH2	1WCB	H	LIRNKANGYT	house mouse	IGHV7-3
H2-10-A	CDRH2	3I02	B	FIRNKVKGYT	house mouse	IGHV7-3
H2-10-A	CDRH2	3GHB	H	RIKSRTDGGT	human	IGHV3-15
H2-10-A	CDRH2	2AEP	H	LIRNKGNGYT	house mouse	IGHV7-3
H2-10-A	CDRH2	4G6F	B	RITGPGEGWS	human	IGHV3-15
H2-10-A	CDRH2	3C6S	D	EIRLKSDNYA	house mouse	IGHV6-3
H2-10-A	CDRH2	3C5S	D	EIRLKSDNYA	house mouse	IGHV6-3
H2-10-A	CDRH2	2G5B	B	FIRNKVNGYT	house mouse	IGHV7-3

H2-10-A	CDRH2	3C6S	F	EIRLKSDNYA	house mouse	IGHV6-3
H2-10-A	CDRH2	2HKF	H	RIRSKSNNYA	house mouse	IGHV10-1
H2-10-A	CDRH2	1RFD	H	EIRNKANNHA	house mouse	IGHV6-6
H2-10-A	CDRH2	3T77	B	FIRNKANGYT	house mouse	IGHV7-3
H2-10-A	CDRH2	1WZ1	H	EIRNKANNHA	house mouse	IGHV6-6
H2-10-A	CDRH2	3SGE	H	RIRSKINNYS	house mouse	IGHV10S3
H2-10-A	CDRH2	2CK0	H	RIRSKGFNFA	house mouse	IGHV10-1
H2-10-A	CDRH2	3DV4	B	FIRNKAKGYT	house mouse	IGHV7-3
H2-10-A	CDRH2	2R2H	B	FIRNKANGYT	house mouse	IGHV7-3
H2-10-A	CDRH2	1QYG	H	EIRNKANNHA	house mouse	IGHV6-6
H2-10-A	CDRH2	1FL6	B	FIRNKANGYT	house mouse	IGHV3-72
H2-10-A	CDRH2	1M7I	B	EIRLKSNNYA	house mouse	IGHV6-6
H2-10-A	CDRH2	1BFO	D	FIRDKAKGYT	Norway rat	IGHV7S6
H2-10-A	CDRH2	3SGD	H	RIRSKINNYS	house mouse	IGHV10S3
H2-10-A	CDRH2	2R1W	B	FIRNKANGYT	house mouse	IGHV7-3
H2-10-A	CDRH2	1NFD	F	RIKNIPNNYA	hamster	IGHV6-6
H2-10-A	CDRH2	3OKO	B	FIRNKAKGYT	house mouse	IGHV7-3
H2-10-A	CDRH2	1CBV	H	RIRSKSNNYA	house mouse	IGHV10S3
H2-10-A	CDRH2	3IET	D	EIRNKANNHE	house mouse	IGHV6-6
H2-10-A	CDRH2	3C6S	H	EIRLKSDNYA	house mouse	IGHV6-3
H2-10-A	CDRH2	3DUS	B	FIRNKAKGYT	house mouse	IGHV7-3
H2-10-A	CDRH2	1AD0	D	FIGNKANGYT	human	IGHV3-72
H2-10-A	CDRH2	3DUR	D	FIRNKAKGYT	house mouse	IGHV7-3
H2-10-A	CDRH2	1DLF	H	EIRNKANNHA	house mouse	IGHV6-6
H2-10-A	CDRH2	1V7M	H	EIRSKVNNHA	house mouse	IGHV6-6
H2-10-A	CDRH2	1M7D	B	EIRLKSNNYA	house mouse	IGHV6-6
H2-10-A	CDRH2	2HKH	H	RIRSKSNNYA	house mouse	IGHV10-1
H2-10-A	CDRH2	1KEM	H	FIRNKAKGYT	house mouse	IGHV7-3
H2-10-A	CDRH2	3IJS	D	FIRNKAKGYT	house mouse	IGHV7-3
H2-10-A	CDRH2	1BFO	B	FIRDKAKGYT	Norway rat	IGHV7S6
H2-10-A	CDRH2	1BFO	H	FIRDKAKGYT	Norway rat	IGHV7S6
H2-10-A	CDRH2	1Q9W	B	FIRNKPKGYT	house mouse	IGHV7-3
H2-10-A	CDRH2	3OKK	B	FIRNKAKGYT	house mouse	IGHV7-3
H2-10-A	CDRH2	1Q1J	H	RIKSRTDGGT	human	IGHV3-15
H2-10-A	CDRH2	1CLO	H	FIGNKANGYT	house mouse	IGHV7-3
H2-10-A	CDRH2	2BMK	H	LIRNKANGYT	house mouse	IGHV7-3
H2-10-A	CDRH2	1M71	B	EIRLKSNNYA	house mouse	IGHV6-6
H2-10-A	CDRH2	3L1O	H	LIRNKAKGYT	house mouse	IGHV7-3
H2-10-A	CDRH2	3HZM	B	FIRNKAKGYT	house mouse	IGHV7-3
H2-10-A	CDRH2	3PHO	B	FIRNKGNGYT	house mouse	IGHV7-3
H2-10-A	CDRH2	1Q9K	B	FIRNKANGYT	house mouse	IGHV7-3
H2-10-A	CDRH2	3HZV	B	FIRNKAKGYT	house mouse	IGHV7-3
H2-10-A	CDRH2	3C6S	B	EIRLKSDNYA	house mouse	IGHV6-3
H2-10-A	CDRH2	3GHB	I	RIKSRTDGGT	human	IGHV3-15
H2-10-A	CDRH2	3DUR	B	FIRNKAKGYT	house mouse	IGHV7-3
H2-10-A	CDRH2	3IKC	B	FIRNKAKGYT	house mouse	IGHV7-3
H2-10-A	CDRH2	3V0V	A	LIRNKRNGDT	house mouse	IGHV7-3
H2-10-A	CDRH2	2ZKH	H	EIRSKVNNHA	house mouse	IGHV6-6
H2-10-A	CDRH2	1FL6	H	FIRNKANGYT	house mouse	IGHV3-72
H2-10-A	CDRH2	1SBS	H	DIRLKSNNYA	house mouse	IGHV6-6

H2-10-A	CDRH2	3IJS	B	FIRNKAKGYT	house mouse	IGHV7-3
H2-10-A	CDRH2	2R23	B	FIRNKANGYT	house mouse	IGHV7-3
H2-10-A	CDRH2	4M1D	I	RIKSRTDGGT	human	IGHV3-15
H2-10-A	CDRH2	2R2B	B	FIRNKANGYT	house mouse	IGHV7-3
H2-10-A	CDRH2	2R1X	B	FIRNKANGYT	house mouse	IGHV7-3
H2-10-A	CDRH2	1Q9L	B	FIRNKANGYT	house mouse	IGHV7-3
H2-10-A	CDRH2	3GGW	B	EIRLKSDNYA	house mouse	IGHV6-3
H2-10-A	CDRH2	3IKC	D	FIRNKAKGYT	house mouse	IGHV7-3
H2-10-A	CDRH2	3OKM	B	FIRNKAKGYT	house mouse	IGHV7-3
H2-10-A	CDRH2	1AXT	H	EIRLKSDNYA	house mouse	IGHV6-3
H2-10-A	CDRH2	3V0W	H	LIRNKRNGDT	house mouse	IGHV7-3
H2-10-A	CDRH2	3I2C	H	RIRSKSHNYA	house mouse	IGHV10-3
H2-10-A	CDRH2	3SY0	B	FIRNKANGYT	house mouse	IGHV7-3
H2-10-A	CDRH2	3NZH	H	QIRLKSYNYA	house mouse	IGHV6-3
H2-10-A	CDRH2	1Q72	H	EIRNKANNHA	house mouse	IGHV6-6
H2-10-A	CDRH2	3GGW	D	EIRLKSDNYA	house mouse	IGHV6-3
Unclustered	CDRH2	1OHQ	B	SIYGPSGS	human	IGHV3-23
Unclustered	CDRH2	3Q6G	I	LISGSGGS	human	IGHV4-59
Unclustered	CDRH2	2QTE	A	RYYSSNSGY	channel catfish	NITR11
Unclustered	CDRH2	3RA7	H	SINIGATY	house mouse	IGHV5-9-3
Unclustered	CDRH2	2AJU	H	YIRSSVI	house mouse	IGHV3-2
Unclustered	CDRH2	1A6V	J	RIDPNSGG	house mouse	IGHV1-72
Unclustered	CDRH2	3UC0	I	YAHSRVS	human	IGHV4-59
Unclustered	CDRH2	3SKJ	I	RIGPSGGP	human	IGHV3-23
Unclustered	CDRH2	2V7N	F	SIWYSGSN	human	IGHV3-30
Unclustered	CDRH2	3UC0	H	YAHSRVS	human	IGHV4-59
Unclustered	CDRH2	2FJF	T	GITPAGGY	human	IGHV3-23
Unclustered	CDRH2	3QEG	H	GIIPIFDV	human	IGHV1-69
Unclustered	CDRH2	2AJS	H	YIRSSVI	house mouse	IGHV3-2
Unclustered	CDRH2	3H3P	H	GVIPLLTI	human	IGHV1-69
Unclustered	CDRH2	1OPG	H	AISGGGTY	house mouse	IGHV5S4
Unclustered	CDRH2	3QCV	H	LINPGSDY	house mouse	IGHV5-51
Unclustered	CDRH2	2FJF	X	GITPAGGY	human	IGHV3-23
Unclustered	CDRH2	1CFV	H	AINSDGEP	house mouse	IGHV5-6-2
Unclustered	CDRH2	2V7N	H	SIWYSGSN	human	IGHV3-30
Unclustered	CDRH2	2Q76	B	EILPGSDS	house mouse	IGHV1-9
Unclustered	CDRH2	3BT2	H	WIFHGSDN	house mouse	IGHV1-66
Unclustered	CDRH2	1UWE	V	GIDPHNGG	house mouse	IGHV1S135
Unclustered	CDRH2	2A6J	B	YINPGNGY	house mouse	IGHV1-58
Unclustered	CDRH2	2HMI	D	TIWWDDD	house mouse	IGHV8-12
Unclustered	CDRH2	4JO3	I	CIYGGGRDI	rabbit	IGHV1S40
Unclustered	CDRH2	2AJ3	B	NVYDSGD	human	IGHV4-59
Unclustered	CDRH2	1BZQ	K	AMDSGGGG	Arabian camel	IGHV1S45
Unclustered	CDRH2	1BLN	B	TISSGGGN	house mouse	IGHV5-9
Unclustered	CDRH2	1NJ9	H	YIDPSNGD	house mouse	IGHV1S135
Unclustered	CDRH2	2GK0	B	VIWSGGS	house mouse	IGHV2-9
Unclustered	CDRH2	2AK1	H	YIRSSVI	house mouse	IGHV3-2
Unclustered	CDRH2	2J4W	H	TISDGNSY	house mouse	IGHV5-4
Unclustered	CDRH2	4F58	I	FVPFDRRI	human	IGHV3-30
Unclustered	CDRH2	3SKJ	H	RIGPSGGP	human	IGHV3-23

Unclustered	CDRH2	1F2X	L	TILGGS	Arabian camel	IGHV1S45
Unclustered	CDRH2	4DN4	H	GIIPIFGT	human	IGHV1-69
Unclustered	CDRH2	3FZU	H	RISSSGDP	human	IGHV3-11
Unclustered	CDRH2	4DKF	H	HIDWYGGD	human	IGHV3-23
Unclustered	CDRH2	4NC1	C	AGSSTGRT	llama	IGHV1S3
Unclustered	CDRH2	1TZG	I	GVIPLLTI	human	IGHV1-69
Unclustered	CDRH2	3QHZ	I	LIDWDDDTYY	human	IGHV2-70
Unclustered	CDRH2	1IGT	B	YISNGGGS	house mouse	IGHV5-12
Unclustered	CDRH2	3C08	H	EFNPSNGR	human	IGHV1-46
Unclustered	CDRH2	2QTE	C	RYYSSNSGY	channel catfish	NITR11
Unclustered	CDRH2	1JGV	H	SIYNGFR	house mouse	IGHV5S21
Unclustered	CDRH2	2A6J	H	YINPGNGY	house mouse	IGHV1-58
Unclustered	CDRH2	2V7N	D	SIWYSGSN	human	IGHV3-30
Unclustered	CDRH2	2V7N	B	SIWYSGSN	human	IGHV3-30
Unclustered	CDRH2	2FJF	I	GITPAGGY	human	IGHV3-23
Unclustered	CDRH2	1PG7	I	LIDPEQGN	human	IGHV3-66
Unclustered	CDRH2	3GJE	H	GIVSSGGS	human	IGHV3-23
Unclustered	CDRH2	2FJG	H	GITPAGGY	human	IGHV3-23
Unclustered	CDRH2	4ERS	H	VMYYDGSN	human	IGHV3-30
Unclustered	CDRH2	4B5E	B	AINLNGGR	llama	IGHV1S3
Unclustered	CDRH2	2FJF	P	GITPAGGY	human	IGHV3-23
Unclustered	CDRH2	3U6R	H	NIIPVYNT	human	IGHV1-69
Unclustered	CDRH2	3KYM	N	WIGPSGGI	human	IGHV3-23
Unclustered	CDRH2	2QQQ	B	RYYSSNSGY	channel catfish	NITR11
Unclustered	CDRH2	2XTJ	D	GINPILGI	human	IGHV1-69
Unclustered	CDRH2	2BFV	H	AINSDGEP	house mouse	IGHV5-6-2
Unclustered	CDRH2	3QHZ	H	LIDWDDDTYY	human	IGHV2-70
Unclustered	CDRH2	4HIX	H	SIRSGGGR	house mouse	IGHV3-7
Unclustered	CDRH2	2AJ3	D	NVYDSGD	human	IGHV4-59
Unclustered	CDRH2	3Q6G	H	LISGSGGS	human	IGHV4-59
Unclustered	CDRH2	1IGJ	B	YISPYSGV	house mouse	IGHV1-34
Unclustered	CDRH2	4HXA	H	VITVKSDIYG	house mouse	IGHV13-2
Unclustered	CDRH2	2Z91	A	YIHYRGT	house mouse	IGHV3-1
Unclustered	CDRH2	4JO2	I	CIETGSSDS	rabbit	IGHV1S40
Unclustered	CDRH2	3GJF	H	GIVSSGGS	human	IGHV3-23
Unclustered	CDRH2	2QTE	D	RYYSSNSGY	channel catfish	NITR11
Unclustered	CDRH2	3LMJ	H	GFGPEENE	human	IGHV1-24
Unclustered	CDRH2	1DBM	H	WINIYTGE	house mouse	IGHV9-3-1
Unclustered	CDRH2	4F58	K	FVPFDRRI	human	IGHV3-30
Unclustered	CDRH2	1FAI	H	YINPGKGY	house mouse	IGHV1-58
Unclustered	CDRH2	2H1P	H	TINSNGDK	house mouse	IGHV5-6-2
Unclustered	CDRH2	1YZZ	B	SISSPGT	Arabian camel	IGHV1S17
Unclustered	CDRH2	3NPS	B	GIIPIFGT	human	IGHV1-69
Unclustered	CDRH2	2X1O	B	TMVWSSDT	llama	IGHV1S1
Unclustered	CDRH2	1IND	H	TTLSGGGF	house mouse	IGHV5-9
Unclustered	CDRH2	2FJF	H	GITPAGGY	human	IGHV3-23
Unclustered	CDRH2	1BZQ	L	AMDSGGGG	Arabian camel	IGHV1S45
Unclustered	CDRH2	2AJ3	F	NVYDSGD	human	IGHV4-59
Unclustered	CDRH2	2W9D	H	NVYPNNGV	house mouse	IGHV1-34
Unclustered	CDRH2	3FCT	D	MIDPNSGG	house mouse	IGHV1S36

Unclustered	CDRH2	1UWG	Y	GIDPHNGG	house mouse	IGHV1S135
Unclustered	CDRH2	2XXC	B	AIRGGDMS	alpaca	IGHV
Unclustered	CDRH2	2AJY	H	YIRSSVI	house mouse	IGHV3-2
Unclustered	CDRH2	4JG0	H	SISTPRGST	human	IGHV3-23
Unclustered	CDRH2	4F58	H	FVPFDRRI	human	IGHV3-30
Unclustered	CDRH2	1BGX	H	YITYSGT	house mouse	IGHV3-2
Unclustered	CDRH2	2QQN	H	QISPAGGY	human	IGHV3-23
Unclustered	CDRH2	2BSE	D	ALNFDYDM	llama	IGHV1S3
Unclustered	CDRH2	4NBY	B	AGSSTGRT	llama	IGHV1S3
Unclustered	CDRH2	1ZV5	A	FINSDGGI	Arabian camel	IGHV1S47
Unclustered	CDRH2	2R8S	H	YISSSYGY	human	IGHV3-48
Unclustered	CDRH2	3QCU	H	LINPGSDY	house mouse	IGHV5-51
Unclustered	CDRH2	4JO4	I	CIYGGGRDI	rabbit	IGHV1S40
Unclustered	CDRH2	3R1G	H	WISPAGGS	human	IGHV3-23
Unclustered	CDRH2	1MCP	H	ASRNKGNKYT	house mouse	IGHV7-1
Unclustered	CDRH2	1AY1	H	YITYSGT	house mouse	IGHV3-2
Unclustered	CDRH2	2FJF	D	GITPAGGY	human	IGHV3-23
Unclustered	CDRH2	1R0A	H	TIWWDDD	house mouse	IGHV8-12
Unclustered	CDRH2	4GRW	I	AIRWSGGS	llama	IGHV1S3
Unclustered	CDRH2	2AJZ	H	YIRSSVI	house mouse	IGHV3-2
Unclustered	CDRH2	1BZQ	M	AMDSGGGG	Arabian camel	IGHV1S45
Unclustered	CDRH2	2V7H	B	EILPGSGS	house mouse	IGHV1-9
Unclustered	CDRH2	1BFV	H	AINSDGEP	house mouse	IGHV5-6-2
Unclustered	CDRH2	2FJF	N	GITPAGGY	human	IGHV3-23
Unclustered	CDRH2	4FNL	I	IINAGGGD	human	IGHV3-23
Unclustered	CDRH2	3W9D	A	RTIPLFGK	house mouse	IGHV1-69
Unclustered	CDRH2	2QTE	B	RYYSSNSGY	channel catfish	NITR11
Unclustered	CDRH2	1BM3	H	AISGGGTY	house mouse	IGHV5S4
Unclustered	CDRH2	1MRF	H	EIDPSDSY	house mouse	IGHV1-50
Unclustered	CDRH2	2X1O	A	TMVWSSDT	llama	IGHV1S1
Unclustered	CDRH2	1IGJ	D	YISPYSGV	house mouse	IGHV1-34
Unclustered	CDRH2	2FJF	K	GITPAGGY	human	IGHV3-23
Unclustered	CDRH2	1UWE	H	GIDPHNGG	house mouse	IGHV1S135
Unclustered	CDRH2	1YC7	B	SISSPGT	Arabian camel	IGHV1S55
Unclustered	CDRH2	2FJF	B	GITPAGGY	human	IGHV3-23
Unclustered	CDRH2	4JO4	H	CIYGGGRDI	rabbit	IGHV1S40
Unclustered	CDRH2	3EAK	B	AIASMGGL	Arabian camel	IGHV1S27
Unclustered	CDRH2	3TNM	H	YIHYSGN	human	IGHV4-30-4
Unclustered	CDRH2	1RMF	H	VISAYSGD	house mouse	IGHV1-67
Unclustered	CDRH2	4AG4	H	NIYPSGGY	house mouse	IGHV1-61
Unclustered	CDRH2	4JG1	H	SISTPRGST	human	IGHV3-23
Unclustered	CDRH2	1MAM	H	FIRNKADGYT	house mouse	IGHV7-3
Unclustered	CDRH2	1INE	H	TTLSGGGF	house mouse	IGHV5-9
Unclustered	CDRH2	4NBY	C	AGSSTGRT	llama	IGHV1S3
Unclustered	CDRH2	2AJV	H	YIRSSVI	house mouse	IGHV3-2
Unclustered	CDRH2	3EAK	A	AIASMGGL	Arabian camel	IGHV1S27
Unclustered	CDRH2	2P4A	D	AMDSGGGG	Arabian camel	IGHV1S45
Unclustered	CDRH2	3H3P	I	GVIPLLTI	human	IGHV1-69
Unclustered	CDRH2	4LMQ	E	ALWGSGG	human	IGHV3-23
Unclustered	CDRH2	1F2X	K	TILGGS	Arabian camel	IGHV1S45

Unclustered	CDRH2	1EO8	H	EILPGSDN	house mouse	IGHV1-9
Unclustered	CDRH2	2QQQ	D	RYYSSNSGY	channel catfish	NITR11
Unclustered	CDRH2	1UWG	H	GIDPHNGG	house mouse	IGHV1S135
Unclustered	CDRH2	1BZ7	B	YISSGGSS	house mouse	IGHV5-17
Unclustered	CDRH2	4F37	F	HIYWDDD	house mouse	IGHV8-12
Unclustered	CDRH2	6FAB	H	YNNPGNGY	house mouse	IGHV1-58
Unclustered	CDRH2	4LAJ	H	CISDSDGR	llama	IGHV1S1
Unclustered	CDRH2	3MCL	H	EVLPGSGK	house mouse	IGHV1-9
Unclustered	CDRH2	3GK8	H	WIFPGDGS	house mouse	IGHV1-85
Unclustered	CDRH2	1JGU	H	SIYNGFR	house mouse	IGHV5S21
Unclustered	CDRH2	4GRW	H	AIRWSGGS	llama	IGHV1S3
Unclustered	CDRH2	4G3Y	H	EIRSKSINSA	house mouse	IGHV6-6
Unclustered	CDRH2	1C1E	H	WINPYTGE	house mouse	IGHV9-1
Unclustered	CDRH2	2AJZ	B	YIRSSVI	house mouse	IGHV3-2
Unclustered	CDRH2	4DKF	I	HIDWYGGD	human	IGHV3-23
Unclustered	CDRH2	4F57	H	FVPFDRRI	human	IGHV3-30
Unclustered	CDRH2	2OJZ	H	WVYPGS	house mouse	IGHV1-84
Unclustered	CDRH2	3ULS	H	IIQKRSKWY	human	IGHV6-1
Unclustered	CDRH2	3QSK	B	AMDSGGGG	Arabian camel	IGHV1S45
Unclustered	CDRH2	4F58	J	FVPFDRRI	human	IGHV3-30
Unclustered	CDRH2	4LAJ	L	CISDSDGR	llama	IGHV1S1
Unclustered	CDRH2	2FD6	H	WIFHGSDN	house mouse	IGHV1-66
Unclustered	CDRH2	2FJF	R	GITPAGGY	human	IGHV3-23
Unclustered	CDRH2	4AT6	G	VIYPGNGD	house mouse	IGHV1-12
Unclustered	CDRH2	2P4A	B	AMDSGGGG	Arabian camel	IGHV1S45
Unclustered	CDRH2	4NC1	D	AGSSTGRT	llama	IGHV1S3
Unclustered	CDRH2	3U6R	A	NIIPVYNT	human	IGHV1-69
Unclustered	CDRH2	1OSP	H	YIRYGGG	house mouse	IGHV3-8
Unclustered	CDRH2	1D6V	H	EILPGSGS	house mouse	IGHV1-9
Unclustered	CDRH2	1BZQ	N	AMDSGGGG	Arabian camel	IGHV1S45
Unclustered	CDRH2	2BSE	F	ALNFDYDM	llama	IGHV1S3
Unclustered	CDRH2	4JN2	B	VIWAGGS	house mouse	IGHV2-9
Unclustered	CDRH2	2BSE	E	ALNFDYDM	llama	IGHV1S3
Unclustered	CDRH2	4DGV	H	VIWFDENN	human	IGHV3-33
Unclustered	CDRH2	2Z4Q	B	NIYPGSGG	house mouse	IGHV1S5
Unclustered	CDRH2	2FJG	B	GITPAGGY	human	IGHV3-23
Unclustered	CDRH2	4LAJ	I	CISDSDGR	llama	IGHV1S1
Unclustered	CDRH2	2OJZ	I	WVYPGS	house mouse	IGHV1-84
Unclustered	CDRH2	2AJX	H	YIRSSVI	house mouse	IGHV3-2
Unclustered	CDRH2	1AD0	B	FIGNKANGYT	human	IGHV3-72
Unclustered	CDRH2	1PG7	H	LIDPEQGN	human	IGHV3-66
Unclustered	CDRH2	2FJF	V	GITPAGGY	human	IGHV3-23
Unclustered	CDRH2	2QJD	A	RYYSSNSGY	channel catfish	NITR11
Unclustered	CDRH2	1YC7	A	SISSPGT	Arabian camel	IGHV1S55
Unclustered	CDRH2	3KYM	H	WIGPSGGI	human	IGHV3-23
Unclustered	CDRH2	4LMQ	H	ALWGSGG	human	IGHV3-23
Unclustered	CDRH2	4FNL	H	IINAGGGD	human	IGHV3-23
Unclustered	CDRH2	3KYM	P	WIGPSGGI	human	IGHV3-23
Unclustered	CDRH2	4HJJ	H	HIYWDDD	human	IGHV2-70
Unclustered	CDRH2	2BJM	H	RIDPNGGG	house mouse	IGHV1-72

Unclustered	CDRH2	1A5F	H	RIDSGSSN	house mouse	IGHV14-3
Unclustered	CDRH2	1UWE	Y	GIDPHNGG	house mouse	IGHV1S135
Unclustered	CDRH2	3SE9	H	WVKTVTGAV	human	IGHV1-2
Unclustered	CDRH2	3FZU	C	RISSSGDP	human	IGHV3-11
Unclustered	CDRH2	2Q76	D	EILPGSDS	house mouse	IGHV1-9
Unclustered	CDRH2	4JN2	H	VIWAGGS	house mouse	IGHV2-9
Unclustered	CDRH2	3H0T	B	RTYYRSKWF	human	IGHV6-1
Unclustered	CDRH2	2QQQ	C	RYYSSNSGY	channel catfish	NITR11
Unclustered	CDRH2	4JO2	H	CIETGSSDS	rabbit	IGHV1S40
Unclustered	CDRH2	1OHQ	A	SIYGPSGS	human	IGHV3-23
Unclustered	CDRH2	3GJF	M	GIVSSGGS	human	IGHV3-23
Unclustered	CDRH2	4FQI	H	GISPIFGS	human	IGHV1-69
Unclustered	CDRH2	3GJE	B	GIVSSGGS	human	IGHV3-23
Unclustered	CDRH2	2QQQ	A	RYYSSNSGY	channel catfish	NITR11
Unclustered	CDRH2	3HC0	H	WIYPGNVH	human	IGHV1-69
Unclustered	CDRH2	4JO3	H	CIYGGGRDI	rabbit	IGHV1S40
Unclustered	CDRH2	1IGM	H	GVFGSGGN	human	IGHV3-23
Unclustered	CDRH2	4B5E	A	AINLNGGR	llama	IGHV1S3
Unclustered	CDRH2	2QJD	B	RYYSSNSGY	channel catfish	NITR11
Unclustered	CDRH2	2FJF	F	GITPAGGY	human	IGHV3-23
Unclustered	CDRH2	4DGY	H	VIWFDENN	human	IGHV3-33
Unclustered	CDRH2	4LEX	H	YIGFSGS	house mouse	IGHV3-8
Unclustered	CDRH2	4JO1	H	CIETGSSDS	rabbit	IGHV1S40
Unclustered	CDRH2	4KRN	A	AIRWSGGY	llama	IGHV1S1
Unclustered	CDRH2	2F19	H	YINPGKGY	house mouse	IGHV1-58
Unclustered	CDRH2	4JO1	I	CIETGSSDS	rabbit	IGHV1S40
Unclustered	CDRH2	3GRW	H	RIYPTNGS	human	IGHV3-66
Unclustered	CDRH2	2OK0	H	WVYPGS	house mouse	IGHV1-84
Unclustered	CDRH2	2P49	B	AMDSGGGG	Arabian camel	IGHV1S45
