# Supplementary material for: Length-independent structural similarities enrich the antibody CDR canonical class model
Source: MAbs. 2016 Mar 10;8(4):751–60. doi: 10.1080/19420862.2016.1158370 (PMC4966832; doi:10.1080/19420862.2016.1158370)
Supplement: Supplemental_Datas.zip [file kmab-08-04-1158370-s001.zip › 2015MABS1071R-s02.docx]

#### Clustering details

Here we describe in detail the clustering results for each non-H3 CDR type. We include descriptions of interactions that stabilize the loop structures and describe sequence patterns. The clusters are ordered first by length of the shortest loop, then by number of structures and, finally, by number of sequences. The clustering results are summarized in Tables S1-S5.

##### 1. CDR-L1 clusters

CDR-L1 is the second most length variable of all CDR types, behind CDR-H3. The shortest loops of this type are of length seven and the longest of length 17. There are 17 clusters in total, 10 containing at least six sequences, two of which contain loops of more than one length (Table S1).

The cluster L1-10,11,12-A contains the majority of loops of length 10 and 11 and three loops of length 12 (Figure S1A). The most common interaction, present in 194 out of 204 sequences in this cluster, is the hydrogen bond formed between a Ser at position 26 and the backbone of the residue at position 3. In addition, all CDRs in this cluster contain a hydrophobic residue at position 33, the side chain of which is buried inside the loop structure.

The second largest cluster of CDR-L1s of length 11 is L1-11-A, coded for by germlines from the lambda Immunoglobulin locus. Loops in this cluster do not have a Ser at position 26; instead all loops in this cluster display an interaction between residues at positions 27 and 30 creating a more ”compact” structure that is aided by presence of two Gly (present in all loops in this cluster) at positions 25 and 29.

The third cluster containing loops of length 11 - L1-11-B contains structures which in all cases are stabilized by the hydrogen bond between the backbone oxygen of Leu at Chothia position 28 and the backbone nitrogens of residues 25 and 31.

Many of the length 12 CDR-L1s not in cluster L1-10,11,12-A are found in cluster L1-12-A which consists of 12 unique sequences. In 11 of the 12 unique sequences in this cluster, there is a hydrogen bond between the sidechain of the residue at position 30 and the backbone of residue 31, an interaction not present in L1-10,11,12-A.

Most unique sequences of length 13 and 14 belong to cluster L1-13,14-A, which forms a compact shape stabilized by a number of interactions. All of the CDRs in this cluster form hydrogen bonds between the backbones of residues 26 and 29, 26 and 30A, and 29 and 30B (Figure S1B). The loops in clusters L1-13-A and L1-14-A, which contain the remaining structures of length 13 and 14, respectively, lack the aforementioned interactions which results in a more extended shape without the ”hoop” in the centre of the CDR.

The majority of the longer CDR-L1 loops are contained in clusters L1-15-A, L1-16-A and L1-17-A. The first part of long CDR-L1 loops, up to the central hydrophobic residue at position 29, resembles the loops in cluster L1-10,11,12-A. The remaining part of the loop structure is a protrusion of varying length stabilized by hydrogen bonds between backbone atoms (see Figure S1C).

##### 2. CDR-L2 clusters

CDR-L2 is the least variable in terms of length of all CDRs, having only two lengths in our set - seven or 11. There are five clusters and only two which contain more than six unique sequences (Table S2).

The vast majority of the CDR-L2s of length seven are in cluster L2-7-A. This shape occurs universally across all available species and in both *κ* and *λ* light chains. The second largest cluster is L2-7-B, also containing loops of length seven. The structures of the CDRs in the two clusters differ only by the conformation of the first three residues. The two clusters containing CDR-L2s of length 11, L2-11-A and L2-11-B, are very small and contain only two and three sequences respectively. L2-11-B is more diverse of the two containing two loops from Rhesus Monkey anti-HIV antibodies and a CDR-L2 from a human pre B-cell receptor.

##### 3. CDR-L3 clusters

CDR-L3 loops in our set span lengths between five and 12 residues. The majority of CDR-L3 loops are of length nine. There are 11 clusters in total, five containing at least six unique sequences, two of which contain loops of more than one length (Table S3).

All CDR-L3 loops of length five are found in cluster L3-5-A and are all from HIV-1 neutralizing antibodies that recognize the epitope of a CD4 binding site^1^.

The cluster L3-8-A contains the majority of loops of length 8. The structure of these loops is stabilized by Gln at Chothia position 90 forming hydrogen bonds with residues at positions 92 (in 97 out of 106 cases), 93 (in 91 out of 106 cases) and the side-chain of the Thr at position 97 (in 86 out of 106 cases) (Figure S1D).

L3-9,10-A contains the largest number of CDR-L3 loops and contains loops of length nine and ten. Most loops of length nine belong in this cluster. The loops in this cluster are stabilized by the same interactions as the loops in cluster L3-8-A the difference being that 325 out of 335 of loops of length nine contain a Pro in cis conformation at position 95 and all loops of length ten contain two Pro at positions 95 and 95A. The Pro create a sharp turn making the loop conformations similar for loops of both lengths (Figure S1E).

Cluster L3-9-A is the second largest cluster containing loops of length nine (107 structures, 22 sequences). The residue at position 91 usually contains a large side-chain with an aromatic ring (Trp, Tyr, Phe). The CDR conformation is stabilized, in 20 out of 22 cases, by a hydrogen bond between backbone carboxyl oxygen of residue 92 and backbone nitrogen of residue 94.

Figure S1F illustrates the differences in conformations between CDR-L3 loops of length nine in cluster L3-9,10-A and in cluster L3-9-A.

The structures of CDR-L3 loops that are part of the cluster L3-10,11-A are stabilized by similar interactions to the CDRs in cluster L3-9-A. The difference is the presence of a highly conserved Asp/Asn forming a hydrogen bond with the residue at position 94 creating a bend in the structure (Figure S1G). In all CDRs in this cluster there is a hydrogen bond between backbone nitrogen of residue at position 92 and the backbone oxygen of residue at position 95. In 17 out of 23 sequences, there is also a hydrogen bond between backbone nitrogen at position 93 and the backbone carboxyl oxygen of residue 28, which is a part of the CDR-L1 loop.

##### 4. CDR-H1 clusters

CDR-H1 loops are three to 13 residues long. CDRs of length seven are most prevalent (87% of all structures). There are 14 clusters, but out of these only four contain at least six unique sequences. There are no clusters containing loops of more than one length. Overall, this CDR type contains the largest number of small clusters, mostly coming from camelid antibodies (Table S4).

The largest cluster of CDR-H1 is H1-7-A containing 73% of all H1 structures. CDRs in this cluster tend to include (221 out of 257 sequences) a residue with a large aromatic ring at position 27 (Phe or Tyr) and, in 218 out of 257 of cases, a hydrogen bond between backbone atoms of residues 28 and 31.

The second cluster containing CDRs of length seven is H1-7-B. The difference in conformation between CDR-H1 loops in H1-7-A and H1-7-B is subtle, but most pronounced around residues 28 and 31.

The other two large clusters are H1-8-A and H1-9-A, containing CDRs of length eight and 10, respectively. All structures in cluster H1-8-A contain a hydrogen bond between the backbone atoms of residues 28 and 31. The structures of loops in cluster H1-9-A are similar to H1-8-A, except for a large “bulge” around residue 32. All CDR structures in cluster H1-9-A contain a bond between backbone atoms of residues 31 and 32.

##### 5. CDR-H2 clusters

CDR-H2 loops in our set show only six lengths, between seven and 12 residues. Most of the CDRs of this type are of length seven, eight or 10. There are 12 clusters in total, five containing at least six unique sequences (Table S5). There are no clusters containing loops of more than one length but the conformations in clusters H2-7-A (containing CDRs of length seven) and H2-8-A (containing CDRs of length eight) are similar.

The majority of CDR-H2s of length seven belong to cluster H2-7-A. The sequence pattern for this cluster shows a preference for Gly at position 55 and a residue with side-chain oxygen at position 56 (Ser/Thr/Asn/Asp). In almost all structures (90 out of 91) there is a bond between residues at positions 52 and 55.

There are two clusters containing loops of length eight - H2-8-A and H2-8-B. In cluster H2-8-A, 195 out of 197 structures contain a bond between backbone atoms of residues 52 and 55 (same as H2-7-A). In addition, 154 loops contain a Pro at position 52A and a Gly at position 56. The structures of CDRs in this cluster are similar to H2-7-A with the Pro-52A creating a sharp turn in the loop.

In contrast, the CDR structures in cluster H2-8-B lack the Pro-52A and the hydrogen bond between backbone atoms of residues 52 and 56. Instead, 92 out of 93 structures contain a hydrogen bond between residues 52 and 54, and, in 76 cases a hydrogen bond between side-chain of residue 52 and residue 56. In 84 cases, the conformation in cluster H2-8-B is also stabilized by the hydrogen bond between the side-chain nitrogen of the framework residue Arg-71 and the backbone oxygen of residue at position 52. The importance of the identity of residue 71 for the conformation of CDR-H2 has been described before^2,3^.

Most of the CDR-H2 loops of length 10 are concentrated in cluster H2-10-A. Loops of this length are usually from mouse antibodies. All structures in this clusters contain hydrogen bonds between residues 52A and 55 and 52B and 54. The structures of loops in this cluster in 23 out of 25 cases also display the interaction between Arg-71 and the backbone carboxyl oxygen of residue at position 52.

As discussed in the main text it is possible for CDRs of the same sequence to belong to different clusters, this structural variation is most common in CDR-H2. For example, the CDR-H2 loop with sequence EILPGSGS in the unliganded structure 1MLB_B belongs to cluster H2-8-A but in the bound form, in structures 1MLC_B and 1MLC_D, the loop belongs to cluster H2-8-D with 2.1 Å difference in RMSD (1MLB are 1MLC are crystal structures of the same antibody). YIWPSGGN is the sequence of CDR-H2 loops in structures 3HI6_X and 3HI6_H and in 3HI6_X the loop belongs to cluster H2-8-B but in 3HI6_H the loop is part of cluster H2-8-H. This result implies there are CDRs which can exist in different structural states. Unfortunately, it will also confuse cluster prediction from sequence.

References

1. Wu X, Zhou T, Zhu J, Zhang B, Georgiev I, Wang C, Chen X, Longo NS, Louder M, McKee K, et al. Focused evolution of HIV-1 neutralizing antibodies revealed by structures and deep sequencing. Science (80- ) [Internet] 2011; 333:1593–602. Available from: http://www.sciencemag.org/content/333/6049/1593.short;

2. North B, Lehmann A, Dunbrack Jr RL. A new clustering of antibody CDR loop conformations. J Mol Biol [Internet] 2011; 406:228–56. Available from: http://www.sciencedirect.com/science/article/pii/S0022283610011496;

3. Tramontano A, Chothia C, Lesk AM. Framework residue 71 is a major determinant of the position and conformation of the second hypervariable region in the VH domains of immunoglobulins. J Mol Biol [Internet] 1990; 215:175–82. Available from: http://www.sciencedirect.com/science/article/pii/S0022283605801020


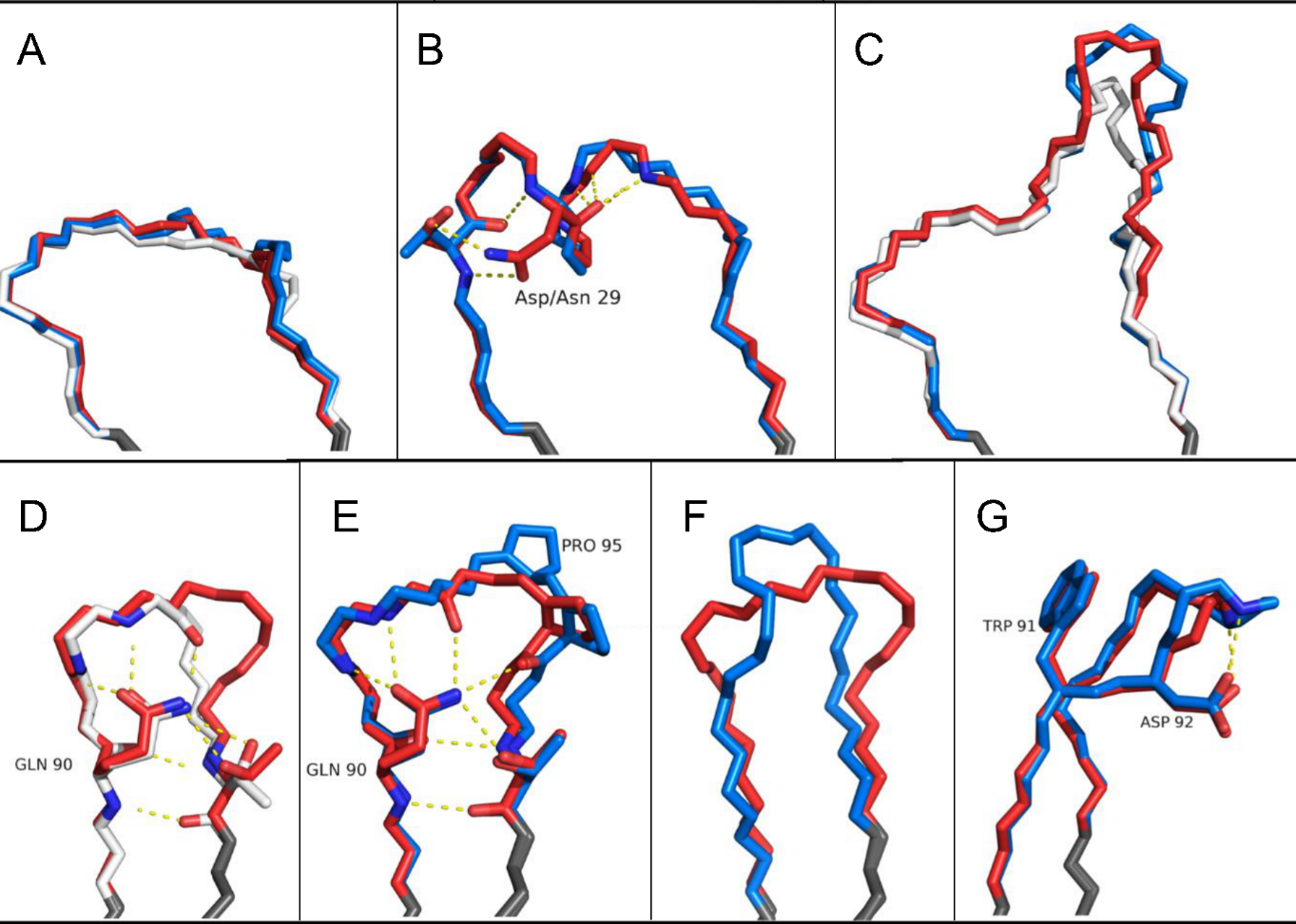


Figure S1: CDRs with different lengths, but similar structures, with their anchors aligned, shown in grey. A) CDR-L1 of 4F33_E (length 10, white), 3SOB_L (length 11, red) and 3EO0_A (length 12, blue) from cluster L1-10,11,12-A. The three CDRs are structurally similar, except for the insertion site at position 30. B) CDR-L1 loops of 4FQJ_L (length 13, red) and 3U2S_L (length 14, blue) from cluster L1-13,14-A. Presence of Asp/Asn at position 29 (the side chain of which is shown in the figure, along with the hydrogen bonds it makes) creates a ”hoop” in the structures of both lengths in cluster L1-13,14-A. C) CDR-L1 of 3QRG L (length 15, white, cluster L1-15-A), 1KFA M (length 16, red, cluster L1-16-A) and 2R1X_ A (length 17, blue, cluster L1-17-A). The structures of longer CDR-L1 loops containing a “protrusion” starting around residue 29. D) CDR-L3 of 4HGW_A, (length 8, white, cluster L3-8-A) and 3RVV_C, (length 9, red, cluster L3-9,10-A). The conformations of CDRs in both clusters appear to be maintained by Gln at position 90 (the side chain of which is shown, along with the contacts to other residues). E) CDR-L3 of 3RVV_C, (length 9, red) and 4HHA_A, (length 10, blue) from cluster L3-9,10-A. CDRs of both length have similar conformations, maintained by the Gln at position 90, with the CDR of length 10 containing an additional Pro at position 95A (The side chains of both Pro are shown in the figure). F) CDR-L3 of 3RVV_C (length 9, red, cluster L3-9,10-A) and 1Y0L_C (length 9, blue, cluster L3-9-A). The alignment shows the difference in conformation between the two largest clusters containing CDRs of length 9. G) CDR-L3 of 3MLX L, (length 10, red) and 4NZT_L, (length 11, blue) from cluster L3-10,11-A. The conformation of both CDRs seems to be stabilized by the interaction between Asn at position 92 and the backbone nitrogen of residue 94 and the presence of large hydrophobic residue at position 91 (the side chains of Asn-92 and Trp-91 are shown in the figure).
