# Supplementary material for: Length-independent structural similarities enrich the antibody CDR canonical class model
Source: MAbs. 2016 Mar 10;8(4):751–60. doi: 10.1080/19420862.2016.1158370 (PMC4966832; doi:10.1080/19420862.2016.1158370)
Supplement: Supplemental_Datas.zip [file kmab-08-04-1158370-s001.zip › 2015MABS1071R-s03.pdf]

Cluster name	Length	Number of structures	Middle structure	Number of unique sequences	Species	Germline
L1-10,11,12-A	10	69	4F33_E	28	Mo	IGKV4
	11	707	3SOB_L	174	Mo, Hu, Ra, Rabbit	IGKV
	12	3	3EOO_A	2	Hu	IGKV3
L1-11-A	11	38	4IMK_C	9	Hu	IGLV3
L1-11-B	11	24	3MLS_M	8	Hu	IGLV3
L1-11-C	11	5	4FQC_L	3	Hu	IGLV3
L1-12-A	12	22	1HQ4_A	12	Hu, Mo	Hu_IGKV, Mo_IGKV4
L1-12-B	12	28	4LLV_L	5	Hu	IGKV3-20*01
L1-12-C	12	13	2OTU_E	3	Mo	IGLV3
L1-12-D	12	7	1HZH_M	3	Hu	IGKV3
L1-13,14-A	13	66	4FQJ_L	23	Hu	IGLV1
	14	51	3U2S_L	14	Hu	IGLV2
L1-13-A	13	23	2WOL_C	6	Hu	IGLV6-57*01
L1-14-A	14	92	1YOL_C	7	Hu, Mo	Mo_IGLV1, Hu_IGLV7
L1-14-B	14	2	4KTD_L	2	Hu	Hu_IGLV5
L1-15-A	15	55	3QRG_L	26	Hu, Mo	IGKV
L1-15-B	15	3	3DGG_C	2	Mo	IGKV3-12*01
L1-16-A	16	273	1KFA_M	65	Hu, Mo	IGKV
L1-17-A	17	113	2R1X_A	31	Hu, Mo	Mo_IGKV8, Hu_IGKV4
L1-17-B	17	3	3LDB_B	2	Ra, Mo	IGKV8
Unclustered	7, 8, 9, 10, 11, 12, 13, 14, 15, 16, 17	104	-	47	-	-

Table S1: Information on canonical forms of CDR-L1. The clusters are ordered first by length, then by number of structures and finally by number of sequences. The germlines are reported in the following way: if over 90% of the structures come from the same allele, that allele is shown in the Table. Otherwise, we report the lowest step in the classification hierarchy that can explain 90% of the data. If two different loci are present in a cluster we report both of them.

Cluster name	Length	Number of structures	Middle structure	Number of unique sequences	Species	Germline
L2-7-A	7	1708	2G5B_A	291	Hu, Mo, Ra,	IGKV, IGLV
L2-7-B	7	21	3I9G_L	6	Hu, Mo	IGKV, IGLV
L2-7-C	7	6	2ABI_A	3	Mo	IGKV10-96*01
L2-11-A	11	13	2GSG_A	2	Mo	IGLV3*01
L2-11-B	11	5	2H3N_C	3	Rhesus monkey, Hu	IGLV, Hu_VPREB1*02
Unclustered	7	9	-	6	-	-

Table S2: Information on canonical forms of CDR-L2. See description below Table S1

Cluster name	Length	Number of Structures	Middle structure	Number of unique structures	Species	Framework germline	Joining germline
L3-5-A	5	17	4JPI_B	6	Hu	IGKV3, IGLV2	IGLJ, IGKJ
L3-7-A	7	2	1DFB_L	2	Hu	IGKV01-5*03	IGKJ3
L3-8-A	8	106	4HGW_A	29	Hu, Mo, Ra	IGKV	IGKJ
L3-8-B	8	9	3VW3_L	4	Mo	IGKV1-117*01	IGKJ
L3-8-C	8	6	2FD6_L	2	Mo	IGKV	IGKJ
L3-8-D	8	3	1TZH_A	2	Mo	IGKV12-44*01	IGKJ
L3-9,10-A	9	1123	3RVV_C	331	Hu, Mo, Ra	IGKV	IGKJ
	10	10	4HHA_A	4	Hu, Mo	Mo_IGKV1-110*01, Hu_IGKV3-11*01	IGKJ5*01
L3-9-A	9	107	1Y0L_C	22	Hu, Mo	IGLV, IGKV4-86*01	IGLJ, IGKL5*01
L3-9-B	9	5	2VXS_O	2	Hu	IGLV6-57*01	IGLJ
L3-9-C	9	2	2HWZ_L	2	Mo	IGKV1-117*01	IGKJ4
L3-10,11-A	10	4	3MLX_L	1	Hu	IGLV1-51*02	IGLJ
	11	49	4NZT_L	22	Hu	IGLV1, IGLV3-21	IGLJ
L3-10-A	10	31	3U2S_L	5	Hu	IGLV2-14*01	IGLJ
L3-10-B	10	9	3U79_B	2	Hu	IGKV1-33*01	IGKJ
L3-10-C	10	4	2DD8_L	3	Hu	IGLV3	IGLJ
L3-10-D	10	3	4JAM_L	2	Hu	IGLV3	IGLJ6
L3-11-A	11	3	3MA9_L	3	Hu	IGLV3, IGKJ1	IGLJ, IGKJ1
L3-12-A	12	4	3QHZ_L	2	Hu	IGLV1-44*01	IGLJ
L3-12-B	12	2	4JY5_L	2	Hu	IGLV3-21	IGLJ
L3-13-A	13	5	2BOS_L	2	Hu	IGLV1-47*01	IGLJ
Unclustered	6, 8, 9, 10, 11, 12 , 13, 19	248	-	80	-	-	-

Table S3: Information on canonical forms of CDR-L3. See description below Table S1

Cluster name	Length	Number of structures	Middle structure	Number of unique sequences	Species	Germline
H1-4-A	4	8	1KXQ_H	3	Camel	IGHV1S45*01
H1-6-A	6	7	2QQQ_B	2	Channel catfish	NITR11
H1-7-A	7	1267	1PLG_H	257	Hu, Mo, Ra, Camel, Llama, Rabbit, Rhesus Monkey, Sheep, Hamster	IGHV
H1-7-B	7	18	4FQQ_F	6	Hu, Mo	Hu_IGHV, Mo_IGHV5S21*01
H1-7-C	7	10	4KPH_H	2	Mo	IGHV3-8*02
H1-7-D	7	8	1BZQ_K	3	Camel, Llama	IGHV1S45
H1-7-E	7	7	4DKA_A	2	Llama	IGV1S3*01
H1-7-F	7	6	4NBZ_D	3	Llama, Mo	Mo_IGHV9-1*01, Llama_IGHV1S3*01
H1-7-G	7	6	3EZJ_B	3	Camel, Llama	IGHV
H1-8-A	8	37	3RVW_D	8	Hu, Mo	Hu_IGHV4-38-2*01, Mo_IGHV3
H1-8-B	8	14	1RVK_H	3	Mo	Mo_IGHV3-2*02
H1-8-C	8	7	1F58_H	2	Hu, Mo	Hu_IGHV4-38-2*01, Mo_IGHV3-1*02
H1-9-A	9	86	3IDN_B	9	Hu, Mo	Mo_IGHV8, Hu_IGHV2
H1-9-B	9	5	3BKJ_H	2	Mo	IGHV8-12*01
Unclustered	3,6,7, 8,9,10, 12,14	248	-	102	-	-

Table S4: Information on canonical forms of CDR-H1. See description below Table S1

Cluster name	Length	Number of structures	Middle structure	Number of unique sequences	Species	Germline
H2-7-A	7	387	3ZKM_H	91	Hu, Mo, Rat, Sheep, Camel, Llama, Rabbit	IGHV
H2-7-B	7	4	4FQC_H	3	Hu	IGHV4
H2-8-A	8	650	1I8M_B	197	Hu, Mo, Rat, Camel, Rhesus Monkey, Llama, Rabbit	IGHV
H2-8-B	8	305	2VXS_K	93	Hu, Mo, Camel, Rat, Llama	IGHV
H2-8-C	8	23	1ZLV_H	2	Hu	IGHV3
H2-8-D	8	19	1YQV_H	9	Hu, Mo	Hu_IGHV1, Mo_IGHV1
H2-8-E	8	8	3OGO_E	3	Hu, Rat, Camel	IGHV
H2-8-F	8	7	2XVM_B	2	Hu, Llama	Hu_IGHV1, Llama_IGHV1S4
H2-8-G	8	6	1ZA6_B	3	Mo	IGHV1
H2-8-H	8	4	3EYV_H	2	Mo, Hu	IGHV
H2-8-I	8	3	3QOS_B	3	Hu, Mo	Mo_IGHV5, Hu_IGHV3
H2-8-J	8	3	1F4X_H	2	Mo	IGHV5
H2-10-A	10	147	3HZV_B	25	Mo, Hu, Ra, Hamster	IGHV
Unclustered	8,9,10, 11,12	213	-	103	-	-

Table S5: Information on canonical forms of CDR-H2. See description below Table S1
