# Supplementary material for: Length-independent structural similarities enrich the antibody CDR canonical class model
Source: MAbs. 2016 Mar 10;8(4):751–60. doi: 10.1080/19420862.2016.1158370 (PMC4966832; doi:10.1080/19420862.2016.1158370)
Supplement: Supplemental_Datas.zip [file kmab-08-04-1158370-s001.zip › 2015MABS1071R-s04.docx]

**This file shows the detailed comparison between our length-independent clustering and the recent clustering of CDR structures by North & Dunbrack** *et al.*

| Our cluster | North & Dunbrack *et al.* | Fraction of CDRs in  North & Dunbrack *et al.* cluster found in our cluster |
| --- | --- | --- |
| **L1-10,11,12-A** | **L1-10-1** | **95%** |
|  | **L1-11-1** | **100%** |
|  | **L1-11-1** | **100%** |
| **L1-11-A** | **L1-11-3** | **40%** |
| **L1-11-B** | **L1-11-3** | **20%** |
| L1-11-C | - | - |
| **L1-12-A** | **L1-12-1** | **100%** |
| L1-12-B | L1-12-2 | 80% |
| L1-12-C | L1-12-3 | 100% |
| L1-12-D | - | - |
| **L1-13,14-A** | **L1-13-1** | **83%** |
|  | **L1-14-2** | **75%** |
| **L1-13-A** | **L1-13-2** | **100%** |
| **L1-14-A** | **L1-14-1** | **100%** |
| L1-14-B | - | - |
| **L1-15-A** | **L1-15-1** | **100%** |
| L1-15-B | L1-15-2 | 100% |
| **L1-16-A** | **L1-16-1** | **90%** |
| **L1-17-A** | **L1-17-1** | **94%** |
| L1-17-B | - | - |

Table S6: The comparison between North & Dunbrack *et al.* work and our clustering for CDR-L1. The first column shows the cluster label in our set while the second contains the labels of corresponding North & Dunbrack *et al.* clusters. The third column shows the fraction of CDRs in North & Dunbrack *et al.* cluster that is contained within our cluster. Two clusters were considered equivalent if our cluster contained at least 50% of CDRs present in the corresponding North & Dunbrack *et al.* cluster. North & Dunbrack *et al.* cluster was considered to be split between two of our clusters if the two clusters together contained at least 50% of CDRs from North & Dunbrack *et al.* cluster (for example the CDRs from North & Dunbrack *et al.* cluster L1-11-3 are split between our clusters L1-11-A and L1-11-B). The clusters in our work which contain at least six unique sequences are shown in bold

| Our cluster | North & Dunbrack *et al.* | Fraction of CDRs in  North & Dunbrack *et al.* cluster found in our cluster |
| --- | --- | --- |
| **L2-7-A** | **L2-8-1** | **100%** |
|  | **L2-8-2** | **100%** |
|  | **L2-8-4** | **100%** |
|  | **L2-8-5** | **100%** |
| L2-7-B | **L2-8-3** | **100%** |
| L2-7-C | - | - |
| L2-11-A | L2-12-2 | 100% |
| L2-11-B | L2-12-1 | 100% |

Table S7: The comparison between North & Dunbrack *et al.* work and our clustering for CDR-L2. See description under Table S6

| Our cluster | North & Dunbrack *et al.* | Fraction of CDRs in  North & Dunbrack *et al.* cluster found in our cluster |
| --- | --- | --- |
| **L3-5-A** | **-** | **-** |
| L3-7-A | L3-7-1 | 100% |
| **L3-8-A** | **L3-8-1** | **93%** |
| L3-8-B | L3-8-2 | 50% |
| L3-8-C | L3-8-cis6-1 | 66% |
| L3-8-D | L3-8-cis6-1 | 33% |
| **L3-9,10-A** | **L3-9-2** | **100%** |
|  | **L3-9-cis7-1** | **98%** |
|  | **L3-9-cis7-2** | **88%** |
|  | **L3-9-cis7-3** | **100%** |
|  | **L3-10-cis7and8-1** | **100%** |
| **L3-9-A** | **L3-9-1** | **68%** |
|  | **L3-9-cis6-1** | **100%** |
| L3-9-B | - | - |
| L3-9-C | - | - |
| **L3-10,11-A** | **L3-11-1** | **63%** |
| L3-10-A | - | - |
| L3-10-B | - | - |
| L3-10-C | - | - |
| L3-10-D | - | - |
| L3-11-A | - | - |
| L3-12-A | - | - |
| L3-12-B | - | - |
| L3-13-A | - | - |

Table S8: The comparison between North & Dunbrack *et al.* work and our clustering for CDR-L3. See description under Table S6

| Our cluster | North & Dunbrack *et al.* | Fraction of CDRs in  North & Dunbrack *et al.* cluster found in our cluster |
| --- | --- | --- |
| H1-4-A | H1-10-1 | 100% |
| H1-6-A | - | - |
| **H1-7-A** | **H1-13-1** | **98%** |
|  | **H1-13-2** | **57%** |
| **H1-7-B** | **-** | **-** |
| H1-7-C | H1-13-7 | 67% |
| H1-7-D | - | - |
| H1-7-E | H1-13-5 | 33% |
| H1-7-F | - | - |
| H1-7-G | H1-13-5 | 33% |
| **H1-8-A** | **H1-14-1** | **91%** |
| H1-8-B | - | - |
| H1-8-C | - | - |
| **H1-9-A** | **H1-15-1** | **78%** |
| H1-9-B | - | - |

Table S9: The comparison between North & Dunbrack *et al.* work and our clustering for CDR-H1. See description under Table S6

| Our cluster | North & Dunbrack *et al.* | Fraction of CDRs in  North & Dunbrack *et al.* cluster found in our cluster |
| --- | --- | --- |
| **H2-7-A** | **H2-9-1** | **96%** |
| H2-7-B | - | - |
| **H2-8-A** | **H2-10-1** | **95%** |
| **H2-8-B** | **H2-10-2** | **90%** |
| H2-8-C | - | - |
| **H2-8-D** | **H2-10-3** | **66%** |
| H2-8-E | - | - |
| H2-8-F | - | - |
| H2-8-G | H2-10-3 | 22% |
| H2-8-H | H2-10-6 | 50% |
| H2-8-I | - | - |
| H2-8-J | - | - |
| **H2-10-A** | **H2-12-1** | **100%** |

Table S10: The comparison between North & Dunbrack *et al.* work and our clustering for CDR-H2. See description under Table S6
