# Supplementary material for: Length-independent structural similarities enrich the antibody CDR canonical class model
Source: MAbs. 2016 Mar 10;8(4):751–60. doi: 10.1080/19420862.2016.1158370 (PMC4966832; doi:10.1080/19420862.2016.1158370)
Supplement: Supplemental_Datas.zip [file kmab-08-04-1158370-s001.zip › 2015MABS1071R-s05.docx]

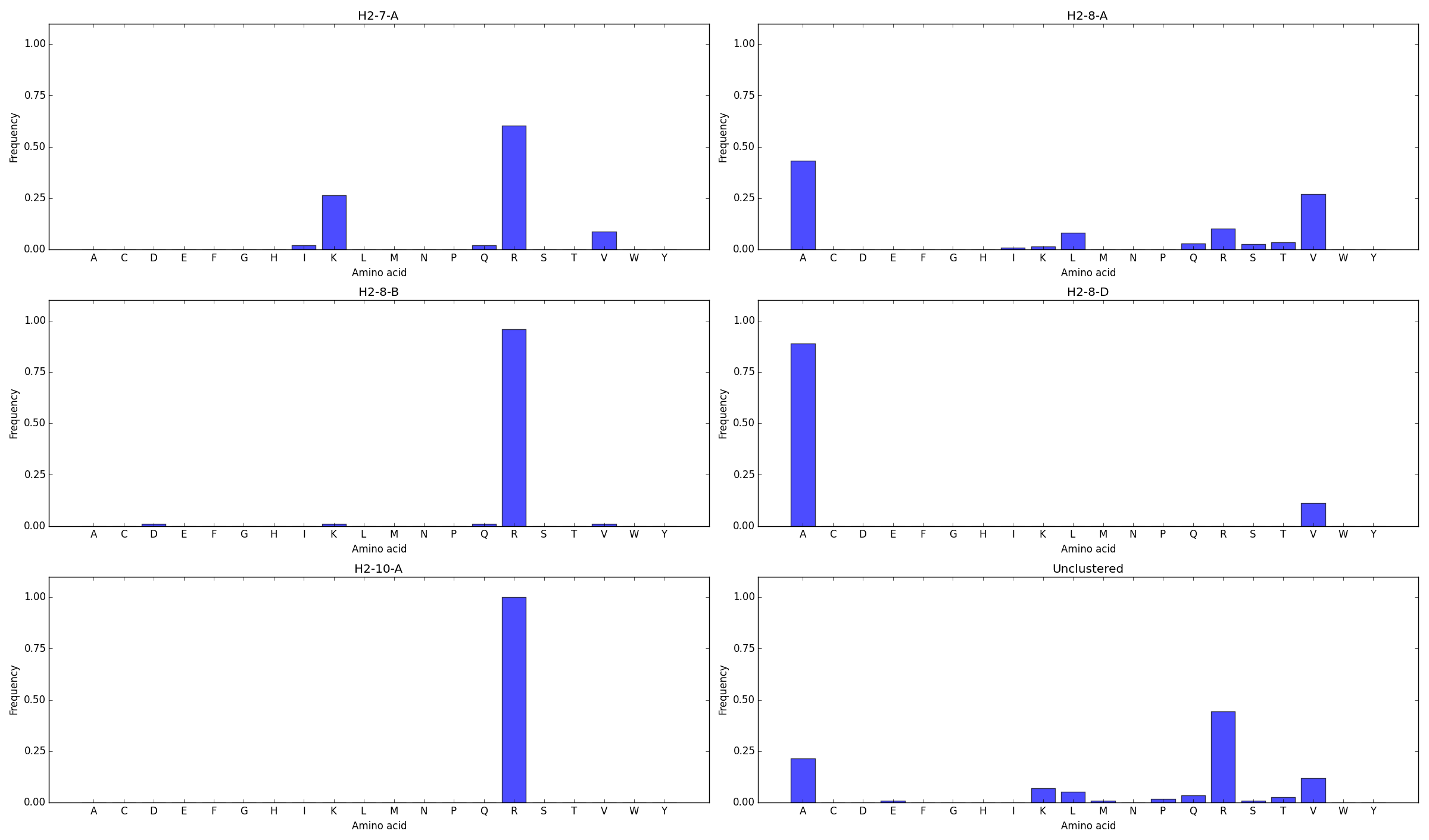


Figure S2: The amino acid distributions of the framework residue at Chothia position 71, plotted for each CDR-H2 cluster that contains at least six unique sequences. The y-axis in each panel shows the frequency of each amino acid type and the x-axis shows all standard amino acids. The “Unclustered” panel shows the distribution for all structures that are not contained within the large clusters.
