# Supplementary material for: Length-independent structural similarities enrich the antibody CDR canonical class model
Source: MAbs. 2016 Mar 10;8(4):751–60. doi: 10.1080/19420862.2016.1158370 (PMC4966832; doi:10.1080/19420862.2016.1158370)
Supplement: Supplemental_Datas.zip [file kmab-08-04-1158370-s001.zip › 2015MABS1071R-s06.docx]

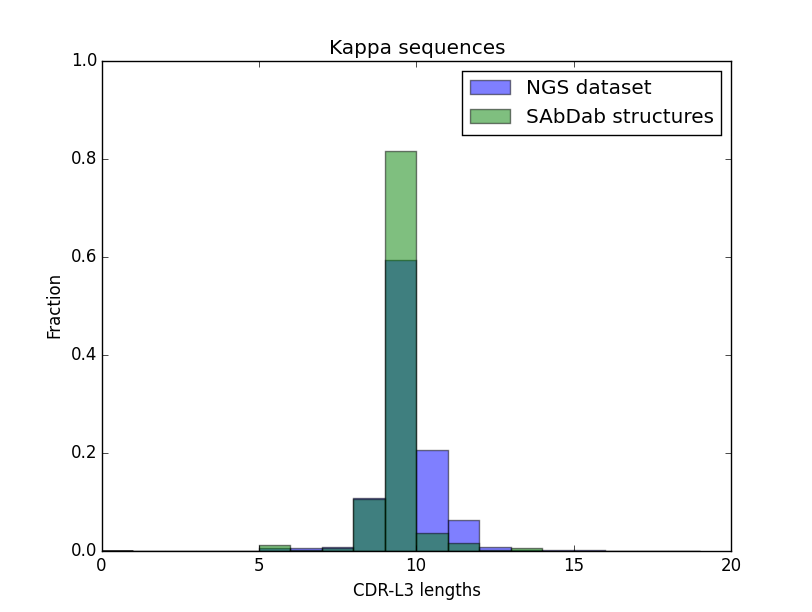


Figure S3: Comparison between the distribution of lengths of unique kappa CDR-L3 sequences in the UCB NGS dataset and in our structural data (Main text Section 2.2). The NGS distribution is shown in blue and the structural distribution is shown in green. The histograms have been normalized and the height of the bars shows the fraction of sequences that have a particular length. The graph shows that sequences of length nine are overrepresented and the sequences of length 10 are underrepresented in the structural dataset.


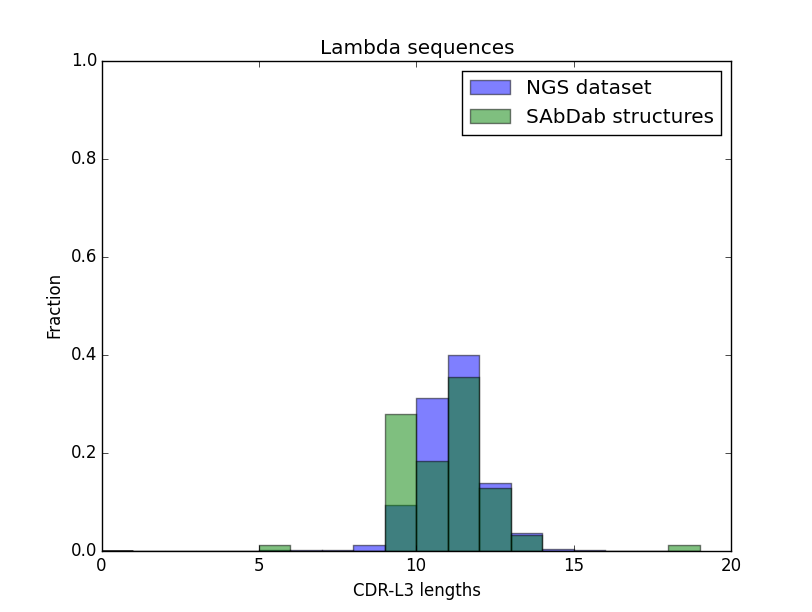


Figure S4: Comparison between the distribution of lengths of unique lambda CDR-L3 sequences in the UCB NGS dataset and the distribution in our structure data (Main text Section 2.2). The NGS distribution is shown in blue and the structural distribution is shown in green. The histograms have been normalized and the height of the bars shows the fraction of sequences that have a particular length. The graph shows that sequences of length nine are overrepresented and the sequences of length 10 are underrepresented in the structural dataset.


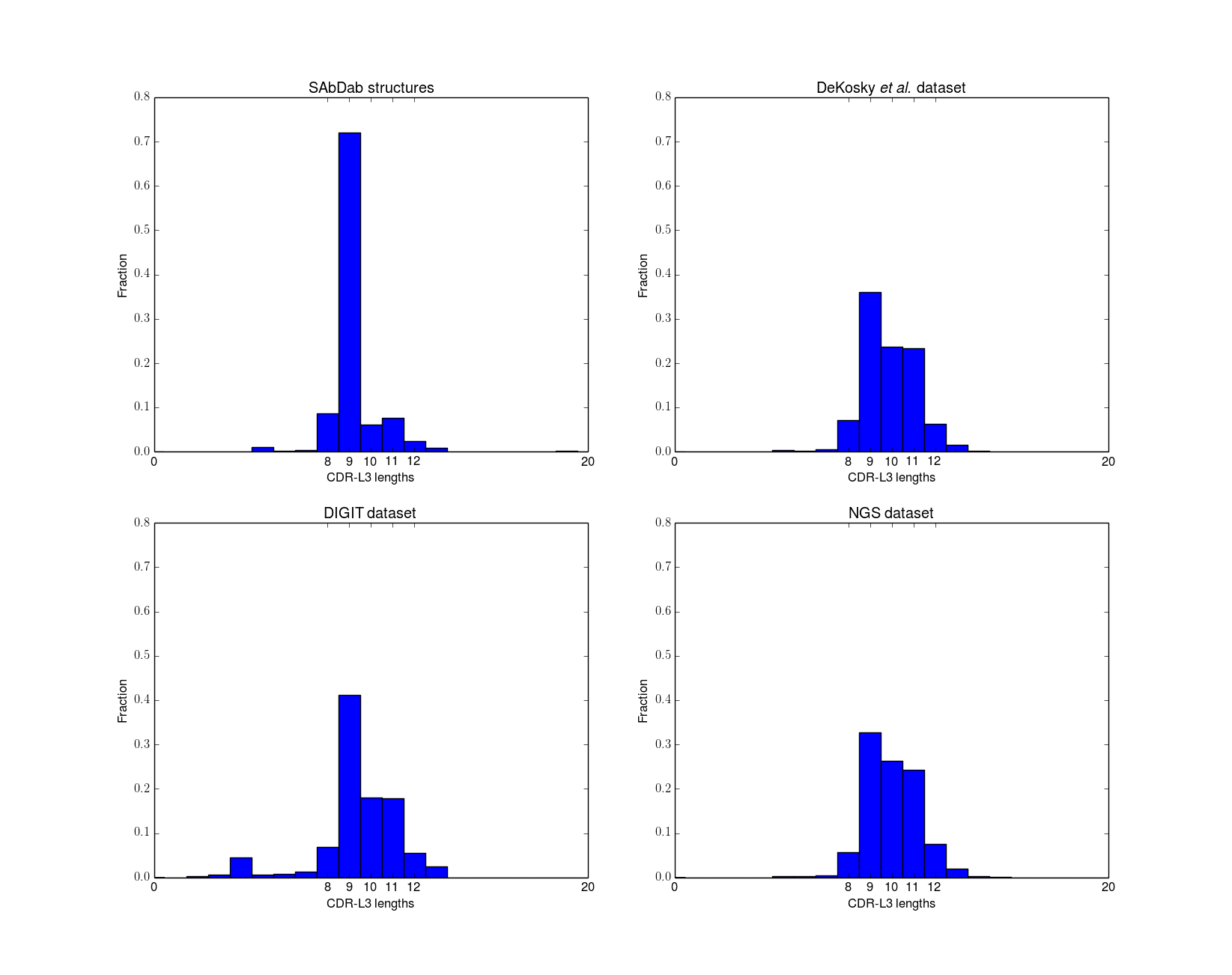
Figure S5: Comparison between the length distributions of the unique CDR-L3 sequences in the three NGS datasets and the distribution in our structure data (Main text Section 2.2). The top left panel shows the distribution in our structural dataset, the top right panel shows the distribution in the DeKosky *et al.* (DeKosky et al., 2014) dataset, the bottom left panel shows the distribution for the DIGIT (Chailyan, Tramontano, & Marcatili, 2012) dataset and the bottom right panel shows the distribution in the UCB NGS dataset. The histograms have been normalized and the height of the bars shows the fraction of sequences with a particular length. The distributions of lengths in the UCB NGS and the DeKosky *et al.* datasets are similar, which is expected as they both contain only human sequences. The structural dataset contains mostly sequences of length nine, other lengths are significantly underrepresented.
