# Supplementary material for: Length-independent structural similarities enrich the antibody CDR canonical class model
Source: MAbs. 2016 Mar 10;8(4):751–60. doi: 10.1080/19420862.2016.1158370 (PMC4966832; doi:10.1080/19420862.2016.1158370)
Supplement: Supplemental_Datas.zip [file kmab-08-04-1158370-s001.zip › 2015MABS1071R-s07.docx]

ROC curves


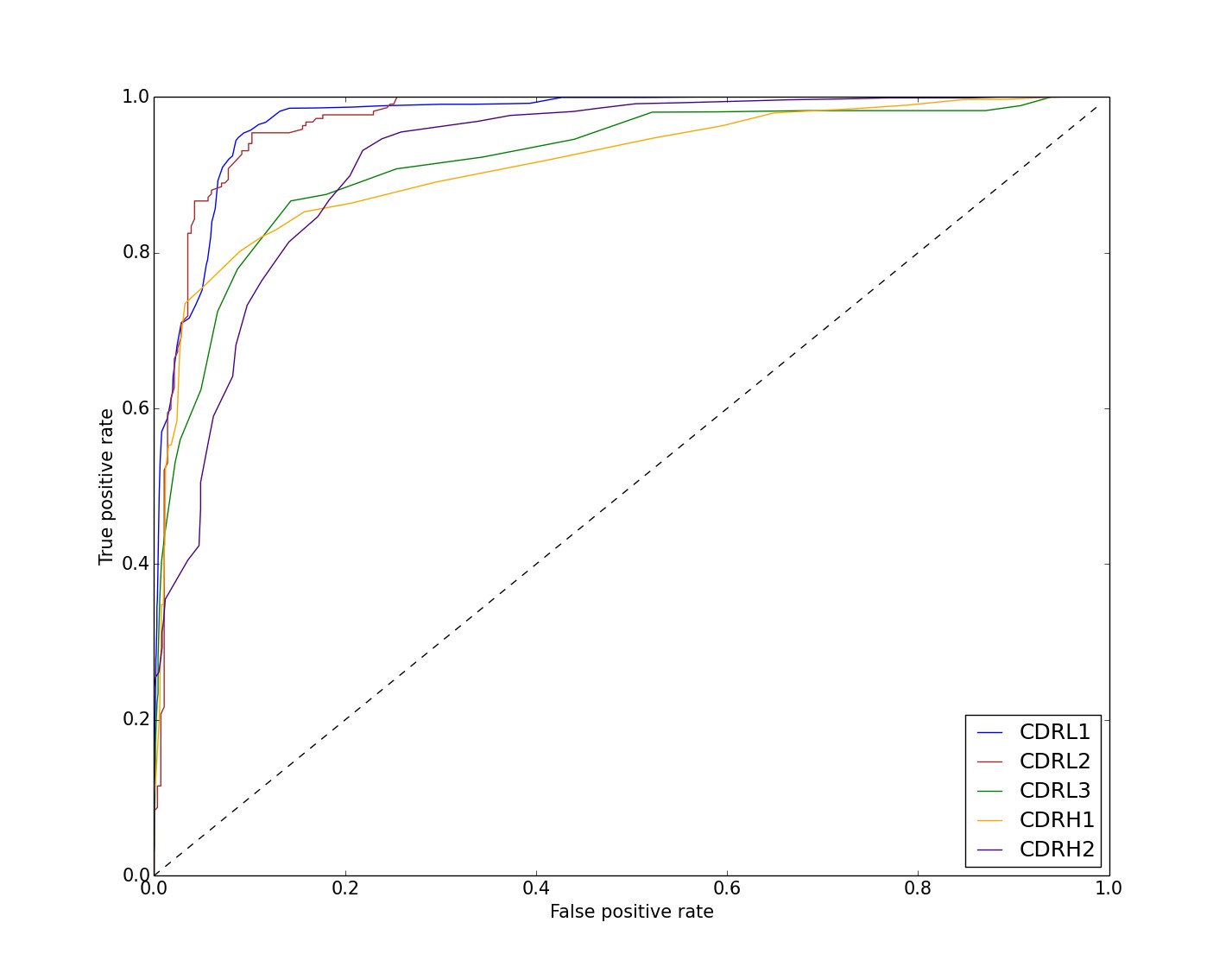


Figure S6: Receiver Operating Characteristic (ROC) curves for length-independent clustering for all CDR types, obtained by macro-averaging the results for each constituent cluster for each CDR type. The ROC curve for CDRL1 is shown in blue, for CDRL2 in brown, for CDRL3 in green, for CDRH1 in yellow and for CDRH2 in violet. The Area Under the Curve (AUC) for CDRL1 is 0.97, for CDRL2 is 0.97, for CDRL3 is 0.92, for CDRH1 is 0.91 and for CDRH2 is 0.92. A perfect model would get an AUC score of 1.0 while a random predictor would receive a score of 0.5.


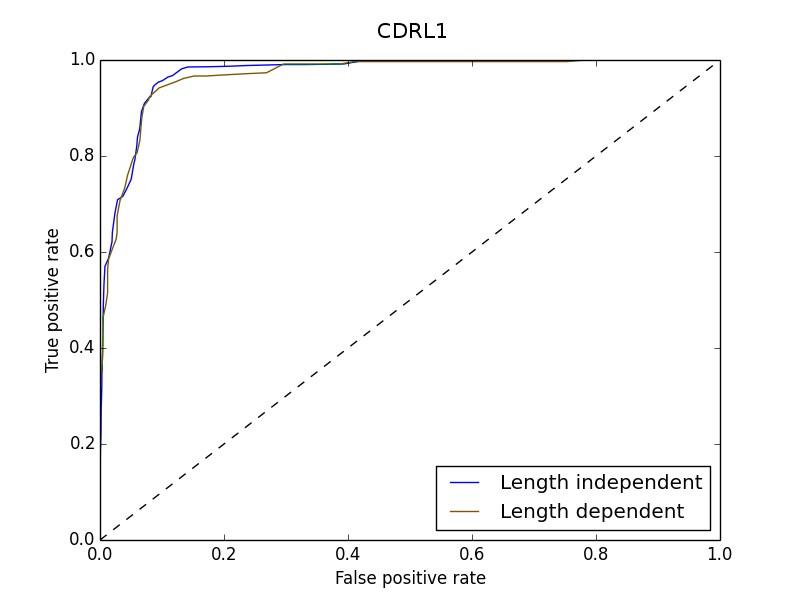


Figure S7: Comparison between ROCs plotted for length-independent and length-dependent clustering of CDR-L1 loops, obtained by macro-averaging the results for each constituent cluster. The length independent curve is shown in blue and the length dependent one in brown. The AUC for both curves is 0.97. The difference between the curves is not statistically significant with a p-value of 0.48 (see Section 4.4).


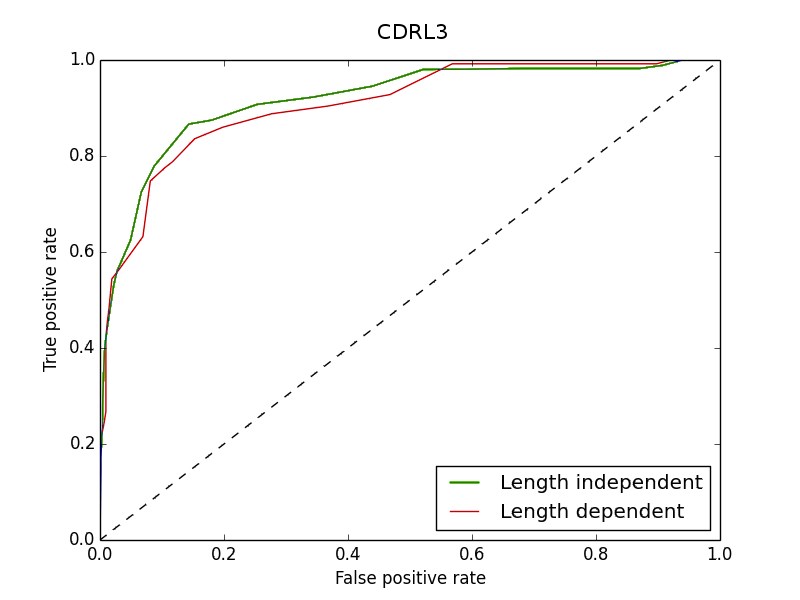


Figure S8: Comparison between ROCs plotted for length-independent and length-dependent clustering of CDR-L3 loops, obtained by macro-averaging the results for each constituent cluster. The length independent curve is shown in green and the length dependent one in red. The AUC for the length-independent curve is 0.92 while the AUC for length-dependent curve is 0.91. The difference between the curves is not statistically significant with a p-value of 0.07 (see Section 4.4).
