# Supplementary figures and images for: Length-independent structural similarities enrich the antibody CDR canonical class model
Source: MAbs. 2016 Mar 10;8(4):751–60. doi: 10.1080/19420862.2016.1158370 (PMC4966832; doi:10.1080/19420862.2016.1158370)

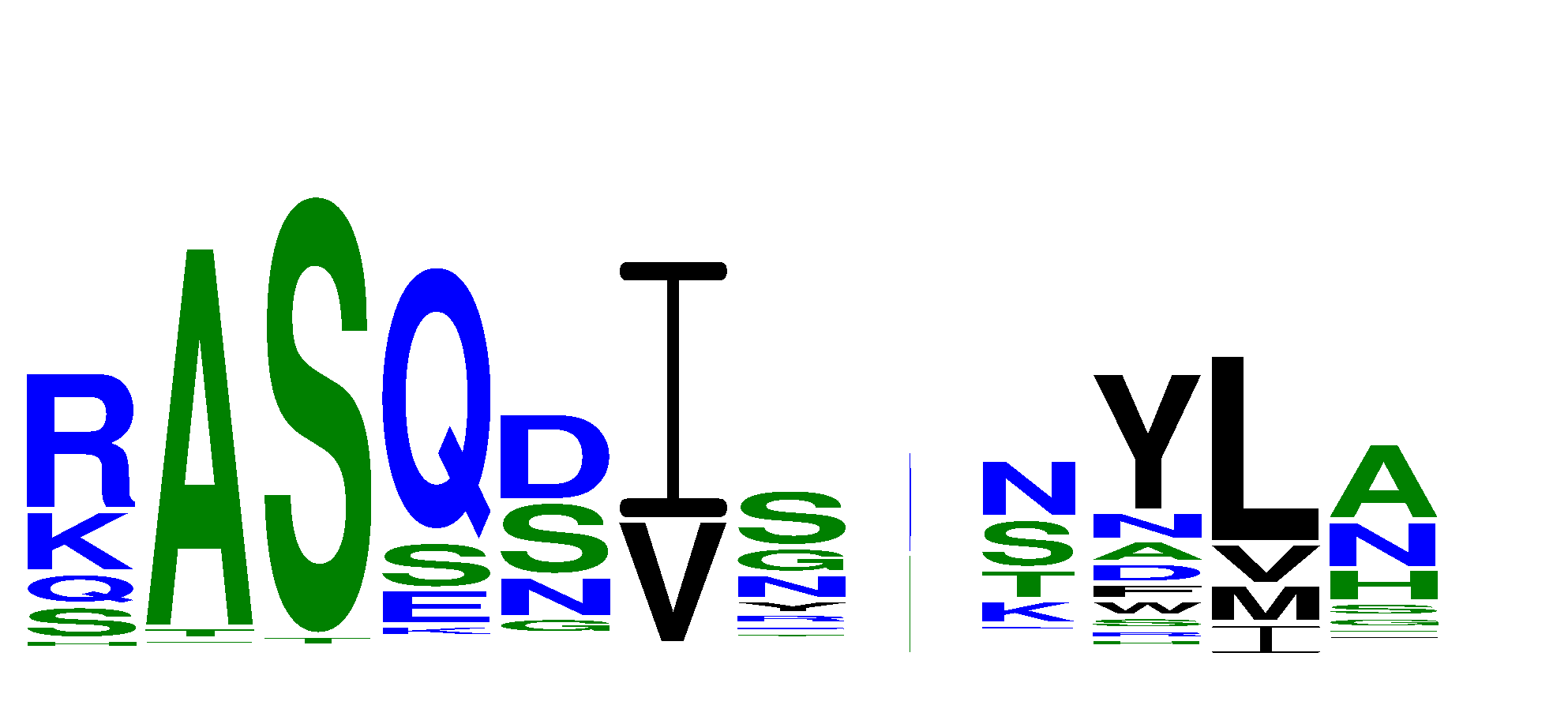


L1-10,11,12-A


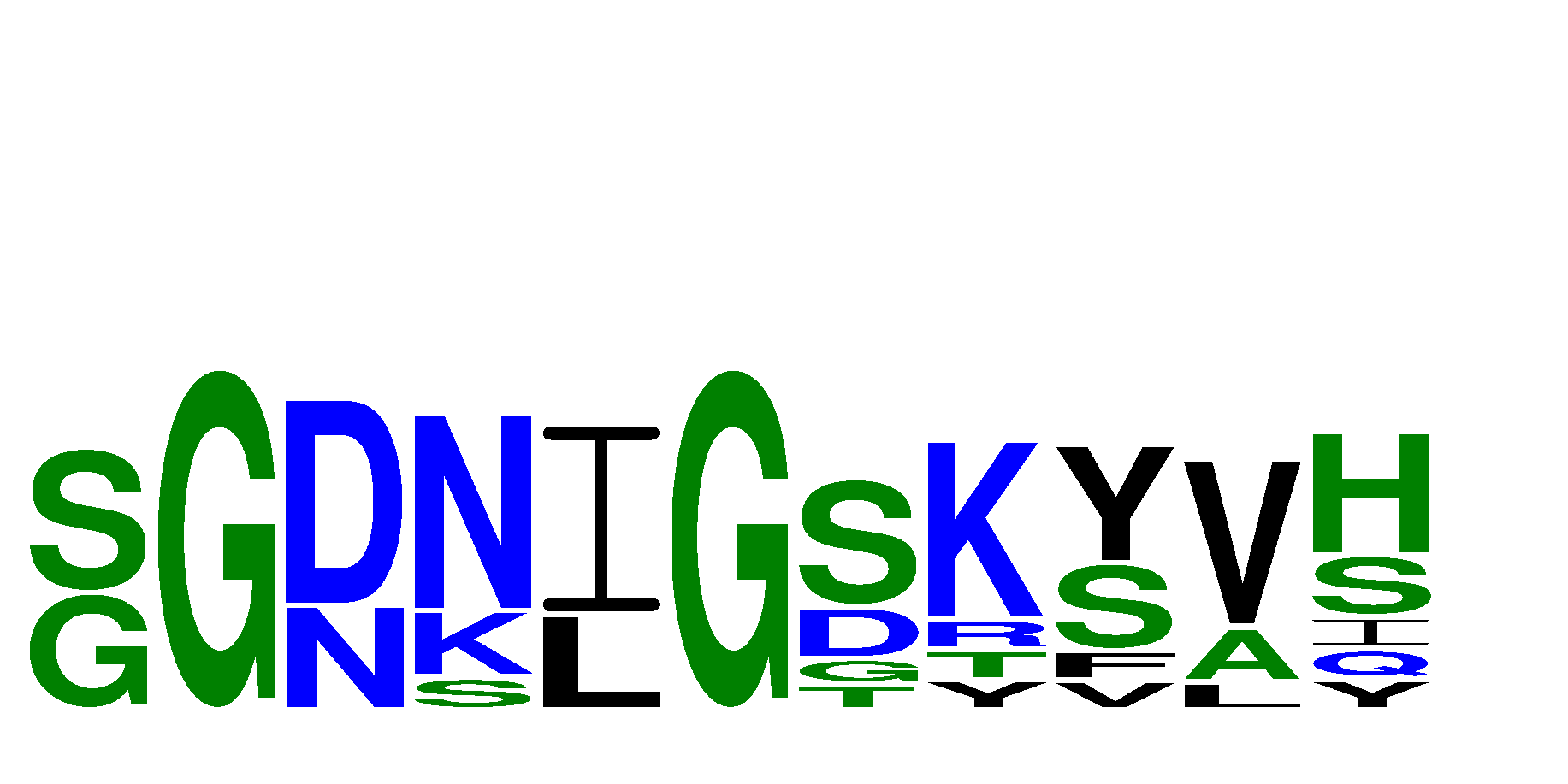


L1-11-A


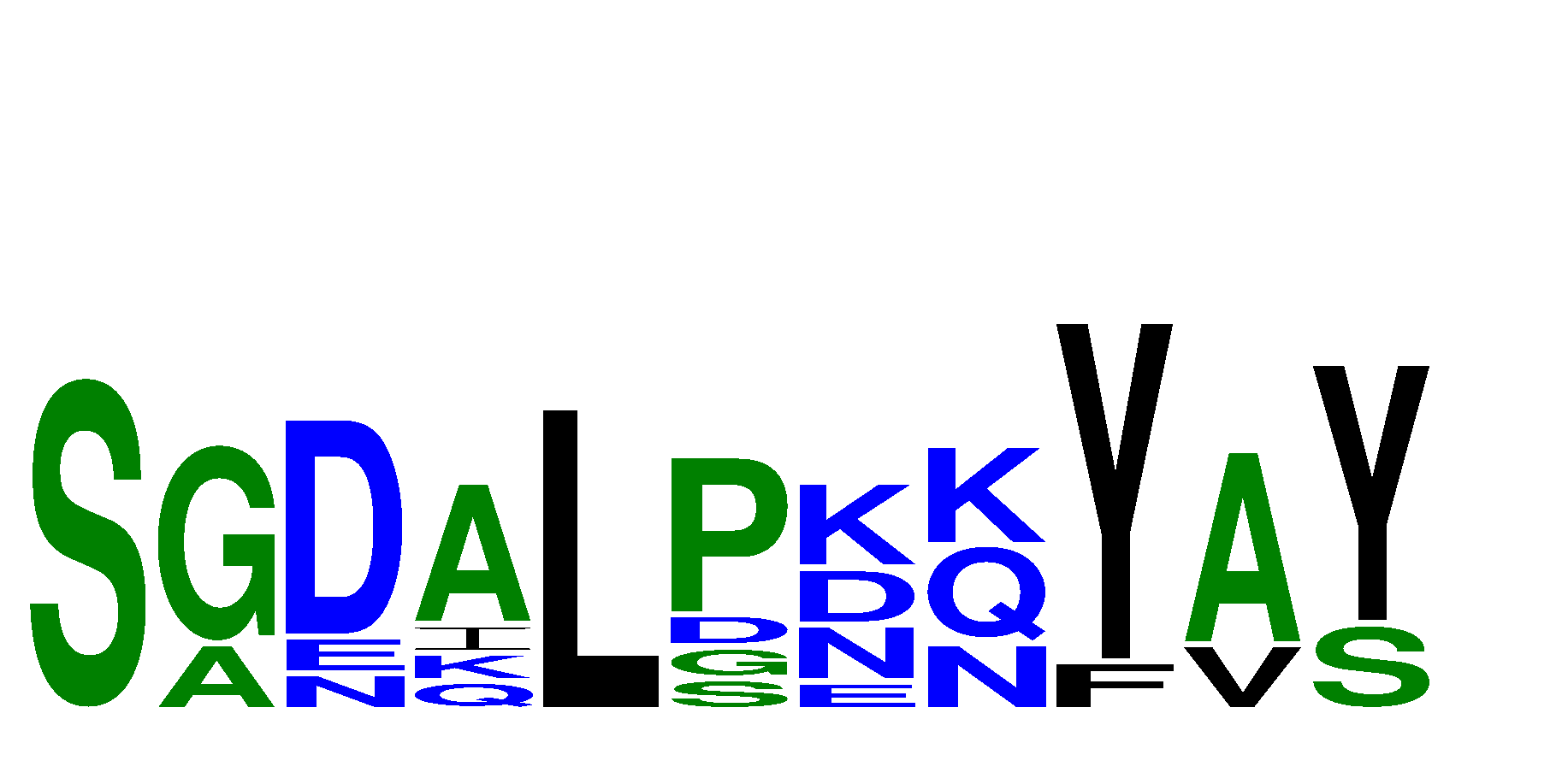


L1-11-B


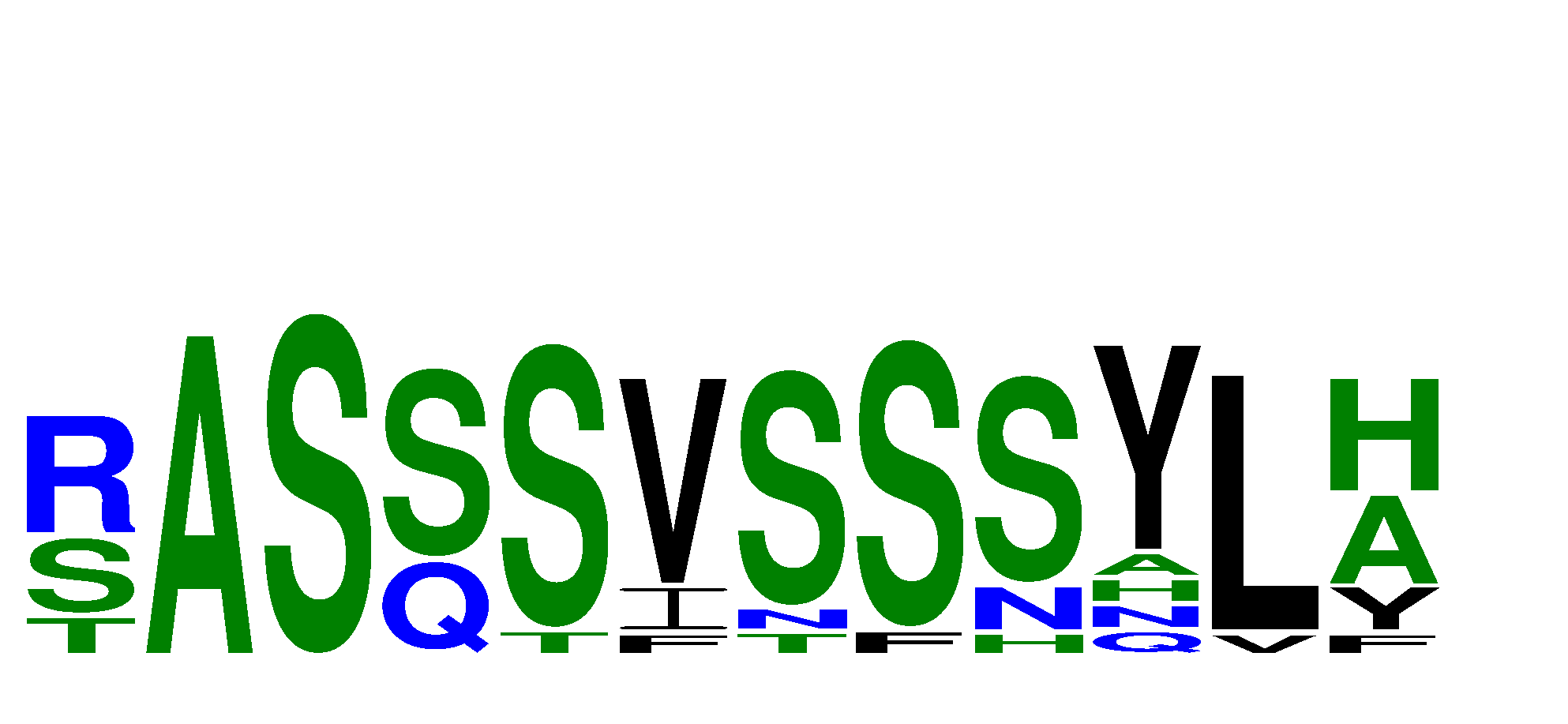


L1-12-A


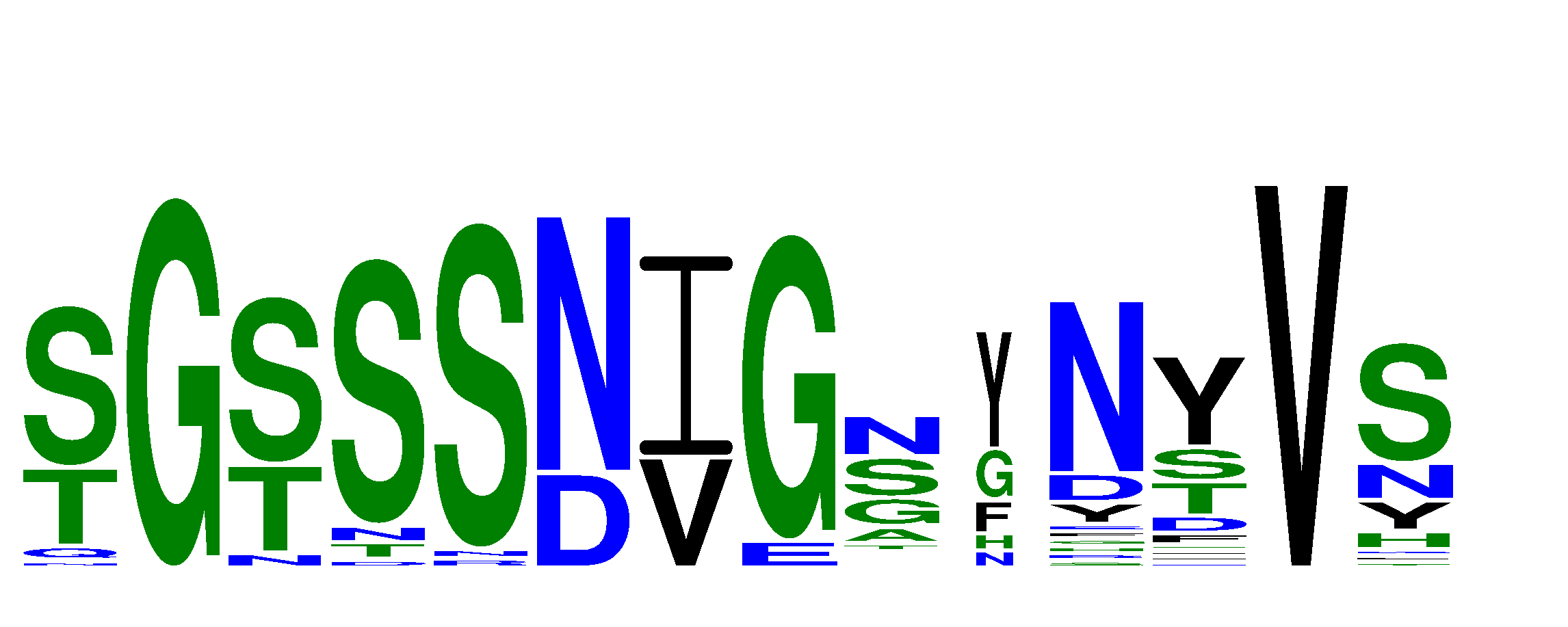


L1-13,14-A


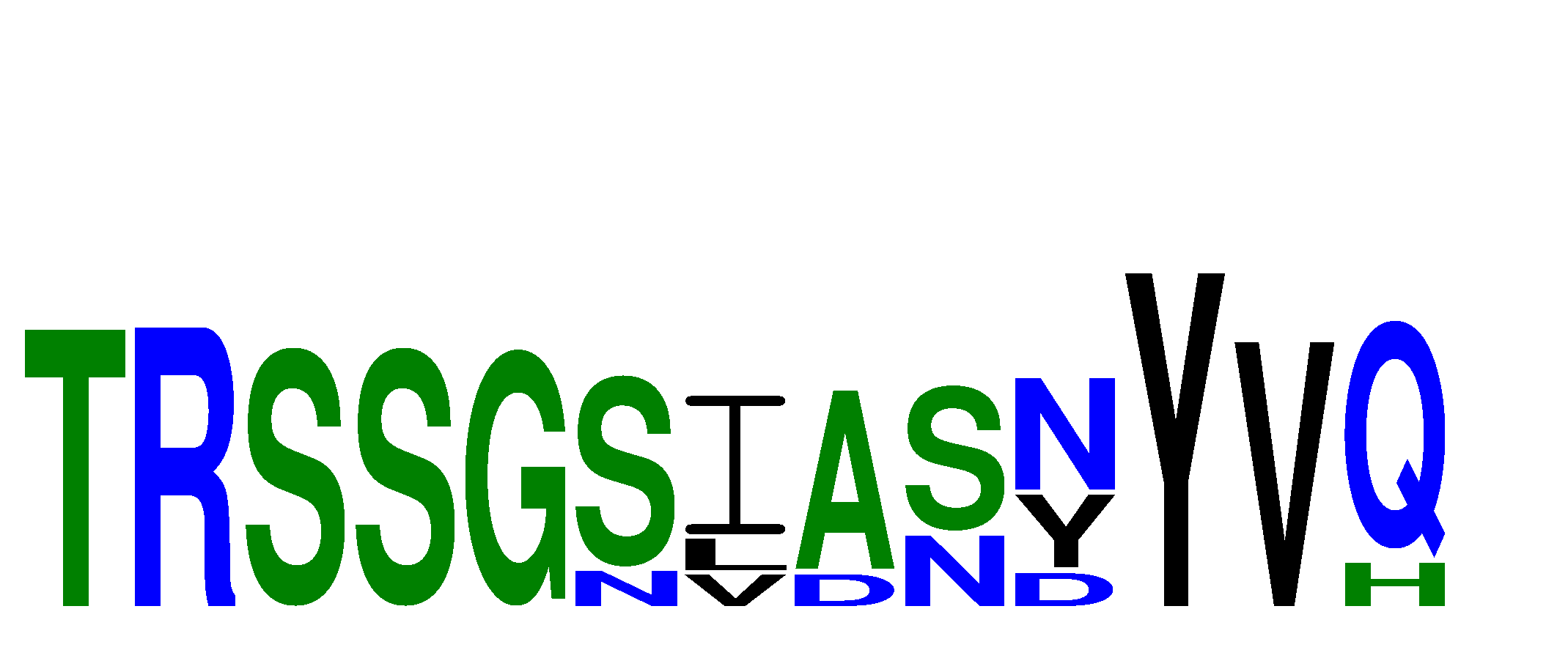


L1-13-A
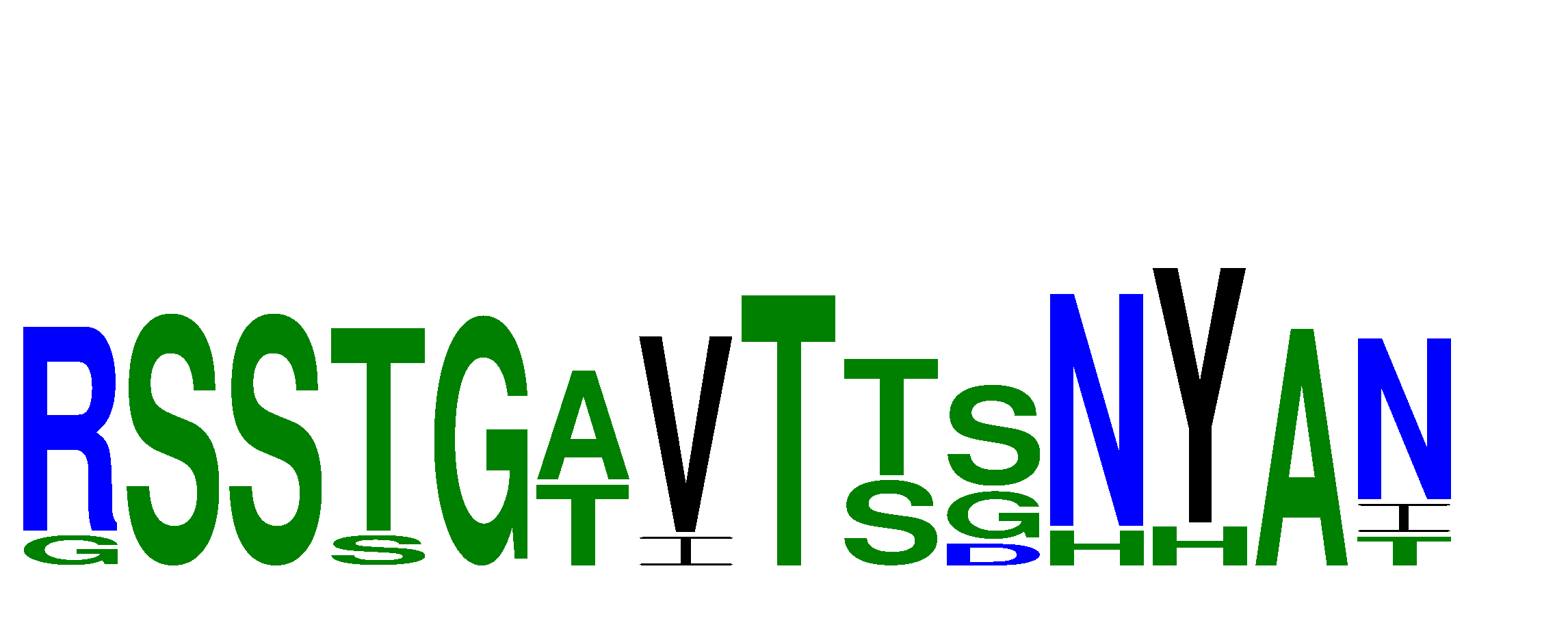


L1-14-A
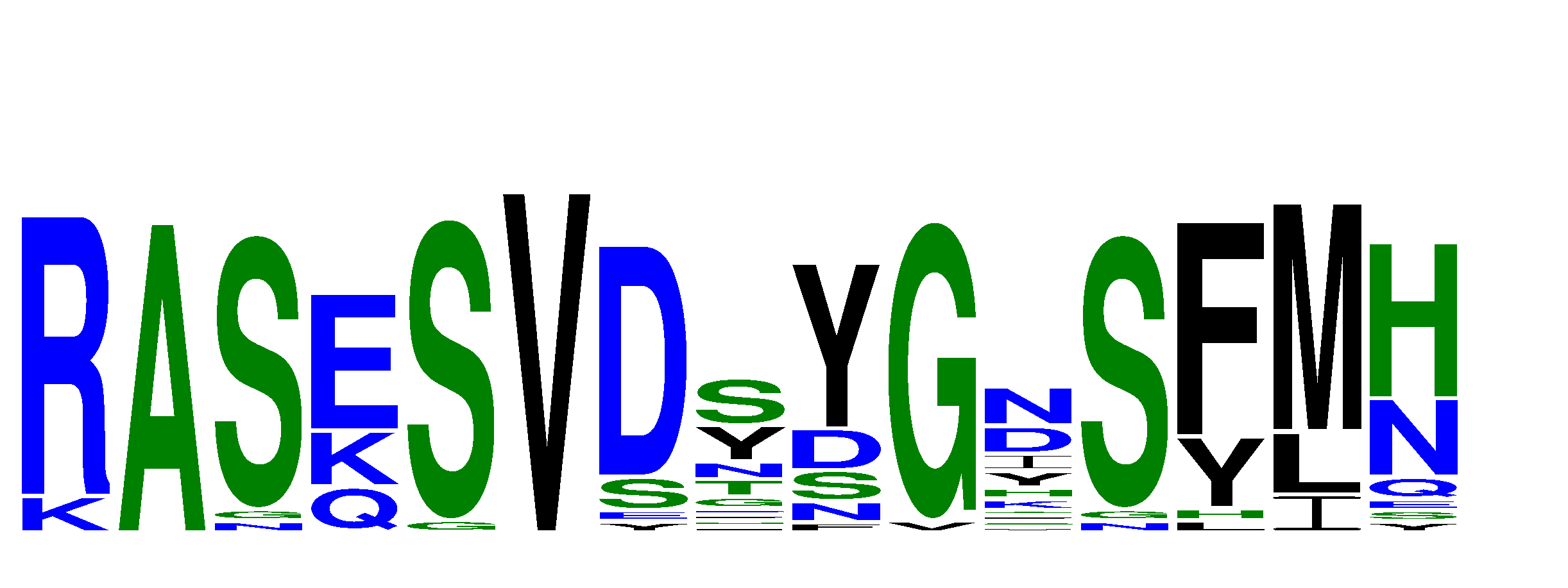


L1-15-A


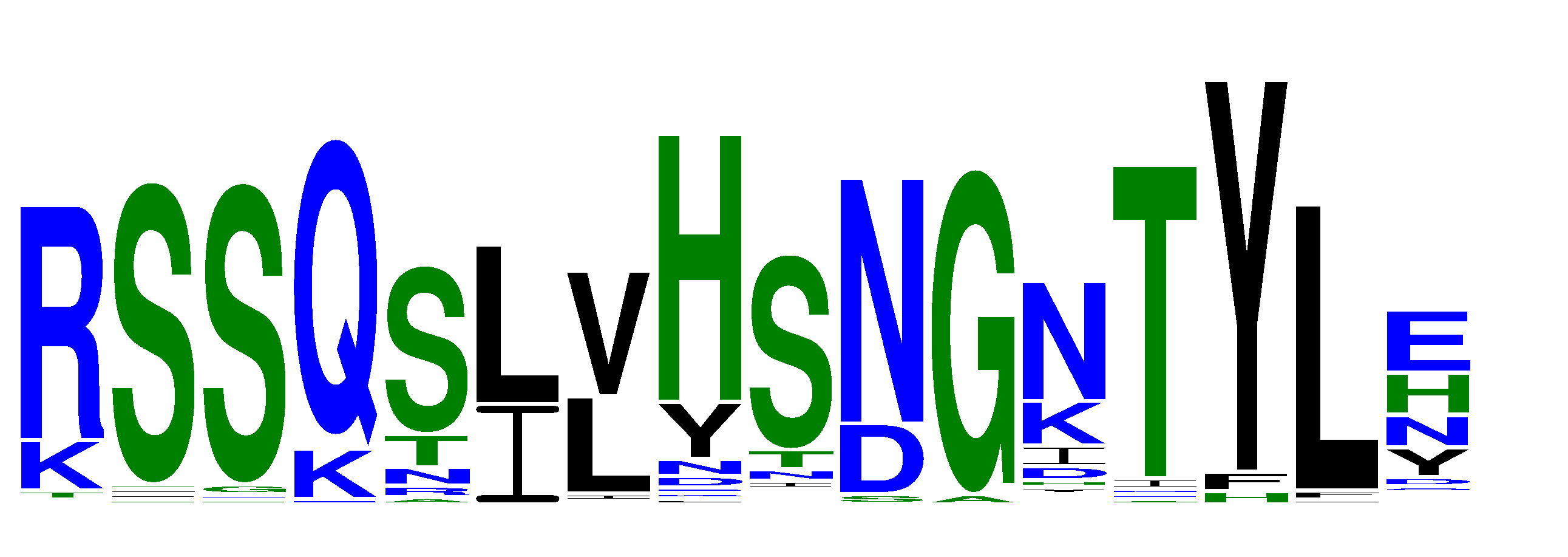


L1-16-A


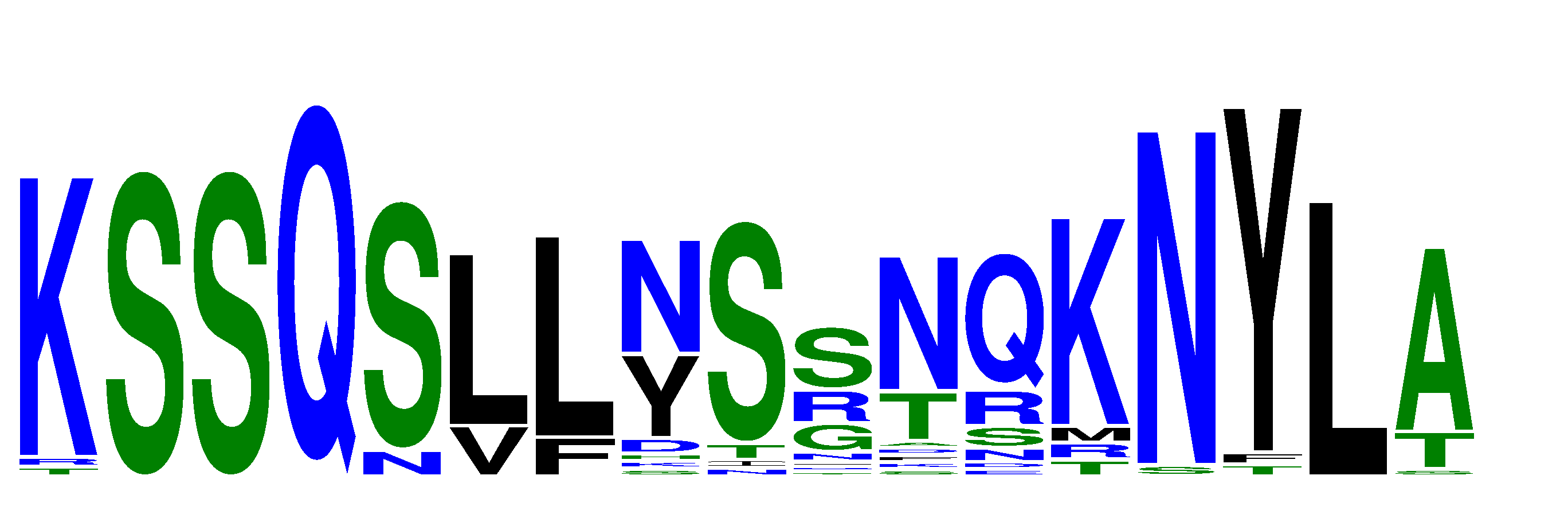


L1-17-A

Supplement: Supplemental_Datas.zip [file kmab-08-04-1158370-s001.zip › 2015MABS1071R-s08.docx]

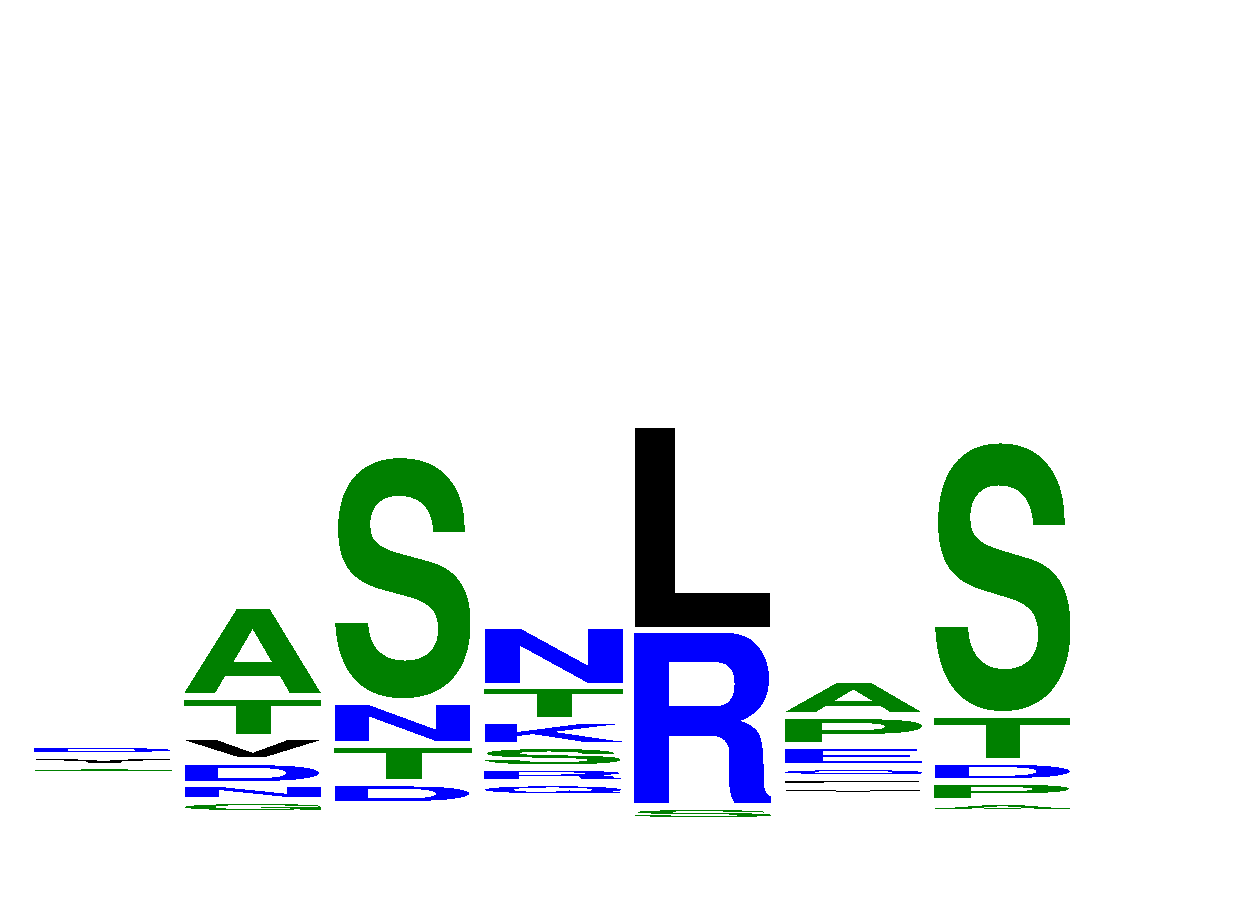


L2-7-A


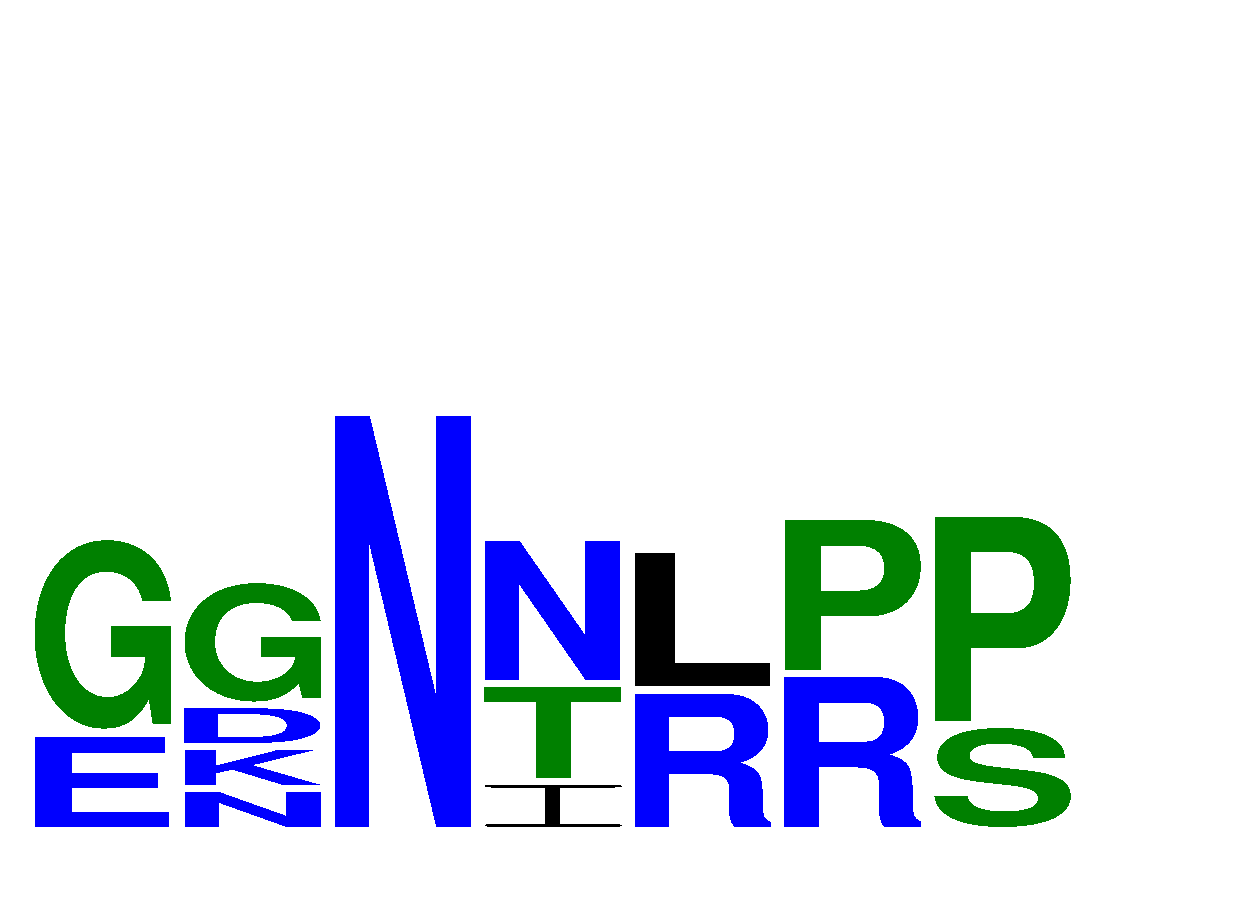


L2-7-B

Supplement: Supplemental_Datas.zip [file kmab-08-04-1158370-s001.zip › 2015MABS1071R-s09.docx]

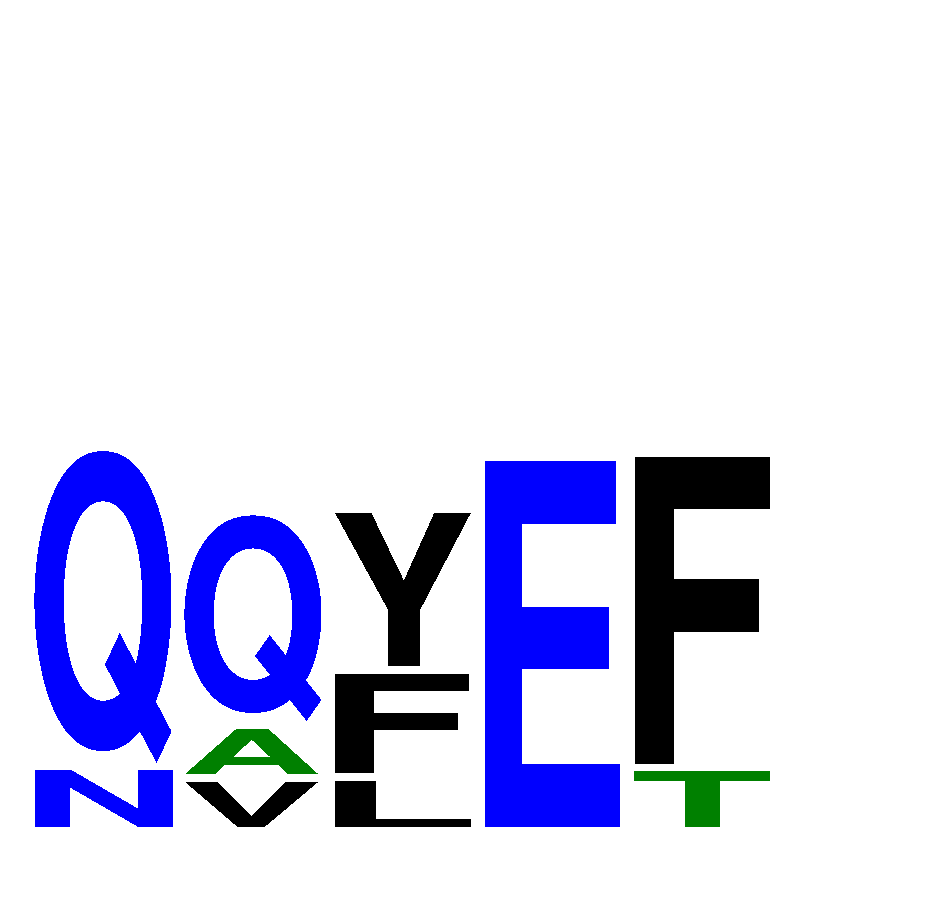


L3-5-A


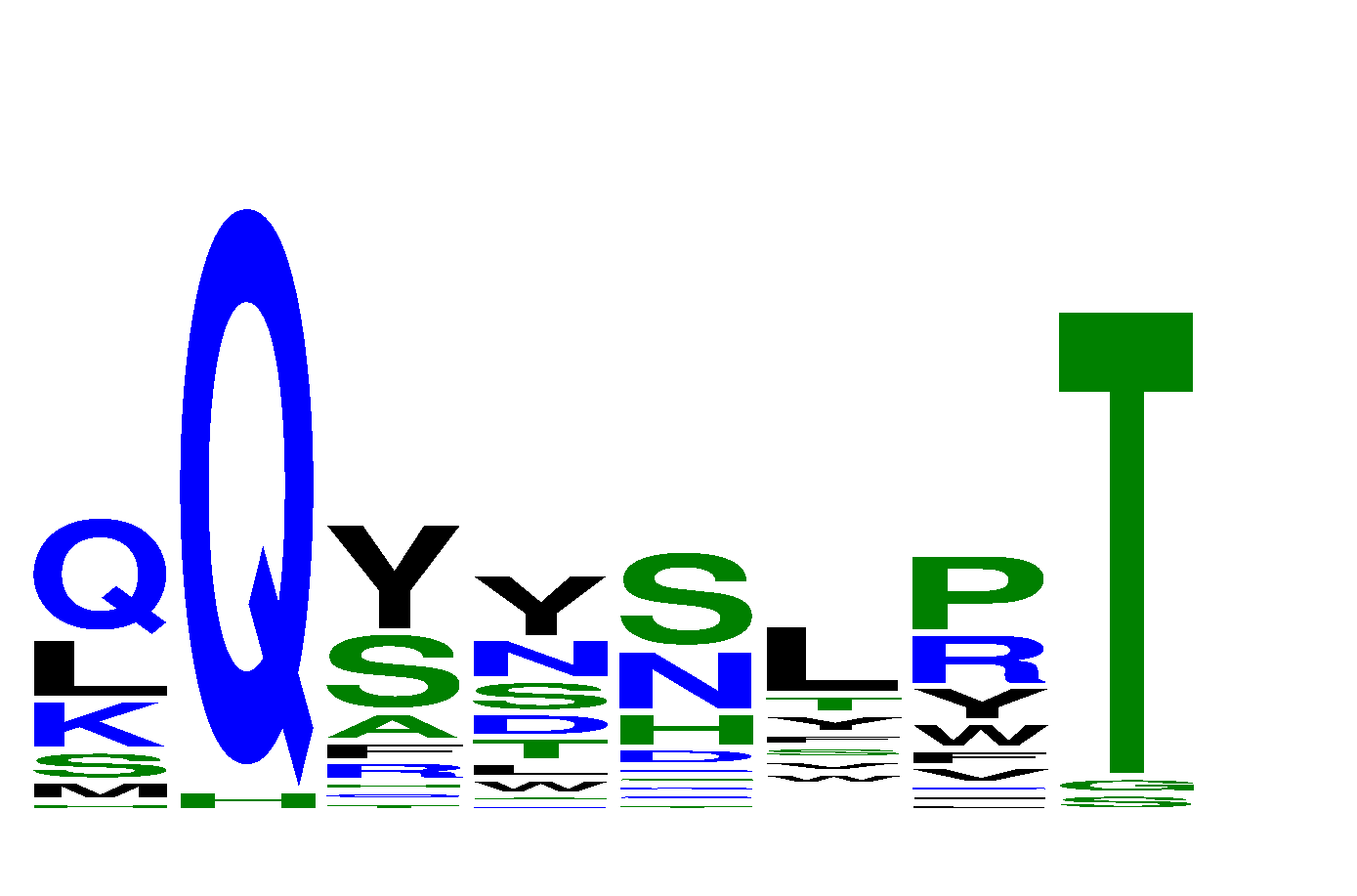


L3-8-A


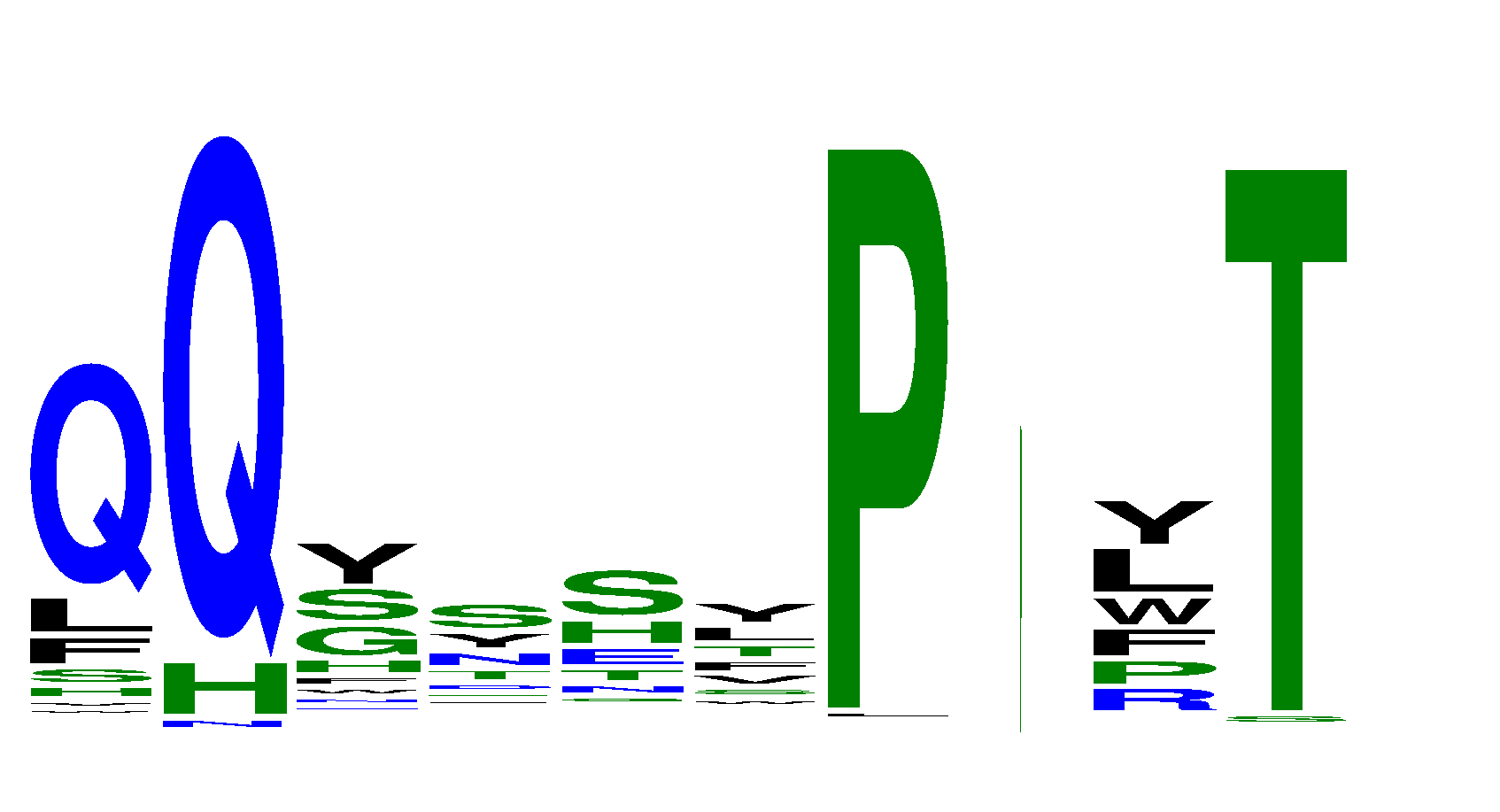


L3-9,10-A


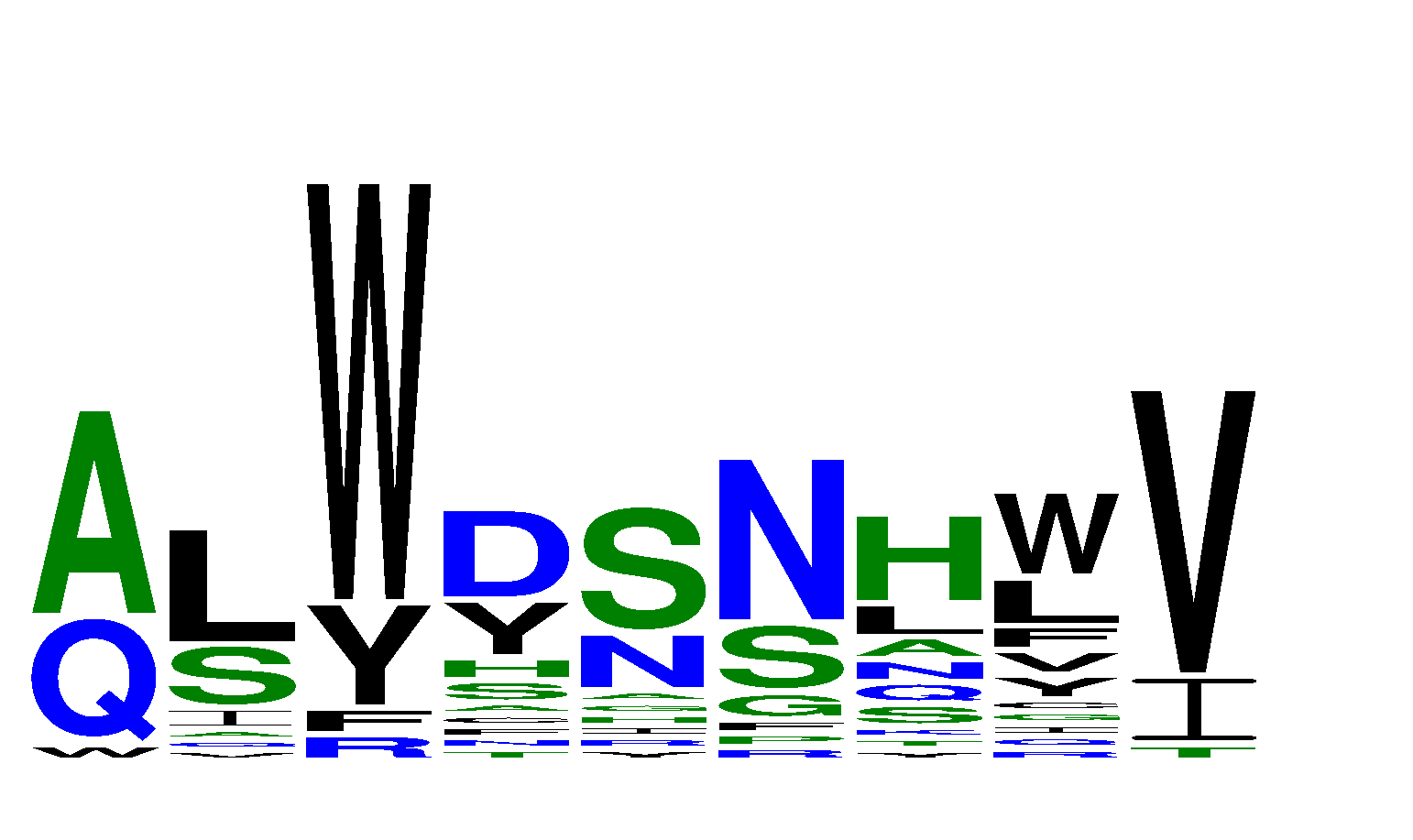


L3-9-A


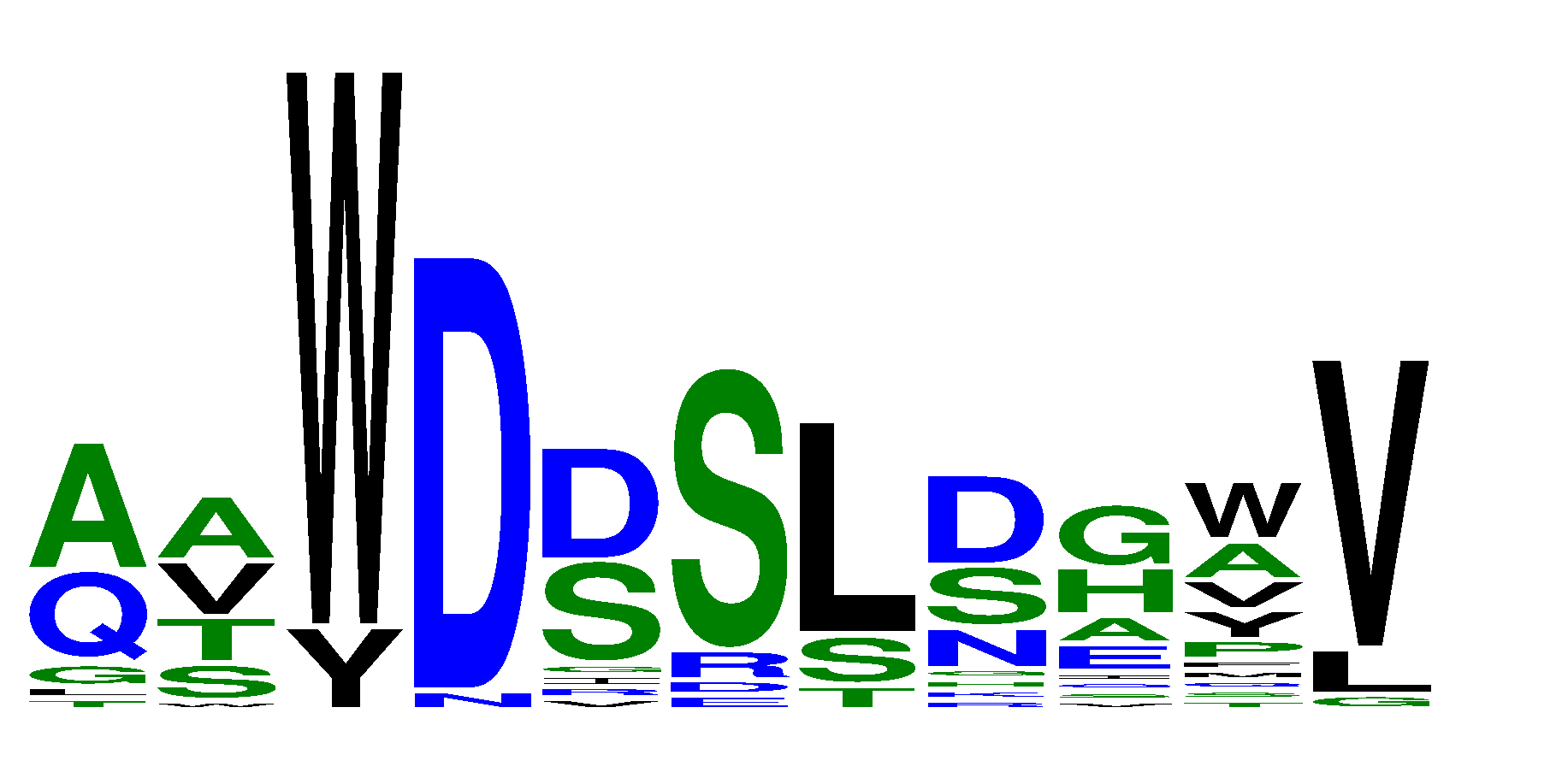


L3-10,11-A

Supplement: Supplemental_Datas.zip [file kmab-08-04-1158370-s001.zip › 2015MABS1071R-s10.docx]

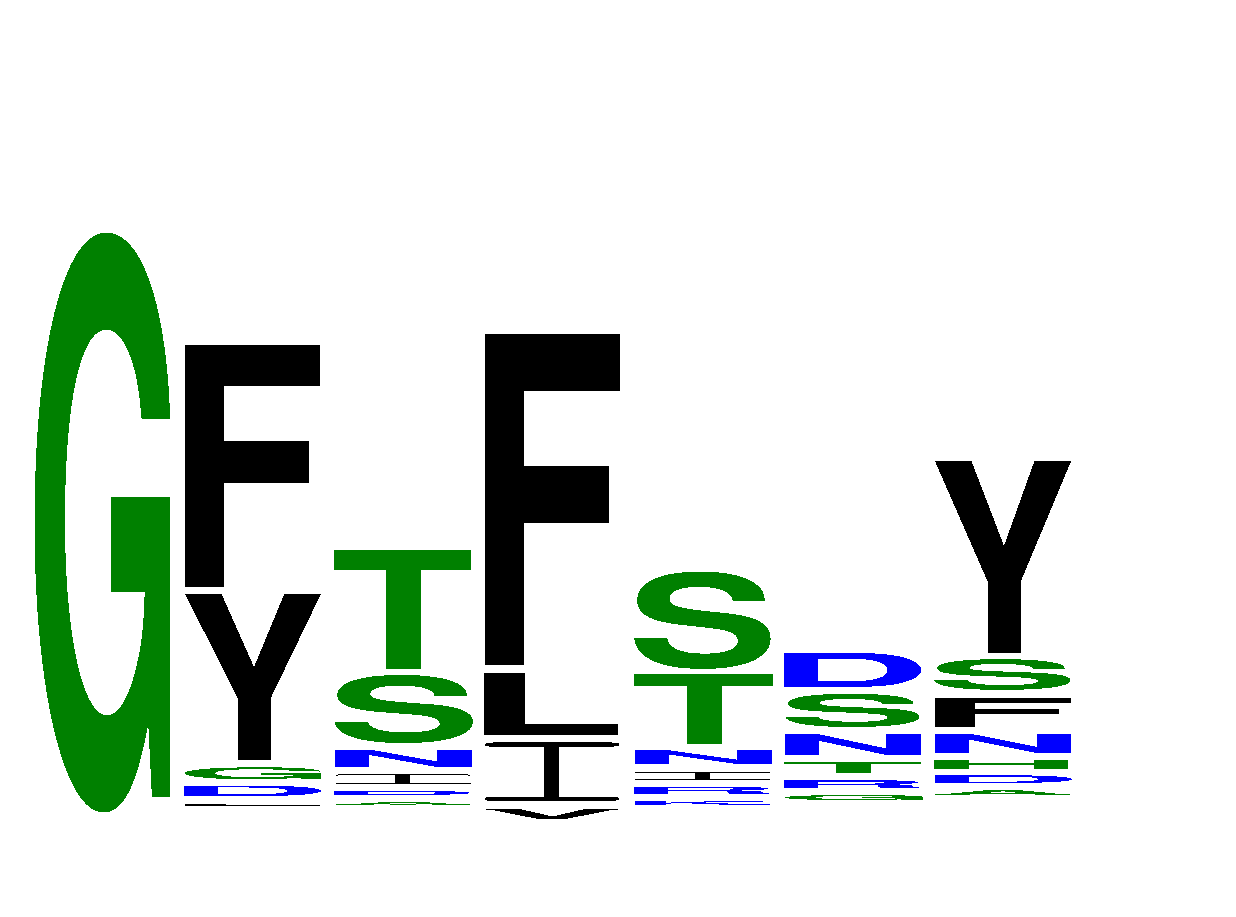


H1-7-A


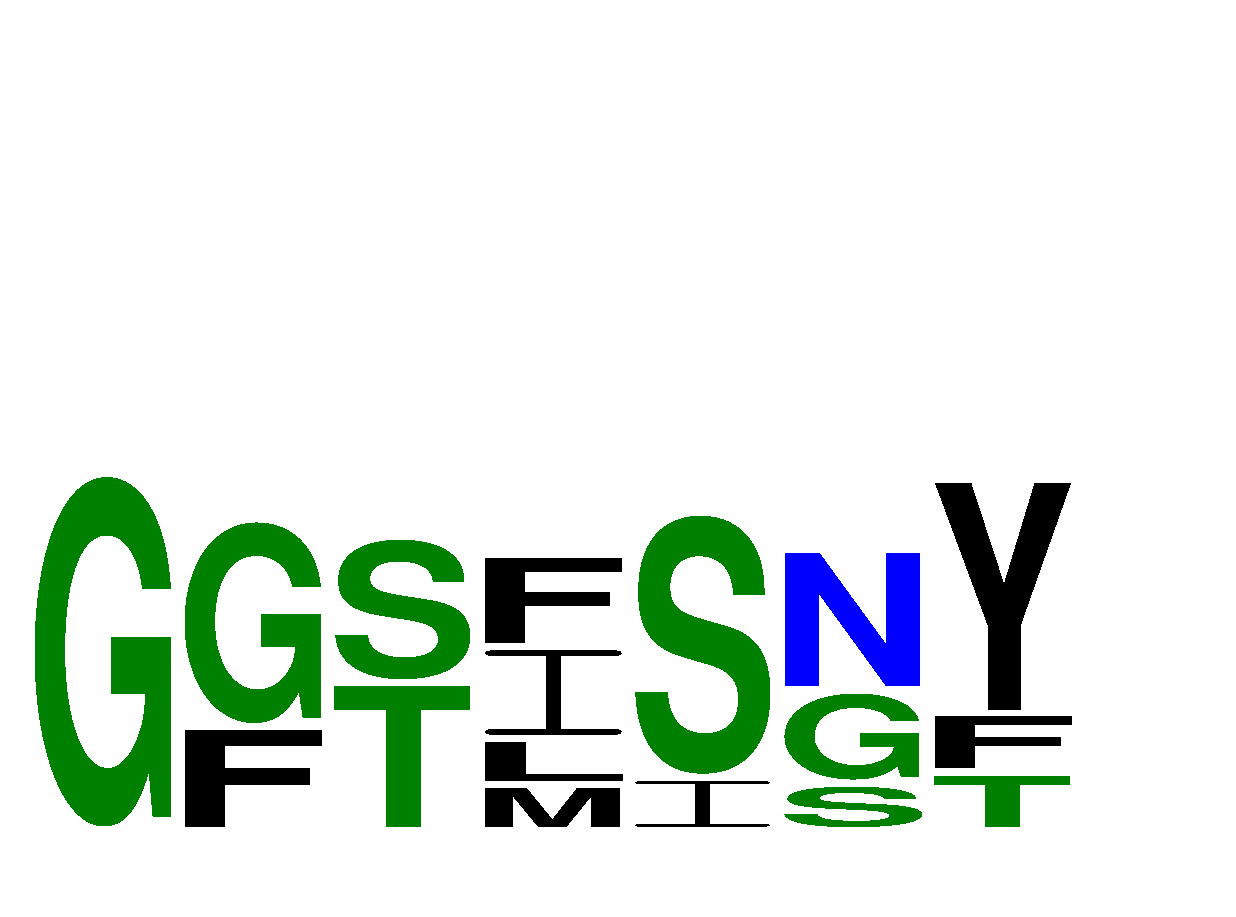


H1-7-B


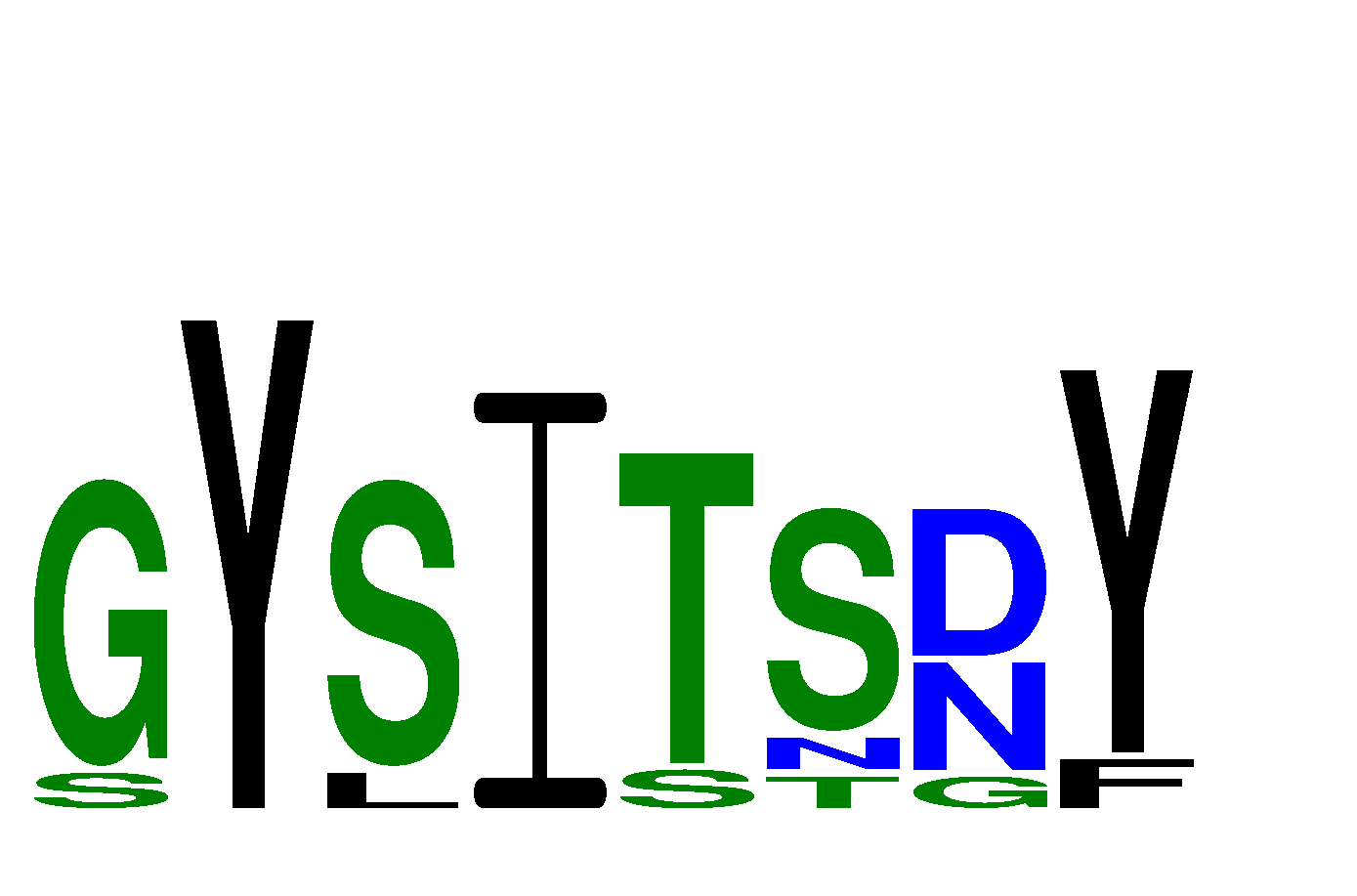


H1-8-A


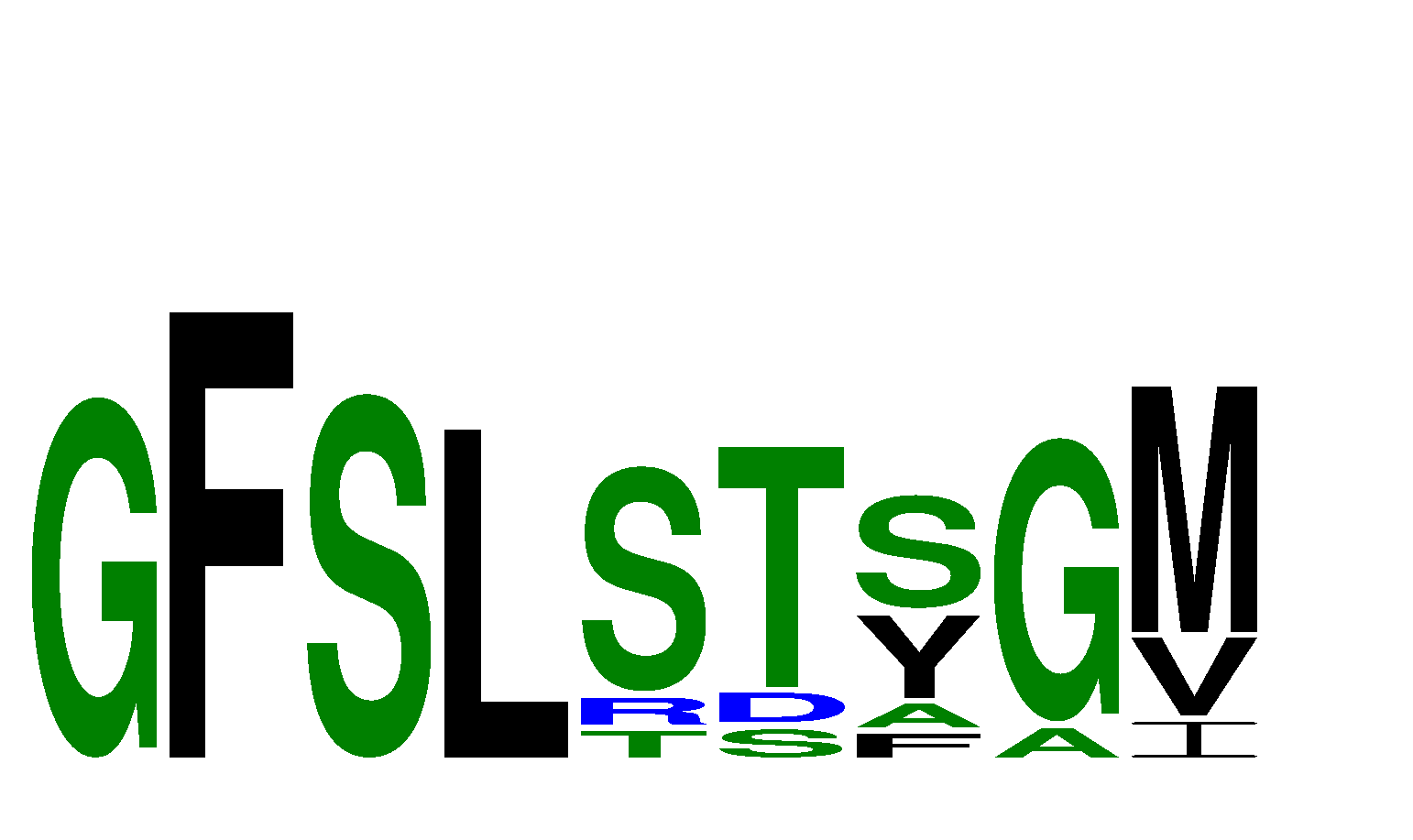


H1-9-A

Supplement: Supplemental_Datas.zip [file kmab-08-04-1158370-s001.zip › 2015MABS1071R-s11.docx]

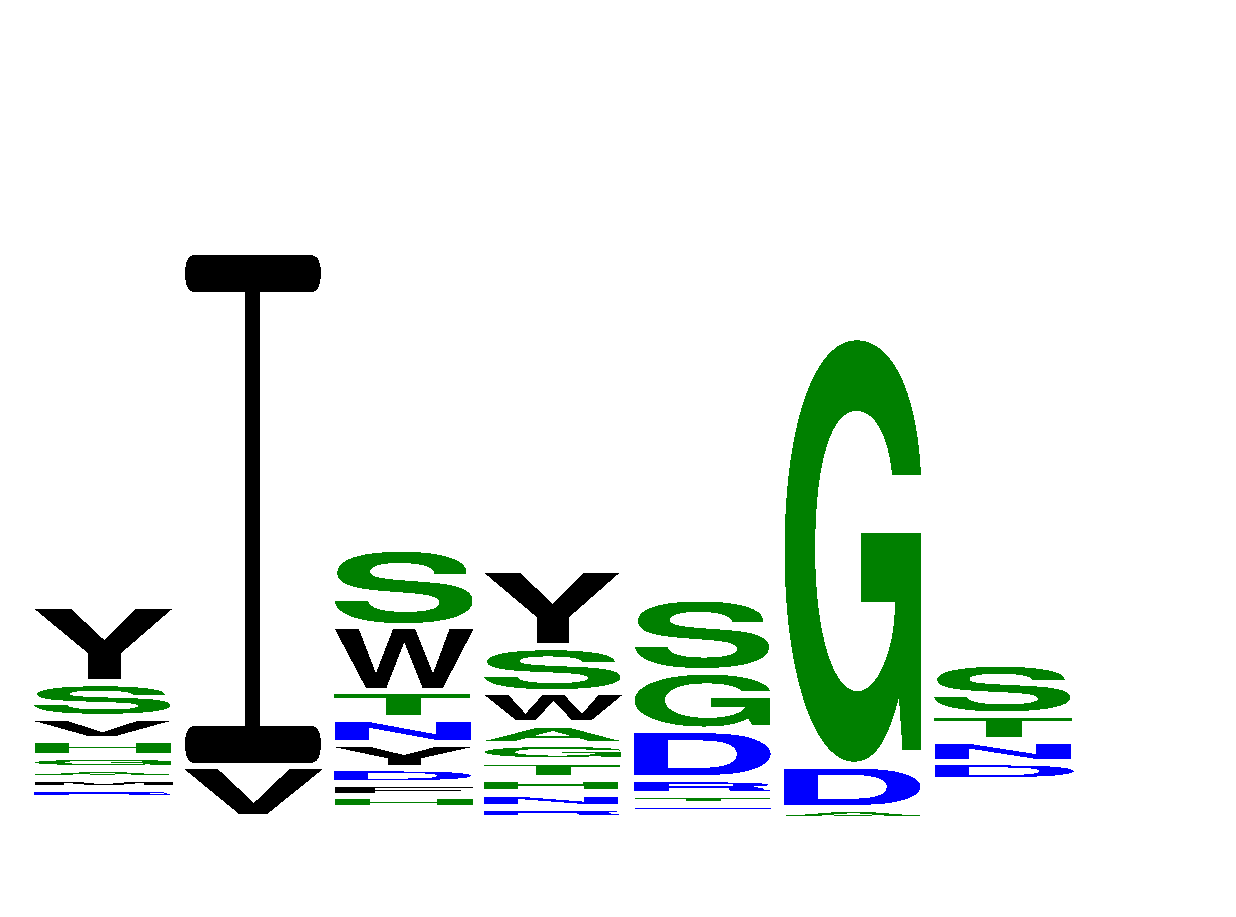


H2-7-A


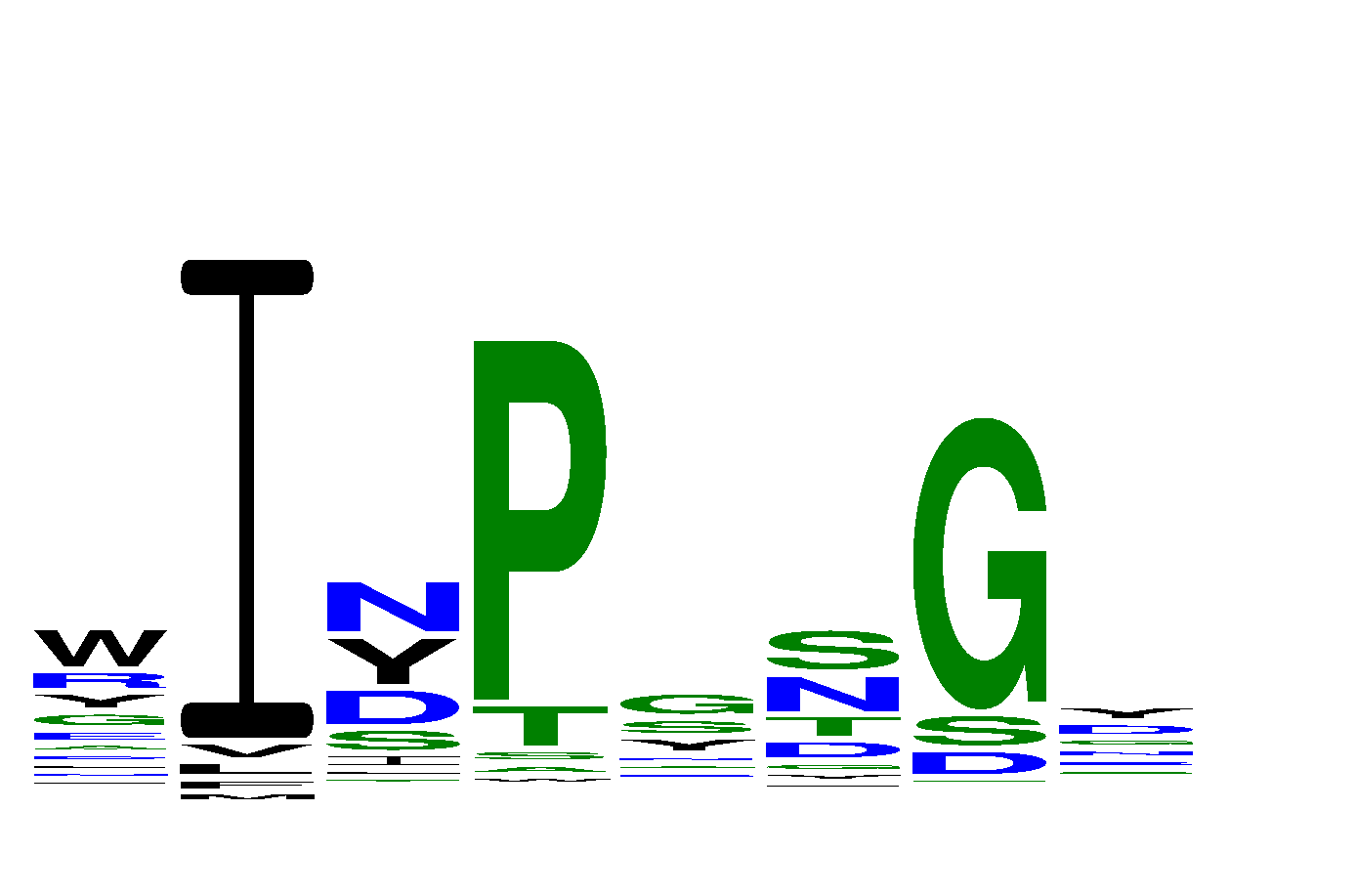


H2-8-A


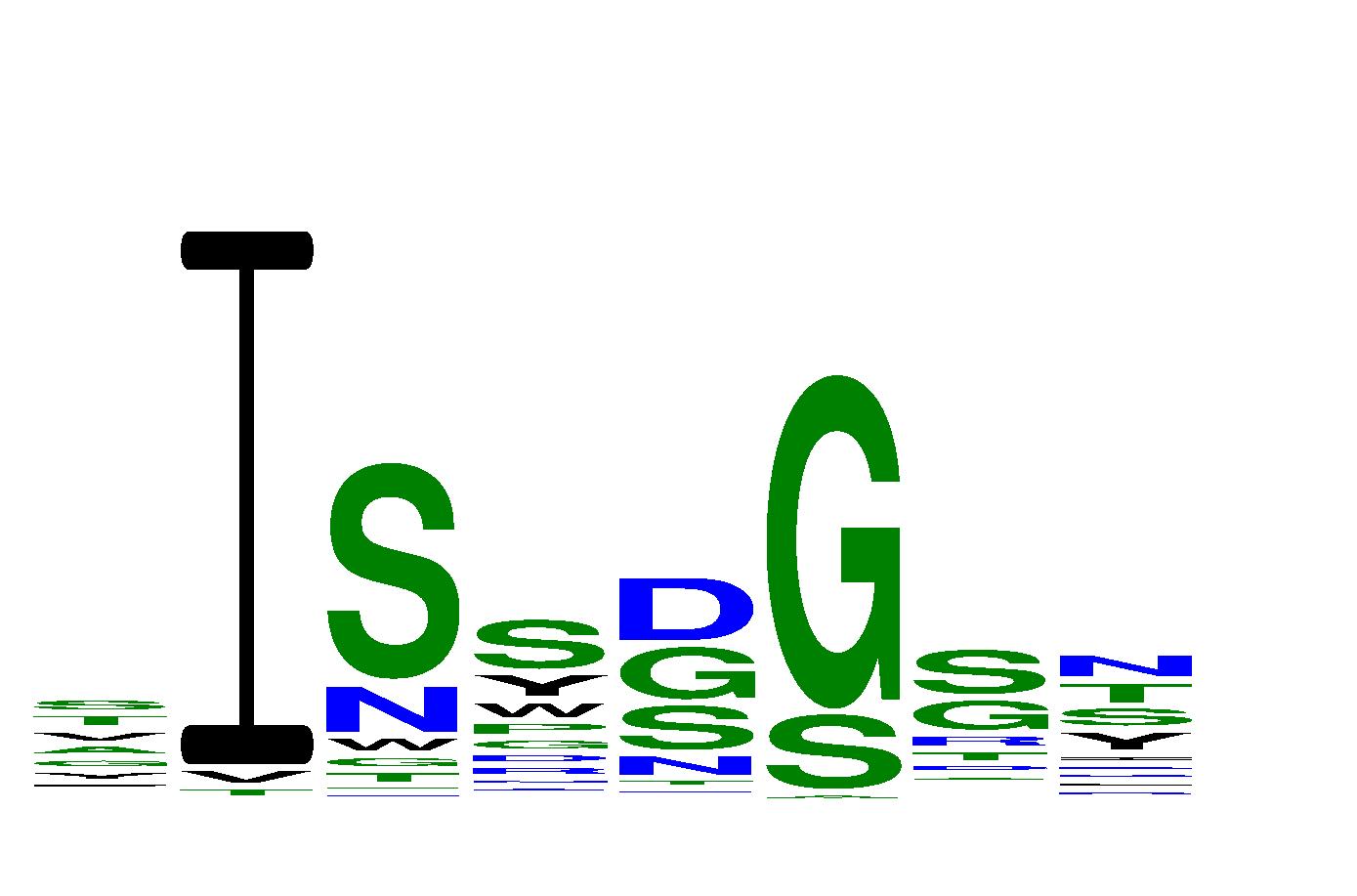


H2-8-B


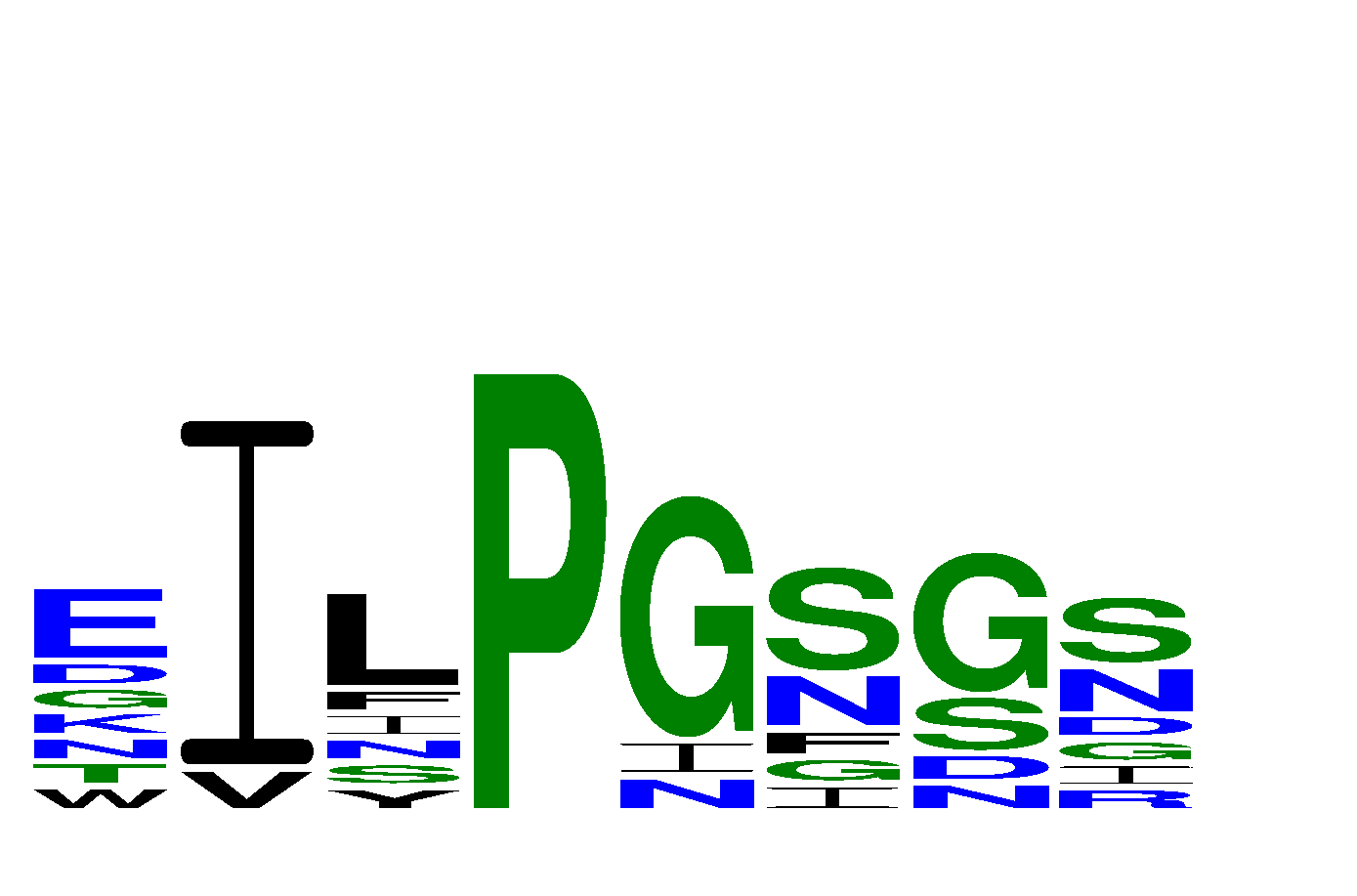


H2-8-D


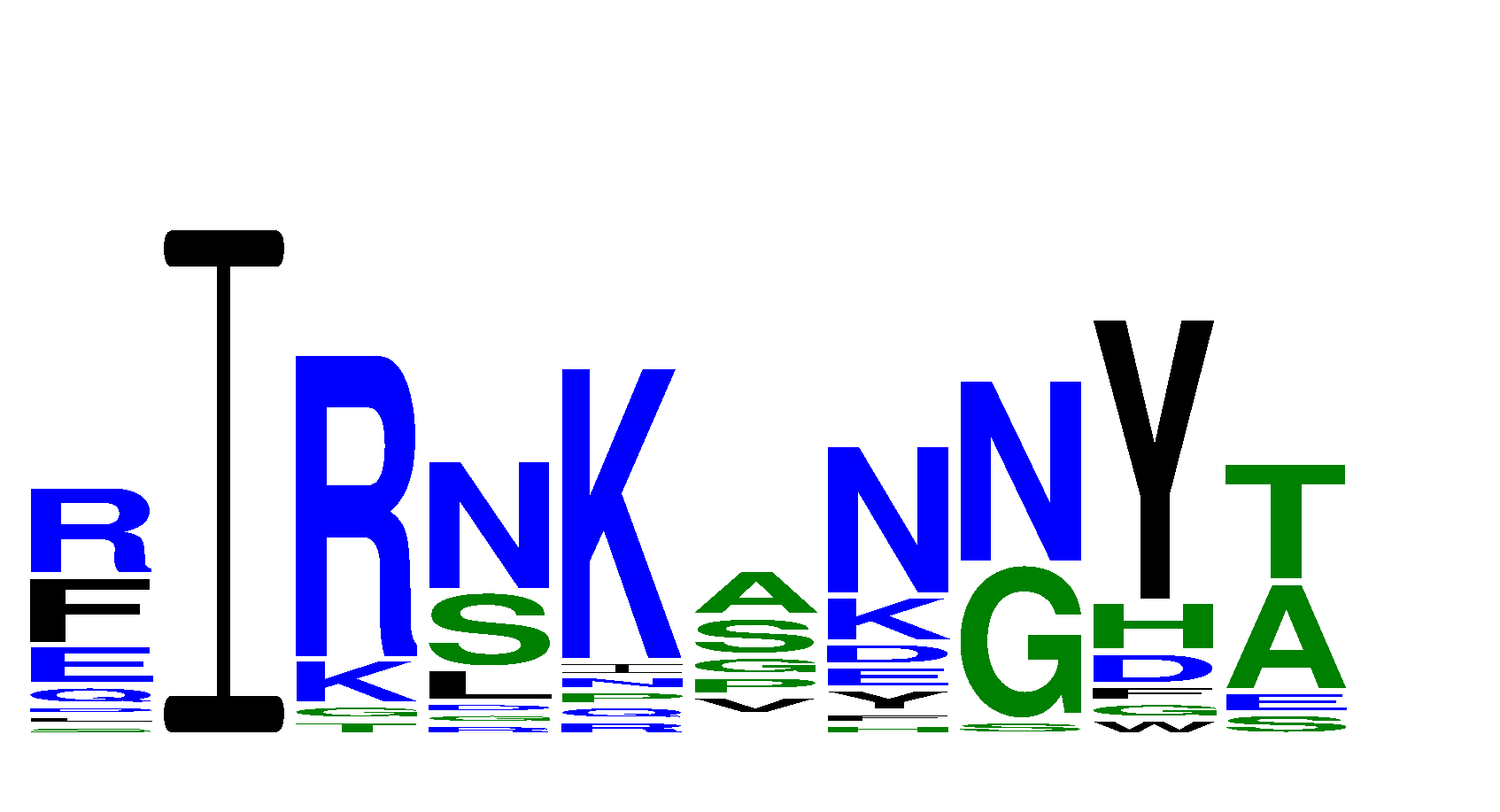


H2-10-A

Supplement: Supplemental_Datas.zip [file kmab-08-04-1158370-s001.zip › 2015MABS1071R-s12.docx]
